# Supplementary material for: Progress and inequality in child immunization in 38 African countries, 2000–2030: A spatio-temporal Bayesian analysis at national and sub-national levels
Source: PLoS Med. 2025 Jul 29;22(7):e1004664. doi: 10.1371/journal.pmed.1004664 (PMC12306735; doi:10.1371/journal.pmed.1004664)
Supplement: S1 File — Table A. Description of the included DHS surveys by countries. Table B. List of child immunization indicators with definitions and calculations. Table C. Model comparison and selection. Table D. Model validation by immunization indicators. Table E. Model validation by countries. Table F. Immunization coverage 2000–2030, and % reach global targets, at the national level. Table G. Immunization coverage 2000–2030, and % reach global targets, by wealth quintile. Table H. Immunization coverage 2000–2030, and % reach global targets, by educational level. Table I. AARC and Additional values required to reach 2030 targets, at national level. Table J. AARC and Additional values required to reach 2030 targets, by wealth quintile. Table K. AARC and Additional values required to reach 2030 targets, by educational level. Table L. Wealth-related inequality in childhood immunization coverage, 2000–2030. Table M. Education-related inequality in childhood immunization coverage, 2000–2030. Fig A1. Residual histogram for 6 comparing models. Fig A2. Predicted vs. observed values plot for 6 comparing models. Fig A3. Residual vs. fitted plot for 6 comparing models. Fig A4. Posterior Distribution of BCG immunization coverage for 6 comparing models. Fig A5. Trends in BCG immunization coverage at regional levels, using model_besag_1. Fig A6. Trends in BCG immunization coverage at regional levels, using model_besag_2. Fig A7. Trends in BCG immunization coverage at regional levels, using model_bym_1. Fig A8. Trends in BCG immunization coverage at regional levels, using model_bym_2. Fig A9. Trends in BCG immunization coverage at regional levels, using model_bym2_1. Fig A10. Trends in BCG immunization coverage at regional levels, using model_bym2_2. Fig A11. Trends in MCV1 immunization coverage at regional levels, using model_bym2_2. Fig A12. Trends in Polio3 immunization coverage at regional levels, using model_bym2_2. Fig A13. Trends in DPT3 immunization coverage at regional levels, using mod [file pmed.1004664.s001.pdf]

Supplementary

Progress and inequality in childhood immunization in 38 African countries, 2000-2030: A spatio-temporal Bayesian analysis at national and sub-national levels

Table of Contents

Table A. Description of the included DHS surveys by countries ... 3
Table B. List of child immunization indicators with definitions and calculations ... 5
Supplementary Modelling ... 6
Table C. Model comparison and selection ... 12
Table D. Model validation by immunization indicators ... 13
Table E. Model validation by countries ... 14
Table F. Immunization coverage 2000–2030, and % reach global targets, at national level ... 15
Table G. Immunization coverage 2000–2030, and % reach global targets, by wealth quintile ... 19
Table H. Immunization coverage 2000–2030, and % reach global targets, by educational level ... 35
Table I. AARC and Additional values required to reach 2030 targets, at national level ... 42
Table J. AARC and Additional values required to reach 2030 targets, by wealth quintile ... 46
Table K. AARC and Additional values required to reach 2030 targets, by educational level ... 58
Table L. Wealth-related inequality in childhood immunization coverage, 2000–2030 ... 63
Table M. Education-related inequality in childhood immunization coverage, 2000–2030 ... 69
Fig A1. Residual histogram for 6 comparing models ... 75
Fig A2. Predicted vs. observed values plot for 6 comparing models ... 76
Fig A3. Residual vs. fitted plot for 6 comparing models ... 77
Fig A4. Posterior Distribution of BCG immunization coverage for 6 comparing models ... 78
Fig A5. Trends in BCG immunization coverage at regional levels, using model_besag_1 ... 79
Fig A6. Trends in BCG immunization coverage at regional levels, using model_besag_2 ... 80
Fig A7. Trends in BCG immunization coverage at regional levels, using model_bym_1 ... 81
Fig A8. Trends in BCG immunization coverage at regional levels, using model_bym_2 ... 82
Fig A9. Trends in BCG immunization coverage at regional levels, using model_bym2_1 ... 83
Fig A10. Trends in BCG immunization coverage at regional levels, using model_bym2_2 ... 84
Fig A11. Trends in MCV1 immunization coverage at regional levels, using model_bym2_2 ... 85
Fig A12. Trends in Polio3 immunization coverage at regional levels, using model_bym2_2 ... 86
Fig A13. Trends in DPT3 immunization coverage at regional levels, using model_bym2_2 ... 87
Fig A14. Trends in Full immunization coverage at regional levels, using model_bym2_2 ... 88
Fig A15. BCG coverage, inequality indices (SII, RII), and changes over 2000–2030 (AARC) ... 89
Fig A16. MCV1 coverage, inequality indices (SII, RII), and changes over 2000–2030 (AARC) ... 90
Fig A17. Polio3 coverage, inequality indices (SII, RII), and changes over 2000–2030 (AARC) ... 91
Fig A18. DPT3 coverage, inequality indices (SII, RII), and changes over 2000–2030 (AARC) ... 92
Fig A19. (A) Wealth- and (B) Education-related inequality in BCG coverage ... 93
Fig A20. (A) Wealth- and (B) Education-related inequality in MCV1 coverage ... 94
Fig A21. (A) Wealth- and (B) Education-related inequality in Polio3 coverage ... 95
Fig A22. (A) Wealth- and (B) Education-related inequality in DPT3 coverage ... 96
Fig A23. Two-dimensional graph of changes in socioeconomic-related inequalities, 2000–2020 ... 97
Fig A24. Two-dimensional graph of changes in socioeconomic-related inequalities, 2020–2030 ... 98
Fig A25. Inequality changes in Full immunization coverage (2000–2030) with region names ... 99
Fig A26. Inequality changes in BCG immunization coverage (2000–2030) with region names ... 100
Fig A27. Inequality changes in MCV1 immunization coverage (2000–2030) with region names ... 101
Fig A28. Inequality changes in Polio3 immunization coverage (2000–2030) with region names ... 102
Fig A29. Inequality changes in DPT3 immunization coverage (2000–2030) with region names ... 103
Fig A30. Sensitivity analysis of using recent trend for projections of Full immunization coverage ... 104
Fig A31. Sensitivity analysis of using AARC of best-performing region for 2030 national projections ... 105
Fig A32. Sensitivity analysis of model selection for projections of Full immunization coverage ... 106

Table A. Description of the included DHS surveys by countries

Country	Survey Year	Number of Surveys	Records
Angola	2015	1	14,322
Benin	2001, 2006, 2011, 2017	4	48,420
Burkina Faso	2003, 2010	2	25,689
Burundi	2010, 2016	2	20,934
Cameroon	2004, 2011, 2018	3	29,590
Chad	2004, 2014	2	24,258
Comoros	2012	1	3,149
Congo Brazzaville	2005, 2011	2	14,164
Congo Democratic Republic	2007, 2013	2	27,708
Cote d'Ivoire	2011	1	7,776
Egypt	2000, 2003, 2005, 2008, 2014	5	58,699
Eswatini	2006	1	2,812
Ethiopia	2000, 2005, 2011, 2016, 2019	5	48,782
Gabon	2000, 2012	2	10,472
Gambia	2013, 2019	2	16,450
Ghana	2003, 2008, 2014	3	12,720
Guinea	2005, 2012, 2018	3	21,354
Kenya	2003, 2008, 2014	3	32,992
Lesotho	2004, 2009, 2014	3	10,834
Liberia	2007, 2013, 2019	3	19,109
Madagascar	2003, 2008	2	17,863
Malawi	2000, 2004, 2010, 2016	4	60,093
Mali	2001, 2006, 2012, 2018	4	47,601
Morocco	2003	1	6,180
Mozambique	2003, 2011	2	21,428
Namibia	2000, 2006, 2013	3	14,203
Niger	2006, 2012	2	21,751
Nigeria	2003, 2008, 2013, 2018	4	100,082
Rwanda	2000, 2005, 2008, 2010, 2014	5	38,918
Sao Tome and Principe	2008	1	1,931

Country	Survey Year	Number of Surveys	Records
Senegal	2005, 2010, 2012, 2014, 2015, 2016, 2017	7	62,819
Sierra Leone	2008, 2013, 2019	3	27,468
South Africa	2016	1	3,548
Tanzania	2004, 2010, 2015	3	26,820
Togo	2013	1	6,979
Uganda	2001, 2006, 2011, 2016	4	38,882
Zambia	2001, 2007, 2013, 2018	4	36,694
Zimbabwe	2005, 2010, 2015	3	16,941
**Total**		**104**	**1,000,435**

Table B. List of child immunization indicators with definitions and calculations

Indicator	Definition	Numerator	Denominator
BCG Immunization	The proportion of children aged 12–23 months who received one dose of the Bacillus Calmette-Guérin (BCG) vaccine.	Number of children aged 12–23 months receiving one dose of the Bacillus Calmette-Guérin (BCG) vaccine.	Total number of children aged 12–23 months surveyed.
MCV1 Immunization	The proportion of children aged 12–23 months who received at least one dose of measles-containing vaccine.	Number of children aged 12–23 months receiving one dose of measles-containing vaccine.	Total number of children aged 12–23 months surveyed.
Polio3 Immunization	The proportion of children aged 12–23 months who received three doses of polio vaccine.	Number of children aged 12–23 months receiving three doses of polio vaccine.	Total number of children aged 12–23 months surveyed.
DPT3 Immunization	The proportion of children aged 12–23 months who received three doses of diphtheria, pertussis, and tetanus vaccine.	Number of children aged 12–23 months receiving three doses of diphtheria, pertussis, and tetanus vaccine.	Total number of children aged 12–23 months surveyed.
Full Immunization	The proportion of children aged 12–23 months who received three doses of DPT and Polio vaccines and one dose of BCG and measles vaccines.	Number of children aged 12–23 months receiving three doses of DPT and Polio vaccines and one dose of BCG and measles vaccines.	Total number of children aged 12–23 months surveyed.

*Abbreviations: BCG=Bacillus Calmette–Guérin vaccine; MCV1=one dose of measles-contain vaccine; DPT=three doses of diphtheria, pertussis, and tetanus vaccine; Polio3=three doses of polio vaccine; *All definitions and measurement adapted from the global monitoring report on Universal Health Coverage.*[1]

Supplementary Modelling

Random effect models

In a simple random effects model, we estimated the coverage of childhood vaccination for each area (nation or region) assuming that the number of vaccinated children Y_i observed in area i follows the binomial distribution as

$$\boldsymbol{Y_i \sim Binomial(n_i, p_i),\ \ i = 1, \dots, n,}$$

with a vaccination rate p_i. The random effects model for the vaccination rate p_i can then be specified as

$$\boldsymbol{logit(p_i) = \alpha + u_i, \qquad u_i \sim N(0, \sigma^2)}$$

where α is the population logit vaccination rate, whose non-informative prior is assigned as

$$\boldsymbol{\alpha \sim N(0, 1/\tau), \qquad \tau = 10^{-6}}$$

and u_i is the random effect, capturing area-specific deviations from α, with the default prior for the precision $(1/\sigma^2)$ specified as

$$\boldsymbol{1/\sigma^2 \ \sim Gamma\left(1, 5 * 10^{-5}\right).}$$

Spatial small-area models

Estimates for small areas, where a small number of participants is available, can become less reliable because the number of events is exceedingly low. In such cases, it is preferable to use models that allow for borrowing information from neighboring areas and incorporating covariate information. This approach facilitates smoothing or shrinking of extreme values based on small sample sizes, ensuring more robust and reliable risk estimates.[2,3]

A simple spatial model canspecify the vaccination rate as

$$\boldsymbol{logit(p_i) = \alpha + u_i + v_i}$$

where α represents the overall vaccination rate, and u_i serves as a random effect unique to area i, capturing a spatial dependence structure with vaccination. In addition, v_i represents an unstructured exchangeable component that accounts for uncorrelated noise, $v_i \sim N(0, \sigma_v^2)$.

Similarly, a spatial model with covariates can be expressed as

$$\boldsymbol{logit(p_i) = d_i^T \beta + u_i \ + v_i}$$

where $d_i = (1,\ d_{i1}, \dots d_{ik})^T$ denotes a vector comprising of the intercept and k covariates at area i, and $\beta = (\beta_0, \beta_1, \dots \beta_k)^T$ represents the coefficient vector.

Spatio-temporal models

In our study, as the vaccination rate varies over time, a spatio-temporal model is needed to allow not only considering spatial structure but also incorporating temporal as well as spatio-temporal interactions.[4,5]

We estimated the coverage of the childhood vaccination indicators $\boldsymbol{Y_{ij}}$ for each area i (nation or region) and year j using the model proposed by Bernardinelli et al,[6] assuming a parametric time trend as follows

$$\boldsymbol{Y_{ij} \sim Binomial\left(n_{ij}, p_{ij}\right), \qquad i \ = \ 1, \dots, I, \qquad j \ = \ 1, \dots, J}$$

$$\boldsymbol{logit\left(p_{ij}\right) = \alpha + u_i \ + v_i + (\beta + \delta_i) \times t_j}$$

Here, α represents the intercept, the term $(u_i \ + v_i)$ is the random area effect, β reflects the global temporal linear trend effect, and δ_i is the interaction effect between space and time, representing the distinction between the global

trend β and the trend specific to the area i. This model accommodates individual time trends for each area, where the spatial intercept is expressed as $(\alpha + u_i + v_i)$ and the slope as $(\beta + \delta_i)$.

We expanded this model further to integrate covariates such as socioeconomic factors (wealth quintile, educational levels). We included random slope effects between these variables and areas, and interaction effects between these variables and survey year, allowing for specific spatial and temporal effects of covariates.

Spatial neighborhood structure

Spatial neighborhood structures play a crucial role in the analysis of areal data, offering insights into spatial relationships among regions. In spatial neighborhood matrices, the (i, j) −element of a matrix Wis denoted as w_{ij}, which signifies the spatial connection between areas i and j, where $i, j \in \{1, \dots n\}$.

The matrix W delineates a neighborhood structure across the entire study regions, with its elements serving as weights. Areas closer to i are assigned higher weights than those farther away. The simplest form of neighborhood matrix is the adjacent matrix whose element takes a binary value as $w_{ij} = 1$ if regions i and j share any common boundary, and $w_{ij} = 0$ otherwise.

Besag-York-Mollié (BYM) model

The Besag-York-Mollié (BYM) model introduced by Besag et al. in 1991 is a widely used spatial model in disease mapping.[7] In this model, the spatial random effect u_i follows a Conditional Autoregressive (CAR) distribution, providing a smoothing effect to the data based on a defined neighborhood structure. In general, this structure identifies areas as neighbors if they share a common boundary.

$$\boldsymbol{u_i | u_{-i} \sim N\left(\overline{u}_{\delta_i}, \frac{\sigma_u^2}{n_{\delta_i}}\right)}$$

where $\overline{u}_{\delta_i} = n_{\delta_i}^{-1} \sum_{j \in \delta_i} u_j$ represents the average of neighboring random effects, δ_i denotes the set of neighboring areas, and n_{δ_i} indicates the number of neighbors for area i. Meanwhile, the unstructured component v_i is modeled as independent and identically distributed (iid) normal variables with zero mean and variance σ_v^2.

BYM2 model

The model can be updated using a novel parametrization of the BYM model known as BYM2, introduced by Simpson et al (2017).[8] In the BYM2 model, a scaled spatially structured component $\boldsymbol{u}_*$ and an unstructured component $\boldsymbol{v}_*$ are employed as

$$\boldsymbol{b = \frac{1}{\sqrt{\tau_b}}\left(\sqrt{1-\phi}\, v_* + \sqrt{\phi}\, u_*\right)}$$

In this context, the precision parameter $\tau_b > 0$ governs the influence of the weighted sum of $\boldsymbol{u}_*$ and $\boldsymbol{v}_*$ on the overall marginal variance. Meanwhile, the mixing parameter ϕ, ranging from 0 to 1, quantifies the proportion of the marginal variance attributed to the structured effect $\boldsymbol{u}_*$.

As in the BYM2, spatial random effect b_i replaces the traditional spatial term $u_i + v_i$, the updated model with BYM2 is:

$$\boldsymbol{logit(p_{ij}) = \alpha + b_i + (\beta + \delta_i) \times t_j}$$

where b_i now incorporates both structured (spatially correlated) and unstructured (iid) components using BYM2.

Model Descriptions

We applied six hierarchical spatio-temporal models to estimate childhood immunization coverage across countries, regions, and years, incorporating spatial dependencies, temporal trends, and socioeconomic group effects. These models share a common structure and progressively increase in complexity.

Let $\boldsymbol{Y_{ijtk}}$ represents the number of immunized children in nation I, region j, year t, and socioeconomic group t, modelled as a binomial outcome based on the number of surveyed children $\boldsymbol{n_{ijtk}}$ and the underlying immunization probability $\boldsymbol{p_{ijtk}}$. The logit of the immunization probability is modeled using the following common components:

- α: Global intercept
- $\beta_1 Year$: Global linear temporal trends
- $\beta_2 Group$: Fixed effect for socioeconomic group (e.g., wealth quintile or education level).
- $\beta_3(Year \times Group)$: Interaction term to capture subgroup-specific trends over time.
- ψ_{ijk}: Random effect capturing unstructured heterogeneity in socioeconomic group across spatial areas.
- ξ_{it}: Country-year–specific unstructured random effect
- ζ_{ijt}: Region-year–specific unstructured random effect

The five models differ in their specification of spatial structure:

- Models 1 and 2 use structured spatial effects via intrinsic conditional autoregressive (ICAR) models (Besag model).
- Models 3 and 4 incorporate both structured and unstructured spatial effects following the Besag-York-Mollié (BYM) framework.
- Models 5 and 6 employ a reparameterised BYM2 framework, which improves spatial modelling by balancing structured and unstructured components with a mixing parameter.

Below, we provide the detailed formulation for each model, highlighting the structure of national- and regional-level spatial effects.

Model 1: model_besag_1

This model is a spatio-temporal hierarchical Besag model that accounts for both nation-level and region-level spatial structures while incorporating random temporal effects. The nation-level and region-level spatial effects follow separate Besag conditional autoregressive (CAR) models, assuming they are independent.

Now Y_{ijtk} represents coverage of a childhood immunization indicator in nation i, region j, year t, and subgroup k (in which, subgroup is either wealth quintile or educational level)

$$\boldsymbol{Y_{ijtk} \sim Binomial(n_{ijtk}, p_{ijtk})}$$

$$\boldsymbol{logit(p_{ijtk}) = \alpha + u_{(nat)i} + u_{(reg)ij} + \beta_1 Year + \beta_2 Group + \beta_3 (Year \times Group) + \psi_{ijk} + \xi_{it} + \zeta_{ijt}}$$

where:

- $u_{(nat)i}$ represents nation-level structured spatial effect, follows a Besag CAR model based on the national adjacency matrix $g_{(nat)}$

$$\boldsymbol{u_{(nat)i} \sim CAR(g_{nat}, \sigma^2_{u(nat)})}$$

$$\boldsymbol{u_{(nat)_i} | u_{(nat)-i} \sim N\left(n_{\delta_i}^{-1} \sum_{z \in \delta_i} u_{(nat)z}, \frac{\sigma^2_{u(nat)}}{n_{\delta_i}}\right)}$$

  - with δ_i denotes the set of neighboring nations, and
  - n_{δ_i} indicates the number of neighbors for nation i

- $u_{(reg)ij}$, similarly, represents region-level structured spatial effect, follows a separate Besag CAR model, using the regional adjacency matrix $g_{(reg)}$

$$\boldsymbol{u_{(reg)ij} \sim CAR(g_{reg}, \sigma^2_{u(reg)})}$$

$$\boldsymbol{u_{(reg)ij} | u_{(reg)-ij} \sim N\left(n^{-1}_{\delta_{ij}} \sum_{z \in \delta_{ij}} u_{(reg)z}, \frac{\sigma^2_{u(reg)}}{n_{\delta_{ij}}}\right)}$$

  - with δ_{ij} denotes the set of neighboring regions, and
  - $n_{\delta_{ij}}$ indicates the number of neighbors for region ij

- $\beta_1 Year$ represents fixed temporal trend or the global linear time trend.
- $\beta_2 Group$ represents fixed effect of socioeconomic group (e.g., wealth quintile or educational level) or global socioeconomic group effects.
- $\beta_3(Year \times Group)$ represents interaction terms between temporal and socioeconomic group.
- ψ_{ijk} represents random effect of socioeconomic group within each region (iid random effect).

$$\boldsymbol{\psi_{ijk} \sim N(0, \sigma^2_{\psi})}$$

- ξ_{it} represents nation-specific spatio-temporal effect (iid random effect).

$$\boldsymbol{\xi_{it} \sim N(0, \sigma^2_{\xi})}$$

- ζ_{ijt} represents region-specific spatio-temporal effect (iid random effect).

$$\boldsymbol{\zeta_{it} \sim N(0, \sigma^2_{\zeta})}$$

Model 2: model_besag_2

This model extends the Model 1 by employing a nested structured spatial effect, where structured region-level effects are nested within the nation-level effects, provides a more realistic spatial dependency structure.

$$\boldsymbol{logit(p_{ijtk}) = \alpha + u^*_{(reg)ij} + \beta_1 Year + \beta_2 Group + \beta_3(Year \times Group) + \psi_{ijk} + \xi_{it} + \zeta_{ijt}}$$

- where $u^*_{(reg)ij}$ represents nested region-level structured spatial effect, follows an intrinsic Conditional Autoregressive (ICAR) model based on the regional adjacency matrix $g_{(reg)}$, and conditionally linked to the nation-level spatial effect $u_{(nat)i}$, which is modeled using the national adjacency matrix $g_{(nat)}$.

$$\boldsymbol{u^*_{(reg)ij} \sim u_{(reg)ij} | u_{(reg)-ij}, u_{(nat)i} \sim N\left(n^{-1}_{\delta_{ij}} \sum_{z \in \delta_{ij}} u_{(reg)z} + u_{(nat)i}, \frac{\sigma^2_{u(reg)}}{n_{\delta_{ij}}}\right)}$$

  - with δ_{ij} denotes the set of neighboring regions, and
  - $n_{\delta_{ij}}$ indicates the number of neighbors for region ij
  - The term $u_{(nat)i}$ ensures that region-level spatial variability is conditioned on the corresponding nation-level spatial effect.

Model 3: model_bym_1

This model extends the Besag model to Besag-York-Mollié (BYM) model by incorporating unstructured spatial effects (iid terms) for region-level.

$$\boldsymbol{logit(p_{ijtk}) = \alpha + u_{(reg)ij} + v_{(reg)ij} + \beta_1 Year + \beta_2 Group + \beta_3(Year \times Group) + \psi_{ijk} + \xi_{it} + \zeta_{ijt}}$$

where:

- $u_{(reg)ij}$, represents region-level structured spatial effect, modelled with BYM, using the regional adjacency matrix $g_{(reg)}$

$$\boldsymbol{u_{(reg)ij} \sim CAR(g_{reg}, \sigma^2_{u(reg)})}$$

$$\boldsymbol{u_{(reg)ij}|u_{(reg)-ij} \sim N\left(n_{\delta_{ij}}^{-1}\sum_{z\in\delta_{ij}} u_{(reg)z}, \frac{\sigma^2_{u_{(reg)}}}{n_{\delta_{ij}}}\right)}$$

- with δ_{ij} denotes the set of neighboring regions, and
- $n_{\delta_{ij}}$ indicates the number of neighbors for region ij

- $v_{(reg)ij}$ represents the unstructured spatial effect (iid), capturing random effects at the region ij

$$\boldsymbol{v_{(reg)ij} \sim N(0, \sigma^2_{v_{(reg)}})}$$

Model 4: model_bym_2

This model extends the Model 3 by nesting regional spatial effects within national-level spatial structure. This structure allows each region's pattern to be influenced not only by nearby regions but also by the spatial structure across countries.

$$\boldsymbol{logit(p_{ijtk}) = \alpha + u^*_{(reg)ij} + v^*_{(reg)ij} + \beta_1 Year + \beta_2 Group + \beta_3(Year \times Group) + \psi_{ijk} + \xi_{it} + \zeta_{ijt}}$$

- Where $u^*_{(reg)ij}$ represents the nested structured spatial effect at the region level, modelled using an intrinsic Conditional Autoregressive (ICAR) model based on the regional adjacency matrix $g_{(reg)}$, and conditionally linked to the nation-level spatial effect $u_{(nat)i}$, which is modeled using the national adjacency matrix $g_{(nat)}$.
- $v^*_{(reg)ij}$ represents the nested unstructured spatial effect (iid), capturing random effects at the region j within nation i

$$\boldsymbol{v^*_{(reg)ij} \sim v_{(reg)ij}|\, v_{(nat)i} \sim N(0, \sigma^2_{v_{(reg)}})}$$

Model 5: model_bym2_1

This model employs the Besag-York-Mollié 2 (BYM2) model, an improved version of the original BYM model. This approach enhances spatial modeling by reparameterizing the structured (CAR) and unstructured (iid) components at regional level.

$$\boldsymbol{logit(p_{ijtk}) = \alpha + b_{(reg)ij} + \beta_1 Year + \beta_2 Group + \beta_3(Year \times Group) + \psi_{ijk} + \xi_{it} + \zeta_{ijt}}$$

where:

- $b_{(reg)ij}$ represents the region-level spatial effect, modeled with BYM2, using the regional adjacency matrix $g_{(reg)}$

$$\boldsymbol{b_{(reg)ij} = \frac{1}{\sqrt{\tau_{b_{(reg)}}}}\left(\sqrt{1-\phi_{(reg)}}\,v_{(reg)} + \sqrt{\phi_{(reg)}}\,u_{(reg)}\right)}$$

- with $\tau_{b_{(reg)}}$ represents the precision parameter controlling the total variance of $b_{(reg)i}$
- $\phi_{(reg)}$ represens the mixing parameter ($0 \le \phi \le 1$) that determines the balance between structured $u_{(reg)}$ and unstructured $v_{(reg)}$

Model 6: model_bym2_2

This model extends Model 5 by nesting regional spatial effects within national-level spatial structure. This structure allows each region's pattern to be influenced not only not only from its neighboring regions (within the same country) but also from the national-level spatial structure.

$$\boldsymbol{logit(p_{ijtk}) = \alpha + b^*_{(reg)ij} + \beta_1 Year + \beta_2 Group + \beta_3(Year \times Group) + \psi_{ijk} + \xi_{it} + \zeta_{ijt}}$$

where:

- $b^*_{(reg)ij}$: regional spatial effect (BYM2) nested within national structure, using the regional adjacency matrix $g_{(reg)}$ and national adjacency matrix $g_{(nat)}$.

$$\boldsymbol{b^*_{(reg)ij}} = \frac{\mathbf{1}}{\sqrt{\boldsymbol{\tau_{b_{(reg)}}}}}\left(\sqrt{\mathbf{1}-\boldsymbol{\phi_{(reg)}}}\boldsymbol{v^*_{(reg)}} + \sqrt{\boldsymbol{\phi_{(reg)}}}\boldsymbol{u^*_{(reg)}}\right)$$

- $u^*_{(reg)ij}$: nested structured spatial effect at the region level, modelled using an intrinsic Conditional Autoregressive (ICAR) model based on the regional adjacency matrix $g_{(reg)}$, and conditionally linked to the nation-level spatial effect $u_{(nat)i}$, which is modeled using the national adjacency matrix $g_{(nat)}$.
- $v^*_{(reg)ij}$: nested unstructured spatial effect (iid), capturing random effects at the region j within nation i

Table C. Model comparison and selection

Model	Fit Indices		Absolute Difference (%)			Mean Error (%)			Run Time (s)
	DIC	WAIC	In-sample	Out-of-sample	Full-period	In-sample	Out-of-sample	Full-period	
BCG_model_besag_1	4,358	4,658	1.85	5.77	2.28	-0.12	1.75	0.08	281.66
BCG_model_besag_2	4,392	5,953	1.78	6.70	2.33	-0.04	0.62	0.03	278.33
BCG_model_bym_1	4,363	4,655	1.73	5.80	2.18	-0.13	1.65	0.07	365.72
BCG_model_bym_2	4,336	4,600	1.56	6.00	2.06	-0.17	1.26	-0.02	360.30
BCG_model_bym2_1	4,363	4,655	1.73	5.80	2.18	-0.13	1.64	0.07	474.34
BCG_model_bym2_2	4,336	4,600	1.57	6.00	2.06	-0.17	1.27	-0.01	293.57
MCV1_model_besag_1	4,113	7,658	3.33	11.18	4.20	-0.01	7.99	0.88	234.32
MCV1_model_besag_2	3,813	8,911	3.25	11.17	4.13	0.13	7.24	0.92	169.44
MCV1_model_bym_1	4,096	7,649	3.13	11.14	4.02	0.00	7.89	0.87	393.42
MCV1_model_bym_2	4,055	7,662	2.91	11.30	3.84	-0.01	7.86	0.86	320.39
MCV1_model_bym2_1	4,096	7,649	3.13	11.14	4.02	0.00	7.89	0.87	375.36
MCV1_model_bym2_2	4,056	7,663	2.91	11.30	3.84	-0.01	7.86	0.86	264.80
Polio3_model_besag_1	4,887	7,311	2.48	8.57	3.15	-0.07	4.97	0.49	295.13
Polio3_model_besag_2	4,995	7,947	2.33	9.18	3.09	0.02	3.92	0.45	147.36
Polio3_model_bym_1	4,875	7,241	2.34	8.59	3.03	-0.07	4.90	0.49	367.15
Polio3_model_bym_2	4,854	7,179	2.11	8.74	2.85	-0.08	4.77	0.46	324.94
Polio3_model_bym2_1	4,875	7,240	2.34	8.59	3.03	-0.07	4.89	0.48	554.15
Polio3_model_bym2_2	4,854	7,180	2.11	8.74	2.85	-0.08	4.77	0.46	240.21
DPT3_model_besag_1	4,853	7,845	2.83	7.61	3.36	-0.01	2.75	0.30	264.34
DPT3_model_besag_2	4,852	8,689	2.67	8.95	3.36	0.10	1.57	0.26	145.92
DPT3_model_bym_1	4,821	7,851	2.69	7.72	3.25	0.00	2.69	0.30	321.86
DPT3_model_bym_2	4,761	7,738	2.44	8.10	3.06	-0.01	2.68	0.29	327.94
DPT3_model_bym2_1	4,821	7,850	2.69	7.72	3.24	0.00	2.69	0.30	394.94
DPT3_model_bym2_2	4,761	7,739	2.44	8.10	3.07	-0.01	2.68	0.29	240.22
Full_model_besag_1	4,304	9,165	3.33	10.65	4.14	0.01	5.32	0.60	249.05
Full_model_besag_2	4,137	10,457	3.18	11.28	4.08	0.15	4.43	0.63	159.12
Full_model_bym_1	4,257	9,004	3.12	10.62	3.96	0.03	5.27	0.61	268.72
Full_model_bym_2	4,203	9,055	2.89	10.83	3.77	0.03	5.27	0.61	319.74
Full_model_bym2_1	4,256	9,014	3.12	10.61	3.96	0.03	5.27	0.61	381.93
Full_model_bym2_2	4,203	9,056	2.89	10.83	3.77	0.03	5.27	0.61	250.63

Notes: DIC = Deviance Information Criterion; WAIC = Watanabe-Akaike Information Criterion; AD = Absolute Difference.
Model comparison and selection were conducted using data from 2000 to 2016, with 2017–2019 held out for out-of-sample validation. Models were evaluated based on in-sample (2000–2016), out-of-sample (2017–2019), and full-period (2000–2019) performance.
The final model selected was model_bym2_2. Both model_bym_2 and model_bym2_2 demonstrated similar performance in terms of DIC, WAIC, average absolute difference, and mean error. Both incorporated a nested spatial structure, enhancing interpretability of national and regional spatial effects in a multi-country hierarchical context. Model_bym2_2 was ultimately chosen for its superior computational efficiency.

Table D. Model validation by immunization indicators

Indicators	Models	Average AD (%)	Mean Error (%)
Full immunization	bym2_2	3.4	0.12
BCG immunization	bym2_2	1.9	0.01
MCV1 immunization	bym2_2	3.5	0.17
Polio3 immunization	bym2_2	2.7	0.11
DPT3 immunization	bym2_2	2.8	0.12

Notes: AD=Absolute Difference

Table E. Model validation by countries

Country Names	In-sample Validation		FIC Error Check	
	Average AD (%)	Mean Error (%)	Proportion (%)	Magnitude (%)
Angola	0.0	0.0	0.0	–
Benin	3.0	-0.9	0.0	–
Burkina Faso	1.6	0.0	0.7	0
Burundi	0.4	0.0	0.0	–
Cameroon	3.1	0.3	0.0	–
Chad	0.6	0.0	0.0	–
Comoros	0.2	0.1	0.0	–
Congo Brazzaville	1.0	0.1	0.0	–
Congo Democratic Republic	1.2	0.0	0.3	1.4
Cote d'Ivoire	0.0	0.0	0.0	–
Egypt	1.3	-0.1	1.8	2.3
Eswatini	0.0	0.0	0.0	–
Ethiopia	4.2	0.4	0.3	2.9
Gabon	0.7	0.1	2.6	3.5
Gambia	0.8	-0.1	0.0	–
Ghana	2.6	-0.4	0.0	–
Guinea	4.4	-0.1	0.0	–
Kenya	2.8	-0.1	0.0	–
Lesotho	3.7	-0.1	0.0	–
Liberia	5.0	0.9	0.0	–
Madagascar	2.6	0.9	0.0	–
Malawi	2.5	0.3	0.0	–
Mali	5.7	0.4	2.4	3.1
Morocco	0.5	-0.5	0.0	–
Mozambique	1.2	0.0	0.0	–
Namibia	4.0	0.1	0.0	–
Niger	0.9	0.1	3.2	4.2
Nigeria	3.4	-0.5	0.0	–
Rwanda	10.0	2.1	3.7	2.6
Sao Tome and Principe	0.5	-0.2	0.0	–
Senegal	2.0	0.0	2.4	1.7
Sierra Leone	4.1	0.8	1.6	2.3
South Africa	0.0	0.0	0.0	–
Tanzania	1.0	0.0	0.0	–
Togo	0.0	0.0	0.0	–
Uganda	1.8	0.1	0.0	–
Zambia	3.0	-0.2	0.0	–
Zimbabwe	2.8	0.0	0.0	–
**Overall**	**2.9**	**0.1**	**0.8**	**2.6**

Notes: This table evaluates internal consistency by comparing modelled full immunization coverage (FIC) estimates with each component vaccine (BCG, MCV1, Polio3, and DPT3). Instances where FIC exceeded any component were identified. Across all countries, regions, and time points, fewer than 1% of estimates showed minor exceedances, typically ⩽3 percentage points. These discrepancies likely reflect survey sampling variability, reporting errors, or data inconsistencies. Given their rarity and small magnitude, they were considered to have minimal impact on study conclusions.

Table F. Immunization coverage 2000–2030, and % reach global targets, at national level

Indicators	Country	Estimates and Projections of Immunization Coverage (%)			Probability Achieving Targets (%)	
		Year 2000	Year 2020	Year 2030	UHC2030	IA2030
Full immunization	Angola	33.2 (12.2 to 64.0)	32.7 (24.0 to 42.9)	32.5 (11.8 to 63.4)	0.1	0.0
	Benin	53.2 (51.0 to 55.4)	64.7 (62.6 to 66.8)	70.0 (66.4 to 73.3)	0.0	0.0
	Burkina Faso	28.9 (26.1 to 31.8)	98.4 (97.9 to 98.8)	99.9 (99.8 to 99.9)	100.0	100.0
	Burundi	90.9 (86.3 to 94.0)	88.7 (86.3 to 90.8)	87.5 (80.5 to 92.3)	98.1	17.9
	Cameroon	52.8 (49.8 to 55.8)	58.8 (56.4 to 61.2)	61.7 (57.2 to 66.1)	0.0	0.0
	Chad	9.2 (7.2 to 11.7)	39.7 (35.7 to 43.9)	62.7 (53.8 to 70.9)	0.0	0.0
	Comoros	50.0 (23.9 to 76.1)	74.5 (56.9 to 86.6)	83.3 (46.5 to 96.6)	59.9	24.9
	Congo Brazzaville	59.8 (53.8 to 65.5)	39.9 (33.1 to 47.0)	30.7 (20.4 to 43.2)	0.0	0.0
	Congo Democratic Republic	23.3 (19.5 to 27.5)	68.3 (64.2 to 72.1)	85.2 (79.8 to 89.3)	97.0	0.9
	Cote d'Ivoire	41.1 (20.2 to 65.9)	63.1 (42.4 to 79.8)	72.7 (31.4 to 93.9)	32.3	8.5
	Egypt	92.4 (91.6 to 93.2)	82.1 (79.8 to 84.2)	73.8 (68.4 to 78.5)	0.4	0.0
	Eswatini	83.3 (71.0 to 91.0)	95.5 (81.6 to 99.1)	97.8 (75.6 to 99.8)	96.2	88.1
	Ethiopia	14.4 (13.2 to 15.6)	48.7 (46.7 to 50.8)	69.4 (66.0 to 72.5)	0.0	0.0
	Gabon	17.9 (15.5 to 20.5)	48.0 (42.4 to 53.7)	65.6 (56.3 to 73.8)	0.0	0.0
	Gambia	75.8 (65.8 to 83.7)	87.2 (85.2 to 88.9)	90.9 (86.4 to 94.0)	100.0	67.5
	Ghana	70.6 (66.4 to 74.6)	85.6 (82.3 to 88.3)	90.3 (85.8 to 93.5)	100.0	55.9
	Guinea	49.2 (45.1 to 53.2)	28.5 (26.1 to 31.0)	20.3 (16.8 to 24.4)	0.0	0.0
	Kenya	54.2 (50.7 to 57.7)	87.3 (85.7 to 88.7)	94.3 (92.8 to 95.5)	100.0	100.0
	Lesotho	67.4 (62.3 to 72.1)	74.6 (69.1 to 79.4)	77.8 (68.1 to 85.2)	30.0	0.0
	Liberia	41.2 (36.4 to 46.1)	62.1 (59.2 to 64.9)	71.5 (66.1 to 76.4)	0.0	0.0
	Madagascar	48.4 (43.6 to 53.2)	81.3 (74.5 to 86.7)	90.4 (82.6 to 94.9)	99.3	54.9
	Malawi	70.8 (69.2 to 72.3)	89.3 (88.2 to 90.3)	94.0 (92.8 to 94.9)	100.0	100.0
	Mali	36.6 (34.9 to 38.3)	66.6 (64.6 to 68.5)	78.7 (76.0 to 81.2)	16.0	0.0
	Morocco	86.6 (81.4 to 90.5)	96.5 (80.2 to 99.5)	98.3 (73.2 to 99.9)	95.7	88.6
	Mozambique	62.2 (59.0 to 65.2)	74.5 (70.1 to 78.4)	79.5 (72.8 to 85.0)	44.1	0.0
	Namibia	68.6 (65.7 to 71.3)	80.7 (76.9 to 84.1)	85.3 (79.8 to 89.5)	97.1	1.3
	Niger	15.0 (12.4 to 18.0)	80.3 (76.3 to 83.8)	95.1 (92.6 to 96.8)	100.0	100.0
	Nigeria	17.5 (16.2 to 19.0)	43.9 (42.5 to 45.3)	60.0 (57.0 to 62.9)	0.0	0.0
	Rwanda	67.7 (65.5 to 69.8)	91.1 (89.6 to 92.4)	95.8 (94.4 to 96.8)	100.0	100.0
	Sao Tome and Principe	66.7 (45.5 to 82.5)	88.8 (68.7 to 96.7)	94.1 (60.9 to 99.4)	88.0	68.6
	Senegal	53.2 (50.2 to 56.2)	84.9 (83.7 to 86.0)	92.6 (91.2 to 93.8)	100.0	100.0
	Sierra Leone	44.0 (39.1 to 49.0)	70.7 (68.5 to 72.8)	80.9 (77.1 to 84.2)	68.1	0.0
	South Africa	47.5 (17.9 to 79.4)	61.2 (51.4 to 70.0)	67.5 (36.6 to 88.0)	15.2	1.1
	Tanzania	72.1 (69.0 to 75.0)	82.1 (79.7 to 84.2)	85.9 (82.1 to 89.0)	99.8	0.4
	Togo	50.5 (24.1 to 76.4)	78.4 (65.7 to 87.3)	87.2 (59.9 to 96.9)	75.8	35.9
	Uganda	39.0 (36.8 to 41.2)	72.1 (70.2 to 74.0)	83.9 (81.5 to 86.1)	99.9	0.0
	Zambia	67.7 (65.3 to 69.9)	81.3 (79.8 to 82.8)	86.3 (84.0 to 88.3)	100.0	0.0
	Zimbabwe	42.4 (37.8 to 47.2)	84.1 (81.2 to 86.6)	93.4 (90.7 to 95.4)	100.0	99.1
BCG immunization	Angola	66.2 (32.3 to 89.1)	73.6 (63.2 to 81.8)	76.9 (44.2 to 93.2)	39.6	8.2
	Benin	88.9 (87.5 to 90.2)	87.8 (86.3 to 89.2)	87.2 (84.1 to 89.8)	100.0	1.5
	Burkina Faso	67.4 (63.2 to 71.3)	99.7 (99.5 to 99.8)	100.0 (99.9 to 100.0)	100.0	100.0
	Burundi	98.7 (96.9 to 99.5)	97.7 (96.5 to 98.5)	97.0 (91.8 to 98.9)	100.0	99.1
	Cameroon	85.8 (83.5 to 87.8)	87.0 (85.3 to 88.5)	87.6 (84.3 to 90.3)	100.0	3.9
	Chad	33.2 (29.2 to 37.5)	70.2 (67.1 to 73.0)	83.6 (79.4 to 87.2)	95.4	0.0
	Comoros	74.1 (44.7 to 91.2)	90.5 (79.6 to 95.8)	94.5 (71.4 to 99.1)	93.3	75.0
	Congo Brazzaville	85.4 (79.7 to 89.6)	97.2 (95.3 to 98.3)	98.8 (97.0 to 99.5)	100.0	100.0
	Congo Democratic Republic	54.4 (48.1 to 60.5)	91.5 (89.6 to 93.1)	97.0 (95.4 to 98.1)	100.0	100.0
	Cote d'Ivoire	71.9 (45.6 to 88.8)	89.7 (77.3 to 95.6)	94.1 (69.4 to 99.1)	92.1	72.3
	Egypt	98.8 (98.4 to 99.1)	98.8 (98.2 to 99.3)	98.9 (97.6 to 99.5)	100.0	100.0
	Eswatini	95.2 (89.4 to 97.9)	99.2 (95.4 to 99.9)	99.7 (94.1 to 100.0)	99.8	98.9
	Ethiopia	49.1 (47.2 to 51.0)	75.3 (73.7 to 76.9)	84.5 (82.4 to 86.3)	100.0	0.0
	Gabon	89.0 (86.8 to 90.9)	93.0 (90.2 to 95.0)	94.4 (90.4 to 96.8)	100.0	98.2
	Gambia	96.6 (89.8 to 99.0)	99.2 (98.7 to 99.5)	99.6 (98.7 to 99.9)	100.0	100.0
	Ghana	89.1 (85.6 to 91.8)	98.3 (97.2 to 99.0)	99.3 (98.5 to 99.7)	100.0	100.0
	Guinea	83.3 (80.3 to 85.9)	74.6 (72.0 to 77.0)	69.3 (63.3 to 74.6)	0.0	0.0
	Kenya	84.2 (80.9 to 87.0)	98.5 (98.0 to 98.9)	99.6 (99.3 to 99.7)	100.0	100.0
	Lesotho	94.3 (91.2 to 96.4)	98.1 (96.4 to 99.0)	98.9 (96.6 to 99.7)	100.0	100.0
	Liberia	66.1 (59.5 to 72.1)	94.6 (93.3 to 95.6)	98.1 (97.1 to 98.8)	100.0	100.0
	Madagascar	64.0 (58.9 to 68.9)	95.6 (93.1 to 97.2)	98.7 (97.1 to 99.4)	100.0	100.0

Indicators	Country	Estimates and Projections of Immunization Coverage (%)			Probability Achieving Targets (%)	
		Year 2000	Year 2020	Year 2030	UHC2030	IA2030
	Malawi	91.2 (90.2 to 92.2)	98.4 (98.0 to 98.8)	99.4 (99.1 to 99.6)	100.0	100.0
	Mali	69.5 (67.7 to 71.2)	86.7 (85.2 to 88.0)	91.7 (90.0 to 93.0)	100.0	97.6
	Morocco	97.7 (96.0 to 98.7)	99.6 (97.1 to 100.0)	99.9 (96.3 to 100.0)	99.8	99.4
	Mozambique	85.6 (83.1 to 87.8)	94.2 (92.0 to 95.8)	96.4 (93.8 to 97.9)	100.0	100.0
	Namibia	91.1 (89.2 to 92.7)	96.6 (94.8 to 97.7)	97.9 (95.8 to 98.9)	100.0	100.0
	Niger	38.3 (33.2 to 43.8)	95.4 (94.0 to 96.6)	99.2 (98.6 to 99.5)	100.0	100.0
	Nigeria	36.8 (34.8 to 38.8)	66.7 (65.4 to 67.9)	78.8 (76.7 to 80.6)	10.1	0.0
	Rwanda	95.5 (94.5 to 96.4)	98.5 (97.8 to 99.0)	99.1 (98.3 to 99.6)	100.0	100.0
	Sao Tome and Principe	92.1 (80.8 to 96.9)	98.6 (94.2 to 99.7)	99.4 (93.1 to 100.0)	99.8	98.8
	Senegal	90.2 (88.1 to 92.0)	96.4 (95.7 to 97.0)	97.9 (97.0 to 98.5)	100.0	100.0
	Sierra Leone	58.8 (50.1 to 67.0)	97.9 (97.3 to 98.4)	99.6 (99.4 to 99.8)	100.0	100.0
	South Africa	81.9 (48.6 to 95.7)	93.5 (89.7 to 95.9)	96.2 (85.8 to 99.0)	99.4	92.4
	Tanzania	88.8 (86.1 to 91.0)	97.7 (96.9 to 98.3)	99.0 (98.2 to 99.4)	100.0	100.0
	Togo	85.9 (62.4 to 95.7)	97.5 (94.8 to 98.8)	99.0 (94.6 to 99.8)	100.0	99.6
	Uganda	78.1 (75.8 to 80.2)	98.0 (97.5 to 98.4)	99.5 (99.2 to 99.6)	100.0	100.0
	Zambia	92.1 (90.6 to 93.4)	96.7 (95.9 to 97.3)	97.9 (97.0 to 98.5)	100.0	100.0
	Zimbabwe	65.9 (60.4 to 71.0)	94.5 (92.8 to 95.8)	98.1 (96.8 to 98.8)	100.0	100.0
MCV1 immunization	Angola	56.7 (28.3 to 81.0)	56.3 (46.2 to 65.9)	56.0 (28.1 to 80.9)	3.1	0.1
	Benin	62.7 (60.5 to 64.8)	69.5 (67.5 to 71.5)	72.6 (69.1 to 75.9)	0.0	0.0
	Burkina Faso	39.1 (35.8 to 42.4)	98.6 (98.1 to 98.9)	99.9 (99.8 to 99.9)	100.0	100.0
	Burundi	93.1 (88.7 to 95.9)	94.3 (92.6 to 95.6)	94.8 (90.5 to 97.2)	100.0	98.3
	Cameroon	67.3 (64.4 to 70.0)	66.9 (64.6 to 69.2)	66.8 (62.2 to 71.0)	0.0	0.0
	Chad	14.7 (12.2 to 17.6)	75.7 (72.9 to 78.4)	93.0 (90.7 to 94.8)	100.0	99.4
	Comoros	63.5 (34.5 to 85.2)	81.7 (66.3 to 91.0)	87.8 (54.2 to 97.7)	74.1	40.1
	Congo Brazzaville	58.3 (51.9 to 64.4)	84.7 (80.0 to 88.4)	91.6 (85.9 to 95.2)	100.0	74.3
	Congo Democratic Republic	52.5 (46.8 to 58.2)	79.6 (76.3 to 82.6)	88.0 (83.2 to 91.6)	99.9	15.3
	Cote d'Ivoire	55.1 (31.8 to 76.4)	71.5 (52.9 to 84.9)	78.2 (40.0 to 95.1)	44.7	13.7
	Egypt	97.9 (97.5 to 98.2)	81.0 (77.9 to 83.7)	56.3 (47.7 to 64.5)	0.0	0.0
	Eswatini	87.9 (77.7 to 93.8)	96.2 (83.4 to 99.2)	97.9 (75.5 to 99.9)	96.2	88.6
	Ethiopia	28.7 (27.1 to 30.4)	63.4 (61.5 to 65.3)	78.3 (75.7 to 80.6)	7.5	0.0
	Gabon	54.9 (51.6 to 58.1)	83.8 (80.4 to 86.7)	91.4 (87.9 to 93.9)	100.0	80.4
	Gambia	95.2 (91.4 to 97.4)	90.0 (88.1 to 91.5)	85.8 (77.9 to 91.1)	93.2	7.0
	Ghana	81.9 (78.1 to 85.2)	92.9 (90.6 to 94.7)	95.7 (92.8 to 97.5)	100.0	99.9
	Guinea	60.4 (56.5 to 64.2)	43.4 (40.6 to 46.2)	35.2 (30.3 to 40.5)	0.0	0.0
	Kenya	69.8 (66.3 to 73.1)	91.8 (90.6 to 93.0)	96.1 (94.9 to 97.1)	100.0	100.0
	Lesotho	79.4 (74.6 to 83.5)	89.9 (86.2 to 92.7)	93.1 (87.8 to 96.2)	100.0	89.8
	Liberia	58.4 (53.2 to 63.5)	76.6 (74.1 to 78.9)	83.3 (79.1 to 86.9)	94.0	0.0
	Madagascar	52.8 (47.9 to 57.7)	86.9 (81.5 to 90.9)	94.2 (88.9 to 97.0)	100.0	94.8
	Malawi	81.0 (79.6 to 82.3)	94.3 (93.5 to 95.0)	97.0 (96.3 to 97.6)	100.0	100.0
	Mali	54.1 (52.3 to 56.0)	76.1 (74.3 to 77.8)	83.9 (81.6 to 86.0)	99.9	0.0
	Morocco	88.5 (83.7 to 91.9)	96.5 (79.2 to 99.5)	98.2 (69.5 to 99.9)	94.7	86.8
	Mozambique	74.7 (71.8 to 77.5)	85.9 (82.6 to 88.7)	89.7 (85.0 to 93.1)	100.0	44.9
	Namibia	79.7 (77.1 to 82.1)	92.4 (90.0 to 94.3)	95.5 (92.9 to 97.2)	100.0	100.0
	Niger	27.4 (23.5 to 31.7)	87.4 (84.4 to 89.8)	96.7 (95.0 to 97.9)	100.0	100.0
	Nigeria	31.0 (29.1 to 32.9)	54.2 (52.9 to 55.6)	65.8 (63.2 to 68.4)	0.0	0.0
	Rwanda	78.0 (76.1 to 79.8)	89.3 (87.5 to 90.9)	92.8 (90.5 to 94.6)	100.0	99.0
	Sao Tome and Principe	76.3 (56.6 to 88.7)	91.7 (74.5 to 97.7)	95.4 (65.1 to 99.6)	91.2	75.3
	Senegal	68.2 (65.3 to 71.0)	86.7 (85.6 to 87.9)	92.0 (90.3 to 93.3)	100.0	99.0
	Sierra Leone	58.4 (53.1 to 63.6)	78.1 (76.1 to 79.9)	85.0 (81.6 to 87.9)	99.7	0.0
	South Africa	74.7 (40.4 to 92.8)	87.3 (81.6 to 91.4)	91.2 (73.6 to 97.5)	92.4	58.7
	Tanzania	77.5 (74.5 to 80.3)	88.6 (86.7 to 90.3)	92.1 (89.4 to 94.2)	100.0	93.9
	Togo	60.0 (32.6 to 82.2)	80.4 (68.8 to 88.5)	87.2 (60.8 to 96.8)	76.2	35.4
	Uganda	56.7 (54.4 to 59.0)	84.5 (83.0 to 85.9)	91.7 (90.1 to 93.1)	100.0	98.2
	Zambia	82.6 (80.6 to 84.4)	88.9 (87.6 to 90.1)	91.3 (89.3 to 92.9)	100.0	90.6
	Zimbabwe	59.0 (54.0 to 63.9)	87.8 (85.2 to 90.0)	94.1 (91.4 to 96.1)	100.0	99.7
Polio3 immunization	Angola	51.0 (21.7 to 79.7)	50.2 (39.1 to 61.2)	49.7 (20.8 to 78.9)	2.0	0.1
	Benin	76.6 (74.7 to 78.5)	77.0 (75.0 to 78.8)	77.1 (73.5 to 80.3)	3.9	0.0
	Burkina Faso	46.4 (42.6 to 50.3)	99.7 (99.5 to 99.8)	100.0 (100.0 to 100.0)	100.0	100.0
	Burundi	92.3 (86.2 to 95.9)	97.7 (96.7 to 98.4)	98.8 (97.3 to 99.5)	100.0	100.0
	Cameroon	78.6 (76.1 to 81.0)	77.8 (75.7 to 79.8)	77.4 (73.2 to 81.1)	9.0	0.0

Indicators	Country	Estimates and Projections of Immunization Coverage (%)			Probability Achieving Targets (%)	
		Year 2000	Year 2020	Year 2030	UHC2030	IA2030
	Chad	35.8 (31.7 to 40.2)	65.2 (62.0 to 68.4)	77.5 (72.2 to 82.0)	14.7	0.0
	Comoros	65.4 (36.5 to 86.2)	84.5 (70.5 to 92.5)	90.3 (60.4 to 98.2)	82.3	51.3
	Congo Brazzaville	82.7 (78.0 to 86.5)	65.7 (57.6 to 72.9)	54.8 (38.9 to 69.7)	0.0	0.0
	Congo Democratic Republic	34.5 (29.6 to 39.8)	89.0 (86.9 to 90.8)	97.0 (95.5 to 97.9)	100.0	100.0
	Cote d'Ivoire	65.3 (39.4 to 84.6)	86.2 (71.9 to 93.8)	91.9 (64.0 to 98.6)	86.8	59.7
	Egypt	95.4 (94.6 to 96.0)	99.0 (98.5 to 99.3)	99.5 (99.1 to 99.7)	100.0	100.0
	Eswatini	90.7 (81.7 to 95.4)	98.2 (91.3 to 99.7)	99.2 (88.9 to 100.0)	99.2	97.0
	Ethiopia	37.0 (35.2 to 38.7)	66.3 (64.5 to 68.1)	78.3 (75.8 to 80.6)	8.0	0.0
	Gabon	36.5 (33.4 to 39.7)	50.1 (44.9 to 55.4)	57.1 (48.4 to 65.3)	0.0	0.0
	Gambia	95.1 (90.2 to 97.6)	94.8 (93.5 to 95.9)	94.7 (90.1 to 97.2)	100.0	97.7
	Ghana	82.1 (78.2 to 85.4)	94.5 (92.5 to 96.0)	97.1 (94.9 to 98.3)	100.0	100.0
	Guinea	64.1 (60.2 to 67.7)	57.3 (54.4 to 60.1)	53.8 (48.1 to 59.3)	0.0	0.0
	Kenya	71.8 (68.2 to 75.1)	95.8 (95.0 to 96.5)	98.6 (98.0 to 99.0)	100.0	100.0
	Lesotho	84.1 (79.9 to 87.5)	86.9 (82.4 to 90.4)	88.2 (79.9 to 93.4)	97.4	28.3
	Liberia	57.2 (51.8 to 62.4)	80.3 (78.0 to 82.5)	87.7 (84.2 to 90.5)	100.0	6.0
	Madagascar	60.2 (55.3 to 65.0)	87.3 (81.8 to 91.4)	93.6 (87.6 to 96.8)	100.0	90.6
	Malawi	83.5 (82.2 to 84.8)	94.8 (94.0 to 95.5)	97.2 (96.5 to 97.8)	100.0	100.0
	Mali	55.4 (53.6 to 57.2)	78.9 (77.2 to 80.5)	86.7 (84.6 to 88.5)	100.0	0.0
	Morocco	93.7 (90.5 to 95.9)	98.8 (91.5 to 99.9)	99.5 (88.6 to 100.0)	99.0	96.8
	Mozambique	71.9 (68.8 to 74.8)	89.4 (86.6 to 91.6)	93.8 (90.7 to 96.0)	100.0	99.0
	Namibia	85.4 (83.2 to 87.4)	87.8 (84.3 to 90.6)	88.9 (83.1 to 92.9)	99.7	31.7
	Niger	37.1 (32.1 to 42.2)	93.2 (91.3 to 94.8)	98.5 (97.6 to 99.1)	100.0	100.0
	Nigeria	36.3 (34.3 to 38.4)	69.9 (68.7 to 71.1)	82.4 (80.6 to 84.1)	99.5	0.0
	Rwanda	85.9 (84.2 to 87.4)	96.8 (95.9 to 97.5)	98.6 (97.8 to 99.1)	100.0	100.0
	Sao Tome and Principe	81.9 (64.3 to 91.9)	95.8 (85.2 to 98.9)	98.1 (81.4 to 99.8)	97.9	91.8
	Senegal	73.5 (70.4 to 76.4)	94.4 (93.7 to 95.0)	97.6 (97.0 to 98.2)	100.0	100.0
	Sierra Leone	53.8 (47.7 to 59.7)	90.0 (88.6 to 91.2)	96.1 (94.8 to 97.1)	100.0	100.0
	South Africa	63.7 (29.2 to 88.3)	77.0 (68.9 to 83.4)	82.2 (55.7 to 94.3)	58.8	15.2
	Tanzania	85.7 (83.2 to 88.0)	90.6 (88.7 to 92.1)	92.4 (89.3 to 94.6)	100.0	94.1
	Togo	72.5 (44.1 to 89.8)	92.4 (86.1 to 96.0)	96.3 (84.2 to 99.2)	98.9	90.6
	Uganda	57.0 (54.6 to 59.3)	89.4 (88.1 to 90.5)	95.5 (94.5 to 96.3)	100.0	100.0
	Zambia	79.5 (77.4 to 81.5)	90.6 (89.4 to 91.7)	93.8 (92.4 to 95.0)	100.0	100.0
	Zimbabwe	55.3 (50.3 to 60.3)	87.6 (85.0 to 89.8)	94.4 (91.8 to 96.2)	100.0	99.9
DPT3 immunization	Angola	38.2 (13.7 to 70.7)	40.4 (30.0 to 51.8)	41.6 (15.4 to 73.5)	0.7	0.0
	Benin	68.0 (65.8 to 70.0)	75.1 (73.1 to 76.9)	78.2 (74.9 to 81.1)	11.7	0.0
	Burkina Faso	38.4 (35.1 to 41.8)	99.1 (98.8 to 99.3)	99.9 (99.9 to 100.0)	100.0	100.0
	Burundi	90.4 (83.4 to 94.6)	97.5 (96.5 to 98.3)	98.8 (97.4 to 99.4)	100.0	100.0
	Cameroon	63.4 (60.3 to 66.3)	72.1 (69.9 to 74.2)	76.0 (72.1 to 79.5)	1.2	0.0
	Chad	16.3 (13.5 to 19.6)	42.6 (38.8 to 46.4)	59.1 (51.1 to 66.6)	0.0	0.0
	Comoros	54.9 (26.9 to 80.2)	82.0 (66.6 to 91.2)	89.8 (59.0 to 98.2)	80.7	49.0
	Congo Brazzaville	73.2 (67.8 to 78.0)	47.8 (40.4 to 55.4)	34.7 (23.1 to 48.4)	0.0	0.0
	Congo Democratic Republic	29.0 (24.7 to 33.8)	75.6 (72.1 to 78.9)	89.5 (85.5 to 92.6)	100.0	39.5
	Cote d'Ivoire	47.1 (23.5 to 72.2)	75.5 (56.0 to 88.2)	85.2 (47.3 to 97.3)	65.2	31.4
	Egypt	93.0 (92.2 to 93.8)	98.3 (97.7 to 98.7)	99.2 (98.7 to 99.5)	100.0	100.0
	Eswatini	90.9 (82.0 to 95.6)	98.7 (93.4 to 99.8)	99.5 (92.6 to 100.0)	99.7	98.5
	Ethiopia	22.6 (21.1 to 24.1)	66.3 (64.5 to 68.1)	83.6 (81.5 to 85.6)	100.0	0.0
	Gabon	41.4 (38.2 to 44.7)	91.2 (89.0 to 92.9)	97.5 (96.4 to 98.3)	100.0	100.0
	Gambia	71.5 (58.4 to 81.7)	93.3 (91.9 to 94.5)	97.0 (95.0 to 98.3)	100.0	100.0
	Ghana	77.6 (73.5 to 81.3)	92.5 (90.1 to 94.3)	95.9 (93.2 to 97.5)	100.0	100.0
	Guinea	56.6 (52.6 to 60.4)	40.3 (37.6 to 43.1)	32.7 (27.9 to 37.8)	0.0	0.0
	Kenya	67.5 (63.9 to 70.9)	94.6 (93.7 to 95.5)	98.1 (97.4 to 98.6)	100.0	100.0
	Lesotho	80.8 (76.2 to 84.6)	87.2 (82.9 to 90.5)	89.6 (82.4 to 94.1)	99.3	44.8
	Liberia	42.6 (37.7 to 47.7)	75.3 (72.7 to 77.7)	86.0 (82.4 to 89.0)	99.9	0.3
	Madagascar	54.2 (49.3 to 59.1)	90.0 (85.5 to 93.2)	96.1 (92.4 to 98.1)	100.0	99.7
	Malawi	82.3 (80.9 to 83.5)	95.2 (94.5 to 95.9)	97.6 (97.0 to 98.1)	100.0	100.0
	Mali	45.1 (43.3 to 46.9)	80.5 (78.9 to 82.0)	90.3 (88.7 to 91.6)	100.0	63.0
	Morocco	93.1 (89.6 to 95.4)	99.0 (92.7 to 99.9)	99.6 (91.4 to 100.0)	99.4	97.9
	Mozambique	69.7 (66.6 to 72.6)	80.9 (77.0 to 84.2)	85.1 (79.3 to 89.5)	96.0	1.4
	Namibia	79.9 (77.4 to 82.3)	86.3 (82.7 to 89.2)	88.7 (83.5 to 92.5)	99.9	27.8
	Niger	17.3 (14.4 to 20.5)	90.7 (88.4 to 92.6)	98.5 (97.7 to 99.1)	100.0	100.0

Indicators	Country	Estimates and Projections of Immunization Coverage (%)			Probability Achieving Targets (%)	
		Year 2000	Year 2020	Year 2030	UHC2030	IA2030
	Nigeria	21.8 (20.2 to 23.4)	52.9 (51.6 to 54.3)	69.4 (66.8 to 71.8)	0.0	0.0
	Rwanda	80.5 (78.6 to 82.3)	96.7 (95.8 to 97.3)	98.7 (98.1 to 99.1)	100.0	100.0
	Sao Tome and Principe	76.8 (56.9 to 89.1)	95.5 (84.6 to 98.8)	98.2 (82.7 to 99.8)	98.2	92.7
	Senegal	68.3 (65.1 to 71.3)	93.2 (92.4 to 93.9)	97.2 (96.5 to 97.8)	100.0	100.0
	Sierra Leone	36.5 (31.4 to 41.9)	89.0 (87.6 to 90.3)	96.8 (95.8 to 97.6)	100.0	100.0
	South Africa	50.9 (19.2 to 82.3)	67.0 (57.3 to 75.3)	74.0 (42.9 to 91.2)	30.2	4.0
	Tanzania	86.3 (83.8 to 88.5)	90.3 (88.3 to 91.9)	91.8 (88.6 to 94.2)	100.0	87.8
	Togo	61.1 (31.8 to 84.0)	89.8 (81.8 to 94.5)	95.4 (81.0 to 99.0)	97.9	85.2
	Uganda	46.6 (44.3 to 48.9)	85.0 (83.6 to 86.4)	93.6 (92.3 to 94.6)	100.0	100.0
	Zambia	76.4 (74.1 to 78.5)	90.9 (89.8 to 92.0)	94.7 (93.4 to 95.7)	100.0	100.0
	Zimbabwe	51.3 (46.1 to 56.4)	91.4 (89.4 to 93.1)	97.1 (95.7 to 98.1)	100.0	100.0

Notes: Countries are listed in alphabetical order. UHC = Probability of achieving the Universal Health Coverage (UHC) benchmark (⩾80% coverage) by 2030. IA = Probability of achieving the Immunization Agenda 2030 (IA2030) target (⩾90% coverage) by 2030.

Table G. Immunization coverage 2000–2030, and % reach global targets, by wealth quintile

Indicators	Country	Wealth Quintile	Estimates and Projections of Immunization Coverage (%)			Probability Achieving Targets (%)	
			Year 2000	Year 2020	Year 2030	UHC2030	IA2030
Full immunization	Angola	Poorest	16.6 (4.4 to 46.9)	11.0 (6.7 to 17.1)	8.8 (2.1 to 29.8)	0.0	0.0
		Poorer	26.7 (7.8 to 61.9)	20.5 (13.3 to 29.9)	17.8 (4.6 to 48.6)	0.0	0.0
		Middle	40.0 (13.4 to 74.9)	31.1 (21.3 to 42.6)	27.0 (7.6 to 61.7)	0.1	0.0
		Richer	58.1 (24.2 to 86.2)	43.4 (31.5 to 55.9)	36.3 (11.3 to 71.2)	0.5	0.0
		Richest	77.9 (44.5 to 94.1)	58.4 (45.4 to 70.1)	47.0 (16.5 to 79.4)	2.2	0.1
	Benin	Poorest	37.9 (35.3 to 40.7)	50.6 (47.7 to 53.5)	57.0 (52.2 to 61.7)	0.0	0.0
		Poorer	44.7 (42.1 to 47.3)	60.7 (58.2 to 63.2)	68.1 (63.9 to 72.1)	0.0	0.0
		Middle	51.5 (49.0 to 54.0)	66.0 (63.7 to 68.3)	72.4 (68.5 to 76.0)	0.0	0.0
		Richer	61.3 (58.7 to 63.9)	70.4 (68.0 to 72.6)	74.4 (70.5 to 77.9)	0.1	0.0
		Richest	74.4 (71.8 to 76.7)	75.8 (73.3 to 78.0)	76.4 (72.5 to 80.0)	2.5	0.0
	Burkina Faso	Poorest	20.7 (17.9 to 23.8)	97.9 (97.2 to 98.4)	99.8 (99.7 to 99.9)	100.0	100.0
		Poorer	23.6 (20.9 to 26.6)	98.4 (97.9 to 98.8)	99.9 (99.8 to 99.9)	100.0	100.0
		Middle	26.7 (24.0 to 29.7)	98.6 (98.1 to 98.9)	99.9 (99.8 to 99.9)	100.0	100.0
		Richer	32.8 (29.6 to 36.3)	98.7 (98.3 to 99.0)	99.9 (99.8 to 99.9)	100.0	100.0
		Richest	44.5 (40.2 to 48.8)	98.9 (98.5 to 99.2)	99.9 (99.8 to 99.9)	100.0	100.0
	Burundi	Poorest	88.6 (82.8 to 92.7)	86.3 (82.9 to 89.1)	85.0 (76.4 to 90.8)	88.9	5.1
		Poorer	89.5 (84.2 to 93.2)	88.7 (86.2 to 90.9)	88.3 (81.4 to 92.9)	98.9	26.7
		Middle	90.3 (85.3 to 93.7)	89.1 (86.7 to 91.1)	88.5 (81.7 to 93.0)	99.1	28.6
		Richer	92.0 (87.7 to 94.9)	89.2 (86.7 to 91.4)	87.6 (80.3 to 92.4)	97.8	19.2
		Richest	94.5 (91.4 to 96.6)	90.0 (87.3 to 92.2)	86.7 (78.7 to 92.0)	95.5	13.1
	Cameroon	Poorest	35.5 (32.1 to 39.1)	42.2 (39.0 to 45.5)	45.7 (40.2 to 51.3)	0.0	0.0
		Poorer	43.9 (40.6 to 47.3)	54.3 (51.5 to 57.1)	59.4 (54.2 to 64.4)	0.0	0.0
		Middle	52.6 (49.3 to 55.9)	61.6 (59.0 to 64.2)	65.9 (61.1 to 70.4)	0.0	0.0
		Richer	64.1 (60.7 to 67.4)	67.9 (65.2 to 70.5)	69.7 (64.9 to 74.2)	0.0	0.0
		Richest	77.8 (74.8 to 80.6)	75.0 (72.1 to 77.7)	73.5 (68.6 to 77.9)	0.1	0.0
	Chad	Poorest	6.8 (5.1 to 9.0)	29.6 (25.6 to 33.8)	50.1 (41.1 to 59.2)	0.0	0.0
		Poorer	8.0 (6.2 to 10.3)	36.4 (32.4 to 40.6)	59.4 (50.5 to 67.8)	0.0	0.0
		Middle	9.4 (7.4 to 11.8)	39.3 (35.3 to 43.5)	61.9 (53.0 to 70.0)	0.0	0.0
		Richer	12.2 (9.8 to 15.2)	41.7 (37.3 to 46.3)	61.9 (52.7 to 70.2)	0.0	0.0
		Richest	18.7 (15.1 to 22.9)	46.0 (40.8 to 51.2)	62.1 (52.5 to 70.8)	0.0	0.0
	Comoros	Poorest	35.5 (13.3 to 66.5)	61.0 (38.9 to 79.3)	72.5 (27.4 to 94.8)	33.3	10.3
		Poorer	43.0 (17.5 to 73.1)	71.0 (50.5 to 85.4)	81.5 (39.1 to 96.8)	54.0	22.9
		Middle	50.7 (22.3 to 78.8)	76.2 (57.2 to 88.4)	84.9 (45.1 to 97.4)	63.8	31.1
		Richer	61.5 (30.5 to 85.4)	80.2 (62.7 to 90.7)	86.6 (48.6 to 97.8)	69.1	36.4
		Richest	75.2 (44.7 to 92.0)	84.7 (69.2 to 93.2)	88.2 (52.1 to 98.1)	74.1	42.3
	Congo Brazzaville	Poorest	46.1 (38.9 to 53.4)	28.6 (22.8 to 35.1)	21.5 (13.4 to 32.6)	0.0	0.0
		Poorer	53.0 (46.3 to 59.6)	37.6 (30.7 to 45.1)	30.6 (19.8 to 44.1)	0.0	0.0
		Middle	59.7 (53.4 to 65.7)	43.1 (35.4 to 51.2)	35.1 (22.9 to 49.6)	0.0	0.0
		Richer	68.9 (63.1 to 74.2)	48.1 (39.4 to 56.9)	37.5 (24.4 to 52.7)	0.0	0.0
		Richest	80.2 (75.5 to 84.2)	54.9 (45.3 to 64.2)	40.1 (25.9 to 56.1)	0.0	0.0
	Congo Democratic Republic	Poorest	13.3 (10.6 to 16.5)	55.1 (50.1 to 60.0)	77.7 (70.1 to 83.8)	24.2	0.0
		Poorer	17.0 (13.8 to 20.7)	65.2 (60.7 to 69.4)	85.0 (79.3 to 89.4)	95.9	1.1
		Middle	21.3 (17.6 to 25.6)	70.4 (66.2 to 74.3)	87.6 (82.6 to 91.3)	99.7	11.2
		Richer	29.1 (24.4 to 34.3)	74.6 (70.6 to 78.3)	88.7 (84.0 to 92.2)	99.9	26.0
		Richest	43.1 (37.2 to 49.3)	79.7 (75.8 to 83.1)	89.9 (85.4 to 93.1)	100.0	47.9
	Cote d'Ivoire	Poorest	28.2 (11.4 to 54.8)	48.9 (27.1 to 70.9)	59.8 (17.4 to 91.2)	15.6	3.4
		Poorer	34.1 (14.5 to 61.4)	59.0 (36.2 to 78.4)	70.6 (25.5 to 94.3)	29.9	8.8
		Middle	40.5 (18.2 to 67.7)	64.3 (41.7 to 81.9)	74.6 (29.6 to 95.3)	37.5	12.5
		Richer	50.4 (24.8 to 75.9)	68.8 (46.6 to 84.7)	76.5 (31.8 to 95.7)	41.4	14.7
		Richest	64.9 (37.2 to 85.4)	74.3 (53.2 to 88.0)	78.4 (34.1 to 96.1)	45.9	17.4
	Egypt	Poorest	90.9 (89.8 to 91.9)	78.7 (75.5 to 81.6)	69.3 (62.7 to 75.1)	0.0	0.0
		Poorer	91.4 (90.4 to 92.3)	81.8 (79.2 to 84.1)	74.5 (68.8 to 79.4)	1.4	0.0
		Middle	91.8 (90.9 to 92.6)	81.9 (79.5 to 84.1)	74.2 (68.7 to 79.1)	0.9	0.0
		Richer	93.1 (92.2 to 93.9)	81.6 (79.1 to 83.9)	71.9 (66.0 to 77.1)	0.1	0.0
		Richest	95.2 (94.4 to 95.8)	82.4 (79.7 to 84.9)	69.6 (63.2 to 75.3)	0.0	0.0
	Eswatini	Poorest	79.5 (63.6 to 89.5)	94.6 (76.3 to 99.0)	97.4 (68.4 to 99.9)	93.9	83.9
		Poorer	80.9 (66.4 to 90.0)	95.6 (80.4 to 99.2)	98.0 (74.6 to 99.9)	96.0	88.4
		Middle	82.1 (68.1 to 90.6)	95.8 (81.1 to 99.2)	98.0 (75.0 to 99.9)	96.1	88.6
		Richer	84.9 (71.8 to 92.5)	95.8 (81.1 to 99.2)	97.9 (73.2 to 99.9)	95.6	87.3

Indicators	Country	Wealth Quintile	Estimates and Projections of Immunization Coverage (%)			Probability Achieving Targets (%)	
			Year 2000	Year 2020	Year 2030	UHC2030	IA2030
		Richest	89.5 (78.1 to 95.2)	96.1 (82.0 to 99.3)	97.7 (71.2 to 99.9)	94.9	85.9
	Ethiopia	Poorest	6.8 (6.0 to 7.8)	33.3 (30.9 to 35.8)	56.6 (52.1 to 60.9)	0.0	0.0
		Poorer	9.4 (8.4 to 10.4)	44.5 (42.0 to 47.0)	69.1 (65.2 to 72.8)	0.0	0.0
		Middle	12.6 (11.5 to 13.9)	51.8 (49.3 to 54.2)	74.5 (71.0 to 77.8)	0.0	0.0
		Richer	18.7 (17.1 to 20.4)	58.3 (55.7 to 60.9)	77.5 (74.1 to 80.6)	6.2	0.0
		Richest	30.9 (28.5 to 33.5)	66.2 (63.5 to 68.9)	80.4 (77.2 to 83.3)	60.5	0.0
	Gabon	Poorest	14.3 (11.8 to 17.2)	54.9 (49.0 to 60.7)	76.7 (68.7 to 83.1)	16.8	0.0
		Poorer	14.4 (12.2 to 16.9)	58.3 (52.5 to 63.8)	80.1 (72.8 to 85.8)	51.5	0.0
		Middle	14.4 (12.2 to 16.9)	57.2 (50.9 to 63.2)	79.0 (71.1 to 85.2)	39.0	0.0
		Richer	16.0 (13.3 to 19.1)	55.4 (48.2 to 62.4)	76.0 (66.8 to 83.3)	15.9	0.0
		Richest	21.0 (17.0 to 25.6)	55.5 (47.0 to 63.6)	73.0 (62.2 to 81.6)	5.9	0.0
	Gambia	Poorest	77.5 (67.1 to 85.3)	88.9 (86.7 to 90.7)	92.4 (88.3 to 95.1)	100.0	89.2
		Poorer	75.6 (65.1 to 83.8)	89.2 (87.3 to 90.8)	93.1 (89.3 to 95.5)	100.0	95.2
		Middle	73.6 (62.7 to 82.2)	87.6 (85.5 to 89.4)	91.8 (87.5 to 94.7)	100.0	82.2
		Richer	74.0 (63.1 to 82.6)	85.5 (82.8 to 87.8)	89.5 (84.0 to 93.2)	99.9	40.8
		Richest	78.0 (67.7 to 85.8)	84.1 (80.6 to 87.2)	86.6 (79.6 to 91.5)	96.9	10.2
	Ghana	Poorest	61.7 (56.3 to 66.7)	82.5 (78.4 to 86.0)	89.0 (83.8 to 92.7)	99.9	32.4
		Poorer	65.1 (60.4 to 69.6)	86.3 (83.0 to 89.0)	92.0 (88.1 to 94.7)	100.0	86.3
		Middle	68.3 (63.7 to 72.6)	87.4 (84.3 to 89.9)	92.5 (88.8 to 95.1)	100.0	92.2
		Richer	73.9 (69.2 to 78.1)	88.2 (85.0 to 90.8)	92.4 (88.4 to 95.0)	100.0	89.6
		Richest	82.0 (77.6 to 85.7)	89.6 (86.3 to 92.2)	92.2 (88.0 to 95.1)	100.0	86.8
	Guinea	Poorest	36.7 (32.4 to 41.3)	18.6 (16.3 to 21.3)	12.6 (9.9 to 15.9)	0.0	0.0
		Poorer	42.6 (38.4 to 47.0)	25.1 (22.6 to 27.8)	18.4 (14.8 to 22.6)	0.0	0.0
		Middle	48.6 (44.3 to 52.9)	29.0 (26.3 to 31.7)	21.1 (17.2 to 25.6)	0.0	0.0
		Richer	57.8 (53.2 to 62.2)	32.6 (29.6 to 35.7)	22.3 (18.1 to 27.1)	0.0	0.0
		Richest	70.8 (66.3 to 74.9)	38.1 (34.3 to 42.0)	23.7 (19.1 to 29.0)	0.0	0.0
	Kenya	Poorest	39.8 (35.8 to 43.9)	80.5 (78.1 to 82.6)	91.1 (88.8 to 93.0)	100.0	84.0
		Poorer	46.4 (42.5 to 50.3)	86.0 (84.2 to 87.6)	94.3 (92.6 to 95.5)	100.0	100.0
		Middle	52.9 (49.1 to 56.7)	88.4 (86.8 to 89.9)	95.2 (93.8 to 96.3)	100.0	100.0
		Richer	62.4 (58.6 to 66.1)	90.2 (88.7 to 91.6)	95.6 (94.2 to 96.7)	100.0	100.0
		Richest	75.0 (71.5 to 78.2)	92.3 (90.8 to 93.6)	96.0 (94.6 to 97.1)	100.0	100.0
	Lesotho	Poorest	60.8 (54.6 to 66.6)	69.7 (63.1 to 75.6)	73.7 (62.7 to 82.4)	8.7	0.0
		Poorer	63.2 (57.7 to 68.4)	74.5 (68.8 to 79.4)	79.2 (69.6 to 86.3)	42.0	0.0
		Middle	65.5 (60.1 to 70.5)	75.4 (69.9 to 80.3)	79.6 (70.2 to 86.6)	46.5	0.1
		Richer	70.4 (65.0 to 75.3)	75.9 (70.2 to 80.9)	78.4 (68.5 to 85.9)	35.8	0.0
		Richest	78.5 (73.4 to 82.8)	77.7 (71.6 to 82.8)	77.3 (66.6 to 85.3)	27.8	0.0
	Liberia	Poorest	28.4 (24.0 to 33.4)	52.7 (49.3 to 56.1)	65.1 (58.9 to 70.9)	0.0	0.0
		Poorer	33.3 (28.8 to 38.2)	61.5 (58.5 to 64.5)	74.1 (68.7 to 78.9)	0.7	0.0
		Middle	38.5 (33.7 to 43.5)	65.7 (62.5 to 68.7)	77.0 (71.7 to 81.5)	10.3	0.0
		Richer	47.0 (41.7 to 52.5)	69.0 (65.3 to 72.4)	77.9 (72.3 to 82.6)	20.2	0.0
		Richest	60.7 (54.9 to 66.2)	73.6 (69.4 to 77.4)	78.9 (72.9 to 83.9)	34.6	0.0
	Madagascar	Poorest	27.5 (22.8 to 32.7)	67.9 (57.8 to 76.5)	83.3 (70.3 to 91.3)	71.9	6.0
		Poorer	38.0 (32.8 to 43.5)	79.6 (71.6 to 85.8)	90.8 (82.3 to 95.4)	99.1	59.1
		Middle	49.6 (44.1 to 55.0)	85.7 (79.3 to 90.3)	93.7 (87.3 to 96.9)	100.0	89.8
		Richer	64.2 (59.0 to 69.1)	89.9 (84.9 to 93.4)	95.2 (90.2 to 97.7)	100.0	97.8
		Richest	80.0 (76.0 to 83.6)	93.5 (89.8 to 95.9)	96.5 (92.5 to 98.4)	100.0	99.7
	Malawi	Poorest	62.9 (60.5 to 65.3)	86.2 (84.6 to 87.6)	92.3 (90.7 to 93.6)	100.0	99.6
		Poorer	66.5 (64.4 to 68.4)	89.3 (88.1 to 90.4)	94.5 (93.3 to 95.4)	100.0	100.0
		Middle	69.7 (67.8 to 71.5)	90.2 (89.1 to 91.2)	94.9 (93.8 to 95.8)	100.0	100.0
		Richer	75.3 (73.4 to 77.1)	90.9 (89.7 to 92.0)	94.8 (93.6 to 95.7)	100.0	100.0
		Richest	83.1 (81.3 to 84.7)	92.1 (90.9 to 93.1)	94.7 (93.4 to 95.8)	100.0	100.0
	Mali	Poorest	25.4 (23.4 to 27.6)	56.7 (53.8 to 59.6)	72.0 (68.1 to 75.6)	0.0	0.0
		Poorer	29.4 (27.5 to 31.4)	64.7 (62.2 to 67.0)	79.3 (76.2 to 82.1)	31.6	0.0
		Middle	33.6 (31.7 to 35.6)	68.0 (65.8 to 70.1)	81.3 (78.5 to 83.8)	82.5	0.0
		Richer	41.2 (38.9 to 43.4)	70.6 (68.3 to 72.8)	81.7 (78.8 to 84.2)	87.5	0.0
		Richest	54.2 (51.5 to 56.8)	74.5 (72.1 to 76.8)	82.1 (79.0 to 84.8)	91.5	0.0
	Morocco	Poorest	76.7 (68.0 to 83.5)	92.8 (62.4 to 99.1)	96.3 (50.0 to 99.9)	87.3	74.0
		Poorer	83.3 (76.8 to 88.2)	95.7 (74.4 to 99.4)	97.9 (65.1 to 99.9)	93.5	84.7
		Middle	88.3 (83.0 to 92.0)	97.0 (80.7 to 99.6)	98.5 (72.5 to 99.9)	95.7	89.1
		Richer	92.8 (88.7 to 95.5)	97.9 (85.3 to 99.7)	98.8 (76.9 to 100.0)	96.8	91.5
		Richest	96.5 (93.8 to 98.0)	98.6 (89.6 to 99.8)	99.1 (80.9 to 100.0)	97.6	93.5

Indicators	Country	Wealth Quintile	Estimates and Projections of Immunization Coverage (%)			Probability Achieving Targets (%)	
			Year 2000	Year 2020	Year 2030	UHC2030	IA2030
	Mozambique	Poorest	45.4 (41.3 to 49.6)	57.3 (51.1 to 63.4)	63.1 (52.9 to 72.1)	0.0	0.0
		Poorer	55.2 (51.5 to 58.9)	69.5 (64.2 to 74.3)	75.5 (67.3 to 82.3)	10.6	0.0
		Middle	64.5 (61.0 to 67.9)	76.2 (71.7 to 80.2)	81.0 (74.0 to 86.4)	61.7	0.0
		Richer	75.3 (72.1 to 78.2)	81.5 (77.6 to 84.8)	84.1 (77.8 to 88.8)	90.9	0.5
		Richest	86.2 (83.7 to 88.3)	86.7 (83.4 to 89.4)	86.9 (81.3 to 91.0)	99.0	7.6
	Namibia	Poorest	64.4 (60.3 to 68.4)	78.6 (73.9 to 82.7)	83.9 (77.6 to 88.7)	89.9	0.5
		Poorer	65.6 (62.2 to 68.9)	81.5 (77.6 to 84.9)	87.0 (81.9 to 90.9)	99.5	7.5
		Middle	66.6 (63.5 to 69.6)	81.5 (77.7 to 84.9)	86.8 (81.5 to 90.7)	99.3	5.9
		Richer	70.4 (67.1 to 73.5)	81.2 (77.0 to 84.7)	85.3 (79.4 to 89.7)	96.3	1.7
		Richest	77.6 (74.0 to 80.8)	81.9 (77.3 to 85.7)	83.8 (77.0 to 88.8)	87.7	0.5
	Niger	Poorest	8.2 (6.4 to 10.4)	69.7 (63.9 to 74.9)	92.1 (87.9 to 94.9)	100.0	85.8
		Poorer	10.5 (8.4 to 13.0)	77.5 (72.8 to 81.5)	94.9 (92.1 to 96.7)	100.0	99.9
		Middle	13.3 (10.8 to 16.1)	81.1 (77.0 to 84.6)	95.8 (93.4 to 97.3)	100.0	100.0
		Richer	18.5 (15.3 to 22.1)	83.9 (80.2 to 87.0)	96.1 (93.9 to 97.6)	100.0	100.0
		Richest	29.1 (24.6 to 34.1)	87.1 (83.9 to 89.8)	96.5 (94.5 to 97.8)	100.0	100.0
	Nigeria	Poorest	3.1 (2.7 to 3.6)	14.8 (13.6 to 16.0)	28.8 (25.5 to 32.4)	0.0	0.0
		Poorer	6.3 (5.6 to 7.1)	29.5 (27.9 to 31.2)	51.0 (47.1 to 55.0)	0.0	0.0
		Middle	12.4 (11.1 to 13.8)	45.6 (43.7 to 47.5)	67.1 (63.6 to 70.5)	0.0	0.0
		Richer	25.2 (23.0 to 27.5)	62.1 (60.1 to 64.0)	78.3 (75.4 to 80.9)	10.7	0.0
		Richest	49.6 (46.4 to 52.8)	77.5 (75.7 to 79.1)	86.5 (84.3 to 88.5)	100.0	0.0
	Rwanda	Poorest	65.9 (63.0 to 68.7)	91.1 (89.5 to 92.6)	96.0 (94.6 to 97.0)	100.0	100.0
		Poorer	65.5 (62.9 to 68.1)	92.0 (90.6 to 93.3)	96.6 (95.5 to 97.5)	100.0	100.0
		Middle	65.0 (62.5 to 67.5)	91.5 (90.0 to 92.8)	96.3 (95.1 to 97.2)	100.0	100.0
		Richer	67.4 (64.7 to 69.9)	90.8 (89.1 to 92.2)	95.6 (94.0 to 96.7)	100.0	100.0
		Richest	73.8 (71.1 to 76.3)	90.6 (88.8 to 92.2)	94.7 (92.9 to 96.1)	100.0	100.0
	Sao Tome and Principe	Poorest	58.3 (34.9 to 78.3)	85.5 (58.8 to 96.1)	92.3 (49.1 to 99.4)	80.7	59.0
		Poorer	62.2 (39.1 to 80.7)	88.8 (66.4 to 97.0)	94.5 (58.6 to 99.6)	87.7	69.8
		Middle	65.9 (42.7 to 83.1)	89.9 (69.0 to 97.3)	95.0 (60.9 to 99.6)	89.1	72.3
		Richer	72.0 (48.9 to 87.2)	90.6 (70.6 to 97.6)	94.9 (60.5 to 99.6)	88.9	71.9
		Richest	80.7 (59.5 to 92.2)	91.9 (73.2 to 98.0)	94.9 (60.1 to 99.6)	88.7	71.7
	Senegal	Poorest	44.3 (40.9 to 47.8)	78.6 (76.9 to 80.3)	88.8 (86.7 to 90.6)	100.0	10.0
		Poorer	49.1 (45.8 to 52.4)	83.6 (82.2 to 84.9)	92.1 (90.6 to 93.5)	100.0	99.6
		Middle	53.8 (50.5 to 57.0)	85.4 (84.1 to 86.7)	92.9 (91.5 to 94.2)	100.0	100.0
		Richer	61.4 (58.1 to 64.7)	86.8 (85.4 to 88.1)	93.0 (91.5 to 94.3)	100.0	100.0
		Richest	72.7 (69.5 to 75.7)	88.8 (87.3 to 90.2)	93.2 (91.5 to 94.6)	100.0	100.0
	Sierra Leone	Poorest	43.3 (37.9 to 48.9)	70.6 (67.9 to 73.2)	81.0 (76.8 to 84.5)	68.7	0.0
		Poorer	42.7 (37.6 to 47.9)	72.8 (70.5 to 75.0)	83.5 (79.9 to 86.6)	97.2	0.0
		Middle	42.0 (37.0 to 47.1)	71.3 (69.0 to 73.5)	82.1 (78.3 to 85.5)	86.9	0.0
		Richer	44.4 (39.2 to 49.7)	69.2 (66.5 to 71.8)	79.0 (74.5 to 82.9)	31.8	0.0
		Richest	51.9 (46.2 to 57.5)	68.6 (65.3 to 71.7)	75.6 (70.3 to 80.3)	3.3	0.0
	South Africa	Poorest	39.2 (11.7 to 76.2)	62.7 (51.1 to 72.9)	73.1 (39.2 to 91.8)	29.3	4.8
		Poorer	38.8 (11.5 to 75.9)	65.4 (54.8 to 74.5)	76.5 (44.0 to 93.0)	38.5	7.6
		Middle	38.3 (11.2 to 75.6)	63.8 (53.3 to 73.1)	74.8 (42.0 to 92.3)	33.6	5.9
		Richer	40.8 (12.2 to 77.7)	61.6 (50.4 to 71.6)	71.0 (37.3 to 90.9)	24.3	3.4
		Richest	48.4 (15.6 to 82.9)	61.1 (48.8 to 72.1)	67.1 (32.8 to 89.3)	17.1	1.9
	Tanzania	Poorest	61.0 (56.8 to 65.1)	73.0 (69.3 to 76.4)	78.0 (72.2 to 82.9)	22.4	0.0
		Poorer	67.3 (63.6 to 70.8)	80.2 (77.5 to 82.7)	85.1 (80.8 to 88.5)	98.9	0.2
		Middle	72.9 (69.6 to 76.0)	83.5 (81.1 to 85.7)	87.4 (83.7 to 90.4)	100.0	4.8
		Richer	80.0 (77.0 to 82.7)	86.1 (83.8 to 88.1)	88.5 (84.9 to 91.3)	100.0	15.5
		Richest	87.9 (85.5 to 89.9)	89.0 (86.8 to 90.8)	89.5 (85.9 to 92.2)	100.0	37.0
	Togo	Poorest	40.6 (15.8 to 71.1)	75.6 (60.2 to 86.5)	86.9 (54.9 to 97.3)	72.1	35.8
		Poorer	42.8 (17.0 to 72.9)	79.5 (65.7 to 88.8)	89.8 (62.1 to 98.0)	82.4	49.0
		Middle	44.9 (18.2 to 74.6)	80.2 (66.7 to 89.1)	90.0 (62.6 to 98.0)	83.0	49.8
		Richer	50.3 (21.5 to 78.6)	80.4 (66.9 to 89.3)	89.2 (60.6 to 97.8)	80.3	45.8
		Richest	60.5 (29.0 to 84.9)	81.7 (68.6 to 90.2)	88.4 (58.7 to 97.7)	77.6	42.2
	Uganda	Poorest	35.5 (32.7 to 38.3)	69.9 (67.4 to 72.3)	82.7 (79.7 to 85.3)	96.2	0.0
		Poorer	36.2 (33.7 to 38.8)	73.2 (71.1 to 75.3)	85.7 (83.3 to 87.9)	100.0	0.0
		Middle	36.8 (34.4 to 39.3)	72.9 (70.7 to 74.9)	85.2 (82.6 to 87.5)	100.0	0.0
		Richer	40.5 (37.9 to 43.2)	72.0 (69.6 to 74.3)	83.3 (80.3 to 85.9)	98.5	0.0
		Richest	49.3 (46.2 to 52.3)	72.5 (69.8 to 75.1)	81.3 (77.8 to 84.4)	77.9	0.0
	Zambia	Poorest	57.8 (54.5 to 60.9)	74.9 (72.5 to 77.1)	81.5 (78.2 to 84.3)	81.8	0.0

Indicators	Country	Wealth Quintile	Estimates and Projections of Immunization Coverage (%)			Probability Achieving Targets (%)	
			Year 2000	Year 2020	Year 2030	UHC2030	IA2030
		Poorer	62.7 (59.9 to 65.5)	80.7 (78.9 to 82.4)	86.8 (84.4 to 88.9)	100.0	0.1
		Middle	67.3 (64.7 to 69.9)	83.0 (81.4 to 84.6)	88.3 (86.1 to 90.2)	100.0	4.4
		Richer	74.2 (71.5 to 76.6)	84.8 (83.0 to 86.4)	88.6 (86.2 to 90.6)	100.0	9.7
		Richest	83.0 (80.6 to 85.2)	87.3 (85.4 to 88.9)	89.0 (86.4 to 91.2)	100.0	19.8
	Zimbabwe	Poorest	34.0 (29.3 to 39.0)	79.9 (76.0 to 83.4)	91.7 (88.1 to 94.3)	100.0	84.3
		Poorer	37.2 (32.6 to 42.1)	84.0 (80.9 to 86.6)	94.0 (91.3 to 95.8)	100.0	99.7
		Middle	40.4 (35.7 to 45.3)	85.1 (82.3 to 87.6)	94.3 (91.8 to 96.1)	100.0	99.9
		Richer	46.9 (41.7 to 52.2)	85.9 (83.1 to 88.4)	94.1 (91.5 to 96.0)	100.0	99.8
		Richest	58.4 (52.6 to 64.0)	87.5 (84.6 to 89.9)	94.0 (91.2 to 95.9)	100.0	99.6
BCG immunization	Angola	Poorest	49.7 (17.1 to 83.0)	42.0 (29.4 to 55.5)	38.3 (11.0 to 75.0)	1.0	0.0
		Poorer	66.7 (29.5 to 90.8)	63.6 (50.3 to 74.8)	62.0 (24.7 to 88.7)	13.1	1.6
		Middle	78.4 (43.0 to 94.8)	79.1 (68.7 to 86.6)	79.5 (43.7 to 94.9)	48.3	14.5
		Richer	90.3 (65.7 to 97.9)	88.9 (82.0 to 93.3)	88.1 (59.7 to 97.3)	77.7	40.4
		Richest	96.9 (86.4 to 99.4)	96.0 (92.9 to 97.7)	95.4 (80.4 to 99.0)	97.7	84.7
	Benin	Poorest	78.2 (75.4 to 80.8)	72.8 (69.6 to 75.8)	69.8 (63.7 to 75.3)	0.0	0.0
		Poorer	85.6 (83.6 to 87.3)	84.1 (82.0 to 86.0)	83.3 (79.1 to 86.8)	94.1	0.0
		Middle	89.8 (88.2 to 91.2)	90.3 (88.8 to 91.6)	90.6 (87.9 to 92.8)	100.0	67.7
		Richer	94.9 (93.8 to 95.7)	94.2 (93.0 to 95.1)	93.8 (91.7 to 95.4)	100.0	99.9
		Richest	98.1 (97.6 to 98.5)	97.5 (96.9 to 98.0)	97.2 (96.0 to 98.0)	100.0	100.0
	Burkina Faso	Poorest	51.9 (45.9 to 57.8)	99.4 (99.0 to 99.7)	100.0 (99.9 to 100.0)	100.0	100.0
		Poorer	59.9 (54.9 to 64.7)	99.7 (99.4 to 99.8)	100.0 (99.9 to 100.0)	100.0	100.0
		Middle	64.9 (60.2 to 69.4)	99.8 (99.6 to 99.9)	100.0 (100.0 to 100.0)	100.0	100.0
		Richer	76.5 (72.1 to 80.5)	99.8 (99.7 to 99.9)	100.0 (100.0 to 100.0)	100.0	100.0
		Richest	88.3 (84.9 to 91.0)	99.9 (99.9 to 100.0)	100.0 (100.0 to 100.0)	100.0	100.0
	Burundi	Poorest	98.0 (95.0 to 99.3)	95.3 (92.4 to 97.1)	92.9 (80.8 to 97.5)	97.9	74.1
		Poorer	98.6 (96.4 to 99.5)	97.1 (95.5 to 98.2)	96.0 (88.7 to 98.6)	99.9	95.7
		Middle	98.9 (97.1 to 99.6)	98.1 (96.9 to 98.8)	97.5 (92.7 to 99.1)	100.0	99.5
		Richer	99.4 (98.3 to 99.8)	98.7 (97.7 to 99.2)	98.1 (94.3 to 99.4)	100.0	99.9
		Richest	99.7 (99.2 to 99.9)	99.4 (98.8 to 99.7)	99.0 (96.8 to 99.7)	100.0	100.0
	Cameroon	Poorest	74.2 (70.1 to 77.8)	73.1 (69.5 to 76.5)	72.6 (66.0 to 78.3)	0.4	0.0
		Poorer	82.4 (79.5 to 85.0)	84.1 (81.8 to 86.1)	84.9 (80.6 to 88.4)	98.7	0.1
		Middle	87.2 (84.8 to 89.3)	90.2 (88.6 to 91.6)	91.5 (88.7 to 93.6)	100.0	86.6
		Richer	93.4 (91.8 to 94.7)	94.0 (92.7 to 95.1)	94.3 (92.2 to 95.9)	100.0	100.0
		Richest	97.5 (96.6 to 98.1)	97.4 (96.6 to 98.0)	97.4 (96.2 to 98.2)	100.0	100.0
	Chad	Poorest	24.2 (20.1 to 29.0)	57.5 (53.3 to 61.7)	73.6 (67.1 to 79.3)	1.2	0.0
		Poorer	27.5 (23.3 to 32.1)	65.7 (62.1 to 69.2)	81.1 (75.9 to 85.5)	67.5	0.0
		Middle	28.7 (24.7 to 33.1)	70.8 (67.4 to 74.0)	85.6 (81.2 to 89.1)	99.3	0.6
		Richer	37.7 (33.1 to 42.6)	75.0 (71.6 to 78.2)	87.0 (82.7 to 90.3)	99.9	4.1
		Richest	54.4 (49.2 to 59.6)	84.0 (80.9 to 86.6)	91.7 (88.4 to 94.1)	100.0	85.5
	Comoros	Poorest	64.1 (30.9 to 87.9)	84.9 (67.6 to 93.8)	90.9 (54.5 to 98.8)	80.6	54.0
		Poorer	69.0 (36.0 to 90.0)	89.4 (76.2 to 95.6)	94.2 (66.4 to 99.2)	90.7	71.3
		Middle	71.3 (38.3 to 91.1)	91.8 (81.1 to 96.7)	96.0 (74.4 to 99.5)	95.3	82.2
		Richer	79.8 (48.8 to 94.3)	93.6 (84.5 to 97.5)	96.6 (77.4 to 99.6)	96.6	85.9
		Richest	89.1 (65.3 to 97.3)	96.4 (90.5 to 98.7)	98.0 (85.3 to 99.7)	98.9	94.1
	Congo Brazzaville	Poorest	69.9 (59.4 to 78.7)	93.3 (89.4 to 95.9)	97.2 (93.0 to 98.9)	100.0	99.7
		Poorer	82.2 (75.1 to 87.6)	97.1 (95.1 to 98.3)	98.9 (97.1 to 99.6)	100.0	100.0
		Middle	89.1 (84.1 to 92.7)	98.6 (97.5 to 99.2)	99.5 (98.6 to 99.8)	100.0	100.0
		Richer	95.4 (92.6 to 97.1)	99.3 (98.6 to 99.7)	99.7 (99.2 to 99.9)	100.0	100.0
		Richest	98.6 (97.4 to 99.2)	99.8 (99.5 to 99.9)	99.9 (99.7 to 100.0)	100.0	100.0
	Congo Democratic Republic	Poorest	40.6 (34.1 to 47.5)	84.4 (81.0 to 87.3)	93.9 (90.5 to 96.1)	100.0	98.7
		Poorer	47.9 (41.2 to 54.5)	89.7 (87.3 to 91.6)	96.4 (94.3 to 97.7)	100.0	100.0
		Middle	52.4 (45.7 to 59.0)	92.5 (90.7 to 94.1)	97.7 (96.3 to 98.5)	100.0	100.0
		Richer	65.2 (58.7 to 71.2)	94.6 (93.0 to 95.8)	98.1 (97.0 to 98.9)	100.0	100.0
		Richest	80.7 (75.5 to 85.0)	97.2 (96.2 to 97.9)	99.0 (98.3 to 99.4)	100.0	100.0
	Cote d'Ivoire	Poorest	57.8 (29.1 to 82.2)	79.3 (57.7 to 91.3)	86.5 (43.3 to 98.1)	67.0	37.1
		Poorer	66.8 (37.6 to 87.2)	87.0 (70.8 to 94.8)	92.4 (59.6 to 99.0)	85.3	61.3
		Middle	72.5 (43.9 to 90.0)	91.3 (79.3 to 96.6)	95.4 (71.8 to 99.4)	94.0	78.8
		Richer	83.1 (58.9 to 94.5)	94.1 (85.3 to 97.8)	96.7 (77.9 to 99.6)	96.8	86.5

Indicators	Country	Wealth Quintile	Estimates and Projections of Immunization Coverage (%)			Probability Achieving Targets (%)	
			Year 2000	Year 2020	Year 2030	UHC2030	IA2030
		Richest	92.3 (77.2 to 97.7)	97.2 (92.4 to 99.0)	98.3 (87.6 to 99.8)	99.3	95.9
	Egypt	Poorest	98.1 (97.4 to 98.6)	97.6 (96.2 to 98.5)	97.4 (94.5 to 98.8)	100.0	100.0
		Poorer	98.5 (98.0 to 98.8)	98.4 (97.6 to 99.0)	98.4 (96.7 to 99.3)	100.0	100.0
		Middle	98.7 (98.2 to 99.0)	98.8 (98.2 to 99.3)	98.9 (97.7 to 99.5)	100.0	100.0
		Richer	99.2 (98.8 to 99.4)	99.1 (98.6 to 99.5)	99.1 (98.1 to 99.6)	100.0	100.0
		Richest	99.6 (99.4 to 99.7)	99.5 (99.2 to 99.7)	99.5 (98.8 to 99.8)	100.0	100.0
	Eswatini	Poorest	94.3 (86.0 to 97.8)	98.9 (93.0 to 99.9)	99.5 (90.3 to 100.0)	99.3	97.6
		Poorer	94.4 (87.2 to 97.7)	99.1 (94.6 to 99.9)	99.7 (92.9 to 100.0)	99.6	98.5
		Middle	94.0 (86.3 to 97.5)	99.2 (95.2 to 99.9)	99.7 (94.2 to 100.0)	99.7	98.9
		Richer	95.4 (88.3 to 98.2)	99.3 (95.4 to 99.9)	99.7 (94.0 to 100.0)	99.7	98.8
		Richest	97.3 (91.8 to 99.1)	99.5 (96.7 to 99.9)	99.8 (95.6 to 100.0)	99.8	99.3
	Ethiopia	Poorest	39.5 (36.9 to 42.3)	64.6 (62.1 to 67.1)	75.4 (71.8 to 78.6)	0.2	0.0
		Poorer	43.4 (40.9 to 45.9)	71.9 (69.6 to 74.0)	82.3 (79.4 to 84.9)	94.5	0.0
		Middle	44.5 (42.1 to 46.9)	76.2 (74.1 to 78.2)	86.5 (84.0 to 88.6)	100.0	0.0
		Richer	54.4 (51.7 to 57.1)	79.6 (77.4 to 81.7)	87.6 (85.0 to 89.8)	100.0	1.5
		Richest	70.0 (67.2 to 72.5)	87.1 (85.2 to 88.7)	92.0 (89.9 to 93.6)	100.0	97.2
	Gabon	Poorest	81.2 (77.4 to 84.5)	90.1 (86.5 to 92.8)	92.9 (88.2 to 95.8)	100.0	90.4
		Poorer	85.7 (83.0 to 88.0)	93.7 (91.2 to 95.5)	95.9 (92.9 to 97.7)	100.0	99.9
		Middle	88.1 (85.3 to 90.4)	95.6 (93.6 to 97.1)	97.4 (95.3 to 98.6)	100.0	100.0
		Richer	92.8 (90.3 to 94.7)	96.9 (95.1 to 98.1)	98.0 (96.1 to 99.0)	100.0	100.0
		Richest	96.7 (95.0 to 97.9)	98.5 (97.3 to 99.1)	99.0 (97.7 to 99.5)	100.0	100.0
	Gambia	Poorest	97.2 (90.5 to 99.2)	98.9 (98.1 to 99.4)	99.4 (97.8 to 99.8)	100.0	100.0
		Poorer	97.2 (90.8 to 99.2)	99.1 (98.6 to 99.5)	99.5 (98.4 to 99.9)	100.0	100.0
		Middle	96.9 (89.8 to 99.1)	99.2 (98.6 to 99.5)	99.6 (98.6 to 99.9)	100.0	100.0
		Richer	97.5 (91.5 to 99.3)	99.2 (98.5 to 99.6)	99.6 (98.4 to 99.9)	100.0	100.0
		Richest	98.5 (94.3 to 99.6)	99.5 (98.8 to 99.8)	99.7 (98.8 to 99.9)	100.0	100.0
	Ghana	Poorest	83.7 (78.3 to 87.9)	97.0 (95.2 to 98.2)	98.8 (97.3 to 99.5)	100.0	100.0
		Poorer	86.8 (82.7 to 90.1)	98.0 (96.8 to 98.8)	99.3 (98.4 to 99.7)	100.0	100.0
		Middle	88.3 (84.3 to 91.5)	98.6 (97.6 to 99.1)	99.5 (98.9 to 99.8)	100.0	100.0
		Richer	92.5 (89.1 to 95.0)	98.9 (98.1 to 99.4)	99.6 (99.0 to 99.8)	100.0	100.0
		Richest	96.4 (94.0 to 97.8)	99.4 (98.9 to 99.7)	99.8 (99.4 to 99.9)	100.0	100.0
	Guinea	Poorest	75.0 (70.6 to 79.0)	58.6 (54.5 to 62.6)	49.2 (41.8 to 56.7)	0.0	0.0
		Poorer	80.3 (76.7 to 83.4)	69.5 (66.3 to 72.6)	63.1 (56.1 to 69.6)	0.0	0.0
		Middle	83.2 (79.9 to 86.0)	76.8 (73.9 to 79.4)	73.0 (66.8 to 78.5)	0.5	0.0
		Richer	89.5 (87.0 to 91.6)	82.4 (79.6 to 84.9)	77.7 (71.7 to 82.7)	19.3	0.0
		Richest	95.0 (93.5 to 96.2)	90.3 (88.1 to 92.2)	86.7 (81.9 to 90.4)	99.6	4.2
	Kenya	Poorest	72.9 (67.5 to 77.7)	96.9 (96.0 to 97.6)	99.1 (98.5 to 99.4)	100.0	100.0
		Poorer	80.2 (76.2 to 83.7)	98.2 (97.7 to 98.7)	99.5 (99.2 to 99.7)	100.0	100.0
		Middle	84.5 (81.0 to 87.5)	98.9 (98.5 to 99.2)	99.7 (99.5 to 99.8)	100.0	100.0
		Richer	91.3 (88.6 to 93.4)	99.3 (99.0 to 99.5)	99.8 (99.7 to 99.9)	100.0	100.0
		Richest	96.3 (94.8 to 97.4)	99.7 (99.5 to 99.8)	99.9 (99.8 to 100.0)	100.0	100.0
	Lesotho	Poorest	91.2 (86.0 to 94.5)	96.8 (93.8 to 98.3)	98.1 (94.0 to 99.4)	100.0	99.8
		Poorer	92.9 (89.0 to 95.5)	97.9 (95.9 to 98.9)	98.8 (96.4 to 99.6)	100.0	100.0
		Middle	93.8 (90.1 to 96.1)	98.4 (96.9 to 99.2)	99.2 (97.5 to 99.8)	100.0	100.0
		Richer	96.1 (93.2 to 97.7)	98.8 (97.5 to 99.4)	99.4 (97.8 to 99.8)	100.0	100.0
		Richest	98.1 (96.3 to 99.0)	99.4 (98.6 to 99.7)	99.6 (98.7 to 99.9)	100.0	100.0
	Liberia	Poorest	48.2 (40.3 to 56.1)	90.8 (88.8 to 92.6)	97.0 (95.4 to 98.0)	100.0	100.0
		Poorer	57.0 (49.8 to 64.0)	94.4 (93.1 to 95.5)	98.4 (97.5 to 99.0)	100.0	100.0
		Middle	62.9 (55.8 to 69.5)	96.3 (95.2 to 97.1)	99.0 (98.4 to 99.4)	100.0	100.0
		Richer	75.5 (69.0 to 81.0)	97.5 (96.5 to 98.1)	99.3 (98.8 to 99.6)	100.0	100.0
		Richest	88.0 (83.4 to 91.4)	98.8 (98.2 to 99.2)	99.6 (99.3 to 99.8)	100.0	100.0
	Madagascar	Poorest	41.3 (34.7 to 48.2)	90.7 (85.2 to 94.2)	97.3 (93.7 to 98.9)	100.0	99.9
		Poorer	55.4 (48.9 to 61.7)	95.3 (92.3 to 97.2)	98.8 (97.1 to 99.5)	100.0	100.0
		Middle	66.2 (60.2 to 71.7)	97.5 (95.7 to 98.5)	99.4 (98.6 to 99.8)	100.0	100.0
		Richer	81.4 (76.9 to 85.3)	98.6 (97.6 to 99.2)	99.6 (99.1 to 99.9)	100.0	100.0
		Richest	92.8 (90.4 to 94.6)	99.5 (99.0 to 99.7)	99.9 (99.6 to 99.9)	100.0	100.0
	Malawi	Poorest	89.0 (87.2 to 90.5)	97.8 (97.2 to 98.3)	99.1 (98.6 to 99.4)	100.0	100.0
		Poorer	89.9 (88.6 to 91.2)	98.3 (97.9 to 98.7)	99.3 (99.0 to 99.6)	100.0	100.0
		Middle	89.9 (88.5 to 91.1)	98.6 (98.2 to 98.9)	99.5 (99.2 to 99.7)	100.0	100.0
		Richer	92.6 (91.3 to 93.7)	98.8 (98.4 to 99.1)	99.5 (99.3 to 99.7)	100.0	100.0
		Richest	95.8 (94.9 to 96.6)	99.2 (99.0 to 99.5)	99.7 (99.5 to 99.8)	100.0	100.0

Indicators	Country	Wealth Quintile	Estimates and Projections of Immunization Coverage (%)			Probability Achieving Targets (%)	
			Year 2000	Year 2020	Year 2030	UHC2030	IA2030
	Mali	Poorest	56.5 (53.6 to 59.3)	77.4 (74.7 to 79.8)	84.7 (81.5 to 87.5)	99.7	0.0
		Poorer	62.5 (60.1 to 64.9)	84.0 (82.1 to 85.7)	90.3 (88.1 to 92.1)	100.0	60.8
		Middle	65.8 (63.5 to 68.0)	87.8 (86.3 to 89.2)	93.3 (91.7 to 94.6)	100.0	100.0
		Richer	75.9 (73.7 to 78.0)	90.7 (89.3 to 91.9)	94.5 (93.0 to 95.6)	100.0	100.0
		Richest	87.1 (85.3 to 88.7)	94.8 (93.8 to 95.7)	96.8 (95.7 to 97.6)	100.0	100.0
	Morocco	Poorest	96.1 (92.8 to 97.9)	99.2 (93.1 to 99.9)	99.7 (89.7 to 100.0)	99.0	97.2
		Poorer	97.2 (95.2 to 98.4)	99.6 (95.9 to 100.0)	99.8 (94.3 to 100.0)	99.6	98.7
		Middle	97.8 (95.9 to 98.9)	99.7 (97.3 to 100.0)	99.9 (96.5 to 100.0)	99.8	99.3
		Richer	98.8 (97.3 to 99.5)	99.8 (98.1 to 100.0)	99.9 (97.4 to 100.0)	99.9	99.5
		Richest	99.5 (98.6 to 99.8)	99.9 (99.0 to 100.0)	100.0 (98.6 to 100.0)	100.0	99.8
	Mozambique	Poorest	76.6 (72.3 to 80.5)	86.7 (81.7 to 90.5)	90.2 (83.0 to 94.5)	99.5	52.7
		Poorer	83.3 (80.1 to 86.0)	92.2 (89.1 to 94.5)	94.8 (90.7 to 97.1)	100.0	98.7
		Middle	87.2 (84.5 to 89.5)	95.1 (93.0 to 96.5)	97.0 (94.6 to 98.4)	100.0	100.0
		Richer	92.9 (91.0 to 94.5)	96.8 (95.4 to 97.8)	97.9 (96.1 to 98.9)	100.0	100.0
		Richest	97.1 (96.0 to 97.9)	98.6 (97.8 to 99.1)	99.0 (98.0 to 99.5)	100.0	100.0
	Namibia	Poorest	88.9 (85.7 to 91.5)	94.9 (92.1 to 96.7)	96.6 (93.1 to 98.3)	100.0	99.9
		Poorer	89.9 (87.6 to 91.9)	96.1 (94.1 to 97.4)	97.6 (95.2 to 98.8)	100.0	100.0
		Middle	89.9 (87.6 to 91.8)	96.7 (94.9 to 97.9)	98.1 (96.3 to 99.1)	100.0	100.0
		Richer	92.6 (90.5 to 94.3)	97.1 (95.5 to 98.2)	98.2 (96.4 to 99.2)	100.0	100.0
		Richest	95.9 (94.2 to 97.1)	98.2 (97.0 to 99.0)	98.9 (97.5 to 99.5)	100.0	100.0
	Niger	Poorest	27.7 (22.6 to 33.5)	91.7 (88.9 to 93.9)	98.4 (97.1 to 99.1)	100.0	100.0
		Poorer	32.3 (27.0 to 38.1)	94.3 (92.3 to 95.7)	99.0 (98.2 to 99.4)	100.0	100.0
		Middle	34.6 (29.3 to 40.4)	95.6 (94.1 to 96.8)	99.3 (98.7 to 99.6)	100.0	100.0
		Richer	45.6 (39.4 to 51.8)	96.6 (95.3 to 97.5)	99.4 (98.9 to 99.7)	100.0	100.0
		Richest	63.4 (57.0 to 69.4)	98.1 (97.3 to 98.7)	99.6 (99.4 to 99.8)	100.0	100.0
	Nigeria	Poorest	10.7 (9.5 to 12.0)	33.5 (31.6 to 35.4)	50.8 (46.7 to 54.9)	0.0	0.0
		Poorer	19.9 (18.1 to 21.9)	55.5 (53.5 to 57.5)	73.7 (70.4 to 76.7)	0.0	0.0
		Middle	31.7 (29.2 to 34.2)	73.6 (71.8 to 75.2)	87.2 (85.2 to 89.0)	100.0	0.1
		Richer	55.1 (52.0 to 58.2)	85.8 (84.5 to 87.1)	93.1 (91.7 to 94.2)	100.0	100.0
		Richest	81.0 (78.5 to 83.2)	94.9 (94.1 to 95.5)	97.5 (96.8 to 98.0)	100.0	100.0
	Rwanda	Poorest	94.4 (92.8 to 95.7)	97.8 (96.7 to 98.6)	98.7 (97.4 to 99.3)	100.0	100.0
		Poorer	94.8 (93.5 to 95.8)	98.3 (97.5 to 98.9)	99.0 (98.2 to 99.5)	100.0	100.0
		Middle	94.6 (93.3 to 95.7)	98.5 (97.8 to 99.0)	99.2 (98.5 to 99.6)	100.0	100.0
		Richer	96.0 (94.8 to 96.9)	98.7 (98.0 to 99.1)	99.3 (98.5 to 99.6)	100.0	100.0
		Richest	97.7 (96.9 to 98.4)	99.2 (98.7 to 99.5)	99.5 (99.0 to 99.8)	100.0	100.0
	Sao Tome and Principe	Poorest	90.5 (75.3 to 96.7)	98.3 (91.7 to 99.7)	99.3 (89.4 to 100.0)	99.3	97.2
		Poorer	91.4 (78.0 to 96.9)	98.7 (94.0 to 99.7)	99.5 (92.8 to 100.0)	99.7	98.6
		Middle	91.4 (77.3 to 97.0)	98.9 (95.0 to 99.8)	99.6 (94.5 to 100.0)	99.8	99.1
		Richer	93.8 (81.3 to 98.1)	99.1 (95.5 to 99.8)	99.7 (94.7 to 100.0)	99.8	99.2
		Richest	96.6 (87.3 to 99.1)	99.4 (96.9 to 99.9)	99.8 (96.3 to 100.0)	99.9	99.6
	Senegal	Poorest	85.8 (82.6 to 88.5)	93.0 (91.8 to 94.1)	95.2 (93.3 to 96.5)	100.0	100.0
		Poorer	89.1 (86.6 to 91.1)	95.5 (94.7 to 96.2)	97.2 (96.1 to 98.0)	100.0	100.0
		Middle	90.7 (88.6 to 92.5)	96.8 (96.2 to 97.4)	98.2 (97.4 to 98.7)	100.0	100.0
		Richer	94.4 (92.8 to 95.6)	97.7 (97.1 to 98.2)	98.6 (97.9 to 99.0)	100.0	100.0
		Richest	97.4 (96.5 to 98.1)	98.8 (98.4 to 99.1)	99.2 (98.8 to 99.5)	100.0	100.0
	Sierra Leone	Poorest	55.4 (45.7 to 64.7)	97.4 (96.5 to 98.0)	99.5 (99.1 to 99.7)	100.0	100.0
		Poorer	55.7 (46.5 to 64.4)	97.8 (97.1 to 98.3)	99.6 (99.4 to 99.8)	100.0	100.0
		Middle	53.1 (44.2 to 61.9)	98.0 (97.3 to 98.4)	99.7 (99.5 to 99.8)	100.0	100.0
		Richer	59.3 (50.0 to 67.9)	98.1 (97.4 to 98.6)	99.7 (99.4 to 99.8)	100.0	100.0
		Richest	71.0 (61.9 to 78.6)	98.7 (98.2 to 99.1)	99.8 (99.6 to 99.9)	100.0	100.0
	South Africa	Poorest	78.0 (38.7 to 95.3)	92.7 (87.2 to 95.9)	96.0 (82.7 to 99.2)	98.5	88.5
		Poorer	78.0 (38.8 to 95.3)	93.8 (89.7 to 96.3)	96.9 (86.6 to 99.3)	99.4	94.0
		Middle	76.0 (35.7 to 94.8)	94.1 (90.4 to 96.5)	97.3 (88.4 to 99.4)	99.7	96.0
		Richer	80.1 (40.3 to 96.1)	94.4 (90.2 to 96.8)	97.2 (87.7 to 99.4)	99.6	95.3
		Richest	87.0 (51.3 to 97.7)	96.1 (92.3 to 98.1)	97.9 (90.4 to 99.6)	99.8	97.8
	Tanzania	Poorest	85.0 (81.0 to 88.2)	96.2 (94.7 to 97.3)	98.2 (96.8 to 99.0)	100.0	100.0
		Poorer	87.1 (83.9 to 89.8)	97.3 (96.3 to 98.0)	98.8 (97.9 to 99.3)	100.0	100.0
		Middle	87.9 (84.8 to 90.4)	97.9 (97.1 to 98.5)	99.2 (98.5 to 99.5)	100.0	100.0
		Richer	91.7 (89.1 to 93.7)	98.3 (97.6 to 98.8)	99.3 (98.6 to 99.6)	100.0	100.0
		Richest	95.7 (93.9 to 96.9)	99.0 (98.5 to 99.4)	99.5 (99.1 to 99.8)	100.0	100.0
	Togo	Poorest	77.4 (45.5 to 93.2)	95.5 (90.3 to 98.0)	98.2 (89.3 to 99.7)	99.6	97.0

Indicators	Country	Wealth Quintile	Estimates and Projections of Immunization Coverage (%)			Probability Achieving Targets (%)	
			Year 2000	Year 2020	Year 2030	UHC2030	IA2030
		Poorer	81.1 (51.0 to 94.5)	97.0 (93.5 to 98.6)	98.9 (93.3 to 99.8)	99.9	99.2
		Middle	82.7 (53.3 to 95.2)	97.7 (95.1 to 99.0)	99.2 (95.3 to 99.9)	100.0	99.7
		Richer	88.4 (63.5 to 97.0)	98.2 (96.0 to 99.2)	99.3 (96.0 to 99.9)	100.0	99.8
		Richest	94.1 (77.4 to 98.6)	99.0 (97.7 to 99.6)	99.6 (97.6 to 99.9)	100.0	100.0
	Uganda	Poorest	79.7 (76.6 to 82.5)	97.8 (97.2 to 98.3)	99.3 (99.0 to 99.5)	100.0	100.0
		Poorer	78.2 (75.5 to 80.7)	98.0 (97.4 to 98.4)	99.4 (99.2 to 99.6)	100.0	100.0
		Middle	74.5 (71.8 to 77.1)	97.9 (97.4 to 98.3)	99.5 (99.2 to 99.6)	100.0	100.0
		Richer	77.3 (74.4 to 79.9)	97.8 (97.2 to 98.3)	99.4 (99.1 to 99.6)	100.0	100.0
		Richest	83.8 (81.0 to 86.2)	98.4 (97.8 to 98.8)	99.5 (99.2 to 99.7)	100.0	100.0
	Zambia	Poorest	89.9 (87.6 to 91.8)	94.9 (93.6 to 95.9)	96.4 (94.8 to 97.5)	100.0	100.0
		Poorer	91.1 (89.4 to 92.6)	96.2 (95.4 to 96.9)	97.6 (96.5 to 98.3)	100.0	100.0
		Middle	91.4 (89.6 to 92.9)	96.9 (96.2 to 97.5)	98.2 (97.4 to 98.8)	100.0	100.0
		Richer	94.0 (92.4 to 95.2)	97.4 (96.7 to 98.0)	98.3 (97.5 to 98.9)	100.0	100.0
		Richest	96.8 (95.7 to 97.6)	98.5 (97.9 to 98.9)	99.0 (98.3 to 99.3)	100.0	100.0
	Zimbabwe	Poorest	64.1 (57.7 to 70.1)	93.3 (91.0 to 95.0)	97.5 (95.7 to 98.5)	100.0	100.0
		Poorer	63.9 (58.0 to 69.4)	94.3 (92.4 to 95.7)	98.0 (96.7 to 98.8)	100.0	100.0
		Middle	61.1 (55.0 to 66.7)	94.6 (92.8 to 95.9)	98.3 (97.1 to 99.0)	100.0	100.0
		Richer	66.4 (60.2 to 72.0)	94.7 (93.0 to 96.1)	98.2 (96.9 to 98.9)	100.0	100.0
		Richest	76.5 (70.5 to 81.5)	96.3 (94.9 to 97.4)	98.7 (97.7 to 99.2)	100.0	100.0
MCV1 immunization	Angola	Poorest	40.4 (14.8 to 72.1)	28.1 (19.5 to 38.6)	22.8 (7.1 to 53.9)	0.0	0.0
		Poorer	55.5 (24.3 to 82.7)	42.3 (31.6 to 53.9)	36.0 (12.7 to 68.8)	0.3	0.0
		Middle	68.4 (35.6 to 89.3)	57.7 (46.3 to 68.4)	52.0 (22.0 to 80.9)	3.0	0.1
		Richer	81.3 (52.6 to 94.4)	70.0 (59.4 to 78.9)	63.0 (30.7 to 87.0)	10.8	0.9
		Richest	92.0 (74.2 to 97.8)	83.2 (75.3 to 89.0)	76.4 (45.6 to 92.7)	38.0	7.0
	Benin	Poorest	49.3 (46.4 to 52.2)	56.9 (54.0 to 59.8)	60.6 (55.7 to 65.3)	0.0	0.0
		Poorer	56.1 (53.5 to 58.7)	64.0 (61.5 to 66.4)	67.7 (63.3 to 71.8)	0.0	0.0
		Middle	61.3 (58.8 to 63.8)	70.3 (68.0 to 72.5)	74.3 (70.4 to 77.8)	0.0	0.0
		Richer	69.6 (67.1 to 72.0)	74.3 (72.0 to 76.5)	76.5 (72.6 to 79.9)	2.3	0.0
		Richest	81.1 (78.9 to 83.1)	81.4 (79.3 to 83.4)	81.6 (78.0 to 84.7)	81.7	0.0
	Burkina Faso	Poorest	29.3 (25.7 to 33.3)	98.2 (97.5 to 98.7)	99.8 (99.7 to 99.9)	100.0	100.0
		Poorer	33.4 (30.0 to 37.0)	98.5 (98.0 to 98.9)	99.9 (99.8 to 99.9)	100.0	100.0
		Middle	36.3 (33.0 to 39.8)	98.8 (98.4 to 99.1)	99.9 (99.8 to 99.9)	100.0	100.0
		Richer	43.1 (39.2 to 47.0)	98.9 (98.5 to 99.2)	99.9 (99.8 to 99.9)	100.0	100.0
		Richest	56.6 (52.0 to 61.0)	99.2 (98.9 to 99.4)	99.9 (99.9 to 100.0)	100.0	100.0
	Burundi	Poorest	91.8 (86.1 to 95.3)	92.9 (90.5 to 94.8)	93.5 (87.8 to 96.6)	100.0	90.6
		Poorer	92.5 (87.5 to 95.6)	93.7 (91.7 to 95.2)	94.3 (89.3 to 97.0)	100.0	95.9
		Middle	92.8 (88.0 to 95.8)	94.4 (92.6 to 95.7)	95.0 (90.7 to 97.4)	100.0	98.5
		Richer	94.0 (89.8 to 96.6)	94.5 (92.7 to 95.9)	94.8 (90.1 to 97.3)	100.0	97.7
		Richest	96.1 (93.1 to 97.9)	95.7 (94.0 to 96.9)	95.4 (91.1 to 97.7)	100.0	99.0
	Cameroon	Poorest	52.1 (48.2 to 56.0)	52.0 (48.6 to 55.4)	52.0 (46.2 to 57.7)	0.0	0.0
		Poorer	60.8 (57.4 to 64.1)	61.2 (58.4 to 64.0)	61.5 (56.1 to 66.5)	0.0	0.0
		Middle	67.6 (64.4 to 70.6)	69.5 (67.0 to 71.9)	70.4 (65.7 to 74.8)	0.0	0.0
		Richer	76.6 (73.6 to 79.3)	75.1 (72.6 to 77.5)	74.4 (69.7 to 78.6)	0.4	0.0
		Richest	86.9 (84.6 to 88.9)	83.2 (80.8 to 85.4)	81.1 (76.8 to 84.7)	70.4	0.0
	Chad	Poorest	11.4 (9.0 to 14.3)	71.1 (67.5 to 74.4)	91.5 (88.7 to 93.7)	100.0	86.2
		Poorer	12.7 (10.3 to 15.5)	73.9 (70.8 to 76.8)	92.6 (90.1 to 94.5)	100.0	98.0
		Middle	13.4 (11.0 to 16.1)	76.4 (73.5 to 79.1)	93.7 (91.5 to 95.3)	100.0	99.9
		Richer	16.1 (13.4 to 19.2)	77.3 (74.1 to 80.1)	93.5 (91.1 to 95.2)	100.0	99.7
		Richest	23.5 (19.8 to 27.7)	81.5 (78.4 to 84.3)	94.4 (92.2 to 96.0)	100.0	100.0
	Comoros	Poorest	51.9 (22.9 to 79.7)	73.9 (53.2 to 87.5)	82.1 (39.1 to 97.0)	55.4	24.4
		Poorer	58.3 (28.0 to 83.6)	79.0 (60.7 to 90.1)	86.0 (46.7 to 97.7)	67.0	34.6
		Middle	63.1 (32.0 to 86.2)	83.1 (67.1 to 92.2)	89.3 (54.5 to 98.3)	77.5	46.8
		Richer	70.9 (39.7 to 90.1)	85.6 (70.9 to 93.5)	90.3 (57.1 to 98.5)	80.6	51.1
		Richest	81.9 (54.1 to 94.6)	89.9 (78.0 to 95.7)	92.6 (63.9 to 98.9)	87.6	63.0
	Congo Brazzaville	Poorest	41.2 (33.9 to 48.9)	73.8 (67.1 to 79.5)	84.9 (75.4 to 91.2)	86.6	6.6
		Poorer	50.9 (43.8 to 57.9)	81.0 (75.3 to 85.6)	89.6 (82.2 to 94.2)	99.2	44.8
		Middle	59.1 (52.4 to 65.4)	86.4 (81.6 to 90.1)	93.0 (87.5 to 96.3)	100.0	88.4
		Richer	70.1 (64.0 to 75.6)	89.8 (85.5 to 92.9)	94.4 (89.5 to 97.1)	100.0	96.5
		Richest	83.2 (78.5 to 87.0)	93.7 (90.5 to 95.9)	96.3 (92.6 to 98.2)	100.0	99.8
		Poorest	39.9 (34.0 to 46.1)	71.3 (66.8 to 75.4)	82.8 (76.2 to 87.8)	81.0	0.1
		Poorer	46.1 (40.2 to 52.1)	76.6 (72.8 to 80.0)	86.5 (81.0 to 90.6)	98.8	4.9

Indicators	Country	Wealth Quintile	Estimates and Projections of Immunization Coverage (%)			Probability Achieving Targets (%)	
			Year 2000	Year 2020	Year 2030	UHC2030	IA2030
	Congo Democratic Republic	Middle	50.9 (45.0 to 56.9)	81.0 (77.6 to 83.9)	89.6 (85.1 to 92.9)	100.0	41.9
		Richer	59.5 (53.5 to 65.2)	83.6 (80.4 to 86.4)	90.5 (86.2 to 93.5)	100.0	60.1
		Richest	72.9 (67.5 to 77.8)	88.3 (85.6 to 90.6)	92.7 (89.1 to 95.2)	100.0	93.8
	Cote d'Ivoire	Poorest	42.9 (20.7 to 68.4)	59.7 (37.6 to 78.4)	67.5 (24.6 to 92.9)	24.0	5.8
		Poorer	49.6 (25.5 to 73.9)	66.5 (45.0 to 82.7)	73.8 (30.8 to 94.6)	35.1	10.4
		Middle	54.8 (29.6 to 77.9)	72.4 (52.2 to 86.3)	79.4 (38.1 to 96.0)	48.4	17.8
		Richer	63.6 (37.4 to 83.6)	76.2 (57.0 to 88.5)	81.2 (40.9 to 96.4)	53.3	21.2
		Richest	76.5 (52.3 to 90.7)	82.8 (66.5 to 92.1)	85.4 (48.3 to 97.3)	66.0	31.9
	Egypt	Poorest	97.4 (96.9 to 97.9)	76.9 (72.6 to 80.7)	49.6 (40.2 to 59.0)	0.0	0.0
		Poorer	97.7 (97.2 to 98.0)	78.8 (75.2 to 82.0)	52.5 (43.5 to 61.4)	0.0	0.0
		Middle	97.7 (97.3 to 98.1)	80.3 (77.1 to 83.2)	55.8 (47.0 to 64.3)	0.0	0.0
		Richer	98.1 (97.7 to 98.4)	80.6 (77.2 to 83.5)	54.1 (45.2 to 62.8)	0.0	0.0
		Richest	98.8 (98.4 to 99.0)	83.9 (80.6 to 86.7)	57.1 (47.8 to 65.9)	0.0	0.0
	Eswatini	Poorest	83.3 (68.9 to 91.8)	94.9 (76.7 to 99.1)	97.3 (66.5 to 99.9)	93.3	82.9
		Poorer	85.8 (73.5 to 92.9)	95.9 (80.8 to 99.2)	97.8 (71.8 to 99.9)	95.2	86.7
		Middle	87.4 (75.9 to 93.8)	96.6 (83.9 to 99.4)	98.3 (76.5 to 99.9)	96.6	89.9
		Richer	90.2 (80.0 to 95.5)	97.0 (85.3 to 99.5)	98.4 (77.1 to 99.9)	96.8	90.3
		Richest	94.1 (86.4 to 97.5)	97.8 (88.7 to 99.6)	98.7 (80.6 to 99.9)	97.6	92.6
	Ethiopia	Poorest	18.0 (16.3 to 19.8)	51.7 (49.2 to 54.3)	70.3 (66.7 to 73.7)	0.0	0.0
		Poorer	22.5 (20.7 to 24.3)	59.1 (56.8 to 61.4)	76.4 (73.2 to 79.3)	0.7	0.0
		Middle	26.5 (24.7 to 28.4)	65.9 (63.6 to 68.0)	81.7 (79.0 to 84.1)	89.3	0.0
		Richer	34.3 (32.1 to 36.5)	70.3 (67.9 to 72.5)	83.4 (80.7 to 85.8)	99.3	0.0
		Richest	49.5 (46.8 to 52.2)	78.2 (76.0 to 80.3)	87.3 (84.9 to 89.4)	100.0	0.4
	Gabon	Poorest	44.3 (40.2 to 48.5)	83.0 (79.4 to 86.1)	92.4 (89.2 to 94.7)	100.0	93.8
		Poorer	48.4 (44.9 to 52.0)	85.5 (82.3 to 88.2)	93.7 (90.9 to 95.6)	100.0	99.4
		Middle	51.1 (47.4 to 54.7)	87.6 (84.5 to 90.1)	94.8 (92.4 to 96.5)	100.0	100.0
		Richer	57.5 (53.0 to 61.8)	88.5 (85.1 to 91.3)	94.9 (92.2 to 96.7)	100.0	99.9
		Richest	69.5 (64.3 to 74.2)	91.3 (88.1 to 93.7)	95.8 (93.2 to 97.4)	100.0	100.0
	Gambia	Poorest	95.3 (91.3 to 97.5)	91.9 (89.9 to 93.5)	89.5 (82.8 to 93.7)	99.5	42.0
		Poorer	94.8 (90.6 to 97.2)	91.3 (89.5 to 92.8)	88.7 (81.8 to 93.2)	99.1	32.1
		Middle	93.9 (89.1 to 96.7)	90.5 (88.5 to 92.1)	88.1 (80.8 to 92.9)	98.4	24.9
		Richer	93.8 (88.9 to 96.7)	88.8 (86.2 to 90.9)	85.1 (76.1 to 91.0)	88.4	6.0
		Richest	95.1 (90.9 to 97.4)	89.1 (85.9 to 91.7)	84.1 (74.2 to 90.7)	81.7	4.4
	Ghana	Poorest	76.0 (70.8 to 80.5)	91.0 (87.9 to 93.4)	94.8 (91.2 to 97.0)	100.0	99.3
		Poorer	79.1 (74.8 to 82.8)	92.5 (90.0 to 94.4)	95.7 (92.8 to 97.5)	100.0	99.9
		Middle	81.0 (76.8 to 84.5)	93.7 (91.5 to 95.4)	96.6 (94.1 to 98.0)	100.0	100.0
		Richer	84.8 (80.7 to 88.1)	94.3 (92.1 to 95.9)	96.6 (94.1 to 98.1)	100.0	100.0
		Richest	90.5 (87.1 to 93.1)	95.8 (93.9 to 97.2)	97.3 (95.1 to 98.5)	100.0	100.0
	Guinea	Poorest	49.1 (44.5 to 53.8)	31.5 (28.4 to 34.9)	24.1 (19.7 to 29.2)	0.0	0.0
		Poorer	55.4 (51.1 to 59.6)	37.7 (34.7 to 40.8)	29.7 (24.8 to 35.1)	0.0	0.0
		Middle	60.0 (55.9 to 64.1)	44.0 (41.0 to 47.1)	36.3 (30.8 to 42.1)	0.0	0.0
		Richer	67.9 (63.8 to 71.8)	48.4 (45.1 to 51.8)	38.5 (32.8 to 44.5)	0.0	0.0
		Richest	79.5 (75.9 to 82.7)	58.2 (54.2 to 62.0)	45.4 (38.9 to 52.1)	0.0	0.0
	Kenya	Poorest	54.0 (49.4 to 58.6)	85.7 (83.5 to 87.6)	93.1 (90.9 to 94.8)	100.0	99.6
		Poorer	63.4 (59.3 to 67.4)	90.0 (88.5 to 91.4)	95.4 (93.8 to 96.6)	100.0	100.0
		Middle	70.7 (67.0 to 74.2)	93.1 (91.9 to 94.2)	97.0 (95.8 to 97.8)	100.0	100.0
		Richer	79.6 (76.4 to 82.6)	94.9 (93.8 to 95.8)	97.6 (96.6 to 98.3)	100.0	100.0
		Richest	89.2 (86.9 to 91.1)	96.9 (96.1 to 97.6)	98.4 (97.7 to 98.9)	100.0	100.0
	Lesotho	Poorest	72.8 (66.5 to 78.4)	86.4 (81.4 to 90.2)	90.7 (83.6 to 94.9)	99.7	59.5
		Poorer	76.2 (70.8 to 80.9)	88.6 (84.4 to 91.7)	92.3 (86.4 to 95.8)	100.0	81.4
		Middle	78.3 (73.1 to 82.7)	90.3 (86.7 to 93.1)	93.8 (88.8 to 96.6)	100.0	94.2
		Richer	82.6 (77.7 to 86.5)	91.2 (87.6 to 93.8)	93.9 (88.9 to 96.7)	100.0	94.6
		Richest	89.0 (85.0 to 92.0)	93.5 (90.4 to 95.6)	95.0 (90.6 to 97.4)	100.0	98.4
	Liberia	Poorest	44.9 (39.0 to 50.9)	67.8 (64.5 to 71.0)	77.3 (71.8 to 81.9)	13.3	0.0
		Poorer	51.9 (46.3 to 57.4)	74.1 (71.3 to 76.7)	82.3 (77.7 to 86.2)	84.6	0.0
		Middle	57.4 (51.9 to 62.7)	79.3 (76.6 to 81.7)	86.6 (82.6 to 89.8)	99.9	1.6
		Richer	66.2 (60.8 to 71.2)	82.5 (79.6 to 85.1)	88.0 (84.0 to 91.1)	100.0	11.1
		Richest	78.7 (73.9 to 82.8)	87.8 (85.1 to 90.1)	91.0 (87.4 to 93.6)	100.0	72.2
	Madagascar	Poorest	31.8 (26.6 to 37.4)	77.6 (68.9 to 84.4)	90.4 (81.5 to 95.3)	98.6	54.9
		Poorer	43.1 (37.5 to 48.9)	85.2 (78.6 to 90.0)	94.1 (88.0 to 97.2)	100.0	92.5
		Middle	53.7 (48.2 to 59.2)	90.5 (85.7 to 93.8)	96.4 (92.5 to 98.3)	100.0	99.7

Indicators	Country	Wealth Quintile	Estimates and Projections of Immunization Coverage (%)			Probability Achieving Targets (%)	
			Year 2000	Year 2020	Year 2030	UHC2030	IA2030
		Richer	67.5 (62.3 to 72.3)	93.5 (89.9 to 95.9)	97.4 (94.4 to 98.8)	100.0	100.0
		Richest	82.8 (79.0 to 86.0)	96.4 (94.2 to 97.8)	98.5 (96.5 to 99.3)	100.0	100.0
	Malawi	Poorest	75.3 (73.1 to 77.5)	92.6 (91.4 to 93.6)	96.2 (95.1 to 97.0)	100.0	100.0
		Poorer	78.3 (76.5 to 80.0)	93.8 (92.8 to 94.6)	96.8 (96.0 to 97.5)	100.0	100.0
		Middle	80.0 (78.4 to 81.6)	94.7 (93.9 to 95.4)	97.4 (96.7 to 98.0)	100.0	100.0
		Richer	83.8 (82.2 to 85.3)	95.2 (94.3 to 95.9)	97.4 (96.7 to 98.0)	100.0	100.0
		Richest	89.7 (88.3 to 91.0)	96.4 (95.6 to 97.0)	97.9 (97.2 to 98.4)	100.0	100.0
	Mali	Poorest	43.6 (41.0 to 46.2)	69.0 (66.3 to 71.6)	79.1 (75.7 to 82.2)	29.2	0.0
		Poorer	48.1 (45.8 to 50.4)	73.2 (71.0 to 75.4)	82.5 (79.6 to 85.0)	95.5	0.0
		Middle	51.2 (49.0 to 53.4)	76.9 (74.9 to 78.7)	85.5 (83.1 to 87.7)	100.0	0.0
		Richer	58.1 (55.7 to 60.4)	78.8 (76.8 to 80.7)	85.9 (83.3 to 88.1)	100.0	0.0
		Richest	70.3 (67.8 to 72.7)	83.7 (81.7 to 85.5)	88.3 (85.9 to 90.4)	100.0	5.7
	Morocco	Poorest	80.0 (72.0 to 86.2)	93.1 (62.5 to 99.1)	96.2 (47.5 to 99.9)	86.4	73.0
		Poorer	85.8 (80.0 to 90.2)	95.5 (72.2 to 99.4)	97.5 (58.7 to 99.9)	91.4	81.1
		Middle	89.6 (84.7 to 93.0)	97.0 (79.8 to 99.6)	98.4 (69.3 to 99.9)	94.9	87.6
		Richer	93.5 (89.6 to 96.0)	97.8 (84.6 to 99.7)	98.8 (74.3 to 100.0)	96.2	90.3
		Richest	96.9 (94.4 to 98.3)	98.7 (90.3 to 99.9)	99.2 (81.7 to 100.0)	97.8	94.0
	Mozambique	Poorest	59.8 (55.4 to 64.2)	73.5 (67.4 to 78.8)	79.1 (70.0 to 86.0)	41.1	0.0
		Poorer	70.2 (66.5 to 73.6)	81.8 (77.3 to 85.5)	86.1 (79.4 to 90.9)	96.4	6.0
		Middle	77.8 (74.7 to 80.6)	87.8 (84.5 to 90.4)	91.1 (86.5 to 94.3)	100.0	70.3
		Richer	85.9 (83.3 to 88.1)	91.3 (88.7 to 93.4)	93.3 (89.5 to 95.7)	100.0	96.0
		Richest	93.2 (91.5 to 94.6)	95.0 (93.3 to 96.3)	95.8 (93.2 to 97.4)	100.0	100.0
	Namibia	Poorest	75.1 (71.0 to 78.8)	90.5 (87.2 to 93.0)	94.4 (91.0 to 96.6)	100.0	99.2
		Poorer	77.3 (74.2 to 80.2)	91.7 (89.0 to 93.7)	95.2 (92.2 to 97.0)	100.0	99.9
		Middle	78.4 (75.5 to 81.0)	92.6 (90.2 to 94.5)	95.9 (93.4 to 97.5)	100.0	100.0
		Richer	81.8 (78.8 to 84.4)	92.9 (90.5 to 94.8)	95.7 (93.1 to 97.4)	100.0	100.0
		Richest	87.8 (85.1 to 90.2)	94.5 (92.2 to 96.1)	96.3 (93.8 to 97.9)	100.0	100.0
	Niger	Poorest	20.6 (16.9 to 24.9)	80.4 (75.9 to 84.3)	94.2 (91.0 to 96.3)	100.0	99.3
		Poorer	24.2 (20.3 to 28.6)	83.8 (80.0 to 87.0)	95.4 (92.8 to 97.1)	100.0	100.0
		Middle	27.0 (22.9 to 31.5)	86.5 (83.3 to 89.2)	96.4 (94.3 to 97.7)	100.0	100.0
		Richer	33.3 (28.6 to 38.3)	88.0 (85.0 to 90.5)	96.6 (94.6 to 97.8)	100.0	100.0
		Richest	46.6 (41.0 to 52.3)	91.2 (88.8 to 93.2)	97.3 (95.6 to 98.3)	100.0	100.0
	Nigeria	Poorest	10.6 (9.5 to 11.8)	27.6 (26.0 to 29.3)	40.6 (37.0 to 44.3)	0.0	0.0
		Poorer	18.0 (16.4 to 19.7)	41.9 (40.1 to 43.7)	56.6 (53.0 to 60.2)	0.0	0.0
		Middle	27.6 (25.5 to 29.8)	57.4 (55.5 to 59.2)	71.7 (68.5 to 74.6)	0.0	0.0
		Richer	43.6 (40.9 to 46.4)	69.8 (68.0 to 71.5)	80.0 (77.3 to 82.4)	49.7	0.0
		Richest	67.1 (64.2 to 69.8)	83.1 (81.7 to 84.5)	88.4 (86.5 to 90.2)	100.0	3.8
	Rwanda	Poorest	77.2 (74.7 to 79.6)	89.6 (87.5 to 91.3)	93.2 (90.8 to 95.0)	100.0	99.4
		Poorer	76.9 (74.6 to 79.1)	89.6 (87.7 to 91.3)	93.3 (91.0 to 95.0)	100.0	99.6
		Middle	75.5 (73.3 to 77.7)	89.6 (87.7 to 91.2)	93.5 (91.2 to 95.2)	100.0	99.8
		Richer	76.9 (74.5 to 79.2)	88.7 (86.6 to 90.5)	92.3 (89.7 to 94.3)	100.0	96.0
		Richest	82.4 (80.1 to 84.4)	89.9 (87.8 to 91.7)	92.5 (89.7 to 94.5)	100.0	96.2
	Sao Tome and Principe	Poorest	69.9 (46.6 to 85.9)	89.5 (66.7 to 97.4)	94.2 (55.4 to 99.6)	86.0	67.7
		Poorer	73.5 (51.5 to 87.7)	91.2 (71.5 to 97.8)	95.3 (61.0 to 99.6)	89.5	73.4
		Middle	75.7 (54.1 to 89.0)	92.6 (75.3 to 98.1)	96.2 (66.4 to 99.7)	92.3	78.9
		Richer	80.3 (59.6 to 91.7)	93.3 (77.0 to 98.3)	96.3 (66.8 to 99.7)	92.5	79.3
		Richest	87.4 (70.0 to 95.3)	95.0 (81.6 to 98.8)	96.9 (70.9 to 99.8)	94.4	83.5
	Senegal	Poorest	60.2 (56.5 to 63.8)	82.0 (80.3 to 83.5)	88.7 (86.4 to 90.7)	100.0	10.8
		Poorer	64.7 (61.4 to 67.9)	84.9 (83.5 to 86.3)	90.8 (88.9 to 92.4)	100.0	80.7
		Middle	67.7 (64.6 to 70.8)	87.4 (86.1 to 88.6)	92.6 (91.0 to 94.0)	100.0	99.9
		Richer	73.7 (70.6 to 76.5)	88.7 (87.3 to 89.9)	92.9 (91.1 to 94.3)	100.0	99.9
		Richest	82.9 (80.3 to 85.2)	91.6 (90.3 to 92.8)	94.3 (92.7 to 95.5)	100.0	100.0
	Sierra Leone	Poorest	57.5 (51.6 to 63.3)	77.8 (75.3 to 80.1)	84.9 (81.2 to 88.0)	99.4	0.0
		Poorer	57.5 (51.9 to 62.9)	78.1 (75.9 to 80.1)	85.3 (81.7 to 88.3)	99.7	0.1
		Middle	55.9 (50.4 to 61.3)	78.2 (76.1 to 80.2)	85.8 (82.3 to 88.7)	99.9	0.2
		Richer	58.1 (52.5 to 63.5)	76.9 (74.4 to 79.2)	83.8 (79.7 to 87.2)	96.6	0.0
		Richest	66.3 (60.7 to 71.5)	79.3 (76.5 to 81.8)	84.2 (79.9 to 87.7)	97.2	0.0
	South Africa	Poorest	66.6 (28.6 to 90.8)	86.0 (78.5 to 91.2)	91.5 (71.6 to 97.9)	91.1	59.7
		Poorer	68.7 (30.6 to 91.6)	87.4 (81.2 to 91.7)	92.5 (74.6 to 98.1)	93.9	66.6
		Middle	69.5 (31.2 to 91.9)	88.5 (82.9 to 92.5)	93.4 (77.4 to 98.3)	96.0	73.6
		Richer	73.4 (34.8 to 93.4)	88.7 (82.6 to 92.9)	93.0 (76.2 to 98.2)	95.1	70.7

Indicators	Country	Wealth Quintile	Estimates and Projections of Immunization Coverage (%)			Probability Achieving Targets (%)	
			Year 2000	Year 2020	Year 2030	UHC2030	IA2030
		Richest	81.2 (44.6 to 95.9)	90.9 (84.8 to 94.7)	93.8 (78.1 to 98.5)	96.5	76.3
	Tanzania	Poorest	66.1 (61.7 to 70.2)	80.9 (77.6 to 83.8)	86.2 (81.5 to 89.9)	99.4	2.1
		Poorer	73.6 (70.0 to 76.9)	86.1 (83.7 to 88.2)	90.2 (86.7 to 92.9)	100.0	55.6
		Middle	78.9 (75.8 to 81.8)	89.9 (88.1 to 91.5)	93.2 (90.7 to 95.1)	100.0	99.2
		Richer	85.5 (82.8 to 87.8)	92.2 (90.6 to 93.6)	94.4 (92.2 to 96.0)	100.0	100.0
		Richest	92.3 (90.4 to 93.8)	95.1 (93.9 to 96.1)	96.2 (94.4 to 97.4)	100.0	100.0
	Togo	Poorest	50.4 (22.8 to 77.6)	78.2 (64.1 to 87.9)	87.1 (57.0 to 97.2)	73.7	36.2
		Poorer	52.9 (24.6 to 79.3)	80.2 (67.2 to 89.0)	88.5 (60.4 to 97.5)	78.9	42.4
		Middle	53.9 (25.3 to 80.0)	81.9 (69.8 to 89.9)	89.9 (64.0 to 97.8)	83.7	49.5
		Richer	58.6 (28.9 to 83.1)	82.3 (70.2 to 90.2)	89.4 (62.7 to 97.7)	82.0	46.8
		Richest	69.1 (38.6 to 88.7)	85.6 (74.8 to 92.3)	90.6 (65.8 to 98.0)	86.0	53.4
	Uganda	Poorest	52.5 (49.3 to 55.6)	82.3 (80.3 to 84.1)	90.5 (88.5 to 92.2)	100.0	70.6
		Poorer	54.1 (51.3 to 56.9)	83.6 (81.8 to 85.1)	91.3 (89.5 to 92.9)	100.0	93.0
		Middle	54.2 (51.6 to 56.9)	84.6 (82.9 to 86.1)	92.2 (90.5 to 93.6)	100.0	99.3
		Richer	58.1 (55.3 to 60.9)	84.5 (82.6 to 86.1)	91.5 (89.6 to 93.1)	100.0	94.0
		Richest	67.9 (64.9 to 70.7)	87.0 (85.1 to 88.6)	92.2 (90.3 to 93.8)	100.0	98.7
	Zambia	Poorest	77.6 (74.8 to 80.2)	85.9 (84.1 to 87.6)	89.0 (86.4 to 91.2)	100.0	20.5
		Poorer	80.3 (78.0 to 82.4)	88.0 (86.6 to 89.4)	90.8 (88.6 to 92.6)	100.0	77.9
		Middle	81.9 (79.7 to 83.9)	89.7 (88.4 to 90.9)	92.4 (90.5 to 93.9)	100.0	99.3
		Richer	85.4 (83.2 to 87.3)	90.5 (89.1 to 91.8)	92.5 (90.4 to 94.1)	100.0	99.0
		Richest	90.7 (88.9 to 92.3)	92.8 (91.4 to 94.1)	93.7 (91.8 to 95.2)	100.0	100.0
	Zimbabwe	Poorest	51.0 (45.2 to 56.8)	84.6 (81.0 to 87.7)	92.7 (89.0 to 95.2)	100.0	93.3
		Poorer	54.8 (49.4 to 60.1)	86.8 (83.8 to 89.3)	93.8 (90.8 to 95.9)	100.0	99.1
		Middle	57.1 (51.7 to 62.3)	88.5 (85.9 to 90.7)	94.9 (92.3 to 96.6)	100.0	100.0
		Richer	63.0 (57.5 to 68.2)	89.3 (86.7 to 91.4)	94.9 (92.3 to 96.6)	100.0	99.9
		Richest	73.8 (68.5 to 78.6)	91.8 (89.5 to 93.6)	95.7 (93.4 to 97.2)	100.0	100.0
Polio3 immunization	Angola	Poorest	35.5 (11.5 to 70.2)	27.7 (18.7 to 38.7)	24.2 (6.8 to 57.7)	0.0	0.0
		Poorer	45.8 (16.7 to 78.4)	41.2 (29.8 to 53.4)	38.9 (12.8 to 73.1)	0.6	0.0
		Middle	57.3 (24.0 to 85.2)	50.2 (38.0 to 62.2)	46.6 (16.8 to 78.8)	2.0	0.1
		Richer	71.2 (36.7 to 91.5)	62.3 (50.0 to 73.1)	57.4 (23.7 to 85.2)	7.0	0.5
		Richest	84.6 (56.1 to 96.0)	73.7 (62.7 to 82.4)	66.7 (31.6 to 89.6)	17.5	2.1
	Benin	Poorest	63.5 (60.5 to 66.3)	62.7 (59.7 to 65.7)	62.4 (57.1 to 67.4)	0.0	0.0
		Poorer	70.2 (67.7 to 72.6)	73.1 (70.7 to 75.4)	74.5 (70.2 to 78.3)	0.2	0.0
		Middle	76.7 (74.5 to 78.7)	77.5 (75.4 to 79.5)	77.9 (74.0 to 81.4)	12.5	0.0
		Richer	84.3 (82.4 to 86.0)	83.3 (81.3 to 85.0)	82.7 (79.2 to 85.8)	93.9	0.0
		Richest	91.3 (89.9 to 92.5)	88.2 (86.4 to 89.7)	86.3 (83.0 to 89.0)	100.0	0.3
	Burkina Faso	Poorest	33.6 (29.2 to 38.2)	99.5 (99.2 to 99.7)	100.0 (100.0 to 100.0)	100.0	100.0
		Poorer	38.4 (34.4 to 42.6)	99.7 (99.5 to 99.8)	100.0 (100.0 to 100.0)	100.0	100.0
		Middle	44.3 (40.2 to 48.4)	99.7 (99.6 to 99.8)	100.0 (100.0 to 100.0)	100.0	100.0
		Richer	54.1 (49.6 to 58.6)	99.8 (99.7 to 99.9)	100.0 (100.0 to 100.0)	100.0	100.0
		Richest	67.8 (62.9 to 72.4)	99.8 (99.7 to 99.9)	100.0 (100.0 to 100.0)	100.0	100.0
	Burundi	Poorest	88.5 (79.4 to 93.9)	96.7 (95.0 to 97.8)	98.3 (95.9 to 99.3)	100.0	100.0
		Poorer	90.1 (82.3 to 94.7)	97.6 (96.5 to 98.4)	98.9 (97.4 to 99.5)	100.0	100.0
		Middle	91.8 (85.0 to 95.7)	97.9 (96.9 to 98.5)	98.9 (97.5 to 99.5)	100.0	100.0
		Richer	94.1 (88.8 to 97.0)	98.3 (97.5 to 98.9)	99.1 (97.9 to 99.6)	100.0	100.0
		Richest	96.5 (92.9 to 98.3)	98.7 (97.9 to 99.2)	99.2 (98.1 to 99.7)	100.0	100.0
	Cameroon	Poorest	67.4 (63.6 to 71.1)	66.6 (63.1 to 69.8)	66.1 (60.1 to 71.6)	0.0	0.0
		Poorer	73.3 (70.1 to 76.3)	75.8 (73.3 to 78.2)	77.0 (72.4 to 81.1)	8.0	0.0
		Middle	78.9 (76.1 to 81.4)	79.5 (77.3 to 81.6)	79.8 (75.5 to 83.5)	46.2	0.0
		Richer	85.6 (83.2 to 87.7)	84.5 (82.4 to 86.4)	84.0 (80.1 to 87.2)	97.8	0.0
		Richest	91.9 (90.2 to 93.4)	88.9 (86.9 to 90.6)	87.0 (83.4 to 90.0)	100.0	2.5
	Chad	Poorest	27.1 (22.8 to 32.0)	55.7 (51.6 to 59.8)	69.8 (63.1 to 75.8)	0.0	0.0
		Poorer	30.0 (25.7 to 34.6)	63.3 (59.6 to 66.7)	77.5 (71.9 to 82.3)	16.8	0.0
		Middle	33.6 (29.3 to 38.2)	64.9 (61.4 to 68.3)	78.0 (72.3 to 82.8)	21.4	0.0
		Richer	41.2 (36.5 to 46.0)	69.4 (65.7 to 72.8)	80.3 (74.8 to 84.8)	54.7	0.0
		Richest	53.8 (48.7 to 58.8)	74.2 (70.4 to 77.7)	82.0 (76.4 to 86.4)	76.9	0.0

Indicators	Country	Wealth Quintile	Estimates and Projections of Immunization Coverage (%)			Probability Achieving Targets (%)	
			Year 2000	Year 2020	Year 2030	UHC2030	IA2030
	Comoros	Poorest	53.7 (24.7 to 80.5)	77.2 (57.8 to 89.3)	85.3 (45.4 to 97.5)	64.8	32.4
		Poorer	59.0 (29.0 to 83.7)	83.3 (67.5 to 92.3)	90.3 (57.7 to 98.4)	81.0	51.4
		Middle	64.8 (34.0 to 86.9)	85.3 (70.8 to 93.2)	91.2 (60.3 to 98.6)	83.8	55.6
		Richer	73.3 (43.0 to 91.0)	88.5 (76.0 to 94.9)	92.8 (65.4 to 98.9)	88.7	64.5
		Richest	83.1 (56.6 to 94.9)	91.3 (80.9 to 96.3)	93.9 (69.2 to 99.1)	91.8	71.2
	Congo Brazzaville	Poorest	76.3 (69.7 to 82.0)	58.8 (50.3 to 66.9)	48.7 (32.9 to 64.7)	0.0	0.0
		Poorer	79.3 (73.6 to 84.0)	66.9 (58.6 to 74.2)	59.4 (42.7 to 74.1)	0.2	0.0
		Middle	82.4 (77.5 to 86.4)	69.1 (60.5 to 76.5)	60.7 (43.5 to 75.6)	0.4	0.0
		Richer	86.9 (82.9 to 90.1)	73.8 (65.1 to 81.0)	64.8 (47.0 to 79.1)	1.7	0.0
		Richest	91.9 (89.0 to 94.1)	78.7 (70.0 to 85.4)	67.8 (49.6 to 81.9)	4.9	0.0
	Congo Democratic Republic	Poorest	22.5 (18.3 to 27.2)	82.7 (79.4 to 85.5)	95.1 (92.8 to 96.7)	100.0	100.0
		Poorer	27.1 (22.6 to 32.2)	87.9 (85.5 to 90.0)	97.0 (95.5 to 98.0)	100.0	100.0
		Middle	32.9 (27.8 to 38.4)	89.7 (87.6 to 91.5)	97.4 (96.0 to 98.3)	100.0	100.0
		Richer	43.0 (37.2 to 49.1)	92.3 (90.5 to 93.8)	97.9 (96.8 to 98.7)	100.0	100.0
		Richest	58.3 (51.9 to 64.4)	94.4 (92.9 to 95.6)	98.3 (97.4 to 98.9)	100.0	100.0
	Cote d'Ivoire	Poorest	54.1 (27.8 to 78.5)	80.6 (61.5 to 91.5)	88.6 (52.1 to 98.2)	75.0	44.0
		Poorer	58.6 (31.6 to 81.4)	85.5 (69.8 to 93.8)	92.4 (63.1 to 98.8)	86.9	61.8
		Middle	63.6 (36.1 to 84.4)	86.9 (72.3 to 94.4)	92.8 (64.8 to 98.9)	88.4	64.4
		Richer	71.5 (44.6 to 88.8)	89.4 (76.8 to 95.6)	94.0 (68.8 to 99.1)	91.6	71.1
		Richest	81.3 (57.6 to 93.3)	91.8 (81.3 to 96.6)	94.7 (71.9 to 99.2)	93.6	76.0
	Egypt	Poorest	94.8 (93.7 to 95.7)	98.7 (98.1 to 99.1)	99.4 (98.9 to 99.7)	100.0	100.0
		Poorer	94.8 (93.8 to 95.5)	98.9 (98.5 to 99.2)	99.5 (99.1 to 99.7)	100.0	100.0
		Middle	94.8 (94.0 to 95.6)	98.8 (98.3 to 99.2)	99.4 (99.0 to 99.7)	100.0	100.0
		Richer	95.6 (94.7 to 96.4)	98.9 (98.4 to 99.2)	99.4 (99.0 to 99.7)	100.0	100.0
		Richest	96.9 (96.1 to 97.6)	99.0 (98.5 to 99.3)	99.4 (99.0 to 99.7)	100.0	100.0
	Eswatini	Poorest	89.4 (78.1 to 95.2)	98.2 (90.2 to 99.7)	99.3 (88.0 to 100.0)	99.0	96.5
		Poorer	89.0 (78.3 to 94.7)	98.4 (91.7 to 99.7)	99.4 (90.4 to 100.0)	99.4	97.6
		Middle	88.9 (78.1 to 94.7)	98.2 (91.0 to 99.7)	99.3 (89.1 to 100.0)	99.2	97.1
		Richer	90.3 (79.8 to 95.6)	98.3 (91.1 to 99.7)	99.3 (88.8 to 100.0)	99.2	96.9
		Richest	92.9 (83.4 to 97.0)	98.4 (91.4 to 99.7)	99.3 (88.0 to 100.0)	99.0	96.6
	Ethiopia	Poorest	25.5 (23.4 to 27.6)	55.8 (53.3 to 58.3)	70.9 (67.2 to 74.2)	0.0	0.0
		Poorer	29.4 (27.4 to 31.5)	64.7 (62.5 to 67.0)	79.4 (76.4 to 82.1)	33.4	0.0
		Middle	34.4 (32.3 to 36.5)	67.7 (65.5 to 69.9)	80.7 (77.9 to 83.3)	70.0	0.0
		Richer	43.5 (41.1 to 45.9)	73.2 (70.9 to 75.3)	83.7 (80.9 to 86.1)	99.5	0.0
		Richest	57.6 (54.9 to 60.2)	78.6 (76.4 to 80.7)	85.8 (83.2 to 88.1)	100.0	0.0
	Gabon	Poorest	35.8 (32.0 to 39.9)	58.9 (53.5 to 64.0)	69.6 (61.6 to 76.6)	0.1	0.0
		Poorer	33.3 (30.1 to 36.7)	60.4 (55.1 to 65.4)	72.7 (64.9 to 79.2)	1.3	0.0
		Middle	31.5 (28.3 to 34.9)	56.0 (50.3 to 61.6)	67.9 (59.2 to 75.5)	0.0	0.0
		Richer	33.1 (29.3 to 37.2)	54.8 (48.2 to 61.2)	65.5 (55.8 to 74.0)	0.0	0.0
		Richest	39.0 (33.8 to 44.4)	54.5 (46.8 to 62.0)	62.2 (51.3 to 72.0)	0.0	0.0
	Gambia	Poorest	94.2 (88.1 to 97.3)	94.9 (93.3 to 96.2)	95.3 (90.9 to 97.6)	100.0	98.7
		Poorer	93.9 (87.8 to 97.1)	95.5 (94.2 to 96.5)	96.1 (92.6 to 98.0)	100.0	99.8
		Middle	93.8 (87.6 to 97.0)	95.0 (93.5 to 96.1)	95.5 (91.3 to 97.7)	100.0	99.2
		Richer	94.5 (88.8 to 97.4)	95.0 (93.3 to 96.3)	95.3 (90.7 to 97.6)	100.0	98.6
		Richest	96.0 (91.4 to 98.2)	95.3 (93.2 to 96.7)	94.9 (89.7 to 97.5)	100.0	96.9
	Ghana	Poorest	75.6 (70.3 to 80.2)	92.8 (90.1 to 94.8)	96.3 (93.6 to 97.9)	100.0	100.0
		Poorer	78.1 (73.5 to 82.0)	94.6 (92.7 to 96.1)	97.5 (95.6 to 98.6)	100.0	100.0
		Middle	80.8 (76.5 to 84.5)	95.0 (93.1 to 96.4)	97.6 (95.7 to 98.6)	100.0	100.0
		Richer	85.3 (81.2 to 88.7)	95.9 (94.1 to 97.1)	97.9 (96.2 to 98.8)	100.0	100.0
		Richest	90.6 (87.1 to 93.2)	96.7 (95.1 to 97.8)	98.1 (96.4 to 99.0)	100.0	100.0
	Guinea	Poorest	51.3 (46.6 to 56.0)	42.4 (38.8 to 46.1)	38.1 (32.2 to 44.4)	0.0	0.0
		Poorer	57.6 (53.2 to 61.8)	53.0 (49.7 to 56.2)	50.7 (44.5 to 56.9)	0.0	0.0
		Middle	64.2 (60.1 to 68.2)	57.6 (54.4 to 60.6)	54.1 (47.9 to 60.2)	0.0	0.0
		Richer	73.5 (69.6 to 77.1)	65.0 (61.7 to 68.2)	60.3 (54.0 to 66.3)	0.0	0.0
		Richest	83.8 (80.6 to 86.5)	72.6 (69.0 to 75.9)	65.4 (58.8 to 71.5)	0.0	0.0
	Kenya	Poorest	59.3 (54.4 to 64.0)	93.1 (91.7 to 94.2)	97.6 (96.7 to 98.3)	100.0	100.0
		Poorer	65.0 (60.8 to 69.0)	95.3 (94.4 to 96.1)	98.5 (98.0 to 99.0)	100.0	100.0
		Middle	71.0 (67.1 to 74.5)	96.1 (95.2 to 96.8)	98.7 (98.2 to 99.1)	100.0	100.0
		Richer	79.0 (75.5 to 82.1)	97.1 (96.3 to 97.7)	99.0 (98.5 to 99.3)	100.0	100.0
		Richest	87.4 (84.6 to 89.7)	97.9 (97.3 to 98.4)	99.2 (98.8 to 99.5)	100.0	100.0
	Lesotho	Poorest	79.9 (74.4 to 84.5)	84.2 (78.5 to 88.5)	86.0 (76.3 to 92.2)	90.3	12.5

Indicators	Country	Wealth Quintile	Estimates and Projections of Immunization Coverage (%)			Probability Achieving Targets (%)	
			Year 2000	Year 2020	Year 2030	UHC2030	IA2030
		Poorer	80.9 (76.1 to 85.0)	87.1 (82.6 to 90.6)	89.5 (81.8 to 94.2)	99.0	43.5
		Middle	82.4 (77.7 to 86.2)	87.1 (82.6 to 90.6)	89.1 (81.1 to 93.9)	98.5	37.8
		Richer	85.7 (81.4 to 89.2)	88.5 (84.1 to 91.8)	89.7 (81.9 to 94.4)	99.0	46.2
		Richest	90.3 (86.6 to 93.0)	90.1 (85.7 to 93.2)	90.0 (82.0 to 94.7)	99.0	50.2
	Liberia	Poorest	44.5 (38.5 to 50.6)	74.2 (71.2 to 77.1)	84.5 (80.2 to 88.1)	97.9	0.1
		Poorer	49.3 (43.6 to 55.0)	80.6 (78.2 to 82.8)	89.6 (86.4 to 92.1)	100.0	38.5
		Middle	54.8 (49.2 to 60.3)	82.5 (80.0 to 84.7)	90.3 (87.1 to 92.8)	100.0	57.6
		Richer	63.8 (58.1 to 69.2)	85.9 (83.3 to 88.1)	91.9 (88.9 to 94.1)	100.0	89.9
		Richest	75.6 (70.2 to 80.2)	89.1 (86.4 to 91.2)	93.0 (90.0 to 95.1)	100.0	97.5
	Madagascar	Poorest	38.3 (32.3 to 44.6)	74.4 (64.5 to 82.3)	86.3 (73.8 to 93.4)	86.5	19.3
		Poorer	51.2 (45.1 to 57.2)	85.4 (78.5 to 90.4)	93.3 (86.0 to 96.9)	99.9	84.9
		Middle	64.6 (59.0 to 69.8)	90.2 (85.1 to 93.8)	95.4 (90.1 to 97.9)	100.0	97.7
		Richer	78.7 (74.3 to 82.6)	94.3 (90.9 to 96.5)	97.2 (93.8 to 98.8)	100.0	99.9
		Richest	90.0 (87.3 to 92.2)	96.9 (94.8 to 98.1)	98.3 (96.0 to 99.3)	100.0	100.0
	Malawi	Poorest	78.4 (76.2 to 80.4)	93.0 (91.9 to 94.0)	96.2 (95.1 to 97.1)	100.0	100.0
		Poorer	80.5 (78.7 to 82.1)	94.8 (93.9 to 95.5)	97.4 (96.7 to 98.0)	100.0	100.0
		Middle	82.8 (81.3 to 84.3)	95.1 (94.2 to 95.7)	97.5 (96.7 to 98.0)	100.0	100.0
		Richer	86.9 (85.4 to 88.2)	95.9 (95.1 to 96.5)	97.8 (97.1 to 98.3)	100.0	100.0
		Richest	91.6 (90.3 to 92.7)	96.7 (96.0 to 97.3)	98.0 (97.3 to 98.5)	100.0	100.0
	Mali	Poorest	43.3 (40.7 to 46.0)	70.6 (67.9 to 73.2)	81.0 (77.7 to 83.9)	72.9	0.0
		Poorer	47.6 (45.3 to 49.9)	77.3 (75.2 to 79.2)	86.8 (84.4 to 88.9)	100.0	0.1
		Middle	52.6 (50.4 to 54.8)	79.0 (77.1 to 80.8)	87.4 (85.1 to 89.4)	100.0	0.4
		Richer	61.3 (58.9 to 63.6)	82.7 (80.8 to 84.4)	89.2 (87.1 to 91.0)	100.0	21.2
		Richest	73.1 (70.7 to 75.4)	86.2 (84.4 to 87.9)	90.5 (88.3 to 92.3)	100.0	68.4
	Morocco	Poorest	88.7 (82.9 to 92.7)	97.5 (82.1 to 99.7)	98.8 (74.9 to 100.0)	96.3	90.7
		Poorer	92.1 (88.1 to 94.8)	98.5 (89.1 to 99.8)	99.4 (85.3 to 100.0)	98.4	95.5
		Middle	94.7 (91.5 to 96.7)	98.9 (91.9 to 99.9)	99.5 (88.4 to 100.0)	98.9	96.8
		Richer	96.9 (94.5 to 98.3)	99.3 (94.6 to 99.9)	99.7 (91.8 to 100.0)	99.4	98.0
		Richest	98.5 (97.0 to 99.3)	99.6 (96.5 to 100.0)	99.8 (94.0 to 100.0)	99.6	98.7
	Mozambique	Poorest	55.8 (51.3 to 60.3)	79.1 (73.7 to 83.6)	86.7 (80.0 to 91.4)	97.5	10.0
		Poorer	65.7 (61.9 to 69.4)	87.2 (83.7 to 90.1)	92.8 (88.8 to 95.4)	100.0	92.6
		Middle	74.9 (71.6 to 78.0)	90.6 (87.9 to 92.8)	94.6 (91.5 to 96.6)	100.0	99.6
		Richer	84.5 (81.8 to 86.9)	94.0 (92.0 to 95.5)	96.4 (94.1 to 97.7)	100.0	100.0
		Richest	92.3 (90.4 to 93.8)	96.3 (95.0 to 97.3)	97.5 (95.9 to 98.5)	100.0	100.0
	Namibia	Poorest	82.8 (79.4 to 85.7)	85.2 (80.6 to 88.9)	86.3 (79.0 to 91.3)	95.8	8.6
		Poorer	83.4 (80.6 to 85.8)	87.7 (84.1 to 90.6)	89.5 (83.8 to 93.4)	99.8	41.4
		Middle	84.3 (81.8 to 86.6)	87.4 (83.8 to 90.4)	88.8 (82.8 to 92.9)	99.6	30.6
		Richer	87.1 (84.6 to 89.2)	88.5 (84.9 to 91.4)	89.2 (83.2 to 93.2)	99.7	37.3
		Richest	91.0 (88.7 to 92.9)	89.9 (86.2 to 92.7)	89.3 (82.9 to 93.5)	99.6	39.3
	Niger	Poorest	26.3 (21.6 to 31.6)	89.7 (86.6 to 92.2)	97.7 (96.2 to 98.7)	100.0	100.0
		Poorer	29.9 (25.1 to 35.1)	92.5 (90.3 to 94.3)	98.5 (97.5 to 99.1)	100.0	100.0
		Middle	34.4 (29.4 to 39.7)	93.3 (91.2 to 94.8)	98.6 (97.7 to 99.2)	100.0	100.0
		Richer	42.9 (37.3 to 48.7)	94.6 (92.9 to 95.9)	98.8 (98.0 to 99.3)	100.0	100.0
		Richest	56.5 (50.3 to 62.4)	95.9 (94.5 to 96.9)	99.0 (98.3 to 99.4)	100.0	100.0
	Nigeria	Poorest	18.1 (16.4 to 19.8)	50.5 (48.5 to 52.4)	68.7 (65.4 to 71.8)	0.0	0.0
		Poorer	24.9 (23.0 to 26.9)	64.6 (62.9 to 66.3)	81.1 (78.7 to 83.2)	81.6	0.0
		Middle	33.9 (31.6 to 36.3)	72.0 (70.4 to 73.5)	85.2 (83.1 to 87.0)	100.0	0.0
		Richer	48.2 (45.4 to 51.0)	80.4 (79.0 to 81.8)	89.6 (88.0 to 91.1)	100.0	32.4
		Richest	66.9 (64.0 to 69.6)	87.3 (86.0 to 88.4)	92.7 (91.3 to 93.8)	100.0	100.0
	Rwanda	Poorest	84.2 (81.8 to 86.4)	96.6 (95.5 to 97.4)	98.5 (97.6 to 99.0)	100.0	100.0
		Poorer	84.1 (81.9 to 86.0)	97.1 (96.2 to 97.7)	98.8 (98.1 to 99.2)	100.0	100.0
		Middle	84.3 (82.3 to 86.1)	96.8 (95.9 to 97.5)	98.6 (97.9 to 99.1)	100.0	100.0
		Richer	86.4 (84.4 to 88.2)	97.0 (96.1 to 97.7)	98.6 (97.9 to 99.1)	100.0	100.0
		Richest	90.1 (88.2 to 91.7)	97.2 (96.3 to 97.9)	98.6 (97.7 to 99.1)	100.0	100.0
	Sao Tome and Principe	Poorest	77.1 (55.7 to 90.0)	94.8 (80.7 to 98.8)	97.7 (76.2 to 99.8)	96.4	88.2
		Poorer	78.6 (58.1 to 90.5)	95.9 (84.9 to 99.0)	98.4 (82.2 to 99.9)	98.1	92.8
		Middle	80.3 (60.3 to 91.5)	96.0 (85.2 to 99.0)	98.3 (81.9 to 99.9)	98.0	92.5
		Richer	84.2 (65.3 to 93.7)	96.5 (86.8 to 99.2)	98.4 (83.1 to 99.9)	98.3	93.3
		Richest	89.3 (73.0 to 96.2)	97.1 (88.3 to 99.3)	98.5 (83.5 to 99.9)	98.4	93.7
	Senegal	Poorest	63.5 (59.3 to 67.4)	90.8 (89.6 to 91.9)	95.9 (94.8 to 96.8)	100.0	100.0
		Poorer	69.0 (65.4 to 72.4)	93.8 (92.9 to 94.5)	97.5 (96.8 to 98.1)	100.0	100.0

Indicators	Country	Wealth Quintile	Estimates and Projections of Immunization Coverage (%)			Probability Achieving Targets (%)	
			Year 2000	Year 2020	Year 2030	UHC2030	IA2030
		Middle	74.6 (71.3 to 77.6)	94.7 (94.0 to 95.4)	97.8 (97.1 to 98.3)	100.0	100.0
		Richer	81.9 (79.0 to 84.4)	96.1 (95.4 to 96.7)	98.3 (97.7 to 98.7)	100.0	100.0
		Richest	89.3 (87.1 to 91.2)	97.2 (96.6 to 97.7)	98.6 (98.1 to 99.0)	100.0	100.0
	Sierra Leone	Poorest	50.5 (43.8 to 57.3)	89.1 (87.3 to 90.6)	95.8 (94.3 to 97.0)	100.0	100.0
		Poorer	50.5 (44.1 to 56.8)	90.6 (89.2 to 91.9)	96.7 (95.5 to 97.6)	100.0	100.0
		Middle	51.1 (44.9 to 57.3)	90.0 (88.5 to 91.3)	96.4 (95.0 to 97.3)	100.0	100.0
		Richer	55.6 (49.2 to 61.9)	90.5 (89.0 to 91.9)	96.3 (94.9 to 97.4)	100.0	100.0
		Richest	64.4 (57.7 to 70.5)	91.3 (89.6 to 92.8)	96.2 (94.7 to 97.3)	100.0	100.0
	South Africa	Poorest	55.5 (21.0 to 85.6)	77.9 (68.4 to 85.1)	85.5 (59.0 to 96.0)	71.0	27.6
		Poorer	54.3 (20.2 to 85.0)	80.0 (71.9 to 86.2)	88.0 (64.5 to 96.7)	80.6	38.3
		Middle	53.8 (19.8 to 84.8)	78.1 (69.7 to 84.6)	86.2 (60.9 to 96.1)	73.8	29.6
		Richer	57.2 (21.7 to 86.7)	78.3 (69.5 to 85.1)	85.6 (59.8 to 95.9)	71.6	27.4
		Richest	64.8 (27.0 to 90.3)	79.2 (69.5 to 86.4)	84.6 (57.5 to 95.7)	67.4	23.9
	Tanzania	Poorest	78.5 (74.5 to 81.9)	84.2 (80.9 to 87.0)	86.6 (81.2 to 90.6)	99.1	4.9
		Poorer	82.7 (79.6 to 85.5)	89.3 (87.1 to 91.2)	91.7 (88.2 to 94.2)	100.0	84.5
		Middle	86.6 (84.0 to 88.9)	91.1 (89.2 to 92.7)	92.8 (89.7 to 95.0)	100.0	96.5
		Richer	91.1 (89.0 to 92.8)	93.5 (91.9 to 94.8)	94.4 (91.9 to 96.2)	100.0	99.9
		Richest	95.1 (93.7 to 96.2)	95.4 (94.1 to 96.5)	95.6 (93.4 to 97.1)	100.0	100.0
	Togo	Poorest	64.1 (32.9 to 86.5)	91.2 (83.1 to 95.6)	96.1 (81.8 to 99.3)	98.2	88.0
		Poorer	65.3 (34.0 to 87.1)	92.8 (86.3 to 96.4)	97.1 (86.2 to 99.5)	99.3	93.9
		Middle	67.0 (35.7 to 88.1)	92.7 (86.2 to 96.3)	97.0 (85.5 to 99.4)	99.2	93.0
		Richer	72.0 (40.8 to 90.5)	93.5 (87.4 to 96.7)	97.1 (86.2 to 99.5)	99.3	93.8
		Richest	79.7 (50.6 to 93.7)	94.3 (88.8 to 97.2)	97.2 (86.3 to 99.5)	99.3	94.0
	Uganda	Poorest	55.2 (52.0 to 58.4)	88.6 (87.1 to 90.0)	95.1 (93.9 to 96.1)	100.0	100.0
		Poorer	54.5 (51.6 to 57.3)	90.0 (88.7 to 91.1)	96.1 (95.1 to 96.9)	100.0	100.0
		Middle	54.4 (51.7 to 57.1)	89.0 (87.7 to 90.3)	95.5 (94.4 to 96.4)	100.0	100.0
		Richer	58.1 (55.2 to 61.0)	89.3 (87.9 to 90.6)	95.4 (94.2 to 96.3)	100.0	100.0
		Richest	66.0 (62.9 to 69.0)	90.0 (88.4 to 91.4)	95.1 (93.7 to 96.2)	100.0	100.0
	Zambia	Poorest	71.6 (68.4 to 74.6)	86.4 (84.5 to 88.0)	91.0 (88.7 to 92.8)	100.0	81.2
		Poorer	75.5 (72.9 to 77.9)	90.2 (88.9 to 91.4)	94.1 (92.6 to 95.3)	100.0	100.0
		Middle	79.5 (77.1 to 81.6)	91.3 (90.1 to 92.4)	94.6 (93.1 to 95.7)	100.0	100.0
		Richer	85.0 (82.8 to 87.0)	93.2 (92.0 to 94.2)	95.5 (94.2 to 96.5)	100.0	100.0
		Richest	90.9 (89.1 to 92.5)	94.9 (93.8 to 95.8)	96.2 (94.9 to 97.2)	100.0	100.0
	Zimbabwe	Poorest	48.0 (42.3 to 53.7)	84.0 (80.4 to 87.1)	92.6 (89.1 to 95.1)	100.0	93.4
		Poorer	50.2 (44.9 to 55.5)	87.3 (84.5 to 89.7)	94.7 (92.1 to 96.5)	100.0	99.9
		Middle	53.2 (47.9 to 58.5)	87.6 (84.8 to 89.8)	94.6 (92.0 to 96.4)	100.0	99.9
		Richer	60.0 (54.4 to 65.3)	89.1 (86.6 to 91.2)	95.1 (92.6 to 96.7)	100.0	100.0
		Richest	70.3 (64.7 to 75.4)	90.9 (88.4 to 92.8)	95.3 (92.9 to 97.0)	100.0	100.0
DPT3 immunization	Angola	Poorest	20.3 (5.2 to 55.2)	18.0 (11.2 to 27.2)	16.9 (4.0 to 48.7)	0.0	0.0
		Poorer	29.9 (8.5 to 67.3)	30.0 (20.0 to 41.9)	30.0 (8.1 to 66.6)	0.2	0.0
		Middle	43.0 (14.0 to 78.5)	40.7 (28.7 to 53.6)	39.6 (11.9 to 75.3)	1.0	0.0
		Richer	58.2 (23.0 to 87.1)	53.1 (39.9 to 65.7)	50.6 (17.4 to 82.6)	4.1	0.3
		Richest	77.1 (41.9 to 94.3)	65.3 (52.1 to 76.3)	58.4 (22.4 to 86.7)	9.1	0.9
	Benin	Poorest	51.9 (49.0 to 54.9)	60.3 (57.3 to 63.2)	64.3 (59.3 to 69.0)	0.0	0.0
		Poorer	59.9 (57.2 to 62.5)	71.0 (68.6 to 73.3)	75.8 (71.9 to 79.4)	1.0	0.0
		Middle	68.4 (66.0 to 70.8)	76.4 (74.3 to 78.4)	79.8 (76.3 to 82.9)	45.4	0.0
		Richer	76.7 (74.4 to 78.8)	81.5 (79.5 to 83.3)	83.6 (80.4 to 86.3)	98.5	0.0
		Richest	86.8 (85.0 to 88.4)	85.7 (83.8 to 87.4)	85.2 (81.9 to 87.9)	99.9	0.0
	Burkina Faso	Poorest	27.6 (24.0 to 31.5)	98.8 (98.3 to 99.1)	99.9 (99.9 to 100.0)	100.0	100.0
		Poorer	31.3 (28.0 to 34.9)	99.1 (98.8 to 99.4)	99.9 (99.9 to 100.0)	100.0	100.0
		Middle	36.6 (33.2 to 40.2)	99.2 (98.9 to 99.4)	99.9 (99.9 to 100.0)	100.0	100.0
		Richer	43.3 (39.3 to 47.2)	99.4 (99.1 to 99.5)	100.0 (99.9 to 100.0)	100.0	100.0
		Richest	57.0 (52.3 to 61.5)	99.5 (99.2 to 99.6)	100.0 (99.9 to 100.0)	100.0	100.0
	Burundi	Poorest	89.9 (82.0 to 94.5)	97.5 (96.3 to 98.4)	98.8 (97.3 to 99.5)	100.0	100.0
		Poorer	89.4 (81.5 to 94.1)	97.8 (96.8 to 98.5)	99.0 (97.8 to 99.6)	100.0	100.0
		Middle	89.4 (81.6 to 94.1)	97.6 (96.5 to 98.3)	98.9 (97.5 to 99.5)	100.0	100.0
		Richer	89.8 (82.1 to 94.4)	97.4 (96.3 to 98.2)	98.7 (97.2 to 99.4)	100.0	100.0
		Richest	92.3 (86.0 to 95.9)	97.2 (95.9 to 98.1)	98.4 (96.3 to 99.3)	100.0	100.0
	Cameroon	Poorest	46.1 (42.2 to 50.0)	56.9 (53.4 to 60.3)	62.1 (56.4 to 67.5)	0.0	0.0
		Poorer	54.9 (51.3 to 58.4)	68.7 (66.0 to 71.3)	74.7 (70.2 to 78.7)	0.4	0.0

Indicators	Country	Wealth Quintile	Estimates and Projections of Immunization Coverage (%)			Probability Achieving Targets (%)	
			Year 2000	Year 2020	Year 2030	UHC2030	IA2030
		Middle	64.6 (61.3 to 67.8)	75.0 (72.6 to 77.2)	79.3 (75.4 to 82.8)	36.1	0.0
		Richer	74.1 (71.0 to 77.1)	80.8 (78.6 to 82.9)	83.6 (80.0 to 86.7)	97.6	0.0
		Richest	85.5 (83.1 to 87.7)	85.6 (83.4 to 87.5)	85.6 (82.0 to 88.6)	99.8	0.1
	Chad	Poorest	11.5 (9.0 to 14.6)	31.3 (27.5 to 35.3)	46.0 (37.8 to 54.3)	0.0	0.0
		Poorer	13.5 (10.9 to 16.6)	38.9 (35.1 to 42.9)	56.3 (48.1 to 64.1)	0.0	0.0
		Middle	16.4 (13.6 to 19.8)	42.2 (38.3 to 46.2)	58.5 (50.3 to 66.3)	0.0	0.0
		Richer	20.6 (17.2 to 24.5)	46.3 (42.0 to 50.7)	61.2 (52.8 to 68.9)	0.0	0.0
		Richest	31.1 (26.5 to 36.0)	50.6 (45.6 to 55.6)	60.7 (51.9 to 68.9)	0.0	0.0
	Comoros	Poorest	39.8 (15.1 to 71.4)	71.3 (49.4 to 86.2)	82.8 (39.1 to 97.2)	57.3	26.6
		Poorer	47.2 (19.4 to 77.1)	79.7 (61.2 to 90.6)	89.1 (52.6 to 98.3)	76.4	46.3
		Middle	56.0 (25.4 to 82.8)	83.5 (67.3 to 92.5)	91.0 (58.0 to 98.6)	82.4	54.7
		Richer	65.4 (33.2 to 88.0)	87.1 (73.0 to 94.4)	92.7 (63.4 to 98.9)	87.6	63.7
		Richest	78.7 (48.5 to 93.7)	90.0 (77.9 to 95.8)	93.4 (65.5 to 99.0)	89.4	67.3
	Congo Brazzaville	Poorest	59.2 (51.7 to 66.4)	33.9 (27.2 to 41.2)	23.3 (14.3 to 35.5)	0.0	0.0
		Poorer	66.9 (60.3 to 72.9)	45.4 (37.6 to 53.4)	34.8 (22.5 to 49.5)	0.0	0.0
		Middle	74.8 (69.3 to 79.6)	52.6 (44.1 to 60.9)	40.4 (26.6 to 55.9)	0.0	0.0
		Richer	82.0 (77.4 to 85.8)	60.3 (51.2 to 68.8)	46.8 (31.4 to 62.8)	0.0	0.0
		Richest	90.1 (87.1 to 92.5)	67.7 (58.1 to 76.0)	50.1 (33.5 to 66.6)	0.0	0.0
	Congo Democratic Republic	Poorest	15.8 (12.7 to 19.4)	61.7 (56.8 to 66.3)	82.5 (76.0 to 87.5)	79.2	0.1
		Poorer	20.7 (17.0 to 24.9)	72.4 (68.3 to 76.1)	89.3 (84.8 to 92.5)	100.0	34.4
		Middle	27.7 (23.2 to 32.7)	77.8 (74.2 to 81.0)	91.4 (87.6 to 94.0)	100.0	78.4
		Richer	37.0 (31.7 to 42.7)	82.8 (79.6 to 85.6)	93.2 (90.1 to 95.4)	100.0	97.8
		Richest	54.3 (48.0 to 60.4)	86.9 (84.0 to 89.3)	94.0 (91.1 to 96.0)	100.0	99.4
	Cote d'Ivoire	Poorest	33.3 (13.4 to 61.9)	64.8 (40.7 to 83.1)	78.0 (31.2 to 96.4)	45.1	18.0
		Poorer	39.0 (16.6 to 67.5)	73.4 (50.9 to 87.9)	85.1 (42.5 to 97.7)	63.6	32.7
		Middle	46.3 (21.1 to 73.8)	77.1 (56.1 to 89.9)	87.0 (46.5 to 98.1)	69.2	38.4
		Richer	54.8 (27.2 to 80.0)	81.0 (61.6 to 91.8)	88.8 (50.9 to 98.4)	75.0	45.1
		Richest	69.2 (40.5 to 88.3)	84.4 (66.9 to 93.5)	89.3 (52.0 to 98.4)	76.4	46.9
	Egypt	Poorest	90.9 (89.4 to 92.1)	97.7 (96.9 to 98.3)	98.9 (98.2 to 99.3)	100.0	100.0
		Poorer	91.7 (90.5 to 92.7)	98.2 (97.6 to 98.6)	99.2 (98.7 to 99.5)	100.0	100.0
		Middle	92.7 (91.7 to 93.6)	98.3 (97.7 to 98.7)	99.2 (98.7 to 99.5)	100.0	100.0
		Richer	94.0 (92.9 to 94.8)	98.4 (97.9 to 98.8)	99.2 (98.7 to 99.5)	100.0	100.0
		Richest	96.1 (95.3 to 96.8)	98.6 (98.0 to 98.9)	99.1 (98.6 to 99.5)	100.0	100.0
	Eswatini	Poorest	88.5 (76.1 to 94.9)	98.3 (90.5 to 99.7)	99.4 (88.9 to 100.0)	99.1	97.0
		Poorer	89.4 (78.5 to 95.0)	98.7 (92.6 to 99.8)	99.5 (91.9 to 100.0)	99.5	98.2
		Middle	90.6 (80.6 to 95.6)	98.7 (93.0 to 99.8)	99.5 (91.9 to 100.0)	99.5	98.2
		Richer	92.0 (82.4 to 96.5)	98.8 (93.4 to 99.8)	99.5 (92.0 to 100.0)	99.5	98.2
		Richest	94.8 (86.9 to 98.0)	98.9 (93.5 to 99.8)	99.5 (90.9 to 100.0)	99.4	97.8
	Ethiopia	Poorest	12.6 (11.3 to 14.1)	53.4 (50.8 to 55.9)	76.3 (73.0 to 79.3)	0.7	0.0
		Poorer	16.0 (14.6 to 17.5)	63.9 (61.5 to 66.1)	84.3 (81.9 to 86.5)	100.0	0.0
		Middle	21.0 (19.4 to 22.7)	69.1 (66.9 to 71.2)	86.7 (84.5 to 88.6)	100.0	0.0
		Richer	27.9 (25.9 to 30.0)	74.5 (72.3 to 76.5)	88.9 (86.9 to 90.7)	100.0	11.5
		Richest	42.5 (39.9 to 45.3)	79.2 (77.1 to 81.3)	89.6 (87.6 to 91.4)	100.0	35.5
	Gabon	Poorest	26.9 (23.4 to 30.7)	87.5 (84.6 to 89.9)	96.8 (95.4 to 97.8)	100.0	100.0
		Poorer	32.3 (29.0 to 35.7)	91.4 (89.2 to 93.1)	98.0 (97.1 to 98.7)	100.0	100.0
		Middle	39.4 (35.9 to 43.1)	92.9 (90.9 to 94.5)	98.3 (97.5 to 98.9)	100.0	100.0
		Richer	48.2 (43.6 to 52.7)	94.4 (92.4 to 95.9)	98.6 (97.8 to 99.1)	100.0	100.0
		Richest	63.6 (57.9 to 68.8)	95.6 (93.7 to 96.9)	98.7 (97.8 to 99.2)	100.0	100.0
	Gambia	Poorest	73.8 (60.3 to 83.9)	93.8 (92.1 to 95.1)	97.2 (95.1 to 98.4)	100.0	100.0
		Poorer	71.9 (58.4 to 82.3)	94.1 (92.7 to 95.2)	97.6 (95.7 to 98.6)	100.0	100.0
		Middle	71.1 (57.5 to 81.7)	93.3 (91.8 to 94.6)	97.1 (94.9 to 98.3)	100.0	100.0
		Richer	71.1 (57.4 to 81.8)	92.6 (90.7 to 94.2)	96.6 (94.0 to 98.1)	100.0	100.0
		Richest	76.4 (63.4 to 85.9)	91.8 (89.2 to 93.9)	95.5 (91.9 to 97.5)	100.0	99.6
	Ghana	Poorest	69.4 (63.9 to 74.4)	90.3 (87.1 to 92.7)	94.9 (91.7 to 97.0)	100.0	99.6
		Poorer	72.9 (68.2 to 77.2)	92.8 (90.5 to 94.6)	96.6 (94.3 to 97.9)	100.0	100.0
		Middle	77.0 (72.6 to 80.9)	93.6 (91.4 to 95.2)	96.8 (94.7 to 98.1)	100.0	100.0
		Richer	81.4 (77.0 to 85.1)	94.4 (92.4 to 96.0)	97.1 (95.1 to 98.3)	100.0	100.0
		Richest	88.2 (84.4 to 91.2)	95.2 (93.1 to 96.7)	97.0 (94.7 to 98.3)	100.0	100.0
	Guinea	Poorest	44.5 (40.0 to 49.2)	28.3 (25.3 to 31.5)	21.7 (17.5 to 26.4)	0.0	0.0
		Poorer	50.1 (45.8 to 54.4)	36.6 (33.6 to 39.7)	30.4 (25.4 to 35.9)	0.0	0.0
		Middle	57.0 (52.8 to 61.2)	40.9 (37.9 to 44.0)	33.4 (28.1 to 39.0)	0.0	0.0

Indicators	Country	Wealth Quintile	Estimates and Projections of Immunization Coverage (%)			Probability Achieving Targets (%)	
			Year 2000	Year 2020	Year 2030	UHC2030	IA2030
		Richer	64.7 (60.4 to 68.7)	46.1 (42.7 to 49.5)	36.9 (31.2 to 42.9)	0.0	0.0
		Richest	76.9 (72.9 to 80.4)	51.5 (47.4 to 55.5)	37.5 (31.4 to 44.0)	0.0	0.0
	Kenya	Poorest	56.1 (51.5 to 60.7)	92.0 (90.5 to 93.2)	97.2 (96.1 to 97.9)	100.0	100.0
		Poorer	60.9 (56.7 to 64.9)	94.2 (93.2 to 95.1)	98.1 (97.4 to 98.6)	100.0	100.0
		Middle	66.7 (62.8 to 70.3)	95.0 (94.0 to 95.8)	98.3 (97.7 to 98.8)	100.0	100.0
		Richer	72.8 (69.1 to 76.3)	95.8 (94.9 to 96.6)	98.5 (97.9 to 99.0)	100.0	100.0
		Richest	82.5 (79.3 to 85.4)	96.5 (95.6 to 97.2)	98.5 (97.8 to 99.0)	100.0	100.0
	Lesotho	Poorest	75.0 (69.0 to 80.2)	82.9 (77.2 to 87.5)	86.1 (76.8 to 92.1)	91.4	12.0
		Poorer	77.2 (72.0 to 81.7)	86.5 (82.0 to 90.1)	89.8 (82.7 to 94.3)	99.4	47.8
		Middle	80.2 (75.4 to 84.2)	87.4 (83.2 to 90.8)	90.1 (83.1 to 94.4)	99.6	51.9
		Richer	83.4 (78.8 to 87.2)	88.6 (84.3 to 91.8)	90.6 (83.7 to 94.8)	99.7	58.4
		Richest	89.2 (85.4 to 92.1)	89.7 (85.3 to 92.9)	89.9 (82.2 to 94.5)	99.2	48.9
	Liberia	Poorest	27.6 (23.1 to 32.6)	66.6 (63.3 to 69.7)	82.0 (77.4 to 85.8)	81.4	0.0
		Poorer	33.1 (28.4 to 38.1)	75.1 (72.4 to 77.6)	88.2 (84.8 to 90.9)	100.0	9.9
		Middle	40.4 (35.3 to 45.6)	79.0 (76.3 to 81.4)	89.8 (86.7 to 92.3)	100.0	45.1
		Richer	49.1 (43.5 to 54.7)	82.7 (79.9 to 85.2)	91.4 (88.5 to 93.7)	100.0	84.6
		Richest	64.5 (58.5 to 70.0)	86.0 (83.0 to 88.6)	91.9 (88.7 to 94.2)	100.0	89.3
	Madagascar	Poorest	32.0 (26.6 to 37.9)	80.3 (72.0 to 86.6)	92.3 (84.4 to 96.4)	99.7	75.9
		Poorer	44.3 (38.5 to 50.3)	88.9 (83.5 to 92.8)	96.2 (92.0 to 98.3)	100.0	99.5
		Middle	58.7 (53.0 to 64.1)	92.9 (89.0 to 95.5)	97.5 (94.6 to 98.9)	100.0	100.0
		Richer	72.6 (67.6 to 77.0)	95.6 (93.0 to 97.3)	98.4 (96.4 to 99.3)	100.0	100.0
		Richest	86.6 (83.3 to 89.4)	97.3 (95.6 to 98.4)	98.9 (97.3 to 99.5)	100.0	100.0
	Malawi	Poorest	77.4 (75.2 to 79.5)	93.8 (92.7 to 94.7)	96.9 (96.0 to 97.6)	100.0	100.0
		Poorer	79.4 (77.6 to 81.1)	95.2 (94.4 to 95.9)	97.8 (97.2 to 98.3)	100.0	100.0
		Middle	82.0 (80.5 to 83.5)	95.5 (94.8 to 96.1)	97.9 (97.2 to 98.4)	100.0	100.0
		Richer	85.0 (83.4 to 86.4)	95.9 (95.2 to 96.6)	98.0 (97.3 to 98.5)	100.0	100.0
		Richest	90.2 (88.8 to 91.4)	96.3 (95.5 to 97.0)	97.8 (97.0 to 98.4)	100.0	100.0
	Mali	Poorest	32.6 (30.3 to 35.0)	72.8 (70.2 to 75.2)	86.3 (83.7 to 88.5)	100.0	0.0
		Poorer	37.0 (34.8 to 39.2)	79.1 (77.2 to 80.9)	90.6 (88.8 to 92.1)	100.0	75.2
		Middle	42.9 (40.8 to 45.1)	81.5 (79.8 to 83.1)	91.4 (89.8 to 92.8)	100.0	95.9
		Richer	50.1 (47.7 to 52.5)	84.1 (82.3 to 85.6)	92.4 (90.8 to 93.7)	100.0	99.8
		Richest	63.8 (61.2 to 66.4)	86.4 (84.6 to 88.0)	92.3 (90.6 to 93.8)	100.0	99.4
	Morocco	Poorest	87.8 (81.5 to 92.2)	97.9 (84.3 to 99.8)	99.2 (79.8 to 100.0)	97.4	93.2
		Poorer	91.4 (87.1 to 94.4)	98.8 (90.4 to 99.9)	99.5 (88.2 to 100.0)	98.9	96.7
		Middle	94.3 (91.0 to 96.5)	99.1 (93.0 to 99.9)	99.7 (90.9 to 100.0)	99.2	97.7
		Richer	96.4 (93.7 to 98.0)	99.4 (95.0 to 99.9)	99.8 (93.2 to 100.0)	99.5	98.4
		Richest	98.3 (96.5 to 99.2)	99.6 (96.4 to 100.0)	99.8 (94.2 to 100.0)	99.6	98.7
	Mozambique	Poorest	53.5 (49.2 to 57.8)	65.0 (58.6 to 70.9)	70.2 (60.0 to 78.7)	1.0	0.0
		Poorer	63.4 (59.6 to 67.0)	76.5 (71.6 to 80.8)	81.7 (74.2 to 87.4)	69.0	0.1
		Middle	73.3 (70.0 to 76.3)	82.4 (78.5 to 85.8)	86.0 (79.9 to 90.5)	97.3	4.3
		Richer	82.0 (79.1 to 84.5)	87.4 (84.2 to 90.1)	89.6 (84.7 to 93.1)	100.0	42.2
		Richest	90.8 (88.9 to 92.5)	91.2 (88.6 to 93.3)	91.4 (87.0 to 94.4)	100.0	75.6
	Namibia	Poorest	77.0 (73.2 to 80.4)	84.4 (80.0 to 88.0)	87.3 (81.1 to 91.7)	98.8	13.2
		Poorer	77.6 (74.5 to 80.4)	86.7 (83.2 to 89.6)	90.0 (85.0 to 93.4)	100.0	49.4
		Middle	79.0 (76.2 to 81.5)	86.6 (83.1 to 89.5)	89.4 (84.3 to 93.0)	99.9	39.5
		Richer	81.1 (78.1 to 83.7)	86.8 (83.1 to 89.8)	89.1 (83.6 to 92.9)	99.9	33.8
		Richest	86.5 (83.6 to 88.9)	87.0 (82.9 to 90.3)	87.3 (80.8 to 91.9)	98.4	14.4
	Niger	Poorest	9.4 (7.5 to 11.9)	85.3 (81.6 to 88.4)	97.7 (96.4 to 98.6)	100.0	100.0
		Poorer	11.9 (9.6 to 14.6)	89.8 (87.1 to 91.9)	98.6 (97.8 to 99.1)	100.0	100.0
		Middle	15.5 (12.8 to 18.7)	91.6 (89.4 to 93.4)	98.8 (98.1 to 99.3)	100.0	100.0
		Richer	20.7 (17.3 to 24.7)	93.3 (91.4 to 94.8)	99.0 (98.4 to 99.4)	100.0	100.0
		Richest	32.9 (28.0 to 38.1)	94.7 (93.0 to 95.9)	99.1 (98.5 to 99.4)	100.0	100.0
	Nigeria	Poorest	4.2 (3.6 to 4.8)	21.1 (19.7 to 22.7)	40.0 (36.2 to 44.0)	0.0	0.0
		Poorer	8.3 (7.4 to 9.3)	39.6 (37.8 to 41.5)	63.8 (60.1 to 67.3)	0.0	0.0
		Middle	16.7 (15.1 to 18.4)	56.8 (54.9 to 58.7)	77.1 (74.2 to 79.8)	1.6	0.0
		Richer	31.6 (29.1 to 34.2)	73.1 (71.4 to 74.7)	86.8 (84.8 to 88.6)	100.0	0.0
		Richest	58.4 (55.2 to 61.5)	84.9 (83.5 to 86.2)	91.9 (90.3 to 93.2)	100.0	99.1
	Rwanda	Poorest	79.2 (76.5 to 81.7)	96.7 (95.8 to 97.4)	98.8 (98.2 to 99.2)	100.0	100.0
		Poorer	78.6 (76.2 to 80.8)	97.1 (96.3 to 97.7)	99.0 (98.5 to 99.3)	100.0	100.0
		Middle	78.9 (76.7 to 81.0)	96.8 (96.0 to 97.5)	98.9 (98.3 to 99.2)	100.0	100.0
		Richer	79.9 (77.5 to 82.1)	96.7 (95.8 to 97.4)	98.7 (98.1 to 99.2)	100.0	100.0

Indicators	Country	Wealth Quintile	Estimates and Projections of Immunization Coverage (%)			Probability Achieving Targets (%)	
			Year 2000	Year 2020	Year 2030	UHC2030	IA2030
		Richest	84.7 (82.4 to 86.8)	96.5 (95.5 to 97.3)	98.4 (97.6 to 99.0)	100.0	100.0
	Sao Tome and Principe	Poorest	70.3 (46.3 to 86.5)	94.2 (78.5 to 98.7)	97.7 (75.2 to 99.8)	96.1	87.7
		Poorer	72.6 (49.6 to 87.5)	95.5 (83.2 to 99.0)	98.4 (81.5 to 99.9)	97.9	92.4
		Middle	75.8 (53.4 to 89.3)	95.8 (84.3 to 99.0)	98.4 (82.0 to 99.9)	98.0	92.7
		Richer	79.4 (57.3 to 91.6)	96.2 (85.3 to 99.1)	98.5 (82.5 to 99.9)	98.1	93.1
		Richest	86.2 (66.8 to 95.0)	96.5 (86.1 to 99.2)	98.3 (80.9 to 99.9)	97.7	92.0
	Senegal	Poorest	57.5 (53.4 to 61.4)	88.8 (87.5 to 90.0)	95.1 (93.8 to 96.1)	100.0	100.0
		Poorer	63.7 (60.1 to 67.1)	92.3 (91.4 to 93.2)	96.9 (96.1 to 97.6)	100.0	100.0
		Middle	70.6 (67.3 to 73.7)	93.7 (92.9 to 94.5)	97.4 (96.7 to 98.0)	100.0	100.0
		Richer	77.4 (74.3 to 80.2)	95.0 (94.2 to 95.7)	97.8 (97.2 to 98.3)	100.0	100.0
		Richest	86.5 (84.1 to 88.7)	96.1 (95.3 to 96.7)	98.0 (97.3 to 98.5)	100.0	100.0
	Sierra Leone	Poorest	34.3 (28.8 to 40.2)	88.2 (86.4 to 89.8)	96.6 (95.4 to 97.5)	100.0	100.0
		Poorer	34.2 (29.0 to 39.7)	89.7 (88.2 to 91.0)	97.3 (96.3 to 98.0)	100.0	100.0
		Middle	35.2 (30.1 to 40.7)	89.2 (87.7 to 90.5)	97.0 (96.0 to 97.8)	100.0	100.0
		Richer	37.3 (31.9 to 43.0)	89.0 (87.3 to 90.5)	96.8 (95.6 to 97.6)	100.0	100.0
		Richest	46.1 (39.8 to 52.5)	88.8 (86.8 to 90.6)	96.0 (94.5 to 97.1)	100.0	100.0
	South Africa	Poorest	41.9 (12.4 to 79.1)	69.7 (58.4 to 78.9)	80.4 (48.2 to 94.6)	51.3	14.3
		Poorer	40.4 (11.8 to 78.0)	71.6 (61.5 to 79.9)	83.0 (52.8 to 95.4)	60.6	19.9
		Middle	40.2 (11.6 to 78.0)	69.5 (59.2 to 78.0)	80.7 (49.2 to 94.6)	52.4	14.5
		Richer	41.0 (11.8 to 78.8)	67.8 (56.8 to 77.0)	78.6 (45.9 to 93.9)	45.2	10.8
		Richest	48.6 (15.1 to 83.8)	66.2 (53.9 to 76.6)	73.8 (39.2 to 92.3)	31.6	5.7
	Tanzania	Poorest	77.5 (73.5 to 81.1)	82.3 (78.7 to 85.3)	84.3 (78.3 to 88.9)	92.7	0.5
		Poorer	83.3 (80.2 to 86.0)	88.7 (86.4 to 90.6)	90.7 (86.9 to 93.5)	100.0	66.5
		Middle	88.3 (85.9 to 90.4)	91.5 (89.7 to 93.1)	92.8 (89.7 to 95.0)	100.0	96.4
		Richer	92.3 (90.4 to 93.9)	93.9 (92.4 to 95.1)	94.6 (92.0 to 96.3)	100.0	99.9
		Richest	96.2 (95.0 to 97.1)	95.6 (94.3 to 96.7)	95.4 (93.0 to 96.9)	100.0	100.0
	Togo	Poorest	51.4 (21.3 to 80.1)	88.6 (78.3 to 94.4)	95.4 (78.0 to 99.2)	96.7	82.6
		Poorer	53.2 (22.6 to 81.2)	90.7 (82.2 to 95.4)	96.6 (83.0 to 99.4)	98.5	90.1
		Middle	56.3 (24.8 to 83.1)	90.9 (82.7 to 95.5)	96.5 (82.8 to 99.4)	98.5	89.8
		Richer	60.4 (27.8 to 85.5)	91.4 (83.4 to 95.8)	96.5 (82.8 to 99.4)	98.5	89.8
		Richest	70.3 (37.0 to 90.4)	91.9 (84.0 to 96.1)	96.1 (80.8 to 99.3)	97.8	87.0
	Uganda	Poorest	44.3 (41.2 to 47.4)	84.2 (82.3 to 85.8)	93.2 (91.7 to 94.5)	100.0	100.0
		Poorer	44.0 (41.3 to 46.7)	86.0 (84.5 to 87.4)	94.5 (93.3 to 95.5)	100.0	100.0
		Middle	45.0 (42.4 to 47.6)	85.3 (83.7 to 86.7)	93.9 (92.6 to 95.0)	100.0	100.0
		Richer	47.0 (44.2 to 49.9)	84.9 (83.2 to 86.5)	93.4 (91.9 to 94.7)	100.0	100.0
		Richest	55.9 (52.8 to 59.1)	84.6 (82.6 to 86.5)	92.0 (90.0 to 93.6)	100.0	97.7
	Zambia	Poorest	66.5 (63.1 to 69.7)	86.6 (84.8 to 88.3)	92.2 (90.2 to 93.7)	100.0	98.4
		Poorer	71.5 (68.8 to 74.1)	90.6 (89.4 to 91.7)	95.0 (93.7 to 96.0)	100.0	100.0
		Middle	77.1 (74.6 to 79.4)	92.1 (91.0 to 93.1)	95.6 (94.5 to 96.5)	100.0	100.0
		Richer	82.5 (80.1 to 84.6)	93.6 (92.5 to 94.5)	96.3 (95.2 to 97.1)	100.0	100.0
		Richest	89.6 (87.7 to 91.3)	94.8 (93.7 to 95.8)	96.4 (95.2 to 97.3)	100.0	100.0
	Zimbabwe	Poorest	44.4 (38.7 to 50.2)	89.3 (86.5 to 91.6)	96.5 (94.5 to 97.7)	100.0	100.0
		Poorer	46.5 (41.2 to 51.9)	91.4 (89.3 to 93.2)	97.4 (96.0 to 98.3)	100.0	100.0
		Middle	50.0 (44.6 to 55.3)	91.8 (89.7 to 93.4)	97.4 (96.0 to 98.3)	100.0	100.0
		Richer	54.5 (48.8 to 60.1)	92.3 (90.3 to 93.9)	97.4 (96.0 to 98.4)	100.0	100.0
		Richest	65.5 (59.4 to 71.1)	92.8 (90.8 to 94.5)	97.1 (95.5 to 98.2)	100.0	100.0

Notes: Countries are listed in alphabetical order. UHC = Probability of achieving the Universal Health Coverage (UHC) benchmark (≥80% coverage) by 2030. IA = Probability of achieving the Immunization Agenda 2030 (IA2030) target (≥90% coverage) by 2030.

Table H. Immunization coverage 2000–2030, and % reach global targets, by educational level

Indicators	Country	Educational Level	Estimates and Projections of Immunization Coverage (%)			Probability Achieving Targets (%)	
			Year 2000	Year 2020	Year 2030	UHC2030	IA2030
Full immunization	Angola	Primary or lower	28.3 (8.4 to 63.6)	20.9 (13.7 to 30.4)	17.8 (4.6 to 48.4)	0.0	0.0
		Secondary or higher	69.7 (34.7 to 91.2)	48.4 (36.0 to 60.7)	37.4 (11.8 to 72.0)	0.5	0.0
	Benin	Primary or lower	51.4 (49.1 to 53.6)	61.5 (59.2 to 63.7)	66.2 (62.3 to 69.9)	0.0	0.0
		Secondary or higher	79.3 (76.0 to 82.1)	77.8 (74.8 to 80.5)	77.0 (72.7 to 80.8)	6.5	0.0
	Burkina Faso	Primary or lower	28.0 (25.3 to 30.9)	98.3 (97.8 to 98.7)	99.9 (99.8 to 99.9)	100.0	100.0
		Secondary or higher	52.6 (43.9 to 61.2)	99.0 (98.5 to 99.4)	99.9 (99.8 to 99.9)	100.0	100.0
	Burundi	Primary or lower	90.9 (86.2 to 94.1)	88.4 (85.9 to 90.6)	87.0 (79.5 to 92.0)	96.8	14.1
		Secondary or higher	94.8 (91.2 to 97.0)	89.5 (85.8 to 92.3)	85.3 (76.2 to 91.3)	89.1	7.2
	Cameroon	Primary or lower	44.7 (41.6 to 48.0)	49.4 (46.5 to 52.2)	51.7 (46.5 to 56.9)	0.0	0.0
		Secondary or higher	76.2 (73.0 to 79.1)	70.0 (67.3 to 72.6)	66.6 (61.7 to 71.2)	0.0	0.0
	Chad	Primary or lower	8.6 (6.7 to 11.0)	35.8 (31.8 to 39.9)	57.5 (48.3 to 66.1)	0.0	0.0
		Secondary or higher	27.7 (21.8 to 34.6)	57.8 (51.3 to 64.0)	72.1 (62.8 to 79.9)	2.3	0.0
	Comoros	Primary or lower	44.3 (18.2 to 73.8)	72.4 (52.2 to 86.4)	82.7 (41.2 to 97.0)	57.2	25.4
		Secondary or higher	63.2 (31.6 to 86.3)	77.5 (58.9 to 89.2)	83.0 (42.0 to 97.1)	58.1	26.0
	Congo Brazzaville	Primary or lower	48.3 (41.8 to 54.9)	31.5 (25.3 to 38.5)	24.4 (15.5 to 36.2)	0.0	0.0
		Secondary or higher	71.1 (65.4 to 76.2)	42.3 (35.0 to 50.0)	28.6 (18.5 to 41.4)	0.0	0.0
	Congo Democratic Republic	Primary or lower	16.8 (13.7 to 20.3)	63.8 (59.3 to 68.1)	83.9 (78.0 to 88.5)	91.1	0.3
		Secondary or higher	36.0 (30.6 to 41.8)	74.9 (70.9 to 78.6)	87.3 (82.2 to 91.1)	99.6	9.4
	Cote d'Ivoire	Primary or lower	40.1 (18.0 to 67.3)	58.9 (36.1 to 78.3)	67.7 (23.0 to 93.5)	25.2	6.8
		Secondary or higher	74.4 (46.9 to 90.6)	79.0 (59.1 to 90.7)	81.1 (38.0 to 96.7)	52.8	22.2
	Egypt	Primary or lower	90.5 (89.4 to 91.5)	80.4 (77.2 to 83.3)	72.9 (66.4 to 78.6)	0.6	0.0
		Secondary or higher	94.2 (93.5 to 95.0)	81.1 (78.6 to 83.3)	68.7 (62.7 to 74.1)	0.0	0.0
	Eswatini	Primary or lower	78.8 (63.4 to 88.7)	93.5 (73.0 to 98.7)	96.6 (62.9 to 99.8)	91.6	79.0
		Secondary or higher	89.6 (79.3 to 95.0)	95.3 (79.4 to 99.1)	96.9 (65.3 to 99.8)	92.6	80.9
	Ethiopia	Primary or lower	12.8 (11.7 to 14.0)	45.8 (43.7 to 48.0)	67.0 (63.4 to 70.3)	0.0	0.0
		Secondary or higher	45.9 (41.7 to 50.3)	74.8 (71.5 to 77.8)	84.7 (81.5 to 87.4)	99.7	0.0
	Gabon	Primary or lower	15.2 (12.8 to 17.9)	51.4 (45.0 to 57.8)	72.0 (62.9 to 79.6)	1.8	0.0
		Secondary or higher	19.8 (16.8 to 23.1)	46.9 (40.9 to 53.0)	62.6 (52.8 to 71.4)	0.0	0.0
	Gambia	Primary or lower	75.8 (65.6 to 83.8)	87.3 (85.2 to 89.2)	91.1 (86.5 to 94.2)	100.0	70.6
		Secondary or higher	82.0 (72.6 to 88.7)	85.9 (83.0 to 88.4)	87.6 (81.5 to 91.9)	99.1	15.3
	Ghana	Primary or lower	64.1 (59.2 to 68.6)	83.1 (79.1 to 86.5)	89.1 (83.9 to 92.8)	99.9	33.8
		Secondary or higher	80.2 (75.9 to 83.9)	87.2 (83.8 to 89.9)	89.8 (84.8 to 93.2)	100.0	45.6
	Guinea	Primary or lower	48.4 (44.4 to 52.5)	25.8 (23.4 to 28.3)	17.4 (14.2 to 21.3)	0.0	0.0
		Secondary or higher	75.7 (70.3 to 80.4)	41.1 (36.2 to 46.2)	24.8 (19.8 to 30.7)	0.0	0.0
	Kenya	Primary or lower	50.0 (46.3 to 53.6)	84.7 (82.8 to 86.4)	92.9 (91.0 to 94.4)	100.0	99.8
		Secondary or higher	73.9 (69.9 to 77.5)	90.5 (88.8 to 92.0)	94.6 (92.8 to 95.9)	100.0	100.0
	Lesotho	Primary or lower	63.1 (57.7 to 68.3)	71.9 (65.5 to 77.6)	75.8 (64.9 to 84.1)	18.0	0.0
		Secondary or higher	75.8 (70.4 to 80.5)	74.0 (68.1 to 79.1)	73.0 (62.0 to 81.8)	6.4	0.0
	Liberia	Primary or lower	37.0 (32.5 to 41.9)	58.8 (55.7 to 61.9)	69.0 (63.2 to 74.3)	0.0	0.0
		Secondary or higher	58.4 (52.1 to 64.3)	67.4 (63.5 to 71.0)	71.5 (65.3 to 77.0)	0.1	0.0
	Madagascar	Primary or lower	40.2 (35.3 to 45.4)	80.8 (73.6 to 86.5)	91.3 (83.8 to 95.6)	99.6	66.8
		Secondary or higher	74.6 (69.7 to 79.0)	91.8 (87.5 to 94.7)	95.6 (91.0 to 97.9)	100.0	98.8
	Malawi	Primary or lower	69.3 (67.7 to 70.9)	88.0 (86.8 to 89.2)	93.0 (91.7 to 94.1)	100.0	100.0
		Secondary or higher	86.9 (84.6 to 88.8)	92.9 (91.5 to 94.0)	94.8 (93.4 to 96.0)	100.0	100.0
	Mali	Primary or lower	35.5 (33.8 to 37.2)	63.8 (61.7 to 66.0)	76.0 (73.0 to 78.8)	0.2	0.0
		Secondary or higher	65.5 (61.3 to 69.5)	78.8 (75.7 to 81.5)	83.8 (80.5 to 86.7)	98.7	0.0
	Morocco	Primary or lower	85.0 (79.1 to 89.4)	95.6 (74.0 to 99.4)	97.7 (63.1 to 99.9)	92.7	83.0
		Secondary or higher	95.2 (91.2 to 97.4)	97.8 (85.2 to 99.7)	98.6 (73.3 to 99.9)	95.9	89.4
	Mozambique	Primary or lower	62.0 (58.8 to 65.1)	70.1 (65.2 to 74.6)	73.8 (65.6 to 80.5)	3.6	0.0
		Secondary or higher	87.2 (83.5 to 90.1)	85.5 (81.2 to 89.0)	84.7 (78.1 to 89.5)	92.5	1.4
	Namibia	Primary or lower	62.9 (59.4 to 66.3)	79.0 (74.1 to 83.1)	84.8 (78.5 to 89.5)	93.7	1.4
		Secondary or higher	74.4 (71.1 to 77.4)	79.6 (75.4 to 83.2)	81.9 (75.3 to 87.0)	72.8	0.0
	Niger	Primary or lower	14.0 (11.5 to 16.8)	80.0 (75.8 to 83.5)	95.2 (92.6 to 96.9)	100.0	100.0
		Secondary or higher	39.2 (31.7 to 47.3)	90.6 (87.2 to 93.2)	97.4 (95.6 to 98.4)	100.0	100.0
	Nigeria	Primary or lower	8.3 (7.4 to 9.3)	25.7 (24.3 to 27.1)	40.3 (36.8 to 43.8)	0.0	0.0
		Secondary or higher	46.7 (43.7 to 49.8)	67.0 (65.3 to 68.6)	75.5 (72.6 to 78.1)	0.0	0.0
	Rwanda	Primary or lower	66.3 (64.0 to 68.5)	91.2 (89.7 to 92.5)	95.9 (94.6 to 96.9)	100.0	100.0
		Secondary or higher	78.2 (74.6 to 81.5)	92.0 (89.9 to 93.7)	95.3 (93.5 to 96.7)	100.0	100.0

Indicators	Country	Educational Level	Estimates and Projections of Immunization Coverage (%)			Probability Achieving Targets (%)	
			Year 2000	Year 2020	Year 2030	UHC2030	IA2030
	Sao Tome and Principe	Primary or lower	64.8 (41.7 to 82.2)	89.3 (67.9 to 97.2)	94.7 (59.9 to 99.6)	88.3	70.7
		Secondary or higher	74.2 (49.7 to 89.0)	88.8 (66.0 to 97.1)	93.0 (52.3 to 99.4)	82.9	61.9
	Senegal	Primary or lower	52.4 (49.3 to 55.4)	82.9 (81.5 to 84.2)	91.1 (89.4 to 92.5)	100.0	90.2
		Secondary or higher	83.4 (79.9 to 86.4)	93.1 (91.7 to 94.3)	95.7 (94.4 to 96.6)	100.0	100.0
	Sierra Leone	Primary or lower	42.9 (38.0 to 47.9)	68.9 (66.4 to 71.2)	79.1 (74.9 to 82.8)	33.1	0.0
		Secondary or higher	61.2 (54.8 to 67.2)	73.8 (70.8 to 76.5)	79.0 (74.5 to 82.9)	31.7	0.0
	South Africa	Primary or lower	35.2 (10.0 to 73.4)	50.0 (34.9 to 65.0)	57.6 (22.4 to 86.1)	8.0	0.7
		Secondary or higher	57.2 (21.1 to 87.4)	59.9 (49.1 to 69.7)	61.3 (27.3 to 86.6)	9.6	0.8
	Tanzania	Primary or lower	71.8 (68.6 to 74.8)	80.4 (77.8 to 82.9)	83.9 (79.5 to 87.6)	96.1	0.0
		Secondary or higher	87.9 (84.7 to 90.5)	87.7 (84.8 to 90.1)	87.6 (83.3 to 90.9)	99.9	8.2
	Togo	Primary or lower	46.6 (19.4 to 75.8)	75.3 (60.0 to 86.1)	85.0 (51.3 to 96.9)	66.0	29.3
		Secondary or higher	72.8 (41.4 to 91.0)	85.0 (73.3 to 92.2)	89.2 (60.5 to 97.8)	80.3	45.7
	Uganda	Primary or lower	36.8 (34.6 to 39.0)	69.7 (67.5 to 71.8)	82.0 (79.3 to 84.5)	92.8	0.0
		Secondary or higher	56.3 (52.6 to 59.9)	75.5 (72.9 to 78.0)	82.7 (79.5 to 85.5)	95.1	0.0
	Zambia	Primary or lower	63.8 (61.2 to 66.3)	78.2 (76.3 to 80.0)	83.7 (80.9 to 86.1)	99.4	0.0
		Secondary or higher	82.5 (79.9 to 84.7)	85.3 (83.5 to 87.0)	86.6 (83.9 to 88.9)	100.0	0.1
	Zimbabwe	Primary or lower	31.7 (27.2 to 36.6)	81.4 (77.6 to 84.7)	93.1 (89.9 to 95.3)	100.0	97.3
		Secondary or higher	49.7 (44.6 to 54.8)	84.9 (82.0 to 87.4)	93.1 (90.2 to 95.2)	100.0	98.0
BCG immunization	Angola	Primary or lower	61.5 (25.5 to 88.5)	66.0 (53.1 to 76.6)	68.1 (30.5 to 91.0)	21.2	3.4
		Secondary or higher	87.2 (58.8 to 97.1)	85.7 (77.5 to 91.2)	84.9 (53.5 to 96.4)	66.4	27.3
	Benin	Primary or lower	88.4 (86.9 to 89.7)	85.7 (83.9 to 87.4)	84.2 (80.5 to 87.3)	98.7	0.0
		Secondary or higher	98.1 (97.2 to 98.7)	96.7 (95.3 to 97.7)	95.7 (93.4 to 97.2)	100.0	100.0
	Burkina Faso	Primary or lower	66.7 (62.4 to 70.6)	99.7 (99.4 to 99.8)	100.0 (99.9 to 100.0)	100.0	100.0
		Secondary or higher	90.9 (83.7 to 95.2)	99.9 (99.8 to 100.0)	100.0 (100.0 to 100.0)	100.0	100.0
	Burundi	Primary or lower	98.8 (97.1 to 99.6)	97.4 (95.9 to 98.3)	96.1 (89.1 to 98.6)	99.9	96.3
		Secondary or higher	99.6 (98.7 to 99.9)	98.8 (97.4 to 99.4)	97.8 (93.0 to 99.3)	100.0	99.5
	Cameroon	Primary or lower	82.5 (79.8 to 85.0)	80.6 (78.0 to 82.9)	79.5 (74.4 to 83.9)	42.3	0.0
		Secondary or higher	96.6 (95.4 to 97.4)	94.7 (93.4 to 95.7)	93.4 (90.8 to 95.3)	100.0	99.4
	Chad	Primary or lower	32.0 (28.0 to 36.3)	66.9 (63.6 to 70.0)	80.7 (75.8 to 84.8)	61.7	0.0
		Secondary or higher	68.2 (60.9 to 74.7)	86.9 (83.2 to 89.9)	92.1 (88.5 to 94.7)	100.0	88.7
	Comoros	Primary or lower	68.8 (35.7 to 89.8)	89.6 (76.6 to 95.8)	94.5 (67.7 to 99.3)	91.5	73.0
		Secondary or higher	82.1 (51.9 to 95.2)	92.9 (83.0 to 97.2)	95.6 (73.1 to 99.4)	94.6	80.2
	Congo Brazzaville	Primary or lower	78.0 (70.1 to 84.3)	94.9 (91.6 to 96.9)	97.7 (94.2 to 99.1)	100.0	99.9
		Secondary or higher	93.2 (89.7 to 95.6)	98.1 (96.7 to 98.9)	99.0 (97.4 to 99.6)	100.0	100.0
	Congo Democratic Republic	Primary or lower	45.0 (38.6 to 51.4)	88.8 (86.3 to 90.9)	96.1 (94.0 to 97.5)	100.0	100.0
		Secondary or higher	74.6 (68.5 to 79.8)	95.4 (94.0 to 96.4)	98.2 (97.1 to 98.9)	100.0	100.0
	Cote d'Ivoire	Primary or lower	70.0 (41.0 to 88.8)	88.8 (74.1 to 95.6)	93.6 (63.6 to 99.2)	88.7	67.5
		Secondary or higher	91.0 (71.5 to 97.6)	96.1 (89.2 to 98.7)	97.5 (82.1 to 99.7)	98.1	91.2
	Egypt	Primary or lower	98.4 (97.9 to 98.8)	98.4 (97.4 to 99.1)	98.4 (96.4 to 99.3)	100.0	100.0
		Secondary or higher	99.1 (98.8 to 99.4)	98.8 (98.1 to 99.2)	98.6 (97.0 to 99.3)	100.0	100.0
	Eswatini	Primary or lower	93.7 (85.7 to 97.4)	98.7 (92.1 to 99.8)	99.4 (88.7 to 100.0)	99.1	96.9
		Secondary or higher	97.1 (92.3 to 98.9)	99.2 (95.0 to 99.9)	99.6 (91.7 to 100.0)	99.5	98.1
	Ethiopia	Primary or lower	47.4 (45.5 to 49.3)	73.5 (71.7 to 75.2)	82.9 (80.6 to 85.0)	99.3	0.0
		Secondary or higher	84.7 (81.2 to 87.6)	92.5 (90.5 to 94.0)	94.8 (93.0 to 96.2)	100.0	100.0
	Gabon	Primary or lower	84.2 (81.0 to 87.0)	89.2 (84.9 to 92.3)	91.1 (84.8 to 94.9)	99.9	66.1
		Secondary or higher	93.3 (91.2 to 95.0)	94.0 (91.3 to 95.9)	94.3 (90.0 to 96.8)	100.0	97.4
	Gambia	Primary or lower	97.1 (90.8 to 99.2)	98.9 (98.2 to 99.4)	99.4 (97.8 to 99.8)	100.0	100.0
		Secondary or higher	99.0 (95.8 to 99.8)	99.5 (98.9 to 99.7)	99.6 (98.7 to 99.9)	100.0	100.0
	Ghana	Primary or lower	85.8 (81.3 to 89.3)	97.5 (95.9 to 98.5)	99.0 (97.7 to 99.6)	100.0	100.0
		Secondary or higher	94.7 (91.8 to 96.6)	98.8 (97.9 to 99.3)	99.4 (98.7 to 99.8)	100.0	100.0
	Guinea	Primary or lower	83.0 (80.0 to 85.7)	71.7 (68.9 to 74.4)	64.7 (58.2 to 70.6)	0.0	0.0
		Secondary or higher	95.6 (93.5 to 97.1)	89.1 (85.4 to 92.0)	83.4 (76.6 to 88.5)	85.2	0.4
	Kenya	Primary or lower	82.3 (78.7 to 85.4)	98.0 (97.4 to 98.5)	99.4 (99.0 to 99.6)	100.0	100.0
		Secondary or higher	93.7 (91.0 to 95.7)	99.1 (98.7 to 99.4)	99.7 (99.4 to 99.8)	100.0	100.0
	Lesotho	Primary or lower	93.0 (89.1 to 95.5)	96.8 (93.9 to 98.4)	97.9 (93.3 to 99.3)	100.0	99.6

Indicators	Country	Educational Level	Estimates and Projections of Immunization Coverage (%)			Probability Achieving Targets (%)	
			Year 2000	Year 2020	Year 2030	UHC2030	IA2030
		Secondary or higher	97.7 (95.7 to 98.8)	98.6 (97.2 to 99.3)	98.9 (96.5 to 99.7)	100.0	100.0
	Liberia	Primary or lower	63.0 (56.2 to 69.3)	93.0 (91.4 to 94.3)	97.4 (96.0 to 98.3)	100.0	100.0
		Secondary or higher	85.3 (79.2 to 89.8)	97.0 (95.8 to 97.9)	98.7 (97.9 to 99.2)	100.0	100.0
	Madagascar	Primary or lower	56.0 (50.1 to 61.7)	95.4 (92.6 to 97.1)	98.8 (97.3 to 99.5)	100.0	100.0
		Secondary or higher	90.8 (87.2 to 93.4)	99.1 (98.4 to 99.5)	99.7 (99.3 to 99.9)	100.0	100.0
	Malawi	Primary or lower	90.7 (89.6 to 91.7)	98.2 (97.7 to 98.5)	99.2 (98.8 to 99.5)	100.0	100.0
		Secondary or higher	97.7 (96.5 to 98.5)	99.4 (99.1 to 99.6)	99.7 (99.5 to 99.8)	100.0	100.0
	Mali	Primary or lower	68.7 (66.9 to 70.4)	84.7 (83.1 to 86.2)	89.8 (87.8 to 91.5)	100.0	42.2
		Secondary or higher	92.8 (90.3 to 94.7)	95.9 (94.5 to 97.0)	97.0 (95.6 to 97.9)	100.0	100.0
	Morocco	Primary or lower	97.5 (95.5 to 98.6)	99.5 (95.6 to 99.9)	99.8 (93.4 to 100.0)	99.5	98.5
		Secondary or higher	99.4 (98.4 to 99.8)	99.9 (98.5 to 100.0)	99.9 (97.4 to 100.0)	99.9	99.6
	Mozambique	Primary or lower	85.6 (83.1 to 87.9)	93.2 (90.7 to 95.1)	95.4 (92.1 to 97.4)	100.0	99.7
		Secondary or higher	94.3 (91.3 to 96.3)	96.5 (94.4 to 97.8)	97.3 (94.7 to 98.6)	100.0	100.0
	Namibia	Primary or lower	88.4 (85.7 to 90.6)	94.9 (92.0 to 96.8)	96.7 (93.2 to 98.4)	100.0	99.9
		Secondary or higher	94.1 (92.2 to 95.5)	96.6 (94.7 to 97.8)	97.4 (94.8 to 98.7)	100.0	100.0
	Niger	Primary or lower	36.8 (31.6 to 42.2)	95.3 (93.8 to 96.5)	99.2 (98.6 to 99.5)	100.0	100.0
		Secondary or higher	79.8 (70.2 to 86.9)	99.0 (98.3 to 99.4)	99.8 (99.6 to 99.9)	100.0	100.0
	Nigeria	Primary or lower	23.3 (21.5 to 25.3)	48.3 (46.6 to 50.0)	62.1 (58.8 to 65.3)	0.0	0.0
		Secondary or higher	80.7 (78.2 to 83.1)	90.3 (89.3 to 91.2)	93.3 (91.9 to 94.4)	100.0	100.0
	Rwanda	Primary or lower	95.2 (94.1 to 96.1)	98.5 (97.8 to 99.0)	99.2 (98.4 to 99.6)	100.0	100.0
		Secondary or higher	97.5 (96.0 to 98.5)	98.9 (98.1 to 99.4)	99.3 (98.4 to 99.7)	100.0	100.0
	Sao Tome and Principe	Primary or lower	91.1 (77.7 to 96.7)	98.1 (91.5 to 99.6)	99.1 (88.1 to 99.9)	99.1	96.6
		Secondary or higher	97.7 (91.0 to 99.4)	99.4 (96.5 to 99.9)	99.7 (94.7 to 100.0)	99.8	99.2
	Senegal	Primary or lower	90.2 (88.0 to 92.0)	95.8 (95.0 to 96.4)	97.3 (96.2 to 98.1)	100.0	100.0
		Secondary or higher	98.4 (97.3 to 99.1)	99.1 (98.5 to 99.4)	99.3 (98.8 to 99.6)	100.0	100.0
	Sierra Leone	Primary or lower	58.3 (49.6 to 66.6)	97.5 (96.8 to 98.1)	99.5 (99.2 to 99.7)	100.0	100.0
		Secondary or higher	77.5 (67.4 to 85.1)	98.6 (97.9 to 99.0)	99.7 (99.4 to 99.8)	100.0	100.0
	South Africa	Primary or lower	76.3 (36.0 to 94.9)	91.1 (81.9 to 95.9)	94.8 (75.7 to 99.1)	95.5	78.8
		Secondary or higher	86.1 (51.0 to 97.4)	93.5 (89.4 to 96.0)	95.6 (81.7 to 99.0)	98.2	86.6
	Tanzania	Primary or lower	88.6 (85.8 to 90.9)	97.4 (96.5 to 98.1)	98.8 (97.9 to 99.3)	100.0	100.0
		Secondary or higher	95.2 (92.2 to 97.0)	98.6 (97.7 to 99.1)	99.2 (98.5 to 99.6)	100.0	100.0
	Togo	Primary or lower	84.7 (57.1 to 95.8)	96.6 (92.7 to 98.5)	98.5 (91.0 to 99.8)	99.8	98.1
		Secondary or higher	96.8 (85.1 to 99.4)	99.1 (97.7 to 99.7)	99.5 (97.1 to 99.9)	100.0	99.9
	Uganda	Primary or lower	77.0 (74.6 to 79.2)	97.6 (97.1 to 98.1)	99.3 (99.0 to 99.5)	100.0	100.0
		Secondary or higher	88.3 (85.1 to 90.8)	98.5 (98.0 to 98.9)	99.5 (99.2 to 99.7)	100.0	100.0
	Zambia	Primary or lower	91.1 (89.4 to 92.5)	95.8 (94.8 to 96.6)	97.2 (95.9 to 98.0)	100.0	100.0
		Secondary or higher	96.4 (95.1 to 97.4)	97.8 (97.0 to 98.3)	98.2 (97.3 to 98.8)	100.0	100.0
	Zimbabwe	Primary or lower	59.1 (52.6 to 65.3)	94.0 (91.8 to 95.6)	98.1 (96.7 to 98.9)	100.0	100.0
		Secondary or higher	69.8 (64.1 to 74.9)	94.7 (93.1 to 96.0)	98.0 (96.7 to 98.8)	100.0	100.0
MCV1 immunization	Angola	Primary or lower	55.1 (23.4 to 83.0)	42.2 (31.2 to 54.0)	36.0 (12.3 to 69.5)	0.3	0.0
		Secondary or higher	88.3 (64.8 to 96.9)	74.8 (64.8 to 82.9)	65.1 (31.8 to 88.3)	14.0	1.4
	Benin	Primary or lower	61.2 (58.9 to 63.4)	66.2 (64.0 to 68.4)	68.7 (64.7 to 72.3)	0.0	0.0
		Secondary or higher	85.4 (82.7 to 87.8)	82.9 (80.1 to 85.3)	81.5 (77.4 to 84.9)	77.1	0.0
	Burkina Faso	Primary or lower	37.9 (34.7 to 41.3)	98.6 (98.1 to 98.9)	99.9 (99.8 to 99.9)	100.0	100.0
		Secondary or higher	68.5 (59.5 to 76.3)	99.4 (99.0 to 99.6)	99.9 (99.9 to 100.0)	100.0	100.0
	Burundi	Primary or lower	93.6 (89.3 to 96.3)	93.7 (91.8 to 95.2)	93.8 (88.4 to 96.7)	100.0	93.2
		Secondary or higher	97.5 (95.0 to 98.8)	96.3 (94.3 to 97.7)	95.6 (91.0 to 97.9)	100.0	98.8
	Cameroon	Primary or lower	61.3 (58.1 to 64.5)	58.0 (55.1 to 60.9)	56.3 (50.9 to 61.6)	0.0	0.0
		Secondary or higher	85.5 (83.1 to 87.6)	77.3 (74.9 to 79.7)	72.2 (67.4 to 76.6)	0.0	0.0
	Chad	Primary or lower	13.9 (11.5 to 16.7)	73.7 (70.7 to 76.5)	92.1 (89.5 to 94.1)	100.0	94.9
		Secondary or higher	36.0 (29.5 to 43.0)	86.6 (83.2 to 89.4)	95.6 (93.6 to 97.0)	100.0	100.0
	Comoros	Primary or lower	57.8 (27.0 to 83.4)	80.3 (62.3 to 91.0)	87.6 (49.5 to 98.1)	71.6	40.2
		Secondary or higher	73.2 (41.4 to 91.3)	84.4 (68.7 to 93.1)	88.4 (51.8 to 98.2)	74.3	43.2
	Congo Brazzaville	Primary or lower	45.5 (38.7 to 52.5)	77.3 (71.0 to 82.5)	87.3 (78.9 to 92.6)	96.0	18.9
		Secondary or higher	72.0 (66.0 to 77.3)	87.4 (83.1 to 90.8)	92.0 (86.0 to 95.5)	100.0	77.4
		Primary or lower	43.8 (38.1 to 49.7)	75.1 (71.1 to 78.7)	85.6 (79.9 to 89.9)	97.3	2.1

Indicators	Country	Educational Level	Estimates and Projections of Immunization Coverage (%)			Probability Achieving Targets (%)	
			Year 2000	Year 2020	Year 2030	UHC2030	IA2030
	Congo Democratic Republic	Secondary or higher	69.9 (64.2 to 75.1)	85.7 (82.7 to 88.2)	90.5 (86.3 to 93.6)	100.0	61.1
	Cote d'Ivoire	Primary or lower	54.9 (29.0 to 78.4)	67.6 (45.6 to 83.8)	73.2 (28.9 to 94.8)	34.3	10.6
		Secondary or higher	83.9 (61.3 to 94.6)	85.6 (70.0 to 93.8)	86.3 (48.9 to 97.7)	68.5	35.4
	Egypt	Primary or lower	97.3 (96.8 to 97.8)	76.7 (72.1 to 80.8)	50.0 (40.0 to 59.9)	0.0	0.0
		Secondary or higher	98.5 (98.2 to 98.8)	80.3 (77.1 to 83.2)	50.2 (41.4 to 58.9)	0.0	0.0
	Eswatini	Primary or lower	84.1 (70.6 to 92.1)	94.5 (75.4 to 99.0)	96.9 (62.8 to 99.8)	91.9	80.2
		Secondary or higher	92.9 (84.7 to 96.8)	96.6 (83.4 to 99.4)	97.7 (69.5 to 99.9)	94.4	85.2
	Ethiopia	Primary or lower	26.9 (25.3 to 28.6)	61.3 (59.3 to 63.3)	76.7 (73.9 to 79.2)	0.4	0.0
		Secondary or higher	64.3 (60.0 to 68.4)	83.7 (81.1 to 86.0)	89.7 (87.2 to 91.7)	100.0	37.9
	Gabon	Primary or lower	46.7 (42.8 to 50.6)	82.3 (78.3 to 85.7)	91.5 (87.8 to 94.1)	100.0	80.3
		Secondary or higher	61.2 (57.2 to 65.1)	84.8 (81.3 to 87.7)	91.3 (87.6 to 93.9)	100.0	77.2
	Gambia	Primary or lower	95.2 (91.3 to 97.4)	89.3 (87.2 to 91.1)	84.5 (75.8 to 90.4)	86.3	3.7
		Secondary or higher	97.2 (94.5 to 98.6)	90.7 (88.1 to 92.7)	83.8 (74.5 to 90.1)	81.0	2.7
	Ghana	Primary or lower	77.3 (72.8 to 81.4)	90.7 (87.5 to 93.1)	94.2 (90.3 to 96.6)	100.0	98.1
		Secondary or higher	89.5 (86.2 to 92.1)	94.1 (91.9 to 95.8)	95.7 (92.6 to 97.5)	100.0	99.9
	Guinea	Primary or lower	59.8 (55.9 to 63.6)	40.5 (37.6 to 43.4)	31.5 (26.7 to 36.7)	0.0	0.0
		Secondary or higher	81.9 (77.4 to 85.7)	57.8 (52.6 to 62.9)	43.0 (36.0 to 50.3)	0.0	0.0
	Kenya	Primary or lower	66.3 (62.6 to 69.9)	89.4 (87.8 to 90.9)	94.6 (92.8 to 95.9)	100.0	100.0
		Secondary or higher	87.2 (84.2 to 89.7)	95.1 (93.9 to 96.1)	97.0 (95.8 to 97.9)	100.0	100.0
	Lesotho	Primary or lower	76.0 (70.5 to 80.7)	87.2 (82.3 to 90.8)	90.9 (83.7 to 95.1)	99.6	61.6
		Secondary or higher	87.1 (82.7 to 90.5)	90.6 (86.9 to 93.3)	92.0 (85.8 to 95.6)	99.9	77.0
	Liberia	Primary or lower	55.2 (49.8 to 60.4)	70.9 (67.9 to 73.7)	77.4 (72.0 to 82.0)	14.1	0.0
		Secondary or higher	80.4 (75.4 to 84.6)	84.3 (81.4 to 86.9)	86.0 (81.6 to 89.5)	99.5	1.1
	Madagascar	Primary or lower	44.7 (39.5 to 50.1)	86.4 (80.5 to 90.7)	94.7 (89.5 to 97.4)	100.0	96.5
		Secondary or higher	79.1 (74.5 to 83.1)	95.2 (92.3 to 97.0)	97.8 (95.3 to 99.0)	100.0	100.0
	Malawi	Primary or lower	80.0 (78.6 to 81.4)	93.6 (92.7 to 94.4)	96.5 (95.6 to 97.2)	100.0	100.0
		Secondary or higher	91.6 (89.7 to 93.2)	96.3 (95.4 to 97.1)	97.6 (96.8 to 98.2)	100.0	100.0
	Mali	Primary or lower	53.2 (51.4 to 55.1)	73.9 (71.9 to 75.8)	81.7 (79.0 to 84.1)	89.5	0.0
		Secondary or higher	79.2 (75.6 to 82.4)	86.3 (83.7 to 88.4)	89.0 (86.2 to 91.2)	100.0	19.9
	Morocco	Primary or lower	86.9 (81.4 to 91.0)	95.5 (72.2 to 99.5)	97.4 (57.7 to 99.9)	91.0	80.6
		Secondary or higher	95.8 (92.3 to 97.8)	98.0 (85.1 to 99.8)	98.6 (71.3 to 100.0)	95.4	88.8
	Mozambique	Primary or lower	74.6 (71.6 to 77.4)	83.1 (79.1 to 86.5)	86.4 (80.2 to 90.9)	97.8	6.3
		Secondary or higher	93.7 (91.0 to 95.6)	94.3 (91.7 to 96.1)	94.5 (91.0 to 96.7)	100.0	99.2
	Namibia	Primary or lower	73.6 (70.2 to 76.8)	89.2 (85.5 to 92.1)	93.5 (89.3 to 96.1)	100.0	95.5
		Secondary or higher	86.3 (83.6 to 88.5)	92.5 (90.1 to 94.4)	94.6 (91.3 to 96.6)	100.0	99.5
	Niger	Primary or lower	26.4 (22.5 to 30.7)	86.8 (83.7 to 89.4)	96.6 (94.7 to 97.8)	100.0	100.0
		Secondary or higher	63.0 (54.2 to 71.1)	95.4 (93.4 to 96.8)	98.6 (97.6 to 99.2)	100.0	100.0
	Nigeria	Primary or lower	20.4 (18.8 to 22.1)	38.1 (36.5 to 39.7)	48.8 (45.4 to 52.2)	0.0	0.0
		Secondary or higher	65.0 (62.2 to 67.8)	74.7 (73.2 to 76.2)	78.9 (76.3 to 81.3)	18.4	0.0
	Rwanda	Primary or lower	76.9 (74.8 to 78.8)	89.1 (87.2 to 90.7)	92.8 (90.4 to 94.6)	100.0	98.8
		Secondary or higher	87.2 (84.3 to 89.6)	91.7 (89.4 to 93.6)	93.4 (90.6 to 95.4)	100.0	98.9
	Sao Tome and Principe	Primary or lower	74.1 (52.0 to 88.2)	91.4 (71.7 to 97.9)	95.3 (60.8 to 99.7)	89.5	73.6
		Secondary or higher	85.0 (65.0 to 94.4)	93.3 (76.2 to 98.4)	95.6 (62.3 to 99.7)	90.3	75.2
	Senegal	Primary or lower	67.7 (64.7 to 70.5)	84.8 (83.5 to 86.1)	90.2 (88.2 to 91.8)	100.0	56.6
		Secondary or higher	91.3 (88.8 to 93.2)	94.9 (93.6 to 95.9)	96.1 (94.8 to 97.1)	100.0	100.0
	Sierra Leone	Primary or lower	57.8 (52.4 to 63.0)	75.9 (73.6 to 78.0)	82.7 (78.7 to 86.1)	91.0	0.0
		Secondary or higher	75.0 (69.3 to 80.0)	82.1 (79.5 to 84.4)	85.0 (81.1 to 88.2)	99.3	0.1
	South Africa	Primary or lower	62.7 (24.8 to 89.7)	75.5 (61.6 to 85.4)	80.6 (46.1 to 95.2)	52.0	16.5
		Secondary or higher	84.3 (50.5 to 96.6)	86.7 (80.3 to 91.2)	87.8 (62.3 to 96.8)	78.6	37.8
	Tanzania	Primary or lower	77.3 (74.2 to 80.2)	87.1 (84.8 to 89.0)	90.5 (87.1 to 93.0)	100.0	61.7
		Secondary or higher	92.0 (89.3 to 94.1)	93.8 (91.8 to 95.3)	94.5 (92.0 to 96.3)	100.0	99.9
	Togo	Primary or lower	56.0 (26.4 to 81.8)	77.3 (62.8 to 87.3)	84.7 (51.2 to 96.7)	65.1	28.1
		Secondary or higher	80.4 (52.1 to 93.9)	87.9 (77.9 to 93.7)	90.6 (64.7 to 98.1)	85.3	53.1
	Uganda	Primary or lower	54.6 (52.2 to 57.0)	82.1 (80.4 to 83.8)	90.0 (88.0 to 91.6)	100.0	49.4
		Secondary or higher	74.3 (70.9 to 77.4)	88.0 (86.1 to 89.7)	92.1 (90.1 to 93.7)	100.0	98.2
	Zambia	Primary or lower	80.5 (78.3 to 82.5)	87.0 (85.4 to 88.5)	89.5 (87.1 to 91.5)	100.0	33.2
		Secondary or higher	90.8 (88.9 to 92.4)	91.4 (89.9 to 92.7)	91.7 (89.4 to 93.5)	100.0	93.3

Indicators	Country	Educational Level	Estimates and Projections of Immunization Coverage (%)			Probability Achieving Targets (%)	
			Year 2000	Year 2020	Year 2030	UHC2030	IA2030
	Zimbabwe	Primary or lower	47.1 (41.4 to 52.8)	84.4 (80.7 to 87.4)	93.0 (89.5 to 95.4)	100.0	95.7
		Secondary or higher	66.9 (61.7 to 71.6)	89.0 (86.5 to 91.1)	94.2 (91.4 to 96.1)	100.0	99.7
Polio3 immunization	Angola	Primary or lower	47.4 (17.3 to 79.8)	39.6 (28.4 to 51.9)	35.9 (11.3 to 70.8)	0.4	0.0
		Secondary or higher	80.0 (47.7 to 94.7)	64.1 (51.8 to 74.7)	54.4 (21.4 to 83.8)	5.2	0.4
	Benin	Primary or lower	75.6 (73.6 to 77.5)	74.1 (71.9 to 76.1)	73.3 (69.3 to 76.9)	0.0	0.0
		Secondary or higher	93.3 (91.6 to 94.7)	88.7 (86.3 to 90.8)	85.6 (81.6 to 88.8)	99.6	0.2
	Burkina Faso	Primary or lower	45.3 (41.5 to 49.2)	99.6 (99.5 to 99.8)	100.0 (100.0 to 100.0)	100.0	100.0
		Secondary or higher	77.7 (68.1 to 85.2)	99.9 (99.8 to 99.9)	100.0 (100.0 to 100.0)	100.0	100.0
	Burundi	Primary or lower	92.0 (85.5 to 95.8)	97.6 (96.6 to 98.4)	98.7 (97.1 to 99.5)	100.0	100.0
		Secondary or higher	96.1 (91.4 to 98.3)	98.2 (96.9 to 99.0)	98.8 (97.0 to 99.5)	100.0	100.0
	Cameroon	Primary or lower	74.3 (71.3 to 77.1)	72.3 (69.5 to 74.8)	71.2 (65.9 to 75.9)	0.0	0.0
		Secondary or higher	90.8 (88.9 to 92.5)	84.6 (82.4 to 86.5)	80.3 (75.9 to 84.1)	55.8	0.0
	Chad	Primary or lower	34.4 (30.2 to 38.8)	62.0 (58.5 to 65.3)	74.2 (68.3 to 79.3)	1.2	0.0
		Secondary or higher	70.1 (63.5 to 76.0)	81.8 (77.4 to 85.5)	86.1 (80.9 to 90.1)	98.7	2.9
	Comoros	Primary or lower	59.3 (28.8 to 83.9)	83.9 (68.1 to 92.8)	90.8 (58.5 to 98.6)	82.2	53.8
		Secondary or higher	74.7 (43.7 to 91.8)	86.7 (72.7 to 94.1)	90.7 (58.5 to 98.5)	81.9	53.0
	Congo Brazzaville	Primary or lower	77.0 (71.1 to 82.1)	60.3 (51.7 to 68.4)	50.6 (34.7 to 66.3)	0.0	0.0
		Secondary or higher	88.1 (84.4 to 91.1)	67.5 (59.1 to 74.9)	52.3 (36.1 to 68.0)	0.0	0.0
	Congo Democratic Republic	Primary or lower	27.1 (22.7 to 32.0)	87.2 (84.7 to 89.3)	96.7 (95.1 to 97.8)	100.0	100.0
		Secondary or higher	49.9 (43.6 to 56.3)	91.8 (89.9 to 93.4)	97.4 (96.1 to 98.3)	100.0	100.0
	Cote d'Ivoire	Primary or lower	62.9 (35.2 to 84.1)	85.5 (69.5 to 93.8)	91.7 (60.2 to 98.8)	84.5	58.0
		Secondary or higher	84.9 (61.5 to 95.2)	92.4 (81.8 to 97.0)	94.7 (71.1 to 99.2)	93.2	75.2
	Egypt	Primary or lower	94.0 (92.9 to 94.9)	98.7 (98.1 to 99.1)	99.4 (98.9 to 99.7)	100.0	100.0
		Secondary or higher	96.7 (95.9 to 97.3)	98.9 (98.4 to 99.2)	99.3 (98.8 to 99.6)	100.0	100.0
	Eswatini	Primary or lower	88.2 (76.7 to 94.4)	97.5 (87.2 to 99.6)	98.9 (82.9 to 99.9)	98.1	94.0
		Secondary or higher	94.1 (86.7 to 97.5)	98.1 (90.1 to 99.7)	98.9 (83.7 to 99.9)	98.3	94.4
	Ethiopia	Primary or lower	35.3 (33.5 to 37.1)	64.2 (62.2 to 66.1)	76.5 (73.7 to 79.0)	0.3	0.0
		Secondary or higher	73.7 (69.8 to 77.3)	85.0 (82.4 to 87.3)	89.0 (86.3 to 91.2)	100.0	19.7
	Gabon	Primary or lower	34.9 (31.4 to 38.7)	55.4 (49.5 to 61.2)	65.4 (56.5 to 73.4)	0.0	0.0
		Secondary or higher	38.1 (34.3 to 42.1)	46.8 (41.3 to 52.4)	51.2 (42.2 to 60.1)	0.0	0.0
	Gambia	Primary or lower	94.7 (89.4 to 97.5)	94.1 (92.5 to 95.4)	93.8 (88.4 to 96.8)	100.0	93.1
		Secondary or higher	97.7 (94.8 to 99.0)	95.8 (94.1 to 97.1)	94.5 (89.4 to 97.2)	100.0	96.1
	Ghana	Primary or lower	76.4 (71.6 to 80.6)	92.4 (89.6 to 94.5)	95.9 (92.9 to 97.7)	100.0	99.9
		Secondary or higher	90.8 (87.6 to 93.3)	95.8 (94.0 to 97.1)	97.2 (95.0 to 98.5)	100.0	100.0
	Guinea	Primary or lower	63.5 (59.6 to 67.2)	53.8 (50.8 to 56.7)	48.7 (42.9 to 54.6)	0.0	0.0
		Secondary or higher	87.5 (83.7 to 90.5)	74.1 (69.3 to 78.5)	64.8 (57.2 to 71.6)	0.0	0.0
	Kenya	Primary or lower	68.8 (64.9 to 72.4)	95.0 (94.0 to 95.8)	98.2 (97.5 to 98.7)	100.0	100.0
		Secondary or higher	85.2 (81.7 to 88.2)	96.8 (95.9 to 97.5)	98.6 (97.9 to 99.0)	100.0	100.0
	Lesotho	Primary or lower	81.1 (76.3 to 85.2)	84.0 (78.2 to 88.4)	85.2 (74.8 to 91.8)	86.0	9.4
		Secondary or higher	90.1 (86.5 to 92.9)	87.3 (82.6 to 90.8)	85.6 (75.7 to 91.8)	88.4	10.0
	Liberia	Primary or lower	53.3 (47.8 to 58.7)	77.8 (75.1 to 80.3)	86.0 (82.0 to 89.2)	99.8	0.6
		Secondary or higher	74.5 (68.6 to 79.6)	84.7 (81.8 to 87.2)	88.4 (84.4 to 91.4)	100.0	16.3
	Madagascar	Primary or lower	52.6 (47.0 to 58.1)	86.2 (80.0 to 90.7)	93.7 (87.3 to 97.0)	100.0	89.9
		Secondary or higher	87.0 (83.3 to 90.0)	95.9 (93.2 to 97.5)	97.7 (94.9 to 99.0)	100.0	100.0
	Malawi	Primary or lower	82.5 (81.2 to 83.8)	94.1 (93.2 to 94.8)	96.7 (95.8 to 97.4)	100.0	100.0
		Secondary or higher	94.2 (92.6 to 95.5)	97.1 (96.3 to 97.8)	98.0 (97.2 to 98.6)	100.0	100.0
	Mali	Primary or lower	54.3 (52.5 to 56.2)	76.7 (74.9 to 78.5)	84.6 (82.2 to 86.7)	100.0	0.0
		Secondary or higher	82.7 (79.2 to 85.6)	89.0 (86.8 to 91.0)	91.4 (89.0 to 93.3)	100.0	88.1
	Morocco	Primary or lower	93.1 (89.4 to 95.5)	98.5 (88.8 to 99.8)	99.3 (83.7 to 100.0)	98.2	94.9
		Secondary or higher	97.9 (95.6 to 99.0)	99.3 (94.1 to 99.9)	99.6 (89.3 to 100.0)	99.0	97.1
	Mozambique	Primary or lower	71.8 (68.7 to 74.7)	87.4 (84.2 to 90.1)	92.0 (87.9 to 94.8)	100.0	85.2
		Secondary or higher	90.7 (87.2 to 93.3)	94.2 (91.8 to 96.0)	95.5 (92.6 to 97.3)	100.0	99.9
	Namibia	Primary or lower	81.9 (78.9 to 84.5)	86.1 (81.5 to 89.7)	87.9 (80.8 to 92.5)	98.4	21.4
		Secondary or higher	89.1 (86.7 to 91.0)	87.3 (83.6 to 90.3)	86.3 (79.3 to 91.2)	96.4	7.8
	Niger	Primary or lower	36.0 (31.1 to 41.2)	93.0 (91.0 to 94.6)	98.5 (97.5 to 99.1)	100.0	100.0

Indicators	Country	Educational Level	Estimates and Projections of Immunization Coverage (%)			Probability Achieving Targets (%)	
			Year 2000	Year 2020	Year 2030	UHC2030	IA2030
		Secondary or higher	68.9 (59.7 to 76.8)	97.0 (95.4 to 98.0)	99.2 (98.5 to 99.6)	100.0	100.0
	Nigeria	Primary or lower	26.3 (24.4 to 28.2)	58.9 (57.3 to 60.4)	74.1 (71.5 to 76.6)	0.0	0.0
		Secondary or higher	68.1 (65.2 to 70.8)	84.0 (82.8 to 85.2)	89.2 (87.6 to 90.7)	100.0	15.0
	Rwanda	Primary or lower	85.2 (83.3 to 86.8)	96.8 (95.9 to 97.5)	98.6 (97.9 to 99.1)	100.0	100.0
		Secondary or higher	91.1 (88.4 to 93.2)	97.1 (95.9 to 98.0)	98.4 (97.3 to 99.0)	100.0	100.0
	Sao Tome and Principe	Primary or lower	80.0 (59.9 to 91.3)	95.9 (84.7 to 99.1)	98.3 (81.2 to 99.9)	97.8	92.0
		Secondary or higher	87.2 (68.0 to 95.5)	96.1 (84.9 to 99.1)	97.9 (78.2 to 99.9)	97.0	89.7
	Senegal	Primary or lower	73.0 (69.8 to 76.0)	93.6 (92.8 to 94.4)	97.2 (96.4 to 97.8)	100.0	100.0
		Secondary or higher	92.5 (89.8 to 94.5)	97.6 (96.8 to 98.2)	98.7 (98.1 to 99.1)	100.0	100.0
	Sierra Leone	Primary or lower	53.0 (46.9 to 59.0)	89.1 (87.5 to 90.5)	95.7 (94.1 to 96.8)	100.0	100.0
		Secondary or higher	70.9 (63.7 to 77.3)	91.6 (89.8 to 93.1)	95.8 (94.2 to 97.0)	100.0	100.0
	South Africa	Primary or lower	53.7 (19.4 to 85.1)	72.7 (58.1 to 83.6)	80.1 (45.7 to 95.0)	50.3	15.1
		Secondary or higher	69.5 (31.2 to 92.1)	76.3 (67.4 to 83.3)	79.3 (47.7 to 94.0)	47.4	11.4
	Tanzania	Primary or lower	85.6 (83.0 to 87.9)	89.5 (87.4 to 91.3)	91.1 (87.5 to 93.8)	100.0	74.9
		Secondary or higher	94.8 (92.7 to 96.4)	94.2 (92.2 to 95.8)	93.9 (90.7 to 96.0)	100.0	98.9
	Togo	Primary or lower	68.2 (36.7 to 88.7)	91.6 (84.0 to 95.8)	96.1 (81.5 to 99.3)	98.1	87.6
		Secondary or higher	85.8 (59.7 to 96.0)	95.0 (89.8 to 97.6)	97.1 (85.8 to 99.5)	99.2	93.4
	Uganda	Primary or lower	55.0 (52.6 to 57.5)	88.2 (86.9 to 89.5)	94.9 (93.7 to 95.8)	100.0	100.0
		Secondary or higher	72.7 (69.1 to 76.1)	91.0 (89.4 to 92.3)	95.1 (93.8 to 96.2)	100.0	100.0
	Zambia	Primary or lower	76.6 (74.2 to 78.8)	88.6 (87.1 to 89.9)	92.3 (90.4 to 93.8)	100.0	99.0
		Secondary or higher	90.5 (88.5 to 92.2)	93.3 (92.0 to 94.4)	94.4 (92.7 to 95.7)	100.0	100.0
	Zimbabwe	Primary or lower	44.5 (39.0 to 50.1)	85.4 (82.0 to 88.3)	94.1 (91.1 to 96.1)	100.0	99.4
		Secondary or higher	62.4 (57.1 to 67.4)	88.2 (85.6 to 90.4)	94.1 (91.3 to 96.0)	100.0	99.6
DPT3 immunization	Angola	Primary or lower	32.3 (9.4 to 69.8)	28.9 (19.2 to 40.7)	27.3 (7.1 to 63.6)	0.1	0.0
		Secondary or higher	71.5 (34.9 to 92.5)	56.7 (43.3 to 68.9)	48.7 (16.3 to 81.5)	3.3	0.2
	Benin	Primary or lower	66.5 (64.3 to 68.6)	72.0 (69.8 to 74.0)	74.5 (70.8 to 77.9)	0.0	0.0
		Secondary or higher	90.1 (87.9 to 92.0)	87.9 (85.5 to 89.9)	86.6 (83.1 to 89.4)	100.0	0.8
	Burkina Faso	Primary or lower	37.5 (34.2 to 40.9)	99.1 (98.7 to 99.3)	99.9 (99.9 to 100.0)	100.0	100.0
		Secondary or higher	63.5 (53.9 to 72.2)	99.5 (99.2 to 99.7)	99.9 (99.9 to 100.0)	100.0	100.0
	Burundi	Primary or lower	90.0 (82.6 to 94.4)	97.5 (96.5 to 98.3)	98.8 (97.4 to 99.5)	100.0	100.0
		Secondary or higher	92.5 (85.1 to 96.4)	97.1 (95.4 to 98.2)	98.2 (95.9 to 99.2)	100.0	100.0
	Cameroon	Primary or lower	56.5 (53.1 to 59.7)	64.3 (61.4 to 67.0)	67.9 (63.0 to 72.6)	0.0	0.0
		Secondary or higher	83.9 (81.2 to 86.2)	81.6 (79.3 to 83.6)	80.3 (76.3 to 83.8)	56.5	0.0
	Chad	Primary or lower	15.5 (12.8 to 18.7)	38.7 (35.0 to 42.6)	54.0 (45.9 to 61.9)	0.0	0.0
		Secondary or higher	42.2 (35.2 to 49.5)	60.6 (54.5 to 66.4)	69.1 (60.1 to 76.9)	0.2	0.0
	Comoros	Primary or lower	48.4 (20.2 to 77.8)	80.3 (62.0 to 91.0)	89.4 (53.5 to 98.4)	77.3	47.4
		Secondary or higher	68.3 (35.6 to 89.4)	85.1 (69.7 to 93.4)	90.3 (56.2 to 98.5)	80.3	51.4
	Congo Brazzaville	Primary or lower	64.2 (57.7 to 70.3)	40.0 (32.7 to 47.8)	28.9 (18.4 to 42.3)	0.0	0.0
		Secondary or higher	81.8 (77.3 to 85.6)	50.6 (42.6 to 58.6)	32.9 (21.3 to 46.9)	0.0	0.0
	Congo Democratic Republic	Primary or lower	21.6 (17.9 to 25.9)	71.3 (67.3 to 75.1)	88.2 (83.5 to 91.6)	99.9	16.9
		Secondary or higher	44.5 (38.6 to 50.6)	81.6 (78.2 to 84.5)	91.2 (87.5 to 94.0)	100.0	76.3
	Cote d'Ivoire	Primary or lower	45.2 (20.4 to 72.9)	73.3 (50.8 to 87.8)	83.3 (39.2 to 97.4)	58.5	28.0
		Secondary or higher	77.3 (49.2 to 92.4)	87.4 (71.7 to 95.0)	90.8 (56.2 to 98.7)	81.1	53.6
	Egypt	Primary or lower	91.0 (89.8 to 92.1)	97.8 (97.0 to 98.4)	98.9 (98.2 to 99.3)	100.0	100.0
		Secondary or higher	95.2 (94.3 to 95.9)	98.1 (97.5 to 98.6)	98.9 (98.2 to 99.3)	100.0	100.0
	Eswatini	Primary or lower	88.4 (76.7 to 94.5)	97.9 (89.0 to 99.7)	99.2 (86.2 to 100.0)	98.7	95.7
		Secondary or higher	94.8 (87.8 to 97.8)	98.6 (92.3 to 99.8)	99.3 (88.0 to 100.0)	99.0	96.6
	Ethiopia	Primary or lower	20.9 (19.5 to 22.4)	64.4 (62.4 to 66.3)	82.6 (80.3 to 84.6)	98.6	0.0
		Secondary or higher	57.3 (52.8 to 61.7)	84.9 (82.4 to 87.1)	92.0 (90.0 to 93.6)	100.0	97.6
	Gabon	Primary or lower	31.0 (27.5 to 34.7)	89.0 (86.2 to 91.3)	97.2 (95.8 to 98.1)	100.0	100.0
		Secondary or higher	50.3 (46.2 to 54.5)	91.8 (89.6 to 93.6)	97.4 (96.1 to 98.3)	100.0	100.0
	Gambia	Primary or lower	71.1 (57.8 to 81.6)	93.4 (91.8 to 94.6)	97.1 (95.0 to 98.4)	100.0	100.0
		Secondary or higher	78.0 (65.0 to 87.1)	92.6 (90.4 to 94.2)	95.9 (92.9 to 97.7)	100.0	99.9
	Ghana	Primary or lower	71.2 (66.3 to 75.7)	90.4 (87.3 to 92.8)	94.8 (91.4 to 96.9)	100.0	99.5
		Secondary or higher	87.1 (83.4 to 90.1)	94.0 (91.8 to 95.7)	96.0 (93.3 to 97.6)	100.0	100.0

Indicators	Country	Educational Level	Estimates and Projections of Immunization Coverage (%)			Probability Achieving Targets (%)	
			Year 2000	Year 2020	Year 2030	UHC2030	IA2030
	Guinea	Primary or lower	55.9 (51.9 to 59.9)	37.0 (34.2 to 39.9)	28.6 (24.0 to 33.6)	0.0	0.0
		Secondary or higher	81.5 (76.8 to 85.3)	55.5 (50.2 to 60.7)	39.9 (33.1 to 47.1)	0.0	0.0
	Kenya	Primary or lower	64.3 (60.5 to 68.0)	93.7 (92.6 to 94.7)	97.7 (96.9 to 98.3)	100.0	100.0
		Secondary or higher	81.7 (77.9 to 85.0)	95.8 (94.7 to 96.6)	98.1 (97.2 to 98.7)	100.0	100.0
	Lesotho	Primary or lower	77.7 (72.5 to 82.1)	85.2 (80.0 to 89.3)	88.2 (79.6 to 93.4)	97.0	28.2
		Secondary or higher	87.0 (82.6 to 90.3)	87.2 (82.7 to 90.7)	87.3 (78.7 to 92.8)	95.6	19.7
	Liberia	Primary or lower	38.4 (33.6 to 43.4)	72.3 (69.4 to 75.0)	84.2 (80.2 to 87.6)	97.9	0.0
		Secondary or higher	61.5 (54.9 to 67.6)	80.4 (77.2 to 83.2)	86.8 (82.7 to 90.0)	99.9	2.4
	Madagascar	Primary or lower	45.8 (40.5 to 51.3)	89.6 (84.7 to 93.1)	96.5 (92.8 to 98.3)	100.0	99.8
		Secondary or higher	82.6 (78.2 to 86.2)	96.7 (94.6 to 98.0)	98.7 (97.0 to 99.4)	100.0	100.0
	Malawi	Primary or lower	81.3 (79.9 to 82.6)	94.6 (93.8 to 95.3)	97.2 (96.5 to 97.8)	100.0	100.0
		Secondary or higher	92.8 (90.9 to 94.2)	96.9 (96.1 to 97.6)	98.0 (97.3 to 98.6)	100.0	100.0
	Mali	Primary or lower	43.9 (42.1 to 45.7)	78.7 (76.9 to 80.4)	88.9 (87.1 to 90.5)	100.0	9.4
		Secondary or higher	74.2 (70.0 to 78.0)	89.3 (87.1 to 91.1)	93.4 (91.6 to 94.9)	100.0	100.0
	Morocco	Primary or lower	92.2 (88.3 to 94.9)	98.7 (89.8 to 99.8)	99.5 (86.5 to 100.0)	98.6	96.0
		Secondary or higher	97.5 (94.7 to 98.8)	99.3 (94.3 to 99.9)	99.7 (90.7 to 100.0)	99.2	97.6
	Mozambique	Primary or lower	69.6 (66.5 to 72.6)	77.8 (73.3 to 81.7)	81.2 (74.1 to 86.8)	64.6	0.0
		Secondary or higher	88.7 (85.1 to 91.5)	88.0 (84.0 to 91.1)	87.7 (81.6 to 91.9)	99.1	16.2
	Namibia	Primary or lower	75.2 (71.9 to 78.2)	84.0 (79.3 to 87.7)	87.3 (80.8 to 91.8)	98.5	14.2
		Secondary or higher	84.7 (82.0 to 87.1)	85.5 (81.7 to 88.5)	85.8 (79.4 to 90.5)	96.4	4.2
	Niger	Primary or lower	16.1 (13.4 to 19.3)	90.6 (88.3 to 92.5)	98.6 (97.7 to 99.1)	100.0	100.0
		Secondary or higher	44.9 (36.4 to 53.7)	96.2 (94.5 to 97.3)	99.3 (98.7 to 99.6)	100.0	100.0
	Nigeria	Primary or lower	10.7 (9.7 to 11.9)	33.8 (32.3 to 35.4)	51.3 (47.7 to 54.8)	0.0	0.0
		Secondary or higher	56.9 (53.8 to 60.0)	77.5 (76.0 to 78.9)	84.7 (82.6 to 86.6)	100.0	0.0
	Rwanda	Primary or lower	79.8 (77.8 to 81.7)	96.7 (95.9 to 97.4)	98.8 (98.2 to 99.2)	100.0	100.0
		Secondary or higher	85.3 (81.9 to 88.2)	96.4 (95.1 to 97.4)	98.3 (97.3 to 98.9)	100.0	100.0
	Sao Tome and Principe	Primary or lower	74.6 (52.2 to 88.5)	95.2 (82.2 to 98.9)	98.1 (79.1 to 99.9)	97.3	90.6
		Secondary or higher	85.5 (64.7 to 94.9)	96.1 (84.5 to 99.1)	98.0 (78.5 to 99.9)	97.1	90.1
	Senegal	Primary or lower	67.7 (64.4 to 70.8)	92.1 (91.2 to 93.0)	96.5 (95.6 to 97.2)	100.0	100.0
		Secondary or higher	92.9 (90.3 to 94.9)	97.8 (97.1 to 98.4)	98.8 (98.3 to 99.2)	100.0	100.0
	Sierra Leone	Primary or lower	35.4 (30.4 to 40.8)	88.4 (86.8 to 89.8)	96.6 (95.4 to 97.5)	100.0	100.0
		Secondary or higher	51.4 (43.9 to 58.9)	90.0 (88.2 to 91.6)	96.3 (95.0 to 97.3)	100.0	100.0
	South Africa	Primary or lower	39.5 (11.2 to 77.8)	61.0 (45.0 to 74.8)	70.8 (32.6 to 92.1)	26.4	4.9
		Secondary or higher	57.3 (20.3 to 88.1)	66.4 (55.7 to 75.5)	70.6 (35.0 to 91.1)	24.4	3.7
	Tanzania	Primary or lower	86.1 (83.6 to 88.3)	89.2 (87.0 to 91.1)	90.5 (86.7 to 93.4)	100.0	61.9
		Secondary or higher	94.8 (92.7 to 96.3)	93.7 (91.5 to 95.3)	93.1 (89.5 to 95.5)	100.0	95.9
	Togo	Primary or lower	55.9 (24.6 to 82.8)	89.0 (79.4 to 94.5)	95.4 (77.9 to 99.2)	96.6	82.3
		Secondary or higher	76.1 (43.6 to 92.8)	92.6 (85.3 to 96.5)	96.1 (81.1 to 99.3)	97.9	87.3
	Uganda	Primary or lower	44.8 (42.4 to 47.1)	83.9 (82.3 to 85.4)	93.0 (91.6 to 94.1)	100.0	100.0
		Secondary or higher	61.6 (57.8 to 65.3)	86.4 (84.4 to 88.1)	92.6 (90.8 to 94.1)	100.0	99.7
	Zambia	Primary or lower	73.2 (70.7 to 75.6)	89.0 (87.6 to 90.3)	93.3 (91.7 to 94.6)	100.0	100.0
		Secondary or higher	88.8 (86.6 to 90.7)	93.5 (92.3 to 94.5)	95.1 (93.7 to 96.2)	100.0	100.0
	Zimbabwe	Primary or lower	42.1 (36.6 to 47.8)	90.4 (87.8 to 92.5)	97.2 (95.5 to 98.2)	100.0	100.0
		Secondary or higher	57.6 (52.1 to 63.0)	91.6 (89.5 to 93.3)	96.8 (95.2 to 97.9)	100.0	100.0

Notes: Countries are listed in alphabetical order. UHC = Probability of achieving the Universal Health Coverage (UHC) benchmark (≥80% coverage) by 2030. IA = Probability of achieving the Immunization Agenda 2030 (IA2030) target (≥90% coverage) by 2030.

Table I. AARC and Additional values required to reach 2030 targets, at national level

Indicators	Country	Average Annual Rate of Change (95% CrI)			Additional AARC Required to Reach Targets (95% CrI)	
		2000–2030	2000–2020	2020–2030	UHC2030	IA2030
Full immunization	Angola	0.61 (-2.46 to 3.91)	0.66 (-1.78 to 5.23)	0.51 (-3.83 to 1.31)	1.36 (0.10 to 7.32)	1.67 (0.12 to 7.53)
	Benin	-0.07 (-0.23 to 0.05)	-0.06 (-0.21 to 0.06)	-0.07 (-0.26 to 0.05)	–	0.32 (0.07 to 0.66)
	Burkina Faso	1.32 (1.12 to 1.53)	1.98 (1.66 to 2.30)	0.03 (0.02 to 0.05)	–	–
	Burundi	-0.06 (-0.24 to 0.06)	-0.05 (-0.14 to 0.07)	-0.08 (-0.45 to 0.04)	–	0.37 (0.13 to 0.62)
	Cameroon	0.05 (-0.13 to 0.27)	0.05 (-0.12 to 0.29)	0.05 (-0.14 to 0.22)	–	0.31 (0.02 to 0.70)
	Chad	3.15 (2.59 to 3.69)	3.83 (3.07 to 4.63)	1.78 (1.63 to 1.88)	0.06 (0.02 to 0.17)	0.75 (0.35 to 1.24)
	Comoros	0.77 (-0.89 to 2.71)	0.93 (-0.68 to 3.88)	0.39 (-1.23 to 0.52)	0.75 (0.07 to 3.82)	0.60 (0.02 to 3.83)
	Congo Brazzaville	0.49 (0.28 to 0.76)	0.65 (0.33 to 1.07)	0.16 (0.12 to 0.20)	–	–
	Congo Democratic Republic	1.97 (1.54 to 2.34)	2.66 (2.00 to 3.24)	0.58 (0.52 to 0.64)	–	–
	Cote d'Ivoire	0.92 (-0.50 to 3.00)	1.14 (-0.44 to 4.34)	0.44 (-0.62 to 0.54)	1.27 (0.25 to 4.48)	0.47 (0.02 to 3.34)
	Egypt	0.00 (-0.04 to 0.03)	0.00 (-0.03 to 0.03)	0.00 (-0.05 to 0.02)	–	–
	Eswatini	0.16 (-0.14 to 0.37)	0.23 (-0.11 to 0.55)	0.04 (-0.19 to 0.07)	–	0.19 (0.19 to 0.19)
	Ethiopia	1.82 (1.64 to 1.99)	2.16 (1.90 to 2.39)	1.15 (1.09 to 1.20)	–	0.65 (0.40 to 0.90)
	Gabon	0.20 (0.03 to 0.34)	0.22 (0.03 to 0.41)	0.16 (0.03 to 0.21)	–	0.03 (0.00 to 0.06)
	Gambia	0.11 (-0.01 to 0.39)	0.14 (-0.01 to 0.56)	0.04 (-0.01 to 0.06)	–	–
	Ghana	0.36 (0.26 to 0.51)	0.49 (0.33 to 0.72)	0.11 (0.07 to 0.14)	–	–
	Guinea	-0.61 (-1.00 to -0.26)	-0.55 (-0.84 to -0.25)	-0.73 (-1.34 to -0.28)	1.40 (0.69 to 2.37)	2.60 (1.88 to 3.57)
	Kenya	0.56 (0.44 to 0.69)	0.79 (0.60 to 0.99)	0.11 (0.09 to 0.13)	–	–
	Lesotho	0.16 (0.03 to 0.29)	0.20 (0.03 to 0.40)	0.08 (0.03 to 0.10)	–	–
	Liberia	1.28 (0.98 to 1.68)	1.74 (1.29 to 2.36)	0.37 (0.33 to 0.41)	–	–
	Madagascar	1.43 (1.19 to 1.71)	1.99 (1.59 to 2.44)	0.32 (0.22 to 0.41)	–	–
	Malawi	0.29 (0.25 to 0.33)	0.38 (0.33 to 0.45)	0.09 (0.08 to 0.10)	–	–
	Mali	0.92 (0.79 to 1.03)	1.10 (0.92 to 1.25)	0.56 (0.53 to 0.59)	–	0.03 (0.00 to 0.04)
	Morocco	0.07 (-0.03 to 0.14)	0.10 (-0.03 to 0.20)	0.02 (-0.04 to 0.04)	–	–
	Mozambique	0.41 (0.24 to 0.53)	0.50 (0.27 to 0.68)	0.23 (0.18 to 0.25)	–	–
	Namibia	0.24 (0.15 to 0.35)	0.29 (0.18 to 0.45)	0.13 (0.11 to 0.15)	–	–
	Niger	3.23 (2.76 to 3.73)	4.68 (3.93 to 5.48)	0.39 (0.31 to 0.49)	–	–
	Nigeria	2.57 (2.34 to 2.84)	3.02 (2.72 to 3.37)	1.68 (1.59 to 1.77)	0.17 (0.02 to 0.39)	1.37 (1.15 to 1.59)
	Rwanda	0.12 (0.07 to 0.16)	0.15 (0.08 to 0.21)	0.06 (0.05 to 0.07)	–	–
	Sao Tome and Principe	0.27 (-0.06 to 0.73)	0.35 (-0.05 to 1.07)	0.07 (-0.06 to 0.13)	1.84 (1.67 to 2.01)	1.46 (0.05 to 3.18)
	Senegal	0.27 (0.18 to 0.37)	0.33 (0.21 to 0.47)	0.15 (0.13 to 0.17)	–	–
	Sierra Leone	1.79 (1.35 to 2.30)	2.60 (1.94 to 3.37)	0.18 (0.14 to 0.21)	–	–
	South Africa	0.47 (-0.46 to 2.31)	0.57 (-0.36 to 3.25)	0.27 (-0.65 to 0.44)	0.38 (0.13 to 0.66)	0.45 (0.08 to 1.81)
	Tanzania	0.37 (0.26 to 0.49)	0.48 (0.32 to 0.67)	0.13 (0.11 to 0.15)	–	–
	Togo	0.47 (0.05 to 1.21)	0.64 (0.05 to 1.73)	0.14 (0.04 to 0.19)	–	–
	Uganda	0.81 (0.72 to 0.94)	1.14 (1.00 to 1.34)	0.15 (0.13 to 0.17)	–	–
	Zambia	0.20 (0.14 to 0.27)	0.24 (0.16 to 0.34)	0.12 (0.10 to 0.14)	–	–
	Zimbabwe	1.32 (1.05 to 1.58)	1.80 (1.37 to 2.22)	0.38 (0.32 to 0.43)	–	–
BCG immunization	Angola	0.53 (-4.84 to 5.73)	0.54 (-4.11 to 6.79)	0.51 (-6.28 to 3.55)	6.46 (1.04 to 16.56)	7.73 (2.28 to 17.86)
	Benin	0.45 (0.21 to 0.67)	0.48 (0.21 to 0.73)	0.39 (0.20 to 0.55)	0.29 (0.02 to 0.73)	1.46 (1.07 to 1.90)
	Burkina Faso	3.25 (2.93 to 3.58)	4.88 (4.38 to 5.38)	0.08 (0.06 to 0.11)	–	–
	Burundi	0.29 (0.08 to 0.54)	0.38 (0.09 to 0.75)	0.13 (0.07 to 0.14)	–	–
	Cameroon	0.59 (0.33 to 0.92)	0.64 (0.35 to 1.02)	0.51 (0.31 to 0.72)	0.54 (0.18 to 1.00)	1.74 (1.25 to 2.20)
	Chad	4.37 (3.27 to 5.49)	4.90 (3.53 to 6.40)	3.32 (2.76 to 3.79)	3.16 (1.91 to 4.68)	4.42 (3.16 to 5.96)
	Comoros	1.65 (-0.57 to 4.48)	2.02 (-0.52 to 6.41)	0.84 (-0.66 to 1.04)	0.98 (0.05 to 3.86)	0.86 (0.02 to 5.05)
	Congo Brazzaville	-2.42 (-3.93 to -0.82)	-2.07 (-3.12 to -0.77)	-3.11 (-5.53 to -0.91)	8.21 (4.30 to 12.12)	9.46 (5.52 to 13.39)
	Congo Democratic Republic	3.82 (3.21 to 4.46)	4.88 (3.98 to 5.91)	1.70 (1.61 to 1.77)	–	0.16 (0.01 to 0.56)
	Cote d'Ivoire	2.05 (-1.10 to 5.43)	2.48 (-1.00 to 7.78)	1.13 (-1.30 to 1.35)	1.30 (0.07 to 5.21)	1.09 (0.10 to 5.69)
	Egypt	0.21 (0.17 to 0.25)	0.27 (0.20 to 0.34)	0.09 (0.08 to 0.10)	–	–
	Eswatini	0.31 (-0.08 to 0.70)	0.44 (-0.07 to 1.03)	0.07 (-0.09 to 0.13)	–	0.95 (0.78 to 1.13)
	Ethiopia	4.46 (4.18 to 4.76)	5.53 (5.11 to 5.96)	2.35 (2.27 to 2.43)	–	0.75 (0.57 to 1.00)
	Gabon	2.89 (2.61 to 3.16)	4.01 (3.57 to 4.45)	0.67 (0.58 to 0.80)	–	–
	Gambia	1.01 (0.54 to 1.80)	1.32 (0.64 to 2.49)	0.39 (0.32 to 0.45)	–	–
	Ghana	0.70 (0.44 to 0.89)	0.87 (0.50 to 1.17)	0.35 (0.30 to 0.40)	–	–
	Guinea	-1.78 (-2.41 to -1.08)	-1.65 (-2.19 to -1.03)	-2.02 (-2.85 to -1.17)	9.06 (7.57 to 10.60)	10.33 (8.83 to 11.88)
	Kenya	1.27 (1.09 to 1.45)	1.73 (1.44 to 2.00)	0.36 (0.32 to 0.39)	–	–
	Lesotho	0.34 (-0.17 to 0.64)	0.37 (-0.16 to 0.76)	0.27 (-0.18 to 0.41)	0.20 (0.10 to 0.44)	0.28 (0.02 to 1.16)
	Liberia	2.41 (1.92 to 2.89)	2.94 (2.27 to 3.62)	1.35 (1.22 to 1.46)	–	0.45 (0.13 to 0.89)
	Madagascar	1.96 (1.58 to 2.38)	2.59 (2.00 to 3.33)	0.65 (0.50 to 0.78)	–	–
	Malawi	0.58 (0.51 to 0.65)	0.75 (0.63 to 0.85)	0.25 (0.23 to 0.27)	–	–
	Mali	2.36 (2.20 to 2.52)	2.98 (2.74 to 3.22)	1.15 (1.11 to 1.18)	–	0.03 (0.00 to 0.14)
	Morocco	0.22 (-0.08 to 0.35)	0.31 (-0.07 to 0.52)	0.05 (-0.09 to 0.11)	2.21 (1.75 to 2.68)	2.04 (0.26 to 3.82)
	Mozambique	0.69 (0.41 to 0.98)	0.77 (0.43 to 1.16)	0.53 (0.35 to 0.64)	0.30 (0.16 to 0.68)	0.54 (0.09 to 1.16)
	Namibia	0.35 (0.07 to 0.59)	0.38 (0.07 to 0.69)	0.28 (0.07 to 0.38)	0.39 (0.39 to 0.39)	0.22 (0.01 to 0.79)

Indicators	Country	Average Annual Rate of Change (95% CrI)			Additional AARC Required to Reach Targets (95% CrI)	
		2000–2030	2000–2020	2020–2030	UHC2030	IA2030
	Niger	5.94 (5.25 to 6.57)	8.58 (7.43 to 9.62)	0.83 (0.69 to 1.01)	–	–
	Nigeria	3.92 (3.55 to 4.28)	4.51 (4.05 to 5.00)	2.73 (2.57 to 2.87)	1.49 (1.08 to 1.90)	2.73 (2.31 to 3.13)
	Rwanda	0.68 (0.61 to 0.77)	0.92 (0.80 to 1.05)	0.21 (0.18 to 0.24)	–	–
	Sao Tome and Principe	0.80 (-0.33 to 1.97)	1.04 (-0.30 to 2.93)	0.24 (-0.40 to 0.35)	1.14 (0.33 to 1.62)	0.97 (0.22 to 2.72)
	Senegal	1.19 (1.02 to 1.34)	1.57 (1.32 to 1.81)	0.42 (0.39 to 0.44)	–	–
	Sierra Leone	3.32 (2.87 to 3.85)	4.57 (3.88 to 5.35)	0.85 (0.78 to 0.91)	–	–
	South Africa	1.29 (-1.74 to 5.57)	1.43 (-1.46 to 7.50)	0.99 (-2.31 to 2.00)	1.49 (0.10 to 6.13)	2.06 (0.25 to 6.70)
	Tanzania	0.21 (-0.01 to 0.38)	0.23 (-0.01 to 0.44)	0.17 (-0.01 to 0.26)	–	0.09 (0.01 to 0.38)
	Togo	1.55 (-0.30 to 4.01)	2.02 (-0.28 to 5.81)	0.57 (-0.35 to 0.67)	0.91 (0.04 to 2.45)	0.55 (0.03 to 3.20)
	Uganda	2.36 (2.19 to 2.54)	3.06 (2.80 to 3.34)	0.96 (0.91 to 1.01)	–	–
	Zambia	0.72 (0.59 to 0.85)	0.88 (0.70 to 1.07)	0.40 (0.37 to 0.43)	–	–
	Zimbabwe	2.15 (1.76 to 2.62)	2.93 (2.35 to 3.68)	0.61 (0.51 to 0.68)	–	–
MCV1 immunization	Angola	0.26 (-5.07 to 5.22)	0.26 (-4.43 to 5.88)	0.26 (-6.34 to 3.92)	9.03 (3.09 to 18.37)	10.28 (4.11 to 19.68)
	Benin	0.91 (0.63 to 1.18)	0.97 (0.66 to 1.29)	0.77 (0.56 to 0.95)	1.37 (0.89 to 1.96)	2.58 (2.10 to 3.17)
	Burkina Faso	4.23 (3.90 to 4.57)	6.33 (5.80 to 6.86)	0.15 (0.12 to 0.19)	–	–
	Burundi	-0.14 (-0.51 to 0.17)	-0.14 (-0.41 to 0.18)	-0.16 (-0.71 to 0.15)	0.13 (0.02 to 0.29)	0.42 (0.02 to 1.16)
	Cameroon	0.53 (0.13 to 1.01)	0.55 (0.13 to 1.08)	0.49 (0.13 to 0.87)	2.61 (1.87 to 3.40)	3.83 (3.09 to 4.63)
	Chad	6.69 (5.33 to 7.85)	7.70 (5.93 to 9.23)	4.68 (4.13 to 5.07)	2.54 (1.39 to 4.03)	3.80 (2.65 to 5.31)
	Comoros	1.70 (-1.82 to 4.32)	1.98 (-1.56 to 5.93)	1.10 (-2.34 to 1.37)	1.59 (0.07 to 6.30)	1.32 (0.12 to 7.19)
	Congo Brazzaville	-2.06 (-3.58 to -0.84)	-1.88 (-3.15 to -0.81)	-2.41 (-4.45 to -0.91)	9.53 (6.41 to 13.44)	10.80 (7.66 to 14.73)
	Congo Democratic Republic	4.48 (3.73 to 5.15)	5.64 (4.53 to 6.65)	2.22 (2.14 to 2.32)	0.56 (0.40 to 0.71)	0.52 (0.12 to 1.12)
	Cote d'Ivoire	1.84 (-2.54 to 5.78)	2.07 (-2.24 to 7.94)	1.38 (-3.14 to 1.92)	2.07 (0.14 to 13.70)	2.60 (0.18 to 12.81)
	Egypt	-0.74 (-0.98 to -0.53)	-0.59 (-0.74 to -0.44)	-1.06 (-1.46 to -0.71)	0.81 (0.23 to 1.47)	1.99 (1.40 to 2.66)
	Eswatini	0.54 (-0.50 to 1.16)	0.70 (-0.43 to 1.69)	0.19 (-0.63 to 0.28)	0.77 (0.25 to 7.47)	0.55 (0.05 to 7.12)
	Ethiopia	5.39 (4.99 to 5.89)	6.30 (5.75 to 6.99)	3.60 (3.45 to 3.73)	1.51 (1.05 to 1.96)	2.75 (2.29 to 3.20)
	Gabon	4.40 (3.43 to 5.20)	5.03 (3.76 to 6.15)	3.14 (2.72 to 3.42)	2.11 (0.95 to 3.80)	3.36 (2.19 to 5.06)
	Gambia	0.65 (0.16 to 1.11)	0.75 (0.16 to 1.39)	0.44 (0.14 to 0.58)	–	0.12 (0.01 to 0.49)
	Ghana	0.82 (0.51 to 1.14)	0.95 (0.56 to 1.41)	0.54 (0.40 to 0.61)	–	0.10 (0.01 to 0.42)
	Guinea	-2.85 (-3.79 to -2.11)	-2.65 (-3.47 to -2.00)	-3.25 (-4.43 to -2.34)	14.05 (12.47 to 16.34)	15.37 (13.78 to 17.67)
	Kenya	1.86 (1.60 to 2.08)	2.40 (2.05 to 2.75)	0.77 (0.72 to 0.81)	–	–
	Lesotho	0.43 (-0.20 to 0.94)	0.46 (-0.20 to 1.06)	0.39 (-0.21 to 0.70)	0.61 (0.04 to 1.83)	1.53 (0.73 to 2.93)
	Liberia	1.87 (1.24 to 2.44)	2.10 (1.33 to 2.83)	1.43 (1.05 to 1.70)	1.14 (0.38 to 1.98)	2.36 (1.59 to 3.20)
	Madagascar	2.08 (1.51 to 2.59)	2.60 (1.74 to 3.43)	1.05 (0.90 to 1.11)	0.42 (0.17 to 0.44)	0.25 (0.02 to 1.25)
	Malawi	0.94 (0.83 to 1.04)	1.16 (1.00 to 1.30)	0.51 (0.48 to 0.53)	–	–
	Mali	2.59 (2.37 to 2.84)	3.04 (2.73 to 3.39)	1.69 (1.62 to 1.76)	0.21 (0.01 to 0.50)	1.39 (1.08 to 1.71)
	Morocco	0.40 (-0.83 to 0.63)	0.51 (-0.69 to 0.91)	0.14 (-1.12 to 0.22)	1.44 (0.41 to 8.63)	1.56 (0.31 to 9.80)
	Mozambique	0.83 (0.43 to 1.18)	0.92 (0.46 to 1.35)	0.67 (0.39 to 0.83)	0.28 (0.02 to 1.08)	1.23 (0.67 to 2.08)
	Namibia	0.71 (0.39 to 0.96)	0.79 (0.42 to 1.13)	0.54 (0.34 to 0.64)	0.10 (0.04 to 0.61)	0.59 (0.17 to 1.16)
	Niger	6.30 (5.58 to 7.09)	8.71 (7.44 to 9.99)	1.72 (1.45 to 1.97)	–	–
	Nigeria	4.16 (3.73 to 4.59)	4.66 (4.12 to 5.21)	3.16 (2.94 to 3.38)	3.03 (2.53 to 3.60)	4.29 (3.79 to 4.86)
	Rwanda	1.16 (1.02 to 1.29)	1.49 (1.27 to 1.70)	0.50 (0.46 to 0.53)	–	–
	Sao Tome and Principe	1.16 (-1.39 to 2.81)	1.49 (-1.18 to 4.12)	0.47 (-1.81 to 0.65)	1.15 (0.10 to 8.76)	1.11 (0.12 to 6.81)
	Senegal	1.85 (1.68 to 2.10)	2.35 (2.09 to 2.71)	0.87 (0.84 to 0.90)	–	–
	Sierra Leone	2.04 (1.52 to 2.61)	2.38 (1.72 to 3.15)	1.34 (1.13 to 1.54)	0.15 (0.01 to 0.48)	1.14 (0.68 to 1.58)
	South Africa	1.28 (-3.26 to 4.86)	1.40 (-2.55 to 6.15)	1.07 (-4.62 to 2.43)	2.01 (0.14 to 9.69)	2.64 (0.41 to 10.88)
	Tanzania	0.57 (0.30 to 0.82)	0.63 (0.31 to 0.94)	0.45 (0.27 to 0.56)	–	0.48 (0.13 to 0.89)
	Togo	1.75 (-0.84 to 4.22)	2.11 (-0.77 to 5.83)	1.04 (-0.99 to 1.25)	1.07 (0.04 to 4.76)	0.93 (0.04 to 4.93)
	Uganda	2.60 (2.32 to 2.82)	3.14 (2.75 to 3.45)	1.52 (1.44 to 1.58)	–	0.71 (0.46 to 1.02)
	Zambia	0.82 (0.65 to 1.00)	0.93 (0.72 to 1.17)	0.60 (0.51 to 0.66)	–	0.42 (0.20 to 0.67)
	Zimbabwe	2.66 (2.24 to 3.15)	3.46 (2.83 to 4.24)	1.05 (0.96 to 1.15)	–	0.06 (0.04 to 0.08)
Polio3 immunization	Angola	-0.02 (-2.84 to 3.95)	-0.02 (-2.36 to 4.81)	-0.02 (-3.80 to 2.18)	3.74 (0.74 to 9.11)	4.85 (1.01 to 10.34)
	Benin	0.51 (0.24 to 0.71)	0.53 (0.25 to 0.77)	0.45 (0.23 to 0.61)	0.96 (0.60 to 1.49)	2.16 (1.80 to 2.69)
	Burkina Faso	3.16 (2.88 to 3.49)	4.70 (4.27 to 5.23)	0.13 (0.10 to 0.17)	–	–
	Burundi	0.05 (-0.21 to 0.29)	0.05 (-0.18 to 0.35)	0.04 (-0.28 to 0.18)	–	0.02 (0.02 to 0.35)
	Cameroon	-0.04 (-0.35 to 0.31)	-0.04 (-0.34 to 0.33)	-0.04 (-0.38 to 0.29)	1.86 (1.15 to 2.47)	3.07 (2.35 to 3.68)
	Chad	6.35 (5.56 to 7.02)	8.56 (7.33 to 9.62)	2.07 (1.92 to 2.21)	–	0.04 (0.00 to 0.08)
	Comoros	1.22 (-1.66 to 3.01)	1.43 (-1.31 to 4.19)	0.72 (-2.37 to 0.93)	1.24 (0.11 to 7.46)	0.79 (0.05 to 7.55)
	Congo Brazzaville	1.51 (1.08 to 1.98)	1.87 (1.27 to 2.60)	0.78 (0.70 to 0.85)	–	0.11 (0.01 to 0.49)
	Congo Democratic Republic	1.73 (1.25 to 2.30)	2.09 (1.43 to 2.91)	1.00 (0.87 to 1.08)	0.05 (0.05 to 0.05)	0.27 (0.01 to 0.77)
	Cote d'Ivoire	1.04 (-2.62 to 3.51)	1.15 (-2.12 to 4.69)	0.82 (-3.63 to 1.26)	1.95 (0.11 to 10.59)	2.03 (0.19 to 9.39)
	Egypt	-1.85 (-2.26 to -1.37)	-0.95 (-1.11 to -0.77)	-3.64 (-4.57 to -2.57)	3.51 (2.14 to 4.71)	4.70 (3.32 to 5.90)

Indicators	Country	Average Annual Rate of Change (95% CrI)			Additional AARC Required to Reach Targets (95% CrI)	
		2000–2030	2000–2020	2020–2030	UHC2030	IA2030
	Eswatini	0.35 (-1.18 to 0.80)	0.45 (-0.81 to 1.17)	0.13 (-1.90 to 0.22)	2.30 (0.81 to 8.08)	0.63 (0.04 to 7.57)
	Ethiopia	3.42 (3.13 to 3.67)	4.06 (3.66 to 4.42)	2.12 (2.04 to 2.20)	0.24 (0.03 to 0.58)	1.44 (1.13 to 1.79)
	Gabon	1.70 (1.48 to 2.00)	2.12 (1.77 to 2.57)	0.87 (0.80 to 0.93)	–	0.08 (0.00 to 0.44)
	Gambia	-0.33 (-0.73 to -0.02)	-0.27 (-0.47 to -0.02)	-0.44 (-1.26 to -0.02)	0.20 (0.01 to 0.46)	0.47 (0.04 to 1.42)
	Ghana	0.50 (0.28 to 0.68)	0.60 (0.31 to 0.87)	0.29 (0.22 to 0.33)	–	–
	Guinea	-1.78 (-2.53 to -1.21)	-1.64 (-2.27 to -1.14)	-2.06 (-3.05 to -1.36)	8.36 (7.05 to 10.18)	9.62 (8.30 to 11.45)
	Kenya	1.06 (0.89 to 1.26)	1.36 (1.11 to 1.66)	0.45 (0.42 to 0.48)	–	–
	Lesotho	0.54 (0.15 to 0.79)	0.64 (0.15 to 0.98)	0.35 (0.13 to 0.41)	–	0.15 (0.02 to 0.57)
	Liberia	1.21 (0.70 to 1.63)	1.38 (0.75 to 1.94)	0.85 (0.58 to 1.00)	0.13 (0.02 to 0.44)	0.77 (0.34 to 1.39)
	Madagascar	1.93 (1.41 to 2.35)	2.51 (1.69 to 3.17)	0.80 (0.66 to 0.90)	–	0.21 (0.04 to 0.46)
	Malawi	0.61 (0.54 to 0.67)	0.77 (0.68 to 0.86)	0.29 (0.27 to 0.30)	–	–
	Mali	1.48 (1.28 to 1.67)	1.72 (1.47 to 1.98)	0.98 (0.92 to 1.05)	–	0.70 (0.46 to 0.99)
	Morocco	0.34 (-0.74 to 0.56)	0.43 (-0.60 to 0.81)	0.13 (-1.01 to 0.20)	1.51 (0.10 to 12.37)	0.72 (0.09 to 10.09)
	Mozambique	0.61 (0.35 to 0.85)	0.69 (0.38 to 1.04)	0.44 (0.30 to 0.51)	–	0.17 (0.01 to 0.55)
	Namibia	0.61 (0.39 to 0.78)	0.75 (0.45 to 1.00)	0.33 (0.28 to 0.36)	–	–
	Niger	4.29 (3.67 to 4.89)	5.98 (4.96 to 6.95)	1.01 (0.86 to 1.17)	–	–
	Nigeria	2.51 (2.21 to 2.90)	2.81 (2.44 to 3.29)	1.94 (1.77 to 2.12)	2.02 (1.62 to 2.41)	3.25 (2.85 to 3.64)
	Rwanda	0.58 (0.41 to 0.71)	0.69 (0.47 to 0.87)	0.38 (0.31 to 0.41)	–	0.04 (0.02 to 0.13)
	Sao Tome and Principe	0.80 (-1.02 to 1.83)	0.99 (-0.84 to 2.61)	0.30 (-1.39 to 0.47)	1.56 (0.42 to 4.33)	0.79 (0.03 to 5.20)
	Senegal	1.01 (0.84 to 1.19)	1.22 (0.98 to 1.48)	0.58 (0.54 to 0.63)	–	0.04 (0.01 to 0.08)
	Sierra Leone	1.26 (0.88 to 1.71)	1.47 (0.99 to 2.06)	0.85 (0.68 to 1.00)	–	0.57 (0.22 to 0.96)
	South Africa	0.71 (-0.53 to 3.27)	0.82 (-0.45 to 4.59)	0.46 (-0.71 to 0.77)	0.32 (0.01 to 3.16)	0.44 (0.02 to 1.91)
	Tanzania	0.56 (0.37 to 0.76)	0.65 (0.42 to 0.93)	0.39 (0.29 to 0.45)	–	0.08 (0.01 to 0.25)
	Togo	1.31 (-0.86 to 3.81)	1.56 (-0.76 to 5.32)	0.81 (-1.07 to 1.01)	0.63 (0.08 to 5.03)	0.97 (0.05 to 3.98)
	Uganda	1.63 (1.46 to 1.80)	2.03 (1.78 to 2.28)	0.83 (0.79 to 0.86)	–	0.06 (0.01 to 0.12)
	Zambia	0.34 (0.21 to 0.46)	0.38 (0.23 to 0.52)	0.26 (0.18 to 0.31)	–	0.03 (0.00 to 0.16)
	Zimbabwe	1.57 (1.25 to 1.92)	2.01 (1.56 to 2.54)	0.70 (0.64 to 0.75)	–	–
DPT3 immunization	Angola	0.21 (-3.81 to 4.46)	0.21 (-3.13 to 5.45)	0.20 (-5.15 to 2.50)	4.52 (1.22 to 12.25)	5.73 (1.33 to 13.51)
	Benin	0.02 (-0.17 to 0.21)	0.02 (-0.17 to 0.22)	0.02 (-0.18 to 0.20)	0.38 (0.07 to 0.78)	1.55 (1.20 to 1.97)
	Burkina Faso	2.59 (2.35 to 2.88)	3.90 (3.52 to 4.34)	0.03 (0.02 to 0.05)	–	–
	Burundi	0.23 (0.08 to 0.45)	0.29 (0.09 to 0.62)	0.11 (0.06 to 0.13)	–	–
	Cameroon	-0.05 (-0.33 to 0.18)	-0.05 (-0.31 to 0.18)	-0.05 (-0.37 to 0.17)	0.34 (0.03 to 0.87)	1.51 (1.10 to 2.06)
	Chad	2.61 (1.96 to 3.12)	3.05 (2.19 to 3.75)	1.74 (1.48 to 1.92)	0.38 (0.04 to 1.15)	1.54 (0.95 to 2.35)
	Comoros	1.13 (-1.03 to 3.26)	1.36 (-0.88 to 4.61)	0.63 (-1.36 to 0.82)	0.80 (0.01 to 2.82)	0.95 (0.04 to 3.98)
	Congo Brazzaville	-1.45 (-2.53 to -0.42)	-1.21 (-1.93 to -0.40)	-1.93 (-3.74 to -0.48)	4.01 (1.38 to 6.96)	5.22 (2.57 to 8.18)
	Congo Democratic Republic	3.52 (2.88 to 4.10)	4.88 (3.88 to 5.82)	0.86 (0.75 to 0.96)	–	–
	Cote d'Ivoire	1.16 (-0.98 to 2.98)	1.42 (-0.85 to 4.24)	0.59 (-1.25 to 0.73)	1.10 (0.02 to 3.77)	0.67 (0.06 to 4.04)
	Egypt	0.14 (0.11 to 0.17)	0.18 (0.13 to 0.23)	0.06 (0.05 to 0.06)	–	–
	Eswatini	0.30 (-0.14 to 0.70)	0.40 (-0.13 to 1.05)	0.08 (-0.17 to 0.15)	0.35 (0.35 to 0.35)	0.78 (0.29 to 1.47)
	Ethiopia	2.54 (2.30 to 2.73)	2.97 (2.66 to 3.23)	1.67 (1.59 to 1.75)	0.23 (0.01 to 0.56)	1.42 (1.19 to 1.77)
	Gabon	1.48 (0.85 to 2.14)	1.57 (0.88 to 2.35)	1.29 (0.79 to 1.70)	3.56 (2.11 to 5.01)	4.80 (3.34 to 6.27)
	Gambia	-0.02 (-0.27 to 0.21)	-0.02 (-0.20 to 0.24)	-0.02 (-0.40 to 0.14)	–	0.07 (0.06 to 0.62)
	Ghana	0.56 (0.36 to 0.72)	0.71 (0.43 to 0.98)	0.26 (0.23 to 0.30)	–	–
	Guinea	-0.57 (-1.14 to -0.06)	-0.55 (-1.05 to -0.06)	-0.62 (-1.32 to -0.06)	4.01 (2.99 to 5.33)	5.23 (4.21 to 6.56)
	Kenya	1.06 (0.90 to 1.23)	1.45 (1.19 to 1.73)	0.28 (0.25 to 0.31)	–	–
	Lesotho	0.14 (-0.34 to 0.48)	0.14 (-0.31 to 0.56)	0.13 (-0.40 to 0.33)	0.09 (0.03 to 0.44)	0.36 (0.04 to 1.26)
	Liberia	1.43 (1.02 to 1.89)	1.71 (1.15 to 2.35)	0.88 (0.73 to 1.00)	–	0.27 (0.04 to 0.63)
	Madagascar	1.46 (0.98 to 1.92)	1.84 (1.14 to 2.58)	0.69 (0.60 to 0.76)	–	0.19 (0.03 to 0.55)
	Malawi	0.50 (0.43 to 0.57)	0.63 (0.53 to 0.73)	0.25 (0.23 to 0.26)	–	–
	Mali	1.50 (1.32 to 1.69)	1.77 (1.54 to 2.05)	0.94 (0.89 to 0.99)	–	0.38 (0.16 to 0.63)
	Morocco	0.20 (-0.30 to 0.31)	0.27 (-0.24 to 0.46)	0.05 (-0.40 to 0.10)	0.44 (0.12 to 0.71)	1.29 (0.12 to 1.88)
	Mozambique	0.88 (0.69 to 1.08)	1.08 (0.81 to 1.38)	0.49 (0.45 to 0.52)	–	0.24 (0.24 to 0.24)
	Namibia	0.11 (-0.17 to 0.34)	0.11 (-0.16 to 0.39)	0.10 (-0.19 to 0.26)	–	0.29 (0.03 to 0.98)
	Niger	3.31 (2.83 to 3.78)	4.71 (3.98 to 5.47)	0.56 (0.45 to 0.68)	–	–
	Nigeria	2.80 (2.57 to 3.04)	3.37 (3.05 to 3.72)	1.67 (1.60 to 1.72)	0.01 (0.01 to 0.01)	0.88 (0.70 to 1.09)
	Rwanda	0.46 (0.39 to 0.53)	0.61 (0.49 to 0.72)	0.18 (0.15 to 0.19)	–	–
	Sao Tome and Principe	0.60 (-0.60 to 1.69)	0.78 (-0.49 to 2.49)	0.19 (-0.84 to 0.31)	0.99 (0.07 to 3.61)	0.89 (0.14 to 4.57)
	Senegal	0.96 (0.79 to 1.13)	1.27 (1.03 to 1.54)	0.34 (0.32 to 0.36)	–	–
	Sierra Leone	1.98 (1.59 to 2.37)	2.63 (2.05 to 3.22)	0.66 (0.62 to 0.72)	–	–
	South Africa	0.82 (-1.81 to 4.40)	0.90 (-1.35 to 5.94)	0.64 (-2.72 to 1.37)	0.77 (0.05 to 5.77)	1.31 (0.08 to 6.07)
	Tanzania	0.26 (0.06 to 0.40)	0.28 (0.06 to 0.47)	0.20 (0.06 to 0.28)	–	0.06 (0.01 to 0.28)
	Togo	0.98 (-0.23 to 2.77)	1.25 (-0.21 to 4.00)	0.39 (-0.26 to 0.48)	0.62 (0.28 to 2.03)	0.36 (0.04 to 2.69)
	Uganda	1.73 (1.60 to 1.87)	2.27 (2.07 to 2.48)	0.66 (0.62 to 0.70)	–	–
	Zambia	0.55 (0.42 to 0.69)	0.65 (0.48 to 0.84)	0.35 (0.30 to 0.39)	–	–
	Zimbabwe	1.78 (1.43 to 2.17)	2.30 (1.76 to 2.89)	0.74 (0.68 to 0.80)	–	0.06 (0.06 to 0.06)

Notes: AARC = Average Annual Rate of Change. The additional AARC required to reach global targets was calculated as the difference between the projected AARC for 2020–2030 and the AARC needed to achieve the target coverage by 2030.

Table J. AARC and Additional values required to reach 2030 targets, by wealth quintile

Indicators	Country	Wealth Quintile	Average Annual Rate of Change (95% CrI)			Added AARC Needed to Achieve Targets (95% CrI)	
			2000–2030	2000–2020	2020–2030	UHC2030	IA2030
Full immunization	Angola	Poorest	-0.56 (-6.02 to 5.19)	-0.55 (-4.78 to 6.27)	-0.59 (-8.43 to 3.01)	7.34 (0.54 to 19.25)	8.58 (1.66 to 20.34)
		Poorer	-0.01 (-3.93 to 3.98)	-0.01 (-2.70 to 5.13)	-0.01 (-6.26 to 1.71)	2.65 (0.08 to 10.75)	3.57 (0.41 to 11.99)
		Middle	0.17 (-2.34 to 2.94)	0.18 (-1.48 to 3.97)	0.16 (-4.05 to 0.93)	1.53 (0.12 to 5.93)	1.40 (0.05 to 6.77)
		Richer	-0.02 (-1.32 to 1.43)	-0.02 (-0.75 to 1.93)	-0.02 (-2.43 to 0.46)	0.78 (0.01 to 5.83)	0.80 (0.03 to 3.99)
		Richest	-0.04 (-0.58 to 0.49)	-0.03 (-0.29 to 0.66)	-0.04 (-1.15 to 0.15)	1.91 (0.01 to 3.81)	0.41 (0.01 to 4.98)
	Benin	Poorest	-0.39 (-0.74 to -0.07)	-0.37 (-0.67 to -0.07)	-0.44 (-0.88 to -0.08)	1.40 (0.74 to 2.29)	2.60 (1.93 to 3.49)
		Poorer	-0.09 (-0.31 to 0.11)	-0.09 (-0.29 to 0.11)	-0.10 (-0.36 to 0.10)	0.13 (0.00 to 0.24)	0.80 (0.40 to 1.30)
		Middle	0.03 (-0.08 to 0.15)	0.03 (-0.08 to 0.16)	0.03 (-0.09 to 0.13)	–	0.09 (0.00 to 0.39)
		Richer	-0.03 (-0.13 to 0.04)	-0.03 (-0.12 to 0.04)	-0.03 (-0.15 to 0.04)	–	–
		Richest	-0.03 (-0.07 to 0.00)	-0.03 (-0.06 to 0.00)	-0.03 (-0.09 to 0.00)	–	–
	Burkina Faso	Poorest	2.19 (1.80 to 2.62)	3.27 (2.68 to 3.94)	0.05 (0.03 to 0.09)	–	–
		Poorer	1.70 (1.44 to 2.05)	2.55 (2.14 to 3.09)	0.03 (0.02 to 0.06)	–	–
		Middle	1.43 (1.22 to 1.73)	2.14 (1.81 to 2.59)	0.02 (0.01 to 0.04)	–	–
		Richer	0.89 (0.73 to 1.12)	1.33 (1.09 to 1.68)	0.02 (0.01 to 0.03)	–	–
		Richest	0.41 (0.32 to 0.55)	0.61 (0.47 to 0.82)	0.01 (0.00 to 0.01)	–	–
	Burundi	Poorest	-0.19 (-0.61 to 0.06)	-0.14 (-0.33 to 0.07)	-0.26 (-1.19 to 0.05)	0.68 (0.03 to 1.31)	0.31 (0.04 to 1.85)
		Poorer	-0.09 (-0.36 to 0.06)	-0.07 (-0.19 to 0.07)	-0.11 (-0.67 to 0.05)	–	0.16 (0.01 to 0.77)
		Middle	-0.04 (-0.22 to 0.05)	-0.04 (-0.12 to 0.06)	-0.05 (-0.43 to 0.04)	–	–
		Richer	-0.04 (-0.18 to 0.03)	-0.03 (-0.09 to 0.03)	-0.05 (-0.39 to 0.02)	–	–
		Richest	-0.02 (-0.09 to 0.01)	-0.02 (-0.05 to 0.01)	-0.03 (-0.18 to 0.01)	–	–
	Cameroon	Poorest	-0.04 (-0.45 to 0.26)	-0.04 (-0.43 to 0.27)	-0.04 (-0.50 to 0.24)	1.01 (0.24 to 1.84)	2.18 (1.39 to 3.03)
		Poorer	0.11 (-0.14 to 0.31)	0.12 (-0.13 to 0.33)	0.11 (-0.15 to 0.26)	0.04 (0.02 to 0.06)	0.57 (0.21 to 1.03)
		Middle	0.17 (0.02 to 0.30)	0.18 (0.02 to 0.34)	0.15 (0.02 to 0.22)	–	0.06 (0.00 to 0.32)
		Richer	0.04 (-0.05 to 0.12)	0.04 (-0.05 to 0.14)	0.04 (-0.06 to 0.10)	–	–
		Richest	-0.00 (-0.04 to 0.04)	-0.00 (-0.04 to 0.04)	-0.00 (-0.04 to 0.03)	–	–
	Chad	Poorest	3.70 (2.97 to 4.61)	4.31 (3.39 to 5.60)	2.47 (2.15 to 2.74)	0.91 (0.15 to 1.88)	2.13 (1.35 to 3.11)
		Poorer	3.65 (2.94 to 4.39)	4.40 (3.48 to 5.51)	2.12 (1.93 to 2.28)	0.17 (0.01 to 0.75)	1.10 (0.49 to 1.79)
		Middle	3.73 (3.04 to 4.37)	4.65 (3.66 to 5.60)	1.91 (1.76 to 2.02)	0.08 (0.01 to 0.15)	0.52 (0.12 to 1.09)
		Richer	2.82 (2.28 to 3.44)	3.51 (2.74 to 4.40)	1.48 (1.37 to 1.59)	–	0.34 (0.02 to 0.86)
		Richest	1.74 (1.40 to 2.14)	2.17 (1.68 to 2.76)	0.87 (0.77 to 0.97)	–	0.07 (0.00 to 0.54)
	Comoros	Poorest	1.18 (-1.08 to 3.44)	1.42 (-0.91 to 4.81)	0.60 (-1.40 to 0.93)	0.82 (0.02 to 6.19)	1.03 (0.01 to 6.72)
		Poorer	1.05 (-0.67 to 2.94)	1.28 (-0.57 to 4.25)	0.47 (-0.87 to 0.63)	0.71 (0.06 to 10.64)	0.56 (0.02 to 5.73)
		Middle	1.01 (-0.38 to 2.79)	1.29 (-0.34 to 4.06)	0.38 (-0.48 to 0.54)	0.30 (0.03 to 7.78)	0.50 (0.06 to 6.33)
		Richer	0.63 (-0.47 to 2.13)	0.78 (-0.39 to 3.10)	0.27 (-0.62 to 0.42)	1.05 (0.09 to 7.06)	0.47 (0.04 to 7.56)
		Richest	0.31 (-0.31 to 1.18)	0.39 (-0.25 to 1.71)	0.13 (-0.43 to 0.25)	2.10 (0.22 to 3.71)	0.21 (0.01 to 4.89)
	Congo Brazzaville	Poorest	1.04 (0.58 to 1.63)	1.34 (0.69 to 2.29)	0.39 (0.31 to 0.47)	–	–
		Poorer	0.59 (0.37 to 0.90)	0.79 (0.45 to 1.29)	0.19 (0.14 to 0.23)	–	–
		Middle	0.37 (0.22 to 0.55)	0.50 (0.28 to 0.79)	0.09 (0.06 to 0.13)	–	–
		Richer	0.15 (0.08 to 0.24)	0.20 (0.10 to 0.34)	0.04 (0.03 to 0.06)	–	–
		Richest	0.04 (0.02 to 0.08)	0.06 (0.03 to 0.11)	0.01 (0.01 to 0.02)	–	–
	Congo Democratic Republic	Poorest	2.80 (2.21 to 3.53)	3.66 (2.80 to 4.81)	1.06 (0.96 to 1.19)	–	0.11 (0.11 to 0.11)
		Poorer	2.32 (1.86 to 2.87)	3.12 (2.40 to 3.98)	0.73 (0.64 to 0.79)	–	–
		Middle	2.09 (1.62 to 2.57)	2.88 (2.15 to 3.61)	0.54 (0.46 to 0.61)	–	–
		Richer	1.37 (1.02 to 1.75)	1.88 (1.34 to 2.45)	0.37 (0.31 to 0.42)	–	–
		Richest	0.67 (0.48 to 0.89)	0.92 (0.63 to 1.26)	0.19 (0.15 to 0.22)	–	–
	Cote d'Ivoire	Poorest	1.54 (-1.55 to 4.03)	1.88 (-1.33 to 5.73)	0.83 (-2.01 to 1.11)	1.40 (0.05 to 9.62)	1.26 (0.04 to 8.47)
		Poorer	1.23 (-0.90 to 3.24)	1.54 (-0.77 to 4.67)	0.56 (-1.16 to 0.69)	1.00 (0.03 to 5.98)	0.64 (0.02 to 6.28)
		Middle	1.03 (-0.51 to 2.77)	1.30 (-0.44 to 4.06)	0.39 (-0.66 to 0.50)	0.50 (0.12 to 3.14)	0.56 (0.01 to 4.33)
		Richer	0.57 (-0.44 to 1.73)	0.72 (-0.35 to 2.48)	0.23 (-0.63 to 0.33)	0.97 (0.12 to 2.17)	0.68 (0.04 to 3.35)
		Richest	0.23 (-0.23 to 0.84)	0.30 (-0.18 to 1.19)	0.10 (-0.32 to 0.16)	0.56 (0.56 to 0.56)	0.65 (0.54 to 1.73)
	Egypt	Poorest	-0.02 (-0.13 to 0.04)	-0.02 (-0.11 to 0.04)	-0.02 (-0.18 to 0.03)	–	–
		Poorer	-0.00 (-0.07 to 0.04)	-0.00 (-0.06 to 0.05)	-0.00 (-0.10 to 0.03)	–	–
		Middle	0.01 (-0.03 to 0.04)	0.01 (-0.03 to 0.05)	0.01 (-0.04 to 0.03)	–	–
		Richer	-0.00 (-0.04 to 0.02)	-0.00 (-0.03 to 0.03)	-0.00 (-0.05 to 0.02)	–	–
		Richest	-0.00 (-0.02 to 0.01)	-0.00 (-0.02 to 0.01)	-0.00 (-0.04 to 0.01)	–	–
	Eswatini	Poorest	0.18 (-0.15 to 0.45)	0.24 (-0.13 to 0.64)	0.05 (-0.20 to 0.12)	0.34 (0.34 to 0.34)	0.51 (0.11 to 1.52)
		Poorer	0.18 (-0.10 to 0.43)	0.25 (-0.09 to 0.63)	0.04 (-0.13 to 0.09)	–	0.67 (0.33 to 1.02)
		Middle	0.20 (-0.04 to 0.48)	0.27 (-0.04 to 0.71)	0.04 (-0.04 to 0.09)	–	0.63 (0.63 to 0.63)
		Richer	0.15 (-0.05 to 0.40)	0.21 (-0.05 to 0.58)	0.04 (-0.06 to 0.08)	0.03 (0.03 to 0.03)	0.66 (0.12 to 1.20)
		Richest	0.09 (-0.06 to 0.30)	0.12 (-0.05 to 0.43)	0.02 (-0.07 to 0.06)	–	0.56 (0.56 to 0.56)
	Ethiopia	Poorest	2.16 (1.81 to 2.47)	2.48 (2.02 to 2.89)	1.54 (1.38 to 1.67)	0.63 (0.24 to 1.20)	1.84 (1.45 to 2.41)
		Poorer	2.13 (1.87 to 2.43)	2.51 (2.16 to 2.92)	1.37 (1.26 to 1.45)	0.06 (0.00 to 0.25)	0.92 (0.62 to 1.35)
		Middle	2.22 (2.02 to 2.47)	2.69 (2.43 to 3.07)	1.27 (1.21 to 1.34)	–	0.41 (0.17 to 0.70)
		Richer	1.60 (1.39 to 1.87)	1.93 (1.64 to 2.30)	0.96 (0.89 to 1.03)	–	0.27 (0.05 to 0.54)
		Richest	0.91 (0.72 to 1.08)	1.10 (0.84 to 1.32)	0.54 (0.49 to 0.59)	–	0.07 (0.03 to 0.11)
	Gabon	Poorest	0.46 (0.17 to 0.69)	0.53 (0.18 to 0.83)	0.31 (0.15 to 0.38)	–	0.10 (0.00 to 0.44)
		Poorer	0.38 (0.21 to 0.53)	0.46 (0.23 to 0.66)	0.23 (0.17 to 0.27)	–	–
		Middle	0.34 (0.21 to 0.47)	0.42 (0.24 to 0.59)	0.18 (0.14 to 0.22)	–	–
		Richer	0.19 (0.09 to 0.27)	0.22 (0.09 to 0.35)	0.11 (0.07 to 0.15)	–	–
		Richest	0.07 (0.04 to 0.14)	0.09 (0.04 to 0.17)	0.05 (0.03 to 0.07)	–	–
	Gambia	Poorest	0.07 (-0.04 to 0.35)	0.09 (-0.03 to 0.50)	0.04 (-0.05 to 0.07)	–	–
		Poorer	0.08 (-0.02 to 0.33)	0.10 (-0.02 to 0.47)	0.04 (-0.02 to 0.06)	–	–
		Middle	0.09 (-0.01 to 0.39)	0.11 (-0.01 to 0.56)	0.04 (-0.02 to 0.06)	–	–
		Richer	0.06 (-0.02 to 0.32)	0.08 (-0.02 to 0.46)	0.03 (-0.02 to 0.06)	–	–
		Richest	0.03 (-0.02 to 0.21)	0.04 (-0.01 to 0.29)	0.02 (-0.02 to 0.04)	–	–
	Ghana	Poorest	0.56 (0.35 to 0.76)	0.74 (0.43 to 1.07)	0.18 (0.13 to 0.24)	–	–
		Poorer	0.45 (0.32 to 0.59)	0.60 (0.41 to 0.84)	0.13 (0.09 to 0.16)	–	–
		Middle	0.40 (0.27 to 0.55)	0.55 (0.35 to 0.77)	0.10 (0.07 to 0.12)	–	–
		Richer	0.24 (0.15 to 0.36)	0.33 (0.19 to 0.52)	0.07 (0.04 to 0.09)	–	–
		Richest	0.11 (0.06 to 0.19)	0.15 (0.08 to 0.27)	0.03 (0.02 to 0.05)	–	–
	Guinea	Poorest	-1.40 (-2.00 to -0.78)	-1.24 (-1.68 to -0.72)	-1.73 (-2.62 to -0.90)	4.88 (3.34 to 6.56)	6.10 (4.56 to 7.79)

Indicators	Country	Wealth Quintile	Average Annual Rate of Change (95% CrI)			Added AARC Needed to Achieve Targets (95% CrI)	
			2000–2030	2000–2020	2020–2030	UHC2030	IA2030
		Poorer	-0.78 (-1.28 to -0.33)	-0.70 (-1.07 to -0.31)	-0.95 (-1.68 to -0.36)	2.36 (1.40 to 3.56)	3.56 (2.59 to 4.77)
		Middle	-0.42 (-0.82 to -0.06)	-0.38 (-0.70 to -0.06)	-0.48 (-1.04 to -0.07)	0.91 (0.21 to 1.89)	2.09 (1.28 to 3.03)
		Richer	-0.46 (-0.75 to -0.17)	-0.40 (-0.62 to -0.16)	-0.57 (-1.02 to -0.18)	0.39 (0.03 to 1.10)	1.46 (0.89 to 2.27)
		Richest	-0.32 (-0.51 to -0.13)	-0.26 (-0.39 to -0.12)	-0.42 (-0.74 to -0.14)	–	0.41 (0.04 to 0.99)
	Kenya	Poorest	1.01 (0.80 to 1.28)	1.41 (1.08 to 1.81)	0.22 (0.18 to 0.27)	–	–
		Poorer	0.72 (0.56 to 0.87)	1.01 (0.77 to 1.25)	0.13 (0.11 to 0.15)	–	–
		Middle	0.56 (0.43 to 0.68)	0.79 (0.60 to 0.97)	0.08 (0.06 to 0.11)	–	–
		Richer	0.30 (0.22 to 0.40)	0.42 (0.32 to 0.57)	0.05 (0.04 to 0.07)	–	–
		Richest	0.12 (0.09 to 0.17)	0.17 (0.12 to 0.24)	0.02 (0.02 to 0.03)	–	–
	Lesotho	Poorest	0.25 (0.04 to 0.51)	0.31 (0.04 to 0.70)	0.12 (0.03 to 0.17)	–	–
		Poorer	0.22 (0.06 to 0.37)	0.27 (0.06 to 0.52)	0.09 (0.05 to 0.12)	–	–
		Middle	0.19 (0.07 to 0.34)	0.26 (0.07 to 0.47)	0.07 (0.05 to 0.10)	–	–
		Richer	0.11 (0.03 to 0.24)	0.15 (0.03 to 0.31)	0.05 (0.02 to 0.08)	–	–
		Richest	0.05 (0.01 to 0.12)	0.07 (0.01 to 0.16)	0.02 (0.01 to 0.04)	–	–
	Liberia	Poorest	2.33 (1.76 to 2.96)	3.19 (2.28 to 4.16)	0.66 (0.58 to 0.75)	–	–
		Poorer	1.82 (1.38 to 2.27)	2.53 (1.85 to 3.22)	0.41 (0.37 to 0.46)	–	–
		Middle	1.52 (1.12 to 1.88)	2.14 (1.52 to 2.69)	0.28 (0.24 to 0.33)	–	–
		Richer	0.92 (0.69 to 1.18)	1.29 (0.93 to 1.68)	0.18 (0.14 to 0.23)	–	–
		Richest	0.41 (0.27 to 0.57)	0.57 (0.37 to 0.81)	0.09 (0.06 to 0.11)	–	–
	Madagascar	Poorest	2.89 (2.14 to 3.46)	3.98 (2.77 to 4.97)	0.71 (0.49 to 0.98)	–	–
		Poorer	1.96 (1.48 to 2.35)	2.74 (1.97 to 3.41)	0.36 (0.24 to 0.52)	–	–
		Middle	1.36 (1.06 to 1.68)	1.94 (1.44 to 2.46)	0.20 (0.12 to 0.30)	–	–
		Richer	0.68 (0.51 to 0.85)	0.97 (0.71 to 1.24)	0.11 (0.06 to 0.16)	–	–
		Richest	0.24 (0.18 to 0.32)	0.35 (0.24 to 0.47)	0.04 (0.02 to 0.06)	–	–
	Malawi	Poorest	0.36 (0.31 to 0.42)	0.47 (0.40 to 0.57)	0.13 (0.11 to 0.15)	–	–
		Poorer	0.33 (0.29 to 0.38)	0.45 (0.38 to 0.52)	0.10 (0.09 to 0.12)	–	–
		Middle	0.34 (0.30 to 0.38)	0.47 (0.41 to 0.54)	0.09 (0.08 to 0.10)	–	–
		Richer	0.25 (0.21 to 0.29)	0.33 (0.27 to 0.39)	0.07 (0.06 to 0.09)	–	–
		Richest	0.13 (0.11 to 0.16)	0.18 (0.14 to 0.22)	0.04 (0.03 to 0.05)	–	–
	Mali	Poorest	1.36 (1.11 to 1.56)	1.59 (1.26 to 1.87)	0.91 (0.80 to 1.00)	0.08 (0.08 to 0.08)	0.61 (0.33 to 1.00)
		Poorer	1.22 (1.03 to 1.40)	1.47 (1.21 to 1.74)	0.72 (0.67 to 0.77)	–	0.07 (0.00 to 0.31)
		Middle	1.17 (0.99 to 1.30)	1.46 (1.19 to 1.64)	0.60 (0.57 to 0.64)	–	–
		Richer	0.74 (0.63 to 0.85)	0.90 (0.75 to 1.06)	0.41 (0.38 to 0.44)	–	–
		Richest	0.35 (0.28 to 0.44)	0.42 (0.33 to 0.55)	0.20 (0.18 to 0.23)	–	–
	Morocco	Poorest	0.13 (-0.10 to 0.24)	0.17 (-0.09 to 0.35)	0.03 (-0.12 to 0.08)	1.58 (1.21 to 1.96)	1.77 (0.10 to 3.14)
		Poorer	0.09 (-0.04 to 0.15)	0.12 (-0.04 to 0.22)	0.02 (-0.04 to 0.05)	0.72 (0.26 to 1.17)	1.43 (0.49 to 2.35)
		Middle	0.07 (-0.01 to 0.13)	0.09 (-0.01 to 0.19)	0.01 (-0.01 to 0.04)	0.45 (0.45 to 0.45)	0.64 (0.09 to 1.63)
		Richer	0.04 (-0.03 to 0.09)	0.05 (-0.02 to 0.14)	0.01 (-0.03 to 0.02)	0.56 (0.56 to 0.56)	0.66 (0.11 to 1.73)
		Richest	0.01 (-0.02 to 0.04)	0.02 (-0.01 to 0.06)	0.00 (-0.02 to 0.01)	–	0.63 (0.10 to 1.16)
	Mozambique	Poorest	0.52 (0.22 to 0.83)	0.59 (0.23 to 1.01)	0.38 (0.19 to 0.48)	0.48 (0.13 to 0.84)	0.24 (0.02 to 1.16)
		Poorer	0.42 (0.20 to 0.61)	0.49 (0.21 to 0.78)	0.27 (0.17 to 0.31)	–	0.11 (0.01 to 0.47)
		Middle	0.34 (0.23 to 0.49)	0.42 (0.26 to 0.64)	0.20 (0.17 to 0.22)	–	–
		Richer	0.17 (0.08 to 0.27)	0.20 (0.08 to 0.34)	0.11 (0.07 to 0.13)	–	–
		Richest	0.06 (0.02 to 0.10)	0.07 (0.02 to 0.13)	0.04 (0.02 to 0.06)	–	–
	Namibia	Poorest	0.27 (0.08 to 0.44)	0.32 (0.08 to 0.55)	0.17 (0.07 to 0.21)	–	0.28 (0.28 to 0.28)
		Poorer	0.27 (0.14 to 0.38)	0.33 (0.15 to 0.50)	0.15 (0.11 to 0.18)	–	–
		Middle	0.29 (0.16 to 0.39)	0.36 (0.19 to 0.52)	0.15 (0.11 to 0.17)	–	–
		Richer	0.19 (0.09 to 0.28)	0.24 (0.10 to 0.37)	0.11 (0.07 to 0.13)	–	–
		Richest	0.10 (0.04 to 0.15)	0.12 (0.04 to 0.19)	0.06 (0.03 to 0.08)	–	–
	Niger	Poorest	4.35 (3.61 to 5.09)	6.21 (5.04 to 7.42)	0.69 (0.54 to 0.92)	–	–
		Poorer	3.83 (3.20 to 4.46)	5.53 (4.53 to 6.55)	0.49 (0.37 to 0.61)	–	–
		Middle	3.60 (3.00 to 4.10)	5.24 (4.29 to 6.06)	0.38 (0.27 to 0.48)	–	–
		Richer	2.67 (2.17 to 3.17)	3.89 (3.11 to 4.67)	0.28 (0.20 to 0.37)	–	–
		Richest	1.53 (1.22 to 1.87)	2.22 (1.75 to 2.74)	0.16 (0.11 to 0.20)	–	–
	Nigeria	Poorest	5.31 (4.75 to 5.97)	5.87 (5.18 to 6.63)	4.24 (3.90 to 4.63)	4.84 (4.09 to 5.62)	6.14 (5.37 to 6.91)
		Poorer	4.46 (4.00 to 4.91)	5.27 (4.64 to 5.88)	2.86 (2.72 to 3.01)	0.87 (0.49 to 1.29)	2.10 (1.71 to 2.52)
		Middle	3.44 (3.05 to 3.78)	4.31 (3.73 to 4.79)	1.71 (1.65 to 1.79)	–	0.32 (0.13 to 0.58)
		Richer	1.77 (1.57 to 1.99)	2.26 (1.97 to 2.57)	0.82 (0.78 to 0.85)	–	–
		Richest	0.62 (0.52 to 0.73)	0.80 (0.64 to 0.96)	0.27 (0.25 to 0.29)	–	–
	Rwanda	Poorest	0.15 (0.07 to 0.22)	0.18 (0.08 to 0.29)	0.08 (0.06 to 0.10)	–	–
		Poorer	0.15 (0.09 to 0.20)	0.19 (0.10 to 0.27)	0.07 (0.06 to 0.08)	–	–
		Middle	0.16 (0.11 to 0.22)	0.21 (0.13 to 0.29)	0.07 (0.06 to 0.08)	–	–
		Richer	0.11 (0.07 to 0.17)	0.14 (0.08 to 0.23)	0.06 (0.04 to 0.07)	–	–
		Richest	0.06 (0.03 to 0.10)	0.08 (0.03 to 0.13)	0.03 (0.02 to 0.04)	–	–
	Sao Tome and Principe	Poorest	0.32 (-0.07 to 0.89)	0.43 (-0.06 to 1.32)	0.08 (-0.08 to 0.19)	–	0.48 (0.34 to 0.66)
		Poorer	0.29 (-0.03 to 0.94)	0.40 (-0.03 to 1.39)	0.07 (-0.03 to 0.14)	–	0.08 (0.08 to 0.08)
		Middle	0.28 (0.03 to 0.92)	0.39 (0.03 to 1.37)	0.06 (0.01 to 0.13)	–	–
		Richer	0.19 (-0.03 to 0.81)	0.27 (-0.03 to 1.16)	0.05 (-0.03 to 0.11)	–	–
		Richest	0.10 (-0.03 to 0.52)	0.14 (-0.03 to 0.78)	0.03 (-0.03 to 0.08)	–	–
	Senegal	Poorest	0.33 (0.20 to 0.51)	0.38 (0.22 to 0.63)	0.22 (0.16 to 0.28)	–	–
		Poorer	0.28 (0.19 to 0.41)	0.34 (0.21 to 0.52)	0.17 (0.14 to 0.20)	–	–
		Middle	0.26 (0.18 to 0.35)	0.32 (0.22 to 0.45)	0.14 (0.12 to 0.15)	–	–
		Richer	0.14 (0.09 to 0.21)	0.17 (0.10 to 0.26)	0.09 (0.07 to 0.10)	–	–
		Richest	0.06 (0.03 to 0.09)	0.07 (0.04 to 0.12)	0.04 (0.03 to 0.05)	–	–
	Sierra Leone	Poorest	2.00 (1.40 to 2.68)	2.90 (1.98 to 3.95)	0.22 (0.18 to 0.27)	–	–
		Poorer	1.95 (1.47 to 2.62)	2.84 (2.11 to 3.88)	0.19 (0.15 to 0.22)	–	–
		Middle	2.10 (1.59 to 2.82)	3.08 (2.28 to 4.19)	0.17 (0.14 to 0.21)	–	–
		Richer	1.74 (1.29 to 2.30)	2.55 (1.84 to 3.40)	0.16 (0.12 to 0.20)	–	–
		Richest	1.14 (0.79 to 1.60)	1.66 (1.13 to 2.36)	0.11 (0.08 to 0.14)	–	–
	South Africa	Poorest	0.67 (-0.34 to 3.23)	0.82 (-0.28 to 4.71)	0.33 (-0.45 to 0.53)	1.38 (1.38 to 1.38)	0.33 (0.09 to 2.56)
		Poorer	0.69 (-0.25 to 3.21)	0.90 (-0.21 to 4.63)	0.30 (-0.32 to 0.43)	0.69 (0.69 to 0.69)	0.18 (0.04 to 1.86)
		Middle	0.80 (-0.18 to 3.48)	1.04 (-0.16 to 5.03)	0.30 (-0.21 to 0.46)	0.76 (0.76 to 0.76)	0.09 (0.03 to 1.94)

Indicators	Country	Wealth Quintile	Average Annual Rate of Change (95% CrI)			Added AARC Needed to Achieve Targets (95% CrI)	
			2000–2030	2000–2020	2020–2030	UHC2030	IA2030
		Richer	0.62 (-0.25 to 3.27)	0.80 (-0.22 to 4.72)	0.26 (-0.32 to 0.44)	0.95 (0.95 to 0.95)	0.20 (0.02 to 2.13)
		Richest	0.37 (-0.22 to 2.35)	0.48 (-0.18 to 3.40)	0.17 (-0.29 to 0.34)	0.27 (0.27 to 0.27)	0.14 (0.01 to 1.44)
		Poorest	0.47 (0.35 to 0.67)	0.60 (0.42 to 0.91)	0.20 (0.17 to 0.24)	–	–
		Poorer	0.42 (0.30 to 0.58)	0.56 (0.38 to 0.80)	0.15 (0.13 to 0.18)	–	–
	Tanzania	Middle	0.40 (0.30 to 0.56)	0.54 (0.39 to 0.78)	0.13 (0.10 to 0.15)	–	–
		Richer	0.27 (0.19 to 0.40)	0.35 (0.24 to 0.55)	0.09 (0.08 to 0.11)	–	–
		Richest	0.13 (0.09 to 0.21)	0.17 (0.12 to 0.28)	0.05 (0.04 to 0.07)	–	–
		Poorest	0.86 (-0.03 to 2.64)	1.16 (-0.03 to 3.89)	0.24 (-0.03 to 0.33)	1.18 (1.18 to 1.18)	1.21 (0.05 to 2.36)
		Poorer	0.72 (0.01 to 2.34)	0.98 (0.01 to 3.45)	0.18 (0.01 to 0.23)	0.13 (0.13 to 0.13)	1.30 (1.30 to 1.30)
	Togo	Middle	0.65 (0.06 to 2.29)	0.91 (0.07 to 3.40)	0.14 (0.05 to 0.19)	–	0.56 (0.56 to 0.56)
		Richer	0.42 (0.01 to 1.63)	0.57 (0.01 to 2.40)	0.10 (0.01 to 0.15)	–	0.20 (0.20 to 0.20)
		Richest	0.21 (-0.01 to 1.16)	0.29 (-0.01 to 1.72)	0.05 (-0.01 to 0.09)	–	–
		Poorest	0.74 (0.61 to 0.87)	1.03 (0.84 to 1.22)	0.16 (0.13 to 0.19)	–	–
		Poorer	0.80 (0.70 to 0.93)	1.13 (0.97 to 1.33)	0.15 (0.12 to 0.18)	–	–
	Uganda	Middle	0.97 (0.85 to 1.09)	1.38 (1.18 to 1.58)	0.16 (0.13 to 0.19)	–	–
		Richer	0.86 (0.72 to 0.99)	1.20 (1.00 to 1.41)	0.16 (0.13 to 0.19)	–	–
		Richest	0.57 (0.49 to 0.70)	0.80 (0.67 to 0.99)	0.12 (0.09 to 0.15)	–	–
		Poorest	0.23 (0.14 to 0.33)	0.27 (0.15 to 0.41)	0.16 (0.11 to 0.20)	–	–
		Poorer	0.22 (0.14 to 0.31)	0.27 (0.16 to 0.39)	0.14 (0.11 to 0.16)	–	–
	Zambia	Middle	0.24 (0.17 to 0.31)	0.29 (0.20 to 0.39)	0.13 (0.11 to 0.15)	–	–
		Richer	0.15 (0.09 to 0.21)	0.18 (0.11 to 0.27)	0.09 (0.07 to 0.11)	–	–
		Richest	0.07 (0.04 to 0.11)	0.09 (0.05 to 0.14)	0.05 (0.03 to 0.06)	–	–
		Poorest	1.41 (1.11 to 1.76)	1.90 (1.43 to 2.45)	0.44 (0.37 to 0.53)	–	–
		Poorer	1.44 (1.16 to 1.77)	1.96 (1.52 to 2.48)	0.40 (0.33 to 0.46)	–	–
	Zimbabwe	Middle	1.62 (1.28 to 1.93)	2.23 (1.72 to 2.75)	0.39 (0.32 to 0.46)	–	–
		Richer	1.34 (1.04 to 1.64)	1.83 (1.37 to 2.29)	0.36 (0.29 to 0.42)	–	–
		Richest	0.84 (0.63 to 1.10)	1.15 (0.83 to 1.54)	0.24 (0.19 to 0.29)	–	–
		Poorest	-0.95 (-8.70 to 7.88)	-0.94 (-7.88 to 8.73)	-0.97 (-10.35 to 6.27)	17.42 (5.24 to 32.37)	18.80 (6.56 to 33.81)
		Poorer	-0.31 (-7.31 to 7.29)	-0.30 (-6.25 to 8.57)	-0.31 (-9.38 to 4.88)	10.88 (1.64 to 24.78)	12.19 (2.90 to 26.14)
	Angola	Middle	-0.53 (-6.35 to 5.96)	-0.52 (-5.08 to 7.26)	-0.55 (-8.88 to 3.41)	7.73 (0.50 to 20.01)	8.96 (1.67 to 21.31)
		Richer	-0.72 (-5.59 to 4.58)	-0.69 (-4.03 to 5.76)	-0.78 (-8.61 to 2.33)	5.25 (0.35 to 16.47)	6.41 (0.94 to 17.61)
		Richest	-1.12 (-5.03 to 2.41)	-0.99 (-3.01 to 2.99)	-1.40 (-8.92 to 1.36)	4.01 (0.40 to 13.66)	4.90 (0.31 to 14.88)
		Poorest	0.69 (0.31 to 1.11)	0.72 (0.32 to 1.19)	0.62 (0.30 to 0.94)	2.25 (1.54 to 3.10)	3.47 (2.75 to 4.33)
		Poorer	0.79 (0.49 to 1.11)	0.85 (0.52 to 1.24)	0.66 (0.44 to 0.84)	0.57 (0.09 to 1.11)	1.76 (1.26 to 2.31)
	Benin	Middle	0.50 (0.24 to 0.73)	0.54 (0.25 to 0.80)	0.43 (0.23 to 0.58)	0.20 (0.00 to 0.50)	1.24 (0.85 to 1.68)
		Richer	0.28 (0.06 to 0.48)	0.29 (0.06 to 0.52)	0.25 (0.06 to 0.39)	0.06 (0.02 to 0.13)	0.79 (0.40 to 1.21)
		Richest	-0.07 (-0.23 to 0.07)	-0.07 (-0.21 to 0.07)	-0.08 (-0.26 to 0.07)	–	0.58 (0.26 to 0.92)
		Poorest	4.40 (3.96 to 4.94)	6.62 (5.93 to 7.44)	0.11 (0.08 to 0.15)	–	–
		Poorer	3.95 (3.54 to 4.36)	5.95 (5.31 to 6.57)	0.08 (0.06 to 0.11)	–	–
	Burkina Faso	Middle	3.42 (3.07 to 3.80)	5.13 (4.60 to 5.73)	0.07 (0.05 to 0.09)	–	–
		Richer	2.84 (2.54 to 3.14)	4.26 (3.81 to 4.73)	0.06 (0.04 to 0.09)	–	–
		Richest	1.89 (1.64 to 2.14)	2.83 (2.45 to 3.20)	0.05 (0.04 to 0.07)	–	–
		Poorest	0.32 (0.14 to 0.65)	0.42 (0.16 to 0.90)	0.13 (0.09 to 0.16)	–	–
		Poorer	0.35 (0.16 to 0.63)	0.46 (0.20 to 0.89)	0.12 (0.10 to 0.15)	–	–
	Burundi	Middle	0.34 (0.15 to 0.65)	0.45 (0.17 to 0.90)	0.13 (0.10 to 0.15)	–	–
		Richer	0.32 (0.13 to 0.63)	0.41 (0.15 to 0.88)	0.13 (0.09 to 0.16)	–	–
		Richest	0.22 (0.05 to 0.47)	0.26 (0.05 to 0.62)	0.11 (0.04 to 0.15)	–	–
		Poorest	1.00 (0.50 to 1.47)	1.06 (0.52 to 1.60)	0.88 (0.47 to 1.22)	2.56 (1.72 to 3.57)	3.79 (2.94 to 4.81)
		Poorer	1.02 (0.67 to 1.33)	1.11 (0.71 to 1.50)	0.83 (0.59 to 1.01)	0.70 (0.30 to 1.36)	1.90 (1.49 to 2.56)
	Cameroon	Middle	0.67 (0.38 to 1.00)	0.72 (0.40 to 1.12)	0.55 (0.34 to 0.74)	0.24 (0.01 to 0.61)	1.30 (0.90 to 1.80)
		Richer	0.39 (0.11 to 0.61)	0.42 (0.11 to 0.68)	0.33 (0.10 to 0.48)	0.15 (0.03 to 0.22)	0.77 (0.38 to 1.26)
		Richest	-0.00 (-0.21 to 0.16)	-0.00 (-0.20 to 0.17)	-0.00 (-0.23 to 0.15)	0.14 (0.14 to 0.14)	0.54 (0.20 to 0.97)
		Poorest	4.69 (3.27 to 5.87)	5.08 (3.45 to 6.51)	3.94 (2.92 to 4.59)	5.98 (4.43 to 8.02)	7.28 (5.72 to 9.34)
		Poorer	4.87 (3.71 to 5.94)	5.44 (4.04 to 6.84)	3.76 (3.09 to 4.21)	3.83 (2.49 to 5.46)	5.10 (3.75 to 6.75)
BCG immunization	Chad	Middle	4.29 (3.28 to 5.14)	4.80 (3.55 to 5.88)	3.29 (2.72 to 3.70)	3.35 (2.26 to 4.85)	4.61 (3.52 to 6.13)
		Richer	3.64 (2.61 to 4.53)	4.05 (2.80 to 5.25)	2.78 (2.22 to 3.14)	2.86 (1.70 to 4.36)	4.12 (2.94 to 5.62)
		Richest	2.22 (1.33 to 2.93)	2.43 (1.40 to 3.29)	1.81 (1.18 to 2.20)	2.90 (1.75 to 4.38)	4.13 (2.99 to 5.63)
		Poorest	2.47 (-1.16 to 6.48)	2.97 (-1.08 to 9.26)	1.37 (-1.34 to 1.79)	1.31 (0.11 to 6.75)	1.40 (0.05 to 7.71)
		Poorer	2.24 (-0.65 to 5.78)	2.81 (-0.61 to 8.34)	1.03 (-0.73 to 1.29)	0.93 (0.12 to 4.71)	0.92 (0.04 to 4.98)
	Comoros	Middle	1.73 (-0.78 to 4.69)	2.13 (-0.71 to 6.81)	0.79 (-0.93 to 0.98)	1.09 (0.02 to 5.72)	0.72 (0.01 to 4.32)
		Richer	1.22 (-0.74 to 3.86)	1.55 (-0.64 to 5.53)	0.56 (-0.91 to 0.79)	0.67 (0.03 to 4.84)	0.61 (0.02 to 4.36)
		Richest	0.61 (-0.96 to 2.55)	0.75 (-0.77 to 3.61)	0.33 (-1.33 to 0.53)	0.96 (0.07 to 4.64)	0.53 (0.04 to 4.25)
		Poorest	-3.09 (-5.04 to -1.50)	-2.79 (-4.36 to -1.42)	-3.69 (-6.40 to -1.67)	12.92 (8.85 to 17.85)	14.22 (10.12 to 19.17)
		Poorer	-2.18 (-3.89 to -0.87)	-1.93 (-3.25 to -0.83)	-2.65 (-5.16 to -0.96)	8.55 (5.41 to 12.92)	9.81 (6.65 to 14.19)
	Congo Brazzaville	Middle	-2.09 (-3.61 to -0.89)	-1.79 (-2.90 to -0.83)	-2.68 (-5.01 to -1.01)	7.15 (3.81 to 11.13)	8.39 (5.02 to 12.38)
		Richer	-1.92 (-3.22 to -0.80)	-1.57 (-2.46 to -0.71)	-2.59 (-4.84 to -0.97)	5.56 (2.27 to 9.24)	6.78 (3.47 to 10.48)
		Richest	-2.02 (-3.18 to -0.97)	-1.48 (-2.19 to -0.78)	-3.10 (-5.19 to -1.39)	4.90 (2.01 to 8.35)	6.10 (3.20 to 9.57)
		Poorest	5.72 (4.78 to 6.61)	7.12 (5.70 to 8.55)	2.93 (2.73 to 3.14)	0.18 (0.02 to 1.06)	0.90 (0.29 to 1.76)
		Poorer	5.02 (4.24 to 5.79)	6.50 (5.30 to 7.72)	2.11 (1.97 to 2.26)	–	0.19 (0.01 to 0.70)
	Congo Democratic Republic	Middle	4.06 (3.41 to 4.74)	5.31 (4.27 to 6.37)	1.61 (1.51 to 1.72)	–	0.10 (0.00 to 0.54)
		Richer	3.12 (2.55 to 3.72)	4.10 (3.22 to 5.01)	1.19 (1.07 to 1.28)	–	0.08 (0.04 to 0.23)
		Richest	1.85 (1.44 to 2.29)	2.39 (1.77 to 3.04)	0.78 (0.71 to 0.87)	–	0.20 (0.20 to 0.20)
		Poorest	2.83 (-2.48 to 7.78)	3.31 (-2.21 to 11.16)	1.71 (-2.95 to 2.17)	1.68 (0.22 to 10.46)	2.11 (0.04 to 11.47)
		Poorer	2.63 (-1.60 to 7.02)	3.20 (-1.43 to 10.22)	1.37 (-1.94 to 1.62)	1.34 (0.04 to 7.43)	1.24 (0.06 to 7.97)
	Cote d'Ivoire	Middle	2.14 (-1.46 to 6.14)	2.57 (-1.29 to 8.92)	1.10 (-1.79 to 1.31)	1.34 (0.03 to 6.37)	1.15 (0.16 to 7.28)
		Richer	1.61 (-1.37 to 5.19)	1.96 (-1.19 to 7.56)	0.84 (-1.73 to 1.04)	1.22 (0.12 to 5.40)	0.92 (0.05 to 6.50)
		Richest	0.85 (-1.69 to 3.67)	0.98 (-1.34 to 5.31)	0.51 (-2.40 to 0.76)	1.26 (0.05 to 5.65)	0.81 (0.02 to 5.91)
		Poorest	0.28 (0.21 to 0.34)	0.37 (0.27 to 0.45)	0.12 (0.10 to 0.14)	–	–
		Poorer	0.27 (0.22 to 0.31)	0.35 (0.27 to 0.42)	0.10 (0.08 to 0.11)	–	–
	Egypt	Middle	0.23 (0.18 to 0.26)	0.29 (0.23 to 0.35)	0.09 (0.08 to 0.10)	–	–
		Richer	0.18 (0.14 to 0.23)	0.24 (0.18 to 0.30)	0.08 (0.07 to 0.09)	–	–
		Richest	0.10 (0.07 to 0.14)	0.13 (0.09 to 0.17)	0.06 (0.05 to 0.07)	–	–

Indicators	Country	Wealth Quintile	Average Annual Rate of Change (95% CrI)			Added AARC Needed to Achieve Targets (95% CrI)	
			2000–2030	2000–2020	2020–2030	UHC2030	IA2030
	Eswatini	Poorest	0.36 (-0.26 to 0.91)	0.48 (-0.23 to 1.33)	0.09 (-0.34 to 0.17)	–	0.34 (0.03 to 1.08)
		Poorer	0.34 (-0.18 to 0.79)	0.46 (-0.16 to 1.16)	0.08 (-0.22 to 0.15)	–	0.12 (0.01 to 0.95)
		Middle	0.30 (-0.16 to 0.66)	0.41 (-0.14 to 0.98)	0.07 (-0.21 to 0.14)	–	0.39 (0.12 to 1.05)
		Richer	0.26 (-0.19 to 0.58)	0.35 (-0.17 to 0.87)	0.06 (-0.23 to 0.13)	–	0.35 (0.09 to 1.07)
		Richest	0.16 (-0.34 to 0.44)	0.20 (-0.27 to 0.62)	0.05 (-0.47 to 0.11)	0.47 (0.31 to 0.64)	0.74 (0.35 to 1.81)
	Ethiopia	Poorest	6.24 (5.70 to 6.69)	7.55 (6.81 to 8.18)	3.66 (3.48 to 3.82)	0.47 (0.07 to 0.98)	1.70 (1.30 to 2.22)
		Poorer	5.70 (5.35 to 6.09)	7.17 (6.64 to 7.82)	2.82 (2.71 to 2.95)	–	0.68 (0.39 to 0.97)
		Middle	4.87 (4.52 to 5.16)	6.17 (5.65 to 6.61)	2.29 (2.18 to 2.39)	–	0.38 (0.16 to 0.63)
		Richer	3.95 (3.62 to 4.23)	5.04 (4.54 to 5.48)	1.79 (1.68 to 1.88)	–	0.14 (0.01 to 0.38)
		Richest	2.50 (2.24 to 2.77)	3.15 (2.77 to 3.53)	1.23 (1.15 to 1.32)	–	0.10 (0.01 to 0.29)
	Gabon	Poorest	4.37 (3.89 to 4.88)	6.09 (5.35 to 6.88)	1.03 (0.84 to 1.23)	–	–
		Poorer	3.77 (3.46 to 4.13)	5.36 (4.79 to 5.92)	0.71 (0.57 to 0.85)	–	–
		Middle	3.07 (2.82 to 3.40)	4.36 (3.91 to 4.86)	0.57 (0.46 to 0.69)	–	–
		Richer	2.41 (2.10 to 2.70)	3.40 (2.95 to 3.85)	0.44 (0.35 to 0.57)	–	–
		Richest	1.47 (1.25 to 1.75)	2.03 (1.72 to 2.42)	0.32 (0.24 to 0.42)	–	–
	Gambia	Poorest	0.91 (0.49 to 1.56)	1.19 (0.60 to 2.18)	0.35 (0.28 to 0.44)	–	–
		Poorer	1.01 (0.59 to 1.74)	1.33 (0.73 to 2.43)	0.35 (0.29 to 0.41)	–	–
		Middle	1.02 (0.57 to 1.80)	1.34 (0.69 to 2.50)	0.39 (0.32 to 0.44)	–	–
		Richer	0.99 (0.54 to 1.70)	1.29 (0.64 to 2.34)	0.42 (0.33 to 0.49)	–	–
		Richest	0.72 (0.32 to 1.42)	0.89 (0.36 to 1.91)	0.37 (0.24 to 0.51)	–	0.01 (0.01 to 0.01)
	Ghana	Poorest	1.04 (0.76 to 1.35)	1.32 (0.89 to 1.76)	0.50 (0.43 to 0.58)	–	0.02 (0.02 to 0.02)
		Poorer	0.93 (0.70 to 1.19)	1.19 (0.85 to 1.60)	0.40 (0.35 to 0.45)	–	–
		Middle	0.75 (0.52 to 0.99)	0.96 (0.63 to 1.30)	0.34 (0.29 to 0.39)	–	–
		Richer	0.57 (0.40 to 0.80)	0.73 (0.48 to 1.05)	0.27 (0.23 to 0.33)	–	–
		Richest	0.30 (0.17 to 0.46)	0.36 (0.18 to 0.59)	0.18 (0.13 to 0.24)	–	–
	Guinea	Poorest	-2.38 (-3.22 to -1.49)	-2.25 (-2.99 to -1.44)	-2.63 (-3.67 to -1.59)	13.55 (11.54 to 15.49)	14.86 (12.84 to 16.82)
		Poorer	-1.64 (-2.51 to -0.87)	-1.55 (-2.31 to -0.85)	-1.82 (-2.89 to -0.92)	9.94 (8.35 to 11.97)	11.22 (9.62 to 13.27)
		Middle	-1.77 (-2.59 to -1.02)	-1.64 (-2.33 to -0.98)	-2.02 (-3.08 to -1.11)	8.99 (7.33 to 10.76)	10.25 (8.59 to 12.04)
		Richer	-1.88 (-2.55 to -1.13)	-1.70 (-2.23 to -1.07)	-2.24 (-3.19 to -1.27)	7.95 (6.33 to 9.53)	9.20 (7.57 to 10.79)
		Richest	-2.40 (-3.00 to -1.77)	-2.02 (-2.44 to -1.55)	-3.19 (-4.13 to -2.23)	7.78 (6.11 to 9.38)	9.02 (7.34 to 10.63)
	Kenya	Poorest	1.82 (1.52 to 2.18)	2.47 (2.00 to 2.99)	0.55 (0.49 to 0.61)	–	–
		Poorer	1.60 (1.35 to 1.86)	2.22 (1.81 to 2.58)	0.41 (0.37 to 0.45)	–	–
		Middle	1.30 (1.08 to 1.51)	1.79 (1.44 to 2.11)	0.35 (0.31 to 0.38)	–	–
		Richer	1.01 (0.84 to 1.22)	1.38 (1.11 to 1.70)	0.28 (0.25 to 0.32)	–	–
		Richest	0.59 (0.47 to 0.73)	0.78 (0.61 to 0.98)	0.21 (0.18 to 0.24)	–	–
	Lesotho	Poorest	0.50 (-0.07 to 0.95)	0.55 (-0.07 to 1.14)	0.40 (-0.07 to 0.59)	0.25 (0.02 to 0.61)	0.52 (0.03 to 1.63)
		Poorer	0.53 (0.07 to 0.92)	0.61 (0.07 to 1.13)	0.38 (0.07 to 0.50)	–	0.24 (0.01 to 0.98)
		Middle	0.41 (-0.04 to 0.75)	0.46 (-0.04 to 0.91)	0.31 (-0.05 to 0.43)	–	0.29 (0.00 to 0.89)
		Richer	0.29 (-0.10 to 0.58)	0.32 (-0.10 to 0.69)	0.23 (-0.10 to 0.35)	–	0.23 (0.01 to 0.86)
		Richest	0.04 (-0.34 to 0.28)	0.04 (-0.30 to 0.32)	0.04 (-0.41 to 0.21)	0.10 (0.01 to 0.19)	0.26 (0.01 to 1.11)
	Liberia	Poorest	3.70 (3.02 to 4.39)	4.52 (3.56 to 5.46)	2.09 (1.91 to 2.29)	0.10 (0.00 to 0.54)	0.92 (0.44 to 1.43)
		Poorer	3.33 (2.71 to 3.91)	4.21 (3.31 to 5.04)	1.61 (1.49 to 1.71)	–	0.21 (0.03 to 0.59)
		Middle	2.71 (2.23 to 3.19)	3.44 (2.74 to 4.14)	1.29 (1.19 to 1.39)	–	0.11 (0.00 to 0.36)
		Richer	2.09 (1.69 to 2.53)	2.63 (2.05 to 3.26)	0.99 (0.90 to 1.12)	–	0.04 (0.00 to 0.29)
		Richest	1.19 (0.89 to 1.51)	1.45 (1.05 to 1.90)	0.65 (0.56 to 0.78)	–	0.08 (0.02 to 0.23)
	Madagascar	Poorest	3.57 (2.68 to 4.33)	4.64 (3.26 to 5.91)	1.40 (1.09 to 1.63)	0.03 (0.03 to 0.03)	0.22 (0.03 to 1.13)
		Poorer	2.60 (2.05 to 3.09)	3.52 (2.60 to 4.36)	0.79 (0.59 to 0.97)	–	0.07 (0.07 to 0.19)
		Middle	1.69 (1.32 to 2.04)	2.29 (1.67 to 2.89)	0.49 (0.36 to 0.62)	–	–
		Richer	1.02 (0.75 to 1.28)	1.38 (0.96 to 1.80)	0.29 (0.21 to 0.37)	–	–
		Richest	0.44 (0.30 to 0.57)	0.57 (0.38 to 0.78)	0.15 (0.11 to 0.19)	–	–
	Malawi	Poorest	0.76 (0.61 to 0.86)	0.97 (0.76 to 1.13)	0.33 (0.30 to 0.36)	–	–
		Poorer	0.70 (0.61 to 0.78)	0.91 (0.77 to 1.04)	0.27 (0.25 to 0.29)	–	–
		Middle	0.59 (0.51 to 0.65)	0.76 (0.65 to 0.87)	0.24 (0.22 to 0.26)	–	–
		Richer	0.48 (0.38 to 0.54)	0.61 (0.48 to 0.70)	0.21 (0.19 to 0.23)	–	–
		Richest	0.27 (0.21 to 0.32)	0.33 (0.25 to 0.40)	0.15 (0.13 to 0.17)	–	–
	Mali	Poorest	3.30 (3.07 to 3.58)	4.11 (3.75 to 4.50)	1.71 (1.61 to 1.82)	–	0.42 (0.21 to 0.63)
		Poorer	3.04 (2.82 to 3.25)	3.90 (3.56 to 4.21)	1.36 (1.29 to 1.43)	–	0.05 (0.00 to 0.14)
		Middle	2.56 (2.36 to 2.74)	3.27 (2.97 to 3.53)	1.16 (1.09 to 1.21)	–	0.02 (0.00 to 0.12)
		Richer	2.06 (1.88 to 2.22)	2.62 (2.34 to 2.85)	0.95 (0.88 to 1.00)	–	–
		Richest	1.23 (1.09 to 1.39)	1.52 (1.31 to 1.73)	0.67 (0.62 to 0.72)	–	0.01 (0.01 to 0.01)
	Morocco	Poorest	0.40 (-0.48 to 0.74)	0.54 (-0.40 to 1.10)	0.10 (-0.61 to 0.20)	2.21 (0.33 to 3.61)	0.77 (0.15 to 4.80)
		Poorer	0.28 (-0.25 to 0.48)	0.39 (-0.22 to 0.71)	0.06 (-0.30 to 0.13)	0.39 (0.16 to 1.43)	0.90 (0.16 to 2.61)
		Middle	0.18 (-0.18 to 0.31)	0.25 (-0.15 to 0.46)	0.04 (-0.22 to 0.09)	0.37 (0.37 to 0.37)	0.69 (0.42 to 1.55)
		Richer	0.11 (-0.16 to 0.20)	0.15 (-0.13 to 0.30)	0.03 (-0.21 to 0.07)	–	0.13 (0.02 to 0.95)
		Richest	0.05 (-0.21 to 0.10)	0.06 (-0.14 to 0.15)	0.01 (-0.33 to 0.04)	–	0.61 (0.61 to 0.61)
	Mozambique	Poorest	0.91 (0.28 to 1.52)	0.98 (0.28 to 1.74)	0.77 (0.26 to 1.10)	1.32 (0.25 to 2.88)	2.53 (1.37 to 4.08)
		Poorer	0.84 (0.44 to 1.25)	0.92 (0.46 to 1.47)	0.66 (0.39 to 0.82)	0.26 (0.01 to 0.89)	1.02 (0.29 to 1.82)
		Middle	0.53 (0.21 to 0.81)	0.59 (0.22 to 0.95)	0.42 (0.20 to 0.54)	0.18 (0.02 to 0.50)	0.47 (0.05 to 1.17)
		Richer	0.29 (0.05 to 0.49)	0.31 (0.05 to 0.57)	0.24 (0.05 to 0.34)	–	0.21 (0.01 to 0.69)
		Richest	0.02 (-0.16 to 0.15)	0.02 (-0.15 to 0.17)	0.02 (-0.18 to 0.13)	–	0.10 (0.01 to 0.46)
	Namibia	Poorest	0.45 (0.11 to 0.71)	0.49 (0.11 to 0.83)	0.35 (0.11 to 0.48)	0.07 (0.00 to 0.50)	0.34 (0.03 to 1.18)
		Poorer	0.51 (0.27 to 0.74)	0.58 (0.29 to 0.88)	0.38 (0.23 to 0.46)	–	0.20 (0.01 to 0.65)
		Middle	0.42 (0.15 to 0.63)	0.47 (0.16 to 0.73)	0.32 (0.14 to 0.42)	–	0.18 (0.02 to 0.74)
		Richer	0.34 (0.07 to 0.51)	0.37 (0.07 to 0.59)	0.27 (0.07 to 0.37)	–	0.20 (0.01 to 0.70)
		Richest	0.04 (-0.26 to 0.26)	0.04 (-0.24 to 0.28)	0.04 (-0.31 to 0.21)	0.24 (0.24 to 0.24)	0.34 (0.02 to 1.00)
	Niger	Poorest	8.09 (7.29 to 8.92)	11.64 (10.35 to 12.93)	1.37 (1.08 to 1.70)	–	–
		Poorer	7.31 (6.58 to 8.06)	10.65 (9.43 to 11.88)	0.94 (0.76 to 1.20)	–	–
		Middle	6.37 (5.67 to 7.13)	9.31 (8.12 to 10.52)	0.76 (0.62 to 0.96)	–	–
		Richer	5.36 (4.68 to 6.01)	7.84 (6.76 to 8.89)	0.60 (0.49 to 0.75)	–	–
		Richest	3.74 (3.17 to 4.36)	5.40 (4.51 to 6.40)	0.46 (0.36 to 0.58)	–	–
	Nigeria	Poorest	7.91 (7.10 to 8.55)	8.55 (7.63 to 9.33)	6.63 (6.12 to 7.01)	7.55 (6.63 to 8.71)	8.90 (7.98 to 10.07)
		Poorer	7.05 (6.42 to 7.58)	8.15 (7.29 to 8.85)	4.89 (4.69 to 5.10)	2.37 (1.87 to 3.13)	3.64 (3.14 to 4.41)

Indicators	Country	Wealth Quintile	Average Annual Rate of Change (95% CrI)			Added AARC Needed to Achieve Targets (95% CrI)	
			2000–2030	2000–2020	2020–2030	UHC2030	IA2030
		Middle	5.24 (4.82 to 5.68)	6.34 (5.73 to 6.96)	3.10 (2.98 to 3.20)	0.37 (0.07 to 0.78)	1.59 (1.28 to 2.00)
		Richer	3.42 (3.09 to 3.79)	4.28 (3.80 to 4.82)	1.73 (1.66 to 1.81)	–	0.36 (0.16 to 0.59)
		Richest	1.52 (1.33 to 1.72)	1.89 (1.62 to 2.17)	0.79 (0.74 to 0.84)	–	0.02 (0.02 to 0.02)
	Rwanda	Poorest	0.74 (0.64 to 0.86)	1.01 (0.85 to 1.20)	0.21 (0.18 to 0.25)	–	–
		Poorer	0.78 (0.68 to 0.89)	1.07 (0.91 to 1.24)	0.20 (0.17 to 0.23)	–	–
		Middle	0.76 (0.66 to 0.86)	1.04 (0.87 to 1.20)	0.21 (0.18 to 0.24)	–	–
		Richer	0.71 (0.60 to 0.82)	0.97 (0.79 to 1.13)	0.21 (0.18 to 0.24)	–	–
		Richest	0.50 (0.41 to 0.60)	0.65 (0.53 to 0.80)	0.19 (0.17 to 0.22)	–	–
	Sao Tome and Principe	Poorest	1.07 (-0.71 to 3.05)	1.42 (-0.60 to 4.57)	0.32 (-0.93 to 0.55)	1.25 (0.16 to 3.83)	0.79 (0.06 to 5.03)
		Poorer	0.97 (-0.46 to 3.06)	1.32 (-0.40 to 4.58)	0.26 (-0.58 to 0.42)	1.38 (0.26 to 2.94)	0.85 (0.03 to 4.13)
		Middle	0.85 (-0.49 to 2.79)	1.15 (-0.42 to 4.19)	0.24 (-0.63 to 0.36)	1.56 (0.19 to 3.14)	0.61 (0.00 to 4.34)
		Richer	0.69 (-0.57 to 2.57)	0.91 (-0.47 to 3.83)	0.20 (-0.79 to 0.32)	1.58 (0.24 to 3.94)	0.64 (0.06 to 5.14)
		Richest	0.40 (-0.73 to 1.91)	0.52 (-0.53 to 2.82)	0.14 (-1.12 to 0.25)	1.54 (0.43 to 3.91)	0.72 (0.00 to 5.11)
	Senegal	Poorest	1.69 (1.40 to 1.92)	2.20 (1.77 to 2.55)	0.68 (0.64 to 0.73)	–	–
		Poorer	1.41 (1.17 to 1.61)	1.88 (1.51 to 2.17)	0.49 (0.46 to 0.51)	–	–
		Middle	1.08 (0.93 to 1.22)	1.42 (1.20 to 1.64)	0.38 (0.36 to 0.41)	–	–
		Richer	0.79 (0.64 to 0.90)	1.04 (0.82 to 1.20)	0.29 (0.26 to 0.32)	–	–
		Richest	0.41 (0.32 to 0.51)	0.52 (0.40 to 0.65)	0.19 (0.17 to 0.22)	–	–
	Sierra Leone	Poorest	3.54 (3.04 to 4.10)	4.87 (4.11 to 5.72)	0.91 (0.82 to 1.02)	–	–
		Poorer	3.56 (3.08 to 4.14)	4.97 (4.22 to 5.87)	0.82 (0.75 to 0.90)	–	–
		Middle	3.45 (2.97 to 3.98)	4.77 (4.03 to 5.61)	0.84 (0.77 to 0.91)	–	–
		Richer	3.23 (2.75 to 3.76)	4.45 (3.72 to 5.27)	0.84 (0.76 to 0.92)	–	–
		Richest	2.47 (2.06 to 3.01)	3.30 (2.70 to 4.13)	0.78 (0.70 to 0.88)	–	–
	South Africa	Poorest	2.08 (-2.78 to 6.68)	2.43 (-2.13 to 9.32)	1.36 (-4.06 to 2.21)	1.56 (0.07 to 10.93)	1.66 (0.09 to 10.98)
		Poorer	2.26 (-2.64 to 6.94)	2.70 (-2.02 to 9.51)	1.42 (-3.86 to 2.05)	1.46 (0.05 to 9.62)	1.39 (0.07 to 10.10)
		Middle	2.26 (-2.76 to 6.90)	2.64 (-2.13 to 9.36)	1.48 (-4.00 to 2.24)	1.64 (0.02 to 10.34)	1.50 (0.03 to 10.56)
		Richer	2.12 (-2.89 to 6.67)	2.42 (-2.20 to 9.10)	1.47 (-4.25 to 2.34)	1.65 (0.06 to 11.42)	1.57 (0.07 to 10.36)
		Richest	1.41 (-3.89 to 5.71)	1.56 (-2.75 to 7.62)	1.10 (-6.27 to 2.36)	2.14 (0.05 to 12.63)	2.17 (0.14 to 12.05)
	Tanzania	Poorest	0.28 (-0.08 to 0.58)	0.30 (-0.08 to 0.65)	0.25 (-0.09 to 0.45)	0.20 (0.00 to 0.45)	0.65 (0.12 to 1.45)
		Poorer	0.28 (0.06 to 0.51)	0.31 (0.06 to 0.59)	0.23 (0.05 to 0.33)	–	0.14 (0.00 to 0.48)
		Middle	0.17 (-0.02 to 0.31)	0.18 (-0.02 to 0.36)	0.14 (-0.02 to 0.22)	–	0.05 (0.01 to 0.13)
		Richer	0.08 (-0.05 to 0.19)	0.08 (-0.05 to 0.21)	0.07 (-0.05 to 0.14)	–	–
		Richest	-0.03 (-0.13 to 0.05)	-0.03 (-0.11 to 0.05)	-0.03 (-0.16 to 0.04)	–	–
	Togo	Poorest	2.02 (0.03 to 4.66)	2.62 (0.03 to 6.81)	0.67 (0.03 to 0.86)	0.70 (0.18 to 2.51)	0.44 (0.01 to 3.08)
		Poorer	1.98 (0.20 to 4.68)	2.67 (0.21 to 6.88)	0.58 (0.18 to 0.69)	0.93 (0.28 to 1.59)	0.30 (0.03 to 2.78)
		Middle	1.80 (0.04 to 4.59)	2.41 (0.04 to 6.74)	0.55 (0.04 to 0.66)	0.96 (0.46 to 1.46)	0.31 (0.03 to 2.64)
		Richer	1.57 (-0.03 to 4.58)	2.08 (-0.03 to 6.70)	0.50 (-0.03 to 0.63)	1.16 (1.04 to 1.28)	0.35 (0.06 to 2.46)
		Richest	1.01 (-0.25 to 3.56)	1.32 (-0.23 to 5.23)	0.40 (-0.30 to 0.55)	1.60 (1.44 to 1.77)	0.26 (0.00 to 2.95)
	Uganda	Poorest	2.53 (2.28 to 2.80)	3.29 (2.92 to 3.68)	1.02 (0.95 to 1.10)	–	–
		Poorer	2.59 (2.38 to 2.82)	3.42 (3.10 to 3.78)	0.95 (0.89 to 1.01)	–	–
		Middle	2.49 (2.29 to 2.69)	3.27 (2.96 to 3.58)	0.97 (0.92 to 1.02)	–	–
		Richer	2.32 (2.10 to 2.54)	3.01 (2.67 to 3.34)	0.95 (0.89 to 1.02)	–	–
		Richest	1.67 (1.45 to 1.88)	2.09 (1.77 to 2.40)	0.83 (0.78 to 0.91)	–	0.00 (0.00 to 0.01)
	Zambia	Poorest	1.09 (0.94 to 1.28)	1.33 (1.11 to 1.60)	0.62 (0.56 to 0.68)	–	0.01 (0.01 to 0.01)
		Poorer	0.96 (0.78 to 1.08)	1.20 (0.96 to 1.39)	0.47 (0.44 to 0.51)	–	–
		Middle	0.72 (0.59 to 0.83)	0.89 (0.71 to 1.05)	0.37 (0.34 to 0.40)	–	–
		Richer	0.52 (0.41 to 0.63)	0.63 (0.49 to 0.79)	0.28 (0.25 to 0.31)	–	–
		Richest	0.24 (0.15 to 0.32)	0.28 (0.17 to 0.38)	0.16 (0.12 to 0.19)	–	–
	Zimbabwe	Poorest	2.60 (2.16 to 3.01)	3.52 (2.84 to 4.23)	0.77 (0.64 to 0.90)	–	–
		Poorer	2.47 (2.09 to 2.92)	3.40 (2.75 to 4.14)	0.64 (0.53 to 0.74)	–	–
		Middle	2.23 (1.82 to 2.64)	3.05 (2.41 to 3.70)	0.60 (0.51 to 0.70)	–	–
		Richer	1.95 (1.54 to 2.37)	2.65 (2.03 to 3.29)	0.55 (0.46 to 0.64)	–	–
		Richest	1.31 (0.98 to 1.68)	1.74 (1.23 to 2.30)	0.46 (0.39 to 0.53)	–	–
MCV1 immunization	Angola	Poorest	-2.74 (-10.60 to 5.07)	-2.69 (-9.82 to 5.30)	-2.84 (-12.27 to 4.62)	25.27 (12.14 to 41.47)	26.73 (13.53 to 43.00)
		Poorer	-1.90 (-9.31 to 5.02)	-1.85 (-8.08 to 5.44)	-2.00 (-11.65 to 4.18)	16.91 (6.25 to 32.18)	18.27 (7.56 to 33.62)
		Middle	-1.82 (-8.12 to 4.27)	-1.74 (-6.63 to 4.74)	-1.98 (-11.13 to 3.32)	12.37 (3.59 to 26.07)	13.68 (4.85 to 27.44)
		Richer	-1.96 (-7.13 to 3.09)	-1.79 (-5.23 to 3.49)	-2.32 (-10.93 to 2.28)	8.84 (1.85 to 21.33)	10.10 (3.00 to 22.36)
		Richest	-2.00 (-6.41 to 1.43)	-1.65 (-3.89 to 1.61)	-2.67 (-11.01 to 1.07)	6.08 (0.89 to 17.98)	7.20 (1.91 to 19.07)
	Benin	Poorest	1.34 (0.85 to 1.82)	1.42 (0.88 to 1.97)	1.19 (0.79 to 1.49)	3.52 (2.53 to 4.47)	4.76 (3.76 to 5.72)
		Poorer	1.40 (1.09 to 1.80)	1.54 (1.16 to 2.01)	1.15 (0.94 to 1.37)	1.63 (1.05 to 2.28)	2.84 (2.26 to 3.50)
		Middle	1.13 (0.78 to 1.46)	1.24 (0.83 to 1.65)	0.92 (0.69 to 1.10)	1.01 (0.48 to 1.57)	2.22 (1.68 to 2.78)
		Richer	0.64 (0.37 to 0.96)	0.68 (0.38 to 1.05)	0.55 (0.34 to 0.76)	0.74 (0.22 to 1.31)	1.94 (1.40 to 2.50)
		Richest	0.10 (-0.16 to 0.33)	0.10 (-0.15 to 0.35)	0.09 (-0.16 to 0.30)	0.47 (0.05 to 0.98)	1.65 (1.20 to 2.15)
	Burkina Faso	Poorest	5.40 (4.82 to 5.88)	8.11 (7.21 to 8.86)	0.20 (0.15 to 0.27)	–	–
		Poorer	4.94 (4.52 to 5.40)	7.42 (6.76 to 8.15)	0.15 (0.11 to 0.20)	–	–
		Middle	4.48 (4.13 to 4.82)	6.73 (6.17 to 7.27)	0.13 (0.10 to 0.18)	–	–
		Richer	3.77 (3.41 to 4.15)	5.65 (5.09 to 6.24)	0.12 (0.09 to 0.16)	–	–
		Richest	2.73 (2.45 to 3.10)	4.07 (3.62 to 4.64)	0.10 (0.07 to 0.14)	–	–
	Burundi	Poorest	-0.15 (-0.60 to 0.31)	-0.14 (-0.48 to 0.34)	-0.17 (-0.84 to 0.25)	0.30 (0.00 to 0.61)	0.60 (0.07 to 1.59)
		Poorer	-0.03 (-0.41 to 0.33)	-0.03 (-0.35 to 0.37)	-0.04 (-0.54 to 0.25)	–	0.29 (0.02 to 0.96)
		Middle	-0.06 (-0.41 to 0.28)	-0.06 (-0.34 to 0.31)	-0.06 (-0.55 to 0.21)	–	0.27 (0.02 to 0.90)
		Richer	-0.16 (-0.49 to 0.16)	-0.15 (-0.39 to 0.17)	-0.18 (-0.70 to 0.14)	–	0.34 (0.03 to 1.00)
		Richest	-0.28 (-0.61 to 0.02)	-0.24 (-0.43 to 0.02)	-0.37 (-0.96 to 0.02)	0.16 (0.09 to 0.20)	0.40 (0.04 to 1.16)
	Cameroon	Poorest	0.84 (0.24 to 1.49)	0.87 (0.24 to 1.57)	0.79 (0.24 to 1.34)	5.84 (4.52 to 6.90)	7.10 (5.77 to 8.17)
		Poorer	1.02 (0.56 to 1.54)	1.07 (0.58 to 1.67)	0.91 (0.53 to 1.28)	3.03 (2.17 to 3.82)	4.26 (3.40 to 5.05)
		Middle	0.75 (0.41 to 1.19)	0.79 (0.42 to 1.29)	0.67 (0.38 to 0.99)	1.99 (1.24 to 2.72)	3.21 (2.45 to 3.94)
		Richer	0.29 (-0.07 to 0.65)	0.30 (-0.07 to 0.69)	0.27 (-0.07 to 0.56)	1.37 (0.76 to 2.09)	2.57 (1.96 to 3.30)
		Richest	-0.18 (-0.43 to 0.13)	-0.18 (-0.40 to 0.13)	-0.19 (-0.49 to 0.12)	0.85 (0.29 to 1.46)	2.04 (1.47 to 2.65)
	Chad	Poorest	6.85 (5.16 to 8.39)	7.60 (5.54 to 9.53)	5.38 (4.35 to 6.12)	5.03 (3.11 to 7.36)	6.34 (4.40 to 8.69)
		Poorer	6.93 (5.48 to 8.28)	7.93 (6.09 to 9.80)	5.02 (4.29 to 5.50)	3.12 (1.72 to 5.03)	4.40 (2.99 to 6.32)
		Middle	6.49 (5.03 to 7.77)	7.42 (5.60 to 9.25)	4.63 (4.02 to 5.01)	2.75 (1.26 to 4.44)	4.03 (2.51 to 5.73)
		Richer	5.53 (4.10 to 6.85)	6.31 (4.49 to 8.11)	4.01 (3.30 to 4.40)	2.76 (1.28 to 4.53)	4.03 (2.53 to 5.81)

Indicators	Country	Wealth Quintile	Average Annual Rate of Change (95% CrI)			Added AARC Needed to Achieve Targets (95% CrI)	
			2000–2030	2000–2020	2020–2030	UHC2030	IA2030
		Richest	4.08 (2.77 to 5.26)	4.61 (2.98 to 6.13)	3.05 (2.32 to 3.47)	2.72 (1.18 to 4.46)	3.97 (2.42 to 5.73)
	Comoros	Poorest	2.37 (-1.84 to 6.16)	2.68 (-1.68 to 8.39)	1.66 (-2.15 to 2.36)	1.90 (0.06 to 12.21)	2.35 (0.14 to 12.81)
		Poorer	2.20 (-1.24 to 5.21)	2.61 (-1.14 to 7.15)	1.31 (-1.45 to 1.65)	1.62 (0.07 to 8.92)	1.40 (0.13 to 8.63)
		Middle	1.78 (-1.23 to 4.37)	2.09 (-1.10 to 6.11)	1.04 (-1.50 to 1.29)	1.32 (0.07 to 7.37)	1.14 (0.03 to 8.28)
		Richer	1.13 (-1.27 to 3.36)	1.33 (-1.09 to 4.67)	0.74 (-1.64 to 0.99)	0.97 (0.02 to 7.05)	1.09 (0.03 to 7.42)
		Richest	0.54 (-1.46 to 2.16)	0.62 (-1.13 to 2.97)	0.41 (-2.10 to 0.69)	1.00 (0.05 to 6.08)	1.02 (0.04 to 6.27)
	Congo Brazzaville	Poorest	-2.43 (-4.24 to -0.83)	-2.29 (-3.89 to -0.81)	-2.71 (-4.94 to -0.86)	13.54 (9.58 to 18.35)	14.85 (10.87 to 19.69)
		Poorer	-1.70 (-3.37 to -0.31)	-1.60 (-3.04 to -0.31)	-1.91 (-4.02 to -0.32)	9.66 (6.28 to 13.92)	10.93 (7.54 to 15.22)
		Middle	-1.72 (-3.14 to -0.31)	-1.59 (-2.79 to -0.31)	-1.99 (-3.90 to -0.32)	8.30 (4.91 to 12.29)	9.56 (6.15 to 13.58)
		Richer	-1.92 (-3.35 to -0.65)	-1.70 (-2.88 to -0.62)	-2.35 (-4.28 to -0.71)	7.43 (4.13 to 11.65)	8.67 (5.35 to 12.92)
		Richest	-2.21 (-3.55 to -1.09)	-1.82 (-2.78 to -0.97)	-2.99 (-5.11 to -1.32)	6.70 (3.50 to 10.63)	7.93 (4.71 to 11.88)
	Congo Democratic Republic	Poorest	5.99 (4.80 to 7.19)	7.26 (5.60 to 9.09)	3.46 (3.18 to 3.66)	0.57 (0.02 to 1.63)	1.65 (0.70 to 2.76)
		Poorer	5.43 (4.50 to 6.49)	6.86 (5.48 to 8.51)	2.67 (2.49 to 2.80)	0.16 (0.00 to 0.64)	0.62 (0.07 to 1.46)
		Middle	4.74 (3.97 to 5.62)	6.03 (4.84 to 7.45)	2.19 (2.06 to 2.30)	0.18 (0.08 to 0.27)	0.35 (0.01 to 0.96)
		Richer	3.70 (3.04 to 4.64)	4.70 (3.72 to 6.18)	1.73 (1.63 to 1.83)	0.11 (0.11 to 0.11)	0.27 (0.01 to 0.82)
		Richest	2.45 (1.88 to 3.13)	3.09 (2.25 to 4.13)	1.20 (1.10 to 1.30)	–	0.22 (0.00 to 0.63)
	Cote d'Ivoire	Poorest	3.02 (-2.80 to 7.95)	3.38 (-2.61 to 10.74)	2.25 (-3.18 to 3.01)	3.49 (0.13 to 15.89)	3.74 (0.09 to 17.09)
		Poorer	2.94 (-2.15 to 7.10)	3.42 (-1.96 to 9.88)	1.86 (-2.52 to 2.36)	2.78 (0.14 to 11.52)	2.67 (0.12 to 12.75)
		Middle	2.39 (-2.12 to 6.11)	2.77 (-1.89 to 8.48)	1.51 (-2.56 to 1.94)	2.51 (0.24 to 11.75)	2.29 (0.06 to 10.82)
		Richer	1.78 (-2.25 to 5.20)	2.03 (-1.93 to 7.31)	1.12 (-2.88 to 1.55)	2.43 (0.09 to 10.73)	2.08 (0.08 to 10.28)
		Richest	0.91 (-2.51 to 3.61)	1.00 (-2.01 to 4.93)	0.68 (-3.56 to 1.11)	2.47 (0.08 to 10.53)	1.91 (0.06 to 10.68)
	Egypt	Poorest	-0.89 (-1.22 to -0.62)	-0.71 (-0.92 to -0.52)	-1.25 (-1.85 to -0.82)	1.38 (0.61 to 2.30)	2.57 (1.79 to 3.49)
		Poorer	-0.66 (-0.91 to -0.42)	-0.54 (-0.71 to -0.37)	-0.90 (-1.32 to -0.53)	0.66 (0.10 to 1.38)	1.84 (1.21 to 2.55)
		Middle	-0.68 (-1.01 to -0.48)	-0.55 (-0.76 to -0.41)	-0.93 (-1.50 to -0.63)	0.68 (0.12 to 1.58)	1.86 (1.30 to 2.77)
		Richer	-0.83 (-1.15 to -0.61)	-0.64 (-0.82 to -0.49)	-1.23 (-1.80 to -0.84)	1.00 (0.35 to 1.86)	2.18 (1.53 to 3.05)
		Richest	-1.01 (-1.29 to -0.77)	-0.70 (-0.85 to -0.57)	-1.63 (-2.19 to -1.16)	1.31 (0.61 to 2.15)	2.49 (1.79 to 3.33)
	Eswatini	Poorest	0.65 (-1.18 to 1.52)	0.84 (-0.91 to 2.20)	0.21 (-1.69 to 0.38)	1.60 (0.43 to 5.08)	0.97 (0.11 to 6.26)
		Poorer	0.63 (-0.82 to 1.32)	0.86 (-0.67 to 1.92)	0.19 (-1.12 to 0.31)	1.96 (0.54 to 4.39)	0.66 (0.10 to 5.59)
		Middle	0.60 (-0.91 to 1.17)	0.78 (-0.72 to 1.72)	0.18 (-1.28 to 0.31)	1.79 (0.03 to 4.74)	0.65 (0.03 to 5.94)
		Richer	0.49 (-1.19 to 1.00)	0.62 (-0.89 to 1.43)	0.16 (-1.79 to 0.28)	2.17 (0.22 to 5.39)	0.66 (0.02 to 6.58)
		Richest	0.30 (-1.16 to 0.72)	0.37 (-0.82 to 1.03)	0.12 (-1.85 to 0.24)	1.14 (0.02 to 6.54)	0.58 (0.02 to 7.38)
	Ethiopia	Poorest	7.28 (6.71 to 7.92)	8.21 (7.52 to 9.00)	5.44 (5.12 to 5.78)	3.76 (2.98 to 4.70)	5.05 (4.27 to 6.00)
		Poorer	6.90 (6.41 to 7.45)	8.11 (7.45 to 8.88)	4.51 (4.32 to 4.69)	1.54 (0.94 to 2.17)	2.79 (2.19 to 3.43)
		Middle	6.11 (5.60 to 6.56)	7.33 (6.62 to 8.00)	3.71 (3.57 to 3.86)	0.73 (0.28 to 1.21)	1.97 (1.51 to 2.45)
		Richer	4.88 (4.42 to 5.32)	5.88 (5.24 to 6.50)	2.88 (2.77 to 3.00)	0.35 (0.01 to 0.82)	1.54 (1.14 to 2.00)
		Richest	3.26 (2.95 to 3.59)	3.91 (3.48 to 4.38)	1.95 (1.83 to 2.08)	0.10 (0.01 to 0.37)	1.13 (0.76 to 1.50)
	Gabon	Poorest	5.76 (4.85 to 6.61)	6.96 (5.71 to 8.25)	3.38 (3.11 to 3.65)	0.57 (0.03 to 1.68)	1.66 (0.82 to 2.85)
		Poorer	5.96 (5.06 to 6.70)	7.34 (6.07 to 8.55)	3.21 (2.94 to 3.45)	0.34 (0.02 to 1.15)	1.20 (0.45 to 2.10)
		Middle	5.87 (5.10 to 6.67)	7.20 (6.00 to 8.47)	3.28 (3.00 to 3.54)	0.45 (0.04 to 1.32)	1.39 (0.48 to 2.45)
		Richer	5.37 (4.47 to 6.15)	6.45 (5.22 to 7.65)	3.20 (2.89 to 3.52)	0.73 (0.04 to 1.96)	1.81 (0.82 to 3.19)
		Richest	4.32 (3.34 to 5.09)	5.08 (3.87 to 6.24)	2.78 (2.37 to 3.17)	1.08 (0.09 to 2.83)	2.24 (1.03 to 4.05)
	Gambia	Poorest	0.58 (0.12 to 1.16)	0.67 (0.12 to 1.46)	0.39 (0.11 to 0.56)	–	0.09 (0.00 to 0.56)
		Poorer	0.71 (0.25 to 1.26)	0.85 (0.27 to 1.62)	0.43 (0.21 to 0.56)	–	0.04 (0.01 to 0.48)
		Middle	0.73 (0.25 to 1.35)	0.87 (0.27 to 1.73)	0.47 (0.22 to 0.62)	–	0.16 (0.01 to 0.76)
		Richer	0.63 (0.07 to 1.24)	0.72 (0.07 to 1.54)	0.45 (0.06 to 0.66)	0.01 (0.01 to 0.01)	0.22 (0.01 to 0.91)
		Richest	0.34 (-0.23 to 0.93)	0.37 (-0.22 to 1.12)	0.29 (-0.26 to 0.58)	0.13 (0.00 to 0.73)	0.41 (0.04 to 1.31)
	Ghana	Poorest	1.25 (0.80 to 1.66)	1.49 (0.90 to 2.09)	0.76 (0.60 to 0.85)	–	0.22 (0.02 to 0.84)
		Poorer	1.18 (0.77 to 1.53)	1.46 (0.88 to 1.95)	0.65 (0.55 to 0.71)	–	0.08 (0.00 to 0.39)
		Middle	1.02 (0.70 to 1.33)	1.24 (0.80 to 1.70)	0.57 (0.48 to 0.63)	–	0.08 (0.00 to 0.32)
		Richer	0.77 (0.47 to 1.02)	0.92 (0.53 to 1.27)	0.46 (0.35 to 0.54)	–	0.08 (0.00 to 0.34)
		Richest	0.41 (0.13 to 0.62)	0.46 (0.14 to 0.74)	0.29 (0.12 to 0.39)	–	0.12 (0.01 to 0.38)
	Guinea	Poorest	-3.55 (-4.46 to -2.41)	-3.37 (-4.20 to -2.32)	-3.90 (-4.99 to -2.58)	19.55 (17.00 to 21.98)	20.92 (18.35 to 23.36)
		Poorer	-2.77 (-3.59 to -1.74)	-2.62 (-3.35 to -1.68)	-3.09 (-4.08 to -1.87)	15.24 (13.28 to 17.46)	16.57 (14.60 to 18.81)
		Middle	-2.76 (-3.48 to -1.74)	-2.58 (-3.18 to -1.66)	-3.12 (-4.05 to -1.89)	13.89 (11.83 to 15.66)	15.20 (13.14 to 16.98)
		Richer	-3.13 (-3.86 to -2.35)	-2.83 (-3.45 to -2.17)	-3.74 (-4.72 to -2.70)	13.09 (11.43 to 15.08)	14.39 (12.72 to 16.39)
		Richest	-3.58 (-4.20 to -2.71)	-3.05 (-3.52 to -2.37)	-4.63 (-5.58 to -3.40)	12.36 (10.14 to 14.03)	13.63 (11.41 to 15.32)
	Kenya	Poorest	2.81 (2.41 to 3.17)	3.59 (3.02 to 4.18)	1.25 (1.17 to 1.34)	–	0.05 (0.00 to 0.27)
		Poorer	2.41 (2.12 to 2.72)	3.17 (2.73 to 3.64)	0.92 (0.86 to 0.97)	–	–
		Middle	1.98 (1.76 to 2.24)	2.60 (2.25 to 3.00)	0.74 (0.69 to 0.79)	–	–
		Richer	1.44 (1.22 to 1.64)	1.88 (1.55 to 2.19)	0.58 (0.53 to 0.64)	–	–
		Richest	0.84 (0.68 to 0.99)	1.06 (0.84 to 1.30)	0.39 (0.35 to 0.45)	–	–
	Lesotho	Poorest	0.59 (-0.24 to 1.34)	0.63 (-0.23 to 1.52)	0.52 (-0.25 to 0.96)	1.07 (0.04 to 2.73)	2.12 (0.92 to 3.90)
		Poorer	0.71 (0.10 to 1.35)	0.77 (0.10 to 1.57)	0.59 (0.10 to 0.88)	0.53 (0.03 to 1.51)	1.39 (0.41 to 2.58)
		Middle	0.61 (-0.04 to 1.22)	0.66 (-0.04 to 1.42)	0.51 (-0.04 to 0.81)	0.44 (0.02 to 1.58)	1.31 (0.41 to 2.50)
		Richer	0.33 (-0.31 to 0.94)	0.34 (-0.30 to 1.08)	0.30 (-0.34 to 0.66)	0.54 (0.03 to 1.65)	1.44 (0.48 to 2.77)
		Richest	-0.09 (-0.69 to 0.48)	-0.08 (-0.61 to 0.53)	-0.09 (-0.85 to 0.39)	0.64 (0.02 to 1.96)	1.58 (0.57 to 3.01)
	Liberia	Poorest	2.70 (1.99 to 3.46)	3.01 (2.15 to 3.96)	2.09 (1.68 to 2.48)	2.15 (1.32 to 3.20)	3.39 (2.55 to 4.45)
		Poorer	2.65 (2.03 to 3.31)	3.06 (2.26 to 3.94)	1.85 (1.56 to 2.09)	0.78 (0.14 to 1.54)	2.00 (1.35 to 2.77)
		Middle	2.30 (1.70 to 2.85)	2.66 (1.89 to 3.43)	1.59 (1.32 to 1.80)	0.43 (0.02 to 1.19)	1.62 (1.04 to 2.40)
		Richer	1.65 (1.12 to 2.15)	1.89 (1.24 to 2.52)	1.20 (0.91 to 1.42)	0.39 (0.03 to 0.94)	1.51 (0.91 to 2.14)
		Richest	0.85 (0.45 to 1.30)	0.94 (0.47 to 1.50)	0.68 (0.40 to 0.92)	0.30 (0.03 to 0.90)	1.37 (0.73 to 2.08)
	Madagascar	Poorest	3.83 (2.54 to 4.59)	4.72 (2.89 to 5.96)	2.02 (1.79 to 2.22)	0.49 (0.02 to 1.70)	0.77 (0.04 to 2.69)
		Poorer	2.97 (2.15 to 3.56)	3.79 (2.54 to 4.78)	1.31 (1.10 to 1.45)	0.15 (0.15 to 0.15)	0.27 (0.02 to 1.08)
		Middle	2.17 (1.56 to 2.63)	2.82 (1.84 to 3.56)	0.88 (0.73 to 1.00)	–	0.20 (0.00 to 0.67)
		Richer	1.34 (0.90 to 1.64)	1.72 (1.06 to 2.19)	0.56 (0.46 to 0.66)	–	0.12 (0.03 to 0.46)
		Richest	0.64 (0.37 to 0.82)	0.79 (0.42 to 1.08)	0.30 (0.25 to 0.36)	–	–
	Malawi	Poorest	1.28 (1.13 to 1.43)	1.58 (1.37 to 1.80)	0.69 (0.64 to 0.74)	–	–
		Poorer	1.18 (1.07 to 1.31)	1.49 (1.32 to 1.68)	0.57 (0.54 to 0.60)	–	–
		Middle	1.03 (0.93 to 1.13)	1.29 (1.14 to 1.45)	0.50 (0.48 to 0.53)	–	–
		Richer	0.77 (0.67 to 0.87)	0.94 (0.81 to 1.10)	0.41 (0.38 to 0.44)	–	–
		Richest	0.44 (0.36 to 0.52)	0.52 (0.41 to 0.63)	0.28 (0.25 to 0.32)	–	–
	Mali	Poorest	3.50 (3.21 to 3.84)	4.05 (3.67 to 4.51)	2.40 (2.27 to 2.55)	1.12 (0.59 to 1.70)	2.35 (1.81 to 2.94)

Indicators	Country	Wealth Quintile	Average Annual Rate of Change (95% CrI)			Added AARC Needed to Achieve Targets (95% CrI)	
			2000–2030	2000–2020	2020–2030	UHC2030	IA2030
		Poorer	3.37 (3.10 to 3.67)	4.03 (3.66 to 4.46)	2.07 (1.98 to 2.17)	0.16 (0.02 to 0.48)	1.30 (0.96 to 1.68)
		Middle	2.95 (2.71 to 3.33)	3.53 (3.21 to 4.07)	1.80 (1.74 to 1.87)	0.08 (0.01 to 0.35)	1.06 (0.71 to 1.36)
		Richer	2.30 (2.11 to 2.56)	2.73 (2.46 to 3.09)	1.47 (1.39 to 1.55)	0.07 (0.02 to 0.23)	1.00 (0.74 to 1.28)
		Richest	1.40 (1.18 to 1.61)	1.61 (1.33 to 1.90)	0.98 (0.87 to 1.06)	0.06 (0.00 to 0.19)	0.93 (0.61 to 1.26)
	Morocco	Poorest	0.72 (-1.16 to 1.18)	0.88 (-0.98 to 1.71)	0.28 (-1.51 to 0.43)	2.45 (0.28 to 12.29)	0.79 (0.05 to 6.05)
		Poorer	0.52 (-0.69 to 0.80)	0.66 (-0.59 to 1.16)	0.18 (-0.90 to 0.29)	1.18 (0.65 to 7.56)	0.66 (0.01 to 8.78)
		Middle	0.35 (-0.48 to 0.57)	0.44 (-0.41 to 0.80)	0.12 (-0.62 to 0.19)	0.48 (0.03 to 5.14)	1.15 (0.04 to 6.34)
		Richer	0.19 (-0.46 to 0.38)	0.24 (-0.37 to 0.54)	0.08 (-0.64 to 0.12)	0.70 (0.28 to 3.64)	0.94 (0.54 to 4.83)
		Richest	0.08 (-0.47 to 0.20)	0.10 (-0.33 to 0.26)	0.04 (-0.74 to 0.07)	0.41 (0.07 to 2.49)	0.62 (0.01 to 3.67)
	Mozambique	Poorest	1.08 (0.39 to 1.72)	1.15 (0.40 to 1.93)	0.95 (0.37 to 1.35)	2.44 (1.13 to 3.90)	3.66 (2.34 to 5.14)
		Poorer	1.06 (0.55 to 1.49)	1.17 (0.58 to 1.71)	0.85 (0.50 to 1.05)	0.58 (0.05 to 1.54)	1.75 (0.94 to 2.69)
		Middle	0.76 (0.35 to 1.15)	0.84 (0.36 to 1.33)	0.60 (0.32 to 0.77)	0.28 (0.01 to 0.81)	1.03 (0.43 to 1.84)
		Richer	0.37 (0.05 to 0.65)	0.40 (0.05 to 0.74)	0.32 (0.05 to 0.47)	0.11 (0.00 to 0.44)	0.66 (0.19 to 1.34)
		Richest	0.04 (-0.21 to 0.23)	0.04 (-0.20 to 0.25)	0.04 (-0.24 to 0.20)	0.10 (0.10 to 0.10)	0.37 (0.04 to 1.03)
	Namibia	Poorest	0.87 (0.48 to 1.20)	0.97 (0.51 to 1.41)	0.65 (0.41 to 0.79)	0.17 (0.00 to 0.69)	0.73 (0.20 to 1.48)
		Poorer	0.94 (0.55 to 1.21)	1.08 (0.59 to 1.46)	0.65 (0.47 to 0.74)	0.03 (0.03 to 0.31)	0.38 (0.03 to 1.04)
		Middle	0.87 (0.51 to 1.14)	0.99 (0.55 to 1.37)	0.62 (0.43 to 0.70)	0.05 (0.02 to 0.06)	0.40 (0.06 to 1.15)
		Richer	0.64 (0.30 to 0.92)	0.71 (0.32 to 1.07)	0.49 (0.27 to 0.62)	0.05 (0.00 to 0.38)	0.56 (0.06 to 1.24)
		Richest	0.25 (-0.09 to 0.52)	0.26 (-0.09 to 0.57)	0.22 (-0.10 to 0.40)	0.15 (0.03 to 0.66)	0.75 (0.17 to 1.64)
	Niger	Poorest	8.27 (7.52 to 9.22)	11.10 (9.75 to 12.63)	2.83 (2.41 to 3.26)	–	0.08 (0.01 to 0.51)
		Poorer	7.56 (6.89 to 8.39)	10.42 (9.25 to 11.82)	2.05 (1.74 to 2.34)	–	–
		Middle	6.74 (6.06 to 7.49)	9.36 (8.22 to 10.65)	1.68 (1.45 to 1.91)	–	–
		Richer	5.60 (5.09 to 6.36)	7.77 (6.92 to 9.03)	1.38 (1.18 to 1.59)	–	–
		Richest	4.05 (3.57 to 4.70)	5.59 (4.82 to 6.60)	1.03 (0.88 to 1.18)	–	–
	Nigeria	Poorest	7.66 (6.78 to 8.43)	8.07 (7.08 to 8.93)	6.86 (6.17 to 7.43)	11.56 (10.34 to 13.03)	12.96 (11.74 to 14.44)
		Poorer	7.22 (6.48 to 7.88)	8.02 (7.14 to 8.88)	5.64 (5.23 to 5.95)	4.83 (4.06 to 5.70)	6.13 (5.36 to 7.01)
		Middle	5.76 (5.23 to 6.33)	6.68 (5.97 to 7.48)	3.93 (3.74 to 4.12)	1.85 (1.33 to 2.42)	3.10 (2.58 to 3.68)
		Richer	3.84 (3.45 to 4.23)	4.59 (4.06 to 5.15)	2.34 (2.20 to 2.45)	0.25 (0.01 to 0.59)	1.43 (1.13 to 1.78)
		Richest	1.88 (1.57 to 2.18)	2.26 (1.85 to 2.69)	1.11 (1.02 to 1.19)	–	0.39 (0.18 to 0.65)
	Rwanda	Poorest	1.25 (1.10 to 1.39)	1.62 (1.39 to 1.84)	0.51 (0.46 to 0.57)	–	–
		Poorer	1.30 (1.17 to 1.45)	1.71 (1.50 to 1.93)	0.49 (0.44 to 0.53)	–	–
		Middle	1.31 (1.18 to 1.49)	1.71 (1.51 to 1.98)	0.51 (0.46 to 0.55)	–	–
		Richer	1.17 (1.03 to 1.32)	1.50 (1.28 to 1.72)	0.51 (0.47 to 0.55)	–	–
		Richest	0.83 (0.71 to 0.98)	1.04 (0.85 to 1.24)	0.44 (0.40 to 0.49)	–	–
	Sao Tome and Principe	Poorest	1.39 (-1.03 to 3.20)	1.74 (-0.92 to 4.49)	0.62 (-1.23 to 0.93)	1.00 (0.06 to 10.91)	1.27 (0.02 to 10.68)
		Poorer	1.31 (-0.70 to 2.85)	1.68 (-0.64 to 4.06)	0.52 (-0.83 to 0.74)	0.94 (0.03 to 13.46)	0.82 (0.06 to 9.36)
		Middle	1.17 (-0.72 to 2.48)	1.46 (-0.65 to 3.57)	0.43 (-0.87 to 0.64)	0.88 (0.00 to 12.25)	0.67 (0.01 to 8.58)
		Richer	0.83 (-0.75 to 2.10)	1.03 (-0.66 to 3.03)	0.36 (-0.94 to 0.58)	0.95 (0.04 to 12.84)	0.85 (0.07 to 9.18)
		Richest	0.46 (-1.07 to 1.50)	0.53 (-0.87 to 2.14)	0.23 (-1.45 to 0.50)	0.78 (0.01 to 12.02)	0.74 (0.02 to 8.75)
	Senegal	Poorest	2.35 (1.97 to 2.69)	2.92 (2.36 to 3.39)	1.22 (1.15 to 1.29)	–	0.15 (0.01 to 0.41)
		Poorer	2.14 (1.85 to 2.44)	2.73 (2.30 to 3.17)	0.97 (0.93 to 1.01)	–	–
		Middle	1.85 (1.61 to 2.12)	2.36 (2.00 to 2.76)	0.84 (0.81 to 0.88)	–	–
		Richer	1.40 (1.17 to 1.66)	1.75 (1.41 to 2.13)	0.69 (0.66 to 0.74)	–	–
		Richest	0.84 (0.68 to 1.03)	1.02 (0.81 to 1.27)	0.48 (0.43 to 0.53)	–	–
	Sierra Leone	Poorest	2.07 (1.58 to 2.65)	2.43 (1.80 to 3.21)	1.36 (1.15 to 1.58)	0.14 (0.01 to 0.54)	1.10 (0.66 to 1.59)
		Poorer	2.25 (1.74 to 2.91)	2.69 (2.01 to 3.61)	1.38 (1.18 to 1.55)	0.06 (0.01 to 0.21)	0.75 (0.39 to 1.22)
		Middle	2.24 (1.78 to 2.83)	2.66 (2.03 to 3.47)	1.41 (1.24 to 1.61)	0.09 (0.01 to 0.52)	0.93 (0.55 to 1.38)
		Richer	1.92 (1.34 to 2.58)	2.22 (1.50 to 3.09)	1.32 (1.04 to 1.56)	0.21 (0.01 to 0.70)	1.33 (0.86 to 1.86)
		Richest	1.26 (0.80 to 1.80)	1.40 (0.86 to 2.06)	0.97 (0.69 to 1.28)	0.62 (0.05 to 1.24)	1.81 (1.21 to 2.44)
	South Africa	Poorest	2.23 (-2.04 to 6.54)	2.56 (-1.72 to 8.59)	1.59 (-2.61 to 2.61)	1.48 (0.04 to 6.94)	2.04 (0.15 to 8.12)
		Poorer	2.50 (-1.68 to 6.47)	2.92 (-1.45 to 8.65)	1.63 (-2.14 to 2.40)	1.27 (0.03 to 6.23)	1.59 (0.17 to 6.93)
		Middle	2.51 (-1.88 to 6.32)	2.90 (-1.60 to 8.44)	1.72 (-2.37 to 2.54)	1.27 (0.05 to 6.35)	1.91 (0.18 to 7.42)
		Richer	2.13 (-2.26 to 6.08)	2.43 (-1.91 to 7.84)	1.57 (-2.99 to 2.71)	1.74 (0.03 to 7.54)	2.34 (0.21 to 8.64)
		Richest	1.38 (-2.81 to 5.18)	1.50 (-2.24 to 6.48)	1.14 (-3.95 to 2.62)	2.13 (0.18 to 8.59)	2.81 (0.36 to 9.71)
	Tanzania	Poorest	0.79 (0.41 to 1.20)	0.86 (0.43 to 1.36)	0.65 (0.37 to 0.88)	0.38 (0.04 to 1.03)	1.52 (0.88 to 2.21)
		Poorer	0.79 (0.49 to 1.12)	0.88 (0.53 to 1.32)	0.59 (0.42 to 0.72)	0.04 (0.04 to 0.04)	0.58 (0.19 to 1.00)
		Middle	0.58 (0.36 to 0.84)	0.65 (0.39 to 0.98)	0.44 (0.31 to 0.57)	–	0.33 (0.03 to 0.70)
		Richer	0.31 (0.14 to 0.55)	0.34 (0.14 to 0.63)	0.26 (0.13 to 0.38)	–	0.23 (0.01 to 0.55)
		Richest	0.05 (-0.11 to 0.22)	0.05 (-0.11 to 0.24)	0.05 (-0.12 to 0.18)	–	0.13 (0.01 to 0.42)
	Togo	Poorest	2.41 (-0.27 to 6.35)	2.92 (-0.26 to 9.00)	1.28 (-0.28 to 1.59)	1.11 (0.01 to 3.66)	0.99 (0.05 to 4.25)
		Poorer	2.36 (0.03 to 6.24)	2.91 (0.03 to 8.98)	1.12 (0.03 to 1.33)	0.77 (0.09 to 9.72)	0.72 (0.04 to 3.47)
		Middle	2.14 (-0.14 to 6.23)	2.64 (-0.13 to 8.94)	1.08 (-0.14 to 1.28)	0.70 (0.06 to 9.28)	0.68 (0.04 to 3.11)
		Richer	1.82 (-0.42 to 5.64)	2.23 (-0.39 to 8.08)	0.99 (-0.46 to 1.21)	0.73 (0.04 to 2.57)	0.75 (0.09 to 3.64)
		Richest	1.15 (-0.76 to 4.66)	1.32 (-0.67 to 6.64)	0.75 (-0.92 to 1.04)	0.85 (0.02 to 2.90)	0.80 (0.04 to 3.64)
	Uganda	Poorest	2.85 (2.50 to 3.22)	3.43 (2.96 to 3.93)	1.69 (1.58 to 1.82)	0.06 (0.00 to 0.26)	0.89 (0.57 to 1.27)
		Poorer	2.93 (2.60 to 3.18)	3.59 (3.15 to 3.97)	1.58 (1.52 to 1.66)	–	0.48 (0.27 to 0.77)
		Middle	2.83 (2.53 to 3.11)	3.46 (3.03 to 3.87)	1.57 (1.51 to 1.63)	–	0.55 (0.31 to 0.85)
		Richer	2.43 (2.14 to 2.72)	2.93 (2.51 to 3.31)	1.46 (1.38 to 1.55)	0.06 (0.00 to 0.08)	0.78 (0.46 to 1.15)
		Richest	1.68 (1.39 to 1.95)	1.95 (1.59 to 2.31)	1.14 (1.03 to 1.26)	0.11 (0.00 to 0.41)	1.01 (0.65 to 1.47)
	Zambia	Poorest	1.15 (0.88 to 1.40)	1.29 (0.96 to 1.64)	0.85 (0.69 to 0.96)	0.08 (0.00 to 0.24)	1.01 (0.65 to 1.37)
		Poorer	1.12 (0.89 to 1.33)	1.31 (1.01 to 1.58)	0.74 (0.66 to 0.81)	–	0.33 (0.12 to 0.66)
		Middle	0.91 (0.72 to 1.09)	1.06 (0.81 to 1.30)	0.62 (0.54 to 0.68)	–	0.19 (0.02 to 0.47)
		Richer	0.61 (0.45 to 0.75)	0.69 (0.49 to 0.88)	0.45 (0.36 to 0.51)	–	0.15 (0.03 to 0.42)
		Richest	0.24 (0.11 to 0.36)	0.26 (0.11 to 0.40)	0.21 (0.10 to 0.28)	–	0.13 (0.01 to 0.38)
	Zimbabwe	Poorest	3.38 (2.81 to 3.95)	4.41 (3.51 to 5.27)	1.39 (1.21 to 1.54)	–	0.09 (0.01 to 0.35)
		Poorer	3.17 (2.71 to 3.71)	4.22 (3.50 to 5.02)	1.13 (1.00 to 1.24)	–	–
		Middle	2.87 (2.43 to 3.37)	3.81 (3.12 to 4.62)	1.02 (0.92 to 1.12)	–	–
		Richer	2.38 (1.92 to 2.76)	3.13 (2.42 to 3.70)	0.90 (0.82 to 0.99)	–	–
		Richest	1.62 (1.21 to 1.98)	2.07 (1.49 to 2.62)	0.71 (0.63 to 0.79)	–	–
Polio3 immunization	Angola	Poorest	-1.89 (-8.04 to 4.78)	-1.81 (-6.69 to 5.36)	-2.05 (-10.93 to 3.62)	13.14 (3.55 to 26.32)	14.46 (4.83 to 27.69)
		Poorer	-1.37 (-6.68 to 3.63)	-1.29 (-5.14 to 4.18)	-1.53 (-9.98 to 2.53)	8.13 (1.36 to 20.39)	9.39 (2.59 to 21.70)
		Middle	-0.95 (-5.19 to 2.94)	-0.88 (-3.49 to 3.51)	-1.08 (-8.51 to 1.81)	4.49 (0.35 to 14.81)	5.57 (1.13 to 15.70)

Indicators	Country	Wealth Quintile	Average Annual Rate of Change (95% CrI)			Added AARC Needed to Achieve Targets (95% CrI)	
			2000–2030	2000–2020	2020–2030	UHC2030	IA2030
		Richer	-0.84 (-4.30 to 1.93)	-0.74 (-2.52 to 2.34)	-1.04 (-7.76 to 1.11)	2.81 (0.20 to 11.17)	3.57 (0.30 to 12.39)
		Richest	-0.59 (-2.83 to 0.87)	-0.48 (-1.41 to 1.06)	-0.81 (-5.78 to 0.50)	1.44 (0.05 to 6.98)	1.76 (0.09 to 7.94)
	Benin	Poorest	0.70 (0.33 to 1.13)	0.72 (0.34 to 1.21)	0.64 (0.32 to 0.96)	2.84 (2.02 to 3.52)	4.06 (3.24 to 4.75)
		Poorer	0.66 (0.32 to 0.97)	0.70 (0.33 to 1.05)	0.59 (0.31 to 0.82)	1.66 (1.09 to 2.27)	2.87 (2.30 to 3.48)
		Middle	0.67 (0.41 to 0.89)	0.71 (0.42 to 0.98)	0.58 (0.37 to 0.72)	0.72 (0.30 to 1.17)	1.92 (1.49 to 2.38)
		Richer	0.33 (0.09 to 0.55)	0.34 (0.09 to 0.59)	0.30 (0.09 to 0.46)	0.44 (0.06 to 0.95)	1.63 (1.20 to 2.14)
		Richest	0.02 (-0.16 to 0.20)	0.02 (-0.16 to 0.21)	0.02 (-0.17 to 0.18)	0.11 (0.01 to 0.38)	0.99 (0.63 to 1.41)
	Burkina Faso	Poorest	4.16 (3.76 to 4.63)	6.21 (5.62 to 6.96)	0.17 (0.12 to 0.22)	–	–
		Poorer	3.73 (3.39 to 4.08)	5.58 (5.06 to 6.12)	0.13 (0.10 to 0.18)	–	–
		Middle	3.46 (3.15 to 3.75)	5.17 (4.71 to 5.62)	0.11 (0.08 to 0.15)	–	–
		Richer	2.86 (2.57 to 3.17)	4.27 (3.82 to 4.75)	0.10 (0.07 to 0.13)	–	–
		Richest	1.92 (1.69 to 2.23)	2.86 (2.52 to 3.33)	0.07 (0.05 to 0.09)	–	–
	Burundi	Poorest	0.07 (-0.32 to 0.35)	0.07 (-0.26 to 0.42)	0.06 (-0.44 to 0.21)	–	0.19 (0.01 to 0.76)
		Poorer	0.08 (-0.25 to 0.33)	0.08 (-0.21 to 0.40)	0.07 (-0.32 to 0.19)	–	0.14 (0.03 to 0.58)
		Middle	0.09 (-0.21 to 0.32)	0.10 (-0.18 to 0.39)	0.08 (-0.27 to 0.18)	–	0.07 (0.00 to 0.15)
		Richer	0.04 (-0.26 to 0.26)	0.04 (-0.21 to 0.30)	0.04 (-0.36 to 0.16)	–	0.09 (0.00 to 0.28)
		Richest	-0.02 (-0.26 to 0.14)	-0.02 (-0.19 to 0.16)	-0.02 (-0.39 to 0.10)	–	0.06 (0.04 to 0.09)
	Cameroon	Poorest	-0.03 (-0.54 to 0.53)	-0.03 (-0.53 to 0.55)	-0.03 (-0.58 to 0.50)	4.41 (3.34 to 5.67)	5.65 (4.57 to 6.92)
		Poorer	0.03 (-0.45 to 0.45)	0.03 (-0.43 to 0.46)	0.03 (-0.48 to 0.42)	2.66 (1.82 to 3.68)	3.88 (3.03 to 4.90)
		Middle	0.15 (-0.25 to 0.44)	0.15 (-0.24 to 0.46)	0.14 (-0.26 to 0.39)	1.29 (0.73 to 2.05)	2.50 (1.93 to 3.26)
		Richer	-0.10 (-0.39 to 0.20)	-0.09 (-0.37 to 0.21)	-0.10 (-0.43 to 0.19)	0.70 (0.23 to 1.40)	1.89 (1.42 to 2.60)
		Richest	-0.23 (-0.45 to -0.03)	-0.22 (-0.40 to -0.03)	-0.26 (-0.56 to -0.03)	0.16 (0.01 to 0.60)	1.04 (0.60 to 1.55)
	Chad	Poorest	7.14 (6.34 to 8.09)	9.50 (8.35 to 10.93)	2.56 (2.31 to 2.83)	–	0.13 (0.01 to 0.30)
		Poorer	6.82 (6.12 to 7.65)	9.16 (8.07 to 10.43)	2.29 (2.07 to 2.49)	–	0.04 (0.02 to 0.12)
		Middle	6.67 (5.93 to 7.46)	9.05 (7.88 to 10.31)	2.07 (1.88 to 2.22)	–	–
		Richer	6.03 (5.28 to 6.73)	8.12 (7.00 to 9.24)	1.92 (1.73 to 2.10)	–	–
		Richest	4.70 (3.98 to 5.35)	6.36 (5.32 to 7.43)	1.46 (1.31 to 1.65)	–	–
	Comoros	Poorest	1.65 (-2.34 to 4.62)	1.91 (-1.95 to 6.31)	1.04 (-3.12 to 1.54)	1.63 (0.01 to 9.96)	1.50 (0.03 to 10.29)
		Poorer	1.39 (-2.07 to 3.97)	1.66 (-1.69 to 5.51)	0.85 (-2.82 to 1.16)	1.22 (0.08 to 9.89)	1.32 (0.08 to 8.38)
		Middle	1.22 (-1.76 to 3.50)	1.47 (-1.40 to 4.93)	0.70 (-2.41 to 0.94)	0.95 (0.07 to 6.96)	0.99 (0.03 to 6.27)
		Richer	0.85 (-1.74 to 2.86)	1.01 (-1.24 to 4.01)	0.52 (-2.73 to 0.75)	0.78 (0.03 to 6.54)	0.93 (0.03 to 6.33)
		Richest	0.42 (-1.43 to 1.87)	0.49 (-0.92 to 2.62)	0.29 (-2.48 to 0.51)	1.06 (0.00 to 4.78)	0.83 (0.01 to 5.69)
	Congo Brazzaville	Poorest	2.46 (1.73 to 3.28)	3.00 (1.98 to 4.22)	1.40 (1.21 to 1.56)	0.17 (0.04 to 0.42)	0.58 (0.05 to 1.48)
		Poorer	1.94 (1.36 to 2.55)	2.42 (1.59 to 3.33)	1.00 (0.90 to 1.08)	–	0.24 (0.02 to 0.71)
		Middle	1.54 (1.13 to 2.04)	1.95 (1.36 to 2.74)	0.73 (0.65 to 0.80)	–	0.08 (0.01 to 0.28)
		Richer	1.00 (0.72 to 1.35)	1.26 (0.85 to 1.80)	0.50 (0.42 to 0.57)	–	–
		Richest	0.48 (0.31 to 0.70)	0.60 (0.36 to 0.91)	0.26 (0.21 to 0.32)	–	–
	Congo Democratic Republic	Poorest	2.46 (1.78 to 3.22)	2.94 (2.03 to 4.03)	1.49 (1.27 to 1.64)	0.18 (0.01 to 0.67)	0.85 (0.26 to 1.72)
		Poorer	2.12 (1.48 to 2.72)	2.57 (1.69 to 3.43)	1.22 (1.04 to 1.31)	0.27 (0.12 to 0.41)	0.41 (0.04 to 1.07)
		Middle	1.90 (1.41 to 2.43)	2.35 (1.65 to 3.14)	1.02 (0.91 to 1.08)	–	0.17 (0.01 to 0.59)
		Richer	1.42 (0.96 to 1.82)	1.74 (1.11 to 2.34)	0.79 (0.67 to 0.87)	–	0.12 (0.01 to 0.61)
		Richest	0.81 (0.49 to 1.10)	0.97 (0.55 to 1.39)	0.48 (0.36 to 0.55)	–	0.05 (0.00 to 0.22)
	Cote d'Ivoire	Poorest	1.36 (-2.53 to 4.87)	1.47 (-2.24 to 6.35)	1.15 (-3.11 to 1.97)	3.07 (0.25 to 11.64)	3.48 (0.28 to 12.08)
		Poorer	1.16 (-2.18 to 4.26)	1.27 (-1.90 to 5.71)	0.95 (-2.73 to 1.58)	2.14 (0.11 to 9.20)	2.48 (0.13 to 10.32)
		Middle	1.11 (-1.81 to 3.77)	1.23 (-1.55 to 5.15)	0.84 (-2.29 to 1.29)	1.64 (0.09 to 7.99)	1.76 (0.08 to 7.54)
		Richer	0.71 (-1.86 to 3.10)	0.78 (-1.54 to 4.12)	0.57 (-2.50 to 1.06)	1.40 (0.04 to 6.97)	1.58 (0.07 to 6.99)
		Richest	0.30 (-1.81 to 1.99)	0.31 (-1.33 to 2.76)	0.25 (-2.64 to 0.68)	1.26 (0.06 to 6.41)	1.10 (0.01 to 5.92)
	Egypt	Poorest	-2.22 (-2.84 to -1.60)	-1.17 (-1.44 to -0.91)	-4.31 (-5.66 to -2.97)	4.66 (2.83 to 6.51)	5.85 (4.01 to 7.71)
		Poorer	-2.03 (-2.72 to -1.41)	-1.05 (-1.32 to -0.83)	-3.95 (-5.38 to -2.59)	4.07 (2.29 to 6.14)	5.25 (3.47 to 7.33)
		Middle	-1.83 (-2.43 to -1.35)	-0.97 (-1.19 to -0.77)	-3.53 (-4.86 to -2.51)	3.47 (2.08 to 5.22)	4.65 (3.26 to 6.41)
		Richer	-1.95 (-2.50 to -1.43)	-0.97 (-1.19 to -0.78)	-3.91 (-5.09 to -2.74)	3.80 (2.29 to 5.41)	4.98 (3.47 to 6.60)
		Richest	-1.81 (-2.36 to -1.32)	-0.81 (-1.01 to -0.64)	-3.77 (-5.07 to -2.64)	3.29 (1.85 to 4.92)	4.47 (3.02 to 6.11)
	Eswatini	Poorest	0.53 (-1.05 to 1.26)	0.69 (-0.86 to 1.84)	0.18 (-1.42 to 0.31)	0.97 (0.00 to 17.16)	1.08 (0.12 to 17.42)
		Poorer	0.46 (-0.76 to 1.04)	0.59 (-0.62 to 1.50)	0.15 (-1.10 to 0.25)	1.42 (0.17 to 14.93)	0.90 (0.01 to 16.18)
		Middle	0.41 (-0.57 to 0.86)	0.55 (-0.45 to 1.25)	0.13 (-0.82 to 0.21)	0.61 (0.07 to 12.80)	0.67 (0.06 to 14.03)
		Richer	0.30 (-0.64 to 0.70)	0.39 (-0.49 to 0.99)	0.10 (-0.94 to 0.18)	0.56 (0.00 to 12.89)	0.83 (0.03 to 14.12)
		Richest	0.17 (-0.57 to 0.45)	0.21 (-0.41 to 0.65)	0.06 (-0.90 to 0.12)	3.92 (0.46 to 11.66)	0.56 (0.08 to 12.87)
	Ethiopia	Poorest	4.68 (4.10 to 5.07)	5.46 (4.69 to 6.01)	3.13 (2.90 to 3.28)	1.34 (0.85 to 1.87)	2.58 (2.08 to 3.12)
		Poorer	4.18 (3.75 to 4.55)	4.99 (4.37 to 5.50)	2.60 (2.47 to 2.70)	0.47 (0.07 to 0.90)	1.69 (1.26 to 2.12)
		Middle	3.84 (3.50 to 4.15)	4.69 (4.16 to 5.13)	2.18 (2.09 to 2.26)	0.12 (0.01 to 0.25)	0.98 (0.70 to 1.34)
		Richer	3.03 (2.70 to 3.29)	3.69 (3.24 to 4.06)	1.73 (1.64 to 1.80)	–	0.75 (0.48 to 1.11)
		Richest	1.90 (1.67 to 2.13)	2.30 (1.99 to 2.64)	1.10 (1.02 to 1.17)	–	0.29 (0.11 to 0.60)
	Gabon	Poorest	2.47 (2.10 to 2.84)	3.18 (2.60 to 3.75)	1.07 (0.93 to 1.19)	–	0.05 (0.00 to 0.27)
		Poorer	2.22 (1.94 to 2.50)	2.87 (2.44 to 3.31)	0.91 (0.81 to 1.01)	–	0.02 (0.02 to 0.02)
		Middle	2.07 (1.84 to 2.35)	2.72 (2.35 to 3.14)	0.80 (0.69 to 0.93)	–	–
		Richer	1.67 (1.41 to 1.97)	2.16 (1.78 to 2.58)	0.68 (0.58 to 0.82)	–	–
		Richest	1.06 (0.88 to 1.29)	1.35 (1.10 to 1.66)	0.48 (0.38 to 0.59)	–	–
	Gambia	Poorest	-0.23 (-0.52 to 0.05)	-0.19 (-0.37 to 0.05)	-0.30 (-0.83 to 0.05)	–	0.24 (0.01 to 0.92)
		Poorer	-0.23 (-0.55 to 0.06)	-0.20 (-0.38 to 0.06)	-0.29 (-0.88 to 0.06)	–	0.27 (0.02 to 0.93)
		Middle	-0.23 (-0.55 to 0.11)	-0.20 (-0.40 to 0.11)	-0.28 (-0.84 to 0.10)	–	0.33 (0.01 to 1.08)
		Richer	-0.35 (-0.73 to 0.03)	-0.29 (-0.51 to 0.03)	-0.47 (-1.22 to 0.03)	0.22 (0.03 to 0.69)	0.63 (0.08 to 1.55)
		Richest	-0.43 (-0.83 to -0.07)	-0.33 (-0.55 to -0.07)	-0.61 (-1.37 to -0.08)	0.27 (0.00 to 1.02)	0.73 (0.10 to 1.85)
	Ghana	Poorest	0.73 (0.45 to 1.02)	0.89 (0.50 to 1.36)	0.39 (0.31 to 0.45)	–	0.14 (0.14 to 0.14)
		Poorer	0.65 (0.40 to 0.85)	0.80 (0.45 to 1.11)	0.33 (0.27 to 0.38)	–	–
		Middle	0.60 (0.36 to 0.81)	0.75 (0.43 to 1.06)	0.29 (0.24 to 0.34)	–	–
		Richer	0.45 (0.24 to 0.63)	0.56 (0.27 to 0.81)	0.24 (0.18 to 0.29)	–	–
		Richest	0.25 (0.11 to 0.38)	0.30 (0.12 to 0.48)	0.15 (0.09 to 0.20)	–	–
	Guinea	Poorest	-2.39 (-3.00 to -1.62)	-2.24 (-2.77 to -1.54)	-2.70 (-3.51 to -1.76)	12.50 (10.73 to 14.25)	13.81 (12.01 to 15.56)
		Poorer	-2.07 (-2.69 to -1.41)	-1.92 (-2.45 to -1.34)	-2.37 (-3.18 to -1.55)	10.26 (8.69 to 11.85)	11.53 (9.96 to 13.14)
		Middle	-1.69 (-2.33 to -1.05)	-1.56 (-2.09 to -0.99)	-1.95 (-2.80 to -1.15)	8.17 (6.78 to 9.60)	9.43 (8.03 to 10.87)
		Richer	-1.91 (-2.47 to -1.31)	-1.70 (-2.15 to -1.21)	-2.31 (-3.13 to -1.52)	7.51 (6.23 to 8.97)	8.76 (7.47 to 10.22)
		Richest	-1.86 (-2.39 to -1.41)	-1.56 (-1.94 to -1.23)	-2.47 (-3.26 to -1.76)	5.76 (4.54 to 7.12)	6.98 (5.75 to 8.35)

Indicators	Country	Wealth Quintile	Average Annual Rate of Change (95% CrI)			Added AARC Needed to Achieve Targets (95% CrI)	
			2000–2030	2000–2020	2020–2030	UHC2030	IA2030
	Kenya	Poorest	1.82 (1.49 to 2.19)	2.31 (1.84 to 2.87)	0.83 (0.77 to 0.91)	–	0.04 (0.03 to 0.04)
		Poorer	1.35 (1.11 to 1.64)	1.74 (1.38 to 2.20)	0.58 (0.53 to 0.62)	–	–
		Middle	1.05 (0.87 to 1.26)	1.37 (1.09 to 1.70)	0.40 (0.37 to 0.44)	–	–
		Richer	0.66 (0.53 to 0.83)	0.86 (0.67 to 1.10)	0.28 (0.25 to 0.32)	–	–
		Richest	0.32 (0.25 to 0.43)	0.41 (0.30 to 0.55)	0.15 (0.13 to 0.18)	–	–
	Lesotho	Poorest	0.72 (0.27 to 1.09)	0.84 (0.29 to 1.35)	0.48 (0.24 to 0.59)	0.38 (0.38 to 0.38)	0.22 (0.01 to 0.91)
		Poorer	0.64 (0.28 to 0.92)	0.75 (0.30 to 1.14)	0.41 (0.24 to 0.48)	–	0.12 (0.01 to 0.60)
		Middle	0.59 (0.26 to 0.85)	0.70 (0.27 to 1.08)	0.37 (0.22 to 0.42)	–	0.05 (0.00 to 0.77)
		Richer	0.42 (0.14 to 0.66)	0.49 (0.14 to 0.81)	0.28 (0.12 to 0.35)	–	0.09 (0.00 to 0.95)
		Richest	0.22 (-0.02 to 0.36)	0.24 (-0.02 to 0.43)	0.16 (-0.02 to 0.22)	–	0.19 (0.01 to 0.34)
	Liberia	Poorest	1.84 (1.24 to 2.51)	2.11 (1.36 to 2.97)	1.31 (1.00 to 1.57)	0.43 (0.02 to 1.08)	1.55 (0.89 to 2.27)
		Poorer	1.56 (1.03 to 2.10)	1.81 (1.14 to 2.55)	1.06 (0.82 to 1.21)	0.10 (0.00 to 0.50)	0.87 (0.42 to 1.48)
		Middle	1.40 (0.99 to 1.82)	1.65 (1.12 to 2.26)	0.88 (0.73 to 1.02)	–	0.36 (0.07 to 0.85)
		Richer	0.95 (0.61 to 1.30)	1.11 (0.68 to 1.57)	0.64 (0.48 to 0.79)	–	0.23 (0.04 to 0.67)
		Richest	0.48 (0.25 to 0.71)	0.55 (0.27 to 0.84)	0.35 (0.21 to 0.46)	–	0.12 (0.02 to 0.34)
	Madagascar	Poorest	3.58 (2.82 to 4.23)	4.59 (3.40 to 5.70)	1.53 (1.18 to 1.76)	0.60 (0.60 to 0.60)	0.24 (0.02 to 1.04)
		Poorer	2.70 (2.12 to 3.16)	3.52 (2.62 to 4.38)	0.99 (0.74 to 1.15)	–	0.13 (0.02 to 0.69)
		Middle	2.00 (1.54 to 2.41)	2.68 (1.93 to 3.38)	0.64 (0.47 to 0.76)	–	0.05 (0.05 to 0.05)
		Richer	1.25 (0.93 to 1.56)	1.67 (1.17 to 2.16)	0.41 (0.31 to 0.50)	–	–
		Richest	0.59 (0.40 to 0.78)	0.78 (0.49 to 1.06)	0.21 (0.16 to 0.25)	–	–
	Malawi	Poorest	0.81 (0.72 to 0.95)	1.04 (0.89 to 1.24)	0.38 (0.35 to 0.41)	–	–
		Poorer	0.72 (0.62 to 0.81)	0.91 (0.77 to 1.05)	0.32 (0.30 to 0.34)	–	–
		Middle	0.66 (0.59 to 0.75)	0.86 (0.75 to 0.99)	0.28 (0.26 to 0.30)	–	–
		Richer	0.51 (0.44 to 0.59)	0.64 (0.54 to 0.76)	0.24 (0.22 to 0.26)	–	–
		Richest	0.29 (0.24 to 0.36)	0.36 (0.28 to 0.46)	0.16 (0.14 to 0.18)	–	–
	Mali	Poorest	2.00 (1.73 to 2.31)	2.31 (1.97 to 2.75)	1.37 (1.25 to 1.47)	0.17 (0.02 to 0.62)	1.31 (0.92 to 1.81)
		Poorer	1.81 (1.55 to 2.03)	2.12 (1.78 to 2.43)	1.19 (1.11 to 1.26)	0.07 (0.03 to 0.43)	0.89 (0.58 to 1.27)
		Middle	1.72 (1.51 to 1.89)	2.05 (1.76 to 2.29)	1.07 (1.02 to 1.12)	–	0.52 (0.26 to 0.81)
		Richer	1.32 (1.13 to 1.51)	1.54 (1.30 to 1.80)	0.86 (0.80 to 0.93)	–	0.47 (0.24 to 0.76)
		Richest	0.75 (0.62 to 0.91)	0.86 (0.70 to 1.08)	0.53 (0.48 to 0.60)	–	0.21 (0.02 to 0.43)
	Morocco	Poorest	0.64 (-1.36 to 1.10)	0.80 (-1.10 to 1.58)	0.24 (-1.89 to 0.39)	1.03 (0.01 to 37.19)	1.26 (0.07 to 20.04)
		Poorer	0.43 (-0.98 to 0.73)	0.55 (-0.78 to 1.07)	0.17 (-1.39 to 0.25)	1.01 (0.03 to 30.72)	0.76 (0.08 to 28.33)
		Middle	0.31 (-0.70 to 0.55)	0.40 (-0.56 to 0.80)	0.11 (-1.00 to 0.18)	3.96 (0.62 to 24.43)	0.68 (0.06 to 25.73)
		Richer	0.18 (-0.56 to 0.36)	0.22 (-0.43 to 0.50)	0.07 (-0.83 to 0.13)	3.71 (0.82 to 21.84)	0.61 (0.03 to 23.11)
		Richest	0.08 (-0.45 to 0.18)	0.09 (-0.30 to 0.25)	0.03 (-0.73 to 0.07)	3.13 (1.27 to 16.37)	0.99 (0.03 to 17.59)
	Mozambique	Poorest	0.91 (0.22 to 1.37)	1.00 (0.23 to 1.60)	0.72 (0.21 to 0.92)	0.52 (0.03 to 1.87)	1.32 (0.46 to 2.72)
		Poorer	0.67 (0.25 to 0.99)	0.74 (0.26 to 1.18)	0.51 (0.23 to 0.62)	0.16 (0.02 to 0.76)	0.51 (0.03 to 1.45)
		Middle	0.52 (0.24 to 0.78)	0.59 (0.26 to 0.95)	0.37 (0.21 to 0.44)	–	0.12 (0.01 to 0.61)
		Richer	0.27 (0.06 to 0.46)	0.29 (0.06 to 0.55)	0.21 (0.05 to 0.28)	–	0.08 (0.00 to 0.38)
		Richest	0.09 (-0.03 to 0.20)	0.09 (-0.03 to 0.23)	0.07 (-0.03 to 0.13)	–	–
	Namibia	Poorest	0.78 (0.55 to 1.01)	0.95 (0.63 to 1.28)	0.42 (0.36 to 0.52)	–	–
		Poorer	0.71 (0.51 to 0.88)	0.88 (0.59 to 1.13)	0.38 (0.33 to 0.42)	–	–
		Middle	0.68 (0.53 to 0.81)	0.85 (0.62 to 1.04)	0.34 (0.31 to 0.38)	–	–
		Richer	0.53 (0.39 to 0.66)	0.65 (0.44 to 0.83)	0.30 (0.26 to 0.34)	–	–
		Richest	0.30 (0.19 to 0.41)	0.36 (0.21 to 0.50)	0.19 (0.15 to 0.23)	–	–
	Niger	Poorest	5.15 (4.56 to 5.94)	7.01 (5.96 to 8.31)	1.62 (1.34 to 1.85)	–	0.09 (0.09 to 0.09)
		Poorer	4.67 (4.04 to 5.37)	6.37 (5.36 to 7.50)	1.31 (1.10 to 1.49)	–	–
		Middle	4.31 (3.77 to 4.93)	5.95 (5.13 to 6.93)	1.09 (0.92 to 1.26)	–	–
		Richer	3.59 (3.09 to 4.16)	4.95 (4.15 to 5.85)	0.93 (0.80 to 1.07)	–	–
		Richest	2.46 (2.03 to 2.90)	3.38 (2.71 to 4.07)	0.65 (0.54 to 0.77)	–	–
	Nigeria	Poorest	4.55 (3.88 to 5.20)	4.86 (4.11 to 5.61)	3.92 (3.44 to 4.35)	7.31 (6.37 to 8.43)	8.63 (7.68 to 9.76)
		Poorer	3.90 (3.43 to 4.38)	4.32 (3.75 to 4.91)	3.07 (2.80 to 3.32)	3.64 (3.02 to 4.32)	4.90 (4.28 to 5.59)
		Middle	3.23 (2.87 to 3.62)	3.73 (3.25 to 4.23)	2.25 (2.09 to 2.41)	1.16 (0.77 to 1.56)	2.39 (1.99 to 2.79)
		Richer	2.04 (1.77 to 2.31)	2.38 (2.01 to 2.73)	1.37 (1.27 to 1.47)	0.09 (0.01 to 0.40)	1.21 (0.93 to 1.58)
		Richest	0.91 (0.78 to 1.10)	1.06 (0.88 to 1.31)	0.62 (0.56 to 0.69)	–	0.19 (0.03 to 0.39)
	Rwanda	Poorest	0.62 (0.44 to 0.79)	0.73 (0.49 to 0.97)	0.39 (0.33 to 0.45)	–	0.08 (0.02 to 0.11)
		Poorer	0.63 (0.47 to 0.82)	0.75 (0.54 to 1.02)	0.40 (0.34 to 0.44)	–	0.07 (0.02 to 0.12)
		Middle	0.70 (0.53 to 0.88)	0.84 (0.60 to 1.10)	0.42 (0.38 to 0.46)	–	–
		Richer	0.59 (0.43 to 0.79)	0.69 (0.48 to 0.97)	0.40 (0.33 to 0.45)	–	0.05 (0.00 to 0.18)
		Richest	0.38 (0.23 to 0.53)	0.42 (0.25 to 0.62)	0.28 (0.20 to 0.33)	–	0.04 (0.00 to 0.23)
	Sao Tome and Principe	Poorest	1.03 (-1.96 to 2.37)	1.26 (-1.54 to 3.45)	0.40 (-2.80 to 0.65)	2.27 (0.01 to 8.42)	1.12 (0.05 to 9.20)
		Poorer	0.89 (-1.67 to 2.20)	1.12 (-1.34 to 3.16)	0.35 (-2.32 to 0.52)	1.75 (0.03 to 7.17)	1.02 (0.19 to 7.46)
		Middle	0.82 (-1.32 to 2.14)	1.02 (-1.03 to 3.09)	0.31 (-1.89 to 0.48)	1.53 (0.08 to 5.95)	0.70 (0.02 to 5.97)
		Richer	0.62 (-1.07 to 1.63)	0.77 (-0.82 to 2.35)	0.25 (-1.61 to 0.40)	1.54 (0.02 to 6.70)	0.59 (0.04 to 5.77)
		Richest	0.33 (-1.17 to 1.12)	0.40 (-0.80 to 1.55)	0.15 (-1.74 to 0.30)	0.95 (0.17 to 5.73)	0.61 (0.01 to 5.11)
	Senegal	Poorest	1.31 (0.99 to 1.53)	1.57 (1.15 to 1.87)	0.80 (0.69 to 0.87)	–	0.15 (0.01 to 0.47)
		Poorer	1.14 (0.87 to 1.35)	1.37 (1.01 to 1.68)	0.67 (0.59 to 0.72)	–	0.04 (0.01 to 0.22)
		Middle	1.06 (0.84 to 1.23)	1.29 (1.00 to 1.54)	0.59 (0.54 to 0.62)	–	0.04 (0.04 to 0.04)
		Richer	0.78 (0.61 to 0.93)	0.94 (0.71 to 1.14)	0.47 (0.41 to 0.51)	–	–
		Richest	0.43 (0.29 to 0.54)	0.50 (0.33 to 0.65)	0.28 (0.22 to 0.32)	–	–
	Sierra Leone	Poorest	1.33 (0.90 to 1.80)	1.55 (1.00 to 2.18)	0.89 (0.69 to 1.04)	0.38 (0.38 to 0.38)	0.56 (0.24 to 1.05)
		Poorer	1.34 (0.98 to 1.71)	1.57 (1.11 to 2.09)	0.89 (0.73 to 1.03)	0.28 (0.28 to 0.28)	0.53 (0.18 to 0.95)
		Middle	1.47 (1.04 to 1.86)	1.74 (1.17 to 2.25)	0.94 (0.76 to 1.07)	0.11 (0.01 to 0.21)	0.45 (0.17 to 0.87)
		Richer	1.25 (0.86 to 1.65)	1.44 (0.96 to 1.99)	0.86 (0.65 to 1.03)	0.11 (0.01 to 0.43)	0.71 (0.33 to 1.21)
		Richest	0.81 (0.44 to 1.16)	0.91 (0.47 to 1.36)	0.61 (0.38 to 0.78)	0.15 (0.10 to 0.32)	0.64 (0.29 to 1.14)
	South Africa	Poorest	0.91 (-0.61 to 3.75)	1.10 (-0.54 to 5.27)	0.56 (-0.76 to 0.94)	0.88 (0.04 to 2.50)	0.52 (0.03 to 2.97)
		Poorer	0.87 (-0.47 to 3.70)	1.05 (-0.40 to 5.20)	0.52 (-0.59 to 0.82)	0.81 (0.07 to 2.23)	0.45 (0.00 to 2.40)
		Middle	0.87 (-0.36 to 3.86)	1.05 (-0.32 to 5.45)	0.49 (-0.43 to 0.78)	0.38 (0.07 to 2.20)	0.44 (0.01 to 1.78)
		Richer	0.67 (-0.49 to 3.35)	0.79 (-0.43 to 4.83)	0.42 (-0.60 to 0.77)	0.25 (0.09 to 2.75)	0.43 (0.03 to 1.91)
		Richest	0.39 (-0.55 to 2.56)	0.45 (-0.40 to 3.58)	0.26 (-0.76 to 0.65)	0.27 (0.06 to 2.99)	0.43 (0.07 to 1.55)
	Tanzania	Poorest	0.87 (0.51 to 1.23)	0.98 (0.56 to 1.47)	0.63 (0.43 to 0.76)	0.03 (0.00 to 0.17)	0.45 (0.06 to 1.11)
		Poorer	0.68 (0.41 to 0.92)	0.79 (0.45 to 1.12)	0.47 (0.34 to 0.54)	–	0.14 (0.01 to 0.47)

Indicators	Country	Wealth Quintile	Average Annual Rate of Change (95% CrI)			Added AARC Needed to Achieve Targets (95% CrI)	
			2000–2030	2000–2020	2020–2030	UHC2030	IA2030
		Middle	0.55 (0.36 to 0.73)	0.65 (0.40 to 0.89)	0.36 (0.28 to 0.41)	–	0.05 (0.01 to 0.09)
		Richer	0.33 (0.18 to 0.46)	0.38 (0.19 to 0.56)	0.23 (0.15 to 0.28)	–	–
		Richest	0.14 (0.04 to 0.21)	0.15 (0.04 to 0.25)	0.11 (0.04 to 0.14)	–	–
	Togo	Poorest	2.02 (-0.45 to 5.09)	2.47 (-0.43 to 7.24)	1.05 (-0.49 to 1.33)	0.74 (0.08 to 3.77)	0.94 (0.01 to 3.88)
		Poorer	1.88 (-0.30 to 4.91)	2.32 (-0.29 to 7.02)	0.97 (-0.32 to 1.17)	0.54 (0.02 to 3.07)	0.86 (0.04 to 3.93)
		Middle	1.82 (-0.18 to 4.89)	2.26 (-0.18 to 7.00)	0.89 (-0.19 to 1.08)	0.63 (0.00 to 2.97)	0.71 (0.05 to 2.87)
		Richer	1.52 (-0.42 to 4.28)	1.86 (-0.40 to 6.10)	0.80 (-0.48 to 1.03)	0.63 (0.03 to 3.54)	0.70 (0.06 to 3.04)
		Richest	1.00 (-0.58 to 3.16)	1.20 (-0.51 to 4.48)	0.56 (-0.70 to 0.80)	0.49 (0.02 to 2.73)	0.72 (0.05 to 2.44)
	Uganda	Poorest	1.83 (1.58 to 2.10)	2.27 (1.93 to 2.68)	0.95 (0.89 to 1.02)	–	0.04 (0.00 to 0.21)
		Poorer	1.76 (1.58 to 1.97)	2.20 (1.94 to 2.51)	0.89 (0.85 to 0.94)	–	0.02 (0.00 to 0.07)
		Middle	1.78 (1.58 to 2.01)	2.24 (1.95 to 2.59)	0.87 (0.82 to 0.90)	–	–
		Richer	1.53 (1.34 to 1.74)	1.89 (1.64 to 2.19)	0.80 (0.74 to 0.85)	–	0.03 (0.00 to 0.08)
		Richest	1.02 (0.87 to 1.20)	1.24 (1.03 to 1.49)	0.58 (0.53 to 0.64)	–	–
	Zambia	Poorest	0.47 (0.27 to 0.62)	0.53 (0.29 to 0.71)	0.36 (0.23 to 0.44)	–	0.12 (0.01 to 0.42)
		Poorer	0.43 (0.25 to 0.57)	0.48 (0.27 to 0.66)	0.32 (0.21 to 0.38)	–	0.09 (0.01 to 0.22)
		Middle	0.42 (0.27 to 0.53)	0.48 (0.30 to 0.62)	0.30 (0.22 to 0.35)	–	–
		Richer	0.28 (0.16 to 0.38)	0.31 (0.17 to 0.43)	0.22 (0.14 to 0.27)	–	0.03 (0.03 to 0.03)
		Richest	0.12 (0.03 to 0.19)	0.13 (0.03 to 0.21)	0.10 (0.03 to 0.15)	–	–
	Zimbabwe	Poorest	1.99 (1.58 to 2.55)	2.56 (1.92 to 3.39)	0.90 (0.80 to 1.02)	–	0.11 (0.00 to 0.33)
		Poorer	1.82 (1.43 to 2.24)	2.34 (1.76 to 3.00)	0.78 (0.72 to 0.85)	–	0.08 (0.08 to 0.08)
		Middle	1.71 (1.33 to 2.10)	2.23 (1.67 to 2.84)	0.69 (0.62 to 0.76)	–	–
		Richer	1.37 (1.05 to 1.73)	1.76 (1.29 to 2.28)	0.60 (0.53 to 0.66)	–	0.04 (0.04 to 0.04)
		Richest	0.86 (0.61 to 1.10)	1.09 (0.73 to 1.45)	0.41 (0.35 to 0.48)	–	–
DPT3 immunization	Angola	Poorest	-0.83 (-7.95 to 5.96)	-0.82 (-6.76 to 6.81)	-0.86 (-10.47 to 4.29)	12.08 (2.73 to 26.75)	13.40 (4.00 to 28.13)
		Poorer	-0.30 (-6.19 to 5.66)	-0.30 (-4.85 to 6.88)	-0.31 (-8.80 to 3.25)	7.07 (0.83 to 20.07)	8.18 (1.64 to 20.42)
		Middle	-0.46 (-5.52 to 4.40)	-0.45 (-4.01 to 5.40)	-0.48 (-8.49 to 2.48)	5.34 (0.37 to 17.28)	6.35 (1.05 to 17.31)
		Richer	-0.48 (-4.58 to 3.27)	-0.46 (-2.99 to 4.07)	-0.52 (-7.69 to 1.67)	3.20 (0.15 to 12.82)	4.10 (0.41 to 14.03)
		Richest	-0.66 (-3.79 to 1.78)	-0.59 (-2.19 to 2.17)	-0.80 (-7.18 to 0.95)	2.35 (0.12 to 10.02)	2.78 (0.25 to 11.24)
	Benin	Poorest	-0.05 (-0.41 to 0.28)	-0.05 (-0.40 to 0.29)	-0.05 (-0.44 to 0.27)	2.52 (1.79 to 3.29)	3.74 (3.00 to 4.50)
		Poorer	0.21 (-0.03 to 0.47)	0.21 (-0.03 to 0.51)	0.20 (-0.03 to 0.41)	0.71 (0.16 to 1.17)	1.90 (1.35 to 2.37)
		Middle	0.03 (-0.19 to 0.29)	0.04 (-0.18 to 0.31)	0.03 (-0.20 to 0.26)	0.33 (0.02 to 0.77)	1.48 (0.96 to 1.95)
		Richer	-0.05 (-0.22 to 0.12)	-0.05 (-0.21 to 0.12)	-0.05 (-0.25 to 0.11)	0.08 (0.03 to 0.19)	0.84 (0.47 to 1.27)
		Richest	-0.19 (-0.33 to -0.07)	-0.18 (-0.29 to -0.07)	-0.22 (-0.41 to -0.07)	–	0.43 (0.14 to 0.80)
	Burkina Faso	Poorest	3.73 (3.27 to 4.24)	5.62 (4.90 to 6.40)	0.05 (0.03 to 0.07)	–	–
		Poorer	3.24 (2.94 to 3.67)	4.89 (4.42 to 5.54)	0.03 (0.02 to 0.04)	–	–
		Middle	2.76 (2.46 to 3.10)	4.15 (3.69 to 4.68)	0.03 (0.02 to 0.04)	–	–
		Richer	2.07 (1.76 to 2.37)	3.11 (2.64 to 3.56)	0.02 (0.01 to 0.03)	–	–
		Richest	1.30 (1.07 to 1.53)	1.96 (1.60 to 2.30)	0.01 (0.01 to 0.02)	–	–
	Burundi	Poorest	0.35 (0.10 to 0.78)	0.44 (0.11 to 1.08)	0.16 (0.08 to 0.22)	–	–
		Poorer	0.31 (0.12 to 0.66)	0.40 (0.13 to 0.93)	0.12 (0.08 to 0.15)	–	–
		Middle	0.25 (0.08 to 0.55)	0.31 (0.09 to 0.77)	0.10 (0.06 to 0.13)	–	–
		Richer	0.17 (0.05 to 0.38)	0.22 (0.05 to 0.53)	0.08 (0.04 to 0.10)	–	–
		Richest	0.09 (0.01 to 0.23)	0.11 (0.01 to 0.32)	0.05 (0.01 to 0.07)	–	–
	Cameroon	Poorest	-0.09 (-0.57 to 0.30)	-0.09 (-0.53 to 0.31)	-0.09 (-0.63 to 0.28)	1.96 (1.10 to 2.95)	3.17 (2.30 to 4.17)
		Poorer	0.14 (-0.13 to 0.46)	0.15 (-0.13 to 0.50)	0.14 (-0.14 to 0.40)	0.43 (0.07 to 0.96)	1.61 (1.03 to 2.16)
		Middle	0.03 (-0.25 to 0.28)	0.03 (-0.23 to 0.30)	0.03 (-0.27 to 0.25)	0.21 (0.03 to 0.62)	1.22 (0.81 to 1.76)
		Richer	-0.08 (-0.27 to 0.14)	-0.08 (-0.25 to 0.15)	-0.08 (-0.31 to 0.13)	0.05 (0.01 to 0.06)	0.71 (0.35 to 1.17)
		Richest	-0.19 (-0.35 to -0.05)	-0.18 (-0.29 to -0.05)	-0.23 (-0.45 to -0.05)	–	0.36 (0.05 to 0.75)
	Chad	Poorest	3.20 (2.45 to 4.00)	3.67 (2.72 to 4.69)	2.27 (1.92 to 2.62)	1.41 (0.64 to 2.32)	2.64 (1.87 to 3.56)
		Poorer	3.19 (2.62 to 3.84)	3.75 (3.00 to 4.68)	2.06 (1.84 to 2.28)	0.39 (0.03 to 0.99)	1.55 (0.98 to 2.18)
		Middle	2.81 (2.25 to 3.45)	3.31 (2.56 to 4.17)	1.84 (1.63 to 2.00)	0.37 (0.01 to 1.07)	1.50 (0.95 to 2.27)
		Richer	2.23 (1.77 to 2.77)	2.61 (2.01 to 3.37)	1.47 (1.30 to 1.60)	0.20 (0.02 to 0.75)	1.16 (0.67 to 1.85)
		Richest	1.41 (1.02 to 1.80)	1.61 (1.13 to 2.16)	0.99 (0.81 to 1.13)	0.13 (0.00 to 0.53)	0.97 (0.47 to 1.60)
	Comoros	Poorest	1.44 (-1.58 to 5.25)	1.68 (-1.37 to 7.52)	0.88 (-1.98 to 1.24)	1.29 (0.09 to 5.95)	1.44 (0.06 to 7.08)
		Poorer	1.33 (-0.92 to 4.91)	1.62 (-0.80 to 7.14)	0.70 (-1.17 to 0.92)	0.67 (0.07 to 4.56)	0.98 (0.04 to 4.64)
		Middle	1.10 (-1.02 to 4.18)	1.33 (-0.87 to 6.05)	0.58 (-1.31 to 0.79)	0.82 (0.03 to 6.76)	0.87 (0.06 to 4.57)
		Richer	0.75 (-0.86 to 3.09)	0.91 (-0.72 to 4.43)	0.43 (-1.14 to 0.60)	0.58 (0.06 to 7.83)	0.63 (0.05 to 4.19)
		Richest	0.38 (-1.02 to 2.16)	0.44 (-0.70 to 3.13)	0.26 (-1.48 to 0.43)	0.50 (0.07 to 6.63)	0.50 (0.01 to 4.82)
	Congo Brazzaville	Poorest	-1.54 (-2.99 to -0.58)	-1.34 (-2.37 to -0.54)	-1.94 (-4.27 to -0.64)	5.08 (2.88 to 8.91)	6.31 (4.09 to 10.15)
		Poorer	-0.98 (-2.22 to -0.23)	-0.86 (-1.76 to -0.22)	-1.20 (-3.14 to -0.25)	3.02 (1.23 to 6.25)	4.22 (2.42 to 7.47)
		Middle	-1.03 (-2.25 to -0.32)	-0.90 (-1.73 to -0.30)	-1.31 (-3.28 to -0.36)	2.79 (1.02 to 6.04)	4.00 (2.22 to 7.26)
		Richer	-1.02 (-2.12 to -0.36)	-0.85 (-1.57 to -0.33)	-1.36 (-3.22 to -0.41)	2.26 (0.54 to 5.40)	3.45 (1.63 to 6.61)
		Richest	-1.06 (-2.03 to -0.44)	-0.81 (-1.36 to -0.38)	-1.55 (-3.43 to -0.57)	1.82 (0.21 to 4.55)	2.99 (1.21 to 5.74)
	Congo Democratic Republic	Poorest	4.89 (4.15 to 5.81)	6.67 (5.54 to 8.09)	1.41 (1.22 to 1.59)	–	–
		Poorer	4.28 (3.64 to 5.08)	5.98 (4.99 to 7.25)	0.99 (0.84 to 1.10)	–	–
		Middle	3.65 (3.09 to 4.34)	5.10 (4.22 to 6.21)	0.82 (0.70 to 0.93)	–	–
		Richer	2.74 (2.27 to 3.29)	3.83 (3.08 to 4.71)	0.60 (0.49 to 0.68)	–	–
		Richest	1.72 (1.39 to 2.17)	2.38 (1.87 to 3.04)	0.41 (0.33 to 0.48)	–	–
	Cote d'Ivoire	Poorest	1.67 (-1.50 to 4.05)	2.04 (-1.31 to 5.73)	0.87 (-2.00 to 1.10)	1.18 (0.14 to 7.45)	1.07 (0.03 to 8.15)
		Poorer	1.56 (-1.02 to 3.59)	1.95 (-0.87 to 5.16)	0.70 (-1.32 to 0.84)	0.61 (0.02 to 4.87)	0.74 (0.04 to 5.51)
		Middle	1.30 (-1.10 to 3.10)	1.61 (-0.93 to 4.46)	0.60 (-1.44 to 0.73)	1.11 (0.01 to 4.73)	0.71 (0.00 to 5.77)
		Richer	0.94 (-1.05 to 2.42)	1.14 (-0.84 to 3.48)	0.44 (-1.45 to 0.58)	2.02 (0.07 to 3.68)	0.64 (0.01 to 4.59)
		Richest	0.50 (-1.12 to 1.54)	0.61 (-0.82 to 2.16)	0.28 (-1.71 to 0.41)	1.63 (0.01 to 3.37)	0.59 (0.01 to 4.00)
	Egypt	Poorest	0.15 (0.12 to 0.20)	0.20 (0.15 to 0.26)	0.06 (0.05 to 0.08)	–	–
		Poorer	0.16 (0.13 to 0.20)	0.21 (0.16 to 0.28)	0.06 (0.05 to 0.07)	–	–
		Middle	0.16 (0.12 to 0.19)	0.21 (0.15 to 0.26)	0.06 (0.05 to 0.07)	–	–
		Richer	0.13 (0.10 to 0.16)	0.17 (0.12 to 0.22)	0.06 (0.05 to 0.07)	–	–
		Richest	0.09 (0.06 to 0.11)	0.11 (0.06 to 0.15)	0.04 (0.04 to 0.06)	–	–
	Eswatini	Poorest	0.35 (-0.19 to 0.85)	0.49 (-0.16 to 1.24)	0.09 (-0.24 to 0.17)	0.32 (0.20 to 0.44)	0.56 (0.03 to 1.61)
		Poorer	0.38 (-0.14 to 0.86)	0.51 (-0.13 to 1.28)	0.08 (-0.17 to 0.15)	–	0.56 (0.19 to 0.87)
		Middle	0.37 (-0.21 to 0.85)	0.51 (-0.19 to 1.26)	0.09 (-0.26 to 0.15)	–	0.67 (0.07 to 0.95)
		Richer	0.31 (-0.24 to 0.71)	0.43 (-0.21 to 1.05)	0.08 (-0.31 to 0.14)	0.18 (0.18 to 0.18)	0.37 (0.19 to 1.35)

Indicators	Country	Wealth Quintile	Average Annual Rate of Change (95% CrI)			Added AARC Needed to Achieve Targets (95% CrI)	
			2000–2030	2000–2020	2020–2030	UHC2030	IA2030
		Richest	0.21 (-0.33 to 0.56)	0.29 (-0.26 to 0.80)	0.06 (-0.46 to 0.12)	0.33 (0.33 to 0.33)	0.41 (0.25 to 1.50)
	Ethiopia	Poorest	3.46 (3.05 to 3.83)	3.98 (3.46 to 4.46)	2.40 (2.23 to 2.55)	1.25 (0.84 to 1.77)	2.47 (2.06 to 3.00)
		Poorer	3.36 (3.03 to 3.64)	4.00 (3.54 to 4.42)	2.06 (1.96 to 2.15)	0.15 (0.00 to 0.50)	1.29 (0.97 to 1.69)
		Middle	2.86 (2.59 to 3.18)	3.42 (3.04 to 3.88)	1.77 (1.69 to 1.85)	0.10 (0.01 to 0.37)	1.12 (0.81 to 1.49)
		Richer	2.20 (1.89 to 2.46)	2.63 (2.21 to 3.00)	1.35 (1.26 to 1.43)	0.06 (0.06 to 0.06)	0.75 (0.46 to 1.08)
		Richest	1.33 (1.13 to 1.53)	1.55 (1.30 to 1.81)	0.87 (0.79 to 0.95)	–	0.50 (0.26 to 0.80)
	Gabon	Poorest	2.27 (1.60 to 3.00)	2.55 (1.73 to 3.49)	1.70 (1.32 to 1.97)	1.33 (0.43 to 2.66)	2.55 (1.64 to 3.89)
		Poorer	2.64 (2.02 to 3.23)	3.02 (2.24 to 3.84)	1.87 (1.59 to 2.07)	0.91 (0.20 to 2.14)	2.13 (1.38 to 3.36)
		Middle	2.64 (1.92 to 3.18)	2.98 (2.08 to 3.71)	1.95 (1.59 to 2.19)	1.61 (0.60 to 3.00)	2.84 (1.82 to 4.24)
		Richer	2.34 (1.66 to 2.95)	2.61 (1.78 to 3.40)	1.82 (1.42 to 2.13)	1.95 (0.77 to 3.46)	3.19 (1.98 to 4.71)
		Richest	1.56 (0.81 to 2.23)	1.69 (0.84 to 2.49)	1.31 (0.75 to 1.74)	2.45 (1.17 to 4.43)	3.68 (2.38 to 5.68)
	Gambia	Poorest	0.04 (-0.22 to 0.30)	0.05 (-0.18 to 0.36)	0.04 (-0.30 to 0.17)	–	0.01 (0.01 to 0.38)
		Poorer	0.09 (-0.15 to 0.33)	0.09 (-0.12 to 0.40)	0.07 (-0.20 to 0.17)	–	0.02 (0.02 to 0.02)
		Middle	0.07 (-0.19 to 0.33)	0.07 (-0.15 to 0.40)	0.06 (-0.26 to 0.18)	–	0.02 (0.02 to 0.02)
		Richer	0.04 (-0.23 to 0.26)	0.04 (-0.18 to 0.31)	0.04 (-0.33 to 0.17)	–	0.02 (0.01 to 0.07)
		Richest	-0.03 (-0.28 to 0.17)	-0.03 (-0.21 to 0.20)	-0.03 (-0.41 to 0.12)	–	0.11 (0.01 to 0.24)
	Ghana	Poorest	0.80 (0.50 to 1.09)	1.02 (0.59 to 1.46)	0.37 (0.31 to 0.43)	–	–
		Poorer	0.73 (0.50 to 0.97)	0.94 (0.61 to 1.31)	0.29 (0.25 to 0.34)	–	–
		Middle	0.62 (0.39 to 0.84)	0.79 (0.45 to 1.13)	0.26 (0.22 to 0.31)	–	–
		Richer	0.45 (0.28 to 0.63)	0.57 (0.33 to 0.84)	0.20 (0.16 to 0.24)	–	–
		Richest	0.25 (0.14 to 0.39)	0.31 (0.17 to 0.52)	0.14 (0.10 to 0.17)	–	–
	Guinea	Poorest	-1.08 (-1.73 to -0.28)	-1.03 (-1.62 to -0.27)	-1.17 (-1.96 to -0.28)	7.81 (6.17 to 9.31)	9.07 (7.43 to 10.58)
		Poorer	-0.49 (-0.99 to 0.15)	-0.48 (-0.93 to 0.15)	-0.52 (-1.11 to 0.14)	4.80 (3.44 to 5.84)	6.04 (4.67 to 7.08)
		Middle	-0.63 (-1.12 to -0.05)	-0.60 (-1.04 to -0.05)	-0.69 (-1.30 to -0.06)	4.13 (2.88 to 5.26)	5.36 (4.09 to 6.49)
		Richer	-0.69 (-1.15 to -0.24)	-0.64 (-1.03 to -0.23)	-0.79 (-1.40 to -0.25)	2.93 (1.96 to 4.06)	4.14 (3.16 to 5.27)
		Richest	-0.86 (-1.25 to -0.48)	-0.74 (-1.02 to -0.45)	-1.10 (-1.66 to -0.55)	2.14 (1.19 to 2.98)	3.34 (2.38 to 4.18)
	Kenya	Poorest	1.70 (1.40 to 2.01)	2.31 (1.87 to 2.79)	0.48 (0.42 to 0.54)	–	–
		Poorer	1.41 (1.18 to 1.61)	1.95 (1.60 to 2.28)	0.33 (0.29 to 0.37)	–	–
		Middle	1.12 (0.94 to 1.29)	1.54 (1.26 to 1.82)	0.27 (0.23 to 0.31)	–	–
		Richer	0.75 (0.63 to 0.90)	1.03 (0.84 to 1.27)	0.19 (0.16 to 0.23)	–	–
		Richest	0.42 (0.33 to 0.51)	0.57 (0.43 to 0.71)	0.13 (0.10 to 0.16)	–	–
	Lesotho	Poorest	0.21 (-0.38 to 0.66)	0.22 (-0.35 to 0.76)	0.19 (-0.43 to 0.44)	0.26 (0.02 to 2.10)	0.60 (0.07 to 2.02)
		Poorer	0.31 (-0.18 to 0.73)	0.34 (-0.17 to 0.90)	0.25 (-0.19 to 0.43)	0.30 (0.03 to 0.74)	0.31 (0.02 to 1.39)
		Middle	0.23 (-0.25 to 0.65)	0.24 (-0.23 to 0.77)	0.20 (-0.28 to 0.41)	0.23 (0.09 to 1.07)	0.34 (0.02 to 1.35)
		Richer	0.13 (-0.28 to 0.50)	0.14 (-0.26 to 0.59)	0.12 (-0.32 to 0.34)	0.10 (0.01 to 0.37)	0.31 (0.02 to 1.19)
		Richest	-0.04 (-0.51 to 0.29)	-0.04 (-0.41 to 0.32)	-0.04 (-0.70 to 0.23)	0.20 (0.09 to 0.54)	0.31 (0.01 to 1.37)
	Liberia	Poorest	2.17 (1.55 to 2.68)	2.60 (1.79 to 3.32)	1.30 (1.08 to 1.43)	0.14 (0.05 to 0.33)	0.66 (0.22 to 1.20)
		Poorer	2.01 (1.52 to 2.42)	2.48 (1.79 to 3.09)	1.05 (0.94 to 1.14)	–	0.16 (0.01 to 0.47)
		Middle	1.67 (1.27 to 2.06)	2.04 (1.49 to 2.64)	0.90 (0.81 to 1.00)	–	0.13 (0.01 to 0.44)
		Richer	1.22 (0.86 to 1.61)	1.50 (1.00 to 2.04)	0.67 (0.57 to 0.78)	–	0.06 (0.00 to 0.31)
		Richest	0.70 (0.41 to 0.99)	0.83 (0.45 to 1.21)	0.43 (0.32 to 0.53)	–	0.03 (0.00 to 0.16)
	Madagascar	Poorest	2.70 (1.76 to 3.52)	3.32 (1.99 to 4.67)	1.45 (1.24 to 1.60)	0.33 (0.02 to 2.88)	0.62 (0.04 to 2.03)
		Poorer	2.01 (1.39 to 2.58)	2.58 (1.65 to 3.49)	0.88 (0.70 to 0.99)	0.08 (0.08 to 0.08)	0.21 (0.00 to 1.27)
		Middle	1.31 (0.87 to 1.66)	1.67 (1.02 to 2.27)	0.56 (0.44 to 0.64)	–	0.09 (0.05 to 0.66)
		Richer	0.72 (0.46 to 0.90)	0.92 (0.54 to 1.24)	0.30 (0.23 to 0.36)	–	–
		Richest	0.29 (0.17 to 0.41)	0.37 (0.19 to 0.54)	0.14 (0.11 to 0.18)	–	–
	Malawi	Poorest	0.68 (0.59 to 0.79)	0.85 (0.73 to 1.02)	0.34 (0.31 to 0.37)	–	–
		Poorer	0.64 (0.56 to 0.71)	0.82 (0.71 to 0.94)	0.28 (0.26 to 0.30)	–	–
		Middle	0.54 (0.47 to 0.62)	0.68 (0.58 to 0.80)	0.25 (0.23 to 0.27)	–	–
		Richer	0.39 (0.34 to 0.46)	0.49 (0.41 to 0.59)	0.19 (0.18 to 0.21)	–	–
		Richest	0.22 (0.17 to 0.27)	0.27 (0.20 to 0.34)	0.13 (0.11 to 0.15)	–	–
	Mali	Poorest	2.11 (1.82 to 2.37)	2.49 (2.09 to 2.84)	1.38 (1.27 to 1.48)	0.12 (0.01 to 0.52)	1.09 (0.71 to 1.47)
		Poorer	2.03 (1.80 to 2.24)	2.47 (2.13 to 2.77)	1.17 (1.10 to 1.23)	–	0.36 (0.15 to 0.62)
		Middle	1.69 (1.49 to 1.92)	2.04 (1.77 to 2.38)	1.01 (0.96 to 1.06)	–	0.31 (0.08 to 0.57)
		Richer	1.26 (1.09 to 1.46)	1.50 (1.27 to 1.80)	0.76 (0.71 to 0.81)	–	0.12 (0.01 to 0.30)
		Richest	0.70 (0.57 to 0.86)	0.82 (0.65 to 1.03)	0.48 (0.42 to 0.54)	–	0.07 (0.00 to 0.22)
	Morocco	Poorest	0.35 (-0.37 to 0.63)	0.46 (-0.31 to 0.93)	0.11 (-0.48 to 0.19)	1.25 (0.48 to 7.14)	0.63 (0.07 to 8.36)
		Poorer	0.25 (-0.18 to 0.43)	0.33 (-0.16 to 0.63)	0.07 (-0.23 to 0.12)	3.80 (3.47 to 4.14)	1.11 (0.01 to 5.34)
		Middle	0.17 (-0.16 to 0.28)	0.22 (-0.14 to 0.41)	0.05 (-0.21 to 0.09)	2.85 (2.35 to 3.35)	0.77 (0.30 to 4.54)
		Richer	0.09 (-0.15 to 0.18)	0.12 (-0.13 to 0.26)	0.03 (-0.21 to 0.06)	1.43 (0.55 to 2.31)	0.19 (0.08 to 3.49)
		Richest	0.04 (-0.12 to 0.10)	0.05 (-0.09 to 0.14)	0.01 (-0.17 to 0.03)	1.83 (1.83 to 1.83)	0.59 (0.04 to 3.00)
	Mozambique	Poorest	1.49 (0.98 to 1.84)	1.76 (1.10 to 2.25)	0.92 (0.73 to 1.02)	0.39 (0.17 to 0.85)	0.42 (0.05 to 1.18)
		Poorer	1.16 (0.82 to 1.42)	1.44 (0.95 to 1.82)	0.61 (0.56 to 0.66)	–	0.11 (0.02 to 0.65)
		Middle	0.78 (0.54 to 0.96)	0.96 (0.62 to 1.23)	0.42 (0.37 to 0.47)	–	0.13 (0.06 to 0.19)
		Richer	0.44 (0.30 to 0.57)	0.53 (0.35 to 0.72)	0.25 (0.21 to 0.28)	–	–
		Richest	0.18 (0.10 to 0.26)	0.21 (0.10 to 0.33)	0.12 (0.08 to 0.15)	–	–
	Namibia	Poorest	0.13 (-0.20 to 0.41)	0.13 (-0.19 to 0.46)	0.12 (-0.22 to 0.32)	0.33 (0.08 to 0.45)	0.48 (0.04 to 1.51)
		Poorer	0.23 (-0.04 to 0.50)	0.24 (-0.04 to 0.58)	0.19 (-0.04 to 0.33)	–	0.20 (0.01 to 0.84)
		Middle	0.17 (-0.13 to 0.40)	0.18 (-0.12 to 0.46)	0.15 (-0.14 to 0.29)	0.17 (0.14 to 0.21)	0.24 (0.01 to 0.88)
		Richer	0.07 (-0.20 to 0.29)	0.08 (-0.18 to 0.32)	0.07 (-0.22 to 0.22)	0.05 (0.05 to 0.05)	0.25 (0.01 to 0.78)
		Richest	-0.08 (-0.30 to 0.11)	-0.07 (-0.26 to 0.12)	-0.08 (-0.38 to 0.10)	–	0.27 (0.02 to 0.80)
	Niger	Poorest	4.49 (3.96 to 5.14)	6.36 (5.45 to 7.41)	0.86 (0.66 to 1.06)	–	–
		Poorer	4.03 (3.61 to 4.71)	5.78 (5.09 to 6.87)	0.63 (0.50 to 0.77)	–	–
		Middle	3.57 (3.10 to 4.13)	5.10 (4.37 to 6.04)	0.56 (0.43 to 0.70)	–	–
		Richer	2.82 (2.45 to 3.29)	4.03 (3.43 to 4.80)	0.44 (0.34 to 0.54)	–	–
		Richest	1.89 (1.57 to 2.30)	2.68 (2.19 to 3.32)	0.32 (0.25 to 0.40)	–	–
	Nigeria	Poorest	4.56 (4.14 to 4.98)	5.27 (4.73 to 5.86)	3.13 (2.93 to 3.27)	1.59 (1.10 to 2.07)	2.83 (2.33 to 3.31)
		Poorer	4.02 (3.64 to 4.37)	4.89 (4.36 to 5.40)	2.29 (2.19 to 2.37)	0.06 (0.00 to 0.27)	1.07 (0.76 to 1.40)
		Middle	3.13 (2.78 to 3.46)	3.85 (3.37 to 4.34)	1.69 (1.62 to 1.75)	–	0.56 (0.33 to 0.85)
		Richer	2.09 (1.85 to 2.35)	2.60 (2.26 to 2.98)	1.09 (1.04 to 1.13)	–	0.08 (0.00 to 0.26)
		Richest	1.09 (0.92 to 1.26)	1.34 (1.10 to 1.56)	0.60 (0.55 to 0.65)	–	0.05 (0.05 to 0.05)
	Rwanda	Poorest	0.52 (0.42 to 0.62)	0.69 (0.54 to 0.84)	0.20 (0.17 to 0.23)	–	–

Indicators	Country	Wealth Quintile	Average Annual Rate of Change (95% CrI)			Added AARC Needed to Achieve Targets (95% CrI)	
			2000–2030	2000–2020	2020–2030	UHC2030	IA2030
		Poorer	0.54 (0.46 to 0.62)	0.72 (0.60 to 0.85)	0.18 (0.15 to 0.21)	–	–
		Middle	0.52 (0.45 to 0.61)	0.69 (0.57 to 0.83)	0.19 (0.16 to 0.21)	–	–
		Richer	0.44 (0.36 to 0.53)	0.58 (0.45 to 0.71)	0.17 (0.14 to 0.19)	–	–
		Richest	0.30 (0.22 to 0.38)	0.38 (0.26 to 0.49)	0.14 (0.11 to 0.16)	–	–
	Sao Tome and Principe	Poorest	0.82 (-0.36 to 1.96)	1.10 (-0.32 to 2.89)	0.24 (-0.42 to 0.42)	0.68 (0.02 to 6.64)	0.68 (0.04 to 7.84)
		Poorer	0.76 (-0.14 to 1.83)	1.03 (-0.13 to 2.70)	0.21 (-0.15 to 0.34)	4.09 (4.09 to 4.09)	0.49 (0.01 to 5.29)
		Middle	0.67 (-0.16 to 1.72)	0.90 (-0.15 to 2.55)	0.20 (-0.18 to 0.33)	4.48 (4.48 to 4.48)	0.80 (0.23 to 5.68)
		Richer	0.51 (-0.18 to 1.46)	0.67 (-0.17 to 2.15)	0.16 (-0.22 to 0.29)	1.96 (0.10 to 3.83)	0.36 (0.02 to 5.01)
		Richest	0.30 (-0.28 to 1.04)	0.38 (-0.22 to 1.51)	0.10 (-0.39 to 0.24)	2.44 (0.84 to 4.03)	0.40 (0.13 to 5.22)
	Senegal	Poorest	1.38 (1.15 to 1.64)	1.79 (1.45 to 2.17)	0.55 (0.51 to 0.59)	–	–
		Poorer	1.16 (0.99 to 1.38)	1.54 (1.29 to 1.89)	0.39 (0.37 to 0.42)	–	–
		Middle	0.90 (0.76 to 1.08)	1.19 (0.98 to 1.47)	0.32 (0.30 to 0.34)	–	–
		Richer	0.61 (0.50 to 0.76)	0.80 (0.64 to 1.03)	0.23 (0.20 to 0.25)	–	–
		Richest	0.33 (0.26 to 0.43)	0.42 (0.33 to 0.57)	0.14 (0.12 to 0.17)	–	–
	Sierra Leone	Poorest	2.21 (1.76 to 2.63)	2.95 (2.29 to 3.61)	0.74 (0.66 to 0.84)	–	–
		Poorer	2.22 (1.80 to 2.68)	3.02 (2.36 to 3.71)	0.66 (0.60 to 0.73)	–	–
		Middle	2.18 (1.74 to 2.60)	2.93 (2.27 to 3.58)	0.69 (0.63 to 0.74)	–	–
		Richer	1.89 (1.43 to 2.26)	2.52 (1.85 to 3.10)	0.63 (0.57 to 0.69)	–	–
		Richest	1.38 (1.01 to 1.71)	1.81 (1.27 to 2.31)	0.53 (0.46 to 0.60)	–	–
	South Africa	Poorest	1.38 (-1.24 to 4.80)	1.64 (-1.00 to 6.62)	0.90 (-1.73 to 1.50)	0.70 (0.06 to 5.17)	0.99 (0.04 to 4.60)
		Poorer	1.58 (-1.04 to 5.00)	1.90 (-0.87 to 6.90)	0.93 (-1.39 to 1.35)	0.57 (0.07 to 3.65)	0.79 (0.05 to 3.88)
		Middle	1.53 (-1.20 to 4.94)	1.82 (-1.01 to 6.87)	0.96 (-1.59 to 1.46)	0.75 (0.03 to 4.19)	0.87 (0.03 to 5.34)
		Richer	1.37 (-1.43 to 4.52)	1.61 (-1.16 to 6.24)	0.88 (-1.98 to 1.45)	0.80 (0.03 to 5.15)	0.89 (0.05 to 5.22)
		Richest	0.87 (-1.54 to 3.62)	0.99 (-1.19 to 4.91)	0.65 (-2.21 to 1.37)	0.74 (0.01 to 5.75)	1.08 (0.06 to 5.86)
	Tanzania	Poorest	0.33 (0.05 to 0.59)	0.35 (0.05 to 0.67)	0.28 (0.05 to 0.43)	–	0.40 (0.04 to 0.99)
		Poorer	0.36 (0.13 to 0.55)	0.40 (0.14 to 0.66)	0.27 (0.12 to 0.35)	–	0.06 (0.00 to 0.39)
		Middle	0.23 (0.05 to 0.40)	0.26 (0.06 to 0.47)	0.19 (0.05 to 0.26)	–	0.12 (0.08 to 0.19)
		Richer	0.13 (-0.00 to 0.23)	0.14 (-0.00 to 0.27)	0.11 (-0.00 to 0.16)	–	–
		Richest	0.02 (-0.07 to 0.10)	0.02 (-0.07 to 0.10)	0.02 (-0.08 to 0.08)	–	–
	Togo	Poorest	1.34 (-0.14 to 3.30)	1.74 (-0.13 to 4.77)	0.49 (-0.15 to 0.63)	1.55 (0.44 to 2.91)	0.24 (0.00 to 4.10)
		Poorer	1.30 (-0.04 to 3.27)	1.74 (-0.03 to 4.75)	0.42 (-0.04 to 0.52)	1.39 (0.97 to 1.81)	0.51 (0.13 to 3.00)
		Middle	1.20 (-0.08 to 3.23)	1.57 (-0.08 to 4.69)	0.42 (-0.09 to 0.50)	0.93 (0.15 to 1.66)	0.58 (0.01 to 2.84)
		Richer	0.98 (-0.18 to 2.61)	1.27 (-0.17 to 3.81)	0.35 (-0.21 to 0.45)	0.76 (0.44 to 1.08)	0.86 (0.01 to 2.26)
		Richest	0.63 (-0.31 to 1.94)	0.81 (-0.28 to 2.78)	0.27 (-0.38 to 0.38)	0.66 (0.30 to 1.02)	0.82 (0.03 to 2.20)
	Uganda	Poorest	1.83 (1.65 to 2.05)	2.39 (2.12 to 2.73)	0.72 (0.66 to 0.78)	–	–
		Poorer	1.91 (1.74 to 2.10)	2.54 (2.27 to 2.83)	0.66 (0.60 to 0.71)	–	–
		Middle	1.89 (1.70 to 2.08)	2.49 (2.20 to 2.78)	0.70 (0.65 to 0.75)	–	–
		Richer	1.66 (1.49 to 1.85)	2.17 (1.90 to 2.46)	0.66 (0.60 to 0.70)	–	–
		Richest	1.21 (1.06 to 1.41)	1.54 (1.34 to 1.84)	0.55 (0.50 to 0.60)	–	–
	Zambia	Poorest	0.81 (0.61 to 0.99)	0.95 (0.69 to 1.21)	0.52 (0.45 to 0.58)	–	0.06 (0.01 to 0.22)
		Poorer	0.74 (0.58 to 0.89)	0.91 (0.68 to 1.12)	0.42 (0.38 to 0.45)	–	–
		Middle	0.58 (0.46 to 0.72)	0.70 (0.53 to 0.89)	0.35 (0.31 to 0.38)	–	–
		Richer	0.39 (0.30 to 0.51)	0.47 (0.34 to 0.62)	0.25 (0.21 to 0.28)	–	–
		Richest	0.19 (0.11 to 0.26)	0.21 (0.12 to 0.31)	0.14 (0.09 to 0.17)	–	–
	Zimbabwe	Poorest	2.22 (1.84 to 2.70)	2.85 (2.30 to 3.61)	0.97 (0.86 to 1.07)	–	0.04 (0.02 to 0.11)
		Poorer	2.13 (1.78 to 2.63)	2.80 (2.25 to 3.59)	0.82 (0.73 to 0.89)	–	–
		Middle	1.93 (1.55 to 2.38)	2.51 (1.94 to 3.20)	0.78 (0.70 to 0.85)	–	–
		Richer	1.55 (1.25 to 1.96)	2.01 (1.56 to 2.62)	0.65 (0.58 to 0.71)	–	–
		Richest	1.01 (0.76 to 1.35)	1.28 (0.92 to 1.78)	0.48 (0.42 to 0.56)	–	–

Notes: AARC = Average Annual Rate of Change. The additional AARC required to reach global targets was calculated as the difference between the projected AARC for 2020–2030 and the AARC needed to achieve the target coverage by 2030.

Table K. AARC and Additional values required to reach 2030 targets, by educational level

Indicators	Country	Educational Level	Average Annual Rate of Change (95% CrI)			Added AARC Needed to Achieve Targets (95% CrI)	
			2000–2030	2000–2020	2020–2030	UHC2030	IA2030
Full immunization	Angola	Primary or lower	0.29 (-2.88 to 5.61)	0.30 (-2.20 to 7.58)	0.28 (-4.32 to 1.71)	2.21 (0.10 to 8.22)	3.03 (0.42 to 9.44)
		Secondary or higher	-0.10 (-1.64 to 2.34)	-0.10 (-1.00 to 3.23)	-0.11 (-2.90 to 0.58)	0.89 (0.03 to 4.99)	0.90 (0.07 to 4.89)
	Benin	Primary or lower	-0.17 (-0.34 to 0.00)	-0.16 (-0.31 to 0.00)	-0.18 (-0.41 to 0.00)	0.17 (0.17 to 0.17)	0.67 (0.32 to 1.10)
		Secondary or higher	-0.08 (-0.15 to -0.03)	-0.07 (-0.12 to -0.03)	-0.11 (-0.21 to -0.04)	–	–
	Burkina Faso	Primary or lower	1.35 (1.18 to 1.56)	2.02 (1.76 to 2.34)	0.03 (0.02 to 0.05)	–	–
		Secondary or higher	0.31 (0.18 to 0.62)	0.46 (0.26 to 0.92)	0.01 (0.00 to 0.02)	–	–
	Burundi	Primary or lower	-0.10 (-0.45 to 0.04)	-0.08 (-0.21 to 0.05)	-0.15 (-0.93 to 0.03)	0.15 (0.15 to 0.15)	0.35 (0.04 to 1.31)
		Secondary or higher	-0.06 (-0.23 to 0.01)	-0.04 (-0.10 to 0.01)	-0.10 (-0.54 to 0.01)	–	0.30 (0.30 to 0.30)
	Cameroon	Primary or lower	-0.13 (-0.39 to 0.14)	-0.12 (-0.36 to 0.14)	-0.13 (-0.45 to 0.13)	0.25 (0.02 to 0.86)	1.27 (0.72 to 1.93)
		Secondary or higher	-0.10 (-0.20 to -0.02)	-0.09 (-0.17 to -0.02)	-0.13 (-0.27 to -0.02)	–	–
	Chad	Primary or lower	3.11 (2.52 to 3.64)	3.72 (2.92 to 4.49)	1.89 (1.71 to 2.03)	0.16 (0.02 to 0.63)	1.15 (0.67 to 1.77)
		Secondary or higher	0.98 (0.71 to 1.47)	1.20 (0.84 to 1.82)	0.58 (0.44 to 0.76)	–	0.11 (0.00 to 0.35)
	Comoros	Primary or lower	1.10 (-0.89 to 3.68)	1.39 (-0.73 to 5.44)	0.46 (-1.20 to 0.64)	1.20 (0.01 to 3.29)	0.81 (0.05 to 3.80)
		Secondary or higher	0.52 (-0.83 to 2.54)	0.64 (-0.61 to 3.72)	0.25 (-1.22 to 0.43)	0.70 (0.08 to 2.45)	0.79 (0.05 to 3.24)
	Congo Brazzaville	Primary or lower	0.77 (0.31 to 1.17)	1.02 (0.36 to 1.64)	0.28 (0.21 to 0.34)	–	–
		Secondary or higher	0.20 (0.05 to 0.36)	0.26 (0.05 to 0.50)	0.09 (0.04 to 0.12)	–	–
	Congo Democratic Republic	Primary or lower	2.56 (2.11 to 3.09)	3.46 (2.76 to 4.29)	0.79 (0.70 to 0.88)	–	–
		Secondary or higher	0.93 (0.70 to 1.22)	1.26 (0.91 to 1.68)	0.29 (0.25 to 0.34)	–	–
	Cote d'Ivoire	Primary or lower	1.04 (-1.02 to 2.90)	1.30 (-0.85 to 4.16)	0.46 (-1.38 to 0.58)	0.73 (0.10 to 5.07)	0.85 (0.03 to 6.14)
		Secondary or higher	0.27 (-0.46 to 1.05)	0.33 (-0.31 to 1.48)	0.11 (-0.75 to 0.28)	0.61 (0.01 to 1.49)	0.54 (0.19 to 2.66)
	Egypt	Primary or lower	0.00 (-0.07 to 0.04)	0.00 (-0.06 to 0.04)	0.00 (-0.09 to 0.03)	–	–
		Secondary or higher	-0.02 (-0.08 to 0.01)	-0.02 (-0.06 to 0.02)	-0.02 (-0.12 to 0.01)	–	–
	Eswatini	Primary or lower	0.20 (-0.60 to 0.55)	0.26 (-0.40 to 0.81)	0.05 (-1.00 to 0.12)	1.58 (0.80 to 3.75)	1.15 (0.07 to 4.93)
		Secondary or higher	0.08 (-0.28 to 0.25)	0.10 (-0.19 to 0.36)	0.03 (-0.47 to 0.06)	1.98 (1.64 to 2.32)	0.79 (0.62 to 3.50)
	Ethiopia	Primary or lower	1.87 (1.67 to 2.09)	2.21 (1.93 to 2.50)	1.22 (1.15 to 1.27)	–	0.82 (0.57 to 1.13)
		Secondary or higher	0.37 (0.26 to 0.52)	0.44 (0.30 to 0.63)	0.25 (0.20 to 0.31)	–	–
	Gabon	Primary or lower	0.26 (-0.02 to 0.48)	0.28 (-0.02 to 0.57)	0.21 (-0.02 to 0.31)	–	0.14 (0.02 to 0.86)
		Secondary or higher	0.04 (-0.13 to 0.19)	0.04 (-0.12 to 0.22)	0.04 (-0.16 to 0.12)	–	0.12 (0.05 to 0.44)
	Gambia	Primary or lower	0.08 (-0.02 to 0.33)	0.10 (-0.01 to 0.46)	0.04 (-0.02 to 0.08)	–	–
		Secondary or higher	0.03 (-0.02 to 0.14)	0.03 (-0.01 to 0.19)	0.01 (-0.02 to 0.04)	–	–
	Ghana	Primary or lower	0.48 (0.30 to 0.66)	0.64 (0.38 to 0.94)	0.15 (0.11 to 0.20)	–	–
		Secondary or higher	0.16 (0.09 to 0.28)	0.21 (0.10 to 0.38)	0.06 (0.04 to 0.09)	–	–
	Guinea	Primary or lower	-0.81 (-1.27 to -0.47)	-0.71 (-1.05 to -0.43)	-1.00 (-1.72 to -0.53)	2.08 (1.33 to 3.17)	3.28 (2.52 to 4.37)
		Secondary or higher	-0.44 (-0.71 to -0.25)	-0.34 (-0.51 to -0.21)	-0.63 (-1.11 to -0.34)	0.20 (0.01 to 0.58)	0.71 (0.09 to 1.52)
	Kenya	Primary or lower	0.64 (0.52 to 0.83)	0.89 (0.71 to 1.18)	0.14 (0.11 to 0.16)	–	–
		Secondary or higher	0.21 (0.14 to 0.30)	0.28 (0.19 to 0.43)	0.05 (0.04 to 0.08)	–	–
	Lesotho	Primary or lower	0.17 (-0.04 to 0.37)	0.20 (-0.04 to 0.49)	0.10 (-0.04 to 0.14)	–	–
		Secondary or higher	0.04 (-0.07 to 0.12)	0.05 (-0.06 to 0.15)	0.03 (-0.08 to 0.05)	–	–
	Liberia	Primary or lower	1.46 (1.14 to 1.82)	1.97 (1.47 to 2.53)	0.46 (0.40 to 0.51)	–	–
		Secondary or higher	0.48 (0.32 to 0.72)	0.64 (0.41 to 0.99)	0.17 (0.13 to 0.22)	–	–
	Madagascar	Primary or lower	1.88 (1.49 to 2.30)	2.65 (2.00 to 3.33)	0.36 (0.24 to 0.52)	–	–
		Secondary or higher	0.32 (0.22 to 0.44)	0.45 (0.30 to 0.62)	0.06 (0.04 to 0.10)	–	–
	Malawi	Primary or lower	0.30 (0.26 to 0.34)	0.40 (0.33 to 0.46)	0.11 (0.09 to 0.12)	–	–
		Secondary or higher	0.07 (0.04 to 0.10)	0.09 (0.05 to 0.14)	0.03 (0.02 to 0.05)	–	–
	Mali	Primary or lower	0.90 (0.75 to 1.03)	1.05 (0.86 to 1.24)	0.58 (0.54 to 0.62)	–	0.07 (0.01 to 0.31)
		Secondary or higher	0.15 (0.09 to 0.23)	0.17 (0.10 to 0.26)	0.11 (0.07 to 0.15)	–	–
	Morocco	Primary or lower	0.07 (-0.16 to 0.15)	0.09 (-0.13 to 0.22)	0.02 (-0.22 to 0.04)	–	0.62 (0.29 to 0.96)
		Secondary or higher	0.01 (-0.08 to 0.04)	0.02 (-0.05 to 0.06)	0.00 (-0.13 to 0.02)	–	–
	Mozambique	Primary or lower	0.35 (0.18 to 0.54)	0.41 (0.20 to 0.68)	0.23 (0.15 to 0.26)	–	–
		Secondary or higher	0.10 (0.00 to 0.21)	0.11 (0.00 to 0.26)	0.07 (0.00 to 0.13)	–	–
	Namibia	Primary or lower	0.30 (0.09 to 0.44)	0.36 (0.09 to 0.55)	0.18 (0.08 to 0.22)	–	0.08 (0.02 to 0.13)
		Secondary or higher	0.11 (-0.01 to 0.21)	0.13 (-0.01 to 0.25)	0.08 (-0.01 to 0.12)	–	–
	Niger	Primary or lower	3.34 (2.94 to 3.84)	4.84 (4.19 to 5.65)	0.40 (0.30 to 0.49)	–	–
		Secondary or higher	0.74 (0.42 to 1.10)	1.07 (0.61 to 1.61)	0.08 (0.05 to 0.12)	–	–
	Nigeria	Primary or lower	3.31 (2.90 to 3.77)	3.69 (3.20 to 4.29)	2.54 (2.31 to 2.75)	2.62 (2.09 to 3.22)	3.87 (3.33 to 4.48)
		Secondary or higher	0.49 (0.35 to 0.62)	0.57 (0.39 to 0.75)	0.32 (0.26 to 0.38)	–	–
	Rwanda	Primary or lower	0.14 (0.09 to 0.18)	0.17 (0.10 to 0.24)	0.07 (0.06 to 0.08)	–	–
		Secondary or higher	0.06 (0.03 to 0.11)	0.07 (0.03 to 0.13)	0.04 (0.02 to 0.05)	–	–
	Sao Tome and Principe	Primary or lower	0.29 (-0.17 to 0.91)	0.38 (-0.15 to 1.33)	0.08 (-0.21 to 0.15)	1.19 (1.19 to 1.19)	0.68 (0.35 to 2.37)
		Secondary or higher	0.07 (-0.09 to 0.32)	0.08 (-0.06 to 0.46)	0.02 (-0.13 to 0.06)	–	0.67 (0.67 to 0.67)
	Senegal	Primary or lower	0.26 (0.14 to 0.37)	0.31 (0.15 to 0.46)	0.16 (0.11 to 0.18)	–	–
		Secondary or higher	0.03 (0.01 to 0.08)	0.04 (0.01 to 0.09)	0.02 (0.01 to 0.04)	–	–
	Sierra Leone	Primary or lower	1.82 (1.32 to 2.26)	2.64 (1.86 to 3.33)	0.20 (0.17 to 0.25)	–	–
		Secondary or higher	0.83 (0.48 to 1.30)	1.18 (0.67 to 1.90)	0.11 (0.08 to 0.16)	–	–
	South Africa	Primary or lower	0.80 (-0.80 to 2.78)	1.01 (-0.60 to 3.93)	0.37 (-1.19 to 0.78)	0.93 (0.04 to 4.83)	0.61 (0.04 to 5.90)
		Secondary or higher	0.43 (-0.66 to 1.83)	0.52 (-0.44 to 2.60)	0.23 (-1.09 to 0.40)	0.44 (0.02 to 1.87)	0.40 (0.05 to 3.05)
	Tanzania	Primary or lower	0.36 (0.26 to 0.48)	0.48 (0.32 to 0.65)	0.14 (0.12 to 0.16)	–	–
		Secondary or higher	0.15 (0.07 to 0.23)	0.18 (0.09 to 0.31)	0.07 (0.04 to 0.10)	–	–
	Togo	Primary or lower	0.57 (-0.20 to 2.27)	0.76 (-0.17 to 3.36)	0.17 (-0.26 to 0.23)	–	0.17 (0.10 to 0.97)
		Secondary or higher	0.11 (-0.07 to 0.64)	0.14 (-0.06 to 0.94)	0.04 (-0.08 to 0.08)	–	–
	Uganda	Primary or lower	0.86 (0.76 to 0.96)	1.20 (1.05 to 1.37)	0.17 (0.15 to 0.20)	–	–
		Secondary or higher	0.40 (0.32 to 0.51)	0.55 (0.44 to 0.71)	0.10 (0.07 to 0.13)	–	–
	Zambia	Primary or lower	0.22 (0.14 to 0.30)	0.25 (0.15 to 0.36)	0.14 (0.11 to 0.16)	–	–
		Secondary or higher	0.06 (0.02 to 0.11)	0.07 (0.02 to 0.13)	0.05 (0.01 to 0.07)	–	–
	Zimbabwe	Primary or lower	1.72 (1.36 to 2.07)	2.39 (1.81 to 2.91)	0.43 (0.34 to 0.52)	–	–
		Secondary or higher	1.16 (0.88 to 1.40)	1.57 (1.14 to 1.95)	0.34 (0.29 to 0.40)	–	–
BCG immunization	Angola	Primary or lower	-0.49 (-7.16 to 5.03)	-0.49 (-6.13 to 5.59)	-0.50 (-9.18 to 3.89)	11.32 (4.17 to 24.37)	12.62 (5.21 to 25.67)
		Secondary or higher	-1.26 (-5.86 to 1.94)	-1.13 (-3.88 to 2.23)	-1.51 (-9.66 to 1.37)	5.07 (0.65 to 16.47)	6.26 (1.75 to 17.72)
	Benin	Primary or lower	0.40 (0.14 to 0.61)	0.42 (0.14 to 0.66)	0.36 (0.13 to 0.52)	0.70 (0.30 to 1.27)	1.89 (1.49 to 2.47)
		Secondary or higher	-0.13 (-0.27 to -0.02)	-0.12 (-0.25 to -0.02)	-0.15 (-0.32 to -0.02)	–	0.41 (0.10 to 0.87)

Indicators	Country	Educational Level	Average Annual Rate of Change (95% CrI)			Added AARC Needed to Achieve Targets (95% CrI)	
			2000–2030	2000–2020	2020–2030	UHC2030	IA2030
	Burkina Faso	Primary or lower	3.34 (3.05 to 3.63)	5.01 (4.56 to 5.46)	0.09 (0.06 to 0.11)	–	–
		Secondary or higher	1.50 (1.06 to 2.10)	2.24 (1.57 to 3.14)	0.05 (0.03 to 0.08)	–	–
	Burundi	Primary or lower	0.31 (0.12 to 0.55)	0.40 (0.13 to 0.76)	0.12 (0.08 to 0.15)	–	–
		Secondary or higher	0.18 (0.01 to 0.45)	0.22 (0.01 to 0.59)	0.11 (0.01 to 0.18)	–	–
	Cameroon	Primary or lower	0.60 (0.19 to 0.93)	0.64 (0.19 to 1.00)	0.54 (0.18 to 0.79)	1.68 (1.11 to 2.47)	2.89 (2.31 to 3.69)
		Secondary or higher	-0.16 (-0.36 to 0.01)	-0.16 (-0.33 to 0.01)	-0.17 (-0.42 to 0.01)	0.17 (0.01 to 0.55)	1.19 (0.76 to 1.66)
	Chad	Primary or lower	4.28 (3.08 to 5.35)	4.71 (3.29 to 6.05)	3.41 (2.65 to 3.93)	4.17 (2.75 to 5.98)	5.45 (4.01 to 7.27)
		Secondary or higher	1.66 (0.77 to 2.39)	1.83 (0.82 to 2.71)	1.32 (0.68 to 1.75)	1.46 (0.55 to 2.93)	2.68 (1.76 to 4.16)
	Comoros	Primary or lower	2.21 (-1.49 to 5.69)	2.81 (-1.31 to 8.28)	0.98 (-1.85 to 1.27)	1.25 (0.21 to 6.30)	0.79 (0.05 to 6.93)
		Secondary or higher	1.04 (-1.56 to 3.85)	1.25 (-1.24 to 5.61)	0.57 (-2.43 to 0.81)	1.39 (0.04 to 5.83)	0.80 (0.01 to 6.90)
	Congo Brazzaville	Primary or lower	-2.61 (-4.18 to -1.13)	-2.32 (-3.58 to -1.06)	-3.18 (-5.35 to -1.26)	10.30 (6.63 to 14.69)	11.57 (7.87 to 15.99)
		Secondary or higher	-3.01 (-4.50 to -1.64)	-2.39 (-3.40 to -1.41)	-4.26 (-6.64 to -2.09)	9.00 (5.40 to 13.24)	10.24 (6.62 to 14.50)
	Congo Democratic Republic	Primary or lower	4.75 (4.05 to 5.50)	6.07 (5.02 to 7.26)	2.14 (2.00 to 2.27)	–	0.31 (0.01 to 0.76)
		Secondary or higher	2.38 (1.90 to 2.89)	3.01 (2.31 to 3.81)	1.11 (1.02 to 1.21)	–	0.09 (0.01 to 0.45)
	Cote d'Ivoire	Primary or lower	1.75 (-2.09 to 5.44)	2.04 (-1.82 to 7.64)	1.13 (-2.62 to 1.47)	1.84 (0.10 to 8.32)	1.88 (0.03 to 9.21)
		Secondary or higher	0.41 (-1.74 to 2.12)	0.45 (-1.30 to 2.88)	0.32 (-2.63 to 0.65)	1.57 (0.04 to 5.04)	1.04 (0.05 to 5.99)
	Egypt	Primary or lower	0.28 (0.22 to 0.33)	0.37 (0.28 to 0.45)	0.12 (0.10 to 0.13)	–	–
		Secondary or higher	0.13 (0.09 to 0.16)	0.16 (0.10 to 0.21)	0.07 (0.06 to 0.08)	–	–
	Eswatini	Primary or lower	0.40 (-0.08 to 0.98)	0.54 (-0.08 to 1.45)	0.10 (-0.09 to 0.19)	1.70 (0.63 to 5.24)	1.81 (0.39 to 6.44)
		Secondary or higher	0.16 (-0.15 to 0.48)	0.21 (-0.13 to 0.68)	0.05 (-0.19 to 0.10)	1.84 (0.88 to 2.80)	1.02 (0.12 to 3.98)
	Ethiopia	Primary or lower	4.68 (4.41 to 5.00)	5.78 (5.37 to 6.26)	2.51 (2.44 to 2.60)	0.05 (0.01 to 0.06)	0.90 (0.62 to 1.20)
		Secondary or higher	1.59 (1.32 to 1.80)	1.98 (1.62 to 2.26)	0.81 (0.71 to 0.91)	–	0.00 (0.00 to 0.02)
	Gabon	Primary or lower	3.89 (3.46 to 4.35)	5.44 (4.74 to 6.11)	0.88 (0.72 to 1.07)	–	–
		Secondary or higher	2.22 (1.96 to 2.53)	3.04 (2.64 to 3.52)	0.59 (0.49 to 0.71)	–	–
	Gambia	Primary or lower	1.05 (0.53 to 1.64)	1.38 (0.65 to 2.27)	0.39 (0.31 to 0.47)	–	–
		Secondary or higher	0.69 (0.22 to 1.21)	0.86 (0.25 to 1.60)	0.35 (0.17 to 0.47)	–	–
	Ghana	Primary or lower	0.95 (0.66 to 1.27)	1.18 (0.77 to 1.66)	0.48 (0.42 to 0.54)	–	0.08 (0.08 to 0.08)
		Secondary or higher	0.31 (0.16 to 0.50)	0.36 (0.17 to 0.62)	0.20 (0.13 to 0.26)	–	–
	Guinea	Primary or lower	-2.27 (-2.91 to -1.64)	-2.09 (-2.64 to -1.54)	-2.62 (-3.46 to -1.84)	10.68 (9.19 to 12.29)	11.96 (10.46 to 13.58)
		Secondary or higher	-2.38 (-2.93 to -1.87)	-1.92 (-2.33 to -1.53)	-3.34 (-4.19 to -2.48)	7.04 (5.42 to 8.84)	8.27 (6.64 to 10.08)
	Kenya	Primary or lower	1.42 (1.18 to 1.64)	1.94 (1.56 to 2.27)	0.41 (0.37 to 0.46)	–	–
		Secondary or higher	0.61 (0.45 to 0.76)	0.80 (0.58 to 1.03)	0.24 (0.20 to 0.27)	–	–
	Lesotho	Primary or lower	0.41 (-0.12 to 0.81)	0.45 (-0.11 to 0.98)	0.33 (-0.12 to 0.49)	0.28 (0.05 to 0.99)	0.34 (0.02 to 1.56)
		Secondary or higher	-0.01 (-0.50 to 0.32)	-0.01 (-0.44 to 0.36)	-0.01 (-0.62 to 0.25)	0.50 (0.01 to 1.08)	0.45 (0.02 to 1.82)
	Liberia	Primary or lower	2.60 (2.06 to 3.21)	3.15 (2.42 to 4.00)	1.52 (1.37 to 1.66)	0.12 (0.00 to 0.19)	0.70 (0.29 to 1.21)
		Secondary or higher	1.12 (0.74 to 1.53)	1.30 (0.82 to 1.86)	0.74 (0.56 to 0.91)	–	0.40 (0.08 to 0.94)
	Madagascar	Primary or lower	2.54 (1.96 to 2.99)	3.46 (2.51 to 4.20)	0.74 (0.57 to 0.90)	–	0.16 (0.16 to 0.16)
		Secondary or higher	0.60 (0.43 to 0.81)	0.81 (0.54 to 1.11)	0.20 (0.14 to 0.26)	–	–
	Malawi	Primary or lower	0.60 (0.54 to 0.67)	0.76 (0.67 to 0.86)	0.27 (0.26 to 0.29)	–	–
		Secondary or higher	0.18 (0.14 to 0.25)	0.22 (0.16 to 0.30)	0.11 (0.09 to 0.15)	–	–
	Mali	Primary or lower	2.37 (2.17 to 2.57)	2.95 (2.64 to 3.26)	1.23 (1.18 to 1.27)	–	0.15 (0.02 to 0.35)
		Secondary or higher	0.77 (0.60 to 0.91)	0.93 (0.71 to 1.12)	0.45 (0.38 to 0.53)	–	–
	Morocco	Primary or lower	0.25 (-0.23 to 0.43)	0.33 (-0.20 to 0.64)	0.07 (-0.27 to 0.12)	0.84 (0.84 to 0.84)	0.73 (0.40 to 2.02)
		Secondary or higher	0.07 (-0.22 to 0.16)	0.09 (-0.16 to 0.23)	0.02 (-0.31 to 0.05)	0.03 (0.03 to 0.03)	0.71 (0.21 to 1.20)
	Mozambique	Primary or lower	0.52 (0.12 to 0.86)	0.56 (0.13 to 0.98)	0.44 (0.12 to 0.61)	0.19 (0.01 to 0.89)	1.04 (0.39 to 1.97)
		Secondary or higher	-0.04 (-0.31 to 0.16)	-0.04 (-0.28 to 0.17)	-0.04 (-0.37 to 0.14)	–	0.32 (0.01 to 0.97)
	Namibia	Primary or lower	0.51 (0.19 to 0.80)	0.56 (0.20 to 0.95)	0.39 (0.18 to 0.50)	0.15 (0.04 to 0.27)	0.37 (0.02 to 1.12)
		Secondary or higher	0.04 (-0.29 to 0.34)	0.04 (-0.27 to 0.38)	0.03 (-0.34 to 0.27)	0.14 (0.02 to 0.40)	0.51 (0.08 to 1.35)
	Niger	Primary or lower	6.21 (5.54 to 6.90)	8.99 (7.91 to 10.12)	0.85 (0.70 to 1.02)	–	–
		Secondary or higher	2.64 (1.96 to 3.36)	3.81 (2.80 to 4.88)	0.32 (0.21 to 0.46)	–	–
	Nigeria	Primary or lower	5.40 (4.73 to 5.90)	5.96 (5.16 to 6.61)	4.29 (3.89 to 4.54)	4.72 (4.03 to 5.50)	6.01 (5.32 to 6.79)
		Secondary or higher	1.34 (1.07 to 1.56)	1.56 (1.22 to 1.85)	0.90 (0.78 to 0.98)	–	0.61 (0.40 to 0.88)
	Rwanda	Primary or lower	0.71 (0.62 to 0.81)	0.97 (0.83 to 1.11)	0.21 (0.18 to 0.24)	–	–
		Secondary or higher	0.46 (0.37 to 0.58)	0.60 (0.47 to 0.76)	0.19 (0.15 to 0.24)	–	–
	Sao Tome and Principe	Primary or lower	0.97 (-0.11 to 2.02)	1.31 (-0.10 to 2.97)	0.25 (-0.11 to 0.41)	0.55 (0.17 to 2.06)	0.63 (0.01 to 3.25)
		Secondary or higher	0.51 (-0.31 to 1.18)	0.66 (-0.26 to 1.69)	0.15 (-0.40 to 0.35)	0.77 (0.02 to 2.90)	0.39 (0.02 to 4.09)
	Senegal	Primary or lower	1.17 (1.02 to 1.36)	1.53 (1.29 to 1.81)	0.47 (0.44 to 0.49)	–	–
		Secondary or higher	0.20 (0.14 to 0.27)	0.25 (0.17 to 0.35)	0.10 (0.08 to 0.13)	–	–
	Sierra Leone	Primary or lower	3.35 (2.89 to 3.90)	4.60 (3.91 to 5.43)	0.90 (0.82 to 0.97)	–	–
		Secondary or higher	2.09 (1.64 to 2.62)	2.80 (2.14 to 3.57)	0.68 (0.59 to 0.78)	–	–
	South Africa	Primary or lower	1.78 (-2.83 to 7.54)	1.99 (-2.45 to 10.20)	1.31 (-3.59 to 2.99)	2.55 (0.07 to 11.12)	2.96 (0.16 to 11.49)
		Secondary or higher	0.51 (-3.16 to 5.32)	0.53 (-2.13 to 6.95)	0.46 (-5.18 to 2.03)	2.52 (0.15 to 9.59)	3.01 (0.31 to 10.08)
	Tanzania	Primary or lower	0.17 (-0.00 to 0.36)	0.18 (-0.00 to 0.41)	0.15 (-0.00 to 0.26)	–	0.09 (0.02 to 0.47)
		Secondary or higher	-0.06 (-0.18 to 0.05)	-0.06 (-0.16 to 0.05)	-0.07 (-0.21 to 0.04)	–	0.13 (0.02 to 0.23)
	Togo	Primary or lower	1.74 (0.13 to 4.97)	2.28 (0.13 to 7.28)	0.63 (0.12 to 0.77)	1.25 (0.38 to 1.25)	0.37 (0.03 to 2.44)
		Secondary or higher	0.76 (-0.21 to 2.82)	0.96 (-0.19 to 4.07)	0.33 (-0.24 to 0.51)	1.07 (0.12 to 1.10)	0.28 (0.02 to 2.29)
	Uganda	Primary or lower	2.47 (2.26 to 2.68)	3.18 (2.88 to 3.52)	1.03 (0.98 to 1.09)	–	–
		Secondary or higher	1.36 (1.14 to 1.54)	1.69 (1.40 to 1.96)	0.70 (0.63 to 0.77)	–	0.04 (0.04 to 0.05)
	Zambia	Primary or lower	0.81 (0.68 to 0.97)	0.98 (0.80 to 1.20)	0.47 (0.43 to 0.51)	–	–
		Secondary or higher	0.22 (0.14 to 0.32)	0.25 (0.15 to 0.37)	0.16 (0.12 to 0.21)	–	–
	Zimbabwe	Primary or lower	2.81 (2.44 to 3.27)	3.85 (3.28 to 4.57)	0.72 (0.58 to 0.89)	–	–
		Secondary or higher	1.74 (1.39 to 2.05)	2.34 (1.78 to 2.81)	0.56 (0.50 to 0.64)	–	–
MCV1 immunization	Angola	Primary or lower	-1.86 (-9.04 to 5.39)	-1.81 (-7.92 to 5.82)	-1.96 (-11.22 to 4.53)	16.33 (5.60 to 31.62)	17.69 (6.90 to 33.04)
		Secondary or higher	-2.28 (-6.89 to 2.05)	-1.98 (-4.64 to 2.30)	-2.86 (-11.45 to 1.56)	8.13 (1.38 to 20.06)	9.38 (2.59 to 21.10)
	Benin	Primary or lower	0.85 (0.52 to 1.19)	0.90 (0.54 to 1.30)	0.75 (0.48 to 0.99)	1.94 (1.30 to 2.53)	3.16 (2.51 to 3.75)
		Secondary or higher	-0.10 (-0.32 to 0.12)	-0.10 (-0.30 to 0.12)	-0.10 (-0.36 to 0.11)	0.41 (0.05 to 1.00)	1.58 (1.09 to 2.17)
	Burkina Faso	Primary or lower	4.34 (4.01 to 4.73)	6.50 (5.96 to 7.11)	0.15 (0.12 to 0.19)	–	–
		Secondary or higher	2.19 (1.70 to 2.79)	3.26 (2.53 to 4.17)	0.09 (0.06 to 0.13)	–	–
	Burundi	Primary or lower	-0.15 (-0.54 to 0.18)	-0.14 (-0.43 to 0.19)	-0.16 (-0.77 to 0.15)	0.18 (0.00 to 0.50)	0.40 (0.04 to 1.19)
		Secondary or higher	-0.38 (-0.79 to -0.06)	-0.30 (-0.54 to -0.05)	-0.52 (-1.29 to -0.06)	0.36 (0.02 to 1.30)	0.62 (0.03 to 1.78)
	Cameroon	Primary or lower	0.47 (0.04 to 0.92)	0.48 (0.04 to 0.97)	0.45 (0.04 to 0.84)	4.51 (3.71 to 5.36)	5.75 (4.94 to 6.61)
		Secondary or higher	-0.45 (-0.74 to -0.16)	-0.42 (-0.66 to -0.16)	-0.50 (-0.88 to -0.17)	1.85 (1.19 to 2.54)	3.05 (2.39 to 3.74)

Indicators	Country	Educational Level	Average Annual Rate of Change (95% CrI)			Added AARC Needed to Achieve Targets (95% CrI)	
			2000–2030	2000–2020	2020–2030	UHC2030	IA2030
	Chad	Primary or lower	6.62 (5.23 to 7.74)	7.48 (5.72 to 8.98)	4.89 (4.26 to 5.36)	3.52 (2.25 to 5.31)	4.81 (3.52 to 6.61)
		Secondary or higher	3.26 (2.33 to 4.28)	3.81 (2.60 to 5.08)	2.22 (1.79 to 2.69)	1.05 (0.27 to 2.48)	2.24 (1.33 to 3.71)
	Comoros	Primary or lower	2.04 (-2.38 to 5.49)	2.44 (-2.02 to 7.70)	1.23 (-3.11 to 1.67)	1.11 (0.07 to 8.88)	1.41 (0.05 to 10.05)
		Secondary or higher	0.89 (-2.92 to 3.47)	1.00 (-2.13 to 4.82)	0.65 (-4.46 to 1.14)	1.26 (0.07 to 8.46)	1.42 (0.01 to 9.59)
	Congo Brazzaville	Primary or lower	-2.37 (-4.10 to -0.70)	-2.22 (-3.71 to -0.69)	-2.66 (-4.88 to -0.73)	12.47 (8.36 to 17.26)	13.77 (9.64 to 18.60)
		Secondary or higher	-3.07 (-4.56 to -1.67)	-2.61 (-3.74 to -1.51)	-3.97 (-6.20 to -1.99)	10.55 (7.08 to 14.73)	11.81 (8.32 to 16.02)
	Congo Democratic Republic	Primary or lower	5.52 (4.67 to 6.49)	6.93 (5.63 to 8.45)	2.75 (2.60 to 2.92)	0.18 (0.02 to 0.39)	0.70 (0.14 to 1.40)
		Secondary or higher	2.99 (2.34 to 3.67)	3.72 (2.78 to 4.77)	1.53 (1.42 to 1.69)	–	0.36 (0.02 to 0.93)
	Cote d'Ivoire	Primary or lower	1.78 (-3.11 to 5.90)	1.96 (-2.74 to 8.05)	1.39 (-3.85 to 2.11)	2.53 (0.13 to 12.68)	3.11 (0.07 to 13.09)
		Secondary or higher	0.31 (-2.56 to 2.31)	0.33 (-1.86 to 3.07)	0.27 (-3.94 to 0.88)	1.32 (0.07 to 8.82)	1.44 (0.05 to 8.19)
	Egypt	Primary or lower	-0.73 (-0.98 to -0.44)	-0.60 (-0.77 to -0.39)	-1.00 (-1.40 to -0.56)	0.93 (0.12 to 1.68)	2.11 (1.30 to 2.87)
		Secondary or higher	-1.05 (-1.32 to -0.81)	-0.75 (-0.90 to -0.61)	-1.65 (-2.14 to -1.19)	1.54 (0.82 to 2.29)	2.72 (2.00 to 3.48)
	Eswatini	Primary or lower	0.70 (-1.15 to 1.63)	0.90 (-0.95 to 2.39)	0.24 (-1.56 to 0.40)	1.80 (0.12 to 4.19)	0.96 (0.06 to 5.36)
		Secondary or higher	0.25 (-1.34 to 0.74)	0.30 (-0.92 to 1.06)	0.12 (-2.01 to 0.22)	2.25 (0.09 to 6.09)	0.75 (0.13 to 6.08)
	Ethiopia	Primary or lower	5.69 (5.30 to 6.14)	6.59 (6.08 to 7.24)	3.88 (3.71 to 4.03)	1.88 (1.28 to 2.41)	3.13 (2.53 to 3.67)
		Secondary or higher	2.07 (1.77 to 2.41)	2.47 (2.07 to 2.96)	1.25 (1.11 to 1.42)	–	0.61 (0.27 to 0.98)
	Gabon	Primary or lower	5.33 (4.30 to 6.18)	6.31 (4.87 to 7.58)	3.40 (3.11 to 3.73)	1.19 (0.14 to 2.91)	2.39 (1.15 to 4.04)
		Secondary or higher	3.86 (2.81 to 4.91)	4.33 (3.04 to 5.78)	2.91 (2.34 to 3.27)	2.59 (1.09 to 4.88)	3.84 (2.33 to 6.14)
	Gambia	Primary or lower	0.57 (0.11 to 1.19)	0.65 (0.12 to 1.49)	0.40 (0.11 to 0.58)	–	0.12 (0.00 to 0.53)
		Secondary or higher	0.19 (-0.27 to 0.76)	0.20 (-0.25 to 0.90)	0.17 (-0.32 to 0.49)	0.08 (0.08 to 0.08)	0.40 (0.06 to 1.04)
	Ghana	Primary or lower	1.09 (0.67 to 1.51)	1.28 (0.74 to 1.87)	0.68 (0.53 to 0.78)	0.37 (0.20 to 0.53)	0.23 (0.01 to 0.80)
		Secondary or higher	0.36 (0.04 to 0.65)	0.40 (0.04 to 0.77)	0.28 (0.04 to 0.40)	–	0.20 (0.01 to 0.82)
	Guinea	Primary or lower	-3.32 (-4.28 to -2.45)	-3.08 (-3.90 to -2.31)	-3.79 (-5.02 to -2.74)	15.79 (13.58 to 18.01)	17.12 (14.90 to 19.35)
		Secondary or higher	-3.61 (-4.50 to -2.92)	-2.97 (-3.59 to -2.43)	-4.86 (-6.21 to -3.74)	11.72 (9.86 to 14.04)	12.99 (11.11 to 15.32)
	Kenya	Primary or lower	2.13 (1.78 to 2.41)	2.73 (2.21 to 3.16)	0.93 (0.87 to 0.97)	–	–
		Secondary or higher	0.85 (0.60 to 1.04)	1.05 (0.71 to 1.30)	0.44 (0.38 to 0.50)	–	–
	Lesotho	Primary or lower	0.62 (-0.03 to 1.26)	0.66 (-0.03 to 1.43)	0.53 (-0.03 to 0.91)	0.61 (0.06 to 2.05)	1.67 (0.80 to 3.03)
		Secondary or higher	-0.13 (-0.76 to 0.44)	-0.12 (-0.68 to 0.47)	-0.13 (-0.93 to 0.37)	0.96 (0.10 to 2.46)	2.07 (0.92 to 3.53)
	Liberia	Primary or lower	2.12 (1.50 to 2.73)	2.38 (1.62 to 3.15)	1.62 (1.26 to 1.91)	1.49 (0.85 to 2.43)	2.71 (2.06 to 3.65)
		Secondary or higher	0.70 (0.18 to 1.14)	0.74 (0.18 to 1.27)	0.61 (0.17 to 0.90)	1.13 (0.46 to 2.10)	2.33 (1.66 to 3.31)
	Madagascar	Primary or lower	2.75 (2.16 to 3.44)	3.52 (2.61 to 4.62)	1.23 (0.98 to 1.37)	–	0.15 (0.00 to 1.05)
		Secondary or higher	0.82 (0.60 to 1.10)	1.02 (0.70 to 1.47)	0.40 (0.32 to 0.49)	–	0.13 (0.08 to 0.19)
	Malawi	Primary or lower	0.98 (0.87 to 1.09)	1.20 (1.05 to 1.37)	0.55 (0.52 to 0.57)	–	–
		Secondary or higher	0.29 (0.21 to 0.38)	0.34 (0.23 to 0.44)	0.21 (0.16 to 0.25)	–	–
	Mali	Primary or lower	2.58 (2.28 to 2.81)	2.99 (2.59 to 3.31)	1.76 (1.64 to 1.84)	0.52 (0.17 to 0.93)	1.73 (1.38 to 2.14)
		Secondary or higher	0.81 (0.60 to 1.03)	0.91 (0.65 to 1.17)	0.62 (0.50 to 0.76)	0.13 (0.13 to 0.13)	0.72 (0.43 to 1.09)
	Morocco	Primary or lower	0.48 (-1.41 to 0.77)	0.61 (-1.10 to 1.14)	0.15 (-2.03 to 0.26)	3.15 (0.01 to 11.49)	0.80 (0.04 to 11.83)
		Secondary or higher	0.11 (-1.09 to 0.28)	0.14 (-0.70 to 0.40)	0.04 (-1.81 to 0.11)	1.28 (0.09 to 10.15)	0.86 (0.03 to 11.37)
	Mozambique	Primary or lower	0.52 (0.06 to 0.91)	0.54 (0.06 to 1.00)	0.46 (0.06 to 0.72)	0.95 (0.17 to 1.98)	2.14 (1.34 to 3.16)
		Secondary or higher	-0.13 (-0.35 to 0.07)	-0.13 (-0.32 to 0.08)	-0.14 (-0.43 to 0.07)	0.36 (0.03 to 0.62)	0.66 (0.13 to 1.54)
	Namibia	Primary or lower	0.99 (0.59 to 1.31)	1.12 (0.63 to 1.53)	0.71 (0.50 to 0.83)	0.22 (0.01 to 0.94)	0.64 (0.12 to 1.62)
		Secondary or higher	0.29 (-0.14 to 0.62)	0.31 (-0.13 to 0.69)	0.26 (-0.14 to 0.48)	0.24 (0.01 to 1.13)	1.00 (0.37 to 2.05)
	Niger	Primary or lower	6.63 (5.79 to 7.32)	9.17 (7.79 to 10.30)	1.76 (1.50 to 2.00)	–	–
		Secondary or higher	3.08 (2.46 to 3.78)	4.29 (3.37 to 5.29)	0.72 (0.55 to 0.92)	–	–
	Nigeria	Primary or lower	5.39 (4.81 to 6.03)	5.78 (5.12 to 6.55)	4.60 (4.20 to 5.01)	7.38 (6.64 to 8.34)	8.70 (7.97 to 9.68)
		Secondary or higher	1.60 (1.25 to 1.87)	1.81 (1.37 to 2.14)	1.20 (1.01 to 1.33)	0.58 (0.31 to 1.04)	1.79 (1.52 to 2.25)
	Rwanda	Primary or lower	1.25 (1.11 to 1.39)	1.63 (1.42 to 1.83)	0.51 (0.47 to 0.55)	–	–
		Secondary or higher	0.65 (0.50 to 0.82)	0.81 (0.59 to 1.03)	0.35 (0.30 to 0.41)	–	–
	Sao Tome and Principe	Primary or lower	1.26 (-0.55 to 3.32)	1.61 (-0.52 to 4.84)	0.45 (-0.61 to 0.69)	1.09 (0.16 to 9.29)	0.93 (0.00 to 3.95)
		Secondary or higher	0.73 (-1.35 to 2.50)	0.88 (-1.07 to 3.59)	0.35 (-1.92 to 0.66)	1.52 (0.17 to 7.15)	1.05 (0.05 to 5.30)
	Senegal	Primary or lower	1.86 (1.62 to 2.10)	2.33 (1.97 to 2.67)	0.94 (0.90 to 0.98)	–	0.03 (0.00 to 0.23)
		Secondary or higher	0.45 (0.31 to 0.60)	0.54 (0.37 to 0.73)	0.27 (0.21 to 0.34)	–	–
	Sierra Leone	Primary or lower	2.07 (1.49 to 2.55)	2.40 (1.68 to 3.05)	1.40 (1.14 to 1.57)	0.24 (0.01 to 0.71)	1.31 (0.91 to 1.86)
		Secondary or higher	0.85 (0.40 to 1.29)	0.93 (0.42 to 1.48)	0.68 (0.36 to 0.92)	0.21 (0.02 to 0.77)	1.31 (0.89 to 1.92)
	South Africa	Primary or lower	1.55 (-3.86 to 7.72)	1.67 (-3.41 to 9.89)	1.35 (-5.15 to 3.99)	3.70 (0.18 to 14.70)	4.73 (0.53 to 15.99)
		Secondary or higher	0.19 (-4.03 to 5.36)	0.19 (-2.97 to 6.81)	0.18 (-6.12 to 2.35)	3.11 (0.25 to 12.57)	4.08 (0.43 to 13.33)
	Tanzania	Primary or lower	0.52 (0.23 to 0.77)	0.56 (0.23 to 0.88)	0.42 (0.21 to 0.55)	0.08 (0.03 to 0.29)	0.70 (0.32 to 1.24)
		Secondary or higher	-0.02 (-0.19 to 0.13)	-0.02 (-0.18 to 0.14)	-0.02 (-0.21 to 0.12)	–	0.34 (0.03 to 0.76)
	Togo	Primary or lower	2.04 (-1.11 to 5.32)	2.46 (-1.00 to 7.41)	1.20 (-1.31 to 1.45)	0.91 (0.04 to 5.93)	1.15 (0.11 to 6.17)
		Secondary or higher	0.68 (-1.05 to 2.77)	0.79 (-0.83 to 3.90)	0.48 (-1.50 to 0.79)	0.78 (0.15 to 5.07)	0.71 (0.06 to 5.19)
	Uganda	Primary or lower	2.70 (2.41 to 3.01)	3.23 (2.84 to 3.68)	1.64 (1.56 to 1.72)	0.03 (0.01 to 0.12)	0.94 (0.62 to 1.28)
		Secondary or higher	1.28 (1.03 to 1.53)	1.47 (1.16 to 1.80)	0.91 (0.77 to 1.01)	0.07 (0.01 to 0.20)	0.86 (0.51 to 1.30)
	Zambia	Primary or lower	0.91 (0.70 to 1.10)	1.03 (0.77 to 1.27)	0.67 (0.56 to 0.76)	0.02 (0.02 to 0.02)	0.73 (0.43 to 1.11)
		Secondary or higher	0.16 (0.02 to 0.29)	0.17 (0.02 to 0.31)	0.15 (0.02 to 0.24)	–	0.38 (0.14 to 0.73)
	Zimbabwe	Primary or lower	3.62 (3.14 to 4.22)	4.76 (3.99 to 5.70)	1.34 (1.20 to 1.50)	–	0.08 (0.05 to 0.30)
		Secondary or higher	2.10 (1.62 to 2.54)	2.68 (2.02 to 3.38)	0.91 (0.84 to 0.99)	–	0.06 (0.00 to 0.23)
Polio3 immunization	Angola	Primary or lower	-1.38 (-6.18 to 4.28)	-1.30 (-4.84 to 5.01)	-1.53 (-8.78 to 2.84)	8.09 (1.14 to 19.11)	9.35 (2.32 to 20.38)
		Secondary or higher	-0.97 (-3.57 to 1.30)	-0.79 (-2.03 to 1.58)	-1.33 (-6.90 to 0.78)	2.40 (0.15 to 9.33)	3.11 (0.39 to 10.54)
	Benin	Primary or lower	0.39 (0.12 to 0.62)	0.40 (0.12 to 0.65)	0.36 (0.12 to 0.55)	1.52 (1.12 to 2.08)	2.73 (2.33 to 3.29)
		Secondary or higher	-0.15 (-0.35 to 0.01)	-0.14 (-0.32 to 0.01)	-0.16 (-0.42 to 0.01)	0.13 (0.02 to 0.67)	0.98 (0.61 to 1.50)
	Burkina Faso	Primary or lower	3.29 (2.97 to 3.59)	4.89 (4.41 to 5.38)	0.13 (0.10 to 0.18)	–	–
		Secondary or higher	1.28 (0.89 to 1.77)	1.90 (1.32 to 2.64)	0.06 (0.03 to 0.08)	–	–
	Burundi	Primary or lower	-0.02 (-0.28 to 0.24)	-0.02 (-0.23 to 0.27)	-0.02 (-0.38 to 0.16)	–	0.19 (0.02 to 0.51)
		Secondary or higher	-0.08 (-0.27 to 0.06)	-0.07 (-0.20 to 0.06)	-0.09 (-0.40 to 0.05)	–	0.10 (0.01 to 0.23)
	Cameroon	Primary or lower	-0.28 (-0.71 to 0.15)	-0.27 (-0.68 to 0.15)	-0.29 (-0.78 to 0.15)	3.59 (2.61 to 4.44)	4.81 (3.83 to 5.66)
		Secondary or higher	-0.55 (-0.82 to -0.33)	-0.49 (-0.70 to -0.31)	-0.67 (-1.08 to -0.38)	1.02 (0.47 to 1.72)	2.21 (1.66 to 2.91)
	Chad	Primary or lower	6.57 (5.76 to 7.35)	8.76 (7.57 to 10.10)	2.24 (2.07 to 2.42)	–	0.11 (0.02 to 0.43)
		Secondary or higher	3.31 (2.66 to 4.20)	4.48 (3.57 to 5.75)	0.98 (0.79 to 1.22)	–	–
	Comoros	Primary or lower	1.38 (-1.83 to 4.81)	1.62 (-1.46 to 6.96)	0.78 (-2.55 to 1.09)	1.26 (0.06 to 7.91)	1.20 (0.10 to 6.85)
		Secondary or higher	0.61 (-2.07 to 3.27)	0.70 (-1.43 to 4.55)	0.43 (-3.31 to 0.78)	1.17 (0.04 to 5.87)	1.07 (0.02 to 6.59)
	Congo Brazzaville	Primary or lower	2.26 (1.43 to 3.01)	2.78 (1.64 to 3.92)	1.21 (1.01 to 1.34)	0.33 (0.02 to 1.18)	0.45 (0.02 to 1.63)
		Secondary or higher	0.85 (0.36 to 1.30)	1.01 (0.39 to 1.68)	0.50 (0.30 to 0.58)	–	0.16 (0.01 to 0.74)

Indicators	Country	Educational Level	Average Annual Rate of Change (95% CrI)			Added AARC Needed to Achieve Targets (95% CrI)	
			2000–2030	2000–2020	2020–2030	UHC2030	IA2030
	Congo Democratic Republic	Primary or lower	2.27 (1.48 to 2.82)	2.76 (1.68 to 3.57)	1.31 (1.08 to 1.41)	0.12 (0.04 to 0.58)	0.51 (0.10 to 1.25)
		Secondary or higher	0.87 (0.49 to 1.22)	1.03 (0.54 to 1.53)	0.56 (0.38 to 0.65)	–	0.14 (0.00 to 0.57)
	Cote d'Ivoire	Primary or lower	1.04 (-3.90 to 3.90)	1.13 (-3.11 to 5.24)	0.86 (-5.46 to 1.43)	2.22 (0.05 to 13.57)	2.30 (0.10 to 13.79)
		Secondary or higher	0.15 (-2.77 to 1.40)	0.16 (-1.71 to 1.90)	0.14 (-4.83 to 0.55)	1.10 (0.08 to 8.30)	1.23 (0.04 to 8.51)
	Egypt	Primary or lower	-2.20 (-2.84 to -1.53)	-1.16 (-1.44 to -0.89)	-4.20 (-5.58 to -2.81)	4.62 (2.66 to 6.51)	5.81 (3.84 to 7.70)
		Secondary or higher	-2.22 (-2.81 to -1.64)	-1.03 (-1.22 to -0.81)	-4.57 (-5.96 to -3.29)	4.56 (2.85 to 6.25)	5.74 (4.03 to 7.44)
	Eswatini	Primary or lower	0.46 (-0.95 to 1.17)	0.57 (-0.76 to 1.67)	0.19 (-1.42 to 0.30)	1.33 (0.02 to 5.69)	0.94 (0.01 to 5.61)
		Secondary or higher	0.16 (-0.76 to 0.54)	0.19 (-0.52 to 0.77)	0.08 (-1.24 to 0.16)	0.89 (0.33 to 4.34)	0.68 (0.02 to 4.41)
	Ethiopia	Primary or lower	3.54 (3.28 to 3.80)	4.18 (3.82 to 4.54)	2.26 (2.17 to 2.35)	0.46 (0.18 to 0.87)	1.67 (1.40 to 2.09)
		Secondary or higher	1.10 (0.90 to 1.33)	1.32 (1.05 to 1.60)	0.69 (0.60 to 0.79)	–	0.11 (0.01 to 0.40)
	Gabon	Primary or lower	2.26 (1.97 to 2.60)	2.84 (2.45 to 3.39)	1.06 (0.94 to 1.20)	–	0.08 (0.00 to 0.27)
		Secondary or higher	1.33 (1.10 to 1.61)	1.62 (1.32 to 2.05)	0.73 (0.64 to 0.81)	–	0.09 (0.01 to 0.33)
	Gambia	Primary or lower	-0.39 (-0.82 to -0.01)	-0.31 (-0.52 to -0.01)	-0.56 (-1.43 to -0.01)	0.27 (0.08 to 1.04)	0.67 (0.08 to 1.73)
		Secondary or higher	-0.49 (-0.89 to -0.15)	-0.34 (-0.50 to -0.14)	-0.80 (-1.75 to -0.19)	0.33 (0.02 to 1.02)	0.73 (0.08 to 1.88)
	Ghana	Primary or lower	0.66 (0.37 to 0.94)	0.80 (0.41 to 1.20)	0.38 (0.29 to 0.44)	–	0.11 (0.02 to 0.42)
		Secondary or higher	0.22 (0.07 to 0.41)	0.26 (0.07 to 0.52)	0.16 (0.06 to 0.22)	–	–
	Guinea	Primary or lower	-2.13 (-2.70 to -1.52)	-1.94 (-2.41 to -1.42)	-2.50 (-3.28 to -1.71)	9.51 (8.27 to 10.83)	10.77 (9.54 to 12.11)
		Secondary or higher	-2.11 (-2.67 to -1.62)	-1.73 (-2.09 to -1.36)	-2.89 (-3.79 to -2.13)	6.28 (4.83 to 7.73)	7.50 (6.05 to 8.96)
	Kenya	Primary or lower	1.18 (0.99 to 1.40)	1.50 (1.21 to 1.79)	0.56 (0.53 to 0.60)	–	–
		Secondary or higher	0.36 (0.25 to 0.48)	0.44 (0.29 to 0.60)	0.20 (0.16 to 0.25)	–	–
	Lesotho	Primary or lower	0.61 (0.10 to 0.95)	0.71 (0.10 to 1.20)	0.41 (0.09 to 0.50)	–	0.27 (0.01 to 1.02)
		Secondary or higher	0.19 (-0.16 to 0.46)	0.20 (-0.15 to 0.54)	0.16 (-0.18 to 0.28)	–	0.20 (0.01 to 0.68)
	Liberia	Primary or lower	1.16 (0.62 to 1.66)	1.29 (0.65 to 1.92)	0.89 (0.54 to 1.11)	0.32 (0.03 to 1.08)	1.47 (1.01 to 2.28)
		Secondary or higher	0.24 (-0.05 to 0.50)	0.26 (-0.05 to 0.57)	0.21 (-0.05 to 0.38)	0.22 (0.22 to 0.22)	0.42 (0.06 to 0.98)
	Madagascar	Primary or lower	2.46 (1.94 to 3.01)	3.24 (2.40 to 4.17)	0.93 (0.73 to 1.06)	–	0.10 (0.01 to 0.32)
		Secondary or higher	0.69 (0.50 to 0.95)	0.89 (0.61 to 1.28)	0.28 (0.21 to 0.34)	–	–
	Malawi	Primary or lower	0.63 (0.55 to 0.70)	0.79 (0.67 to 0.89)	0.31 (0.29 to 0.32)	–	–
		Secondary or higher	0.21 (0.15 to 0.28)	0.25 (0.17 to 0.35)	0.13 (0.10 to 0.16)	–	–
	Mali	Primary or lower	1.45 (1.23 to 1.62)	1.67 (1.38 to 1.90)	1.01 (0.93 to 1.07)	0.07 (0.00 to 0.28)	0.98 (0.67 to 1.34)
		Secondary or higher	0.40 (0.22 to 0.54)	0.44 (0.24 to 0.61)	0.31 (0.20 to 0.40)	–	0.13 (0.01 to 0.40)
	Morocco	Primary or lower	0.39 (-1.40 to 0.66)	0.48 (-1.05 to 0.95)	0.14 (-2.02 to 0.22)	1.54 (0.12 to 10.94)	1.00 (0.07 to 9.27)
		Secondary or higher	0.10 (-0.92 to 0.24)	0.12 (-0.61 to 0.33)	0.04 (-1.46 to 0.09)	1.24 (0.19 to 5.96)	0.86 (0.01 to 7.16)
	Mozambique	Primary or lower	0.49 (0.21 to 0.74)	0.53 (0.22 to 0.86)	0.39 (0.19 to 0.51)	0.13 (0.06 to 0.20)	0.43 (0.02 to 0.99)
		Secondary or higher	0.03 (-0.08 to 0.14)	0.03 (-0.08 to 0.15)	0.03 (-0.09 to 0.12)	–	0.05 (0.05 to 0.05)
	Namibia	Primary or lower	0.80 (0.53 to 0.99)	0.98 (0.60 to 1.25)	0.46 (0.39 to 0.51)	–	0.12 (0.04 to 0.39)
		Secondary or higher	0.31 (0.15 to 0.45)	0.35 (0.16 to 0.56)	0.22 (0.13 to 0.27)	–	–
	Niger	Primary or lower	4.44 (3.78 to 4.96)	6.14 (5.10 to 7.04)	1.07 (0.90 to 1.26)	–	–
		Secondary or higher	1.49 (1.13 to 1.93)	2.08 (1.57 to 2.71)	0.33 (0.23 to 0.45)	–	–
	Nigeria	Primary or lower	2.92 (2.41 to 3.37)	3.14 (2.56 to 3.66)	2.49 (2.12 to 2.78)	5.28 (4.54 to 5.96)	6.55 (5.81 to 7.25)
		Secondary or higher	0.64 (0.44 to 0.90)	0.69 (0.46 to 1.01)	0.54 (0.39 to 0.70)	0.22 (0.01 to 0.50)	1.36 (0.96 to 1.68)
	Rwanda	Primary or lower	0.62 (0.49 to 0.77)	0.74 (0.56 to 0.94)	0.40 (0.35 to 0.44)	–	0.09 (0.01 to 0.18)
		Secondary or higher	0.24 (0.10 to 0.35)	0.26 (0.11 to 0.40)	0.18 (0.09 to 0.25)	–	0.09 (0.09 to 0.09)
	Sao Tome and Principe	Primary or lower	0.86 (-0.47 to 2.17)	1.08 (-0.42 to 3.17)	0.33 (-0.58 to 0.49)	0.51 (0.03 to 10.53)	0.70 (0.06 to 3.23)
		Secondary or higher	0.39 (-0.80 to 1.39)	0.47 (-0.66 to 1.99)	0.18 (-1.11 to 0.37)	1.06 (0.04 to 8.56)	0.60 (0.07 to 4.23)
	Senegal	Primary or lower	0.96 (0.81 to 1.19)	1.14 (0.94 to 1.46)	0.61 (0.56 to 0.67)	–	0.06 (0.01 to 0.26)
		Secondary or higher	0.17 (0.11 to 0.27)	0.20 (0.12 to 0.31)	0.13 (0.09 to 0.18)	–	–
	Sierra Leone	Primary or lower	1.24 (0.83 to 1.58)	1.42 (0.92 to 1.87)	0.88 (0.67 to 1.00)	0.09 (0.00 to 0.60)	0.83 (0.46 to 1.30)
		Secondary or higher	0.43 (0.13 to 0.75)	0.46 (0.13 to 0.84)	0.35 (0.12 to 0.54)	–	0.56 (0.20 to 0.99)
	South Africa	Primary or lower	0.74 (-2.69 to 4.94)	0.82 (-1.82 to 6.80)	0.59 (-4.69 to 1.65)	1.48 (0.04 to 9.98)	1.59 (0.07 to 8.45)
		Secondary or higher	0.13 (-2.08 to 2.42)	0.13 (-1.08 to 3.32)	0.12 (-4.06 to 0.69)	0.77 (0.03 to 7.80)	0.75 (0.05 to 5.73)
	Tanzania	Primary or lower	0.53 (0.28 to 0.73)	0.60 (0.30 to 0.88)	0.38 (0.24 to 0.45)	–	0.12 (0.00 to 0.58)
		Secondary or higher	0.10 (-0.04 to 0.21)	0.10 (-0.04 to 0.23)	0.08 (-0.04 to 0.16)	–	0.45 (0.45 to 0.45)
	Togo	Primary or lower	1.41 (-1.29 to 4.23)	1.65 (-1.11 to 5.86)	0.92 (-1.68 to 1.19)	1.01 (0.06 to 5.07)	1.26 (0.06 to 5.40)
		Secondary or higher	0.38 (-1.07 to 2.00)	0.42 (-0.80 to 2.76)	0.29 (-1.61 to 0.62)	0.86 (0.01 to 4.13)	0.66 (0.02 to 4.08)
	Uganda	Primary or lower	1.67 (1.49 to 1.85)	2.05 (1.79 to 2.31)	0.92 (0.87 to 0.96)	–	0.06 (0.00 to 0.24)
		Secondary or higher	0.72 (0.57 to 0.87)	0.84 (0.65 to 1.06)	0.45 (0.40 to 0.52)	–	0.01 (0.01 to 0.01)
	Zambia	Primary or lower	0.34 (0.19 to 0.49)	0.37 (0.20 to 0.56)	0.27 (0.17 to 0.35)	–	0.11 (0.00 to 0.40)
		Secondary or higher	0.03 (-0.10 to 0.13)	0.03 (-0.09 to 0.13)	0.03 (-0.11 to 0.11)	–	0.06 (0.01 to 0.22)
	Zimbabwe	Primary or lower	2.27 (1.86 to 2.75)	2.92 (2.30 to 3.67)	0.97 (0.87 to 1.09)	–	0.06 (0.00 to 0.25)
		Secondary or higher	1.13 (0.87 to 1.45)	1.41 (1.04 to 1.91)	0.56 (0.52 to 0.62)	–	0.06 (0.06 to 0.06)
DPT3 immunization	Angola	Primary or lower	-0.94 (-6.55 to 4.44)	-0.90 (-5.13 to 5.11)	-1.00 (-9.38 to 3.09)	8.43 (1.82 to 20.77)	9.59 (2.91 to 21.54)
		Secondary or higher	-1.24 (-5.24 to 1.62)	-1.08 (-3.14 to 1.85)	-1.56 (-9.32 to 1.15)	3.91 (0.45 to 14.14)	4.95 (1.29 to 15.38)
	Benin	Primary or lower	-0.10 (-0.35 to 0.13)	-0.09 (-0.33 to 0.13)	-0.10 (-0.38 to 0.12)	0.86 (0.38 to 1.39)	2.05 (1.58 to 2.59)
		Secondary or higher	-0.28 (-0.43 to -0.17)	-0.25 (-0.36 to -0.16)	-0.35 (-0.57 to -0.20)	0.07 (0.07 to 0.07)	0.49 (0.18 to 0.94)
	Burkina Faso	Primary or lower	2.65 (2.39 to 2.95)	3.99 (3.59 to 4.44)	0.03 (0.02 to 0.05)	–	–
		Secondary or higher	0.83 (0.55 to 1.20)	1.24 (0.82 to 1.80)	0.01 (0.01 to 0.02)	–	–
	Burundi	Primary or lower	0.25 (0.05 to 0.47)	0.31 (0.06 to 0.65)	0.11 (0.05 to 0.13)	–	–
		Secondary or higher	0.10 (-0.02 to 0.27)	0.12 (-0.02 to 0.36)	0.06 (-0.02 to 0.11)	–	–
	Cameroon	Primary or lower	-0.15 (-0.47 to 0.13)	-0.14 (-0.44 to 0.13)	-0.15 (-0.53 to 0.12)	1.20 (0.60 to 1.88)	2.39 (1.79 to 3.08)
		Secondary or higher	-0.41 (-0.59 to -0.24)	-0.36 (-0.49 to -0.22)	-0.52 (-0.80 to -0.27)	0.18 (0.01 to 0.63)	1.15 (0.70 to 1.64)
	Chad	Primary or lower	2.57 (1.96 to 3.16)	2.96 (2.19 to 3.74)	1.81 (1.51 to 2.01)	0.76 (0.15 to 1.65)	1.97 (1.33 to 2.86)
		Secondary or higher	0.68 (0.35 to 1.10)	0.77 (0.37 to 1.28)	0.51 (0.30 to 0.72)	0.02 (0.02 to 0.02)	0.44 (0.05 to 1.08)
	Comoros	Primary or lower	1.49 (-1.96 to 4.60)	1.83 (-1.54 to 6.65)	0.71 (-2.89 to 0.97)	1.56 (0.06 to 12.77)	1.02 (0.06 to 9.03)
		Secondary or higher	0.74 (-2.32 to 3.05)	0.88 (-1.59 to 4.37)	0.44 (-3.85 to 0.68)	1.38 (0.08 to 10.71)	0.92 (0.03 to 8.84)
	Congo Brazzaville	Primary or lower	-1.43 (-2.68 to -0.20)	-1.24 (-2.13 to -0.19)	-1.79 (-3.81 to -0.21)	4.68 (1.95 to 7.95)	5.90 (3.15 to 9.19)
		Secondary or higher	-1.71 (-2.87 to -0.71)	-1.33 (-1.97 to -0.62)	-2.50 (-4.66 to -0.89)	4.25 (1.62 to 7.34)	5.46 (2.81 to 8.56)
	Congo Democratic Republic	Primary or lower	4.28 (3.72 to 4.94)	5.93 (5.03 to 7.00)	1.04 (0.89 to 1.17)	–	–
		Secondary or higher	2.22 (1.84 to 2.70)	3.05 (2.46 to 3.79)	0.59 (0.51 to 0.68)	–	–
	Cote d'Ivoire	Primary or lower	1.30 (-1.02 to 3.27)	1.61 (-0.88 to 4.65)	0.64 (-1.32 to 0.79)	1.24 (0.01 to 4.21)	0.77 (0.00 to 4.19)
		Secondary or higher	0.39 (-0.95 to 1.53)	0.46 (-0.71 to 2.15)	0.24 (-1.44 to 0.41)	1.09 (0.01 to 2.59)	0.69 (0.04 to 3.19)
	Egypt	Primary or lower	0.19 (0.15 to 0.23)	0.25 (0.18 to 0.31)	0.07 (0.05 to 0.09)	–	–

Indicators	Country	Educational Level	Average Annual Rate of Change (95% CrI)			Added AARC Needed to Achieve Targets (95% CrI)	
			2000–2030	2000–2020	2020–2030	UHC2030	IA2030
		Secondary or higher	0.09 (0.06 to 0.12)	0.11 (0.07 to 0.16)	0.05 (0.04 to 0.05)	–	–
	Eswatini	Primary or lower	0.38 (-0.36 to 0.85)	0.52 (-0.31 to 1.25)	0.11 (-0.46 to 0.20)	0.47 (0.20 to 1.28)	0.70 (0.19 to 2.46)
		Secondary or higher	0.17 (-0.51 to 0.46)	0.22 (-0.36 to 0.68)	0.06 (-0.77 to 0.12)	1.26 (0.55 to 2.09)	0.78 (0.10 to 3.27)
	Ethiopia	Primary or lower	2.63 (2.33 to 2.88)	3.06 (2.67 to 3.41)	1.77 (1.67 to 1.86)	0.43 (0.11 to 0.82)	1.64 (1.32 to 2.03)
		Secondary or higher	0.65 (0.47 to 0.84)	0.74 (0.51 to 0.98)	0.47 (0.36 to 0.56)	–	0.16 (0.01 to 0.41)
	Gabon	Primary or lower	2.09 (1.43 to 2.64)	2.30 (1.52 to 3.04)	1.67 (1.24 to 1.93)	2.08 (0.91 to 3.47)	3.31 (2.12 to 4.72)
		Secondary or higher	0.98 (0.04 to 1.82)	1.01 (0.04 to 1.96)	0.90 (0.04 to 1.51)	4.60 (2.88 to 6.80)	5.85 (4.12 to 8.07)
	Gambia	Primary or lower	-0.03 (-0.32 to 0.26)	-0.03 (-0.23 to 0.30)	-0.03 (-0.48 to 0.18)	–	0.15 (0.00 to 0.48)
		Secondary or higher	-0.11 (-0.31 to 0.06)	-0.10 (-0.20 to 0.07)	-0.14 (-0.52 to 0.05)	–	0.24 (0.05 to 0.35)
	Ghana	Primary or lower	0.75 (0.51 to 1.00)	0.93 (0.59 to 1.33)	0.36 (0.31 to 0.43)	–	–
		Secondary or higher	0.23 (0.09 to 0.37)	0.26 (0.09 to 0.46)	0.14 (0.07 to 0.19)	–	–
	Guinea	Primary or lower	-0.90 (-1.46 to -0.34)	-0.85 (-1.33 to -0.33)	-1.00 (-1.72 to -0.36)	5.06 (3.95 to 6.35)	6.29 (5.17 to 7.59)
		Secondary or higher	-0.99 (-1.43 to -0.67)	-0.82 (-1.13 to -0.59)	-1.35 (-2.03 to -0.83)	2.10 (1.18 to 3.46)	3.29 (2.37 to 4.66)
	Kenya	Primary or lower	1.19 (1.00 to 1.37)	1.62 (1.33 to 1.88)	0.34 (0.30 to 0.39)	–	–
		Secondary or higher	0.48 (0.36 to 0.63)	0.63 (0.45 to 0.85)	0.18 (0.15 to 0.21)	–	–
	Lesotho	Primary or lower	0.17 (-0.36 to 0.51)	0.18 (-0.33 to 0.58)	0.16 (-0.41 to 0.37)	0.28 (0.00 to 1.55)	0.59 (0.06 to 1.85)
		Secondary or higher	-0.17 (-0.60 to 0.13)	-0.16 (-0.49 to 0.13)	-0.19 (-0.83 to 0.12)	0.28 (0.01 to 1.34)	0.55 (0.03 to 1.68)
	Liberia	Primary or lower	1.62 (1.15 to 2.05)	1.91 (1.32 to 2.52)	1.00 (0.82 to 1.13)	0.12 (0.12 to 0.12)	0.45 (0.13 to 0.92)
		Secondary or higher	0.58 (0.28 to 0.91)	0.65 (0.30 to 1.09)	0.43 (0.25 to 0.57)	–	0.21 (0.01 to 0.66)
	Madagascar	Primary or lower	1.97 (1.42 to 2.42)	2.54 (1.71 to 3.29)	0.82 (0.66 to 0.93)	–	0.14 (0.04 to 0.33)
		Secondary or higher	0.40 (0.24 to 0.57)	0.50 (0.28 to 0.75)	0.19 (0.15 to 0.24)	–	–
	Malawi	Primary or lower	0.53 (0.47 to 0.59)	0.66 (0.57 to 0.75)	0.27 (0.26 to 0.29)	–	–
		Secondary or higher	0.13 (0.08 to 0.19)	0.15 (0.09 to 0.22)	0.09 (0.06 to 0.11)	–	–
	Mali	Primary or lower	1.48 (1.32 to 1.66)	1.73 (1.53 to 1.98)	0.98 (0.92 to 1.03)	–	0.64 (0.39 to 0.86)
		Secondary or higher	0.34 (0.22 to 0.49)	0.38 (0.24 to 0.57)	0.26 (0.18 to 0.34)	–	0.06 (0.00 to 0.18)
	Morocco	Primary or lower	0.21 (-0.40 to 0.37)	0.28 (-0.33 to 0.54)	0.06 (-0.53 to 0.11)	2.66 (2.66 to 2.66)	0.30 (0.04 to 3.85)
		Secondary or higher	0.06 (-0.29 to 0.15)	0.07 (-0.22 to 0.21)	0.02 (-0.44 to 0.05)	1.22 (1.22 to 1.22)	0.42 (0.06 to 2.39)
	Mozambique	Primary or lower	0.83 (0.61 to 1.09)	0.98 (0.69 to 1.36)	0.51 (0.44 to 0.56)	–	0.10 (0.00 to 0.33)
		Secondary or higher	0.17 (0.07 to 0.34)	0.19 (0.07 to 0.41)	0.13 (0.06 to 0.21)	–	–
	Namibia	Primary or lower	0.25 (-0.09 to 0.48)	0.27 (-0.09 to 0.55)	0.22 (-0.09 to 0.34)	0.27 (0.08 to 0.27)	0.32 (0.01 to 1.17)
		Secondary or higher	-0.09 (-0.44 to 0.12)	-0.08 (-0.38 to 0.13)	-0.09 (-0.55 to 0.11)	0.17 (0.03 to 0.64)	0.39 (0.03 to 1.39)
	Niger	Primary or lower	3.39 (3.00 to 3.90)	4.82 (4.18 to 5.67)	0.58 (0.46 to 0.71)	–	–
		Secondary or higher	1.25 (0.85 to 1.62)	1.75 (1.17 to 2.31)	0.23 (0.16 to 0.31)	–	–
	Nigeria	Primary or lower	3.48 (3.20 to 3.84)	4.06 (3.68 to 4.55)	2.32 (2.22 to 2.45)	0.80 (0.48 to 1.17)	2.03 (1.70 to 2.39)
		Secondary or higher	0.89 (0.74 to 1.10)	1.04 (0.85 to 1.32)	0.59 (0.53 to 0.66)	–	0.11 (0.01 to 0.26)
	Rwanda	Primary or lower	0.49 (0.41 to 0.58)	0.64 (0.52 to 0.79)	0.18 (0.15 to 0.20)	–	–
		Secondary or higher	0.26 (0.17 to 0.35)	0.32 (0.21 to 0.46)	0.13 (0.10 to 0.16)	–	–
	Sao Tome and Principe	Primary or lower	0.68 (-0.20 to 1.72)	0.91 (-0.19 to 2.55)	0.21 (-0.23 to 0.34)	0.20 (0.15 to 4.44)	0.46 (0.03 to 5.63)
		Secondary or higher	0.39 (-0.42 to 1.31)	0.49 (-0.35 to 1.81)	0.14 (-0.53 to 0.29)	0.90 (0.32 to 6.97)	0.66 (0.15 to 8.17)
	Senegal	Primary or lower	0.97 (0.82 to 1.11)	1.28 (1.05 to 1.47)	0.37 (0.35 to 0.39)	–	–
		Secondary or higher	0.22 (0.15 to 0.31)	0.28 (0.19 to 0.40)	0.11 (0.09 to 0.15)	–	–
	Sierra Leone	Primary or lower	1.95 (1.56 to 2.41)	2.57 (2.00 to 3.27)	0.71 (0.66 to 0.77)	–	–
		Secondary or higher	0.99 (0.70 to 1.32)	1.25 (0.87 to 1.72)	0.46 (0.37 to 0.55)	–	–
	South Africa	Primary or lower	1.23 (-1.57 to 5.34)	1.36 (-1.32 to 7.03)	0.92 (-2.07 to 1.89)	1.14 (0.05 to 5.11)	1.65 (0.07 to 6.19)
		Secondary or higher	0.38 (-1.96 to 3.54)	0.41 (-1.45 to 4.68)	0.34 (-2.98 to 1.29)	1.14 (0.08 to 5.44)	1.51 (0.09 to 6.21)
	Tanzania	Primary or lower	0.21 (-0.00 to 0.38)	0.23 (-0.00 to 0.44)	0.18 (-0.00 to 0.27)	–	0.12 (0.00 to 0.47)
		Secondary or higher	-0.03 (-0.16 to 0.06)	-0.03 (-0.14 to 0.06)	-0.03 (-0.19 to 0.05)	–	0.04 (0.01 to 0.07)
	Togo	Primary or lower	1.14 (-0.04 to 3.17)	1.47 (-0.04 to 4.59)	0.44 (-0.04 to 0.54)	0.35 (0.01 to 0.68)	0.26 (0.01 to 1.86)
		Secondary or higher	0.42 (-0.20 to 1.62)	0.51 (-0.18 to 2.30)	0.20 (-0.25 to 0.32)	0.69 (0.69 to 0.69)	0.40 (0.08 to 1.87)
	Uganda	Primary or lower	1.84 (1.67 to 2.01)	2.39 (2.14 to 2.65)	0.73 (0.68 to 0.77)	–	–
		Secondary or higher	0.91 (0.76 to 1.08)	1.13 (0.93 to 1.38)	0.45 (0.39 to 0.52)	–	–
	Zambia	Primary or lower	0.61 (0.48 to 0.76)	0.71 (0.54 to 0.92)	0.41 (0.35 to 0.45)	–	0.04 (0.04 to 0.04)
		Secondary or higher	0.14 (0.07 to 0.24)	0.15 (0.07 to 0.26)	0.12 (0.06 to 0.18)	–	–
	Zimbabwe	Primary or lower	2.50 (2.01 to 3.00)	3.31 (2.55 to 4.05)	0.97 (0.85 to 1.10)	–	–
		Secondary or higher	1.38 (1.06 to 1.76)	1.75 (1.30 to 2.32)	0.64 (0.59 to 0.70)	–	–

Notes: AARC = Average Annual Rate of Change. The additional AARC required to reach global targets was calculated as the difference between the projected AARC for 2020–2030 and the AARC needed to achieve the target coverage by 2030.

Table L. Wealth-related inequality in childhood immunization coverage, 2000–2030

Indicators	Country	Index	Estimates of Inequalities			Changes in Inequalities		
			Year 2000	Year 2020	Year 2030	2000–2020	2020–2030	2000–2030
Full immunization	Angola	SII	58.0 (40.0 to 64.2)*	46.4 (36.8 to 55.2)*	36.1 (11.1 to 51.5)*	-0.6 (-0.9 to 0.4)	-1.1 (-2.6 to -0.2)*	-0.8 (-1.2 to 0.1)
		RII	4.3 (1.8 to 9.2)*	5.5 (4.0 to 7.5)*	5.6 (2.9 to 9.0)*	1.1 (-3.6 to 6.8)	0.3 (-3.4 to 2.1)	0.9 (-3.5 to 5.3)
	Benin	SII	36.0 (33.4 to 38.7)*	25.2 (22.0 to 27.7)*	19.7 (15.5 to 22.8)*	-0.5 (-0.7 to -0.4)*	-0.6 (-0.7 to -0.4)*	-0.6 (-0.7 to -0.4)*
		RII	1.9 (1.8 to 2.1)*	1.5 (1.4 to 1.6)*	1.3 (1.3 to 1.4)*	-1.3 (-1.6 to -1.0)*	-1.1 (-1.3 to -0.9)*	-1.2 (-1.5 to -0.9)*
	Burkina Faso	SII	23.8 (18.8 to 27.7)*	1.1 (0.6 to 1.5)*	0.1 (0.0 to 0.1)*	-1.1 (-1.3 to -0.9)*	-0.1 (-0.1 to -0.1)*	-0.8 (-0.9 to -0.6)*
		RII	2.1 (1.8 to 2.5)*	1.0 (1.0 to 1.0)*	1.0 (1.0 to 1.0)*	-3.7 (-4.4 to -3.0)*	-0.1 (-0.1 to -0.1)*	-2.5 (-3.0 to -2.0)*
	Burundi	SII	5.7 (3.4 to 9.6)*	3.7 (1.2 to 6.6)*	1.7 (-1.2 to 5.3)	-0.1 (-0.3 to 0.0)	-0.2 (-0.3 to -0.1)*	-0.1 (-0.3 to 0.0)*
		RII	1.1 (1.0 to 1.1)*	1.0 (1.0 to 1.1)*	1.0 (1.0 to 1.1)	-0.1 (-0.3 to 0.1)	-0.2 (-0.3 to -0.1)*	-0.1 (-0.3 to 0.0)
	Cameroon	SII	42.0 (38.6 to 45.8)*	32.9 (28.9 to 37.0)*	27.9 (23.2 to 32.4)*	-0.5 (-0.6 to -0.3)*	-0.5 (-0.7 to -0.3)*	-0.5 (-0.6 to -0.3)*
		RII	2.2 (2.0 to 2.4)*	1.8 (1.6 to 1.9)*	1.6 (1.5 to 1.8)*	-1.0 (-1.5 to -0.6)*	-1.0 (-1.4 to -0.6)*	-1.0 (-1.5 to -0.6)*
	Chad	SII	11.5 (9.4 to 14.8)*	16.2 (11.6 to 21.6)*	12.0 (6.2 to 17.3)*	0.2 (0.0 to 0.5)	-0.4 (-0.6 to -0.2)*	0.0 (-0.2 to 0.2)
		RII	2.7 (2.2 to 3.4)*	1.5 (1.4 to 1.8)*	1.2 (1.1 to 1.4)*	-2.7 (-3.4 to -2.1)*	-2.2 (-2.8 to -1.8)*	-2.6 (-3.2 to -2.0)*
	Comoros	SII	37.5 (24.2 to 47.5)*	22.6 (12.7 to 35.7)*	14.4 (3.4 to 31.3)*	-0.8 (-1.2 to 0.1)	-0.8 (-1.3 to -0.3)*	-0.8 (-1.2 to -0.1)*
		RII	2.1 (1.4 to 3.4)*	1.4 (1.2 to 1.8)*	1.2 (1.0 to 1.9)*	-2.0 (-5.0 to 0.7)	-1.2 (-1.8 to 0.4)	-1.8 (-3.9 to 0.6)
	Congo Brazzaville	SII	34.2 (29.0 to 38.8)*	26.5 (21.2 to 32.2)*	19.1 (12.1 to 26.4)*	-0.4 (-0.5 to -0.2)*	-0.8 (-1.0 to -0.5)*	-0.5 (-0.7 to -0.3)*
		RII	1.7 (1.6 to 2.0)*	1.9 (1.7 to 2.2)*	1.9 (1.6 to 2.3)*	0.5 (-0.2 to 1.3)	-0.3 (-0.8 to 0.3)	0.2 (-0.4 to 1.0)
	Congo Democratic Republic	SII	29.7 (25.5 to 34.4)*	24.6 (21.2 to 28.2)*	12.7 (8.3 to 17.1)*	-0.3 (-0.4 to -0.1)*	-1.2 (-1.4 to -1.0)*	-0.6 (-0.7 to -0.5)*
		RII	3.2 (2.7 to 3.7)*	1.5 (1.4 to 1.6)*	1.2 (1.1 to 1.2)*	-3.9 (-4.7 to -3.2)*	-2.2 (-2.4 to -1.9)*	-3.4 (-3.9 to -2.7)*
	Cote d'Ivoire	SII	34.0 (22.4 to 41.3)*	23.9 (14.8 to 31.8)*	15.7 (3.8 to 25.7)*	-0.5 (-0.7 to -0.2)*	-0.8 (-1.3 to -0.4)*	-0.6 (-0.8 to -0.3)*
		RII	2.3 (1.6 to 3.5)*	1.5 (1.2 to 2.1)*	1.3 (1.0 to 2.0)*	-2.3 (-4.9 to 0.7)	-1.4 (-2.1 to 0.0)*	-2.0 (-3.9 to 0.5)
	Egypt	SII	4.2 (3.1 to 5.1)*	3.7 (0.9 to 6.7)*	0.7 (-4.2 to 5.6)	0.0 (-0.1 to 0.1)	-0.3 (-0.5 to -0.1)*	-0.1 (-0.3 to 0.0)
		RII	1.0 (1.0 to 1.1)*	1.0 (1.0 to 1.1)*	1.0 (0.9 to 1.1)	0.0 (-0.1 to 0.2)	-0.4 (-0.8 to 0.0)*	-0.1 (-0.3 to 0.1)
	Eswatini	SII	8.5 (1.4 to 23.0)*	0.9 (-2.2 to 7.7)	0.1 (-3.7 to 5.9)	-0.3 (-1.1 to 0.0)	-0.1 (-0.4 to 0.0)	-0.3 (-0.8 to 0.0)*
		RII	1.1 (1.0 to 1.4)*	1.0 (1.0 to 1.1)	1.0 (1.0 to 1.1)	-0.4 (-1.5 to 0.0)	-0.1 (-0.5 to 0.0)	-0.3 (-1.0 to 0.0)*
	Ethiopia	SII	24.0 (21.7 to 26.7)*	33.2 (29.9 to 36.7)*	24.3 (20.0 to 28.7)*	0.5 (0.3 to 0.6)*	-0.9 (-1.1 to -0.7)*	0.0 (-0.1 to 0.1)
		RII	4.5 (4.0 to 5.2)*	2.0 (1.9 to 2.2)*	1.4 (1.3 to 1.5)*	-4.0 (-4.5 to -3.5)*	-3.3 (-3.7 to -3.0)*	-3.8 (-4.2 to -3.4)*
	Gabon	SII	6.2 (2.6 to 10.6)*	0.4 (-7.0 to 6.5)	-3.9 (-11.4 to 2.0)	-0.3 (-0.5 to -0.1)*	-0.4 (-0.6 to -0.2)*	-0.3 (-0.5 to -0.2)*
		RII	1.4 (1.2 to 1.8)*	1.0 (0.9 to 1.1)	0.9 (0.8 to 1.0)	-1.8 (-2.4 to -1.2)*	-0.6 (-1.1 to -0.1)*	-1.4 (-2.0 to -0.8)*
	Gambia	SII	0.2 (-5.9 to 5.7)	-4.6 (-8.1 to -1.9)*	-5.6 (-10.3 to -2.8)*	-0.3 (-0.4 to -0.1)*	-0.1 (-0.3 to 0.1)	-0.2 (-0.3 to -0.1)*
		RII	1.0 (0.9 to 1.1)	0.9 (0.9 to 1.0)*	0.9 (0.9 to 1.0)*	-0.3 (-0.5 to -0.1)*	-0.1 (-0.4 to 0.1)	-0.2 (-0.4 to 0.0)*
	Ghana	SII	20.3 (15.2 to 25.7)*	7.1 (3.9 to 10.6)*	3.3 (1.0 to 6.4)*	-0.6 (-0.9 to -0.5)*	-0.4 (-0.5 to -0.3)*	-0.6 (-0.7 to -0.4)*
		RII	1.3 (1.2 to 1.4)*	1.1 (1.0 to 1.1)*	1.0 (1.0 to 1.1)*	-1.0 (-1.4 to -0.7)*	-0.5 (-0.6 to -0.3)*	-0.8 (-1.1 to -0.6)*
	Guinea	SII	33.6 (29.2 to 38.9)*	19.6 (15.6 to 23.7)*	11.4 (7.5 to 15.7)*	-0.7 (-0.9 to -0.6)*	-0.8 (-1.0 to -0.7)*	-0.7 (-0.9 to -0.6)*
		RII	1.9 (1.7 to 2.2)*	2.1 (1.8 to 2.4)*	1.9 (1.6 to 2.3)*	0.3 (-0.3 to 0.9)	-0.7 (-1.3 to -0.1)*	0.0 (-0.6 to 0.6)
	Kenya	SII	35.0 (31.5 to 38.9)*	12.0 (10.0 to 13.7)*	5.0 (3.6 to 6.3)*	-1.1 (-1.3 to -1.0)*	-0.7 (-0.8 to -0.6)*	-1.0 (-1.2 to -0.9)*
		RII	1.9 (1.7 to 2.1)*	1.1 (1.1 to 1.2)*	1.1 (1.0 to 1.1)*	-2.4 (-2.9 to -2.0)*	-0.8 (-0.9 to -0.7)*	-1.9 (-2.2 to -1.6)*
	Lesotho	SII	17.4 (12.3 to 22.0)*	8.4 (2.9 to 13.4)*	3.9 (-1.7 to 9.6)	-0.5 (-0.7 to -0.3)*	-0.4 (-0.5 to -0.3)*	-0.5 (-0.6 to -0.3)*
		RII	1.3 (1.2 to 1.4)*	1.1 (1.0 to 1.2)*	1.1 (1.0 to 1.1)	-0.7 (-1.1 to -0.3)*	-0.6 (-0.7 to -0.4)*	-0.7 (-0.9 to -0.4)*
	Liberia	SII	32.0 (26.5 to 37.4)*	20.9 (15.4 to 25.7)*	13.9 (8.7 to 19.2)*	-0.5 (-0.7 to -0.4)*	-0.7 (-0.8 to -0.6)*	-0.6 (-0.7 to -0.5)*
		RII	2.1 (1.9 to 2.4)*	1.4 (1.3 to 1.5)*	1.2 (1.1 to 1.3)*	-2.0 (-2.6 to -1.6)*	-1.4 (-1.7 to -1.2)*	-1.8 (-2.3 to -1.4)*
	Madagascar	SII	52.2 (47.6 to 56.5)*	25.1 (19.6 to 33.2)*	12.6 (7.6 to 23.1)*	-1.3 (-1.6 to -0.9)*	-1.2 (-1.4 to -1.0)*	-1.3 (-1.5 to -0.9)*
		RII	2.9 (2.5 to 3.4)*	1.4 (1.3 to 1.6)*	1.2 (1.1 to 1.3)*	-3.7 (-4.6 to -2.4)*	-1.7 (-1.9 to -1.5)*	-3.0 (-3.6 to -2.1)*
	Malawi	SII	20.2 (17.1 to 23.1)*	6.0 (4.5 to 7.6)*	2.6 (1.4 to 4.0)*	-0.7 (-0.8 to -0.6)*	-0.3 (-0.4 to -0.3)*	-0.6 (-0.7 to -0.5)*
		RII	1.3 (1.3 to 1.4)*	1.1 (1.1 to 1.1)*	1.0 (1.0 to 1.0)*	-1.0 (-1.2 to -0.9)*	-0.4 (-0.5 to -0.3)*	-0.8 (-1.0 to -0.7)*
	Mali	SII	28.4 (26.0 to 31.6)*	17.9 (14.8 to 21.1)*	10.3 (6.9 to 13.7)*	-0.5 (-0.7 to -0.4)*	-0.8 (-0.9 to -0.6)*	-0.6 (-0.7 to -0.5)*
		RII	2.1 (2.0 to 2.3)*	1.3 (1.2 to 1.4)*	1.1 (1.1 to 1.2)*	-2.3 (-2.7 to -2.0)*	-1.4 (-1.5 to -1.3)*	-2.0 (-2.3 to -1.8)*
	Morocco	SII	19.5 (13.2 to 26.6)*	6.4 (1.1 to 24.3)*	3.3 (0.2 to 27.4)*	-0.7 (-1.2 to 0.4)	-0.2 (-0.4 to 0.3)	-0.5 (-0.9 to 0.4)
		RII	1.3 (1.2 to 1.4)*	1.1 (1.0 to 1.4)*	1.0 (1.0 to 1.5)*	-0.8 (-1.5 to 0.7)	-0.2 (-0.4 to 0.8)	-0.6 (-1.0 to 0.7)
	Mozambique	SII	40.6 (36.1 to 44.1)*	29.1 (24.9 to 33.3)*	23.5 (18.3 to 28.9)*	-0.6 (-0.8 to -0.4)*	-0.5 (-0.7 to -0.4)*	-0.6 (-0.8 to -0.4)*
		RII	1.9 (1.7 to 2.1)*	1.5 (1.4 to 1.6)*	1.4 (1.3 to 1.5)*	-1.1 (-1.7 to -0.6)*	-0.9 (-1.2 to -0.6)*	-1.1 (-1.5 to -0.6)*
	Namibia	SII	12.9 (8.2 to 17.8)*	3.5 (-1.3 to 7.0)	0.1 (-4.7 to 3.2)	-0.5 (-0.6 to -0.4)*	-0.3 (-0.4 to -0.2)*	-0.4 (-0.6 to -0.3)*
		RII	1.2 (1.1 to 1.3)*	1.0 (1.0 to 1.1)	1.0 (0.9 to 1.0)	-0.7 (-0.9 to -0.5)*	-0.4 (-0.6 to -0.3)*	-0.6 (-0.8 to -0.5)*
	Niger	SII	21.0 (17.4 to 23.8)*	17.5 (14.1 to 20.9)*	4.6 (2.8 to 7.0)*	-0.2 (-0.3 to 0.0)*	-1.3 (-1.5 to -1.1)*	-0.5 (-0.6 to -0.5)*
		RII	3.5 (3.0 to 4.1)*	1.3 (1.2 to 1.3)*	1.0 (1.0 to 1.1)*	-5.0 (-5.7 to -4.3)*	-1.8 (-2.1 to -1.4)*	-3.9 (-4.4 to -3.4)*
	Nigeria	SII	46.5 (43.2 to 49.6)*	62.7 (60.9 to 64.5)*	58.0 (55.2 to 60.6)*	0.8 (0.6 to 1.0)*	-0.5 (-0.7 to -0.3)*	0.4 (0.3 to 0.5)*
		RII	16.0 (13.9 to 17.8)*	5.3 (4.9 to 5.7)*	3.0 (2.7 to 3.4)*	-5.4 (-5.9 to -4.8)*	-5.3 (-5.8 to -4.8)*	-5.4 (-5.9 to -4.8)*
	Rwanda	SII	7.6 (4.7 to 10.7)*	-0.4 (-2.1 to 0.9)	-1.2 (-2.4 to -0.2)*	-0.4 (-0.5 to -0.3)*	-0.1 (-0.1 to 0.0)*	-0.3 (-0.4 to -0.2)*
		RII	1.1 (1.1 to 1.2)*	1.0 (1.0 to 1.0)	1.0 (1.0 to 1.0)*	-0.6 (-0.8 to -0.4)*	-0.1 (-0.1 to 0.0)*	-0.4 (-0.5 to -0.3)*
	Sao Tome and Principe	SII	20.8 (8.2 to 35.9)*	6.3 (-0.1 to 19.8)	2.3 (-1.4 to 16.1)	-0.7 (-1.5 to 0.0)	-0.3 (-0.6 to -0.1)*	-0.6 (-1.1 to -0.1)*
		RII	1.3 (1.1 to 1.9)*	1.1 (1.0 to 1.3)	1.0 (1.0 to 1.3)	-1.0 (-2.7 to 0.2)	-0.4 (-0.7 to 0.0)	-0.8 (-2.0 to 0.0)
	Senegal	SII	28.5 (24.3 to 31.5)*	10.2 (8.5 to 12.1)*	4.5 (3.0 to 6.2)*	-0.9 (-1.0 to -0.7)*	-0.6 (-0.6 to -0.5)*	-0.8 (-0.9 to -0.7)*
		RII	1.6 (1.5 to 1.7)*	1.1 (1.1 to 1.2)*	1.1 (1.0 to 1.1)*	-1.8 (-2.2 to -1.5)*	-0.7 (-0.8 to -0.6)*	-1.5 (-1.7 to -1.2)*
	Sierra Leone	SII	8.2 (3.5 to 12.6)*	-1.8 (-6.1 to 1.4)	-5.2 (-9.4 to -2.1)*	-0.5 (-0.7 to -0.4)*	-0.3 (-0.4 to -0.2)*	-0.4 (-0.6 to -0.3)*
		RII	1.2 (1.1 to 1.3)*	1.0 (0.9 to 1.0)	0.9 (0.9 to 1.0)*	-1.0 (-1.4 to -0.7)*	-0.4 (-0.6 to -0.2)*	-0.8 (-1.1 to -0.5)*
	South Africa	SII	7.1 (-3.4 to 17.5)	-2.0 (-12.6 to 9.8)	-5.4 (-15.6 to 3.5)	-0.5 (-0.7 to -0.2)*	-0.4 (-0.6 to 0.1)	-0.4 (-0.6 to -0.2)*
		RII	1.2 (0.9 to 1.7)	1.0 (0.8 to 1.2)	0.9 (0.8 to 1.1)	-1.0 (-2.2 to -0.3)*	-0.6 (-1.5 to 0.3)	-0.9 (-1.8 to -0.3)*
	Tanzania	SII	26.8 (23.0 to 30.9)*	16.1 (12.9 to 19.7)*	11.7 (8.2 to 15.6)*	-0.5 (-0.7 to -0.4)*	-0.4 (-0.6 to -0.3)*	-0.5 (-0.7 to -0.3)*
		RII	1.4 (1.3 to 1.5)*	1.2 (1.2 to 1.3)*	1.2 (1.1 to 1.2)*	-0.8 (-1.2 to -0.5)*	-0.6 (-0.7 to -0.4)*	-0.7 (-1.0 to -0.5)*

Indicators	Country	Index	Estimates of Inequalities			Changes in Inequalities		
			Year 2000	Year 2020	Year 2030	2000–2020	2020–2030	2000–2030
	Togo	SII	17.9 (8.2 to 25.7)*	5.8 (-0.1 to 11.6)	1.6 (-2.8 to 7.0)	-0.6 (-0.8 to -0.3)*	-0.4 (-0.6 to -0.2)*	-0.5 (-0.7 to -0.2)*
		RII	1.4 (1.2 to 1.9)*	1.1 (1.0 to 1.2)	1.0 (1.0 to 1.1)	-1.4 (-2.8 to -0.5)*	-0.6 (-0.9 to -0.2)*	-1.1 (-2.1 to -0.5)*
	Uganda	SII	13.9 (10.6 to 17.4)*	2.9 (-0.1 to 6.5)	-1.1 (-4.1 to 2.0)	-0.5 (-0.7 to -0.4)*	-0.4 (-0.5 to -0.3)*	-0.5 (-0.6 to -0.4)*
		RII	1.4 (1.3 to 1.5)*	1.0 (1.0 to 1.1)	1.0 (1.0 to 1.0)	-1.4 (-1.8 to -1.1)*	-0.5 (-0.7 to -0.4)*	-1.1 (-1.4 to -0.9)*
	Zambia	SII	24.9 (21.8 to 28.5)*	12.3 (9.9 to 14.5)*	7.6 (4.9 to 10.0)*	-0.6 (-0.8 to -0.5)*	-0.5 (-0.6 to -0.4)*	-0.6 (-0.7 to -0.5)*
		RII	1.4 (1.4 to 1.5)*	1.2 (1.1 to 1.2)*	1.1 (1.1 to 1.1)*	-1.0 (-1.3 to -0.8)*	-0.6 (-0.7 to -0.5)*	-0.9 (-1.1 to -0.7)*
	Zimbabwe	SII	24.5 (19.4 to 30.2)*	7.8 (5.0 to 11.0)*	2.3 (0.7 to 4.3)*	-0.8 (-1.0 to -0.7)*	-0.5 (-0.7 to -0.4)*	-0.7 (-0.9 to -0.6)*
		RII	1.7 (1.5 to 2.0)*	1.1 (1.1 to 1.1)*	1.0 (1.0 to 1.0)*	-2.2 (-2.8 to -1.8)*	-0.7 (-0.9 to -0.5)*	-1.7 (-2.1 to -1.4)*
BCG immunization	Angola	SII	49.6 (19.6 to 69.4)*	53.4 (41.9 to 64.6)*	56.0 (21.7 to 72.9)*	0.2 (-1.3 to 2.3)	0.2 (-1.9 to 1.0)	0.2 (-1.5 to 1.8)
		RII	2.0 (1.2 to 4.8)*	2.2 (1.8 to 3.2)*	2.4 (1.3 to 7.0)*	0.6 (-5.1 to 4.8)	0.6 (-2.8 to 8.2)	0.6 (-4.4 to 5.9)
	Benin	SII	19.9 (17.5 to 22.6)*	24.8 (22.2 to 28.0)*	27.4 (23.3 to 33.1)*	0.2 (0.0 to 0.5)*	0.3 (0.0 to 0.5)*	0.3 (0.0 to 0.5)*
		RII	1.3 (1.2 to 1.3)*	1.3 (1.3 to 1.4)*	1.4 (1.3 to 1.5)*	0.3 (0.1 to 0.6)*	0.4 (0.1 to 0.8)*	0.4 (0.1 to 0.7)*
	Burkina Faso	SII	36.1 (30.6 to 41.7)*	0.5 (0.3 to 0.9)*	0.0 (0.0 to 0.1)*	-1.8 (-2.1 to -1.5)*	0.0 (-0.1 to 0.0)*	-1.2 (-1.4 to -1.0)*
		RII	1.7 (1.5 to 1.9)*	1.0 (1.0 to 1.0)*	1.0 (1.0 to 1.0)*	-2.6 (-3.1 to -2.1)*	0.0 (-0.1 to 0.0)*	-1.8 (-2.1 to -1.4)*
	Burundi	SII	1.7 (0.6 to 3.9)*	4.0 (2.2 to 6.4)*	6.3 (2.0 to 15.2)*	0.1 (-0.1 to 0.3)	0.2 (0.0 to 0.9)	0.2 (-0.1 to 0.5)
		RII	1.0 (1.0 to 1.0)*	1.0 (1.0 to 1.1)*	1.1 (1.0 to 1.2)*	0.1 (-0.1 to 0.3)	0.2 (0.0 to 1.1)	0.2 (-0.1 to 0.5)
	Cameroon	SII	23.3 (20.0 to 27.2)*	24.2 (20.9 to 27.5)*	24.9 (19.4 to 30.4)*	0.0 (-0.2 to 0.3)	0.0 (-0.2 to 0.3)	0.0 (-0.2 to 0.3)
		RII	1.3 (1.3 to 1.4)*	1.3 (1.3 to 1.4)*	1.3 (1.2 to 1.5)*	0.0 (-0.2 to 0.4)	0.0 (-0.2 to 0.5)	0.0 (-0.2 to 0.4)
	Chad	SII	30.0 (25.5 to 34.4)*	26.3 (22.6 to 30.4)*	17.9 (13.6 to 23.2)*	-0.2 (-0.4 to 0.1)	-0.8 (-1.1 to -0.6)*	-0.4 (-0.6 to -0.2)*
		RII	2.2 (1.9 to 2.6)*	1.5 (1.4 to 1.6)*	1.2 (1.2 to 1.3)*	-2.1 (-2.8 to -1.3)*	-1.6 (-1.9 to -1.2)*	-1.9 (-2.5 to -1.3)*
	Comoros	SII	23.6 (7.5 to 42.0)*	11.3 (4.8 to 23.5)*	7.1 (1.1 to 30.0)*	-0.6 (-1.7 to 0.5)	-0.4 (-0.7 to 0.5)	-0.5 (-1.3 to 0.6)
		RII	1.4 (1.1 to 2.2)*	1.1 (1.1 to 1.3)*	1.1 (1.0 to 1.5)*	-0.9 (-3.5 to 0.7)	-0.4 (-0.8 to 1.1)	-0.8 (-2.5 to 0.9)
	Congo Brazzaville	SII	27.4 (19.9 to 37.4)*	6.8 (4.2 to 10.0)*	3.1 (1.2 to 6.6)*	-1.0 (-1.6 to -0.5)*	-0.4 (-0.4 to -0.3)*	-0.8 (-1.2 to -0.5)*
		RII	1.4 (1.3 to 1.6)*	1.1 (1.0 to 1.1)*	1.0 (1.0 to 1.1)*	-1.3 (-2.1 to -0.6)*	-0.4 (-0.5 to -0.3)*	-1.0 (-1.5 to -0.6)*
	Congo Democratic Republic	SII	40.1 (35.3 to 45.0)*	12.9 (10.4 to 15.8)*	5.3 (3.2 to 7.9)*	-1.3 (-1.6 to -1.1)*	-0.8 (-0.9 to -0.7)*	-1.2 (-1.4 to -1.0)*
		RII	2.0 (1.8 to 2.3)*	1.2 (1.1 to 1.2)*	1.1 (1.0 to 1.1)*	-2.7 (-3.5 to -2.0)*	-0.9 (-1.0 to -0.8)*	-2.1 (-2.6 to -1.6)*
	Cote d'Ivoire	SII	35.5 (17.6 to 50.8)*	17.5 (7.1 to 35.7)*	10.9 (1.5 to 42.3)*	-1.0 (-2.0 to 0.8)	-0.6 (-0.9 to 0.9)	-0.8 (-1.6 to 0.8)
		RII	1.6 (1.2 to 2.6)*	1.2 (1.1 to 1.6)*	1.1 (1.0 to 1.9)*	-1.5 (-4.3 to 1.2)	-0.7 (-1.0 to 1.8)	-1.3 (-3.1 to 1.4)
	Egypt	SII	1.5 (0.9 to 2.3)*	1.8 (1.1 to 3.2)*	2.0 (0.9 to 4.6)*	0.0 (0.0 to 0.1)	0.0 (0.0 to 0.1)	0.0 (0.0 to 0.1)
		RII	1.0 (1.0 to 1.0)*	1.0 (1.0 to 1.0)*	1.0 (1.0 to 1.0)*	0.0 (0.0 to 0.1)	0.0 (0.0 to 0.2)	0.0 (0.0 to 0.1)
	Eswatini	SII	2.7 (-1.6 to 8.4)	0.4 (-0.2 to 4.2)	0.2 (-0.1 to 5.3)	-0.1 (-0.4 to 0.1)	0.0 (-0.1 to 0.1)	-0.1 (-0.3 to 0.1)
		RII	1.0 (1.0 to 1.1)	1.0 (1.0 to 1.0)	1.0 (1.0 to 1.1)	-0.1 (-0.4 to 0.1)	0.0 (-0.1 to 0.1)	-0.1 (-0.3 to 0.1)
	Ethiopia	SII	30.4 (26.8 to 34.1)*	22.6 (19.6 to 25.5)*	16.7 (13.5 to 20.0)*	-0.4 (-0.6 to -0.2)*	-0.6 (-0.7 to -0.5)*	-0.5 (-0.6 to -0.3)*
		RII	1.8 (1.6 to 1.9)*	1.4 (1.3 to 1.4)*	1.2 (1.2 to 1.3)*	-1.3 (-1.7 to -0.9)*	-1.0 (-1.1 to -0.8)*	-1.2 (-1.5 to -0.9)*
	Gabon	SII	15.5 (12.0 to 19.4)*	8.4 (6.0 to 11.3)*	5.9 (3.6 to 9.7)*	-0.4 (-0.6 to -0.1)*	-0.2 (-0.3 to -0.1)*	-0.3 (-0.5 to -0.1)*
		RII	1.2 (1.1 to 1.3)*	1.1 (1.1 to 1.1)*	1.1 (1.0 to 1.1)*	-0.4 (-0.7 to -0.1)*	-0.3 (-0.3 to -0.1)*	-0.4 (-0.6 to -0.1)*
	Gambia	SII	1.1 (-0.5 to 5.5)	0.6 (-0.1 to 1.2)	0.3 (-0.1 to 1.3)	0.0 (-0.2 to 0.0)	0.0 (-0.1 to 0.0)	0.0 (-0.2 to 0.0)
		RII	1.0 (1.0 to 1.1)	1.0 (1.0 to 1.0)	1.0 (1.0 to 1.0)	0.0 (-0.3 to 0.0)	0.0 (-0.1 to 0.0)	0.0 (-0.2 to 0.0)
	Ghana	SII	12.7 (9.3 to 17.6)*	2.5 (1.5 to 4.0)*	1.1 (0.4 to 2.2)*	-0.5 (-0.7 to -0.3)*	-0.1 (-0.2 to -0.1)*	-0.4 (-0.6 to -0.2)*
		RII	1.2 (1.1 to 1.2)*	1.0 (1.0 to 1.0)*	1.0 (1.0 to 1.0)*	-0.6 (-0.9 to -0.3)*	-0.1 (-0.2 to -0.1)*	-0.4 (-0.6 to -0.3)*
	Guinea	SII	19.9 (16.3 to 24.1)*	31.7 (27.7 to 36.1)*	37.6 (30.8 to 43.7)*	0.6 (0.3 to 0.8)*	0.6 (0.3 to 0.8)*	0.6 (0.3 to 0.8)*
		RII	1.3 (1.2 to 1.3)*	1.5 (1.4 to 1.7)*	1.8 (1.5 to 2.0)*	1.0 (0.5 to 1.4)*	1.3 (0.6 to 2.1)*	1.1 (0.6 to 1.6)*
	Kenya	SII	23.3 (18.6 to 28.3)*	2.8 (2.1 to 3.7)*	0.8 (0.5 to 1.4)*	-1.0 (-1.3 to -0.8)*	-0.2 (-0.2 to -0.2)*	-0.7 (-0.9 to -0.6)*
		RII	1.3 (1.2 to 1.4)*	1.0 (1.0 to 1.0)*	1.0 (1.0 to 1.0)*	-1.2 (-1.6 to -0.9)*	-0.2 (-0.2 to -0.2)*	-0.9 (-1.1 to -0.7)*
	Lesotho	SII	6.8 (3.6 to 12.5)*	2.5 (1.1 to 4.4)*	1.4 (0.4 to 3.9)*	-0.2 (-0.5 to 0.0)*	-0.1 (-0.1 to 0.0)*	-0.2 (-0.4 to 0.0)*
		RII	1.1 (1.0 to 1.1)*	1.0 (1.0 to 1.0)*	1.0 (1.0 to 1.0)*	-0.2 (-0.6 to 0.0)*	-0.1 (-0.2 to 0.0)*	-0.2 (-0.4 to 0.0)*
	Liberia	SII	39.3 (32.7 to 45.3)*	8.0 (6.6 to 9.9)*	2.7 (1.8 to 4.2)*	-1.6 (-1.9 to -1.2)*	-0.5 (-0.6 to -0.5)*	-1.2 (-1.4 to -1.0)*
		RII	1.8 (1.6 to 2.1)*	1.1 (1.1 to 1.1)*	1.0 (1.0 to 1.0)*	-2.5 (-3.3 to -1.8)*	-0.6 (-0.7 to -0.5)*	-1.9 (-2.4 to -1.4)*
	Madagascar	SII	51.2 (44.6 to 56.4)*	8.8 (5.5 to 14.3)*	2.6 (1.1 to 6.6)*	-2.1 (-2.5 to -1.5)*	-0.6 (-0.8 to -0.4)*	-1.6 (-1.8 to -1.3)*
		RII	2.2 (1.9 to 2.6)*	1.1 (1.1 to 1.2)*	1.0 (1.0 to 1.1)*	-3.5 (-4.4 to -2.5)*	-0.7 (-0.9 to -0.5)*	-2.6 (-3.1 to -1.9)*
	Malawi	SII	6.8 (5.2 to 8.7)*	1.4 (1.0 to 1.9)*	0.6 (0.4 to 1.0)*	-0.3 (-0.4 to -0.2)*	-0.1 (-0.1 to -0.1)*	-0.2 (-0.3 to -0.2)*
		RII	1.1 (1.1 to 1.1)*	1.0 (1.0 to 1.0)*	1.0 (1.0 to 1.0)*	-0.3 (-0.4 to -0.2)*	-0.1 (-0.1 to -0.1)*	-0.2 (-0.3 to -0.2)*
	Mali	SII	30.3 (27.4 to 33.9)*	17.5 (14.8 to 20.2)*	12.2 (9.6 to 15.0)*	-0.6 (-0.8 to -0.5)*	-0.5 (-0.6 to -0.4)*	-0.6 (-0.7 to -0.5)*
		RII	1.5 (1.5 to 1.6)*	1.2 (1.2 to 1.3)*	1.1 (1.1 to 1.2)*	-1.1 (-1.4 to -0.8)*	-0.7 (-0.8 to -0.6)*	-1.0 (-1.2 to -0.7)*
	Morocco	SII	3.5 (1.4 to 6.7)*	0.6 (0.1 to 5.0)*	0.2 (0.0 to 5.3)*	-0.1 (-0.3 to 0.1)	0.0 (-0.1 to 0.1)	-0.1 (-0.2 to 0.1)
		RII	1.0 (1.0 to 1.1)*	1.0 (1.0 to 1.1)*	1.0 (1.0 to 1.1)*	-0.1 (-0.3 to 0.1)	0.0 (-0.1 to 0.1)	-0.1 (-0.2 to 0.1)
	Mozambique	SII	20.3 (16.8 to 23.9)*	12.0 (8.6 to 16.9)*	9.1 (5.0 to 15.4)*	-0.4 (-0.7 to -0.1)*	-0.3 (-0.4 to -0.1)*	-0.4 (-0.6 to -0.1)*
		RII	1.3 (1.2 to 1.3)*	1.1 (1.1 to 1.2)*	1.1 (1.1 to 1.2)*	-0.5 (-0.9 to -0.2)*	-0.3 (-0.4 to -0.2)*	-0.4 (-0.7 to -0.2)*
	Namibia	SII	7.0 (3.9 to 10.4)*	3.3 (1.8 to 5.4)*	2.2 (1.0 to 4.6)*	-0.2 (-0.4 to 0.0)*	-0.1 (-0.2 to 0.0)*	-0.1 (-0.3 to 0.0)*
		RII	1.1 (1.0 to 1.1)*	1.0 (1.0 to 1.1)*	1.0 (1.0 to 1.0)*	-0.2 (-0.4 to 0.0)*	-0.1 (-0.2 to 0.0)*	-0.2 (-0.3 to 0.0)*
	Niger	SII	35.3 (28.5 to 40.4)*	6.3 (4.6 to 9.0)*	1.3 (0.7 to 2.4)*	-1.4 (-1.7 to -1.2)*	-0.5 (-0.7 to -0.4)*	-1.1 (-1.3 to -0.9)*
		RII	2.3 (1.9 to 2.7)*	1.1 (1.0 to 1.1)*	1.0 (1.0 to 1.0)*	-3.7 (-4.6 to -2.9)*	-0.5 (-0.7 to -0.4)*	-2.7 (-3.2 to -2.1)*
	Nigeria	SII	70.1 (67.8 to 72.3)*	61.5 (59.4 to 62.9)*	46.7 (42.9 to 50.1)*	-0.4 (-0.6 to -0.3)*	-1.5 (-1.7 to -1.2)*	-0.8 (-0.9 to -0.6)*
		RII	7.6 (6.8 to 8.5)*	2.8 (2.7 to 3.0)*	1.9 (1.8 to 2.1)*	-4.8 (-5.4 to -4.2)*	-3.8 (-4.2 to -3.5)*	-4.4 (-5.0 to -3.9)*
	Rwanda	SII	3.3 (2.0 to 4.8)*	1.2 (0.7 to 2.3)*	0.8 (0.3 to 1.8)*	-0.1 (-0.2 to 0.0)*	0.0 (-0.1 to 0.0)*	-0.1 (-0.1 to 0.0)*
		RII	1.0 (1.0 to 1.1)*	1.0 (1.0 to 1.0)*	1.0 (1.0 to 1.0)*	-0.1 (-0.2 to 0.0)*	-0.1 (-0.1 to 0.0)*	-0.1 (-0.1 to 0.0)*
	Sao Tome and Principe	SII	6.1 (-0.4 to 16.4)	1.0 (0.0 to 6.1)	0.4 (0.0 to 5.5)*	-0.2 (-0.8 to 0.1)	0.0 (-0.1 to 0.0)	-0.2 (-0.5 to 0.1)
		RII	1.1 (1.0 to 1.2)	1.0 (1.0 to 1.1)	1.0 (1.0 to 1.1)*	-0.3 (-0.9 to 0.1)	-0.1 (-0.2 to 0.0)	-0.2 (-0.6 to 0.1)
	Senegal	SII	11.3 (9.2 to 14.4)*	5.9 (4.9 to 6.9)*	4.1 (2.9 to 5.4)*	-0.3 (-0.5 to -0.1)*	-0.2 (-0.2 to -0.1)*	-0.2 (-0.4 to -0.1)*
		RII	1.1 (1.1 to 1.2)*	1.1 (1.1 to 1.1)*	1.0 (1.0 to 1.1)*	-0.3 (-0.5 to -0.2)*	-0.2 (-0.2 to -0.1)*	-0.3 (-0.4 to -0.2)*

Indicators	Country	Index	Estimates of Inequalities			Changes in Inequalities		
			**Year 2000**	**Year 2020**	**Year 2030**	**2000–2020**	**2020–2030**	**2000–2030**
	Sierra Leone	SII	15.7 (9.5 to 23.6)*	1.3 (0.9 to 2.1)*	0.3 (0.1 to 0.5)*	-0.7 (-1.1 to -0.4)*	-0.1 (-0.2 to -0.1)*	-0.5 (-0.8 to -0.3)*
		RII	1.3 (1.2 to 1.5)*	1.0 (1.0 to 1.0)*	1.0 (1.0 to 1.0)*	-1.2 (-1.9 to -0.6)*	-0.1 (-0.2 to -0.1)*	-0.8 (-1.3 to -0.5)*
	South Africa	SII	9.0 (-3.0 to 24.5)	3.5 (-1.5 to 7.9)	1.7 (-0.5 to 8.2)	-0.2 (-1.0 to 0.2)	-0.1 (-0.3 to 0.2)	-0.2 (-0.8 to 0.2)
		RII	1.1 (1.0 to 1.5)	1.0 (1.0 to 1.1)	1.0 (1.0 to 1.1)	-0.3 (-2.0 to 0.2)	-0.1 (-0.4 to 0.2)	-0.3 (-1.4 to 0.3)
	Tanzania	SII	10.6 (7.9 to 14.6)*	2.8 (1.8 to 4.0)*	1.4 (0.7 to 2.3)*	-0.4 (-0.6 to -0.2)*	-0.1 (-0.2 to -0.1)*	-0.3 (-0.4 to -0.2)*
		RII	1.1 (1.1 to 1.2)*	1.0 (1.0 to 1.0)*	1.0 (1.0 to 1.0)*	-0.4 (-0.7 to -0.3)*	-0.1 (-0.2 to -0.1)*	-0.3 (-0.5 to -0.2)*
	Togo	SII	16.8 (6.7 to 33.8)*	3.5 (1.1 to 7.1)*	1.4 (0.1 to 6.3)*	-0.7 (-1.6 to 0.0)	-0.2 (-0.3 to 0.0)	-0.5 (-1.1 to 0.0)
		RII	1.2 (1.1 to 1.7)*	1.0 (1.0 to 1.1)*	1.0 (1.0 to 1.1)*	-0.8 (-2.4 to 0.0)	-0.2 (-0.3 to 0.0)	-0.6 (-1.7 to 0.0)
	Uganda	SII	4.0 (0.8 to 8.2)*	0.6 (0.0 to 1.2)*	0.2 (0.0 to 0.4)	-0.2 (-0.4 to 0.0)*	0.0 (-0.1 to 0.0)*	-0.1 (-0.3 to 0.0)*
		RII	1.1 (1.0 to 1.1)*	1.0 (1.0 to 1.0)*	1.0 (1.0 to 1.0)	-0.2 (-0.5 to 0.0)*	0.0 (-0.1 to 0.0)*	-0.2 (-0.3 to 0.0)*
	Zambia	SII	6.8 (4.9 to 9.0)*	3.6 (2.6 to 5.0)*	2.6 (1.6 to 4.0)*	-0.2 (-0.3 to -0.1)*	-0.1 (-0.1 to -0.1)*	-0.1 (-0.2 to -0.1)*
		RII	1.1 (1.1 to 1.1)*	1.0 (1.0 to 1.1)*	1.0 (1.0 to 1.0)*	-0.2 (-0.3 to -0.1)*	-0.1 (-0.1 to -0.1)*	-0.2 (-0.2 to -0.1)*
	Zimbabwe	SII	12.5 (6.3 to 18.9)*	3.1 (1.7 to 5.1)*	1.2 (0.5 to 2.3)*	-0.5 (-0.8 to -0.2)*	-0.2 (-0.3 to -0.1)*	-0.4 (-0.6 to -0.2)*
		RII	1.2 (1.1 to 1.3)*	1.0 (1.0 to 1.1)*	1.0 (1.0 to 1.0)*	-0.7 (-1.2 to -0.4)*	-0.2 (-0.3 to -0.1)*	-0.5 (-0.9 to -0.3)*
MCV1 immunization	Angola	SII	51.5 (22.0 to 62.9)*	54.9 (48.3 to 58.9)*	50.5 (32.9 to 56.5)*	0.2 (-0.5 to 1.6)	-0.3 (-2.1 to -0.1)*	0.0 (-0.7 to 0.5)
		RII	2.3 (1.3 to 5.0)*	3.0 (2.2 to 4.1)*	3.3 (1.6 to 7.1)*	1.3 (-4.0 to 5.8)	1.2 (-3.0 to 5.8)	1.3 (-3.7 to 5.6)
	Benin	SII	32.1 (28.5 to 35.0)*	24.4 (21.8 to 27.2)*	20.7 (17.7 to 24.6)*	-0.4 (-0.5 to -0.2)*	-0.4 (-0.5 to -0.2)*	-0.4 (-0.5 to -0.2)*
		RII	1.7 (1.6 to 1.8)*	1.4 (1.4 to 1.5)*	1.3 (1.3 to 1.4)*	-0.7 (-1.1 to -0.4)*	-0.6 (-0.9 to -0.4)*	-0.7 (-1.0 to -0.4)*
	Burkina Faso	SII	27.2 (21.7 to 32.3)*	1.0 (0.7 to 1.4)*	0.1 (0.0 to 0.1)*	-1.3 (-1.6 to -1.0)*	-0.1 (-0.1 to -0.1)*	-0.9 (-1.1 to -0.7)*
		RII	1.9 (1.7 to 2.2)*	1.0 (1.0 to 1.0)*	1.0 (1.0 to 1.0)*	-3.2 (-3.8 to -2.5)*	-0.1 (-0.1 to -0.1)*	-2.1 (-2.6 to -1.7)*
	Burundi	SII	4.5 (2.2 to 7.7)*	2.7 (0.7 to 4.8)*	1.9 (0.1 to 5.1)*	-0.1 (-0.2 to 0.1)	-0.1 (-0.1 to 0.0)	-0.1 (-0.2 to 0.1)
		RII	1.0 (1.0 to 1.1)*	1.0 (1.0 to 1.1)*	1.0 (1.0 to 1.1)*	-0.1 (-0.3 to 0.1)	-0.1 (-0.1 to 0.1)	-0.1 (-0.2 to 0.1)
	Cameroon	SII	34.9 (30.6 to 38.2)*	31.2 (27.3 to 34.7)*	29.0 (24.4 to 33.4)*	-0.2 (-0.3 to 0.0)*	-0.2 (-0.4 to -0.1)*	-0.2 (-0.4 to 0.0)*
		RII	1.7 (1.5 to 1.8)*	1.6 (1.5 to 1.7)*	1.6 (1.4 to 1.7)*	-0.2 (-0.6 to 0.1)	-0.3 (-0.6 to 0.1)	-0.2 (-0.6 to 0.1)
	Chad	SII	12.2 (9.3 to 15.7)*	10.4 (7.1 to 14.2)*	2.9 (1.4 to 5.1)*	-0.1 (-0.2 to 0.0)	-0.8 (-1.0 to -0.6)*	-0.3 (-0.4 to -0.2)*
		RII	2.1 (1.8 to 2.4)*	1.1 (1.1 to 1.2)*	1.0 (1.0 to 1.1)*	-2.9 (-3.5 to -2.2)*	-1.1 (-1.4 to -0.8)*	-2.3 (-2.8 to -1.8)*
	Comoros	SII	29.2 (14.2 to 41.2)*	15.2 (8.1 to 27.9)*	9.6 (2.2 to 27.2)*	-0.7 (-1.4 to 0.4)	-0.5 (-0.9 to 0.0)	-0.7 (-1.2 to 0.4)
		RII	1.6 (1.2 to 2.5)*	1.2 (1.1 to 1.5)*	1.1 (1.0 to 1.6)*	-1.4 (-3.8 to 0.9)	-0.7 (-1.2 to 0.9)	-1.2 (-2.9 to 0.9)
	Congo Brazzaville	SII	42.2 (36.2 to 47.7)*	19.9 (16.2 to 23.6)*	11.1 (7.0 to 16.3)*	-1.1 (-1.5 to -0.8)*	-0.9 (-1.0 to -0.7)*	-1.0 (-1.3 to -0.7)*
		RII	2.0 (1.8 to 2.4)*	1.3 (1.2 to 1.3)*	1.1 (1.1 to 1.2)*	-2.3 (-3.2 to -1.6)*	-1.1 (-1.3 to -0.9)*	-1.9 (-2.5 to -1.4)*
	Congo Democratic Republic	SII	33.1 (28.6 to 37.2)*	17.0 (13.8 to 20.4)*	9.8 (6.4 to 14.1)*	-0.8 (-1.0 to -0.6)*	-0.7 (-0.8 to -0.6)*	-0.8 (-0.9 to -0.6)*
		RII	1.8 (1.6 to 2.0)*	1.2 (1.2 to 1.3)*	1.1 (1.1 to 1.2)*	-1.9 (-2.6 to -1.3)*	-1.0 (-1.1 to -0.8)*	-1.6 (-2.1 to -1.2)*
	Cote d'Ivoire	SII	32.2 (23.0 to 39.1)*	23.2 (13.3 to 31.6)*	17.9 (5.1 to 29.5)*	-0.5 (-0.9 to 0.2)	-0.5 (-1.0 to -0.1)*	-0.5 (-0.9 to 0.0)
		RII	1.8 (1.4 to 2.5)*	1.4 (1.2 to 1.8)*	1.3 (1.1 to 1.9)*	-1.1 (-3.6 to 1.1)	-0.8 (-1.4 to 0.7)	-1.0 (-2.8 to 0.9)
	Egypt	SII	1.3 (0.9 to 1.8)*	6.8 (3.1 to 10.7)*	7.3 (0.8 to 14.1)*	0.3 (0.1 to 0.5)*	0.0 (-0.3 to 0.4)	0.2 (0.0 to 0.4)
		RII	1.0 (1.0 to 1.0)*	1.1 (1.0 to 1.1)*	1.2 (1.0 to 1.3)*	0.4 (0.1 to 0.6)*	0.5 (-0.3 to 1.3)	0.4 (0.0 to 0.8)*
	Eswatini	SII	10.7 (3.4 to 19.7)*	2.4 (0.4 to 14.5)*	0.9 (0.0 to 14.4)*	-0.4 (-0.9 to 0.2)	-0.1 (-0.2 to 0.1)	-0.3 (-0.6 to 0.2)
		RII	1.1 (1.0 to 1.3)*	1.0 (1.0 to 1.2)*	1.0 (1.0 to 1.2)*	-0.4 (-1.1 to 0.3)	-0.1 (-0.2 to 0.3)	-0.3 (-0.8 to 0.3)
	Ethiopia	SII	31.5 (28.7 to 34.3)*	26.6 (23.6 to 29.3)*	17.1 (14.2 to 19.8)*	-0.3 (-0.4 to -0.1)*	-1.0 (-1.1 to -0.8)*	-0.5 (-0.6 to -0.4)*
		RII	2.8 (2.5 to 3.0)*	1.5 (1.4 to 1.6)*	1.2 (1.2 to 1.3)*	-3.0 (-3.5 to -2.4)*	-2.0 (-2.1 to -1.7)*	-2.7 (-3.0 to -2.2)*
	Gabon	SII	25.2 (19.2 to 30.4)*	8.4 (5.5 to 11.1)*	3.4 (1.7 to 5.3)*	-0.8 (-1.0 to -0.6)*	-0.5 (-0.6 to -0.4)*	-0.7 (-0.9 to -0.5)*
		RII	1.6 (1.4 to 1.7)*	1.1 (1.1 to 1.1)*	1.0 (1.0 to 1.1)*	-1.8 (-2.2 to -1.3)*	-0.6 (-0.7 to -0.4)*	-1.4 (-1.7 to -1.0)*
	Gambia	SII	0.0 (-2.0 to 1.6)	-2.6 (-6.1 to 0.2)	-4.9 (-10.7 to -0.8)*	-0.1 (-0.2 to 0.0)*	-0.2 (-0.5 to -0.1)*	-0.2 (-0.3 to -0.1)*
		RII	1.0 (1.0 to 1.0)	1.0 (0.9 to 1.0)	0.9 (0.9 to 1.0)*	-0.1 (-0.3 to 0.0)*	-0.3 (-0.7 to -0.1)*	-0.2 (-0.4 to -0.1)*
	Ghana	SII	14.1 (10.1 to 18.6)*	4.6 (2.7 to 6.9)*	2.3 (1.1 to 4.3)*	-0.5 (-0.7 to -0.3)*	-0.2 (-0.3 to -0.2)*	-0.4 (-0.5 to -0.3)*
		RII	1.2 (1.1 to 1.3)*	1.1 (1.0 to 1.1)*	1.0 (1.0 to 1.0)*	-0.6 (-0.9 to -0.4)*	-0.2 (-0.3 to -0.2)*	-0.5 (-0.7 to -0.3)*
	Guinea	SII	30.2 (25.9 to 34.8)*	26.6 (21.7 to 30.4)*	21.2 (15.7 to 25.8)*	-0.2 (-0.4 to 0.0)*	-0.5 (-0.7 to -0.4)*	-0.3 (-0.5 to -0.2)*
		RII	1.6 (1.5 to 1.8)*	1.9 (1.7 to 2.0)*	1.9 (1.6 to 2.1)*	0.7 (0.2 to 1.1)*	0.2 (-0.4 to 0.8)	0.5 (0.0 to 1.0)*
	Kenya	SII	35.1 (30.6 to 39.8)*	11.3 (9.5 to 13.3)*	5.3 (3.9 to 7.3)*	-1.2 (-1.4 to -1.0)*	-0.6 (-0.7 to -0.5)*	-1.0 (-1.2 to -0.8)*
		RII	1.6 (1.5 to 1.8)*	1.1 (1.1 to 1.2)*	1.1 (1.0 to 1.1)*	-1.9 (-2.3 to -1.5)*	-0.7 (-0.8 to -0.6)*	-1.5 (-1.8 to -1.2)*
	Lesotho	SII	15.7 (11.2 to 22.0)*	7.0 (4.0 to 10.7)*	4.3 (1.7 to 8.3)*	-0.4 (-0.7 to -0.2)*	-0.3 (-0.4 to -0.2)*	-0.4 (-0.6 to -0.2)*
		RII	1.2 (1.1 to 1.3)*	1.1 (1.0 to 1.1)*	1.0 (1.0 to 1.1)*	-0.6 (-1.0 to -0.3)*	-0.3 (-0.4 to -0.2)*	-0.5 (-0.8 to -0.3)*
	Liberia	SII	33.7 (28.5 to 39.9)*	19.9 (16.0 to 23.8)*	13.5 (9.9 to 17.4)*	-0.7 (-0.9 to -0.5)*	-0.6 (-0.8 to -0.5)*	-0.7 (-0.9 to -0.5)*
		RII	1.8 (1.6 to 2.0)*	1.3 (1.2 to 1.4)*	1.2 (1.1 to 1.2)*	-1.5 (-2.1 to -1.0)*	-0.9 (-1.2 to -0.7)*	-1.3 (-1.8 to -0.9)*
	Madagascar	SII	50.9 (46.8 to 54.5)*	18.6 (13.0 to 25.5)*	8.0 (3.9 to 14.8)*	-1.6 (-2.0 to -1.2)*	-1.1 (-1.2 to -0.9)*	-1.4 (-1.6 to -1.2)*
		RII	2.6 (2.3 to 2.9)*	1.2 (1.2 to 1.4)*	1.1 (1.0 to 1.2)*	-3.7 (-4.5 to -2.8)*	-1.3 (-1.5 to -1.0)*	-2.9 (-3.4 to -2.3)*
	Malawi	SII	14.2 (12.0 to 17.0)*	3.7 (2.8 to 4.8)*	1.7 (1.0 to 2.5)*	-0.5 (-0.6 to -0.4)*	-0.2 (-0.2 to -0.2)*	-0.4 (-0.5 to -0.3)*
		RII	1.2 (1.2 to 1.2)*	1.0 (1.0 to 1.1)*	1.0 (1.0 to 1.0)*	-0.7 (-0.8 to -0.5)*	-0.2 (-0.3 to -0.2)*	-0.5 (-0.6 to -0.4)*
	Mali	SII	26.7 (23.7 to 29.9)*	14.6 (11.9 to 17.5)*	9.1 (6.5 to 12.6)*	-0.6 (-0.8 to -0.5)*	-0.6 (-0.6 to -0.5)*	-0.6 (-0.7 to -0.5)*
		RII	1.6 (1.5 to 1.7)*	1.2 (1.2 to 1.3)*	1.1 (1.1 to 1.2)*	-1.4 (-1.7 to -1.2)*	-0.8 (-0.9 to -0.7)*	-1.2 (-1.5 to -1.0)*
	Morocco	SII	17.1 (11.0 to 24.8)*	5.2 (0.8 to 23.0)*	2.7 (0.1 to 26.6)*	-0.6 (-1.2 to 0.6)	-0.2 (-0.3 to 0.4)	-0.5 (-0.8 to 0.5)
		RII	1.2 (1.1 to 1.3)*	1.1 (1.0 to 1.3)*	1.0 (1.0 to 1.5)*	-0.7 (-1.4 to 0.8)	-0.2 (-0.3 to 1.2)	-0.6 (-1.0 to 0.9)
	Mozambique	SII	33.0 (28.8 to 37.2)*	21.3 (17.2 to 26.6)*	16.5 (11.0 to 24.3)*	-0.6 (-0.9 to -0.2)*	-0.5 (-0.6 to -0.2)*	-0.5 (-0.8 to -0.2)*
		RII	1.5 (1.4 to 1.7)*	1.3 (1.2 to 1.4)*	1.2 (1.1 to 1.3)*	-0.9 (-1.4 to -0.3)*	-0.6 (-0.8 to -0.3)*	-0.8 (-1.2 to -0.3)*
	Namibia	SII	13.0 (8.3 to 18.1)*	4.1 (2.0 to 6.6)*	1.9 (0.6 to 3.8)*	-0.4 (-0.6 to -0.3)*	-0.2 (-0.3 to -0.1)*	-0.4 (-0.5 to -0.2)*
		RII	1.2 (1.1 to 1.3)*	1.0 (1.0 to 1.1)*	1.0 (1.0 to 1.0)*	-0.6 (-0.8 to -0.3)*	-0.2 (-0.3 to -0.1)*	-0.5 (-0.7 to -0.3)*
	Niger	SII	25.9 (22.0 to 31.2)*	10.9 (8.3 to 14.3)*	3.0 (1.9 to 5.0)*	-0.8 (-0.9 to -0.6)*	-0.8 (-0.9 to -0.6)*	-0.8 (-0.9 to -0.7)*
		RII	2.3 (2.0 to 2.6)*	1.1 (1.1 to 1.2)*	1.0 (1.0 to 1.1)*	-3.4 (-4.0 to -2.7)*	-0.9 (-1.2 to -0.7)*	-2.6 (-3.0 to -2.1)*
	Nigeria	SII	56.6 (54.1 to 58.9)*	55.6 (53.5 to 57.3)*	47.9 (44.6 to 51.2)*	0.0 (-0.2 to 0.1)	-0.8 (-1.0 to -0.6)*	-0.3 (-0.4 to -0.2)*
		RII	6.3 (5.7 to 7.0)*	3.0 (2.8 to 3.2)*	2.2 (2.0 to 2.4)*	-3.7 (-4.2 to -3.1)*	-3.2 (-3.5 to -2.8)*	-3.5 (-4.0 to -3.0)*

Indicators	Country	Index	Estimates of Inequalities			Changes in Inequalities		
			Year 2000	Year 2020	Year 2030	2000–2020	2020–2030	2000–2030
	Rwanda	SII	5.1 (2.4 to 8.1)*	0.3 (-1.7 to 1.7)	-0.8 (-2.6 to 0.5)	-0.2 (-0.3 to -0.1)*	-0.1 (-0.2 to -0.1)*	-0.2 (-0.3 to -0.1)*
		RII	1.1 (1.0 to 1.1)*	1.0 (1.0 to 1.0)	1.0 (1.0 to 1.0)	-0.3 (-0.4 to -0.2)*	-0.1 (-0.2 to -0.1)*	-0.2 (-0.4 to -0.1)*
	Sao Tome and Principe	SII	16.7 (5.4 to 30.4)*	5.0 (0.8 to 21.3)*	2.4 (-0.1 to 23.1)	-0.5 (-1.3 to 0.3)	-0.2 (-0.4 to 0.1)	-0.4 (-0.9 to 0.3)
		RII	1.2 (1.1 to 1.6)*	1.1 (1.0 to 1.3)*	1.0 (1.0 to 1.4)	-0.7 (-2.0 to 0.7)	-0.2 (-0.5 to 0.6)	-0.6 (-1.4 to 0.6)
	Senegal	SII	22.8 (19.2 to 25.9)*	9.5 (8.1 to 11.5)*	5.4 (4.0 to 7.4)*	-0.7 (-0.8 to -0.5)*	-0.4 (-0.5 to -0.3)*	-0.6 (-0.7 to -0.5)*
		RII	1.4 (1.3 to 1.5)*	1.1 (1.1 to 1.1)*	1.1 (1.0 to 1.1)*	-1.1 (-1.3 to -0.8)*	-0.5 (-0.6 to -0.4)*	-0.9 (-1.0 to -0.7)*
	Sierra Leone	SII	8.8 (4.3 to 13.1)*	1.5 (-1.0 to 4.6)	-0.7 (-3.3 to 2.0)	-0.4 (-0.5 to -0.2)*	-0.2 (-0.3 to -0.1)*	-0.3 (-0.5 to -0.2)*
		RII	1.2 (1.1 to 1.2)*	1.0 (1.0 to 1.1)	1.0 (1.0 to 1.0)	-0.6 (-1.0 to -0.3)*	-0.3 (-0.4 to -0.1)*	-0.5 (-0.8 to -0.2)*
	South Africa	SII	13.7 (3.1 to 27.2)*	5.1 (-0.5 to 10.6)	2.1 (-2.2 to 9.3)	-0.4 (-1.0 to 0.1)	-0.2 (-0.5 to 0.0)	-0.4 (-0.8 to 0.1)
		RII	1.2 (1.0 to 1.8)*	1.1 (1.0 to 1.1)	1.0 (1.0 to 1.1)	-0.6 (-2.6 to 0.1)	-0.3 (-0.6 to 0.1)	-0.5 (-1.9 to 0.1)
	Tanzania	SII	26.1 (22.2 to 29.9)*	14.2 (11.9 to 17.7)*	9.9 (7.0 to 14.2)*	-0.6 (-0.8 to -0.4)*	-0.4 (-0.5 to -0.3)*	-0.5 (-0.7 to -0.3)*
		RII	1.4 (1.3 to 1.5)*	1.2 (1.1 to 1.2)*	1.1 (1.1 to 1.2)*	-0.8 (-1.2 to -0.5)*	-0.5 (-0.6 to -0.4)*	-0.7 (-1.0 to -0.5)*
	Togo	SII	17.8 (9.4 to 26.3)*	7.3 (2.7 to 13.6)*	3.1 (0.2 to 11.1)*	-0.5 (-0.9 to -0.1)*	-0.4 (-0.6 to -0.2)*	-0.5 (-0.7 to -0.1)*
		RII	1.4 (1.1 to 1.9)*	1.1 (1.0 to 1.2)*	1.0 (1.0 to 1.2)*	-1.1 (-2.8 to -0.1)*	-0.5 (-0.7 to -0.2)*	-0.9 (-2.1 to -0.1)*
	Uganda	SII	15.3 (11.8 to 18.7)*	4.7 (2.5 to 6.5)*	1.7 (0.1 to 3.3)*	-0.5 (-0.7 to -0.4)*	-0.3 (-0.4 to -0.2)*	-0.5 (-0.6 to -0.3)*
		RII	1.3 (1.2 to 1.4)*	1.1 (1.0 to 1.1)*	1.0 (1.0 to 1.0)*	-1.0 (-1.3 to -0.7)*	-0.4 (-0.5 to -0.3)*	-0.8 (-1.0 to -0.6)*
	Zambia	SII	12.9 (10.3 to 16.0)*	6.8 (5.0 to 9.2)*	4.6 (2.8 to 6.8)*	-0.3 (-0.4 to -0.2)*	-0.2 (-0.3 to -0.2)*	-0.3 (-0.4 to -0.2)*
		RII	1.2 (1.1 to 1.2)*	1.1 (1.1 to 1.1)*	1.1 (1.0 to 1.1)*	-0.4 (-0.5 to -0.2)*	-0.3 (-0.3 to -0.2)*	-0.4 (-0.5 to -0.2)*
	Zimbabwe	SII	22.8 (16.8 to 27.6)*	7.2 (4.7 to 9.8)*	3.0 (1.4 to 5.2)*	-0.8 (-1.0 to -0.5)*	-0.4 (-0.5 to -0.3)*	-0.7 (-0.8 to -0.5)*
		RII	1.4 (1.3 to 1.6)*	1.1 (1.1 to 1.1)*	1.0 (1.0 to 1.1)*	-1.4 (-1.9 to -1.0)*	-0.5 (-0.6 to -0.4)*	-1.1 (-1.5 to -0.8)*
Polio3 immunization	Angola	SII	47.5 (22.9 to 55.0)*	45.5 (39.9 to 50.2)*	39.8 (21.7 to 47.2)*	-0.1 (-0.4 to 1.1)	-0.5 (-2.1 to -0.2)*	-0.2 (-0.5 to 0.0)
		RII	2.5 (1.3 to 4.9)*	2.6 (2.1 to 3.6)*	2.7 (1.5 to 5.0)*	0.2 (-4.2 to 5.0)	0.1 (-3.3 to 3.6)	0.2 (-3.9 to 4.5)
	Benin	SII	27.8 (24.9 to 30.5)*	25.4 (22.3 to 28.1)*	23.7 (19.8 to 28.0)*	-0.1 (-0.3 to 0.0)	-0.2 (-0.3 to 0.0)*	-0.1 (-0.3 to 0.0)
		RII	1.4 (1.4 to 1.5)*	1.4 (1.3 to 1.5)*	1.4 (1.3 to 1.5)*	-0.1 (-0.4 to 0.2)	-0.2 (-0.4 to 0.1)	-0.1 (-0.4 to 0.1)
	Burkina Faso	SII	34.4 (28.7 to 40.2)*	0.3 (0.2 to 0.5)*	0.0 (0.0 to 0.0)*	-1.7 (-2.0 to -1.4)*	0.0 (0.0 to 0.0)*	-1.1 (-1.3 to -1.0)*
		RII	2.0 (1.8 to 2.3)*	1.0 (1.0 to 1.0)*	1.0 (1.0 to 1.0)*	-3.5 (-4.1 to -2.8)*	0.0 (0.0 to 0.0)*	-2.3 (-2.8 to -1.9)*
	Burundi	SII	8.0 (4.0 to 16.0)*	2.1 (1.1 to 3.4)*	1.0 (0.3 to 2.4)*	-0.3 (-0.7 to -0.1)*	-0.1 (-0.2 to -0.1)*	-0.2 (-0.5 to -0.1)*
		RII	1.1 (1.0 to 1.2)*	1.0 (1.0 to 1.0)*	1.0 (1.0 to 1.0)*	-0.3 (-0.8 to -0.1)*	-0.1 (-0.2 to -0.1)*	-0.3 (-0.6 to -0.1)*
	Cameroon	SII	24.4 (21.0 to 27.9)*	22.4 (19.0 to 26.0)*	20.9 (16.7 to 25.8)*	-0.1 (-0.3 to 0.1)	-0.1 (-0.3 to 0.0)	-0.1 (-0.3 to 0.1)
		RII	1.4 (1.3 to 1.4)*	1.3 (1.3 to 1.4)*	1.3 (1.2 to 1.4)*	-0.1 (-0.4 to 0.2)	-0.1 (-0.4 to 0.2)	-0.1 (-0.4 to 0.2)
	Chad	SII	26.7 (22.0 to 31.0)*	18.4 (14.1 to 23.2)*	12.0 (7.8 to 17.5)*	-0.4 (-0.6 to -0.2)*	-0.6 (-0.8 to -0.5)*	-0.5 (-0.6 to -0.3)*
		RII	2.0 (1.7 to 2.3)*	1.3 (1.2 to 1.4)*	1.2 (1.1 to 1.3)*	-2.0 (-2.6 to -1.4)*	-1.3 (-1.5 to -1.0)*	-1.8 (-2.2 to -1.3)*
	Comoros	SII	27.3 (12.6 to 40.3)*	13.0 (5.8 to 24.8)*	7.8 (1.1 to 25.1)*	-0.7 (-1.5 to 0.4)	-0.5 (-0.8 to 0.1)	-0.6 (-1.2 to 0.3)
		RII	1.5 (1.2 to 2.6)*	1.2 (1.1 to 1.4)*	1.1 (1.0 to 1.5)*	-1.2 (-4.1 to 0.7)	-0.6 (-0.9 to 0.7)	-1.0 (-3.0 to 0.7)
	Congo Brazzaville	SII	15.4 (10.9 to 19.9)*	19.9 (13.7 to 25.2)*	18.5 (9.9 to 26.3)*	0.2 (0.0 to 0.5)	-0.1 (-0.4 to 0.1)	0.1 (-0.1 to 0.3)
		RII	1.2 (1.1 to 1.3)*	1.3 (1.2 to 1.5)*	1.4 (1.2 to 1.6)*	0.5 (0.1 to 1.1)*	0.4 (-0.2 to 1.1)	0.5 (0.0 to 1.1)*
	Congo Democratic Republic	SII	35.8 (32.0 to 40.2)*	11.8 (9.5 to 14.1)*	3.3 (2.1 to 4.8)*	-1.2 (-1.4 to -1.1)*	-0.8 (-1.0 to -0.7)*	-1.1 (-1.2 to -1.0)*
		RII	2.6 (2.3 to 3.1)*	1.1 (1.1 to 1.2)*	1.0 (1.0 to 1.1)*	-4.0 (-4.9 to -3.4)*	-1.0 (-1.1 to -0.8)*	-3.0 (-3.6 to -2.6)*
	Cote d'Ivoire	SII	25.9 (13.8 to 37.0)*	10.6 (4.3 to 21.4)*	5.8 (0.8 to 21.2)*	-0.8 (-1.4 to 0.3)	-0.4 (-0.7 to 0.0)*	-0.7 (-1.1 to 0.2)
		RII	1.5 (1.2 to 2.2)*	1.1 (1.0 to 1.4)*	1.1 (1.0 to 1.4)*	-1.3 (-3.4 to 0.6)	-0.5 (-0.8 to 0.5)	-1.1 (-2.5 to 0.5)
	Egypt	SII	2.1 (0.9 to 3.4)*	0.2 (-0.1 to 0.7)	0.0 (-0.2 to 0.3)	-0.1 (-0.1 to 0.0)*	0.0 (0.0 to 0.0)*	-0.1 (-0.1 to 0.0)*
		RII	1.0 (1.0 to 1.0)*	1.0 (1.0 to 1.0)	1.0 (1.0 to 1.0)	-0.1 (-0.1 to 0.0)*	0.0 (0.0 to 0.0)*	-0.1 (-0.1 to 0.0)*
	Eswatini	SII	3.9 (-2.4 to 12.7)	0.1 (-1.9 to 4.2)	0.0 (-3.2 to 3.7)	-0.2 (-0.6 to 0.1)	0.0 (-0.2 to 0.0)	-0.1 (-0.4 to 0.1)
		RII	1.0 (1.0 to 1.2)	1.0 (1.0 to 1.0)	1.0 (1.0 to 1.0)	-0.2 (-0.7 to 0.1)	0.0 (-0.2 to 0.0)	-0.1 (-0.5 to 0.1)
	Ethiopia	SII	32.3 (29.1 to 34.9)*	22.9 (19.8 to 25.4)*	14.9 (11.9 to 18.1)*	-0.5 (-0.6 to -0.3)*	-0.8 (-0.9 to -0.7)*	-0.6 (-0.7 to -0.4)*
		RII	2.3 (2.1 to 2.4)*	1.4 (1.3 to 1.5)*	1.2 (1.2 to 1.3)*	-2.4 (-2.7 to -1.9)*	-1.5 (-1.6 to -1.3)*	-2.1 (-2.3 to -1.7)*
	Gabon	SII	2.9 (-3.8 to 9.0)	-4.5 (-12.0 to 1.9)	-7.7 (-15.3 to -1.2)*	-0.4 (-0.5 to -0.2)*	-0.3 (-0.5 to -0.1)*	-0.4 (-0.5 to -0.2)*
		RII	1.1 (0.9 to 1.3)	0.9 (0.8 to 1.0)	0.9 (0.8 to 1.0)*	-0.8 (-1.3 to -0.4)*	-0.4 (-0.7 to -0.1)*	-0.6 (-1.1 to -0.3)*
	Gambia	SII	1.6 (-0.3 to 4.7)	0.4 (-2.2 to 2.3)	-0.4 (-3.5 to 1.7)	-0.1 (-0.2 to 0.0)*	-0.1 (-0.2 to 0.0)*	-0.1 (-0.1 to 0.0)*
		RII	1.0 (1.0 to 1.1)	1.0 (1.0 to 1.0)	1.0 (1.0 to 1.0)	-0.1 (-0.2 to 0.0)*	-0.1 (-0.2 to 0.0)*	-0.1 (-0.2 to 0.0)*
	Ghana	SII	14.8 (10.6 to 20.1)*	3.9 (2.2 to 5.7)*	1.8 (0.7 to 3.3)*	-0.6 (-0.8 to -0.3)*	-0.2 (-0.3 to -0.1)*	-0.4 (-0.6 to -0.3)*
		RII	1.2 (1.1 to 1.3)*	1.0 (1.0 to 1.1)*	1.0 (1.0 to 1.0)*	-0.7 (-1.0 to -0.4)*	-0.2 (-0.3 to -0.1)*	-0.5 (-0.8 to -0.3)*
	Guinea	SII	32.5 (28.0 to 36.9)*	30.3 (25.8 to 34.8)*	27.3 (22.2 to 33.1)*	-0.1 (-0.3 to 0.1)	-0.3 (-0.5 to -0.1)*	-0.2 (-0.3 to 0.0)
		RII	1.6 (1.5 to 1.8)*	1.7 (1.6 to 1.9)*	1.7 (1.5 to 2.0)*	0.3 (-0.2 to 0.7)	0.0 (-0.4 to 0.6)	0.2 (-0.3 to 0.7)
	Kenya	SII	28.4 (24.1 to 33.2)*	4.8 (4.0 to 6.0)*	1.6 (1.1 to 2.3)*	-1.2 (-1.4 to -0.9)*	-0.3 (-0.4 to -0.3)*	-0.9 (-1.1 to -0.7)*
		RII	1.5 (1.4 to 1.6)*	1.1 (1.0 to 1.1)*	1.0 (1.0 to 1.0)*	-1.7 (-2.1 to -1.3)*	-0.3 (-0.4 to -0.3)*	-1.2 (-1.5 to -1.0)*
	Lesotho	SII	9.9 (5.8 to 14.6)*	6.0 (2.4 to 10.4)*	4.0 (0.1 to 9.5)*	-0.2 (-0.4 to 0.0)*	-0.2 (-0.3 to -0.1)*	-0.2 (-0.4 to 0.0)*
		RII	1.1 (1.1 to 1.2)*	1.1 (1.0 to 1.1)*	1.0 (1.0 to 1.1)*	-0.3 (-0.5 to 0.0)	-0.2 (-0.3 to 0.0)*	-0.3 (-0.4 to 0.0)*
	Liberia	SII	30.6 (25.5 to 35.6)*	14.7 (11.4 to 17.5)*	8.3 (5.6 to 11.0)*	-0.8 (-1.0 to -0.6)*	-0.6 (-0.8 to -0.5)*	-0.8 (-0.9 to -0.6)*
		RII	1.7 (1.5 to 1.9)*	1.2 (1.1 to 1.2)*	1.1 (1.1 to 1.1)*	-1.7 (-2.2 to -1.2)*	-0.9 (-1.0 to -0.7)*	-1.4 (-1.8 to -1.0)*
	Madagascar	SII	51.6 (46.5 to 56.8)*	22.3 (16.2 to 30.3)*	11.9 (5.9 to 22.1)*	-1.4 (-2.0 to -0.9)*	-1.0 (-1.1 to -0.8)*	-1.3 (-1.6 to -0.9)*
		RII	2.3 (2.0 to 2.7)*	1.3 (1.2 to 1.5)*	1.1 (1.1 to 1.3)*	-2.9 (-3.9 to -1.7)*	-1.3 (-1.4 to -1.1)*	-2.3 (-3.0 to -1.6)*
	Malawi	SII	13.3 (11.0 to 15.5)*	3.7 (2.8 to 4.8)*	1.7 (1.1 to 2.5)*	-0.5 (-0.6 to -0.4)*	-0.2 (-0.2 to -0.2)*	-0.4 (-0.5 to -0.3)*
		RII	1.2 (1.1 to 1.2)*	1.0 (1.0 to 1.1)*	1.0 (1.0 to 1.0)*	-0.6 (-0.7 to -0.5)*	-0.2 (-0.2 to -0.2)*	-0.5 (-0.6 to -0.4)*
	Mali	SII	29.9 (26.0 to 33.4)*	15.5 (12.8 to 18.6)*	9.4 (6.9 to 12.2)*	-0.7 (-0.9 to -0.6)*	-0.6 (-0.7 to -0.5)*	-0.7 (-0.8 to -0.6)*
		RII	1.7 (1.6 to 1.8)*	1.2 (1.2 to 1.3)*	1.1 (1.1 to 1.2)*	-1.6 (-1.9 to -1.3)*	-0.9 (-1.0 to -0.8)*	-1.4 (-1.6 to -1.1)*
	Morocco	SII	9.6 (5.9 to 15.5)*	2.1 (0.3 to 10.4)*	0.9 (0.0 to 12.8)*	-0.4 (-0.7 to 0.2)	-0.1 (-0.2 to 0.2)	-0.3 (-0.5 to 0.2)
		RII	1.1 (1.1 to 1.2)*	1.0 (1.0 to 1.1)*	1.0 (1.0 to 1.2)*	-0.4 (-0.8 to 0.2)	-0.1 (-0.2 to 0.3)	-0.3 (-0.6 to 0.2)
	Mozambique	SII	36.1 (31.8 to 40.7)*	17.2 (13.4 to 21.9)*	10.7 (7.2 to 16.3)*	-1.0 (-1.2 to -0.6)*	-0.6 (-0.7 to -0.5)*	-0.9 (-1.0 to -0.6)*
		RII	1.6 (1.5 to 1.8)*	1.2 (1.2 to 1.3)*	1.1 (1.1 to 1.2)*	-1.5 (-1.9 to -1.0)*	-0.8 (-0.9 to -0.6)*	-1.3 (-1.6 to -0.9)*

Indicators	Country	Index	Estimates of Inequalities			Changes in Inequalities		
			Year 2000	Year 2020	Year 2030	2000–2020	2020–2030	2000–2030
	Namibia	SII	8.2 (4.6 to 12.0)*	4.6 (0.8 to 8.5)*	2.7 (-0.9 to 7.3)	-0.2 (-0.3 to 0.0)*	-0.2 (-0.3 to -0.1)*	-0.2 (-0.3 to 0.0)*
		RII	1.1 (1.1 to 1.2)*	1.1 (1.0 to 1.1)*	1.0 (1.0 to 1.1)	-0.2 (-0.4 to 0.0)*	-0.2 (-0.3 to 0.0)*	-0.2 (-0.3 to 0.0)*
	Niger	SII	30.3 (25.2 to 34.4)*	6.1 (4.2 to 8.3)*	1.2 (0.6 to 2.2)*	-1.2 (-1.4 to -1.0)*	-0.5 (-0.6 to -0.4)*	-1.0 (-1.1 to -0.8)*
		RII	2.2 (1.9 to 2.5)*	1.1 (1.0 to 1.1)*	1.0 (1.0 to 1.0)*	-3.4 (-4.2 to -2.9)*	-0.5 (-0.7 to -0.4)*	-2.5 (-3.0 to -2.0)*
	Nigeria	SII	48.7 (46.4 to 51.3)*	36.6 (34.6 to 38.8)*	23.8 (21.2 to 26.7)*	-0.6 (-0.7 to -0.5)*	-1.3 (-1.4 to -1.1)*	-0.8 (-0.9 to -0.7)*
		RII	3.7 (3.4 to 4.0)*	1.7 (1.7 to 1.8)*	1.3 (1.3 to 1.4)*	-3.7 (-4.1 to -3.3)*	-2.4 (-2.6 to -2.3)*	-3.3 (-3.6 to -3.0)*
	Rwanda	SII	6.1 (3.5 to 8.7)*	0.7 (0.0 to 1.6)	0.1 (-0.4 to 0.5)	-0.3 (-0.4 to -0.2)*	-0.1 (-0.1 to 0.0)*	-0.2 (-0.3 to -0.1)*
		RII	1.1 (1.0 to 1.1)*	1.0 (1.0 to 1.0)	1.0 (1.0 to 1.0)	-0.3 (-0.4 to -0.2)*	-0.1 (-0.1 to 0.0)*	-0.2 (-0.3 to -0.1)*
	Sao Tome and Principe	SII	12.1 (3.2 to 25.7)*	2.2 (-0.5 to 11.0)	0.7 (-1.4 to 10.4)	-0.5 (-1.2 to 0.1)	-0.1 (-0.3 to 0.1)	-0.4 (-0.8 to 0.1)
		RII	1.2 (1.0 to 1.5)*	1.0 (1.0 to 1.1)	1.0 (1.0 to 1.1)	-0.6 (-1.7 to 0.2)	-0.1 (-0.3 to 0.2)	-0.4 (-1.2 to 0.2)
	Senegal	SII	25.9 (21.7 to 29.6)*	6.4 (5.2 to 7.5)*	2.7 (2.0 to 3.6)*	-1.0 (-1.2 to -0.8)*	-0.4 (-0.4 to -0.3)*	-0.8 (-0.9 to -0.6)*
		RII	1.4 (1.3 to 1.5)*	1.1 (1.1 to 1.1)*	1.0 (1.0 to 1.0)*	-1.4 (-1.7 to -1.1)*	-0.4 (-0.5 to -0.3)*	-1.0 (-1.3 to -0.8)*
	Sierra Leone	SII	13.7 (8.2 to 19.8)*	2.2 (0.4 to 4.5)*	0.4 (-0.6 to 1.4)	-0.6 (-0.8 to -0.4)*	-0.2 (-0.3 to -0.1)*	-0.4 (-0.6 to -0.3)*
		RII	1.3 (1.2 to 1.4)*	1.0 (1.0 to 1.1)*	1.0 (1.0 to 1.0)	-1.1 (-1.6 to -0.6)*	-0.2 (-0.3 to -0.1)*	-0.8 (-1.2 to -0.5)*
	South Africa	SII	8.6 (-2.9 to 18.1)	1.7 (-7.3 to 9.5)	-1.0 (-9.6 to 6.8)	-0.4 (-0.6 to -0.1)*	-0.3 (-0.5 to 0.1)	-0.3 (-0.5 to -0.1)*
		RII	1.2 (0.9 to 1.5)	1.0 (0.9 to 1.1)	1.0 (0.9 to 1.1)	-0.6 (-1.8 to 0.0)*	-0.3 (-0.8 to 0.2)	-0.5 (-1.3 to 0.0)
	Tanzania	SII	16.7 (13.8 to 20.4)*	11.1 (8.6 to 13.7)*	8.8 (6.0 to 12.3)*	-0.3 (-0.5 to -0.1)*	-0.2 (-0.3 to -0.1)*	-0.3 (-0.4 to -0.1)*
		RII	1.2 (1.2 to 1.3)*	1.1 (1.1 to 1.2)*	1.1 (1.1 to 1.1)*	-0.3 (-0.6 to -0.1)*	-0.3 (-0.4 to -0.1)*	-0.3 (-0.5 to -0.1)*
	Togo	SII	14.4 (4.3 to 24.6)*	2.8 (0.3 to 7.9)*	0.8 (-1.1 to 4.9)	-0.6 (-1.0 to -0.1)*	-0.2 (-0.3 to -0.1)*	-0.4 (-0.8 to -0.1)*
		RII	1.2 (1.1 to 1.6)*	1.0 (1.0 to 1.1)*	1.0 (1.0 to 1.1)	-0.8 (-2.2 to -0.1)*	-0.2 (-0.4 to -0.1)*	-0.6 (-1.5 to -0.1)*
	Uganda	SII	11.0 (7.5 to 14.5)*	1.4 (-0.2 to 3.1)	0.0 (-1.1 to 1.1)	-0.5 (-0.6 to -0.3)*	-0.1 (-0.2 to -0.1)*	-0.4 (-0.5 to -0.3)*
		RII	1.2 (1.1 to 1.3)*	1.0 (1.0 to 1.0)	1.0 (1.0 to 1.0)	-0.8 (-1.1 to -0.6)*	-0.2 (-0.3 to -0.1)*	-0.6 (-0.8 to -0.4)*
	Zambia	SII	19.4 (16.8 to 22.8)*	8.5 (6.8 to 10.1)*	5.1 (3.7 to 6.7)*	-0.5 (-0.7 to -0.4)*	-0.3 (-0.4 to -0.3)*	-0.5 (-0.6 to -0.4)*
		RII	1.3 (1.2 to 1.3)*	1.1 (1.1 to 1.1)*	1.1 (1.0 to 1.1)*	-0.7 (-0.9 to -0.5)*	-0.4 (-0.4 to -0.3)*	-0.6 (-0.8 to -0.5)*
	Zimbabwe	SII	22.3 (16.3 to 27.1)*	6.7 (3.8 to 9.7)*	2.5 (0.9 to 4.4)*	-0.8 (-0.9 to -0.6)*	-0.4 (-0.5 to -0.3)*	-0.7 (-0.8 to -0.5)*
		RII	1.5 (1.3 to 1.6)*	1.1 (1.0 to 1.1)*	1.0 (1.0 to 1.0)*	-1.5 (-1.9 to -1.1)*	-0.5 (-0.6 to -0.3)*	-1.2 (-1.5 to -0.9)*
DPT3 immunization	Angola	SII	54.3 (31.1 to 60.0)*	46.9 (39.4 to 52.7)*	39.1 (16.5 to 48.9)*	-0.4 (-0.6 to 0.6)	-0.8 (-2.4 to -0.3)*	-0.6 (-0.8 to 0.1)
		RII	3.5 (1.6 to 8.5)*	3.7 (2.8 to 4.8)*	3.6 (1.8 to 5.9)*	0.0 (-5.4 to 5.3)	-0.4 (-4.7 to 2.2)	-0.1 (-5.1 to 4.3)
	Benin	SII	34.8 (32.2 to 37.3)*	25.4 (22.5 to 28.5)*	20.8 (16.9 to 25.3)*	-0.5 (-0.7 to -0.3)*	-0.5 (-0.6 to -0.3)*	-0.5 (-0.6 to -0.3)*
		RII	1.7 (1.6 to 1.8)*	1.4 (1.4 to 1.5)*	1.3 (1.2 to 1.4)*	-0.8 (-1.2 to -0.5)*	-0.7 (-0.9 to -0.5)*	-0.8 (-1.1 to -0.5)*
	Burkina Faso	SII	29.4 (24.1 to 34.4)*	0.7 (0.4 to 1.0)*	0.0 (0.0 to 0.1)*	-1.4 (-1.7 to -1.2)*	-0.1 (-0.1 to 0.0)*	-1.0 (-1.1 to -0.8)*
		RII	2.1 (1.8 to 2.4)*	1.0 (1.0 to 1.0)*	1.0 (1.0 to 1.0)*	-3.6 (-4.3 to -2.9)*	-0.1 (-0.1 to 0.0)*	-2.4 (-2.9 to -2.0)*
	Burundi	SII	2.5 (-0.7 to 6.9)	-0.3 (-1.3 to 0.9)	-0.4 (-1.2 to 0.2)	-0.1 (-0.3 to 0.0)*	0.0 (-0.1 to 0.0)	-0.1 (-0.2 to 0.0)
		RII	1.0 (1.0 to 1.1)	1.0 (1.0 to 1.0)	1.0 (1.0 to 1.0)	-0.2 (-0.4 to 0.0)*	0.0 (-0.1 to 0.0)	-0.1 (-0.3 to 0.0)
	Cameroon	SII	39.3 (35.7 to 43.0)*	28.4 (24.5 to 31.5)*	23.2 (18.4 to 27.3)*	-0.5 (-0.7 to -0.4)*	-0.5 (-0.7 to -0.4)*	-0.5 (-0.7 to -0.4)*
		RII	1.9 (1.7 to 2.0)*	1.5 (1.4 to 1.6)*	1.4 (1.3 to 1.5)*	-1.0 (-1.4 to -0.7)*	-0.9 (-1.1 to -0.6)*	-1.0 (-1.3 to -0.6)*
	Chad	SII	20.0 (16.8 to 23.6)*	19.4 (14.3 to 23.8)*	14.7 (8.8 to 20.6)*	0.0 (-0.2 to 0.2)	-0.5 (-0.7 to -0.3)*	-0.2 (-0.4 to 0.0)*
		RII	2.7 (2.3 to 3.4)*	1.6 (1.4 to 1.8)*	1.3 (1.2 to 1.5)*	-2.6 (-3.2 to -2.0)*	-2.0 (-2.5 to -1.7)*	-2.4 (-2.9 to -1.9)*
	Comoros	SII	36.2 (22.9 to 48.4)*	17.3 (8.3 to 29.6)*	9.3 (1.5 to 25.5)*	-1.0 (-1.5 to 0.0)	-0.7 (-1.1 to -0.3)*	-0.9 (-1.4 to -0.1)*
		RII	2.0 (1.3 to 3.2)*	1.2 (1.1 to 1.5)*	1.1 (1.0 to 1.6)*	-2.2 (-5.1 to 0.4)	-1.0 (-1.4 to 0.1)	-1.9 (-3.8 to 0.3)
	Congo Brazzaville	SII	30.3 (25.0 to 37.0)*	33.1 (27.0 to 38.7)*	25.2 (17.5 to 34.4)*	0.1 (-0.1 to 0.4)	-0.8 (-1.2 to -0.3)*	-0.2 (-0.3 to 0.0)
		RII	1.5 (1.4 to 1.7)*	2.0 (1.7 to 2.3)*	2.1 (1.7 to 2.6)*	1.4 (0.5 to 2.4)*	0.6 (-0.1 to 1.5)	1.1 (0.3 to 2.1)*
	Congo Democratic Republic	SII	38.4 (33.9 to 42.5)*	24.9 (22.0 to 28.9)*	11.2 (7.9 to 15.6)*	-0.7 (-0.8 to -0.5)*	-1.4 (-1.5 to -1.2)*	-0.9 (-1.0 to -0.8)*
		RII	3.5 (3.0 to 4.0)*	1.4 (1.3 to 1.5)*	1.1 (1.1 to 1.2)*	-4.4 (-5.1 to -3.6)*	-2.1 (-2.3 to -1.9)*	-3.6 (-4.1 to -3.0)*
	Cote d'Ivoire	SII	34.0 (22.4 to 40.8)*	18.4 (7.3 to 31.2)*	10.3 (0.9 to 25.0)*	-0.8 (-1.1 to 0.0)	-0.8 (-1.1 to -0.4)*	-0.8 (-1.1 to -0.1)*
		RII	2.1 (1.4 to 3.5)*	1.3 (1.1 to 1.8)*	1.1 (1.0 to 1.8)*	-2.3 (-5.4 to 0.7)	-1.2 (-1.5 to 0.3)	-2.0 (-4.0 to 0.6)
	Egypt	SII	5.2 (3.9 to 6.9)*	0.9 (0.4 to 1.4)*	0.2 (0.0 to 0.6)	-0.2 (-0.3 to -0.2)*	-0.1 (-0.1 to 0.0)*	-0.2 (-0.2 to -0.1)*
		RII	1.1 (1.0 to 1.1)*	1.0 (1.0 to 1.0)*	1.0 (1.0 to 1.0)	-0.2 (-0.3 to -0.2)*	-0.1 (-0.1 to 0.0)*	-0.2 (-0.2 to -0.1)*
	Eswatini	SII	5.7 (-0.1 to 17.3)	0.5 (-0.8 to 4.1)	0.1 (-1.8 to 2.7)	-0.2 (-0.9 to 0.0)	0.0 (-0.2 to 0.0)	-0.2 (-0.6 to 0.0)*
		RII	1.1 (1.0 to 1.2)	1.0 (1.0 to 1.0)	1.0 (1.0 to 1.0)	-0.3 (-1.0 to 0.0)	0.0 (-0.2 to 0.0)	-0.2 (-0.7 to 0.0)*
	Ethiopia	SII	30.1 (27.1 to 33.0)*	25.9 (23.6 to 28.7)*	13.4 (10.7 to 16.0)*	-0.2 (-0.4 to -0.1)*	-1.3 (-1.4 to -1.1)*	-0.6 (-0.7 to -0.4)*
		RII	3.4 (3.1 to 3.8)*	1.5 (1.4 to 1.6)*	1.2 (1.1 to 1.2)*	-4.1 (-4.6 to -3.6)*	-2.3 (-2.5 to -2.1)*	-3.5 (-3.9 to -3.1)*
	Gabon	SII	36.9 (30.6 to 43.2)*	8.0 (6.2 to 10.5)*	1.9 (1.2 to 2.9)*	-1.4 (-1.7 to -1.2)*	-0.6 (-0.8 to -0.5)*	-1.2 (-1.4 to -1.0)*
		RII	2.4 (2.1 to 2.8)*	1.1 (1.1 to 1.1)*	1.0 (1.0 to 1.0)*	-3.8 (-4.6 to -3.1)*	-0.7 (-0.9 to -0.5)*	-2.8 (-3.3 to -2.3)*
	Gambia	SII	2.8 (-3.2 to 10.4)	-2.0 (-4.8 to 0.7)	-1.7 (-3.6 to -0.3)*	-0.2 (-0.5 to 0.0)*	0.0 (-0.1 to 0.1)	-0.2 (-0.4 to 0.0)
		RII	1.0 (1.0 to 1.2)	1.0 (0.9 to 1.0)	1.0 (1.0 to 1.0)*	-0.3 (-0.7 to 0.0)	0.0 (-0.1 to 0.1)	-0.2 (-0.5 to 0.1)
	Ghana	SII	19.0 (13.6 to 24.0)*	5.0 (2.7 to 7.2)*	2.1 (0.6 to 3.9)*	-0.7 (-0.9 to -0.5)*	-0.3 (-0.4 to -0.2)*	-0.6 (-0.7 to -0.4)*
		RII	1.3 (1.2 to 1.4)*	1.1 (1.0 to 1.1)*	1.0 (1.0 to 1.0)*	-0.9 (-1.2 to -0.6)*	-0.3 (-0.4 to -0.2)*	-0.7 (-0.9 to -0.5)*
	Guinea	SII	32.2 (26.8 to 36.7)*	22.7 (18.6 to 27.4)*	15.1 (10.8 to 21.1)*	-0.5 (-0.7 to -0.3)*	-0.7 (-0.9 to -0.5)*	-0.6 (-0.7 to -0.4)*
		RII	1.7 (1.5 to 1.9)*	1.8 (1.6 to 2.0)*	1.7 (1.5 to 2.0)*	0.2 (-0.4 to 0.7)	-0.6 (-1.2 to 0.0)	0.0 (-0.6 to 0.5)
	Kenya	SII	26.0 (21.3 to 30.5)*	4.4 (3.2 to 5.7)*	1.3 (0.7 to 2.1)*	-1.1 (-1.3 to -0.9)*	-0.3 (-0.4 to -0.2)*	-0.8 (-1.0 to -0.7)*
		RII	1.5 (1.4 to 1.6)*	1.0 (1.0 to 1.1)*	1.0 (1.0 to 1.0)*	-1.7 (-2.1 to -1.3)*	-0.3 (-0.4 to -0.3)*	-1.2 (-1.5 to -1.0)*
	Lesotho	SII	14.6 (10.1 to 19.5)*	6.9 (3.1 to 10.7)*	3.7 (0.3 to 8.1)*	-0.4 (-0.6 to -0.2)*	-0.3 (-0.4 to -0.2)*	-0.4 (-0.5 to -0.2)*
		RII	1.2 (1.1 to 1.3)*	1.1 (1.0 to 1.1)*	1.0 (1.0 to 1.1)*	-0.5 (-0.8 to -0.2)*	-0.3 (-0.5 to -0.2)*	-0.4 (-0.7 to -0.2)*
	Liberia	SII	37.0 (31.7 to 41.7)*	19.0 (16.1 to 22.6)*	9.6 (7.1 to 12.5)*	-0.9 (-1.1 to -0.7)*	-1.0 (-1.1 to -0.8)*	-0.9 (-1.1 to -0.7)*
		RII	2.3 (2.0 to 2.7)*	1.3 (1.2 to 1.3)*	1.1 (1.1 to 1.2)*	-2.9 (-3.6 to -2.4)*	-1.4 (-1.6 to -1.2)*	-2.4 (-2.9 to -2.0)*
	Madagascar	SII	54.5 (50.0 to 59.6)*	17.2 (12.0 to 24.2)*	6.6 (3.2 to 12.6)*	-1.9 (-2.3 to -1.4)*	-1.0 (-1.2 to -0.9)*	-1.6 (-1.8 to -1.3)*
		RII	2.7 (2.4 to 3.2)*	1.2 (1.1 to 1.3)*	1.1 (1.0 to 1.1)*	-3.9 (-4.9 to -2.8)*	-1.2 (-1.5 to -1.0)*	-3.0 (-3.7 to -2.3)*
	Malawi	SII	12.9 (10.3 to 15.1)*	2.6 (1.7 to 3.5)*	0.9 (0.2 to 1.5)*	-0.5 (-0.6 to -0.4)*	-0.2 (-0.2 to -0.1)*	-0.4 (-0.5 to -0.3)*

Indicators	Country	Index	Estimates of Inequalities			Changes in Inequalities		
			Year 2000	Year 2020	Year 2030	2000–2020	2020–2030	2000–2030
		RII	1.2 (1.1 to 1.2)*	1.0 (1.0 to 1.0)*	1.0 (1.0 to 1.0)*	-0.6 (-0.8 to -0.5)*	-0.2 (-0.2 to -0.1)*	-0.5 (-0.6 to -0.4)*
	Mali	SII	31.3 (28.7 to 33.9)*	13.5 (11.0 to 15.8)*	5.9 (4.0 to 7.9)*	-0.9 (-1.0 to -0.8)*	-0.8 (-0.8 to -0.7)*	-0.8 (-1.0 to -0.8)*
		RII	2.0 (1.8 to 2.1)*	1.2 (1.1 to 1.2)*	1.1 (1.0 to 1.1)*	-2.5 (-2.8 to -2.2)*	-1.0 (-1.1 to -0.9)*	-2.0 (-2.2 to -1.8)*
	Morocco	SII	10.4 (6.2 to 17.9)*	1.4 (0.2 to 11.3)*	0.5 (0.0 to 12.8)*	-0.4 (-0.9 to 0.2)	-0.1 (-0.2 to 0.2)	-0.3 (-0.6 to 0.2)
		RII	1.1 (1.1 to 1.2)*	1.0 (1.0 to 1.1)*	1.0 (1.0 to 1.2)*	-0.5 (-1.0 to 0.3)	-0.1 (-0.2 to 0.3)	-0.3 (-0.7 to 0.3)
	Mozambique	SII	37.4 (33.3 to 41.5)*	26.6 (21.0 to 31.1)*	21.3 (14.6 to 28.5)*	-0.6 (-0.9 to -0.3)*	-0.5 (-0.7 to -0.3)*	-0.5 (-0.8 to -0.3)*
		RII	1.7 (1.6 to 1.8)*	1.4 (1.3 to 1.5)*	1.3 (1.2 to 1.5)*	-0.9 (-1.5 to -0.4)*	-0.8 (-1.0 to -0.4)*	-0.9 (-1.3 to -0.4)*
	Namibia	SII	9.7 (5.8 to 13.9)*	2.7 (-0.8 to 6.0)	0.0 (-3.2 to 3.6)	-0.4 (-0.5 to -0.2)*	-0.3 (-0.4 to -0.2)*	-0.3 (-0.4 to -0.2)*
		RII	1.1 (1.1 to 1.2)*	1.0 (1.0 to 1.1)	1.0 (1.0 to 1.0)	-0.4 (-0.6 to -0.3)*	-0.3 (-0.4 to -0.2)*	-0.4 (-0.5 to -0.3)*
	Niger	SII	23.5 (19.6 to 28.3)*	9.3 (6.9 to 11.9)*	1.3 (0.7 to 2.2)*	-0.7 (-0.9 to -0.6)*	-0.8 (-1.0 to -0.6)*	-0.7 (-0.9 to -0.6)*
		RII	3.5 (2.9 to 4.1)*	1.1 (1.1 to 1.1)*	1.0 (1.0 to 1.0)*	-5.6 (-6.3 to -4.8)*	-0.9 (-1.1 to -0.7)*	-4.0 (-4.5 to -3.5)*
	Nigeria	SII	54.3 (51.5 to 56.9)*	63.6 (62.1 to 65.7)*	51.5 (48.3 to 55.2)*	0.5 (0.3 to 0.6)*	-1.2 (-1.4 to -1.0)*	-0.1 (-0.2 to 0.0)
		RII	14.1 (12.8 to 15.8)*	4.0 (3.8 to 4.3)*	2.3 (2.1 to 2.5)*	-6.1 (-6.7 to -5.4)*	-5.5 (-5.8 to -5.0)*	-5.9 (-6.4 to -5.3)*
	Rwanda	SII	5.4 (2.4 to 8.7)*	-0.2 (-1.0 to 0.6)	-0.4 (-0.9 to 0.0)	-0.3 (-0.4 to -0.2)*	0.0 (-0.1 to 0.0)	-0.2 (-0.3 to -0.1)*
		RII	1.1 (1.0 to 1.1)*	1.0 (1.0 to 1.0)	1.0 (1.0 to 1.0)	-0.3 (-0.5 to -0.2)*	0.0 (-0.1 to 0.0)	-0.2 (-0.4 to -0.1)*
	Sao Tome and Principe	SII	15.0 (3.0 to 31.4)*	2.2 (-0.8 to 9.5)	0.5 (-1.5 to 7.2)	-0.6 (-1.5 to 0.0)	-0.2 (-0.4 to 0.0)	-0.4 (-1.0 to -0.1)*
		RII	1.2 (1.0 to 1.7)*	1.0 (1.0 to 1.1)	1.0 (1.0 to 1.1)	-0.8 (-2.4 to 0.1)	-0.2 (-0.4 to 0.0)	-0.6 (-1.7 to 0.0)*
	Senegal	SII	29.0 (25.6 to 33.1)*	7.3 (6.0 to 8.3)*	2.9 (2.0 to 3.7)*	-1.1 (-1.3 to -0.9)*	-0.4 (-0.5 to -0.4)*	-0.9 (-1.0 to -0.7)*
		RII	1.5 (1.4 to 1.6)*	1.1 (1.1 to 1.1)*	1.0 (1.0 to 1.0)*	-1.6 (-2.0 to -1.3)*	-0.5 (-0.5 to -0.4)*	-1.3 (-1.5 to -1.0)*
	Sierra Leone	SII	12.2 (7.4 to 17.2)*	0.8 (-1.3 to 2.7)	-0.5 (-1.5 to 0.2)	-0.6 (-0.8 to -0.4)*	-0.1 (-0.2 to 0.0)	-0.4 (-0.6 to -0.3)*
		RII	1.4 (1.2 to 1.5)*	1.0 (1.0 to 1.0)	1.0 (1.0 to 1.0)	-1.5 (-2.1 to -1.0)*	-0.1 (-0.3 to 0.0)	-1.0 (-1.5 to -0.7)*
	South Africa	SII	5.8 (-4.1 to 16.8)	-3.5 (-13.6 to 7.2)	-6.1 (-17.7 to 1.1)	-0.5 (-0.7 to -0.2)*	-0.3 (-0.7 to 0.3)	-0.4 (-0.7 to -0.1)*
		RII	1.1 (0.9 to 1.5)	0.9 (0.8 to 1.1)	0.9 (0.7 to 1.0)	-0.9 (-1.9 to -0.1)*	-0.4 (-1.7 to 0.5)	-0.8 (-1.5 to 0.0)
	Tanzania	SII	18.6 (15.1 to 22.5)*	13.4 (10.9 to 16.8)*	10.9 (7.7 to 15.8)*	-0.3 (-0.5 to 0.0)*	-0.2 (-0.4 to -0.1)*	-0.3 (-0.5 to 0.0)*
		RII	1.2 (1.2 to 1.3)*	1.2 (1.1 to 1.2)*	1.1 (1.1 to 1.2)*	-0.3 (-0.6 to 0.0)*	-0.3 (-0.4 to -0.1)*	-0.3 (-0.6 to 0.0)*
	Togo	SII	17.5 (6.4 to 27.1)*	3.0 (-0.5 to 8.3)	0.5 (-1.0 to 4.8)	-0.7 (-1.1 to -0.2)*	-0.3 (-0.4 to -0.1)*	-0.5 (-0.9 to -0.2)*
		RII	1.3 (1.1 to 1.9)*	1.0 (1.0 to 1.1)	1.0 (1.0 to 1.1)	-1.3 (-2.9 to -0.2)*	-0.3 (-0.5 to -0.1)*	-0.9 (-2.0 to -0.3)*
	Uganda	SII	11.7 (7.2 to 15.5)*	0.4 (-2.0 to 2.2)	-1.3 (-2.9 to 0.1)	-0.6 (-0.7 to -0.4)*	-0.2 (-0.2 to -0.1)*	-0.4 (-0.6 to -0.3)*
		RII	1.3 (1.2 to 1.4)*	1.0 (1.0 to 1.0)	1.0 (1.0 to 1.0)	-1.2 (-1.5 to -0.8)*	-0.2 (-0.3 to -0.1)*	-0.8 (-1.1 to -0.6)*
	Zambia	SII	23.4 (20.1 to 26.6)*	8.2 (6.6 to 10.0)*	4.3 (2.8 to 5.7)*	-0.7 (-0.9 to -0.6)*	-0.4 (-0.5 to -0.3)*	-0.6 (-0.7 to -0.5)*
		RII	1.4 (1.3 to 1.4)*	1.1 (1.1 to 1.1)*	1.0 (1.0 to 1.1)*	-1.0 (-1.3 to -0.9)*	-0.5 (-0.5 to -0.4)*	-0.8 (-1.0 to -0.7)*
	Zimbabwe	SII	20.8 (15.2 to 26.5)*	3.4 (1.3 to 5.6)*	0.6 (-0.3 to 1.7)	-0.9 (-1.1 to -0.7)*	-0.3 (-0.4 to -0.2)*	-0.7 (-0.8 to -0.5)*
		RII	1.5 (1.3 to 1.6)*	1.0 (1.0 to 1.1)*	1.0 (1.0 to 1.0)	-1.7 (-2.2 to -1.2)*	-0.3 (-0.5 to -0.2)*	-1.2 (-1.6 to -0.9)*

Notes: SII = Slope Index of Inequality; RII = Relative Index of Inequality

Table M. Education-related inequality in childhood immunization coverage, 2000–2030

Indicators	Country	Index	Estimates of Inequalities			Changes in Inequalities		
			Year 2000	Year 2020	Year 2030	2000–2020	2020–2030	2000–2030
Full immunization	Angola	SII	38.7 (21.7 to 44.5)*	27.5 (21.8 to 32.3)*	19.2 (6.5 to 27.2)*	-0.6 (-0.8 to 0.1)	-0.8 (-1.6 to -0.4)*	-0.7 (-0.9 to -0.1)*
		RII	2.4 (1.4 to 4.0)*	2.3 (2.0 to 2.8)*	2.1 (1.5 to 2.8)*	-0.2 (-3.4 to 3.7)	-0.9 (-2.8 to 0.1)	-0.4 (-3.2 to 2.3)
	Benin	SII	27.9 (25.6 to 30.3)*	16.3 (13.1 to 18.9)*	10.7 (7.0 to 14.0)*	-0.6 (-0.7 to -0.5)*	-0.6 (-0.7 to -0.4)*	-0.6 (-0.7 to -0.5)*
		RII	1.5 (1.5 to 1.6)*	1.3 (1.2 to 1.3)*	1.2 (1.1 to 1.2)*	-1.0 (-1.3 to -0.8)*	-0.8 (-1.0 to -0.7)*	-0.9 (-1.2 to -0.8)*
	Burkina Faso	SII	24.3 (16.9 to 32.4)*	0.7 (0.3 to 1.0)*	0.0 (0.0 to 0.1)	-1.2 (-1.6 to -0.8)*	-0.1 (-0.1 to 0.0)*	-0.8 (-1.1 to -0.6)*
		RII	1.9 (1.6 to 2.2)*	1.0 (1.0 to 1.0)*	1.0 (1.0 to 1.0)	-3.1 (-3.8 to -2.3)*	-0.1 (-0.1 to 0.0)*	-2.1 (-2.6 to -1.5)*
	Burundi	SII	3.8 (1.8 to 5.9)*	0.9 (-2.7 to 3.4)	-1.7 (-6.8 to 1.3)	-0.1 (-0.3 to 0.0)*	-0.3 (-0.5 to -0.2)*	-0.2 (-0.3 to -0.1)*
		RII	1.0 (1.0 to 1.1)*	1.0 (1.0 to 1.0)	1.0 (0.9 to 1.0)	-0.2 (-0.3 to 0.0)*	-0.3 (-0.6 to -0.2)*	-0.2 (-0.4 to -0.1)*
	Cameroon	SII	31.5 (28.5 to 34.2)*	20.7 (17.9 to 23.3)*	15.0 (11.5 to 17.9)*	-0.5 (-0.7 to -0.4)*	-0.6 (-0.7 to -0.5)*	-0.5 (-0.7 to -0.4)*
		RII	1.7 (1.6 to 1.8)*	1.4 (1.4 to 1.5)*	1.3 (1.2 to 1.4)*	-0.9 (-1.2 to -0.7)*	-1.0 (-1.2 to -0.7)*	-0.9 (-1.2 to -0.7)*
	Chad	SII	18.9 (14.2 to 24.2)*	21.9 (16.4 to 26.5)*	14.4 (9.3 to 19.5)*	0.1 (-0.1 to 0.3)	-0.7 (-1.0 to -0.5)*	-0.2 (-0.3 to 0.0)*
		RII	3.2 (2.7 to 3.8)*	1.6 (1.5 to 1.8)*	1.3 (1.2 to 1.4)*	-3.4 (-4.0 to -2.8)*	-2.5 (-2.9 to -2.1)*	-3.1 (-3.6 to -2.6)*
	Comoros	SII	17.4 (9.7 to 25.1)*	4.9 (-0.9 to 11.8)	0.2 (-5.4 to 6.0)	-0.6 (-0.8 to -0.2)*	-0.5 (-0.7 to -0.2)*	-0.6 (-0.7 to -0.3)*
		RII	1.4 (1.1 to 1.9)*	1.1 (1.0 to 1.2)	1.0 (0.9 to 1.1)	-1.4 (-2.8 to 0.0)*	-0.7 (-1.2 to -0.2)*	-1.1 (-2.1 to -0.4)*
	Congo Brazzaville	SII	22.4 (19.4 to 26.2)*	10.6 (7.3 to 14.5)*	4.1 (0.6 to 7.9)*	-0.6 (-0.7 to -0.5)*	-0.7 (-0.8 to -0.6)*	-0.6 (-0.7 to -0.5)*
		RII	1.5 (1.4 to 1.6)*	1.3 (1.2 to 1.5)*	1.2 (1.0 to 1.3)*	-0.4 (-0.9 to 0.1)	-1.3 (-1.9 to -0.9)*	-0.8 (-1.2 to -0.2)*
	Congo Democratic Republic	SII	19.1 (16.0 to 22.2)*	10.9 (8.3 to 13.6)*	3.3 (1.5 to 5.5)*	-0.4 (-0.6 to -0.3)*	-0.8 (-0.9 to -0.7)*	-0.5 (-0.6 to -0.4)*
		RII	2.1 (1.9 to 2.4)*	1.2 (1.1 to 1.2)*	1.0 (1.0 to 1.1)*	-3.0 (-3.4 to -2.5)*	-1.2 (-1.4 to -1.0)*	-2.4 (-2.7 to -2.1)*
	Cote d'Ivoire	SII	32.8 (23.2 to 39.9)*	19.5 (10.5 to 27.9)*	12.3 (2.5 to 22.3)*	-0.7 (-1.1 to 0.1)	-0.7 (-1.1 to -0.5)*	-0.7 (-1.0 to -0.2)*
		RII	1.8 (1.4 to 2.8)*	1.3 (1.1 to 1.7)*	1.2 (1.0 to 1.8)*	-1.6 (-4.2 to 1.1)	-1.1 (-1.4 to 0.2)	-1.4 (-3.2 to 0.8)
	Egypt	SII	3.8 (2.7 to 4.6)*	0.6 (-1.6 to 2.8)	-4.3 (-8.0 to -0.4)*	-0.2 (-0.2 to -0.1)*	-0.5 (-0.7 to -0.3)*	-0.3 (-0.4 to -0.1)*
		RII	1.0 (1.0 to 1.1)*	1.0 (1.0 to 1.0)	0.9 (0.9 to 1.0)*	-0.2 (-0.3 to 0.0)*	-0.7 (-1.0 to -0.4)*	-0.3 (-0.5 to -0.2)*
	Eswatini	SII	10.5 (4.7 to 19.7)*	1.3 (-0.9 to 8.2)	0.1 (-3.6 to 4.8)	-0.4 (-0.9 to 0.0)	-0.1 (-0.5 to 0.0)*	-0.3 (-0.6 to -0.1)*
		RII	1.1 (1.1 to 1.3)*	1.0 (1.0 to 1.1)	1.0 (1.0 to 1.1)	-0.5 (-1.3 to 0.0)	-0.2 (-0.6 to 0.0)*	-0.4 (-0.9 to 0.0)*
	Ethiopia	SII	33.1 (29.3 to 37.5)*	28.9 (25.5 to 32.0)*	17.7 (14.6 to 20.7)*	-0.2 (-0.4 to -0.1)*	-1.1 (-1.3 to -1.0)*	-0.5 (-0.6 to -0.4)*
		RII	3.6 (3.3 to 4.0)*	1.6 (1.5 to 1.7)*	1.3 (1.2 to 1.3)*	-3.9 (-4.3 to -3.5)*	-2.5 (-2.7 to -2.3)*	-3.4 (-3.8 to -3.1)*
	Gabon	SII	4.5 (2.2 to 7.9)*	-4.7 (-9.8 to 0.4)	-9.4 (-15.0 to -4.8)*	-0.5 (-0.6 to -0.3)*	-0.5 (-0.7 to -0.3)*	-0.5 (-0.6 to -0.3)*
		RII	1.3 (1.1 to 1.5)*	0.9 (0.8 to 1.0)	0.9 (0.8 to 0.9)*	-1.8 (-2.2 to -1.4)*	-0.5 (-0.9 to -0.1)*	-1.3 (-1.7 to -1.0)*
	Gambia	SII	6.2 (2.7 to 10.5)*	-1.3 (-4.1 to 1.3)	-3.4 (-6.3 to -1.0)*	-0.4 (-0.5 to -0.3)*	-0.2 (-0.4 to -0.1)*	-0.3 (-0.4 to -0.3)*
		RII	1.1 (1.0 to 1.1)*	1.0 (1.0 to 1.0)	1.0 (0.9 to 1.0)*	-0.5 (-0.7 to -0.3)*	-0.2 (-0.4 to -0.1)*	-0.4 (-0.5 to -0.3)*
	Ghana	SII	16.0 (12.8 to 21.0)*	4.1 (1.4 to 7.1)*	0.8 (-1.5 to 3.1)	-0.6 (-0.7 to -0.5)*	-0.3 (-0.4 to -0.3)*	-0.5 (-0.6 to -0.4)*
		RII	1.2 (1.2 to 1.3)*	1.0 (1.0 to 1.1)*	1.0 (1.0 to 1.0)	-0.9 (-1.1 to -0.7)*	-0.4 (-0.5 to -0.3)*	-0.7 (-0.9 to -0.6)*
	Guinea	SII	27.3 (22.7 to 31.1)*	15.4 (10.4 to 19.6)*	7.6 (3.3 to 11.1)*	-0.6 (-0.7 to -0.5)*	-0.8 (-0.9 to -0.6)*	-0.7 (-0.8 to -0.6)*
		RII	1.6 (1.5 to 1.7)*	1.6 (1.4 to 1.8)*	1.4 (1.2 to 1.7)*	0.1 (-0.5 to 0.6)	-1.1 (-1.7 to -0.6)*	-0.3 (-0.8 to 0.2)
	Kenya	SII	24.1 (20.7 to 27.1)*	5.7 (4.1 to 7.4)*	1.7 (0.5 to 2.8)*	-0.9 (-1.0 to -0.8)*	-0.4 (-0.5 to -0.3)*	-0.7 (-0.8 to -0.6)*
		RII	1.5 (1.4 to 1.6)*	1.1 (1.0 to 1.1)*	1.0 (1.0 to 1.0)*	-1.6 (-1.9 to -1.4)*	-0.5 (-0.6 to -0.4)*	-1.3 (-1.4 to -1.1)*
	Lesotho	SII	12.6 (8.8 to 16.3)*	1.8 (-1.7 to 5.5)	-2.8 (-6.3 to 0.7)	-0.5 (-0.7 to -0.4)*	-0.5 (-0.6 to -0.4)*	-0.5 (-0.6 to -0.4)*
		RII	1.2 (1.1 to 1.3)*	1.0 (1.0 to 1.1)	1.0 (0.9 to 1.0)	-0.8 (-1.0 to -0.6)*	-0.6 (-0.9 to -0.4)*	-0.7 (-0.9 to -0.6)*
	Liberia	SII	21.1 (17.1 to 25.6)*	8.7 (4.4 to 11.8)*	2.5 (-1.6 to 6.0)	-0.6 (-0.8 to -0.5)*	-0.6 (-0.7 to -0.5)*	-0.6 (-0.8 to -0.5)*
		RII	1.6 (1.4 to 1.7)*	1.1 (1.1 to 1.2)*	1.0 (1.0 to 1.1)	-1.6 (-2.0 to -1.3)*	-1.0 (-1.2 to -0.8)*	-1.4 (-1.7 to -1.1)*
	Madagascar	SII	34.6 (29.9 to 38.3)*	10.9 (7.7 to 14.4)*	4.2 (2.1 to 7.2)*	-1.2 (-1.4 to -0.9)*	-0.7 (-0.7 to -0.5)*	-1.0 (-1.2 to -0.8)*
		RII	1.9 (1.7 to 2.1)*	1.1 (1.1 to 1.2)*	1.0 (1.0 to 1.1)*	-2.4 (-3.0 to -1.9)*	-0.8 (-1.0 to -0.6)*	-1.9 (-2.3 to -1.5)*
	Malawi	SII	17.4 (15.3 to 19.6)*	4.8 (3.3 to 6.1)*	1.8 (0.6 to 2.8)*	-0.6 (-0.7 to -0.6)*	-0.3 (-0.3 to -0.3)*	-0.5 (-0.6 to -0.5)*
		RII	1.3 (1.2 to 1.3)*	1.1 (1.0 to 1.1)*	1.0 (1.0 to 1.0)*	-0.9 (-0.9 to -0.8)*	-0.3 (-0.4 to -0.3)*	-0.7 (-0.8 to -0.6)*
	Mali	SII	30.1 (25.9 to 33.7)*	14.8 (11.7 to 17.8)*	7.8 (4.7 to 10.8)*	-0.8 (-0.9 to -0.6)*	-0.7 (-0.8 to -0.6)*	-0.7 (-0.8 to -0.6)*
		RII	1.9 (1.7 to 2.0)*	1.2 (1.2 to 1.3)*	1.1 (1.1 to 1.1)*	-2.0 (-2.2 to -1.7)*	-1.1 (-1.2 to -1.0)*	-1.7 (-1.9 to -1.5)*
	Morocco	SII	10.1 (6.2 to 14.4)*	2.2 (0.3 to 12.6)*	0.7 (0.0 to 12.0)	-0.4 (-0.7 to 0.2)	-0.1 (-0.3 to 0.0)*	-0.3 (-0.5 to 0.2)
		RII	1.1 (1.1 to 1.2)*	1.0 (1.0 to 1.2)*	1.0 (1.0 to 1.2)	-0.5 (-0.8 to 0.4)	-0.1 (-0.3 to 0.2)	-0.3 (-0.5 to 0.3)
	Mozambique	SII	24.9 (22.1 to 27.9)*	15.6 (12.1 to 20.1)*	11.2 (6.9 to 16.5)*	-0.5 (-0.6 to -0.3)*	-0.4 (-0.5 to -0.4)*	-0.5 (-0.6 to -0.3)*
		RII	1.4 (1.3 to 1.5)*	1.2 (1.2 to 1.3)*	1.2 (1.1 to 1.2)*	-0.7 (-0.9 to -0.3)*	-0.6 (-0.7 to -0.4)*	-0.6 (-0.9 to -0.4)*
	Namibia	SII	11.7 (8.3 to 15.0)*	0.7 (-1.7 to 4.5)	-3.0 (-5.4 to 0.4)	-0.5 (-0.7 to -0.4)*	-0.4 (-0.5 to -0.2)*	-0.5 (-0.6 to -0.4)*
		RII	1.2 (1.1 to 1.3)*	1.0 (1.0 to 1.1)	1.0 (0.9 to 1.0)	-0.8 (-1.0 to -0.6)*	-0.5 (-0.6 to -0.3)*	-0.7 (-0.8 to -0.6)*
	Niger	SII	25.3 (19.4 to 32.2)*	10.6 (7.9 to 14.0)*	2.1 (1.2 to 3.6)*	-0.7 (-0.9 to -0.5)*	-0.8 (-1.0 to -0.7)*	-0.8 (-1.0 to -0.6)*
		RII	2.8 (2.4 to 3.4)*	1.1 (1.1 to 1.2)*	1.0 (1.0 to 1.0)*	-4.4 (-5.3 to -3.7)*	-1.0 (-1.3 to -0.8)*	-3.3 (-3.9 to -2.8)*
	Nigeria	SII	38.3 (36.3 to 40.9)*	41.2 (39.8 to 42.6)*	35.1 (32.9 to 37.7)*	0.1 (0.0 to 0.3)	-0.6 (-0.7 to -0.5)*	-0.1 (-0.2 to 0.0)*
		RII	5.6 (5.2 to 6.1)*	2.6 (2.5 to 2.7)*	1.9 (1.8 to 2.0)*	-3.8 (-4.2 to -3.3)*	-3.2 (-3.6 to -2.9)*	-3.6 (-4.0 to -3.2)*
	Rwanda	SII	12.1 (8.8 to 15.8)*	0.9 (-0.5 to 2.2)	-0.5 (-1.6 to 0.3)	-0.6 (-0.7 to -0.4)*	-0.1 (-0.2 to -0.1)*	-0.4 (-0.5 to -0.3)*
		RII	1.2 (1.1 to 1.2)*	1.0 (1.0 to 1.0)	1.0 (1.0 to 1.0)	-0.8 (-1.0 to -0.6)*	-0.2 (-0.2 to -0.1)*	-0.6 (-0.7 to -0.4)*
	Sao Tome and Principe	SII	8.0 (-2.9 to 19.8)	-0.8 (-7.7 to 5.4)	-1.4 (-14.5 to 1.8)	-0.4 (-0.8 to -0.1)*	-0.1 (-0.6 to 0.2)	-0.4 (-0.6 to 0.0)
		RII	1.1 (1.0 to 1.4)	1.0 (0.9 to 1.1)	1.0 (0.8 to 1.0)	-0.6 (-1.4 to 0.0)*	-0.1 (-1.2 to 0.2)	-0.5 (-1.0 to 0.1)
	Senegal	SII	31.1 (27.4 to 34.4)*	10.1 (8.5 to 11.8)*	4.5 (3.4 to 5.9)*	-1.0 (-1.2 to -0.9)*	-0.6 (-0.6 to -0.5)*	-0.9 (-1.0 to -0.8)*
		RII	1.6 (1.5 to 1.7)*	1.1 (1.1 to 1.1)*	1.0 (1.0 to 1.1)*	-1.7 (-2.0 to -1.5)*	-0.7 (-0.7 to -0.6)*	-1.4 (-1.6 to -1.2)*
	Sierra Leone	SII	18.2 (14.8 to 22.7)*	5.0 (2.2 to 7.7)*	-0.1 (-2.7 to 2.3)	-0.7 (-0.8 to -0.6)*	-0.5 (-0.6 to -0.4)*	-0.6 (-0.7 to -0.5)*
		RII	1.4 (1.3 to 1.5)*	1.1 (1.0 to 1.1)*	1.0 (1.0 to 1.0)	-1.4 (-1.7 to -1.1)*	-0.7 (-0.9 to -0.6)*	-1.2 (-1.4 to -1.0)*
	South Africa	SII	19.4 (8.6 to 29.4)*	9.8 (-0.2 to 20.0)	3.2 (-6.1 to 12.1)	-0.5 (-0.7 to 0.0)	-0.6 (-1.0 to -0.5)*	-0.5 (-0.7 to -0.2)*
		RII	1.6 (1.1 to 2.6)*	1.2 (1.0 to 1.6)	1.1 (0.9 to 1.5)	-1.5 (-3.4 to 0.8)	-1.2 (-1.8 to -0.5)*	-1.3 (-2.9 to 0.2)
	Tanzania	SII	16.1 (13.5 to 18.5)*	7.2 (4.9 to 9.5)*	3.7 (1.2 to 6.2)*	-0.4 (-0.6 to -0.3)*	-0.4 (-0.4 to -0.3)*	-0.4 (-0.5 to -0.3)*
		RII	1.2 (1.2 to 1.3)*	1.1 (1.1 to 1.1)*	1.0 (1.0 to 1.1)*	-0.6 (-0.7 to -0.4)*	-0.4 (-0.5 to -0.4)*	-0.5 (-0.7 to -0.4)*

Indicators	Country	Index	Estimates of Inequalities			Changes in Inequalities		
			Year 2000	Year 2020	Year 2030	2000–2020	2020–2030	2000–2030
	Togo	SII	24.9 (14.6 to 31.0)*	9.3 (5.0 to 16.2)*	3.5 (0.7 to 13.1)*	-0.8 (-1.1 to -0.1)*	-0.5 (-0.7 to -0.4)*	-0.7 (-0.9 to -0.2)*
		RII	1.6 (1.2 to 2.2)*	1.1 (1.1 to 1.3)*	1.0 (1.0 to 1.2)*	-1.6 (-3.5 to 0.0)	-0.7 (-0.8 to -0.4)*	-1.3 (-2.6 to -0.1)*
	Uganda	SII	19.6 (16.8 to 22.6)*	5.8 (3.4 to 8.1)*	0.6 (-1.8 to 2.9)	-0.7 (-0.8 to -0.6)*	-0.5 (-0.6 to -0.4)*	-0.6 (-0.7 to -0.5)*
		RII	1.5 (1.4 to 1.6)*	1.1 (1.0 to 1.1)*	1.0 (1.0 to 1.0)	-1.7 (-2.0 to -1.5)*	-0.7 (-0.8 to -0.6)*	-1.4 (-1.6 to -1.2)*
	Zambia	SII	18.8 (16.3 to 21.2)*	7.1 (5.3 to 9.3)*	3.0 (0.9 to 5.1)*	-0.6 (-0.7 to -0.5)*	-0.4 (-0.5 to -0.4)*	-0.5 (-0.6 to -0.4)*
		RII	1.3 (1.2 to 1.3)*	1.1 (1.1 to 1.1)*	1.0 (1.0 to 1.1)*	-0.8 (-1.0 to -0.7)*	-0.5 (-0.6 to -0.5)*	-0.7 (-0.9 to -0.6)*
	Zimbabwe	SII	17.9 (14.1 to 21.3)*	3.5 (1.5 to 5.6)*	0.0 (-1.1 to 1.1)	-0.7 (-0.9 to -0.6)*	-0.4 (-0.5 to -0.2)*	-0.6 (-0.7 to -0.5)*
		RII	1.6 (1.4 to 1.7)*	1.0 (1.0 to 1.1)*	1.0 (1.0 to 1.0)	-2.0 (-2.4 to -1.6)*	-0.4 (-0.6 to -0.3)*	-1.5 (-1.8 to -1.2)*
	Angola	SII	25.4 (10.1 to 36.1)*	19.5 (13.1 to 25.1)*	16.2 (3.8 to 26.7)*	-0.3 (-1.0 to 0.7)	-0.3 (-0.9 to 0.2)	-0.3 (-0.9 to 0.5)
		RII	1.4 (1.1 to 2.6)*	1.3 (1.2 to 1.4)*	1.2 (1.0 to 1.7)*	-0.4 (-4.0 to 1.2)	-0.4 (-1.1 to 1.6)	-0.4 (-3.1 to 1.3)
	Benin	SII	9.6 (8.4 to 11.0)*	10.9 (9.1 to 12.8)*	11.5 (8.5 to 14.8)*	0.1 (-0.1 to 0.2)	0.0 (-0.1 to 0.2)	0.1 (-0.1 to 0.2)
		RII	1.1 (1.1 to 1.1)*	1.1 (1.1 to 1.2)*	1.1 (1.1 to 1.2)*	0.1 (0.0 to 0.2)	0.1 (-0.1 to 0.3)	0.1 (-0.1 to 0.2)
	Burkina Faso	SII	24.4 (16.9 to 29.7)*	0.2 (0.1 to 0.4)*	0.0 (0.0 to 0.0)*	-1.2 (-1.5 to -0.8)*	0.0 (0.0 to 0.0)*	-0.8 (-1.0 to -0.6)*
		RII	1.4 (1.2 to 1.5)*	1.0 (1.0 to 1.0)*	1.0 (1.0 to 1.0)*	-1.5 (-1.9 to -1.1)*	0.0 (0.0 to 0.0)*	-1.0 (-1.3 to -0.7)*
	Burundi	SII	0.7 (0.3 to 1.9)*	1.4 (0.3 to 2.8)*	1.6 (-0.2 to 7.1)	0.0 (0.0 to 0.1)	0.0 (0.0 to 0.4)	0.0 (0.0 to 0.2)
		RII	1.0 (1.0 to 1.0)*	1.0 (1.0 to 1.0)*	1.0 (1.0 to 1.1)	0.0 (0.0 to 0.1)	0.0 (0.0 to 0.4)	0.0 (0.0 to 0.2)
	Cameroon	SII	14.1 (11.9 to 16.4)*	14.1 (11.8 to 16.3)*	13.8 (10.3 to 17.1)*	0.0 (-0.2 to 0.2)	0.0 (-0.2 to 0.1)	0.0 (-0.2 to 0.2)
		RII	1.2 (1.1 to 1.2)*	1.2 (1.1 to 1.2)*	1.2 (1.1 to 1.2)*	0.0 (-0.2 to 0.2)	0.0 (-0.2 to 0.2)	0.0 (-0.2 to 0.2)
	Chad	SII	36.0 (29.2 to 41.5)*	20.2 (16.9 to 23.8)*	11.6 (8.4 to 15.1)*	-0.8 (-1.0 to -0.6)*	-0.9 (-1.0 to -0.7)*	-0.8 (-1.0 to -0.6)*
		RII	2.1 (1.9 to 2.4)*	1.3 (1.2 to 1.4)*	1.1 (1.1 to 1.2)*	-2.4 (-2.9 to -1.9)*	-1.3 (-1.4 to -1.1)*	-2.0 (-2.4 to -1.6)*
	Comoros	SII	12.4 (4.1 to 23.4)*	3.3 (-0.1 to 9.1)	1.1 (-0.6 to 8.3)	-0.5 (-1.0 to 0.1)	-0.2 (-0.3 to 0.0)	-0.4 (-0.7 to 0.1)
		RII	1.2 (1.0 to 1.5)*	1.0 (1.0 to 1.1)	1.0 (1.0 to 1.1)	-0.7 (-2.0 to 0.1)	-0.2 (-0.4 to 0.1)	-0.5 (-1.4 to 0.1)
	Congo	SII	15.1 (10.5 to 20.8)*	3.2 (1.7 to 5.6)*	1.2 (0.5 to 3.8)*	-0.6 (-0.9 to -0.2)*	-0.2 (-0.2 to -0.1)*	-0.5 (-0.7 to -0.2)*
	Brazzaville	RII	1.2 (1.1 to 1.3)*	1.0 (1.0 to 1.1)*	1.0 (1.0 to 1.0)*	-0.7 (-1.1 to -0.3)*	-0.2 (-0.2 to -0.1)*	-0.5 (-0.8 to -0.3)*
	Congo	SII	29.0 (25.3 to 33.8)*	6.6 (5.1 to 8.3)*	2.1 (1.2 to 3.3)*	-1.1 (-1.4 to -0.9)*	-0.4 (-0.5 to -0.4)*	-0.9 (-1.1 to -0.8)*
	Democratic Republic	RII	1.6 (1.5 to 1.8)*	1.1 (1.1 to 1.1)*	1.0 (1.0 to 1.0)*	-2.1 (-2.6 to -1.7)*	-0.5 (-0.6 to -0.4)*	-1.6 (-1.9 to -1.3)*
	Cote d'Ivoire	SII	20.8 (9.1 to 36.2)*	6.9 (2.8 to 17.3)*	3.5 (0.5 to 20.2)*	-0.7 (-1.6 to 0.4)	-0.3 (-0.4 to 0.3)	-0.6 (-1.2 to 0.4)
		RII	1.3 (1.1 to 1.8)*	1.1 (1.0 to 1.2)*	1.0 (1.0 to 1.3)*	-0.9 (-2.9 to 0.5)	-0.3 (-0.5 to 0.6)	-0.8 (-2.0 to 0.5)
	Egypt	SII	0.7 (0.3 to 1.2)*	0.4 (-0.1 to 0.9)	0.2 (-0.4 to 0.9)	0.0 (0.0 to 0.0)	0.0 (0.0 to 0.0)*	0.0 (0.0 to 0.0)
		RII	1.0 (1.0 to 1.0)*	1.0 (1.0 to 1.0)	1.0 (1.0 to 1.0)	0.0 (0.0 to 0.0)	0.0 (0.0 to 0.0)*	0.0 (0.0 to 0.0)
	Eswatini	SII	3.3 (-0.1 to 8.9)	0.4 (-0.2 to 4.7)	0.1 (-0.3 to 5.2)	-0.1 (-0.4 to 0.1)	0.0 (-0.1 to 0.1)	-0.1 (-0.3 to 0.1)
		RII	1.0 (1.0 to 1.1)	1.0 (1.0 to 1.1)	1.0 (1.0 to 1.1)	-0.1 (-0.5 to 0.1)	0.0 (-0.1 to 0.1)	-0.1 (-0.3 to 0.1)
	Ethiopia	SII	37.2 (34.4 to 40.0)*	19.0 (16.7 to 20.9)*	12.0 (9.9 to 14.0)*	-0.9 (-1.0 to -0.8)*	-0.7 (-0.8 to -0.6)*	-0.8 (-0.9 to -0.7)*
		RII	1.8 (1.7 to 1.9)*	1.3 (1.2 to 1.3)*	1.1 (1.1 to 1.2)*	-1.7 (-1.9 to -1.5)*	-1.0 (-1.0 to -0.9)*	-1.5 (-1.6 to -1.3)*
	Gabon	SII	9.0 (6.5 to 11.8)*	4.7 (2.6 to 7.9)*	3.2 (1.0 to 6.7)*	-0.2 (-0.4 to -0.1)*	-0.2 (-0.2 to -0.1)*	-0.2 (-0.3 to -0.1)*
		RII	1.1 (1.1 to 1.1)*	1.1 (1.0 to 1.1)*	1.0 (1.0 to 1.1)*	-0.2 (-0.4 to -0.1)*	-0.2 (-0.2 to -0.1)*	-0.2 (-0.4 to -0.1)*
	Gambia	SII	1.9 (0.5 to 6.0)*	0.5 (0.0 to 1.0)*	0.2 (0.0 to 0.9)	-0.1 (-0.3 to 0.0)*	0.0 (0.0 to 0.0)*	-0.1 (-0.2 to 0.0)*
		RII	1.0 (1.0 to 1.1)*	1.0 (1.0 to 1.0)*	1.0 (1.0 to 1.0)	-0.1 (-0.3 to 0.0)*	0.0 (0.0 to 0.0)*	-0.1 (-0.2 to 0.0)*
BCG immunization	Ghana	SII	8.8 (6.0 to 12.2)*	1.3 (0.6 to 2.4)*	0.4 (0.1 to 1.1)*	-0.4 (-0.6 to -0.2)*	-0.1 (-0.1 to 0.0)*	-0.3 (-0.4 to -0.2)*
		RII	1.1 (1.1 to 1.2)*	1.0 (1.0 to 1.0)*	1.0 (1.0 to 1.0)*	-0.4 (-0.6 to -0.3)*	-0.1 (-0.1 to -0.1)*	-0.3 (-0.5 to -0.2)*
	Guinea	SII	12.5 (10.4 to 15.4)*	17.2 (13.8 to 20.3)*	18.4 (13.7 to 23.9)*	0.2 (0.1 to 0.4)*	0.1 (-0.1 to 0.4)	0.2 (0.0 to 0.4)*
		RII	1.2 (1.1 to 1.2)*	1.2 (1.2 to 1.3)*	1.3 (1.2 to 1.4)*	0.4 (0.1 to 0.6)*	0.3 (0.1 to 0.8)*	0.3 (0.1 to 0.7)*
	Kenya	SII	11.5 (8.5 to 14.8)*	1.1 (0.7 to 1.6)*	0.3 (0.1 to 0.5)*	-0.5 (-0.7 to -0.4)*	-0.1 (-0.1 to -0.1)*	-0.4 (-0.5 to -0.3)*
		RII	1.1 (1.1 to 1.2)*	1.0 (1.0 to 1.0)*	1.0 (1.0 to 1.0)*	-0.6 (-0.8 to -0.4)*	-0.1 (-0.1 to -0.1)*	-0.4 (-0.6 to -0.3)*
	Lesotho	SII	4.6 (2.7 to 7.3)*	1.8 (0.8 to 3.6)*	1.0 (0.3 to 3.3)*	-0.1 (-0.3 to 0.0)*	-0.1 (-0.1 to 0.0)*	-0.1 (-0.2 to 0.0)*
		RII	1.0 (1.0 to 1.1)*	1.0 (1.0 to 1.0)*	1.0 (1.0 to 1.0)*	-0.2 (-0.3 to 0.0)*	-0.1 (-0.1 to 0.0)*	-0.1 (-0.2 to 0.0)*
	Liberia	SII	22.0 (17.1 to 26.9)*	4.0 (2.9 to 5.3)*	1.3 (0.8 to 2.3)*	-0.9 (-1.1 to -0.7)*	-0.3 (-0.3 to -0.2)*	-0.7 (-0.8 to -0.5)*
		RII	1.3 (1.3 to 1.5)*	1.0 (1.0 to 1.1)*	1.0 (1.0 to 1.0)*	-1.3 (-1.6 to -0.9)*	-0.3 (-0.3 to -0.2)*	-0.9 (-1.2 to -0.7)*
	Madagascar	SII	34.2 (28.8 to 39.4)*	3.8 (2.2 to 6.5)*	1.0 (0.4 to 2.4)*	-1.5 (-1.9 to -1.2)*	-0.3 (-0.4 to -0.2)*	-1.1 (-1.3 to -0.9)*
		RII	1.6 (1.5 to 1.8)*	1.0 (1.0 to 1.1)*	1.0 (1.0 to 1.0)*	-2.2 (-2.7 to -1.7)*	-0.3 (-0.4 to -0.2)*	-1.5 (-1.9 to -1.2)*
	Malawi	SII	6.9 (5.5 to 8.2)*	1.2 (0.8 to 1.7)*	0.5 (0.3 to 0.8)*	-0.3 (-0.3 to -0.2)*	-0.1 (-0.1 to -0.1)*	-0.2 (-0.3 to -0.2)*
		RII	1.1 (1.1 to 1.1)*	1.0 (1.0 to 1.0)*	1.0 (1.0 to 1.0)*	-0.3 (-0.4 to -0.2)*	-0.1 (-0.1 to -0.1)*	-0.2 (-0.3 to -0.2)*
	Mali	SII	24.2 (21.0 to 26.3)*	11.2 (9.5 to 13.2)*	7.1 (5.4 to 9.1)*	-0.6 (-0.8 to -0.5)*	-0.4 (-0.4 to -0.4)*	-0.6 (-0.6 to -0.5)*
		RII	1.4 (1.3 to 1.4)*	1.1 (1.1 to 1.2)*	1.1 (1.1 to 1.1)*	-0.9 (-1.0 to -0.7)*	-0.5 (-0.5 to -0.4)*	-0.7 (-0.9 to -0.6)*
	Morocco	SII	1.8 (0.7 to 3.5)*	0.4 (0.0 to 2.8)*	0.1 (0.0 to 3.5)*	-0.1 (-0.2 to 0.1)	0.0 (0.0 to 0.1)	-0.1 (-0.1 to 0.1)
		RII	1.0 (1.0 to 1.0)*	1.0 (1.0 to 1.0)*	1.0 (1.0 to 1.0)*	-0.1 (-0.2 to 0.1)	0.0 (0.0 to 0.1)	-0.1 (-0.1 to 0.1)
	Mozambique	SII	8.8 (5.8 to 11.2)*	3.1 (1.6 to 5.0)*	1.8 (0.4 to 3.5)*	-0.3 (-0.4 to -0.1)*	-0.1 (-0.2 to -0.1)*	-0.2 (-0.3 to -0.1)*
		RII	1.1 (1.1 to 1.1)*	1.0 (1.0 to 1.1)*	1.0 (1.0 to 1.0)*	-0.3 (-0.5 to -0.2)*	-0.2 (-0.2 to -0.1)*	-0.3 (-0.4 to -0.2)*
	Namibia	SII	5.8 (3.6 to 8.6)*	1.8 (0.6 to 3.4)*	0.8 (-0.2 to 2.3)	-0.2 (-0.3 to -0.1)*	-0.1 (-0.1 to -0.1)*	-0.2 (-0.2 to -0.1)*
		RII	1.1 (1.0 to 1.1)*	1.0 (1.0 to 1.0)*	1.0 (1.0 to 1.0)	-0.2 (-0.3 to -0.1)*	-0.1 (-0.2 to -0.1)*	-0.2 (-0.3 to -0.1)*
	Niger	SII	43.3 (34.8 to 49.2)*	3.8 (2.8 to 5.0)*	0.7 (0.4 to 1.1)*	-2.0 (-2.3 to -1.6)*	-0.3 (-0.4 to -0.2)*	-1.4 (-1.6 to -1.1)*
		RII	2.2 (1.9 to 2.4)*	1.0 (1.0 to 1.1)*	1.0 (1.0 to 1.0)*	-3.6 (-4.2 to -3.0)*	-0.3 (-0.4 to -0.2)*	-2.5 (-2.9 to -2.1)*
	Nigeria	SII	57.3 (55.1 to 59.5)*	42.0 (40.1 to 43.6)*	31.2 (27.9 to 34.2)*	-0.8 (-0.9 to -0.6)*	-1.1 (-1.2 to -0.9)*	-0.9 (-1.0 to -0.7)*
		RII	3.5 (3.2 to 3.7)*	1.9 (1.8 to 1.9)*	1.5 (1.4 to 1.6)*	-3.0 (-3.5 to -2.6)*	-2.2 (-2.3 to -2.0)*	-2.7 (-3.1 to -2.4)*
	Rwanda	SII	2.3 (0.9 to 3.5)*	0.4 (0.0 to 0.9)	0.1 (-0.2 to 0.5)	-0.1 (-0.1 to 0.0)*	0.0 (0.0 to 0.0)*	-0.1 (-0.1 to 0.0)*
		RII	1.0 (1.0 to 1.0)*	1.0 (1.0 to 1.0)	1.0 (1.0 to 1.0)	-0.1 (-0.1 to 0.0)*	0.0 (0.0 to 0.0)*	-0.1 (-0.1 to 0.0)*
	Sao Tome	SII	6.7 (2.4 to 15.3)*	1.1 (0.1 to 5.5)*	0.4 (0.0 to 5.8)*	-0.3 (-0.8 to 0.1)	-0.1 (-0.1 to 0.1)	-0.2 (-0.5 to 0.1)
	and Principe	RII	1.1 (1.0 to 1.2)*	1.0 (1.0 to 1.1)*	1.0 (1.0 to 1.1)*	-0.3 (-0.9 to 0.1)	-0.1 (-0.1 to 0.1)	-0.2 (-0.6 to 0.1)
	Senegal	SII	8.1 (6.4 to 10.1)*	3.2 (2.4 to 4.0)*	1.9 (1.3 to 3.0)*	-0.2 (-0.4 to -0.1)*	-0.1 (-0.1 to -0.1)*	-0.2 (-0.3 to -0.1)*
		RII	1.1 (1.1 to 1.1)*	1.0 (1.0 to 1.0)*	1.0 (1.0 to 1.0)*	-0.3 (-0.4 to -0.1)*	-0.1 (-0.2 to -0.1)*	-0.2 (-0.3 to -0.1)*

Indicators	Country	Index	Estimates of Inequalities			Changes in Inequalities		
			Year 2000	Year 2020	Year 2030	2000–2020	2020–2030	2000–2030
	Sierra Leone	SII	19.0 (11.9 to 25.4)*	1.1 (0.5 to 1.8)*	0.2 (0.0 to 0.4)*	-0.9 (-1.2 to -0.6)*	-0.1 (-0.1 to 0.0)*	-0.6 (-0.8 to -0.4)*
		RII	1.3 (1.2 to 1.5)*	1.0 (1.0 to 1.0)*	1.0 (1.0 to 1.0)*	-1.3 (-1.9 to -0.9)*	-0.1 (-0.1 to 0.0)*	-0.9 (-1.3 to -0.6)*
	South Africa	SII	9.7 (1.2 to 26.7)*	2.7 (-1.0 to 9.0)	0.9 (-1.5 to 8.1)	-0.4 (-1.0 to 0.1)	-0.1 (-0.4 to 0.1)	-0.3 (-0.8 to 0.1)
		RII	1.1 (1.0 to 1.6)*	1.0 (1.0 to 1.1)	1.0 (1.0 to 1.1)	-0.5 (-1.9 to 0.2)	-0.2 (-0.5 to 0.2)	-0.4 (-1.4 to 0.2)
	Tanzania	SII	6.3 (3.9 to 9.1)*	1.1 (0.4 to 2.0)*	0.4 (0.0 to 1.0)	-0.3 (-0.4 to -0.2)*	-0.1 (-0.1 to 0.0)*	-0.2 (-0.3 to -0.1)*
		RII	1.1 (1.0 to 1.1)*	1.0 (1.0 to 1.0)*	1.0 (1.0 to 1.0)	-0.3 (-0.4 to -0.2)*	-0.1 (-0.1 to 0.0)*	-0.2 (-0.3 to -0.1)*
	Togo	SII	13.1 (3.7 to 30.0)*	2.3 (0.8 to 5.3)*	0.8 (0.1 to 5.8)*	-0.5 (-1.4 to 0.1)	-0.1 (-0.2 to 0.1)	-0.4 (-1.0 to 0.1)
		RII	1.2 (1.0 to 1.5)*	1.0 (1.0 to 1.1)*	1.0 (1.0 to 1.1)*	-0.6 (-2.1 to 0.1)	-0.1 (-0.2 to 0.1)	-0.5 (-1.4 to 0.1)
	Uganda	SII	11.4 (7.9 to 14.4)*	0.9 (0.5 to 1.4)*	0.2 (0.0 to 0.4)*	-0.5 (-0.7 to -0.4)*	-0.1 (-0.1 to 0.0)*	-0.4 (-0.5 to -0.3)*
		RII	1.1 (1.1 to 1.2)*	1.0 (1.0 to 1.0)*	1.0 (1.0 to 1.0)*	-0.6 (-0.8 to -0.4)*	-0.1 (-0.1 to 0.0)*	-0.5 (-0.6 to -0.3)*
	Zambia	SII	5.4 (4.1 to 6.9)*	2.0 (1.2 to 2.9)*	1.1 (0.4 to 1.9)*	-0.2 (-0.2 to -0.1)*	-0.1 (-0.1 to -0.1)*	-0.1 (-0.2 to -0.1)*
		RII	1.1 (1.0 to 1.1)*	1.0 (1.0 to 1.0)*	1.0 (1.0 to 1.0)*	-0.2 (-0.3 to -0.1)*	-0.1 (-0.1 to -0.1)*	-0.1 (-0.2 to -0.1)*
	Zimbabwe	SII	10.6 (6.7 to 15.1)*	0.7 (-0.2 to 2.1)	0.0 (-0.6 to 0.7)	-0.5 (-0.7 to -0.3)*	-0.1 (-0.2 to 0.0)*	-0.4 (-0.5 to -0.2)*
		RII	1.2 (1.1 to 1.3)*	1.0 (1.0 to 1.0)	1.0 (1.0 to 1.0)	-0.8 (-1.1 to -0.5)*	-0.1 (-0.2 to 0.0)*	-0.6 (-0.8 to -0.3)*
MCV1 immunization	Angola	SII	33.2 (13.8 to 44.8)*	32.5 (27.0 to 37.1)*	27.2 (14.6 to 33.1)*	0.0 (-0.7 to 1.0)	-0.5 (-1.5 to -0.2)*	-0.1 (-0.8 to 0.2)
		RII	1.6 (1.2 to 3.0)*	1.8 (1.5 to 2.1)*	1.8 (1.2 to 2.7)*	0.5 (-3.3 to 3.0)	0.2 (-2.0 to 2.4)	0.4 (-2.9 to 2.8)
	Benin	SII	24.0 (21.8 to 26.5)*	16.7 (13.8 to 19.4)*	12.8 (9.4 to 16.6)*	-0.4 (-0.5 to -0.2)*	-0.4 (-0.5 to -0.3)*	-0.4 (-0.5 to -0.3)*
		RII	1.4 (1.3 to 1.4)*	1.3 (1.2 to 1.3)*	1.2 (1.1 to 1.2)*	-0.5 (-0.7 to -0.3)*	-0.5 (-0.7 to -0.4)*	-0.5 (-0.7 to -0.4)*
	Burkina Faso	SII	30.3 (21.2 to 38.0)*	0.8 (0.5 to 1.3)*	0.1 (0.0 to 0.1)*	-1.5 (-1.8 to -1.0)*	-0.1 (-0.1 to 0.0)*	-1.0 (-1.3 to -0.7)*
		RII	1.8 (1.5 to 2.0)*	1.0 (1.0 to 1.0)*	1.0 (1.0 to 1.0)*	-2.8 (-3.4 to -2.1)*	-0.1 (-0.1 to 0.0)*	-1.9 (-2.3 to -1.4)*
	Burundi	SII	3.5 (1.9 to 6.3)*	2.6 (0.8 to 4.1)*	1.8 (-0.3 to 4.5)	-0.1 (-0.2 to 0.1)	-0.1 (-0.1 to 0.0)	-0.1 (-0.2 to 0.1)
		RII	1.0 (1.0 to 1.1)*	1.0 (1.0 to 1.0)*	1.0 (1.0 to 1.0)	-0.1 (-0.2 to 0.1)	-0.1 (-0.1 to 0.0)	-0.1 (-0.2 to 0.1)
	Cameroon	SII	24.1 (21.2 to 26.7)*	19.4 (16.3 to 21.9)*	15.9 (11.7 to 19.5)*	-0.2 (-0.4 to -0.1)*	-0.3 (-0.5 to -0.2)*	-0.3 (-0.4 to -0.1)*
		RII	1.4 (1.3 to 1.5)*	1.3 (1.3 to 1.4)*	1.3 (1.2 to 1.4)*	-0.2 (-0.5 to 0.1)	-0.4 (-0.6 to -0.1)*	-0.3 (-0.6 to 0.0)
	Chad	SII	22.0 (16.8 to 27.7)*	13.1 (9.5 to 15.7)*	3.6 (2.0 to 5.2)*	-0.5 (-0.7 to -0.3)*	-1.0 (-1.1 to -0.7)*	-0.6 (-0.8 to -0.5)*
		RII	2.6 (2.2 to 3.1)*	1.2 (1.1 to 1.2)*	1.0 (1.0 to 1.1)*	-3.9 (-4.6 to -3.2)*	-1.2 (-1.4 to -1.0)*	-3.0 (-3.6 to -2.5)*
	Comoros	SII	14.1 (6.4 to 22.1)*	3.8 (-1.1 to 10.8)	0.6 (-3.8 to 7.5)	-0.5 (-0.9 to 0.0)	-0.3 (-0.5 to -0.1)*	-0.4 (-0.7 to -0.1)*
		RII	1.3 (1.1 to 1.7)*	1.0 (1.0 to 1.2)	1.0 (0.9 to 1.1)	-0.8 (-2.5 to 0.1)	-0.4 (-0.7 to -0.1)*	-0.7 (-1.7 to 0.0)*
	Congo Brazzaville	SII	26.7 (22.7 to 30.4)*	10.1 (7.1 to 13.8)*	4.6 (2.3 to 8.4)*	-0.8 (-1.0 to -0.6)*	-0.5 (-0.6 to -0.5)*	-0.7 (-0.9 to -0.6)*
		RII	1.6 (1.5 to 1.8)*	1.1 (1.1 to 1.2)*	1.1 (1.0 to 1.1)*	-1.7 (-2.2 to -1.2)*	-0.7 (-0.8 to -0.6)*	-1.4 (-1.7 to -1.0)*
	Congo Democratic Republic	SII	25.9 (22.3 to 29.5)*	10.5 (8.0 to 13.0)*	5.0 (2.8 to 7.7)*	-0.8 (-0.9 to -0.6)*	-0.6 (-0.6 to -0.5)*	-0.7 (-0.8 to -0.5)*
		RII	1.6 (1.5 to 1.7)*	1.1 (1.1 to 1.2)*	1.1 (1.0 to 1.1)*	-1.7 (-2.1 to -1.1)*	-0.7 (-0.8 to -0.6)*	-1.4 (-1.6 to -1.0)*
	Cote d'Ivoire	SII	28.5 (14.1 to 38.6)*	17.5 (9.2 to 28.9)*	12.4 (2.8 to 27.3)*	-0.6 (-1.3 to 0.6)	-0.5 (-0.9 to -0.1)*	-0.6 (-1.1 to 0.2)
		RII	1.5 (1.2 to 2.2)*	1.3 (1.1 to 1.6)*	1.2 (1.0 to 1.8)*	-1.0 (-3.2 to 1.5)	-0.7 (-1.0 to 1.0)	-0.9 (-2.4 to 1.4)
	Egypt	SII	1.2 (0.9 to 1.7)*	3.4 (0.4 to 7.1)*	0.0 (-5.0 to 5.7)	0.1 (0.0 to 0.3)	-0.3 (-0.6 to -0.1)*	0.0 (-0.2 to 0.1)
		RII	1.0 (1.0 to 1.0)*	1.0 (1.0 to 1.1)*	1.0 (0.9 to 1.1)	0.2 (0.0 to 0.4)	-0.4 (-1.1 to 0.3)	0.0 (-0.3 to 0.3)
	Eswatini	SII	8.6 (3.7 to 16.7)*	1.8 (0.1 to 10.1)*	0.6 (-0.7 to 9.6)	-0.3 (-0.8 to 0.1)	-0.1 (-0.3 to 0.0)*	-0.2 (-0.5 to 0.1)
		RII	1.1 (1.0 to 1.2)*	1.0 (1.0 to 1.1)*	1.0 (1.0 to 1.1)	-0.4 (-1.0 to 0.2)	-0.1 (-0.3 to 0.1)	-0.3 (-0.7 to 0.2)
	Ethiopia	SII	37.6 (33.4 to 41.0)*	22.5 (19.9 to 25.1)*	13.1 (10.9 to 15.7)*	-0.7 (-0.9 to -0.6)*	-0.9 (-1.0 to -0.8)*	-0.8 (-0.9 to -0.7)*
		RII	2.4 (2.2 to 2.5)*	1.4 (1.3 to 1.4)*	1.2 (1.1 to 1.2)*	-2.7 (-3.0 to -2.4)*	-1.5 (-1.6 to -1.4)*	-2.3 (-2.5 to -2.1)*
	Gabon	SII	14.4 (10.6 to 19.6)*	2.7 (0.3 to 5.3)*	-0.1 (-1.9 to 1.7)	-0.6 (-0.8 to -0.4)*	-0.3 (-0.4 to -0.2)*	-0.5 (-0.6 to -0.4)*
		RII	1.3 (1.2 to 1.4)*	1.0 (1.0 to 1.1)*	1.0 (1.0 to 1.0)	-1.2 (-1.6 to -0.9)*	-0.3 (-0.5 to -0.2)*	-0.9 (-1.2 to -0.7)*
	Gambia	SII	1.9 (0.9 to 3.8)*	1.4 (-1.1 to 3.3)	-0.7 (-4.4 to 2.1)	0.0 (-0.1 to 0.1)	-0.2 (-0.4 to -0.1)*	-0.1 (-0.2 to 0.0)
		RII	1.0 (1.0 to 1.0)*	1.0 (1.0 to 1.0)	1.0 (0.9 to 1.0)	0.0 (-0.1 to 0.1)	-0.2 (-0.5 to 0.0)*	-0.1 (-0.2 to 0.0)
	Ghana	SII	12.1 (9.0 to 15.6)*	3.4 (1.6 to 6.3)*	1.4 (0.2 to 4.0)*	-0.4 (-0.6 to -0.3)*	-0.2 (-0.3 to -0.2)*	-0.4 (-0.5 to -0.3)*
		RII	1.2 (1.1 to 1.2)*	1.0 (1.0 to 1.1)*	1.0 (1.0 to 1.0)*	-0.5 (-0.7 to -0.3)*	-0.2 (-0.3 to -0.2)*	-0.4 (-0.6 to -0.3)*
	Guinea	SII	21.7 (18.2 to 25.0)*	17.1 (12.1 to 22.5)*	11.2 (6.7 to 16.8)*	-0.2 (-0.4 to -0.1)*	-0.6 (-0.7 to -0.4)*	-0.3 (-0.5 to -0.2)*
		RII	1.4 (1.3 to 1.4)*	1.4 (1.3 to 1.6)*	1.4 (1.2 to 1.6)*	0.2 (-0.2 to 0.6)	-0.4 (-0.9 to 0.1)	0.0 (-0.4 to 0.4)
	Kenya	SII	20.9 (17.6 to 23.9)*	5.7 (4.5 to 7.2)*	2.4 (1.5 to 3.7)*	-0.8 (-0.9 to -0.6)*	-0.3 (-0.4 to -0.3)*	-0.6 (-0.7 to -0.5)*
		RII	1.3 (1.3 to 1.4)*	1.1 (1.0 to 1.1)*	1.0 (1.0 to 1.0)*	-1.1 (-1.3 to -0.9)*	-0.4 (-0.4 to -0.3)*	-0.8 (-1.0 to -0.7)*
	Lesotho	SII	10.8 (7.6 to 14.6)*	3.4 (1.0 to 5.5)*	1.1 (-0.9 to 3.2)	-0.4 (-0.5 to -0.2)*	-0.2 (-0.3 to -0.2)*	-0.3 (-0.4 to -0.2)*
		RII	1.1 (1.1 to 1.2)*	1.0 (1.0 to 1.1)*	1.0 (1.0 to 1.0)	-0.5 (-0.7 to -0.3)*	-0.3 (-0.3 to -0.2)*	-0.4 (-0.6 to -0.3)*
	Liberia	SII	25.0 (20.9 to 29.1)*	13.5 (10.6 to 16.1)*	8.6 (5.8 to 11.9)*	-0.6 (-0.7 to -0.4)*	-0.5 (-0.6 to -0.4)*	-0.6 (-0.7 to -0.4)*
		RII	1.5 (1.4 to 1.6)*	1.2 (1.1 to 1.2)*	1.1 (1.1 to 1.2)*	-1.0 (-1.3 to -0.6)*	-0.7 (-0.8 to -0.5)*	-0.9 (-1.2 to -0.6)*
	Madagascar	SII	34.0 (29.6 to 37.7)*	9.0 (6.1 to 12.1)*	3.3 (1.7 to 5.7)*	-1.2 (-1.5 to -1.0)*	-0.6 (-0.6 to -0.4)*	-1.0 (-1.2 to -0.9)*
		RII	1.8 (1.6 to 1.9)*	1.1 (1.1 to 1.2)*	1.0 (1.0 to 1.1)*	-2.3 (-2.9 to -1.8)*	-0.6 (-0.8 to -0.5)*	-1.7 (-2.1 to -1.4)*
	Malawi	SII	11.5 (9.8 to 13.3)*	2.8 (1.6 to 3.7)*	1.1 (0.4 to 1.8)*	-0.4 (-0.5 to -0.4)*	-0.2 (-0.2 to -0.1)*	-0.3 (-0.4 to -0.3)*
		RII	1.1 (1.1 to 1.2)*	1.0 (1.0 to 1.0)*	1.0 (1.0 to 1.0)*	-0.5 (-0.6 to -0.4)*	-0.2 (-0.2 to -0.1)*	-0.4 (-0.5 to -0.3)*
	Mali	SII	26.1 (23.0 to 28.6)*	12.5 (10.3 to 15.0)*	7.4 (5.4 to 9.9)*	-0.7 (-0.8 to -0.6)*	-0.5 (-0.6 to -0.4)*	-0.6 (-0.7 to -0.5)*
		RII	1.5 (1.4 to 1.5)*	1.2 (1.1 to 1.2)*	1.1 (1.1 to 1.1)*	-1.2 (-1.4 to -1.0)*	-0.7 (-0.8 to -0.6)*	-1.0 (-1.2 to -0.9)*
	Morocco	SII	8.9 (5.3 to 13.1)*	2.3 (0.2 to 13.6)*	1.0 (0.0 to 15.8)*	-0.3 (-0.6 to 0.3)	-0.1 (-0.2 to 0.1)	-0.3 (-0.4 to 0.3)
		RII	1.1 (1.1 to 1.2)*	1.0 (1.0 to 1.2)*	1.0 (1.0 to 1.2)*	-0.4 (-0.7 to 0.4)	-0.1 (-0.2 to 0.4)	-0.3 (-0.5 to 0.4)
	Mozambique	SII	19.1 (15.9 to 21.6)*	11.1 (8.0 to 13.8)*	8.2 (4.7 to 11.4)*	-0.4 (-0.6 to -0.2)*	-0.3 (-0.4 to -0.2)*	-0.4 (-0.5 to -0.2)*
		RII	1.3 (1.2 to 1.3)*	1.1 (1.1 to 1.2)*	1.1 (1.1 to 1.1)*	-0.5 (-0.7 to -0.3)*	-0.4 (-0.4 to -0.3)*	-0.5 (-0.6 to -0.3)*
	Namibia	SII	12.5 (9.7 to 15.6)*	3.2 (1.4 to 5.2)*	1.1 (-0.3 to 2.7)	-0.5 (-0.6 to -0.3)*	-0.2 (-0.3 to -0.1)*	-0.4 (-0.5 to -0.3)*
		RII	1.2 (1.1 to 1.2)*	1.0 (1.0 to 1.1)*	1.0 (1.0 to 1.0)	-0.6 (-0.8 to -0.4)*	-0.2 (-0.3 to -0.2)*	-0.5 (-0.6 to -0.4)*
	Niger	SII	36.6 (29.8 to 44.5)*	8.5 (6.7 to 11.2)*	2.1 (1.2 to 3.4)*	-1.4 (-1.7 to -1.1)*	-0.7 (-0.8 to -0.5)*	-1.1 (-1.4 to -0.9)*
		RII	2.4 (2.0 to 2.8)*	1.1 (1.1 to 1.1)*	1.0 (1.0 to 1.0)*	-3.8 (-4.5 to -3.1)*	-0.7 (-0.9 to -0.6)*	-2.8 (-3.3 to -2.3)*
	Nigeria	SII	44.5 (41.9 to 47.0)*	36.7 (35.0 to 38.5)*	30.1 (27.5 to 33.1)*	-0.4 (-0.5 to -0.3)*	-0.6 (-0.8 to -0.5)*	-0.5 (-0.6 to -0.3)*
		RII	3.2 (3.0 to 3.4)*	2.0 (1.9 to 2.0)*	1.6 (1.5 to 1.7)*	-2.4 (-2.8 to -2.0)*	-1.9 (-2.1 to -1.6)*	-2.2 (-2.6 to -1.8)*

Indicators	Country	Index	Estimates of Inequalities			Changes in Inequalities		
			Year 2000	Year 2020	Year 2030	2000–2020	2020–2030	2000–2030
	Rwanda	SII	10.2 (7.3 to 12.3)*	2.6 (0.7 to 4.3)*	0.6 (-1.2 to 2.2)	-0.4 (-0.5 to -0.3)*	-0.2 (-0.2 to -0.1)*	-0.3 (-0.4 to -0.3)*
		RII	1.1 (1.1 to 1.2)*	1.0 (1.0 to 1.0)*	1.0 (1.0 to 1.0)	-0.5 (-0.6 to -0.4)*	-0.2 (-0.3 to -0.2)*	-0.4 (-0.5 to -0.3)*
	Sao Tome and Principe	SII	10.5 (2.6 to 19.9)*	1.6 (-2.0 to 8.3)	0.2 (-4.8 to 5.8)	-0.4 (-0.9 to -0.1)*	-0.2 (-0.4 to 0.0)	-0.3 (-0.6 to -0.1)*
		RII	1.1 (1.0 to 1.3)*	1.0 (1.0 to 1.1)	1.0 (0.9 to 1.1)	-0.5 (-1.4 to -0.1)*	-0.2 (-0.5 to 0.0)	-0.4 (-1.0 to -0.1)*
	Senegal	SII	23.7 (21.1 to 26.3)*	10.0 (8.6 to 11.6)*	5.9 (4.6 to 7.4)*	-0.7 (-0.8 to -0.6)*	-0.4 (-0.4 to -0.4)*	-0.6 (-0.7 to -0.5)*
		RII	1.3 (1.3 to 1.4)*	1.1 (1.1 to 1.1)*	1.1 (1.1 to 1.1)*	-0.9 (-1.2 to -0.8)*	-0.5 (-0.5 to -0.4)*	-0.8 (-0.9 to -0.7)*
	Sierra Leone	SII	17.0 (13.3 to 21.6)*	6.1 (3.3 to 8.9)*	2.3 (-0.4 to 5.0)	-0.5 (-0.7 to -0.4)*	-0.4 (-0.5 to -0.3)*	-0.5 (-0.6 to -0.4)*
		RII	1.3 (1.2 to 1.4)*	1.1 (1.0 to 1.1)*	1.0 (1.0 to 1.1)	-0.9 (-1.1 to -0.6)*	-0.5 (-0.6 to -0.4)*	-0.8 (-0.9 to -0.6)*
	South Africa	SII	20.6 (4.8 to 34.5)*	11.1 (3.6 to 24.7)*	7.4 (0.5 to 21.4)*	-0.4 (-1.0 to 0.5)	-0.4 (-0.9 to 0.0)*	-0.4 (-0.9 to 0.3)
		RII	1.3 (1.1 to 2.2)*	1.2 (1.0 to 1.4)*	1.1 (1.0 to 1.5)*	-0.7 (-3.0 to 0.9)	-0.5 (-1.1 to 1.0)	-0.6 (-2.3 to 0.9)
	Tanzania	SII	14.5 (11.7 to 17.0)*	6.6 (4.3 to 8.9)*	3.9 (1.7 to 6.6)*	-0.4 (-0.5 to -0.2)*	-0.3 (-0.3 to -0.2)*	-0.4 (-0.5 to -0.2)*
		RII	1.2 (1.1 to 1.2)*	1.1 (1.0 to 1.1)*	1.0 (1.0 to 1.1)*	-0.5 (-0.7 to -0.3)*	-0.3 (-0.3 to -0.2)*	-0.4 (-0.6 to -0.3)*
	Togo	SII	22.8 (11.9 to 32.4)*	10.8 (4.9 to 16.7)*	5.7 (1.0 to 14.5)*	-0.7 (-1.2 to 0.1)	-0.4 (-0.6 to -0.1)*	-0.6 (-1.0 to 0.0)
		RII	1.4 (1.1 to 2.0)*	1.1 (1.1 to 1.3)*	1.1 (1.0 to 1.3)*	-1.1 (-2.9 to 0.3)	-0.5 (-0.7 to 0.0)	-1.0 (-2.2 to 0.2)
	Uganda	SII	19.7 (16.7 to 22.5)*	6.0 (4.2 to 7.6)*	2.2 (0.7 to 3.7)*	-0.7 (-0.8 to -0.6)*	-0.4 (-0.4 to -0.3)*	-0.6 (-0.7 to -0.5)*
		RII	1.4 (1.3 to 1.4)*	1.1 (1.1 to 1.1)*	1.0 (1.0 to 1.0)*	-1.2 (-1.4 to -1.0)*	-0.5 (-0.5 to -0.4)*	-0.9 (-1.1 to -0.8)*
	Zambia	SII	10.2 (8.5 to 12.0)*	4.4 (3.2 to 5.9)*	2.3 (0.6 to 3.8)*	-0.3 (-0.4 to -0.2)*	-0.2 (-0.3 to -0.2)*	-0.3 (-0.3 to -0.2)*
		RII	1.1 (1.1 to 1.2)*	1.1 (1.0 to 1.1)*	1.0 (1.0 to 1.0)*	-0.3 (-0.4 to -0.2)*	-0.2 (-0.3 to -0.2)*	-0.3 (-0.4 to -0.2)*
	Zimbabwe	SII	19.3 (15.9 to 23.7)*	4.8 (2.7 to 6.9)*	1.3 (0.0 to 2.7)	-0.7 (-0.9 to -0.6)*	-0.3 (-0.5 to -0.3)*	-0.6 (-0.7 to -0.5)*
		RII	1.4 (1.3 to 1.5)*	1.1 (1.0 to 1.1)*	1.0 (1.0 to 1.0)	-1.4 (-1.8 to -1.1)*	-0.4 (-0.6 to -0.3)*	-1.1 (-1.4 to -0.9)*
Polio3 immunization	Angola	SII	30.9 (13.0 to 38.2)*	24.1 (19.7 to 28.2)*	16.7 (8.1 to 22.1)*	-0.4 (-0.7 to 0.5)	-0.7 (-1.4 to -0.4)*	-0.4 (-0.7 to -0.1)*
		RII	1.7 (1.2 to 2.6)*	1.6 (1.4 to 1.9)*	1.5 (1.2 to 2.0)*	-0.2 (-2.9 to 2.3)	-0.7 (-1.9 to 0.7)	-0.3 (-2.6 to 1.8)
	Benin	SII	17.6 (15.7 to 19.5)*	14.5 (12.2 to 17.3)*	12.2 (8.7 to 16.0)*	-0.2 (-0.3 to 0.0)*	-0.2 (-0.4 to -0.1)*	-0.2 (-0.3 to 0.0)*
		RII	1.2 (1.2 to 1.3)*	1.2 (1.2 to 1.2)*	1.2 (1.1 to 1.2)*	-0.2 (-0.3 to 0.0)	-0.3 (-0.4 to -0.1)*	-0.2 (-0.3 to 0.0)
	Burkina Faso	SII	32.1 (24.3 to 39.2)*	0.2 (0.1 to 0.3)*	0.0 (0.0 to 0.0)*	-1.6 (-1.9 to -1.2)*	0.0 (0.0 to 0.0)*	-1.1 (-1.3 to -0.8)*
		RII	1.7 (1.5 to 1.9)*	1.0 (1.0 to 1.0)*	1.0 (1.0 to 1.0)*	-2.6 (-3.1 to -2.1)*	0.0 (0.0 to 0.0)*	-1.8 (-2.1 to -1.4)*
	Burundi	SII	4.0 (1.1 to 7.7)*	0.6 (-0.9 to 1.5)	0.0 (-0.9 to 0.7)	-0.2 (-0.3 to -0.1)*	-0.1 (-0.1 to 0.0)	-0.1 (-0.2 to -0.1)*
		RII	1.0 (1.0 to 1.1)*	1.0 (1.0 to 1.0)	1.0 (1.0 to 1.0)	-0.2 (-0.4 to -0.1)*	-0.1 (-0.1 to 0.0)	-0.1 (-0.3 to -0.1)*
	Cameroon	SII	16.5 (14.4 to 18.5)*	12.4 (9.7 to 14.7)*	9.3 (5.3 to 12.4)*	-0.2 (-0.3 to -0.1)*	-0.3 (-0.4 to -0.2)*	-0.2 (-0.4 to -0.1)*
		RII	1.2 (1.2 to 1.3)*	1.2 (1.1 to 1.2)*	1.1 (1.1 to 1.2)*	-0.2 (-0.4 to 0.0)*	-0.4 (-0.5 to -0.1)*	-0.3 (-0.4 to -0.1)*
	Chad	SII	36.0 (30.9 to 39.9)*	19.8 (16.3 to 23.7)*	12.0 (8.0 to 16.3)*	-0.8 (-0.9 to -0.6)*	-0.8 (-0.9 to -0.7)*	-0.8 (-0.9 to -0.6)*
		RII	2.0 (1.9 to 2.2)*	1.3 (1.2 to 1.4)*	1.2 (1.1 to 1.2)*	-2.1 (-2.6 to -1.6)*	-1.3 (-1.4 to -1.1)*	-1.8 (-2.2 to -1.5)*
	Comoros	SII	13.4 (5.0 to 22.3)*	2.4 (-1.7 to 8.0)	-0.1 (-4.2 to 3.8)	-0.6 (-0.9 to -0.1)*	-0.3 (-0.6 to 0.0)*	-0.5 (-0.7 to -0.2)*
		RII	1.2 (1.1 to 1.6)*	1.0 (1.0 to 1.1)	1.0 (0.9 to 1.1)	-0.9 (-2.3 to 0.0)	-0.3 (-1.0 to 0.0)*	-0.7 (-1.6 to -0.2)*
	Congo Brazzaville	SII	11.0 (8.3 to 15.0)*	7.5 (3.7 to 11.2)*	2.3 (-2.9 to 6.7)	-0.2 (-0.4 to 0.0)*	-0.5 (-0.7 to -0.3)*	-0.3 (-0.5 to -0.1)*
		RII	1.1 (1.1 to 1.2)*	1.1 (1.1 to 1.2)*	1.0 (0.9 to 1.1)	-0.1 (-0.4 to 0.2)	-0.7 (-1.2 to -0.3)*	-0.3 (-0.7 to 0.0)
	Congo Democratic Republic	SII	22.8 (19.1 to 25.9)*	4.7 (3.4 to 6.2)*	0.7 (0.3 to 1.4)*	-0.9 (-1.0 to -0.8)*	-0.4 (-0.5 to -0.3)*	-0.7 (-0.8 to -0.6)*
		RII	1.8 (1.7 to 2.0)*	1.1 (1.0 to 1.1)*	1.0 (1.0 to 1.0)*	-2.7 (-3.2 to -2.3)*	-0.4 (-0.5 to -0.3)*	-2.0 (-2.3 to -1.6)*
	Cote d'Ivoire	SII	21.2 (10.5 to 31.0)*	6.4 (2.3 to 14.9)*	2.6 (0.2 to 14.2)*	-0.7 (-1.3 to 0.1)	-0.3 (-0.5 to -0.1)*	-0.6 (-1.0 to 0.0)
		RII	1.3 (1.1 to 1.8)*	1.1 (1.0 to 1.2)*	1.0 (1.0 to 1.2)*	-1.1 (-2.5 to 0.3)	-0.4 (-0.5 to 0.0)	-0.9 (-1.8 to 0.2)
	Egypt	SII	2.7 (1.7 to 3.6)*	0.2 (-0.1 to 0.6)	-0.1 (-0.2 to 0.2)	-0.1 (-0.2 to -0.1)*	0.0 (0.0 to 0.0)*	-0.1 (-0.1 to -0.1)*
		RII	1.0 (1.0 to 1.0)*	1.0 (1.0 to 1.0)	1.0 (1.0 to 1.0)	-0.1 (-0.2 to -0.1)*	0.0 (0.0 to 0.0)*	-0.1 (-0.1 to -0.1)*
	Eswatini	SII	6.0 (1.7 to 11.6)*	0.4 (-0.7 to 3.4)	0.0 (-1.9 to 2.5)	-0.3 (-0.6 to 0.0)*	-0.1 (-0.3 to 0.0)	-0.2 (-0.4 to -0.1)*
		RII	1.1 (1.0 to 1.2)*	1.0 (1.0 to 1.0)	1.0 (1.0 to 1.0)	-0.3 (-0.7 to 0.0)*	-0.1 (-0.3 to 0.0)	-0.2 (-0.5 to -0.1)*
	Ethiopia	SII	38.1 (34.5 to 42.2)*	20.8 (18.3 to 23.4)*	12.4 (9.9 to 14.9)*	-0.9 (-1.0 to -0.7)*	-0.8 (-0.9 to -0.7)*	-0.9 (-1.0 to -0.7)*
		RII	2.1 (2.0 to 2.2)*	1.3 (1.3 to 1.4)*	1.2 (1.1 to 1.2)*	-2.3 (-2.5 to -2.0)*	-1.3 (-1.4 to -1.2)*	-1.9 (-2.1 to -1.7)*
	Gabon	SII	2.8 (-1.0 to 6.8)	-8.7 (-13.6 to -3.5)*	-14.1 (-19.6 to -8.2)*	-0.6 (-0.7 to -0.4)*	-0.5 (-0.7 to -0.4)*	-0.6 (-0.7 to -0.4)*
		RII	1.1 (1.0 to 1.2)	0.8 (0.8 to 0.9)*	0.8 (0.7 to 0.9)*	-1.3 (-1.7 to -0.8)*	-0.7 (-1.2 to -0.4)*	-1.1 (-1.5 to -0.7)*
	Gambia	SII	2.9 (1.2 to 6.1)*	1.8 (-0.2 to 3.3)	0.6 (-1.6 to 3.1)	-0.1 (-0.2 to 0.0)	-0.1 (-0.2 to 0.0)*	-0.1 (-0.2 to 0.0)
		RII	1.0 (1.0 to 1.1)*	1.0 (1.0 to 1.0)	1.0 (1.0 to 1.0)	-0.1 (-0.2 to 0.1)	-0.1 (-0.2 to 0.0)	-0.1 (-0.2 to 0.0)
	Ghana	SII	14.2 (10.6 to 18.0)*	3.4 (1.8 to 5.1)*	1.2 (0.3 to 2.6)*	-0.5 (-0.7 to -0.4)*	-0.2 (-0.3 to -0.1)*	-0.4 (-0.5 to -0.3)*
		RII	1.2 (1.1 to 1.2)*	1.0 (1.0 to 1.1)*	1.0 (1.0 to 1.0)*	-0.7 (-0.9 to -0.4)*	-0.2 (-0.3 to -0.2)*	-0.5 (-0.7 to -0.4)*
	Guinea	SII	23.8 (19.9 to 26.9)*	20.2 (15.1 to 25.6)*	16.0 (9.3 to 22.4)*	-0.2 (-0.3 to 0.1)	-0.4 (-0.6 to -0.2)*	-0.3 (-0.4 to -0.1)*
		RII	1.4 (1.3 to 1.4)*	1.4 (1.3 to 1.5)*	1.3 (1.2 to 1.5)*	0.0 (-0.3 to 0.4)	-0.4 (-0.8 to 0.1)	-0.1 (-0.5 to 0.3)
	Kenya	SII	16.0 (13.3 to 19.8)*	1.9 (1.1 to 2.7)*	0.4 (0.0 to 0.8)*	-0.7 (-0.9 to -0.6)*	-0.1 (-0.2 to -0.1)*	-0.5 (-0.6 to -0.4)*
		RII	1.2 (1.2 to 1.3)*	1.0 (1.0 to 1.0)*	1.0 (1.0 to 1.0)*	-1.0 (-1.2 to -0.8)*	-0.2 (-0.2 to -0.1)*	-0.7 (-0.8 to -0.6)*
	Lesotho	SII	8.8 (6.4 to 11.9)*	3.4 (0.7 to 6.2)*	0.3 (-2.7 to 4.0)	-0.3 (-0.4 to -0.1)*	-0.3 (-0.4 to -0.2)*	-0.3 (-0.4 to -0.2)*
		RII	1.1 (1.1 to 1.2)*	1.0 (1.0 to 1.1)*	1.0 (1.0 to 1.0)	-0.3 (-0.5 to -0.1)*	-0.3 (-0.5 to -0.2)*	-0.3 (-0.5 to -0.2)*
	Liberia	SII	21.2 (17.0 to 24.9)*	6.7 (4.4 to 9.6)*	2.3 (0.2 to 4.9)*	-0.7 (-0.9 to -0.5)*	-0.4 (-0.5 to -0.4)*	-0.6 (-0.7 to -0.5)*
		RII	1.4 (1.3 to 1.5)*	1.1 (1.1 to 1.1)*	1.0 (1.0 to 1.1)*	-1.3 (-1.5 to -0.9)*	-0.6 (-0.7 to -0.5)*	-1.0 (-1.2 to -0.8)*
	Madagascar	SII	34.2 (30.0 to 38.7)*	9.4 (6.5 to 12.9)*	3.9 (2.0 to 7.4)*	-1.3 (-1.5 to -0.9)*	-0.5 (-0.6 to -0.5)*	-1.0 (-1.2 to -0.8)*
		RII	1.7 (1.5 to 1.8)*	1.1 (1.1 to 1.2)*	1.0 (1.0 to 1.1)*	-2.0 (-2.5 to -1.4)*	-0.6 (-0.7 to -0.5)*	-1.5 (-1.9 to -1.1)*
	Malawi	SII	11.7 (10.0 to 13.4)*	3.1 (2.3 to 4.0)*	1.3 (0.7 to 2.0)*	-0.4 (-0.5 to -0.4)*	-0.2 (-0.2 to -0.1)*	-0.3 (-0.4 to -0.3)*
		RII	1.1 (1.1 to 1.2)*	1.0 (1.0 to 1.0)*	1.0 (1.0 to 1.0)*	-0.5 (-0.6 to -0.4)*	-0.2 (-0.2 to -0.2)*	-0.4 (-0.5 to -0.3)*
	Mali	SII	28.3 (24.8 to 31.2)*	12.2 (9.4 to 14.6)*	6.9 (4.2 to 8.9)*	-0.8 (-0.9 to -0.7)*	-0.6 (-0.6 to -0.5)*	-0.7 (-0.8 to -0.6)*
		RII	1.5 (1.4 to 1.6)*	1.2 (1.1 to 1.2)*	1.1 (1.0 to 1.1)*	-1.3 (-1.5 to -1.2)*	-0.7 (-0.8 to -0.6)*	-1.1 (-1.3 to -1.0)*
	Morocco	SII	4.7 (2.6 to 7.6)*	0.6 (0.0 to 5.3)*	0.2 (-0.2 to 5.5)	-0.2 (-0.4 to 0.1)	0.0 (-0.1 to 0.0)	-0.1 (-0.3 to 0.1)
		RII	1.1 (1.0 to 1.1)*	1.0 (1.0 to 1.1)*	1.0 (1.0 to 1.1)	-0.2 (-0.4 to 0.1)	0.0 (-0.1 to 0.0)	-0.2 (-0.3 to 0.1)
	Mozambique	SII	18.9 (15.4 to 22.1)*	6.8 (4.0 to 9.2)*	3.5 (1.3 to 5.8)*	-0.6 (-0.8 to -0.5)*	-0.3 (-0.4 to -0.3)*	-0.5 (-0.6 to -0.4)*
		RII	1.3 (1.2 to 1.3)*	1.1 (1.0 to 1.1)*	1.0 (1.0 to 1.1)*	-0.8 (-1.0 to -0.6)*	-0.4 (-0.4 to -0.3)*	-0.6 (-0.8 to -0.5)*

Indicators	Country	Index	Estimates of Inequalities: Year 2000	Estimates of Inequalities: Year 2020	Estimates of Inequalities: Year 2030	Changes in Inequalities: 2000–2020	Changes in Inequalities: 2020–2030	Changes in Inequalities: 2000–2030
	Namibia	SII	7.2 (4.6 to 9.6)*	1.1 (-1.7 to 4.2)	-1.4 (-5.1 to 1.4)	-0.3 (-0.4 to -0.2)*	-0.3 (-0.4 to -0.2)*	-0.3 (-0.4 to -0.2)*
		RII	1.1 (1.1 to 1.1)*	1.0 (1.0 to 1.0)	1.0 (0.9 to 1.0)	-0.3 (-0.5 to -0.2)*	-0.3 (-0.5 to -0.2)*	-0.3 (-0.4 to -0.2)*
	Niger	SII	32.7 (25.2 to 39.7)*	4.0 (2.8 to 5.8)*	0.7 (0.4 to 1.4)*	-1.4 (-1.8 to -1.1)*	-0.3 (-0.4 to -0.2)*	-1.1 (-1.3 to -0.8)*
		RII	1.9 (1.7 to 2.1)*	1.0 (1.0 to 1.1)*	1.0 (1.0 to 1.0)*	-3.0 (-3.5 to -2.4)*	-0.3 (-0.5 to -0.2)*	-2.1 (-2.5 to -1.7)*
	Nigeria	SII	41.6 (39.0 to 43.9)*	25.3 (23.7 to 26.7)*	15.2 (13.3 to 17.3)*	-0.8 (-0.9 to -0.7)*	-1.0 (-1.1 to -0.9)*	-0.9 (-1.0 to -0.8)*
		RII	2.6 (2.4 to 2.8)*	1.4 (1.4 to 1.5)*	1.2 (1.2 to 1.2)*	-2.9 (-3.3 to -2.6)*	-1.7 (-1.8 to -1.6)*	-2.5 (-2.8 to -2.3)*
	Rwanda	SII	6.1 (3.5 to 8.0)*	0.3 (-0.5 to 1.0)	-0.2 (-0.8 to 0.2)	-0.3 (-0.4 to -0.2)*	-0.1 (-0.1 to 0.0)*	-0.2 (-0.3 to -0.1)*
		RII	1.1 (1.0 to 1.1)*	1.0 (1.0 to 1.0)	1.0 (1.0 to 1.0)	-0.3 (-0.4 to -0.2)*	-0.1 (-0.1 to 0.0)*	-0.2 (-0.3 to -0.2)*
	Sao Tome and Principe	SII	6.7 (-0.5 to 17.0)	0.1 (-3.2 to 3.5)	-0.2 (-4.5 to 1.4)	-0.3 (-0.8 to -0.1)*	-0.1 (-0.3 to 0.1)	-0.3 (-0.6 to 0.0)*
		RII	1.1 (1.0 to 1.3)	1.0 (1.0 to 1.0)	1.0 (0.9 to 1.0)	-0.4 (-1.2 to 0.0)*	-0.1 (-0.4 to 0.1)	-0.3 (-0.8 to 0.0)*
	Senegal	SII	19.4 (16.6 to 22.0)*	4.0 (3.1 to 4.7)*	1.5 (0.9 to 2.0)*	-0.8 (-0.9 to -0.6)*	-0.2 (-0.3 to -0.2)*	-0.6 (-0.7 to -0.5)*
		RII	1.3 (1.2 to 1.3)*	1.0 (1.0 to 1.1)*	1.0 (1.0 to 1.0)*	-1.0 (-1.2 to -0.8)*	-0.3 (-0.3 to -0.2)*	-0.7 (-0.9 to -0.6)*
	Sierra Leone	SII	17.8 (13.1 to 22.9)*	2.5 (0.6 to 4.1)*	0.2 (-0.8 to 1.1)	-0.8 (-1.0 to -0.6)*	-0.2 (-0.3 to -0.1)*	-0.6 (-0.7 to -0.4)*
		RII	1.3 (1.2 to 1.4)*	1.0 (1.0 to 1.0)*	1.0 (1.0 to 1.0)	-1.3 (-1.6 to -0.9)*	-0.3 (-0.4 to -0.1)*	-0.9 (-1.2 to -0.7)*
	South Africa	SII	14.2 (5.2 to 22.9)*	4.4 (-3.2 to 12.4)	-0.3 (-6.6 to 6.6)	-0.5 (-0.8 to -0.2)*	-0.5 (-0.7 to -0.1)*	-0.5 (-0.7 to -0.3)*
		RII	1.3 (1.1 to 1.8)*	1.1 (1.0 to 1.2)	1.0 (0.9 to 1.1)	-0.9 (-2.4 to 0.0)*	-0.6 (-1.0 to -0.1)*	-0.8 (-1.9 to -0.3)*
	Tanzania	SII	9.2 (7.4 to 11.3)*	4.7 (2.8 to 6.4)*	2.7 (0.8 to 4.9)*	-0.2 (-0.3 to -0.1)*	-0.2 (-0.2 to -0.1)*	-0.2 (-0.3 to -0.1)*
		RII	1.1 (1.1 to 1.1)*	1.1 (1.0 to 1.1)*	1.0 (1.0 to 1.1)*	-0.3 (-0.4 to -0.1)*	-0.2 (-0.3 to -0.2)*	-0.2 (-0.3 to -0.1)*
	Togo	SII	17.1 (7.0 to 27.3)*	3.4 (0.8 to 7.1)*	0.9 (-0.4 to 4.8)	-0.7 (-1.2 to -0.1)*	-0.2 (-0.3 to -0.1)*	-0.5 (-0.9 to -0.2)*
		RII	1.3 (1.1 to 1.6)*	1.0 (1.0 to 1.1)*	1.0 (1.0 to 1.1)	-0.9 (-2.2 to -0.1)*	-0.2 (-0.4 to -0.1)*	-0.7 (-1.5 to -0.2)*
	Uganda	SII	17.6 (14.1 to 20.8)*	2.7 (1.0 to 4.3)*	0.3 (-0.7 to 1.2)	-0.7 (-0.9 to -0.6)*	-0.2 (-0.3 to -0.2)*	-0.6 (-0.7 to -0.5)*
		RII	1.3 (1.3 to 1.4)*	1.0 (1.0 to 1.0)*	1.0 (1.0 to 1.0)	-1.2 (-1.4 to -1.0)*	-0.3 (-0.4 to -0.2)*	-0.9 (-1.1 to -0.7)*
	Zambia	SII	13.8 (11.5 to 15.6)*	4.7 (3.3 to 5.9)*	2.1 (0.9 to 3.3)*	-0.5 (-0.6 to -0.4)*	-0.3 (-0.3 to -0.2)*	-0.4 (-0.5 to -0.3)*
		RII	1.2 (1.1 to 1.2)*	1.1 (1.0 to 1.1)*	1.0 (1.0 to 1.0)*	-0.6 (-0.7 to -0.4)*	-0.3 (-0.3 to -0.2)*	-0.5 (-0.6 to -0.4)*
	Zimbabwe	SII	17.7 (13.1 to 21.8)*	2.8 (1.0 to 4.8)*	0.0 (-1.0 to 1.3)	-0.7 (-0.9 to -0.6)*	-0.3 (-0.4 to -0.2)*	-0.6 (-0.7 to -0.5)*
		RII	1.4 (1.3 to 1.5)*	1.0 (1.0 to 1.1)*	1.0 (1.0 to 1.0)	-1.5 (-1.9 to -1.1)*	-0.3 (-0.5 to -0.2)*	-1.1 (-1.4 to -0.8)*
DPT3 immunization	Angola	SII	37.3 (23.2 to 41.7)*	27.4 (22.1 to 31.5)*	19.9 (8.1 to 25.9)*	-0.5 (-0.7 to 0.0)	-0.7 (-1.4 to -0.5)*	-0.6 (-0.7 to -0.3)*
		RII	2.2 (1.3 to 3.6)*	2.0 (1.7 to 2.3)*	1.8 (1.3 to 2.3)*	-0.6 (-3.3 to 2.4)	-1.0 (-2.5 to 0.1)	-0.7 (-3.0 to 1.5)
	Benin	SII	23.7 (21.4 to 26.2)*	15.8 (13.2 to 18.2)*	11.9 (8.8 to 15.0)*	-0.4 (-0.5 to -0.3)*	-0.4 (-0.5 to -0.3)*	-0.4 (-0.5 to -0.3)*
		RII	1.4 (1.3 to 1.4)*	1.2 (1.2 to 1.3)*	1.2 (1.1 to 1.2)*	-0.5 (-0.7 to -0.3)*	-0.5 (-0.6 to -0.4)*	-0.5 (-0.7 to -0.3)*
	Burkina Faso	SII	26.1 (17.2 to 35.0)*	0.4 (0.1 to 0.7)*	0.0 (0.0 to 0.0)	-1.3 (-1.7 to -0.9)*	0.0 (-0.1 to 0.0)*	-0.9 (-1.2 to -0.6)*
		RII	1.7 (1.4 to 1.9)*	1.0 (1.0 to 1.0)*	1.0 (1.0 to 1.0)	-2.6 (-3.2 to -1.8)*	0.0 (-0.1 to 0.0)*	-1.7 (-2.2 to -1.2)*
	Burundi	SII	2.7 (-1.1 to 6.0)	-0.4 (-1.8 to 0.6)	-0.6 (-1.6 to 0.1)	-0.1 (-0.3 to 0.0)*	0.0 (-0.1 to 0.1)	-0.1 (-0.2 to 0.0)*
		RII	1.0 (1.0 to 1.1)	1.0 (1.0 to 1.0)	1.0 (1.0 to 1.0)	-0.2 (-0.3 to 0.0)*	0.0 (-0.1 to 0.1)	-0.1 (-0.2 to 0.0)*
	Cameroon	SII	27.5 (24.5 to 30.1)*	17.2 (14.6 to 20.3)*	12.1 (9.1 to 16.1)*	-0.5 (-0.7 to -0.3)*	-0.5 (-0.6 to -0.4)*	-0.5 (-0.6 to -0.4)*
		RII	1.5 (1.4 to 1.6)*	1.3 (1.2 to 1.3)*	1.2 (1.1 to 1.2)*	-0.8 (-1.1 to -0.5)*	-0.7 (-0.9 to -0.5)*	-0.8 (-1.0 to -0.5)*
	Chad	SII	27.0 (21.8 to 32.9)*	21.6 (17.1 to 26.6)*	14.6 (9.8 to 20.4)*	-0.2 (-0.4 to -0.1)*	-0.7 (-0.9 to -0.5)*	-0.4 (-0.5 to -0.3)*
		RII	2.7 (2.4 to 3.2)*	1.6 (1.4 to 1.7)*	1.3 (1.2 to 1.4)*	-2.7 (-3.3 to -2.2)*	-2.0 (-2.3 to -1.7)*	-2.5 (-3.0 to -2.1)*
	Comoros	SII	18.5 (8.7 to 27.8)*	4.3 (-0.3 to 10.5)	0.5 (-3.4 to 5.6)	-0.7 (-1.0 to -0.1)*	-0.4 (-0.6 to -0.1)*	-0.6 (-0.8 to -0.2)*
		RII	1.4 (1.1 to 2.0)*	1.1 (1.0 to 1.2)	1.0 (1.0 to 1.1)	-1.5 (-3.2 to 0.0)	-0.5 (-0.8 to -0.1)*	-1.1 (-2.2 to -0.2)*
	Congo Brazzaville	SII	17.8 (13.9 to 21.3)*	10.4 (6.0 to 14.6)*	3.7 (-0.7 to 7.7)	-0.4 (-0.5 to -0.2)*	-0.7 (-0.8 to -0.5)*	-0.5 (-0.6 to -0.3)*
		RII	1.3 (1.2 to 1.4)*	1.3 (1.1 to 1.4)*	1.1 (1.0 to 1.3)	-0.1 (-0.5 to 0.5)	-1.1 (-1.7 to -0.6)*	-0.4 (-0.9 to 0.1)
	Congo Democratic Republic	SII	23.3 (19.3 to 27.2)*	10.3 (7.6 to 12.9)*	3.0 (1.4 to 4.9)*	-0.6 (-0.8 to -0.5)*	-0.7 (-0.8 to -0.6)*	-0.7 (-0.8 to -0.6)*
		RII	2.1 (1.8 to 2.3)*	1.1 (1.1 to 1.2)*	1.0 (1.0 to 1.1)*	-2.9 (-3.4 to -2.4)*	-1.0 (-1.2 to -0.8)*	-2.3 (-2.6 to -1.9)*
	Cote d'Ivoire	SII	29.6 (19.0 to 40.1)*	14.1 (6.7 to 23.7)*	7.3 (1.2 to 19.9)*	-0.8 (-1.4 to 0.2)	-0.6 (-0.8 to -0.3)*	-0.7 (-1.2 to 0.0)*
		RII	1.6 (1.3 to 2.4)*	1.2 (1.1 to 1.5)*	1.1 (1.0 to 1.5)*	-1.5 (-4.0 to 0.7)	-0.8 (-1.0 to 0.1)	-1.3 (-2.9 to 0.5)
	Egypt	SII	4.2 (3.1 to 5.4)*	0.3 (-0.1 to 0.8)	-0.1 (-0.4 to 0.2)	-0.2 (-0.2 to -0.2)*	0.0 (-0.1 to 0.0)*	-0.1 (-0.2 to -0.1)*
		RII	1.0 (1.0 to 1.1)*	1.0 (1.0 to 1.0)	1.0 (1.0 to 1.0)	-0.2 (-0.3 to -0.2)*	0.0 (-0.1 to 0.0)*	-0.2 (-0.2 to -0.1)*
	Eswatini	SII	6.4 (2.2 to 12.7)*	0.5 (-0.2 to 3.5)	0.0 (-1.1 to 2.3)	-0.3 (-0.6 to 0.0)*	-0.1 (-0.2 to 0.0)	-0.2 (-0.4 to 0.0)*
		RII	1.1 (1.0 to 1.2)*	1.0 (1.0 to 1.0)	1.0 (1.0 to 1.0)	-0.3 (-0.7 to 0.0)*	-0.1 (-0.2 to 0.0)	-0.2 (-0.5 to 0.0)*
	Ethiopia	SII	36.4 (32.5 to 40.5)*	20.5 (18.1 to 22.7)*	9.5 (7.4 to 11.2)*	-0.8 (-0.9 to -0.7)*	-1.1 (-1.2 to -1.0)*	-0.9 (-1.0 to -0.8)*
		RII	2.8 (2.5 to 3.0)*	1.3 (1.3 to 1.4)*	1.1 (1.1 to 1.1)*	-3.6 (-3.9 to -3.3)*	-1.7 (-1.8 to -1.5)*	-3.0 (-3.2 to -2.7)*
	Gabon	SII	19.5 (15.2 to 23.7)*	2.7 (0.9 to 4.6)*	0.2 (-0.5 to 0.8)	-0.8 (-1.0 to -0.7)*	-0.3 (-0.4 to -0.1)*	-0.6 (-0.8 to -0.5)*
		RII	1.6 (1.5 to 1.8)*	1.0 (1.0 to 1.1)*	1.0 (1.0 to 1.0)	-2.2 (-2.8 to -1.8)*	-0.3 (-0.4 to -0.1)*	-1.6 (-2.0 to -1.3)*
	Gambia	SII	6.9 (2.1 to 11.7)*	-0.8 (-2.6 to 0.9)	-1.2 (-2.6 to -0.3)*	-0.4 (-0.6 to -0.2)*	0.0 (-0.1 to 0.1)	-0.3 (-0.4 to -0.1)*
		RII	1.1 (1.0 to 1.2)*	1.0 (1.0 to 1.0)	1.0 (1.0 to 1.0)*	-0.5 (-0.9 to -0.3)*	0.0 (-0.2 to 0.1)	-0.4 (-0.6 to -0.2)*
	Ghana	SII	15.9 (12.6 to 19.2)*	3.7 (1.9 to 5.7)*	1.2 (-0.1 to 2.8)	-0.6 (-0.8 to -0.4)*	-0.3 (-0.3 to -0.2)*	-0.5 (-0.6 to -0.4)*
		RII	1.2 (1.2 to 1.3)*	1.0 (1.0 to 1.1)*	1.0 (1.0 to 1.0)	-0.8 (-1.0 to -0.6)*	-0.3 (-0.4 to -0.2)*	-0.6 (-0.8 to -0.5)*
	Guinea	SII	25.8 (21.7 to 29.4)*	18.7 (13.5 to 23.4)*	11.5 (6.6 to 17.1)*	-0.4 (-0.5 to -0.2)*	-0.7 (-0.9 to -0.6)*	-0.5 (-0.6 to -0.3)*
		RII	1.5 (1.4 to 1.6)*	1.5 (1.3 to 1.6)*	1.4 (1.2 to 1.6)*	0.2 (-0.2 to 0.6)	-0.7 (-1.3 to -0.2)*	-0.1 (-0.6 to 0.3)
	Kenya	SII	17.6 (13.9 to 21.0)*	2.0 (1.1 to 2.9)*	0.3 (-0.1 to 0.8)	-0.8 (-0.9 to -0.6)*	-0.2 (-0.2 to -0.1)*	-0.6 (-0.7 to -0.5)*
		RII	1.3 (1.2 to 1.3)*	1.0 (1.0 to 1.0)*	1.0 (1.0 to 1.0)	-1.1 (-1.3 to -0.9)*	-0.2 (-0.2 to -0.1)*	-0.8 (-1.0 to -0.6)*
	Lesotho	SII	9.2 (5.9 to 12.6)*	1.9 (-1.0 to 4.8)	-1.1 (-4.3 to 1.9)	-0.4 (-0.5 to -0.3)*	-0.3 (-0.4 to -0.2)*	-0.3 (-0.4 to -0.3)*
		RII	1.1 (1.1 to 1.2)*	1.0 (1.0 to 1.1)	1.0 (0.9 to 1.0)	-0.5 (-0.6 to -0.3)*	-0.3 (-0.5 to -0.2)*	-0.4 (-0.5 to -0.3)*
	Liberia	SII	23.2 (19.1 to 27.6)*	8.2 (5.2 to 10.9)*	2.4 (0.1 to 4.9)*	-0.8 (-0.9 to -0.6)*	-0.6 (-0.6 to -0.5)*	-0.7 (-0.8 to -0.6)*
		RII	1.6 (1.5 to 1.8)*	1.1 (1.1 to 1.2)*	1.0 (1.0 to 1.1)*	-1.8 (-2.2 to -1.4)*	-0.8 (-0.9 to -0.6)*	-1.5 (-1.8 to -1.2)*
	Madagascar	SII	36.3 (31.9 to 40.8)*	6.8 (5.0 to 9.9)*	2.1 (1.1 to 4.1)*	-1.5 (-1.8 to -1.2)*	-0.5 (-0.6 to -0.4)*	-1.1 (-1.3 to -1.0)*
		RII	1.8 (1.6 to 2.0)*	1.1 (1.1 to 1.1)*	1.0 (1.0 to 1.0)*	-2.5 (-3.1 to -1.9)*	-0.5 (-0.7 to -0.4)*	-1.9 (-2.2 to -1.5)*
	Malawi	SII	11.4 (9.5 to 13.3)*	2.3 (1.5 to 3.0)*	0.8 (0.2 to 1.3)*	-0.5 (-0.5 to -0.4)*	-0.2 (-0.2 to -0.1)*	-0.4 (-0.4 to -0.3)*

Indicators	Country	Index	Estimates of Inequalities			Changes in Inequalities		
			Year 2000	Year 2020	Year 2030	2000–2020	2020–2030	2000–2030
		RII	1.1 (1.1 to 1.2)*	1.0 (1.0 to 1.0)*	1.0 (1.0 to 1.0)*	-0.5 (-0.6 to -0.5)*	-0.2 (-0.2 to -0.1)*	-0.4 (-0.5 to -0.4)*
	Mali	SII	30.1 (26.7 to 34.1)*	10.5 (8.4 to 12.5)*	4.4 (2.8 to 5.9)*	-1.0 (-1.1 to -0.9)*	-0.6 (-0.7 to -0.5)*	-0.9 (-1.0 to -0.8)*
		RII	1.7 (1.6 to 1.8)*	1.1 (1.1 to 1.2)*	1.0 (1.0 to 1.1)*	-2.0 (-2.2 to -1.7)*	-0.8 (-0.8 to -0.7)*	-1.6 (-1.8 to -1.4)*
	Morocco	SII	5.3 (3.0 to 8.6)*	0.6 (0.0 to 5.4)*	0.2 (-0.1 to 4.7)	-0.2 (-0.4 to 0.1)	0.0 (-0.1 to 0.0)*	-0.2 (-0.3 to 0.0)
		RII	1.1 (1.0 to 1.1)*	1.0 (1.0 to 1.1)*	1.0 (1.0 to 1.1)	-0.2 (-0.5 to 0.1)	0.0 (-0.1 to 0.0)*	-0.2 (-0.3 to 0.0)
	Mozambique	SII	19.1 (16.1 to 21.9)*	10.1 (6.7 to 13.4)*	6.2 (2.9 to 10.0)*	-0.5 (-0.6 to -0.3)*	-0.4 (-0.5 to -0.3)*	-0.4 (-0.5 to -0.3)*
		RII	1.3 (1.2 to 1.3)*	1.1 (1.1 to 1.2)*	1.1 (1.0 to 1.1)*	-0.6 (-0.8 to -0.4)*	-0.5 (-0.6 to -0.4)*	-0.6 (-0.7 to -0.4)*
	Namibia	SII	9.6 (6.4 to 12.5)*	1.2 (-1.2 to 4.1)	-1.7 (-4.5 to 0.9)	-0.4 (-0.5 to -0.3)*	-0.3 (-0.4 to -0.2)*	-0.4 (-0.5 to -0.3)*
		RII	1.1 (1.1 to 1.2)*	1.0 (1.0 to 1.1)	1.0 (0.9 to 1.0)	-0.5 (-0.7 to -0.4)*	-0.3 (-0.5 to -0.2)*	-0.5 (-0.6 to -0.4)*
	Niger	SII	29.3 (22.3 to 36.8)*	5.5 (3.8 to 7.5)*	0.7 (0.4 to 1.2)*	-1.2 (-1.5 to -0.9)*	-0.5 (-0.6 to -0.3)*	-0.9 (-1.2 to -0.7)*
		RII	2.8 (2.3 to 3.4)*	1.1 (1.0 to 1.1)*	1.0 (1.0 to 1.0)*	-4.7 (-5.7 to -3.9)*	-0.5 (-0.7 to -0.4)*	-3.3 (-4.0 to -2.8)*
	Nigeria	SII	46.4 (43.7 to 49.1)*	43.6 (41.9 to 45.0)*	33.2 (30.7 to 35.7)*	-0.1 (-0.3 to 0.0)*	-1.0 (-1.2 to -0.8)*	-0.4 (-0.6 to -0.3)*
		RII	5.4 (4.9 to 5.8)*	2.3 (2.2 to 2.4)*	1.6 (1.6 to 1.8)*	-4.2 (-4.6 to -3.6)*	-3.2 (-3.5 to -2.9)*	-3.9 (-4.2 to -3.4)*
	Rwanda	SII	5.7 (3.0 to 8.4)*	-0.4 (-1.1 to 0.4)	-0.5 (-1.0 to -0.1)*	-0.3 (-0.4 to -0.2)*	0.0 (-0.1 to 0.0)	-0.2 (-0.3 to -0.1)*
		RII	1.1 (1.0 to 1.1)*	1.0 (1.0 to 1.0)	1.0 (1.0 to 1.0)*	-0.4 (-0.5 to -0.2)*	0.0 (-0.1 to 0.0)	-0.2 (-0.3 to -0.1)*
	Sao Tome and Principe	SII	10.9 (2.7 to 21.7)*	0.7 (-1.9 to 4.8)	0.0 (-3.6 to 2.5)	-0.5 (-1.0 to -0.1)*	-0.1 (-0.3 to 0.0)	-0.4 (-0.7 to -0.1)*
		RII	1.2 (1.0 to 1.4)*	1.0 (1.0 to 1.1)	1.0 (1.0 to 1.0)	-0.6 (-1.5 to -0.1)*	-0.1 (-0.3 to 0.0)	-0.5 (-1.0 to -0.2)*
	Senegal	SII	25.0 (22.1 to 28.2)*	5.7 (4.8 to 6.5)*	2.3 (1.7 to 3.0)*	-1.0 (-1.1 to -0.8)*	-0.3 (-0.4 to -0.3)*	-0.8 (-0.9 to -0.7)*
		RII	1.4 (1.3 to 1.4)*	1.1 (1.1 to 1.1)*	1.0 (1.0 to 1.0)*	-1.3 (-1.5 to -1.1)*	-0.4 (-0.4 to -0.3)*	-1.0 (-1.1 to -0.8)*
	Sierra Leone	SII	16.3 (11.2 to 21.0)*	1.7 (-0.1 to 3.3)	-0.3 (-1.1 to 0.4)	-0.7 (-0.9 to -0.6)*	-0.2 (-0.3 to -0.1)*	-0.6 (-0.7 to -0.4)*
		RII	1.5 (1.3 to 1.6)*	1.0 (1.0 to 1.0)	1.0 (1.0 to 1.0)	-1.8 (-2.2 to -1.4)*	-0.2 (-0.3 to -0.1)*	-1.3 (-1.6 to -1.0)*
	South Africa	SII	15.4 (6.2 to 25.1)*	5.1 (-3.2 to 15.9)	-0.4 (-8.0 to 8.7)	-0.5 (-0.7 to -0.1)*	-0.6 (-0.8 to -0.2)*	-0.5 (-0.7 to -0.2)*
		RII	1.4 (1.1 to 2.2)*	1.1 (1.0 to 1.4)	1.0 (0.9 to 1.3)	-1.3 (-3.2 to 0.1)	-0.9 (-1.5 to -0.2)*	-1.1 (-2.5 to -0.2)*
	Tanzania	SII	8.8 (6.8 to 10.8)*	4.5 (2.4 to 6.4)*	2.5 (0.2 to 4.8)*	-0.2 (-0.3 to -0.1)*	-0.2 (-0.2 to -0.2)*	-0.2 (-0.3 to -0.1)*
		RII	1.1 (1.1 to 1.1)*	1.1 (1.0 to 1.1)*	1.0 (1.0 to 1.1)*	-0.2 (-0.4 to -0.1)*	-0.2 (-0.3 to -0.2)*	-0.2 (-0.3 to -0.2)*
	Togo	SII	18.8 (10.2 to 28.5)*	3.5 (0.8 to 7.4)*	0.7 (-0.6 to 4.2)	-0.8 (-1.2 to -0.3)*	-0.3 (-0.4 to -0.1)*	-0.6 (-0.9 to -0.3)*
		RII	1.4 (1.1 to 1.9)*	1.0 (1.0 to 1.1)*	1.0 (1.0 to 1.0)	-1.3 (-3.0 to -0.3)*	-0.3 (-0.5 to -0.1)*	-1.0 (-2.1 to -0.3)*
	Uganda	SII	16.9 (13.7 to 20.8)*	2.4 (0.5 to 4.2)*	-0.4 (-1.8 to 0.8)	-0.7 (-0.9 to -0.6)*	-0.3 (-0.3 to -0.2)*	-0.6 (-0.7 to -0.5)*
		RII	1.4 (1.3 to 1.5)*	1.0 (1.0 to 1.1)*	1.0 (1.0 to 1.0)	-1.4 (-1.7 to -1.2)*	-0.3 (-0.4 to -0.2)*	-1.1 (-1.3 to -0.9)*
	Zambia	SII	15.7 (13.4 to 18.4)*	4.5 (3.2 to 5.9)*	1.7 (0.8 to 2.8)*	-0.6 (-0.7 to -0.5)*	-0.3 (-0.3 to -0.2)*	-0.5 (-0.5 to -0.4)*
		RII	1.2 (1.2 to 1.3)*	1.1 (1.0 to 1.1)*	1.0 (1.0 to 1.0)*	-0.7 (-0.9 to -0.6)*	-0.3 (-0.4 to -0.3)*	-0.6 (-0.7 to -0.5)*
	Zimbabwe	SII	15.6 (11.1 to 20.5)*	1.1 (-0.4 to 3.0)	-0.3 (-1.0 to 0.3)	-0.7 (-0.9 to -0.6)*	-0.1 (-0.3 to 0.0)*	-0.5 (-0.7 to -0.4)*
		RII	1.4 (1.2 to 1.5)*	1.0 (1.0 to 1.0)	1.0 (1.0 to 1.0)	-1.5 (-2.0 to -1.1)*	-0.2 (-0.3 to 0.0)*	-1.1 (-1.4 to -0.7)*

Notes: SII = Slope Index of Inequality; RII = Relative Index of Inequality

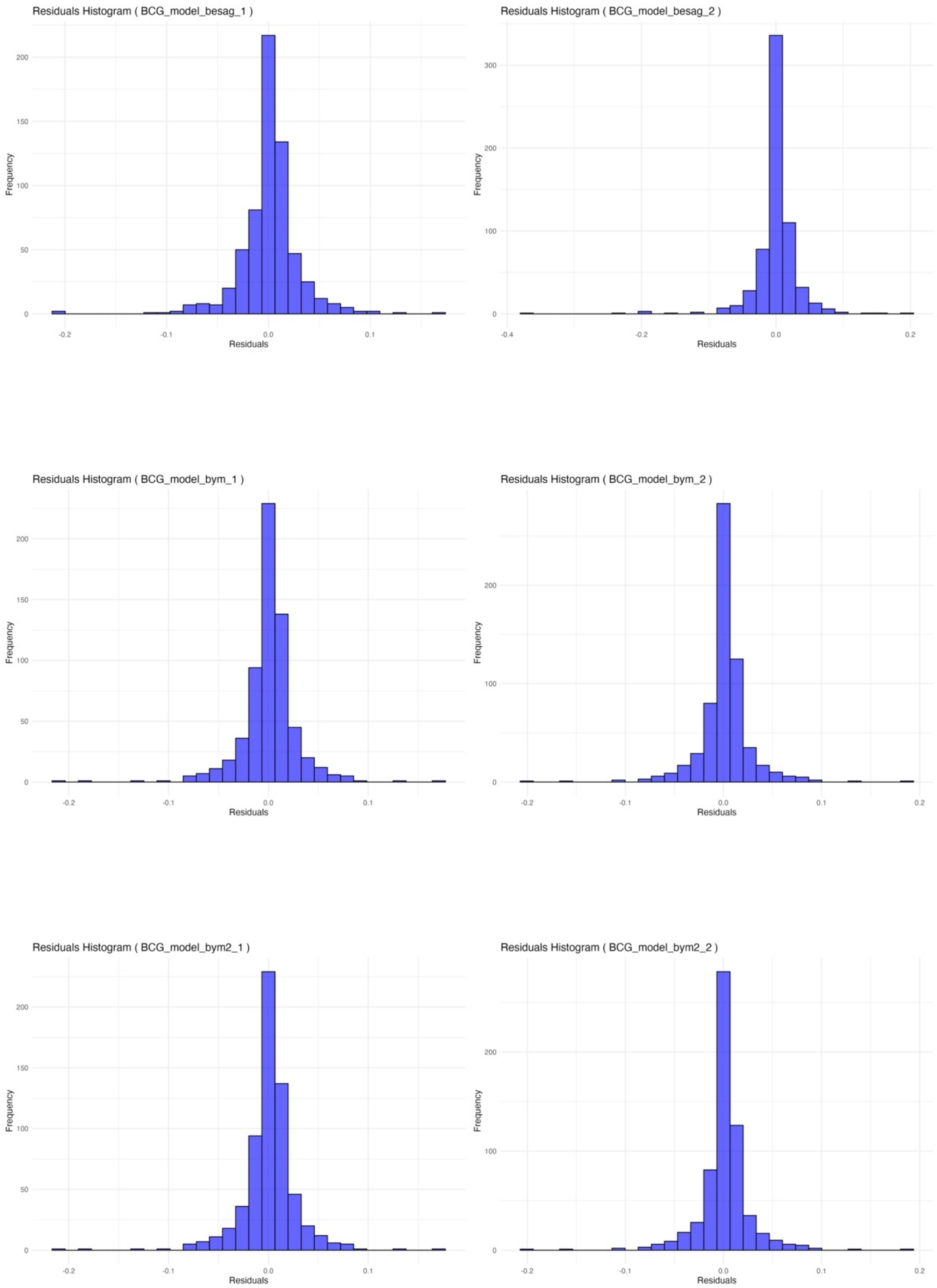

Fig A1. Residual histogram for 6 comparing models

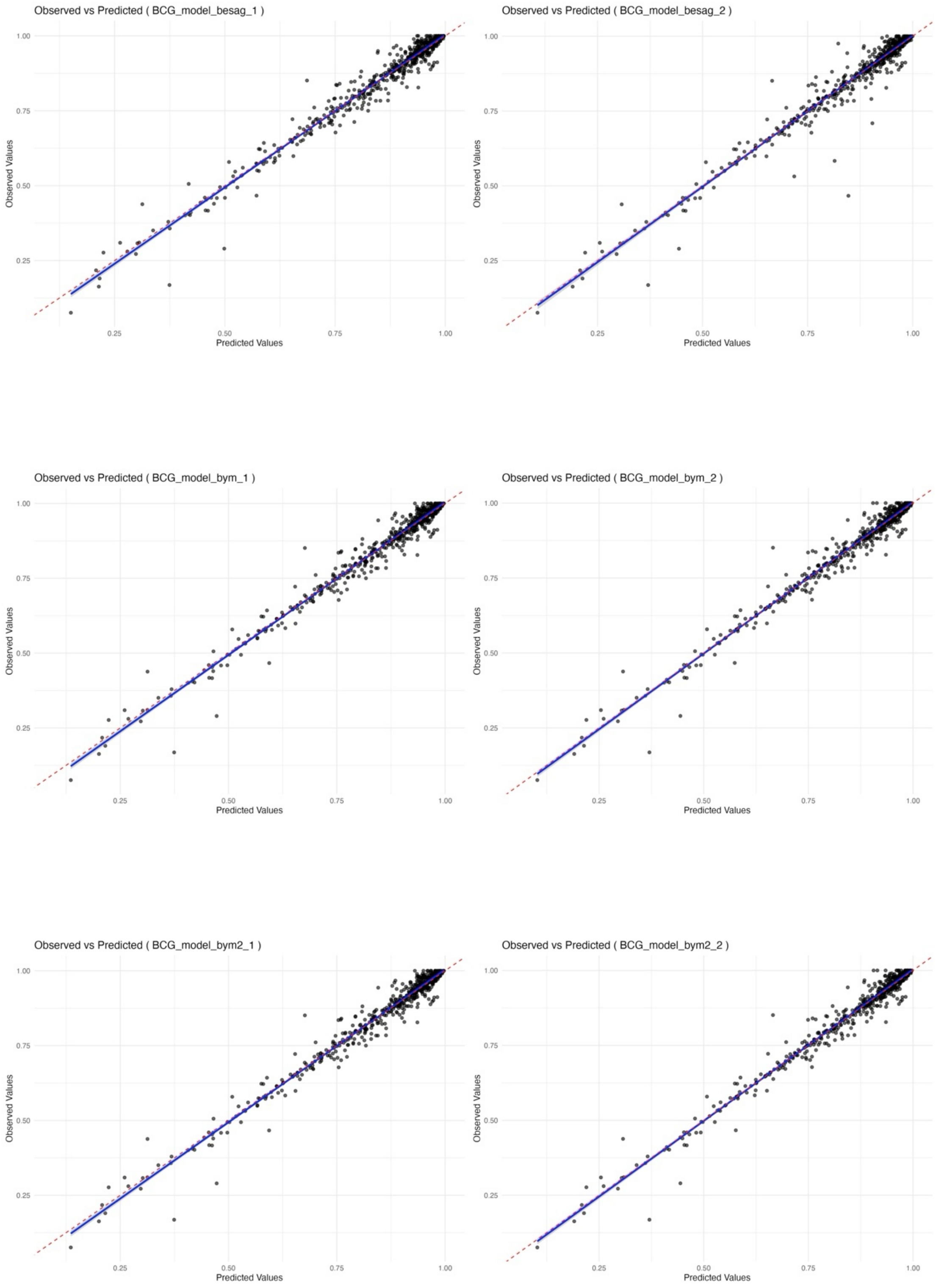

Fig A2. Predicted vs. observed values plot for 6 comparing models

Notes: Each plot compares predicted values with observed values from DHS surveys. The red line represents a perfect agreement, where all predicted values match the observed ones. The blue line represents the best fit for the scatter points. The closer the blue line is to the red line, the better the model's performance.

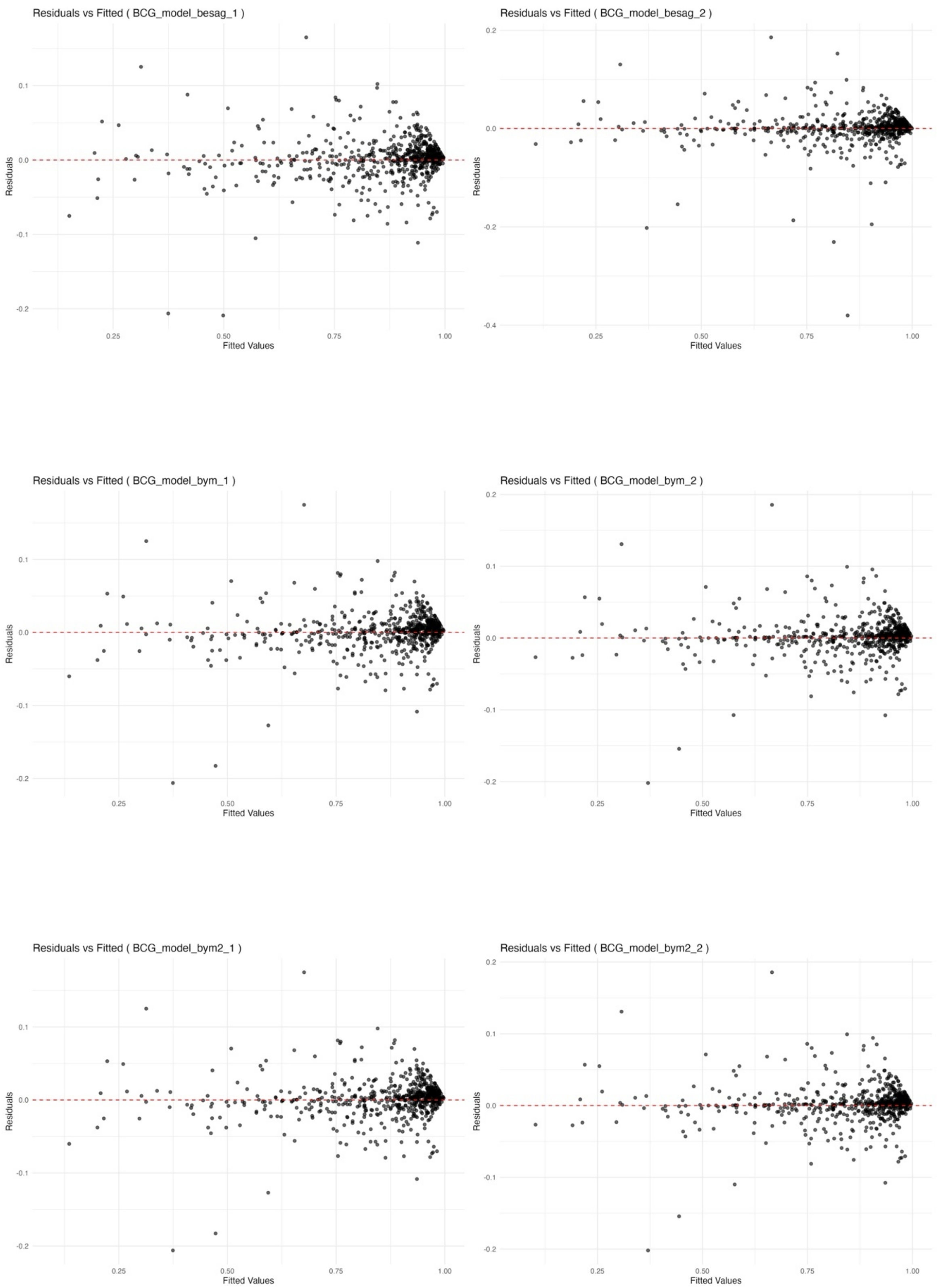

Fig A3. Residual vs. fitted plot for 6 comparing models

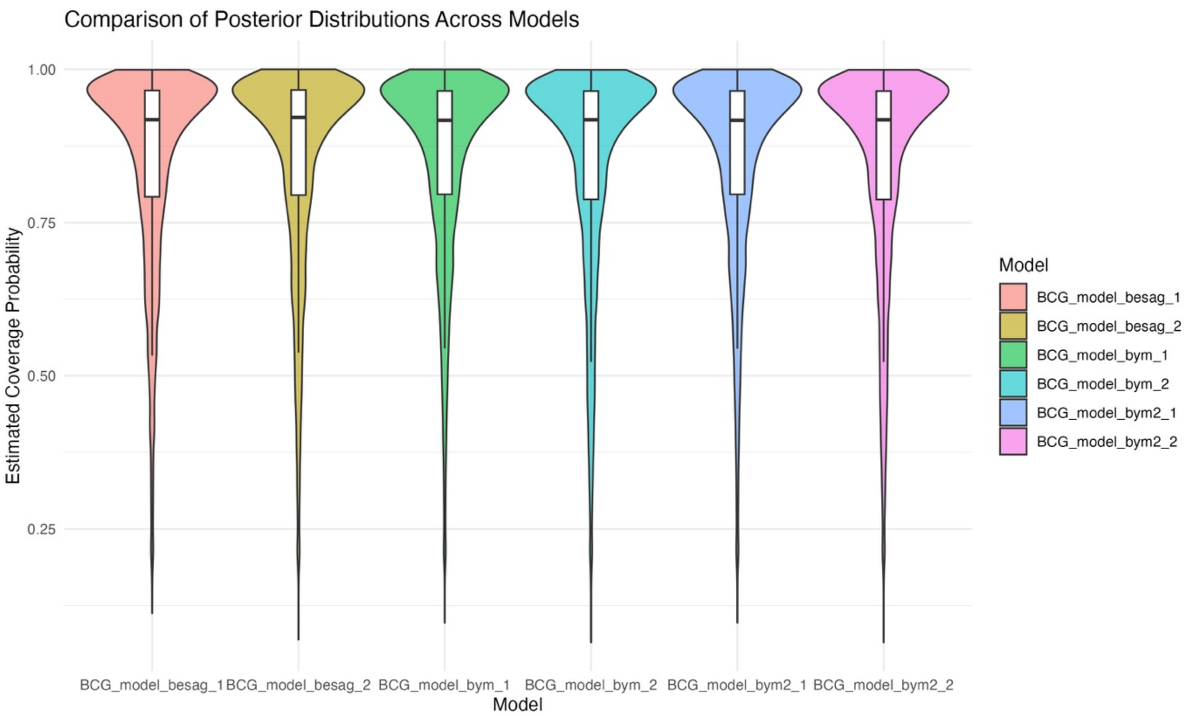

Fig A4. Posterior Distribution of BCG immunization coverage for 6 comparing models

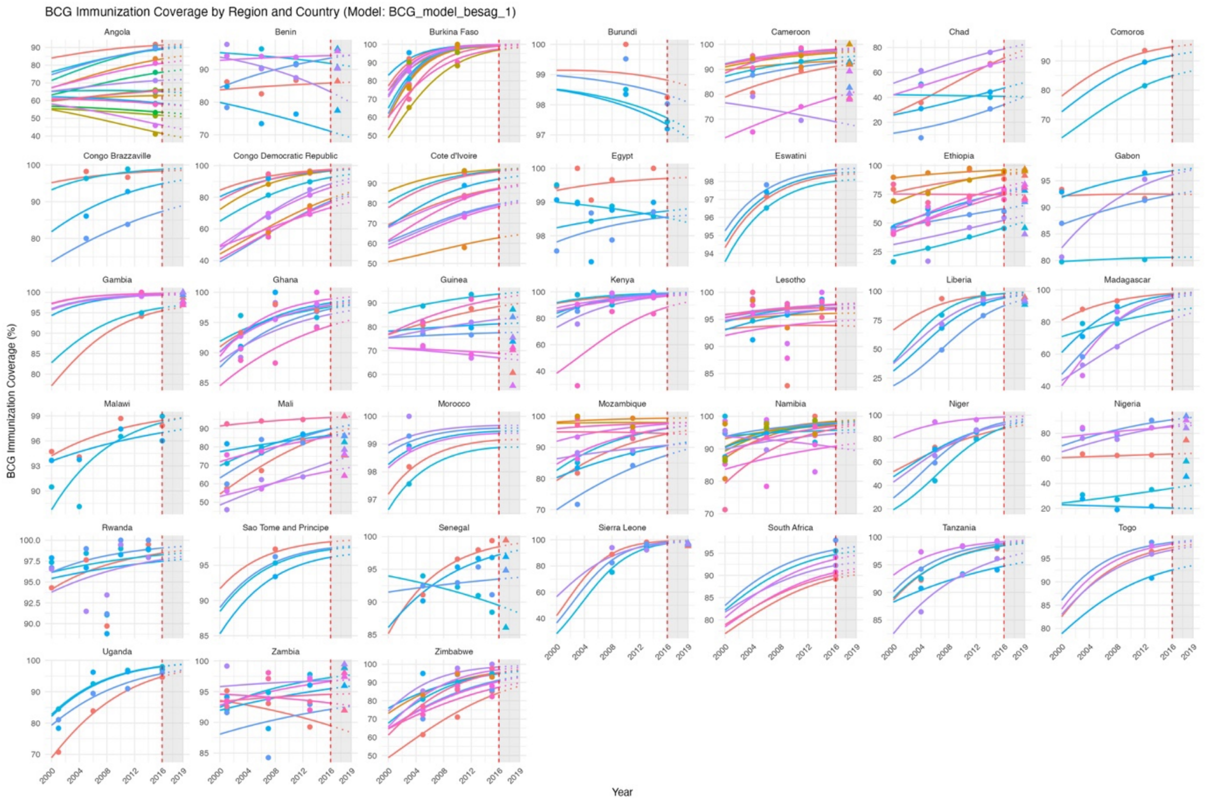

Fig A5. Trends in BCG immunization coverage at regional levels, using model_besag_1

Notes: The model was trained on data from 2000 to 2016 and used to predict coverage for 2017–2019. Red dotted vertical lines separate the model-fitting period (2000–2016) from the prediction period (2017–2019, shaded area). Round dots indicate observed regional coverage during the model-fitting period, while triangle markers represent observed data from 2017–2019, used for out-of-sample validation. Solid lines show estimated trends from 2000–2016; dashed lines represent model-based predictions for 2017–2019.

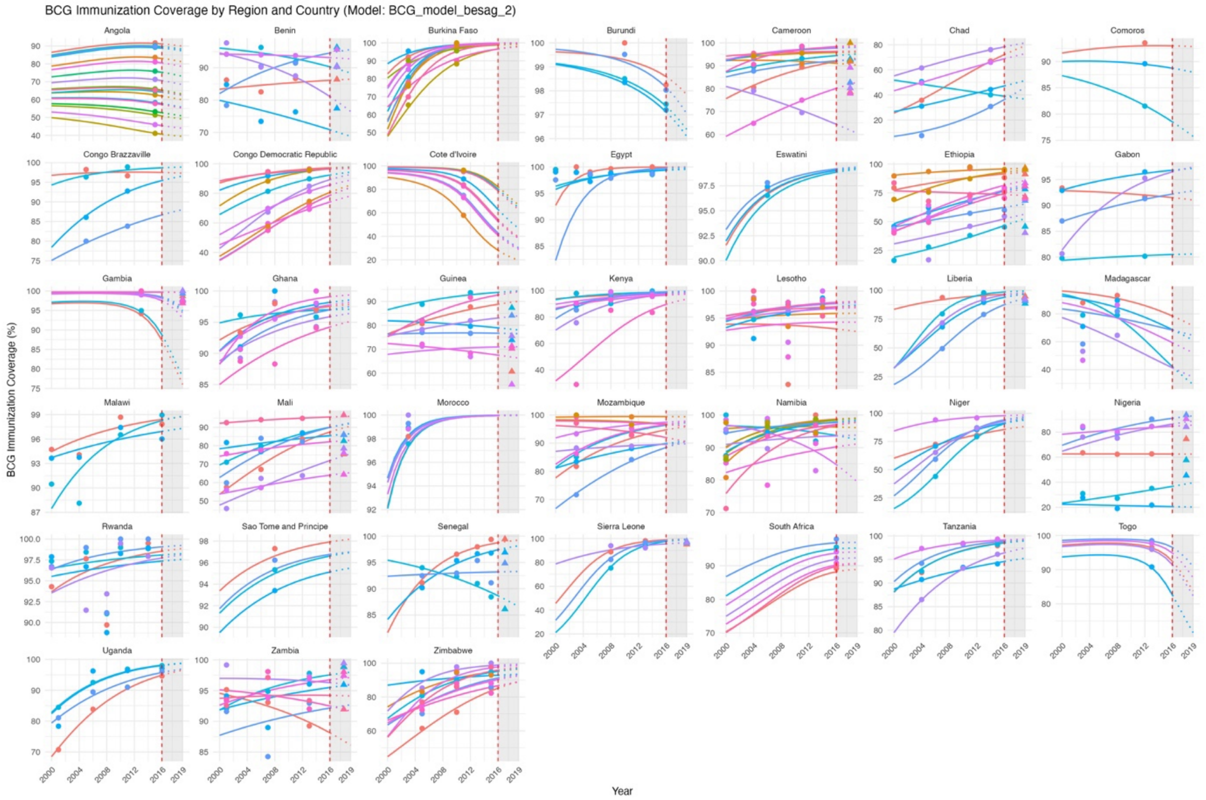

Fig A6. Trends in BCG immunization coverage at regional levels, using model_besag_2

Notes: The model was trained on data from 2000 to 2016 and used to predict coverage for 2017–2019. Red dotted vertical lines separate the model-fitting period (2000–2016) from the prediction period (2017–2019, shaded area). Round dots indicate observed regional coverage during the model-fitting period, while triangle markers represent observed data from 2017–2019, used for out-of-sample validation. Solid lines show estimated trends from 2000–2016; dashed lines represent model-based predictions for 2017–2019.

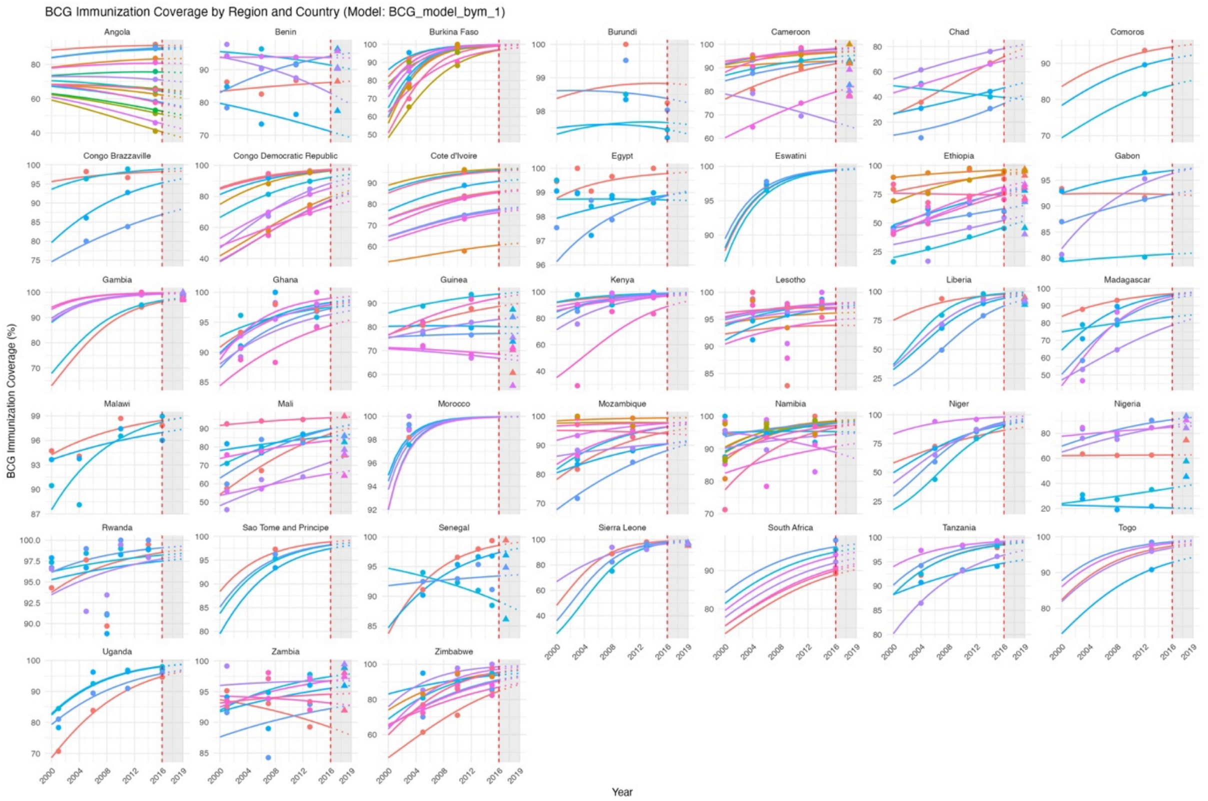

Fig A7. Trends in BCG immunization coverage at regional levels, using model_bym_1

Notes: The model was trained on data from 2000 to 2016 and used to predict coverage for 2017–2019. Red dotted vertical lines separate the model-fitting period (2000–2016) from the prediction period (2017–2019, shaded area). Round dots indicate observed regional coverage during the model-fitting period, while triangle markers represent observed data from 2017–2019, used for out-of-sample validation. Solid lines show estimated trends from 2000–2016; dashed lines represent model-based predictions for 2017–2019.

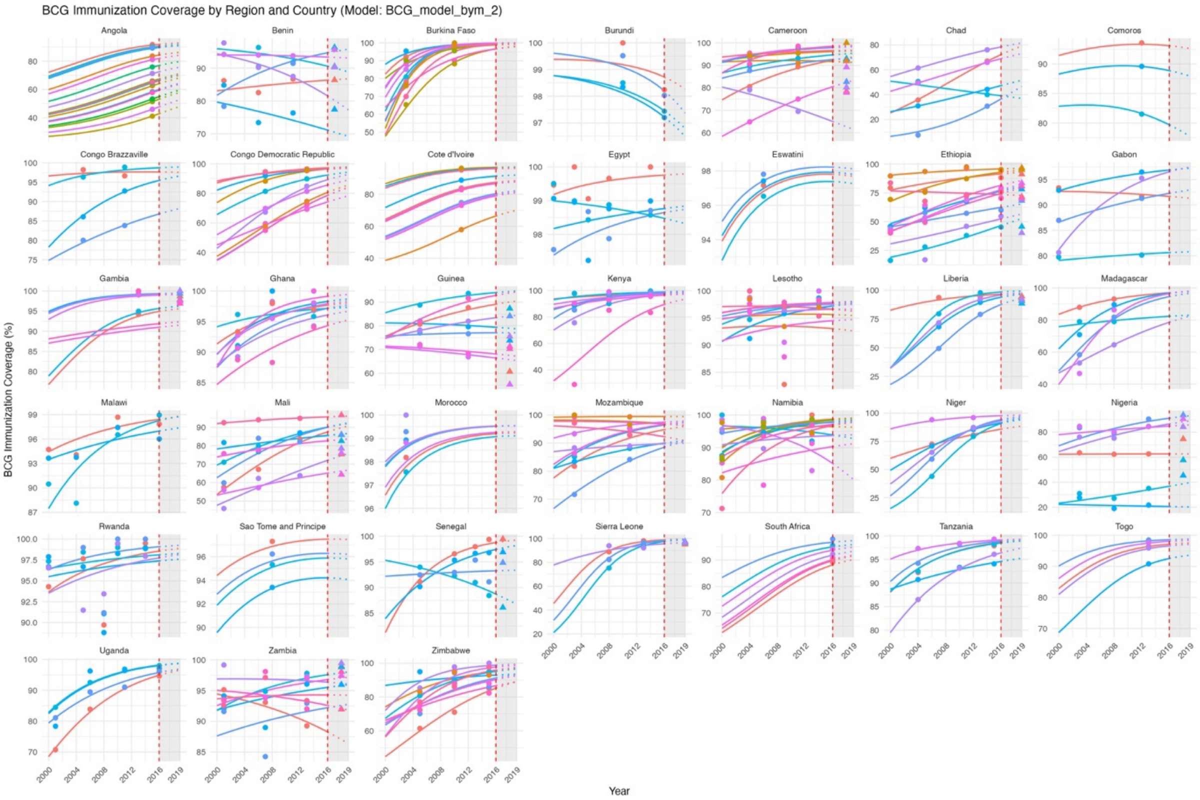

Fig A8. Trends in BCG immunization coverage at regional levels, using model_bym_2

Notes: The model was trained on data from 2000 to 2016 and used to predict coverage for 2017–2019. Red dotted vertical lines separate the model-fitting period (2000–2016) from the prediction period (2017–2019, shaded area). Round dots indicate observed regional coverage during the model-fitting period, while triangle markers represent observed data from 2017–2019, used for out-of-sample validation. Solid lines show estimated trends from 2000–2016; dashed lines represent model-based predictions for 2017–2019.

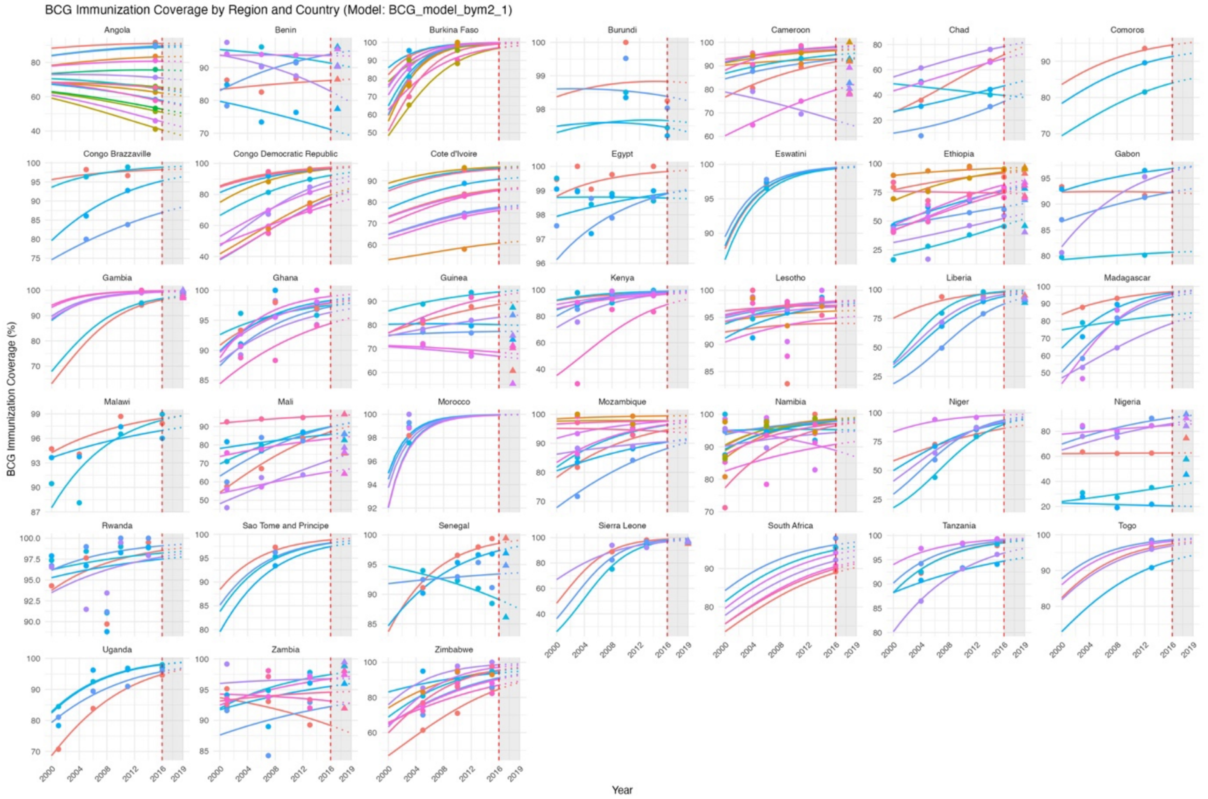

Fig A9. Trends in BCG immunization coverage at regional levels, using model_bym2_1

Notes: The model was trained on data from 2000 to 2016 and used to predict coverage for 2017–2019. Red dotted vertical lines separate the model-fitting period (2000–2016) from the prediction period (2017–2019, shaded area). Round dots indicate observed regional coverage during the model-fitting period, while triangle markers represent observed data from 2017–2019, used for out-of-sample validation. Solid lines show estimated trends from 2000–2016; dashed lines represent model-based predictions for 2017–2019.

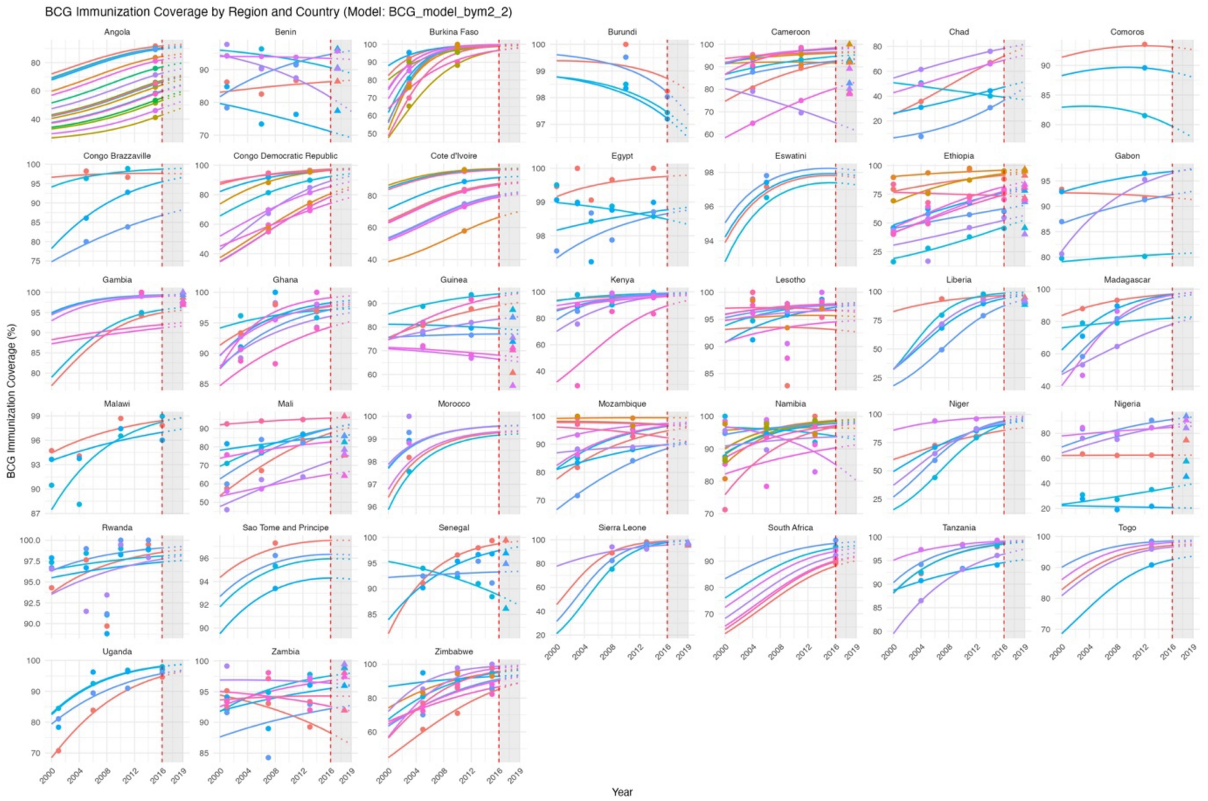

Fig A10. Trends in BCG immunization coverage at regional levels, using model_bym2_2

Notes: The model was trained on data from 2000 to 2016 and used to predict coverage for 2017–2019. Red dotted vertical lines separate the model-fitting period (2000–2016) from the prediction period (2017–2019, shaded area). Round dots indicate observed regional coverage during the model-fitting period, while triangle markers represent observed data from 2017–2019, used for out-of-sample validation. Solid lines show estimated trends from 2000–2016; dashed lines represent model-based predictions for 2017–2019.

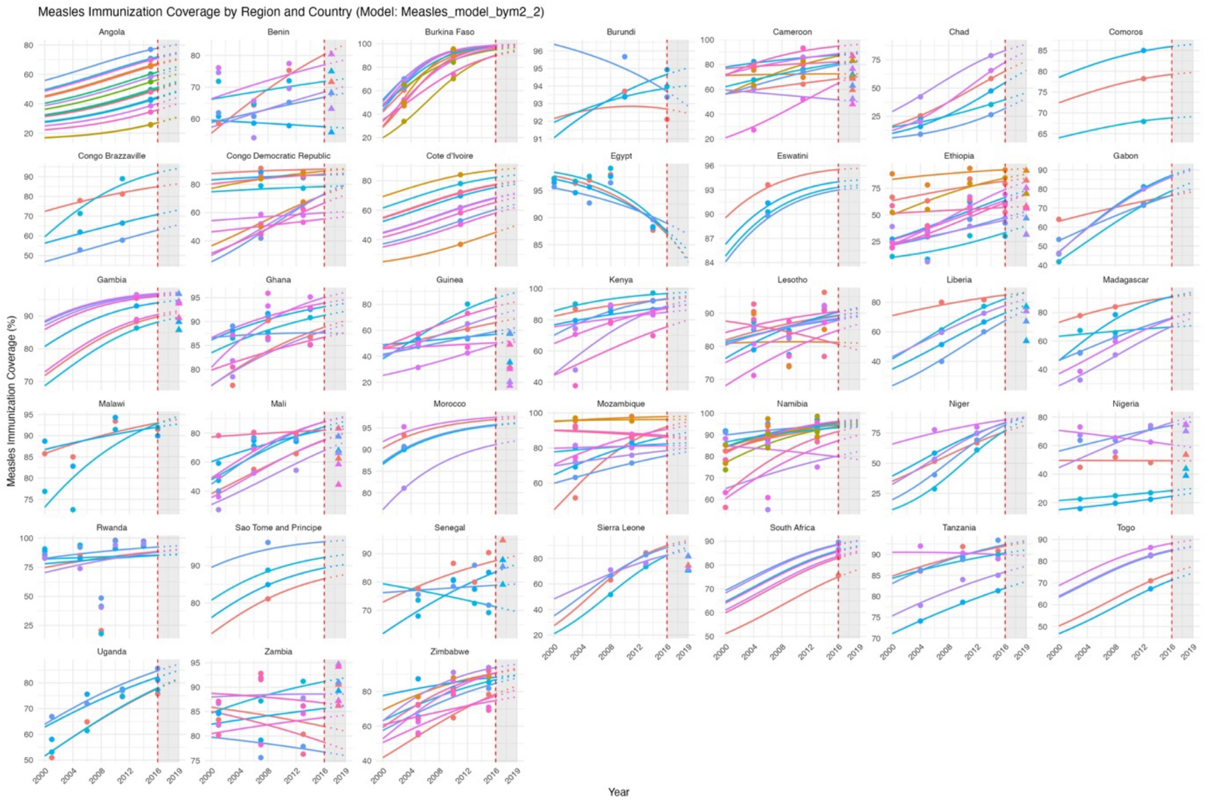

Fig A11. Trends in MCV1 immunization coverage at regional levels, using model_bym2_2

Notes: The model was trained on data from 2000 to 2016 and used to predict coverage for 2017–2019. Red dotted vertical lines separate the model-fitting period (2000–2016) from the prediction period (2017–2019, shaded area). Round dots indicate observed regional coverage during the model-fitting period, while triangle markers represent observed data from 2017–2019, used for out-of-sample validation. Solid lines show estimated trends from 2000–2016; dashed lines represent model-based predictions for 2017–2019.

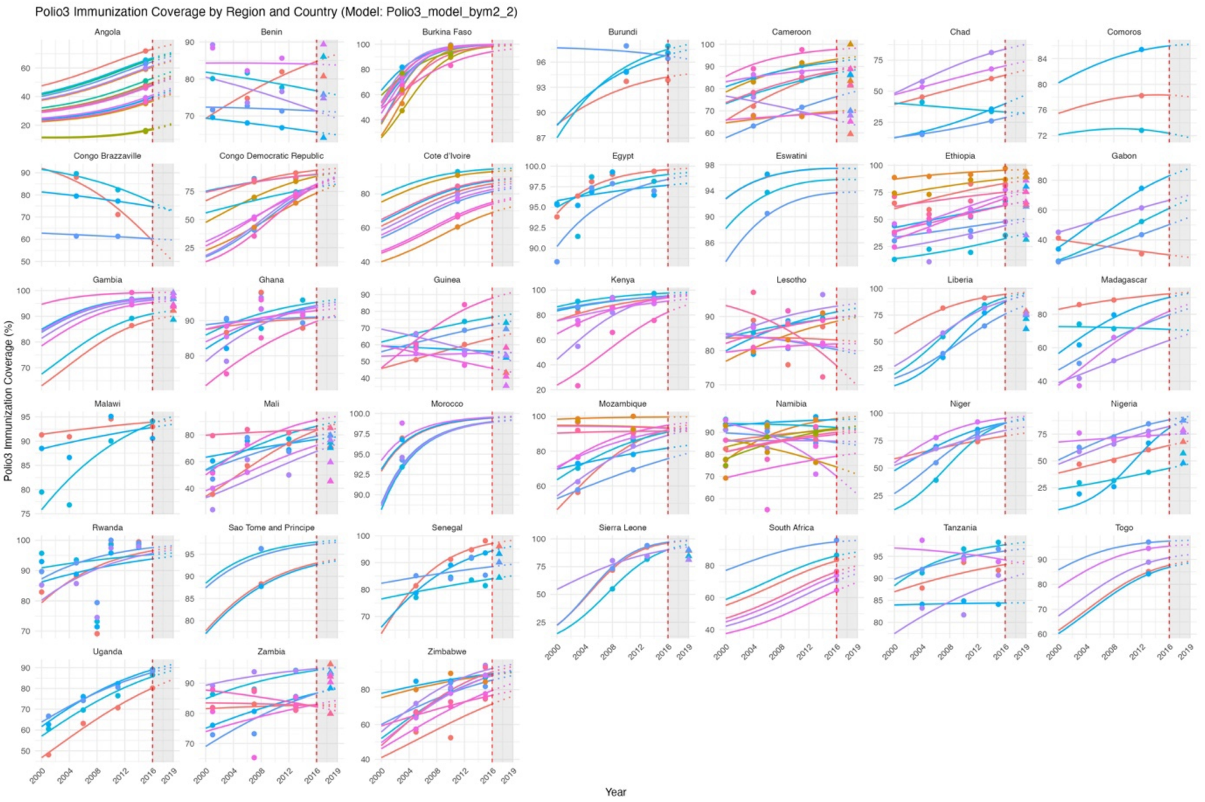

Fig A12. Trends in Polio3 immunization coverage at regional levels, using model_bym2_2

Notes: The model was trained on data from 2000 to 2016 and used to predict coverage for 2017–2019. Red dotted vertical lines separate the model-fitting period (2000–2016) from the prediction period (2017–2019, shaded area). Round dots indicate observed regional coverage during the model-fitting period, while triangle markers represent observed data from 2017–2019, used for out-of-sample validation. Solid lines show estimated trends from 2000–2016; dashed lines represent model-based predictions for 2017–2019.

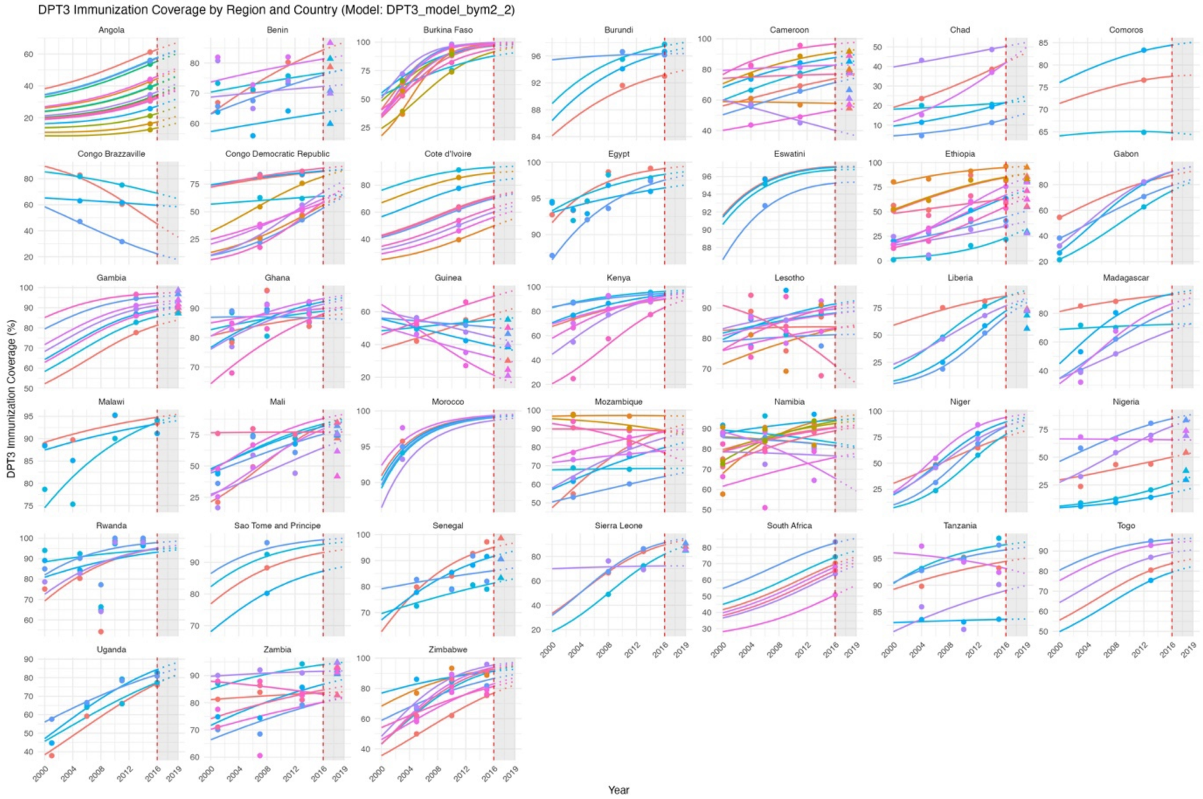

Fig A13. Trends in DPT3 immunization coverage at regional levels, using model_bym2_2

Notes: The model was trained on data from 2000 to 2016 and used to predict coverage for 2017–2019. Red dotted vertical lines separate the model-fitting period (2000–2016) from the prediction period (2017–2019, shaded area). Round dots indicate observed regional coverage during the model-fitting period, while triangle markers represent observed data from 2017–2019, used for out-of-sample validation. Solid lines show estimated trends from 2000–2016; dashed lines represent model-based predictions for 2017–2019.

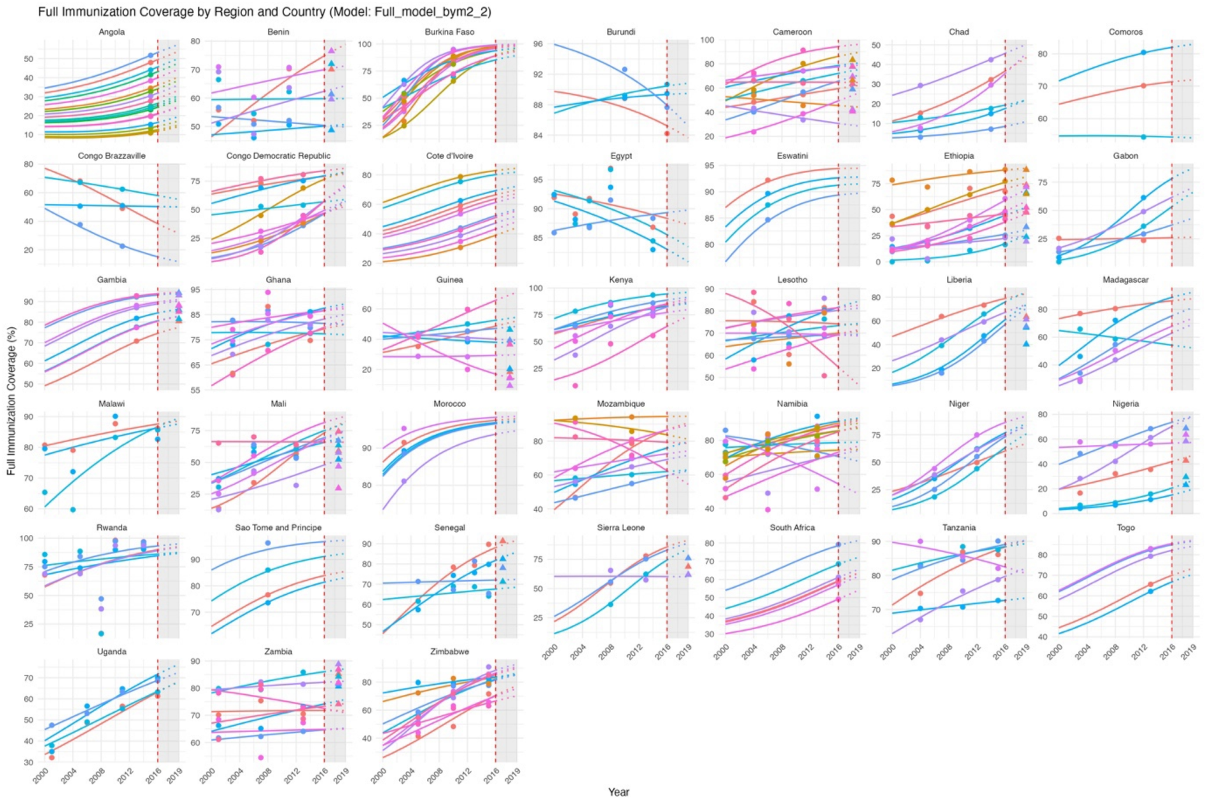

Fig A14. Trends in Full immunization coverage at regional levels, using model_bym2_2

Notes: The model was trained on data from 2000 to 2016 and used to predict coverage for 2017–2019. Red dotted vertical lines separate the model-fitting period (2000–2016) from the prediction period (2017–2019, shaded area). Round dots indicate observed regional coverage during the model-fitting period, while triangle markers represent observed data from 2017–2019, used for out-of-sample validation. Solid lines show estimated trends from 2000–2016; dashed lines represent model-based predictions for 2017–2019.

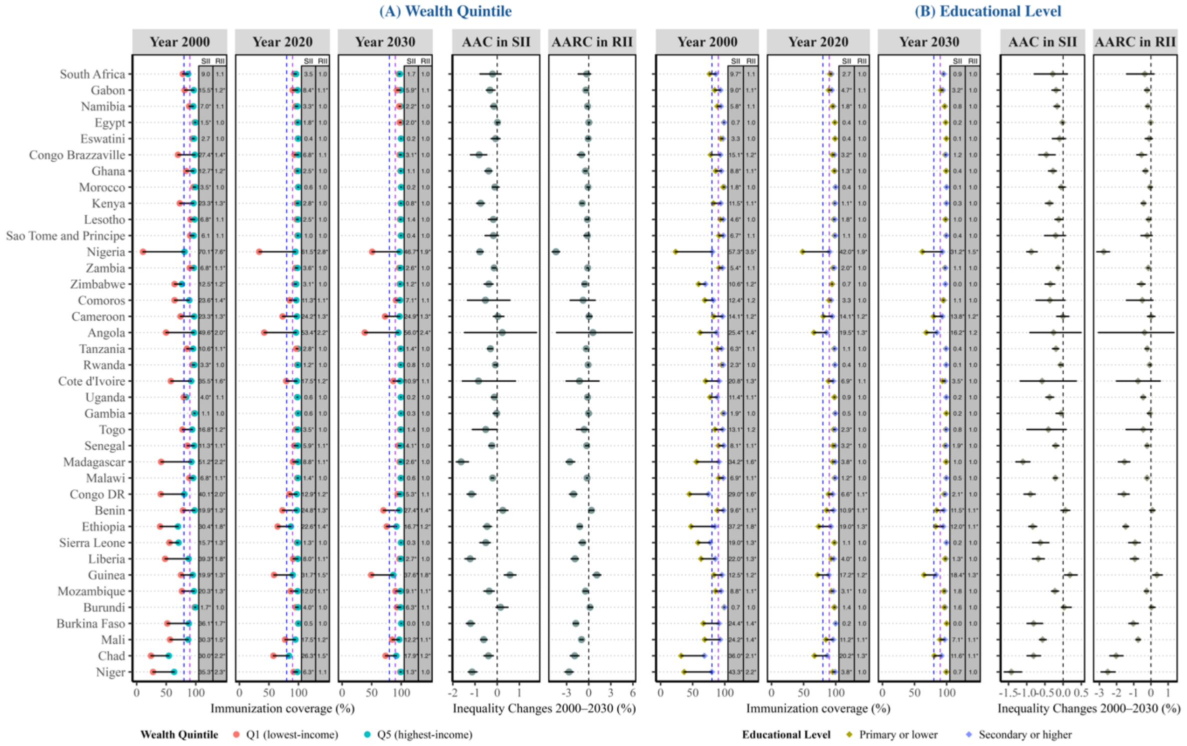

Fig A15. BCG coverage, inequality indices (SII, RII), and changes over 2000–2030 (AARC)

Notes: The Y-axis lists 38 African countries, ordered by Socio-Demographic Index (SDI) from highest (top) to lowest (bottom). Vertical dashed lines mark global targets: the blue line indicates the UHC benchmark (80% coverage), and the purple line indicates the IA2030 target (90% coverage). Abbreviations: SII = Slope Index of Inequality; RII = Relative Index of Inequality; AARC = Average Annual Rate of Change (2000–2030).

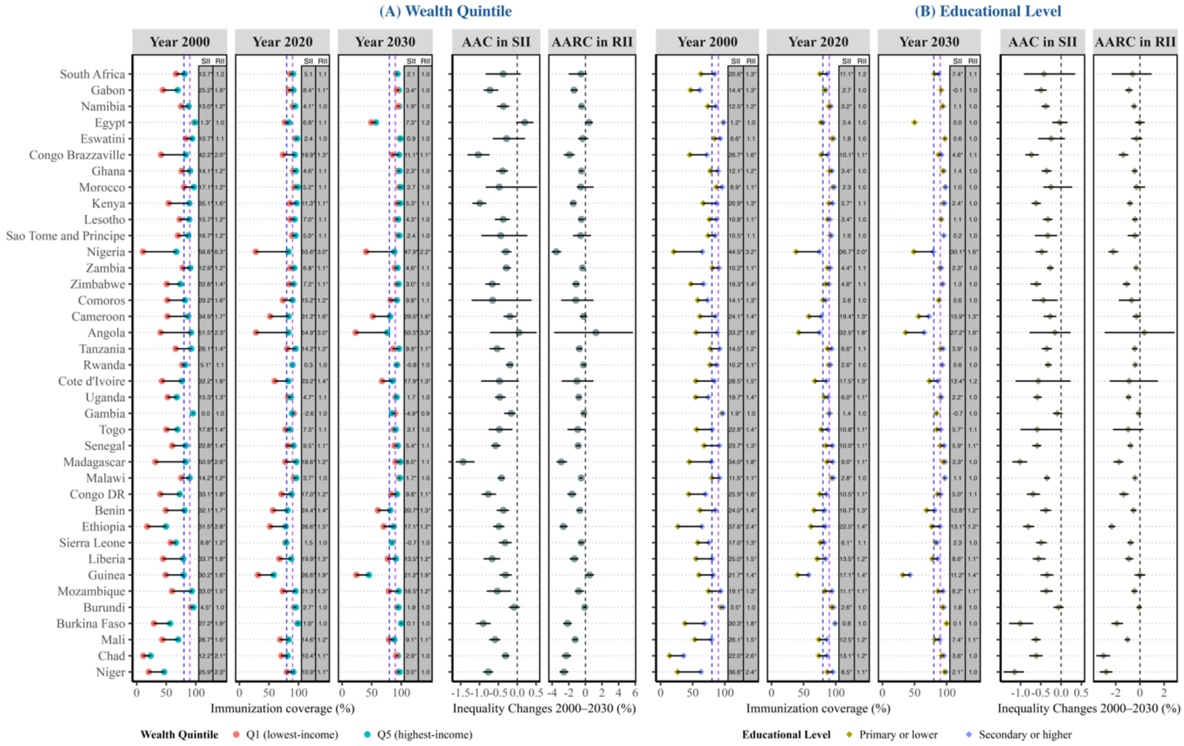

Fig A16. MCV1 coverage, inequality indices (SII, RII), and changes over 2000–2030 (AARC)

Notes: The Y-axis lists 38 African countries, ordered by Socio-Demographic Index (SDI) from highest (top) to lowest (bottom). Vertical dashed lines mark global targets: the blue line indicates the UHC benchmark (80% coverage), and the purple line indicates the IA2030 target (90% coverage). Abbreviations: SII = Slope Index of Inequality; RII = Relative Index of Inequality; AARC = Average Annual Rate of Change (2000–2030).

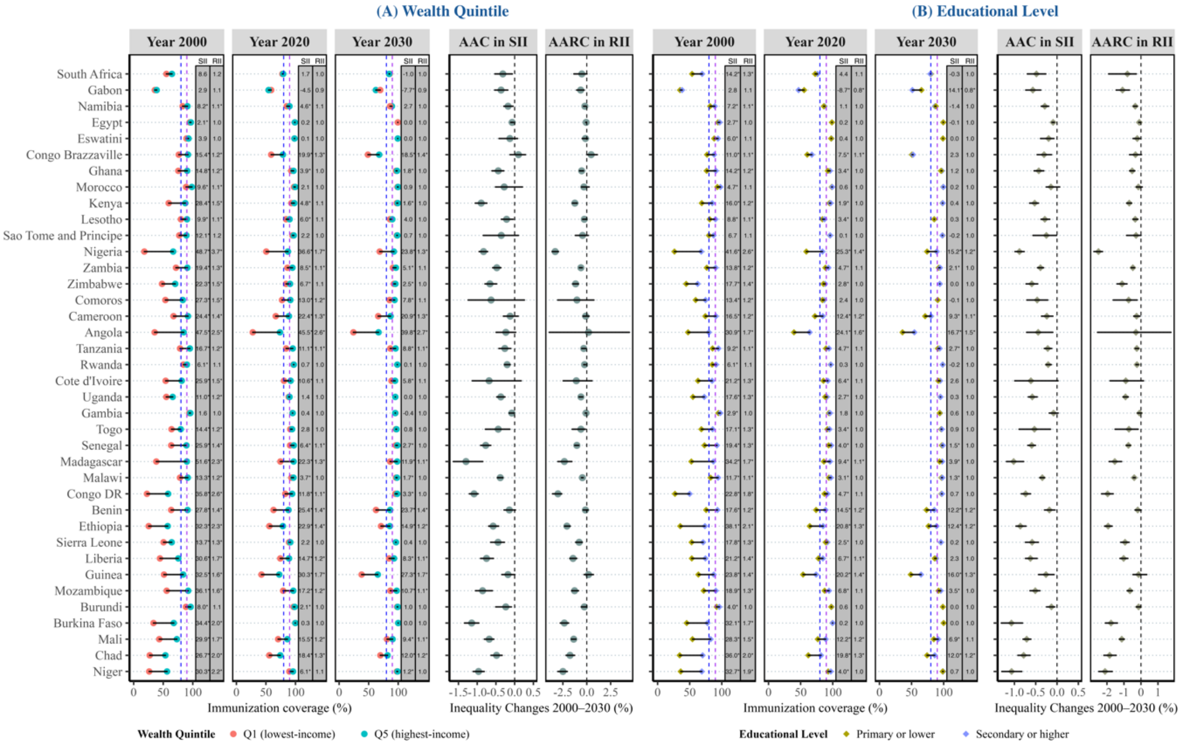

Fig A17. Polio3 coverage, inequality indices (SII, RII), and changes over 2000–2030 (AARC)

Notes: The Y-axis lists 38 African countries, ordered by Socio-Demographic Index (SDI) from highest (top) to lowest (bottom). Vertical dashed lines mark global targets: the blue line indicates the UHC benchmark (80% coverage), and the purple line indicates the IA2030 target (90% coverage). Abbreviations: SII = Slope Index of Inequality; RII = Relative Index of Inequality; AARC = Average Annual Rate of Change (2000–2030).

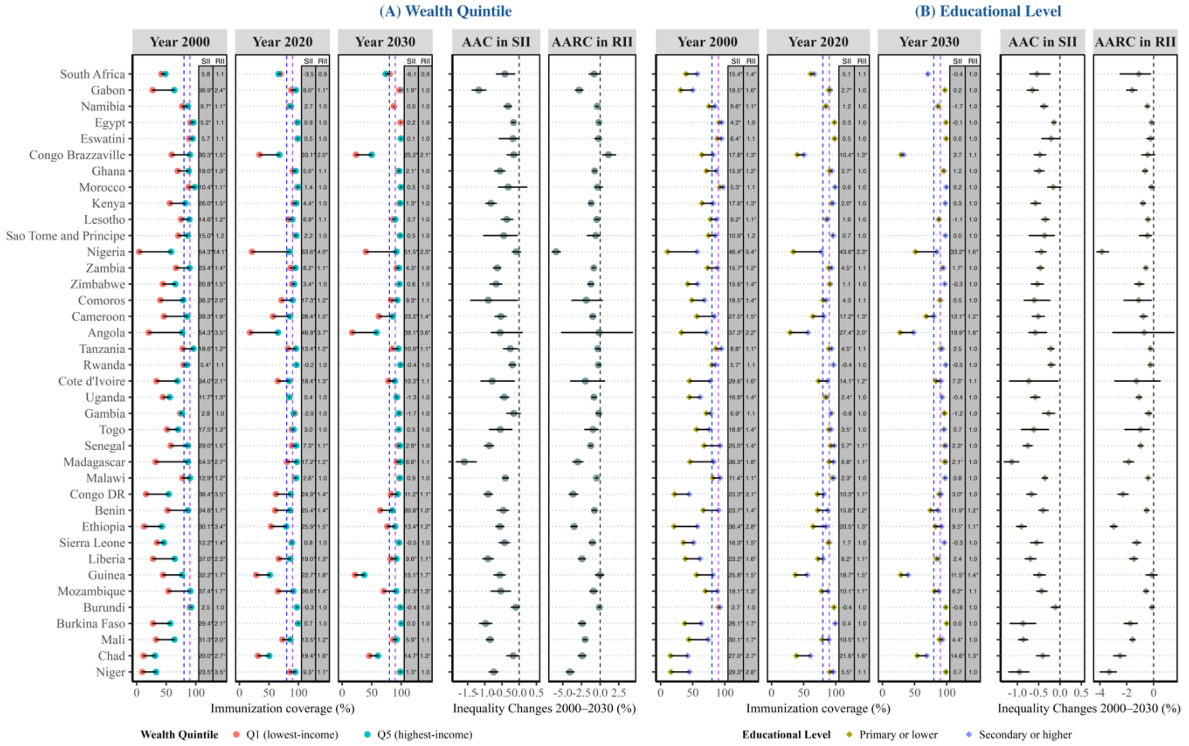

Fig A18. DPT3 coverage, inequality indices (SII, RII), and changes over 2000–2030 (AARC)

Notes: The Y-axis lists 38 African countries, ordered by Socio-Demographic Index (SDI) from highest (top) to lowest (bottom). Vertical dashed lines mark global targets: the blue line indicates the UHC benchmark (80% coverage), and the purple line indicates the IA2030 target (90% coverage). Abbreviations: SII = Slope Index of Inequality; RII = Relative Index of Inequality; AARC = Average Annual Rate of Change (2000–2030).

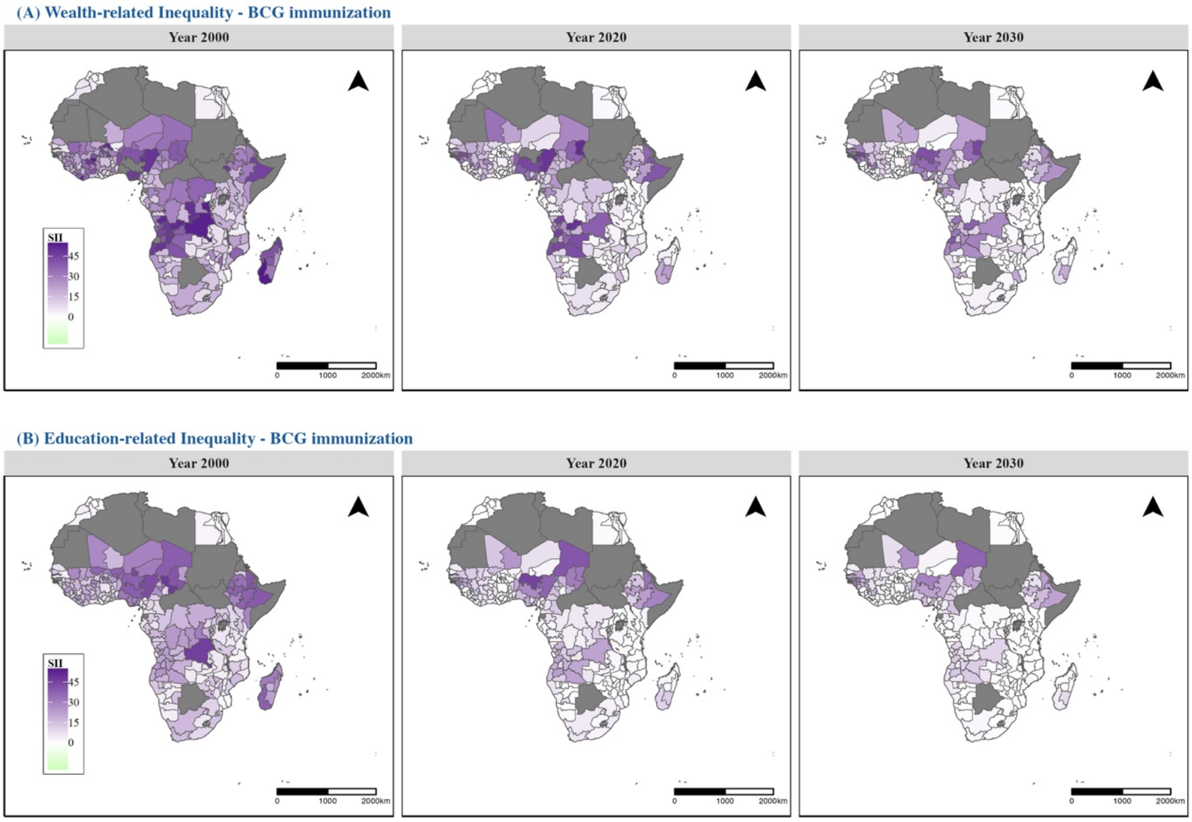

Fig A19. (A) Wealth- and (B) Education-related inequality in BCG coverage

Notes: Inequality is measured using the Slope Index of Inequality (SII). Countries shown in grey indicate data not available. Basemap from GADM (https://gadm.org/).

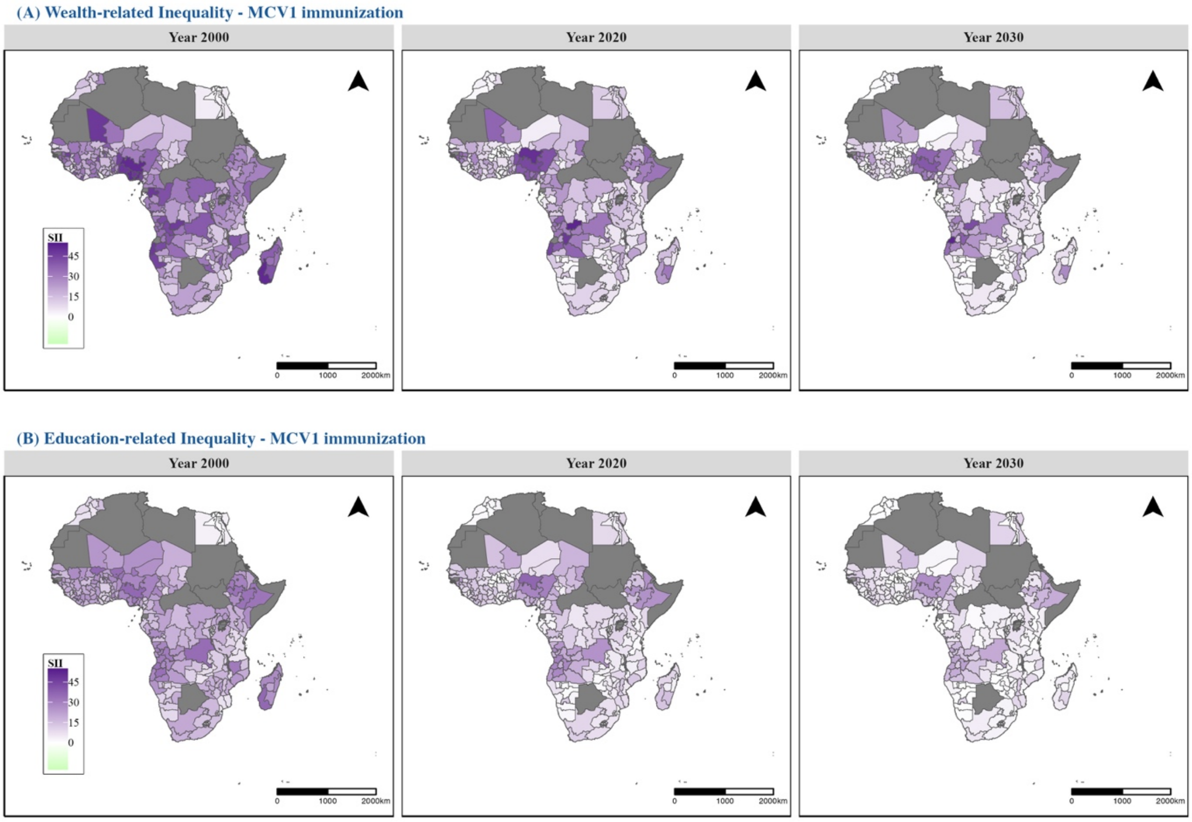

Fig A20. (A) Wealth- and (B) Education-related inequality in MCV1 coverage

Notes: Inequality is measured using the Slope Index of Inequality (SII). Countries shown in grey indicate data not available. Basemap from GADM (https://gadm.org/).

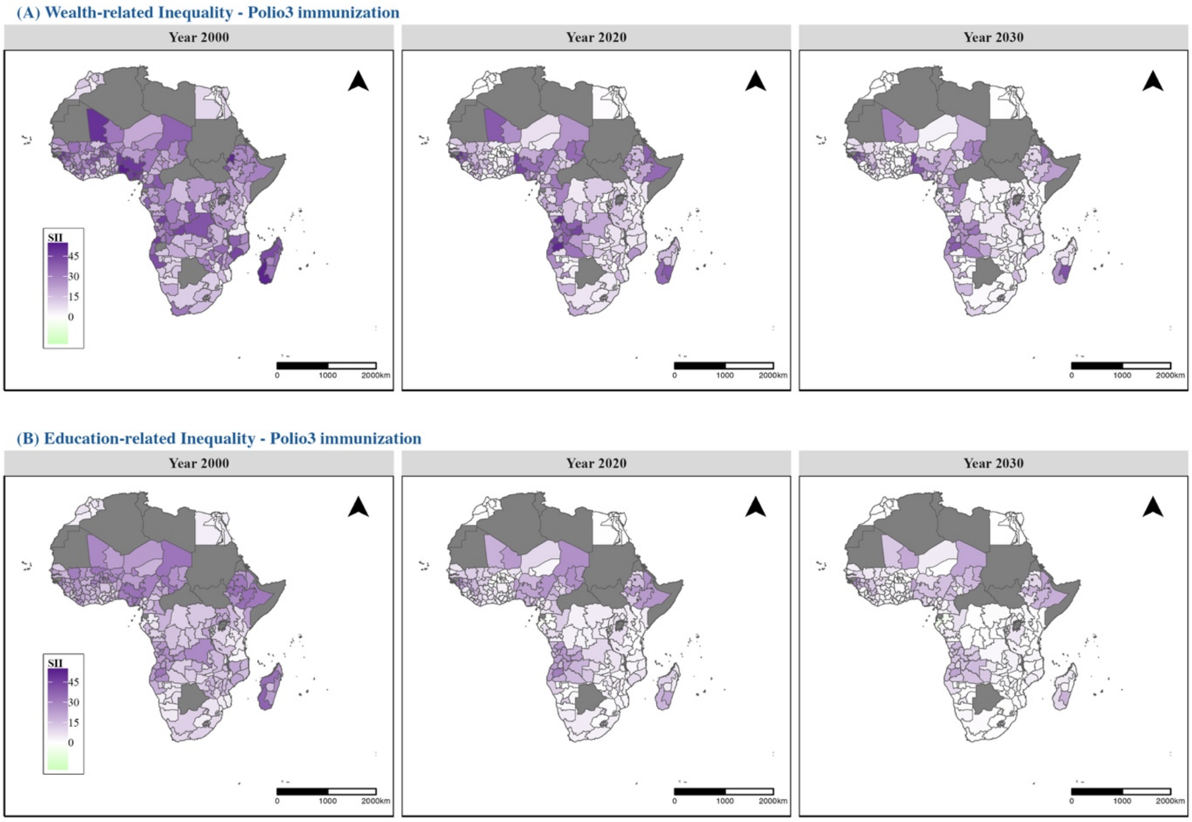

Fig A21. (A) Wealth- and (B) Education-related inequality in Polio3 coverage

Notes: Inequality is measured using the Slope Index of Inequality (SII). Countries shown in grey indicate data not available. Basemap from GADM (https://gadm.org/).

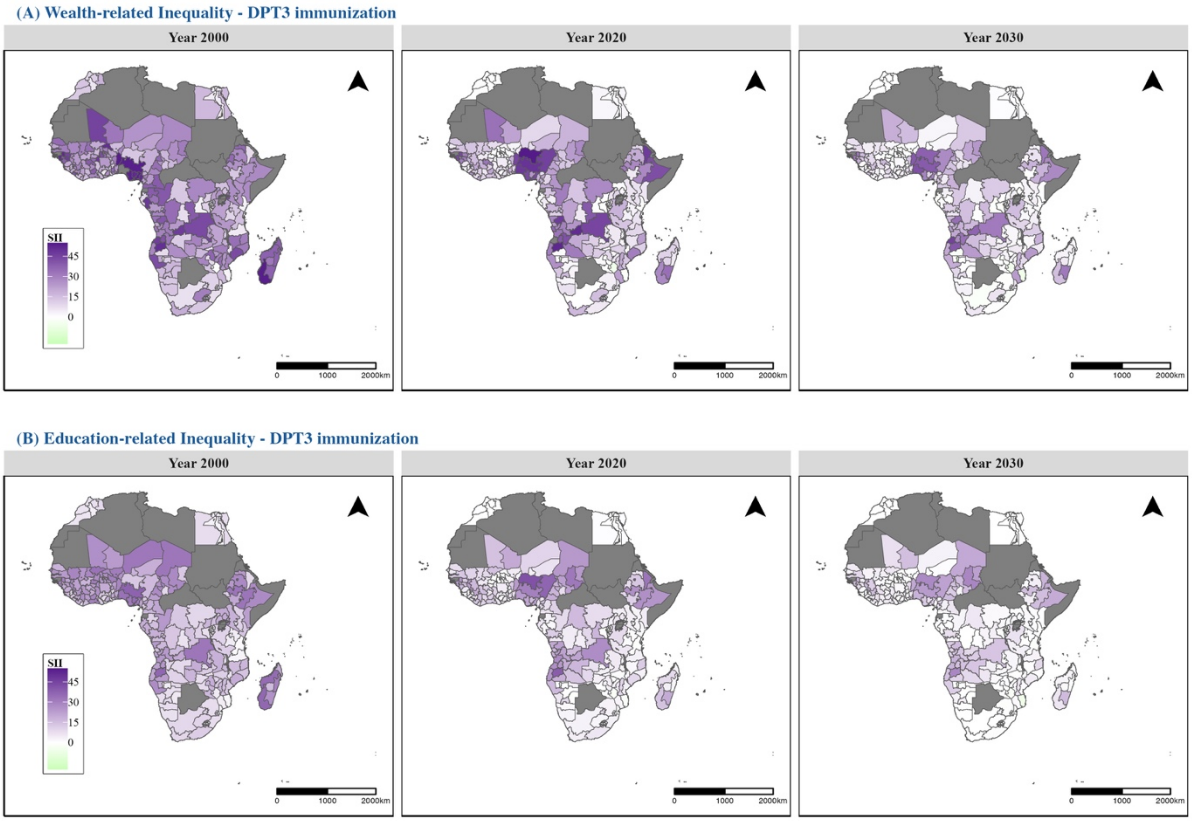

Fig A22. (A) Wealth- and (B) Education-related inequality in DPT3 coverage

Notes: Inequality is measured using the Slope Index of Inequality (SII). Countries shown in grey indicate data not available. Basemap from GADM (https://gadm.org/).

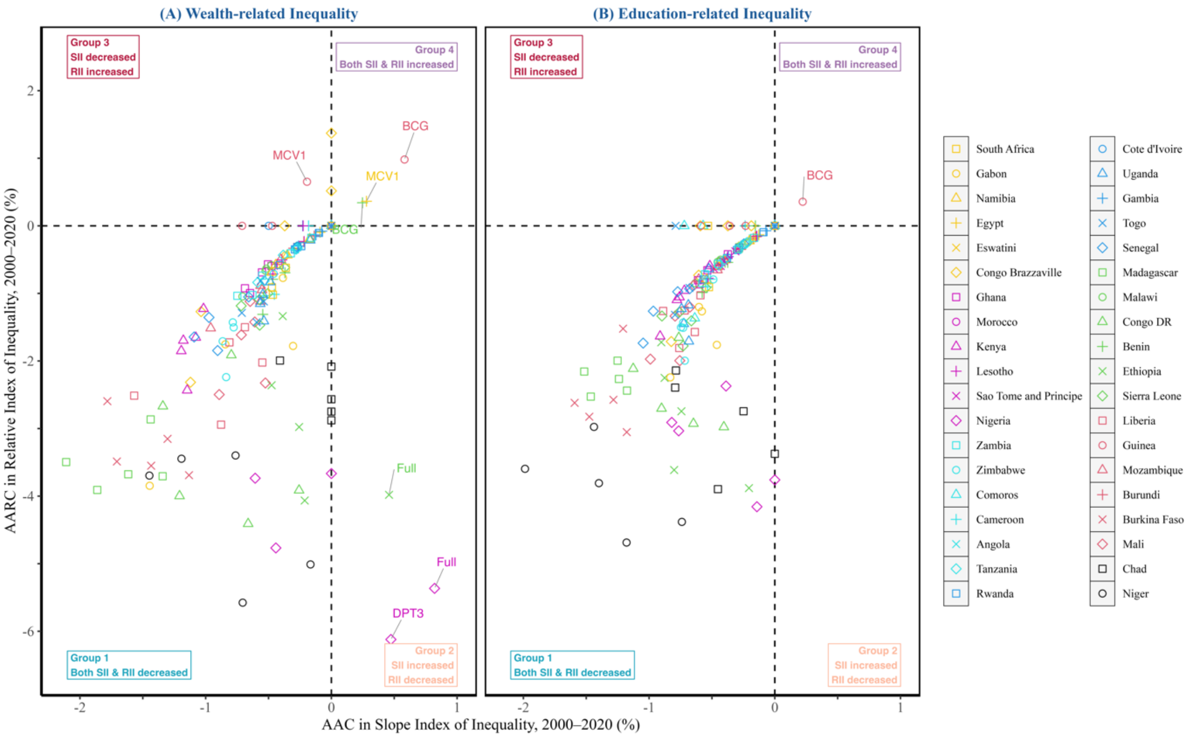

Fig A23. Two-dimensional graph of changes in socioeconomic-related inequalities, 2000–2020

Notes: The analysis includes five immunization indicators: Full Immunization, BCG, MCV1, DPT3, and Polio3.

Abbreviations: AARC = Average Annual Rate of Change; AAC = Absolute Annual Change; SII = Slope Index of Inequality; RII = Relative Index of Inequality.

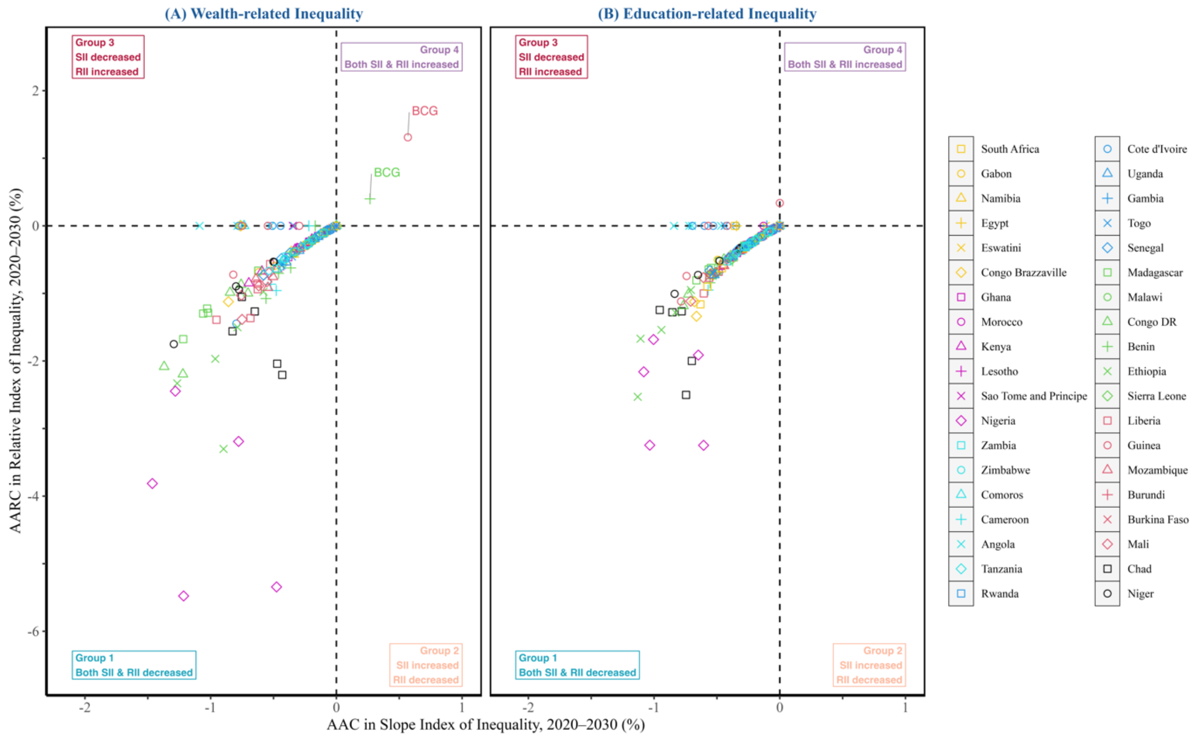

Fig A24. Two-dimensional graph of changes in socioeconomic-related inequalities, 2020–2030

Notes: The analysis includes five immunization indicators: Full Immunization, BCG, MCV1, DPT3, and Polio3.
Abbreviations: AARC = Average Annual Rate of Change; AAC = Absolute Annual Change; SII = Slope Index of Inequality; RII = Relative Index of Inequality.

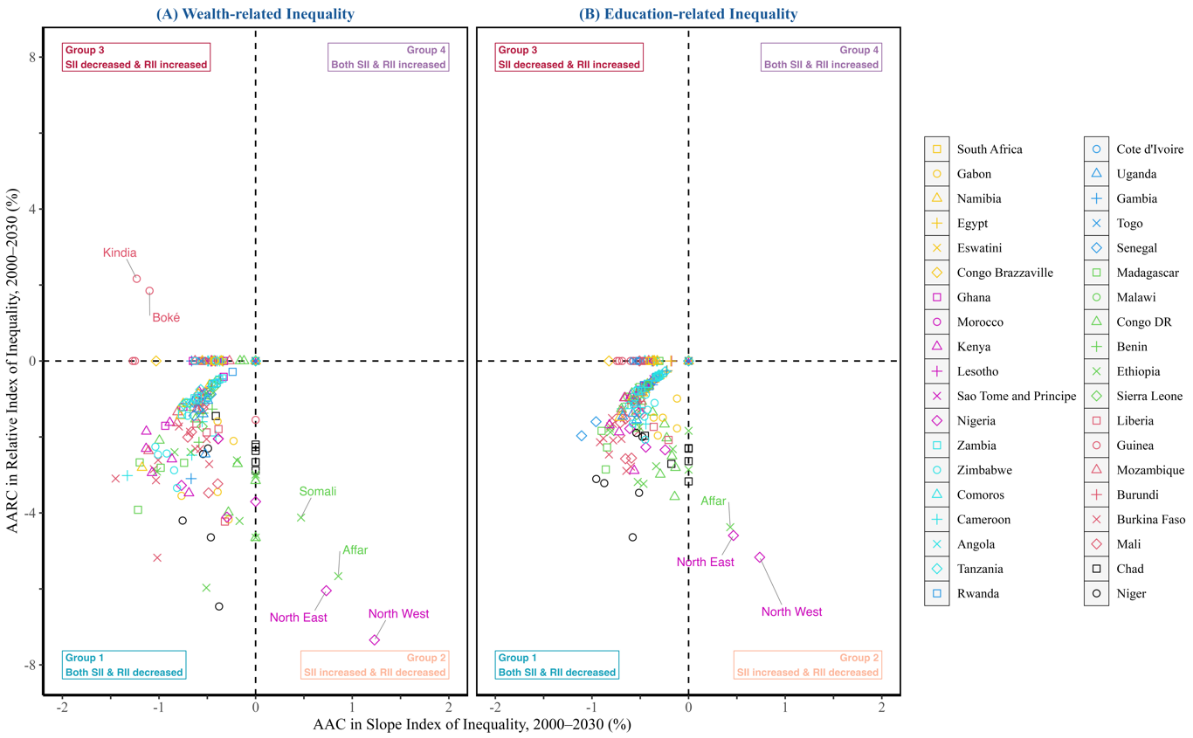

Fig A25. Inequality changes in Full immunization coverage (2000–2030) with region names

Notes: AARC = Average Annual Rate of Change; AAC = Absolute Annual Change; SII = Slope Index of Inequality; RII = Relative Index of Inequality.

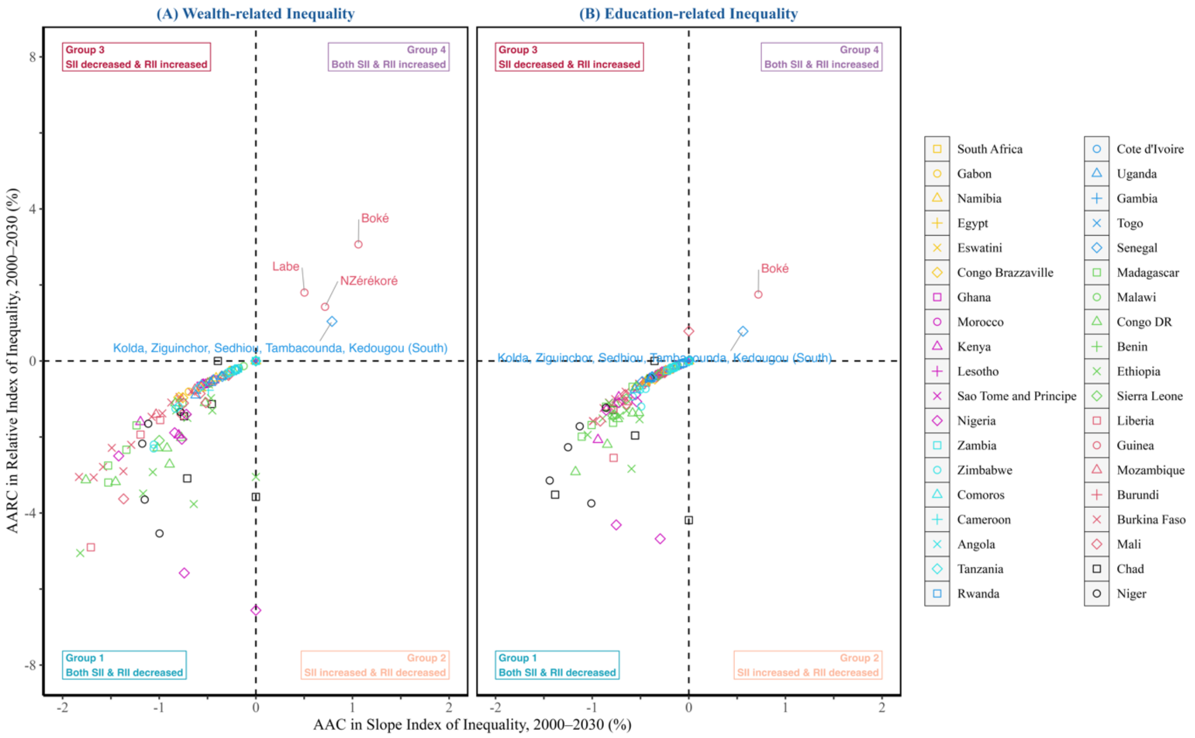

Fig A26. Inequality changes in BCG immunization coverage (2000–2030) with region names

Notes: AARC = Average Annual Rate of Change; AAC = Absolute Annual Change; SII = Slope Index of Inequality; RII = Relative Index of Inequality.

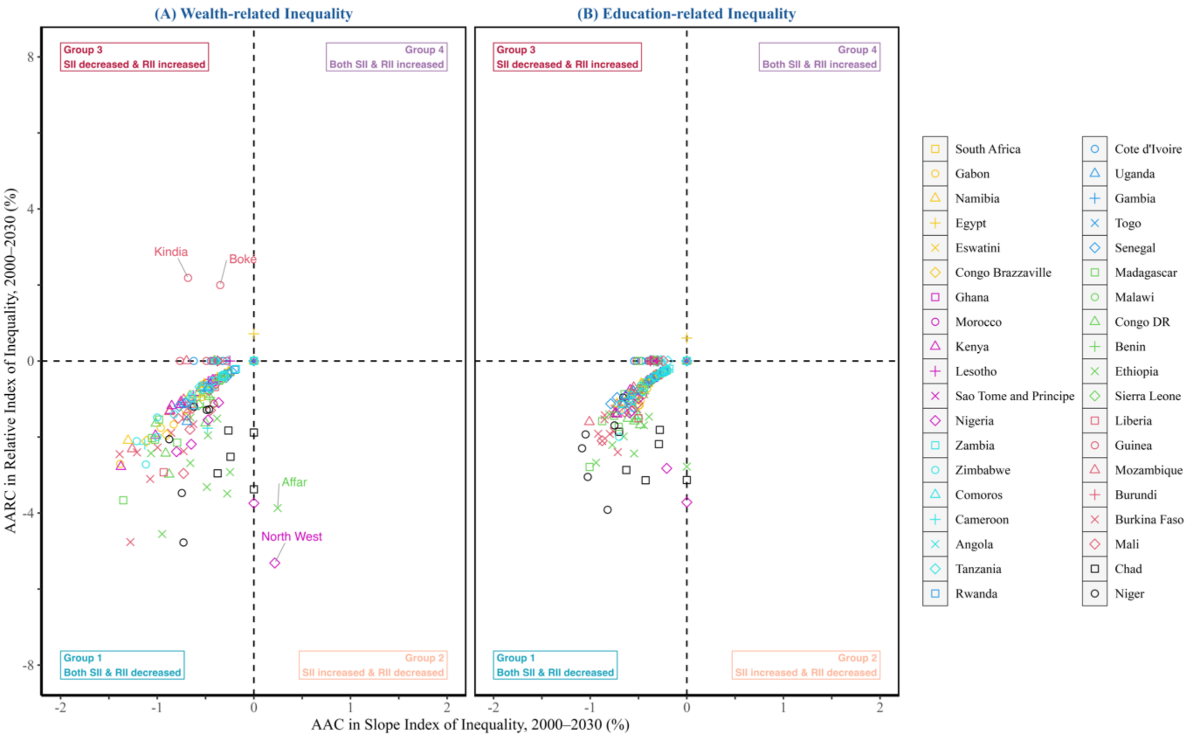

Fig A27. Inequality changes in MCV1 immunization coverage (2000–2030) with region names

Notes: AARC = Average Annual Rate of Change; AAC = Absolute Annual Change; SII = Slope Index of Inequality; RII = Relative Index of Inequality.

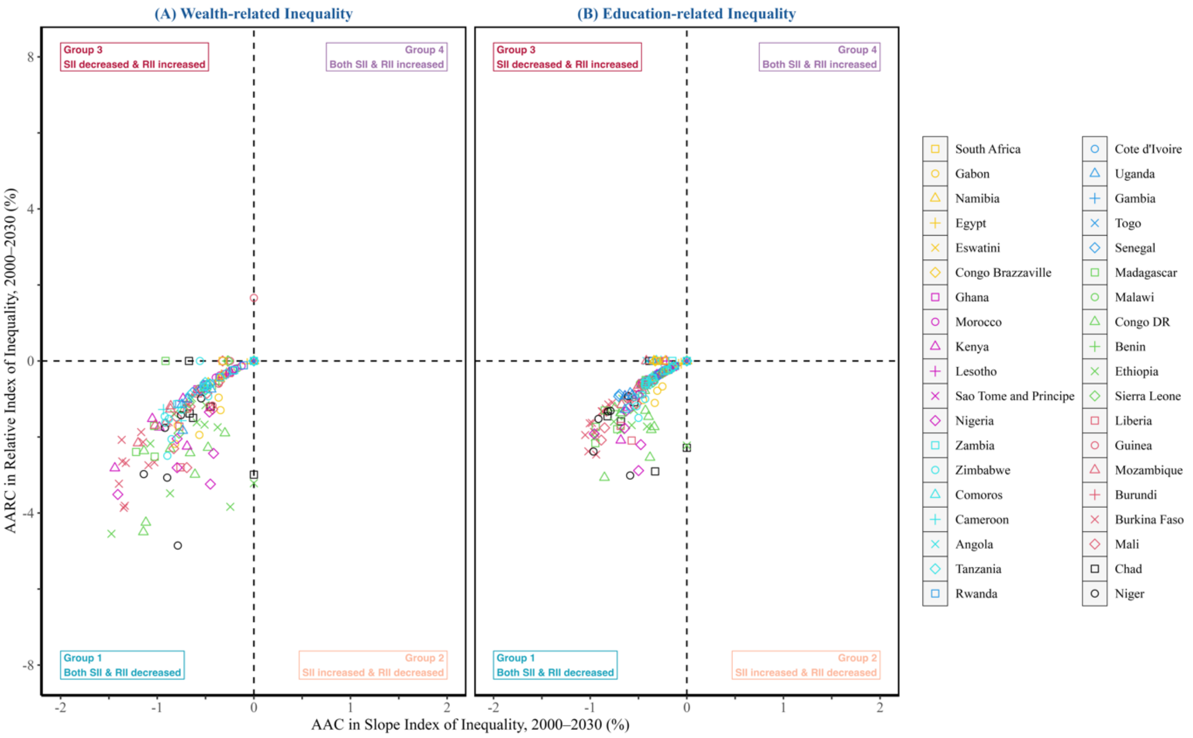

Fig A28. Inequality changes in Polio3 immunization coverage (2000–2030) with region names

Notes: AARC = Average Annual Rate of Change; AAC = Absolute Annual Change; SII = Slope Index of Inequality; RII = Relative Index of Inequality.

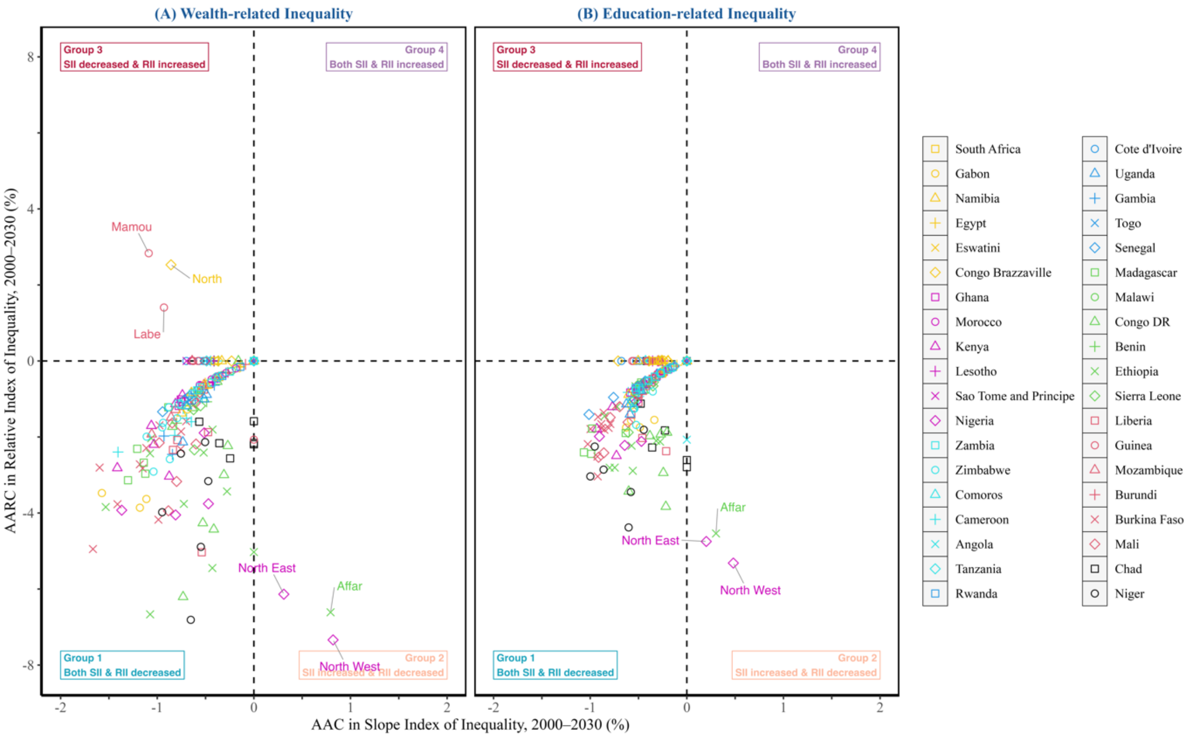

Fig A29. Inequality changes in DPT3 immunization coverage (2000–2030) with region names

Notes: AARC = Average Annual Rate of Change; AAC = Absolute Annual Change; SII = Slope Index of Inequality; RII = Relative Index of Inequality.

(A) Coverage in 2030 - Full Immunization

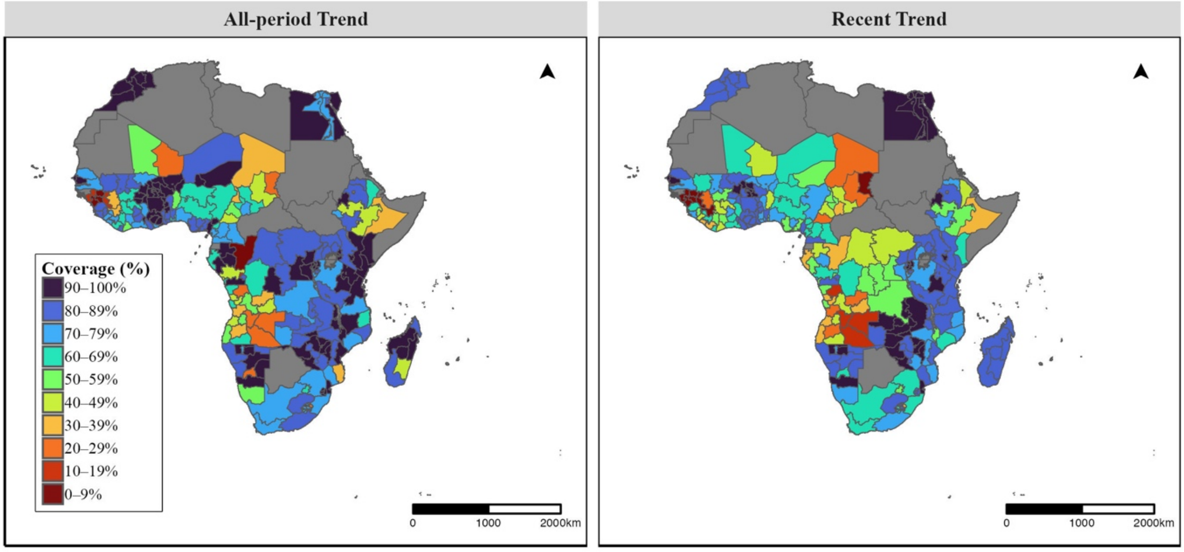

(B) AARC over 2020–2030 - Full Immunization

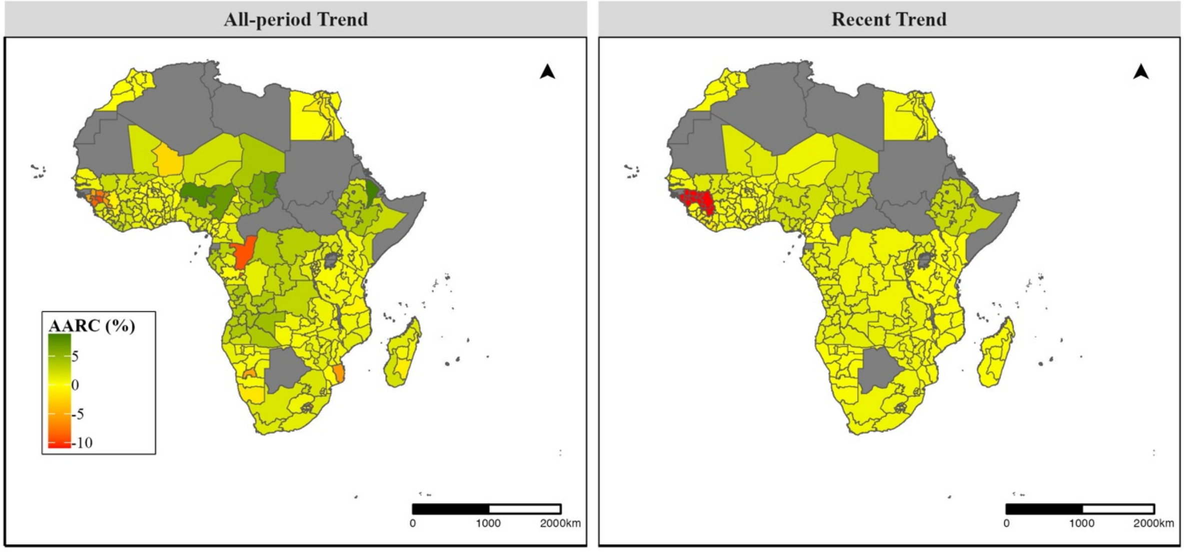

Fig A30. Sensitivity analysis of using recent trend for projections of Full immunization coverage

Notes: All-period trend: Based on overall trends from 2000–2019; Recent trend: Based on more recent trends from 2010–2019. Countries shown in grey indicate data not available. Basemap from GADM (https://gadm.org/). AARC=Average Annual Rate of Change;

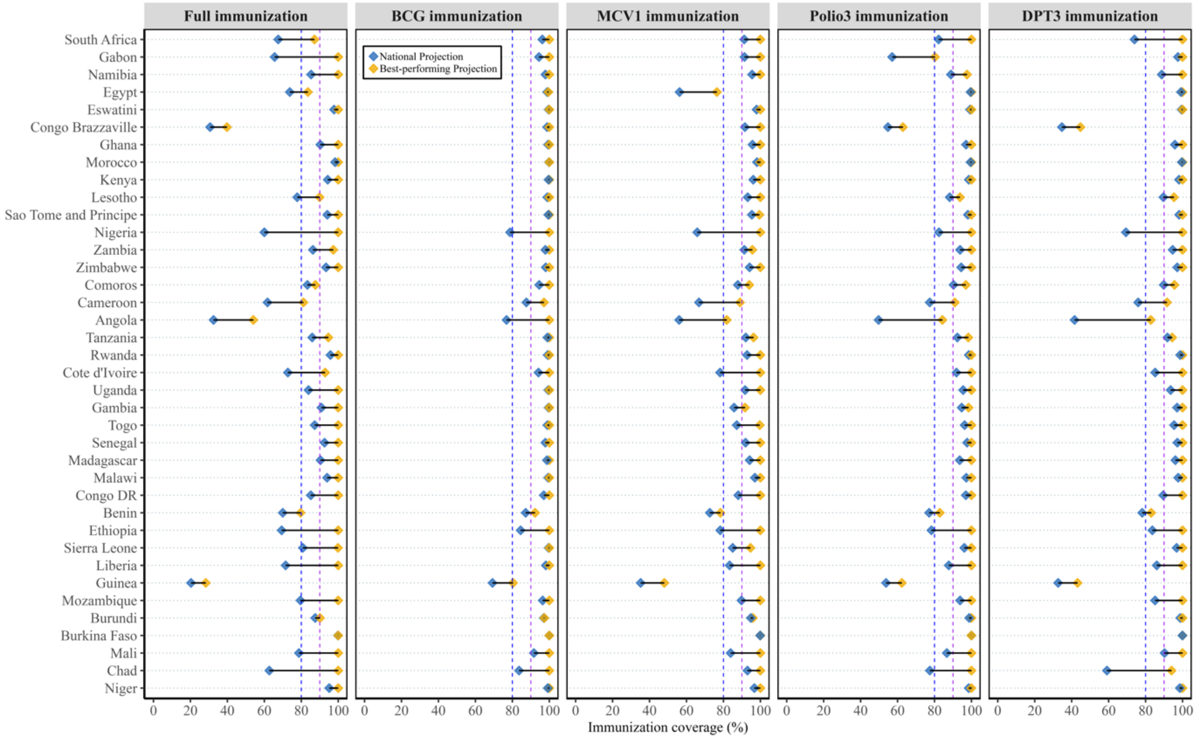

Fig A31. Sensitivity analysis of using AARC of best-performing region for 2030 national projections

Notes: AARC=Average Annual Rate of Change;

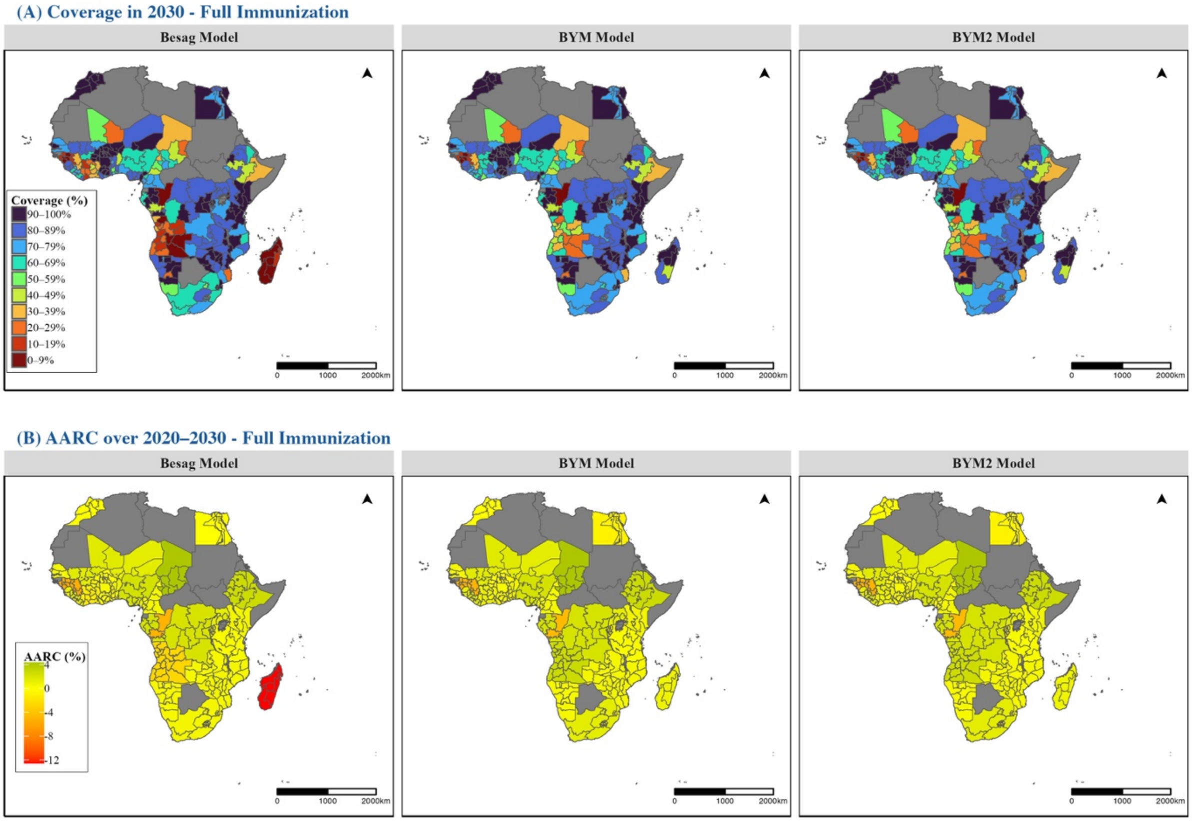

Fig A32. Sensitivity analysis of model selection for projections of Full immunization coverage

Notes: The tested Besag, BYM, and BYM2 models are described in details in Supplementary methods as besag_2, bym_2, and bym2_2 models. Countries shown in grey indicate data not available. Basemap from GADM (https://gadm.org/). AARC=Average Annual Rate of Change;

References:

1. World Health Organization. 2019 Global Monitoring Report: Primary Health Care on the Road to Universal Health Coverage. Geneva, Switzerland: World Health Organization, 2019.
2. Gelfand AE, Diggle P, Guttorp P, Fuentes M. Handbook of Spatial Statistics (1st ed.): CRC Press; 2010.
3. Alexander N. Bayesian Disease Mapping: Hierarchical Modeling in Spatial Epidemiology. *Journal of the Royal Statistical Society Series A: Statistics in Society* 2011; **174**(2): 512-3.
4. Martínez-Beneito MA, López-Quilez A, Botella-Rocamora P. An autoregressive approach to spatio-temporal disease mapping. *Statistics in Medicine* 2008; **27**(15): 2874-89.
5. Ugarte MD, Adin A, Goicoa T, Militino AF. On fitting spatio-temporal disease mapping models using approximate Bayesian inference. *Stat Methods Med Res* 2014; **23**(6): 507-30.
6. Bernardinelli L, Clayton D, Pascutto C, Montomoli C, Ghislandi M, Songini M. Bayesian analysis of space-time variation in disease risk. *Stat Med* 1995; **14**(21-22): 2433-43.
7. Besag J, York J, Mollié A. Bayesian image restoration, with two applications in spatial statistics. *Annals of the Institute of Statistical Mathematics* 1991; **43**(1): 1-20.
8. Simpson D, Rue H, Riebler A, Martins TG, Sørbye SH. Penalising Model Component Complexity: A Principled, Practical Approach to Constructing Priors. *Statistical Science* 2017; **32**(1): 1-28, .
