# Supplementary material for: Progress and inequality in child immunization in 38 African countries, 2000–2030: A spatio-temporal Bayesian analysis at national and sub-national levels
Source: PLoS Med. 2025 Jul 29;22(7):e1004664. doi: 10.1371/journal.pmed.1004664 (PMC12306735; doi:10.1371/journal.pmed.1004664)
Supplement: Attachment — Submitted filename: PlosM_Response to Reviewers_20250530.pdf [file pmed.1004664.s006.pdf]

Dear Editor and Reviewers,

Thank you very much for the opportunity to revise our manuscript entitled *" Progress and inequality in child immunization in 38 African countries, 2000-2030: A spatio-temporal Bayesian analysis at national and sub-national levels"*.

We are also sincerely grateful to you and the reviewers for the insightful and constructive comments, which have greatly helped us improve the quality and clarity of our work.

We have carefully addressed all the comments and suggestions. Revisions are marked using the track changes function in the revised manuscript, as requested. In addition, we provide detailed, point-by-point responses to each reviewer comment below.

We truly appreciate the valuable feedback and hope that the revised version of the manuscript meets your expectations.

Yours sincerely,
The Authors

EDITORIAL COMMENTS:

Comments from the academic editor: Please revise to address the concerns of R2 and R3.

RESPONSE: Thank you for your guidance. We have revised the manuscript to fully address the concerns raised by Reviewers 2 and 3. This includes clarifying methods, expanding validation, and refining the interpretation of projections and inequalities. All changes are reflected in the revised manuscript and detailed in our point-by-point responses.
Please let us know if any further revisions are needed.

REVIEWER #1 COMMENTS:

Comments to the Author: The authors explained their design well and gave good justifications for their model choices. INLA is an appropriate estimation method. The Bayesian models used are established techniques for spatial analysis and the Bayesian approach gave intuitive probability estimates. The full details on the models were in the supplementary. The authors included some residual checks, although some were on an absolute scale. There were extensive sensitivity analyses and additional details in the supplementary.
The main figures look good and are informative. They are relatively dense, but they give details on all countries. Close inspection shows some interesting differences, for example Chad and Burkina Faso, which are close on the socio-demographic index, but with very different predictions.

RESPONSE: We sincerely thank the reviewer for the insightful and encouraging comments. Below, we provide detailed responses to your specific comments and suggestions.

1) **Comment 1:** Why was model bym_2 chosen when bym_1 had a smaller DIC and WAIC (Table S3)? Was the decision purely made on the absolute difference?

RESPONSE: Thank you for this important question. Although model_bym_1 had slightly lower DIC and WAIC values, we selected model_bym_2 based on a broader set of validation criteria. The differences in DIC and WAIC were minimal, while model_bym_2 showed better performance in terms of average absolute difference and mean error. Importantly, model_bym_2 features a nested spatial structure, modelling region-level effects within nation-level effects, which better reflects the hierarchical spatial relationships inherent in our multi-country data.

As detailed in the revised Methods and Supplementary Modelling sections, we re-ran model comparisons across six candidates using a comprehensive validation framework. This included in-sample (2000–2016) and out-of-sample (2017–2019) validation using DIC, WAIC, average absolute difference, mean error, and runtime performance. These updated comparisons are presented in Supplementary Table S3 and Figures S1–S14. Model_bym2_2 has been selected as the final model based on its consistently strong performance across all metrics, its improved interpretability, and its ability to account for both within- and between-region spatial dependencies under a hierarchical framework.

- **Revised Manuscript – Methods – Statistical Analysis:** "Model comparison and selection were conducted using data from 2000 to 2016, with 2017–2019 held out for out-of-sample validation. We assessed model performance using Deviance Information Criterion (DIC), Watanabe-Akaike Information Criterion (WAIC), Absolute Difference, Mean Error, and computational efficiency (runtime in seconds), evaluating each model on in-sample (2000–2016), out-of-sample (2017–2019), and full-period (2000–2019) performance. Among the six model variations, we selected model_bym2_2, a hierarchical BYM2 model that captures within- and between-region spatial dependencies

and accounts for national-level structure. It demonstrated the best overall performance across all immunization indicators based on DIC, WAIC, average absolute difference, and mean error. Its nested spatial structure also enhanced interpretability, aligning well with the hierarchical country–region relationships in the data. Details of model comparison and validation are presented in Supplementary Table S3 and Figures S1–S14."

2) **Comment 2:** Figure 5, try the ggrepel package to move the labels.

RESPONSE: We appreciate the valuable suggestion. We have implemented the ggrepel package to improve label placement in Figure 5, reducing overlap and improving clarity.

3) **Comment 3:** Decimal places are not needed in Table S3 for the DIC and WAIC.

RESPONSE: Thank you. We have removed unnecessary decimal precision and rounded DIC and WAIC to integers for easier comparison in Supplementary Table S3.

4) **Comment 4:** Decimal places could be dropped in Tables S6 and S7. I think the number of countries and estimates makes the numbers hard to take in. Using integers would allow patterns to be discerned for a slight cost in accuracy. However, perhaps the point of these tables is for countries to use the data, in which case a higher accuracy is worthwhile.

RESPONSE: Thank you for this suggestion. We carefully considered the balance between readability and precision in Supplementary Tables S6 and S7. We agree with you that these tables could be used by national policymakers and technical experts who may rely on the exact values for planning and comparison. Thus, we have opted to retain the decimal places to preserve accuracy.

5) **Comment 5:** The authors could consider providing the maps and tables online in an interactive display. That could be useful for policy makers and staff in the individual countries.

RESPONSE: Thank you for this excellent suggestion. We agree that an interactive online dashboard would greatly enhance the accessibility and impact of our findings. We will consider developing and launching such a tool from this study in our future dissemination efforts.

6) **Comment 6:** I would encourage the authors to share their R code, for example on GitHub. This increases transparency and allows others to build on their work.

RESPONSE: Thank you for this valuable suggestion. We fully support open science and will share the R code for our modelling on GitHub following publication. We also plan to develop a separate methodological paper that will provide further technical details and code. This has been clarified in the Data Availability section.

- **Revised Manuscript – Data Sharing Statement:** "All R code used for data analysis and modelling will be made publicly available on GitHub following publication."

REVIEWER #2 COMMENTS:

Comments to the Author: Overall, this is a well written and interesting paper, that makes important contribution to the literature. While there are previous studies discussing progress in vaccine coverage and inequalities, this paper discusses the feasibility of achieving basic immunization targets in Africa by 2030, which is informative and can also serve policymakers. I have some concerns and comments, which are outlined below.

RESPONSES: We sincerely thank the reviewer for these encouraging comments and constructive suggestions. We have addressed all points below and revised the manuscript accordingly.

1) **Comment 1:** Abstract: "Childhood immunization coverage improved significantly across most of the African countries in our sample from 2000 to 2030, yet 13 countries were projected to fall short of full immunization at the national level." - This sentence can be split into two - one describing the past trend and one the projected change to improve clarity. "Despite being high-SDI nations…" - It is unclear in the abstract what SDI stands for. Define the term.

RESPONSES: Thank you for this suggestion. We revised the abstract to clearly separate past trends and future projections and added the definition of SDI (Socio-Demographic Index) at first mention.

- **Revised Manuscript – Abstract:** "Childhood immunization coverage improved significantly across most African countries from 2000 to 2019. However, projections suggest that 12 countries are unlikely to achieve global targets for full immunization by 2030 at the national level if current trends continue. Notably, high–Socio-Demographic Index (SDI) countries such as South Africa, Egypt, and Congo Brazzaville are projected to miss immunization targets across all sub-national regions."

2) **Comment 2:** Main text: When the authors talk about the results of future projections they use past tense, for example, "Socioeconomic inequalities were widespread in 2000 but were projected to decline or level off in 32 countries by 2030…". When referring to projected data, I would suggest avoiding past language, which sounds "factual", whereas these figures are based on assumptions about future trends. Generally, the authors could discuss the projected results with more caution and avoid "factual" language for example "X% of children are estimated to be vaccinated if the current trends continue…" or "countries are projected to reach X% vaccine coverage if they continue with the current rate of vaccination" or something like that.

RESPONSES: Thank you for this valuable comment. We fully agree and have carefully revised the manuscript, specifically the Results, Discussion, and Conclusion sections, to consistently apply cautious, forward-looking language when describing projections, using terms such as "are projected to," "are expected to," and "if current trends continue," to ensure clarity and avoid presenting future estimates as factual certainties.

3) **Comment 3:** The authors mention a target by 2030 of at least 80% coverage for essential vaccines, however, my understanding is that the target is 90%. See for example: https://www.who.int/teams/immunization-vaccines-and-biologicals/strategies/ia2030/explaining-the-immunization-agenda-2030?utm_source=chatgpt.com
The authors should correct the interpretation of the data accordingly, with potentially more countries set to miss the target.

RESPONSES: Thank you for this important clarification. We fully acknowledge that the Immunization Agenda 2030 (IA2030) establishes a global target of at least 90% national coverage for essential vaccines by 2030. In our primary analysis, we applied the 80% threshold to reflect the minimum benchmark commonly used in Universal Health Coverage (UHC) monitoring frameworks and previous global health literature. This target serves as a foundational goal for equitable access to essential services.

In response to your comment, we have now incorporated the 90% IA2030 target into our analysis. Specifically, we assessed the probability of achieving both the UHC (80%) and IA2030 (90%) thresholds across countries and regions. These new results are presented in the main text and supplementary materials, including updated figures and tables. This dual-target approach allows for a more comprehensive interpretation of progress and highlights the significantly greater effort required to reach the higher IA2030 benchmark.

We believe the inclusion of both targets strengthens the policy relevance and comparability of our findings, and we have clarified these updates throughout the revised manuscript.

4) **Comment 4:** In Figure 1, the countries could be ordered from best to worst performing, it would be easier to immediately identify those countries that are on course to achieve the target and those least likely to achieve it. The SDI ranking does not seem to add much to the figure. Also, in the caption of the figure, the authors should specify that the 2030 values are based on current trends or somehow indicate the implicit assumptions about the future course of action made in the 2030 figures.

RESPONSES: Thank you for this helpful suggestion. We appreciate the rationale for reordering countries based on projected performance to facilitate quick visual comparisons. However, we chose to retain the SDI-based ordering for two key reasons, unless the Editor suggests otherwise. First, it ensures consistency across all figures and tables, allowing for easier navigation and cross-referencing. Second, it enables readers to interpret immunization coverage within the broader context of national socio-demographic development, helping to

highlight countries that are over- or underperforming relative to their SDI. This framing aligns with our discussion, where we explicitly compare country performance in relation to SDI across two dedicated paragraphs. We believe keeping the SDI order in the results supports and strengthens this contextual analysis.

To improve usability, we have revised all Supplementary Tables to list countries alphabetically, allowing readers to more easily locate individual countries of interest. We thank the reviewer again for this constructive input, which has improved both the clarity and accessibility of our presentation.

5) **Comment 5:** Also in Figure 1, the authors show the AARC values from 2000 to 2030. I am not sure what the benefit of this AARC number is, since it includes both observed (and modeled) and projected data (including multiple assumptions about the future). Instead, the authors could present AARC values based on the long-term trend (2000 to 2020), and also based on a shorter-term trend (which is now included in the supplement) to show whether progress is accelerating or slowing down, this would add some interesting information (are we still on course or are we falling behind lately).

   And then it could be interesting to calculate the minimum AARC needed to reach the 2030 target to demonstrate how much faster countries would need to increase vaccination efforts to catch up.

RESPONSES: Thank you for this valuable suggestion. We agree that disaggregating the AARC into observed (2000–2020) and projected (2020–2030) periods provides useful insights into recent trends and the momentum needed to achieve the 2030 targets. We have now conducted the analyses for the observed and projected AARC values separately. We believe this combination of overall (2000–2030), observed (2000–2020), projected (2020–2030), and target-oriented added AARC values offers a more complete understanding of progress and gaps. The new analyses are presented in **Supplementary Table S9**. Also, AARC values based on the long-term (2000–2020) and short-term (2010–2020) trends has been presented in **Supplementary Figure S30**.

Note that as you suggested in Comment 3, we also estimated the additional AARC required between 2020 and 2030 to reach both the UHC (80%) and IA2030 (90%) coverage targets. These added AARC values, also shown in **Supplementary Table S9**, provide a practical indication of the level of acceleration needed in the final decade.

We would like to retain the overall AARC from 2000 to 2030 for Figure 1. This is because the 30-year AARC combines past and expected trends, reflecting the cumulative effort needed to meet the 2030 goals. It also supports comparative analyses across countries with different baseline years or levels of immunization performance.

6) **Comment 6:** The authors could also try a few different assumptions for the future, for example vaccination levels in 2030 if AARCs were same, if they were based on the best performing region in each country, or something like that. The authors could play around

with these assumptions a bit more to demonstrate different future scenarios depending on the course taken. Just as an idea.

RESPONSES: Thank you for this thoughtful suggestion. We agree that exploring alternative projection scenarios can offer valuable insights. While developing full scenario-based models was beyond the scope of this study, we have now included a simple comparison of projected 2030 coverage using the observed AARC of the best-performing region within each country, as a sensitivity analysis. This additional analysis, presented in a new Supplementary Figure S31, illustrates the potential impact of accelerated progress based on existing within-country performance. We hope this provides a useful perspective on possible future trajectories.

- **Revised Manuscript – Methods – Sensitivity Analysis**: "In addition, given the variation in progress across sub-national regions within countries, we compared the national 2030 projections with an alternative scenario based on the observed AARC of its best-performing region, defined as the region with the highest AARC during the 2000–2019 period.

- **Revised Manuscript – Results**: "Supplementary Figure S31 shows the national projections based on the observed AARC of best-performing region in each country. This alternative scenario illustrates the potential gains that could be achieved if all regions followed the trajectory of their top-performing one, providing valuable insight for policymakers seeking to accelerate national progress through targeted regional strategies."

7) **Comment 7:** How do the authors deal with countries when they only have 1 survey for that country? This is the case with Angola, Comoros, Cote d'Ivoire, Eswatini, and a few other countries. How are values for the period 2000-2020 and AARCs calculated in that case? The authors should be more explicit about their assumptions and modeling. When calculating the SII and RII for 2030, did the authors use rates of change specific to each wealth/education group? Or did they apply the country-level AARCs?

RESPONSES: Thank you for raising these important points. For countries with only one available survey (e.g., Angola, Comoros, Côte d'Ivoire, Eswatini), we estimated temporal trends using the global time trend combined with spatial borrowing from neighboring countries and sub-national regions through the Bayesian hierarchical framework. This approach allowed us to generate informed estimates in data-sparse contexts, while appropriately incorporating higher uncertainty. We have clarified these assumptions in the revised Methods section.

Regarding the calculation of SII and RII for 2030, we used subgroup-specific rates of change rather than applying national AARCs uniformly. Specifically, we modelled interactions between subgroup (wealth or education level) and time, and included sub-national region-level random slopes to allow subgroup-specific trajectories to vary across sub-national areas. This structure enabled us to project stratified coverage trends to 2030 and derive corresponding inequality indices. We have now detailed this modelling approach in both the revised Methods and Supplementary Modelling sections.

- **Revised Manuscript – Methods – Statistical Analysis**: "To estimate sub-national trends in immunization coverage, we applied Bayesian spatio-temporal models incorporating spatial dependencies and temporal smoothing. In countries with only one available survey, temporal trends were inferred using the global time trend and spatial borrowing from neighboring countries and sub-national regions, with increased uncertainty reflected in the posterior estimates."

- **Revised Manuscript – Methods – Statistical Analysis:** "We conducted stratified analyses by wealth quintile and education level, incorporating random slopes by sub-national region and interaction terms with time. This allowed us to estimate subgroup-specific trajectories and project immunization coverage, AARC, SII, and RII values to 2030, assuming observed trends from 2000–2020 remain constant in the absence of major interventions. Full model details are available in the Supplementary Modelling section."

8) **Comment 8:** "By 2030, wealth-related inequalities were projected to decline in many subnational regions." Again, this result is driven by the assumptions made about the future trends. What were these assumptions, what were the rates assumed for each wealth/education group?

RESPONSES: Thank you for this important observation. Our 2030 projections are based on subgroup-specific temporal trends estimated using Bayesian spatio-temporal models. These models incorporate interaction terms between wealth and education groups and time, along with region-level random slopes, allowing us to estimate distinct trajectories for each subgroup across sub-national regions. We assume that trends observed from 2000 to 2020 continue unchanged through 2030 in the absence of major policy shifts or structural changes. These assumptions are now clarified in the revised Methods section, with full model specifications provided in the Supplementary Modelling (Supplementary Materials – Page 6).

9) **Comment 9:** Concerning the projections, again it may be interesting to add what AARCs are needed for each country/region to reach the 2030 goals and also to close the socioeconomic gaps.

RESPONSES: Thank you for this helpful suggestion. We agree that estimating the additional AARCs needed to reach the 2030 targets offers valuable policy insights. As noted in our response to Comment 5, we have included national-level estimates of the additional AARCs required to achieve both the UHC (80%) and IA2030 (90%) benchmarks in **Supplementary Table S9**. In addition, we have extended this analysis to socioeconomic subgroups, specifically by wealth quintile and education level, and present these results in **Supplementary Tables S10 and S11.**

10) **Comment 10:** In Figure 5, it may be better to use the data for 2000 and 2020, instead of

2000 and 2030, to present factual (modeled?) progress. More discussion is needed about the difference in the interpretation of SII and RII, what do each of the groups mean? What does it mean about where progress needs to be made? What is the benefit of this figure and the presented information?

RESPONSES: Thank you for this insightful comment. We agree that distinguishing between observed (2000–2020) and projected (2020–2030) trends can enhance interpretability. In response, we have added new supplementary figures (Supplementary Figures S23–S24) that present these periods separately. We retained the 2000–2030 results in the main Figure 5 to provide a full trajectory of inequality changes and to maintain consistency with other results across the manuscript.

We have now revised the Results section to improve the interpretation of the Slope Index of Inequality (SII, absolute inequality) and Relative Index of Inequality (RII, relative inequality). Specifically, we clarify how the combination of SII and RII reveals whether improvements are shared equitably or are disproportionately benefiting specific groups. The four quadrant classifications in Figure 5 now guide the reader in identifying areas of progress, persistent gaps, or worsening inequality.

- **Revised Manuscript – Results:** "Figure 5 presents a two-dimensional graph showing changes in absolute (SII) and relative (RII) socioeconomic inequalities from 2000 to 2030 across all immunization indicators in 38 African countries, exhibiting broadly similar patterns for wealth- and education-related inequalities. Countries and indicators are grouped into four categories: (1) progress in both SII and RII, indicating reductions in absolute and relative gaps; (2) progress in absolute terms but persistent relative inequality; (3) progress in relative terms but persistent absolute inequality; and (4) worsening inequalities on both scales. While increases or decreases in SII or RII can be assessed individually, interpreting their combined behavior offers deeper insights into where progress or setbacks are concentrated. For example, reductions in both SII and RII signal progress toward equity; stable SII with declining RII suggests that both disadvantaged and advantaged groups are improving, but at different rates; while rising RII typically points to stagnation or decline among disadvantaged groups. The majority of countries and indicators fell into Group 1, reflecting projected progress toward greater equality. Group 2 patterns suggest slower gains among disadvantaged groups, observed only for Nigeria's full immunization. Group 3 indicating overall declines, observed only for Guinea's MCV1 immunization, consistent with patterns observed in Figure 1 and Supplementary Figure S16. Only two countries fell into Group 4, where both SII and RII worsened, including BCG in Benin and Guinea. These groupings help policymakers pinpoint where targeted interventions are most urgently needed to close persistent or widening gaps. Complementary analyses comparing 2000–2020 (observed) and 2020–2030 (projected) trends, presented in Supplementary Figures S23–S24, provide additional insights into pass progress and future challenges. While six country-indicator combinations showed increasing inequality over 2000–2020, only two are projected to experience rising inequality in 2020–2030, suggesting a potential shift toward more equitable progress if current trajectories are maintained. Supplementary Figures S25–S29 further detail regions experiencing rising inequalities across all five immunization indicators."

REVIEWER #3 COMMENTS:

Comments to the Author: In this manuscript, the authors use spatiotemporal modeling approaches (BYM) to estimate routine childhood immunization coverage for five indicators stratified by space, time, and socioeconomic status, while also projecting trends through 2030 and conducting inequality analyses. In general, the manuscript is very well written; the analysis benefits from a large data set as well as a thorough consideration of both coverage patterns and inequality across multiple dimensions. My comments below mainly focusing on the technical aspects of the manuscript and methods, as the results and discussion are quite thorough.

RESPONSES: We sincerely thank you for the thoughtful and constructive comments. Below, we provide detailed point-by-point responses and have revised the manuscript accordingly to improve clarity, transparency, and methodological rigor.

1) **Major comment 1:** Introduction: I would suggest that the authors more fully review the literature and cite some of the previous multi-country modelling studies of subnational immunization coverage that have been conducted at regional and continental levels. This study does provide substantial novelty beyond previous efforts to estimate subnational coverage, including via stratification by socioeconomic status and producing projections, but a clearer description of prior work in this area and comparison / contrast to the present work would strengthen the introduction.

RESPONSES: Thank you for this suggestion. We have now expanded the Introduction to more fully review and cite prior multi-country modelling studies of sub-national immunization coverage at regional and continental levels. Specifically, we have highlighted how previous studies (e.g., Mosser et al., Utazi et al., Alegana et. al) focused on estimating fine-scale spatial patterns but did not stratify by socioeconomic status or provide forward projections. We now explicitly position our study relative to this prior literature, clarifying the additional contributions it makes.

- **Revised Manuscript – Introduction:** "Notably, prior multi-country modelling efforts, such as Mosser et al. (2019) and Utazi et al. (2018), have mapped fine-scale immunization coverage across Africa, providing valuable spatial insights, but they did not incorporate socioeconomic stratifications, forward projections, or detailed inequality analyses at sub-national levels [17, 18]. A multi-national study with sub-national level analysis is needed to overcome these limitations by integrating both national and sub-national perspectives, enhancing statistical power, and enabling robust trend estimations and inequality assessments across sub-national regions and socioeconomic groups."

2) **Major comment 2:** Methods: The authors use DHS surveys; did they also consider using other surveys that report subnational immunization coverage data (MICS etc.?) Some discussion of this would be helpful, even if in the limitations section.

RESPONSES: Thank you for this helpful comment. We chose to focus on DHS data because it offers the most standardized, comparable, and consistently available multi-country immunization data across Africa, which is critical for harmonized modelling across countries and time periods. While we acknowledge that other sources such as MICS provide valuable sub-national data, integrating them to the DHS dataset poses methodological challenges due to differences in sampling design, indicator definitions, and survey timing. To ensure consistency and reduce complexity, we therefore limited our analysis to DHS data, and we have now clarified this limitation in the revised manuscript.

- **Revised Manuscript – Discussions – Limitations:** "Although we focused on DHS due to its comparability and wide coverage, other sources, such as Multiple Indicator Cluster Surveys (MICS), also offer valuable sub-national data. Yet, methodological differences in sampling and indicator definitions complicate integration, and future work could explore harmonizing these data sources [70]."

3) **Major comment 3:** Methods: do the authors implement any constraints on coverage, particularly for full immunization coverage (ie, FIC has to be less than each of BCG/Polio3/DPT3/MCV1 coverage?). If not, it would be useful to know whether there are locations where the model predicts higher FIC coverage than coverage of one of the other vaccines; ie, to what extent are these estimates internally consistent?

RESPONSES: Thank you for this important comment. We did not apply mathematical constraints to force full immunization coverage (FIC) to remain below or equal to the coverage of its individual components (BCG, Polio3, DPT3, and MCV1). This modelling choice was made to avoid introducing artificial trends and to allow flexibility in reflecting real-world variation in survey reporting and program delivery. While we recognize that FIC should not exceed its components, we conducted a post-modelling consistency check and found that such instances were rare (<1%) and minor in magnitude (typically ≤3 percentage points). These cases were most common in data-sparse settings and had negligible influence on overall conclusions. We have clarified this modelling decision in the Methods section and included the consistency results in Supplementary Table S5.

- **Revised Manuscript – Methods – Validation Analysis:** "We modelled full immunization coverage and its component vaccines (BCG, Polio3, DPT3, and MCV1) independently, without imposing constraints that force full coverage to remain below its components. A post-modelling consistency check revealed only a few minor instances (0.8%) where full coverage slightly exceeded one or more components, with negligible effect on the overall findings (Supplementary Table S5)."

- **Supplementary Table S5 – Table Note:** "Notes: This table evaluates internal consistency by comparing modelled full immunization coverage (FIC) estimates with each component vaccine (BCG, MCV1, Polio3, and DPT3). Instances where FIC exceeded any component were identified. Across all countries, regions, and time points, fewer than 1% of estimates showed minor exceedances, typically ≤3 percentage points. These discrepancies likely reflect survey sampling variability, reporting errors, or data

inconsistencies. Given their rarity and small magnitude, they were considered to have minimal impact on study conclusions."

4) **Major comment 4:** Methods: Why were studies only included if conducted between 2000-2019 (that is, why were more recent studies excluded?) Particularly given the impact of the COVID-19 pandemic on childhood immunization coverage, it would seem like it would be particularly important to include what data is available from the last 5 years if at all possible, as I might expect that this would affect current estimates and particularly future projections. I appreciate that this is given as a limitation in the discussion, but would be interested to understand why the data weren't included instead - as this does seem a rather substantial limitation.

RESPONSES: Thank you for this important comment. We agree that including data post-2019 would enhance the analysis, particularly in capturing the effects of the COVID-19 pandemic on immunization trends. However, at the time of analysis, the harmonized DHS datasets available via IPUMS only extended through 2019. More recent DHS rounds were either unreleased, incomplete, or not yet integrated, making systematic inclusion across countries impractical. To maintain comparability and consistency in modelling, we therefore focused on the 2000–2019 period. This limitation had now been more clearly stated in both the Methods and Discussion sections and identified as an important area for future research.

- **Revised Manuscript – Methods – Data Sources:** "We included all available DHS data for African countries from 2000 to 2019, ultimately including 1,000,435 records of living children from 104 datasets across 38 African countries. While more recent DHS rounds exist, they were neither publicly released nor integrated into the IPUMS system at the time of analysis, making systematic inclusion impractical. Furthermore, direct access to DHS datasets has been constrained following the program's suspension under the recent U.S. administration. To ensure consistency and comparability across countries, we therefore focused our analysis on the 2000–2019 period. Details of included surveys by country are provided in Supplementary Table S1."

- **Revised Manuscript – Discussions – Limitations:** "Second, the analysis was limited to DHS surveys conducted between 2000 and 2019, based on the availability of harmonized data through IPUMS. While more recent surveys may reflect the impact of COVID-19, they were not yet publicly available or consistently integrated, and their absence remains a limitation."

5) **Major comment 5:** Supplemental methods / spatio-temporal models: If I understand the model correctly, the authors model a global temporal linear trend (beta) in addition to individual areal-unit-level effects. One might expect that the overall trends in vaccine coverage are likely to vary within countries substantially - did the authors consider a model wherein country-level aggregate time trends were estimated rather than a single global time trend across all countries, ie. Beta_c where c varies by country? Or are there technical reasons why such a model would not be possible to fit? The same holds true for the BYM2 model, for instance, described on page 7, which also uses a single global time

trend, and I think also to essentially all of the models 1-5 which also include beta_t.

RESPONSES: Thank you for this insightful question. While our models include a global temporal linear trend (β), we fully acknowledge that vaccine coverage trends may differ across countries and regions. To address this, we incorporated hierarchical spatial random effects, both structured and unstructured, at the national and regional levels, along with region- and country-specific random slopes that interact with time. This flexible structure allows for variation in temporal trends across locations, rather than relying solely on a global trend, while maintaining model stability in data-sparse settings. We have clarified this modelling approach more explicitly in the Methods and Supplementary Methods sections.

- **Revised Manuscript – Methods – Statistical Analysis:** "We conducted stratified analyses by wealth quintile and education level, incorporating random slopes by sub-national region and interaction terms with time. This allowed us to estimate subgroup-specific trajectories and project immunization coverage, AARC, SII, and RII values to 2030, assuming observed trends from 2000–2020 remain constant in the absence of major interventions. Full model details are available in the Supplementary Modelling section."

6) Major comment 6: Methods / Supplemental methods: Related to the above, the authors note that the various model formulations are essentially assuming a linear time trend for each area (or, perhaps more precisely, that logit(coverage) is linearly related to time). If I follow the model specifications correctly, in locations with a single survey the temporal trends are essentially driven by the global time trend (beta_t). This is a relatively major assumption and can lead to some dramatic time trends.

a. Did the authors consider alternative methods of attempting to capture trends in time (e.g. using autoregressive or random walk models for the temporal component rather than a linear model, and/or including interactions between the spatial and temporal terms? Or alternatively using covariates to help in form the temporal trends (though appreciate that this may complicate forecasting)?

b. For instance, looking at the model_bym_2 results (Figure S8), BCG coverage increases dramatically in Angola from 2000-2020, by more than 25 percentage points in many locations. If we look at the WUENIC estimates for Angola, however, which include multiple survey time points and incorporate administrative data trends, BCG coverage is essentially estimated to be flat over this time period - with some increases and decreases, but much different than the dramatic continuous increase suggested by this model. I give Angola as one example, but in many cases the time trends implied here are quite different than what are implied by WUENIC. I bring this up mainly because the effects on projection are likely to be substantial - looking at the WUENIC estimates, one wouldn't necessarily expect coverage to increase dramatically by 2030. I appreciate that these are very different estimation methodologies, but it would be helpful to understand how these estimates (aggregated to the national level) compare to the widely-used WUENIC estimates.

RESPONSES: Thank you for this thoughtful and detailed comment. We agree that assuming a linear time trend on the logit scale is a modeling choice, especially in data-sparse contexts. We explored alternative temporal structures, including autoregressive and random walk models; however, these produced unrealistic and discontinued patterns that were not suitable

for generating smooth or interpretable projections. We also considered including covariates such as SDI or HDI, but this would have required projecting those covariates to 2030, which would introduce further uncertainty.

We ultimately selected the logit-linear approach to balance simplicity, interpretability, and computational feasibility across 38 countries and five immunization indicators. This approach has been used in previous global health forecasting studies (e.g., Rahman S. et al. 2018; Rahman M. et al. 2023) and has the advantage of capturing rapid gains at low coverage levels and natural saturation at higher levels. In Angola, for instance, the estimated ~25 percentage point increases occurred in regions starting below 50% coverage, where gains are typically easier. As coverage approaches 80–90%, the model naturally attenuates growth, reflecting diminishing returns.

We have now clarified this modeling rationale and its limitations in the revised manuscript, noting the potential for divergence from administrative estimates (e.g., WUENIC) and the need for more flexible temporal modeling as more recent data become available.

- **Revised Manuscript – Methods – Statistical Analysis:** "To model time trends, we applied a linear specification on the logit scale for each sub-national region, enabling projections to 2030 [29]. Alternative approaches, including autoregressive and random walk models, were tested but resulted in unstable or erratic trends, limiting interpretability. We also explored the use of country-level covariates (e.g., SDI, HDI), but this approach would require projecting those covariates to 2030, introducing further uncertainty. The final logit-linear model offered a balance of simplicity, interpretability, and performance across all indicators and settings, consistent with prior forecasting studies [11, 30]."

- **Revised Manuscript – Discussion – Limitations:** "While the logit-linear time trend structure allows for interpretable and consistent projections, it may over-smooth local variation or diverge from administrative estimates such as WUENIC in settings with sparse data [5]. This is particularly relevant in countries with only one survey, where projections are more reliant on global trends. Nevertheless, this method reflects common epidemiological patterns, for instance, faster initial gains and slowing progress at higher coverage levels, and has been validated in previous global health modelling work [11, 30]."

7) **Major comment 7:** Methods: the authors write that they "conducted stratified analyses by subgroups and incorporated random slopes to account for variations in these variables across locations and included interaction terms between these variables and survey year". The formulas in the supplementary materials, however, seem to be only for the more general spatio-temporal models, and I don't see anything in the descriptions or equations / notations that specifies how this was done. Some additional detail describing this process and perhaps showing these results would be helpful as the stratified analysis inequality is one of the more novel parts of this manuscript, and without additional description of how this was implemented it's hard for the reader to fully interpret the results. For instance, did the authors run a true stratified analysis with separate models for each of the subgroups? Or did they run a single model that incorporated both random slopes by location (were these hierarchical in any way?) and interactions by survey year?

If this is the case, then these seem like they could be highly flexible models - including random slopes on the covariates that vary by subnational location and over time, while also allowing for the spatial effects in the BYM model. I would think that the model might face challenges in accurately estimating all of these location-specific effects, particularly for subnational regions that only have a single survey-year of data if so, but perhaps that wasn't the authors' experience. In any case, a more detailed description of the methods used to accomplish this would be very helpful in the supplemental materials and allow for a more thorough understanding of the results presented here.

RESPONSES: Thank you for this valuable comment. We agree that the subgroup modelling approach warranted clearer explanation given its importance to the inequality analysis. To clarify, we used a unified Bayesian spatio-temporal model incorporating both fixed effects, random effects, and interaction terms to estimate subgroup-specific trends. Rather than fitting separate models for each subgroup (wealth or education group), we included fixed effects for subgroup categories, interaction terms with time, and region-level random slopes to capture temporal variation across subgroups and subnational regions. This allowed us to model subgroup-specific trajectories while maintaining consistency and statistical efficiency.

We have now expanded the **Supplementary Modelling – Model Description (Pages 8–11)** with further detail on the model structure, notation, and example formula. We have also added subgroup-specific outputs to the **Supplementary Tables S7–8 and S10–11**. We appreciate the reviewer's suggestions, which have helped strengthen the clarity and transparency of our methods.

8) **Major comment 8:** Methods: The authors validate the model using average absolute difference.

A few questions about model validation:

a. It might be helpful to also give some additional measures, like the mean error (to assess whether the model is systematically biased either high or low, which metrics like the average absolute difference can't tell us) and the 95% coverage of the uncertainty or predictive intervals (to assess how well uncertainty is calibrated in the model)

b. I would encourage the authors to also provide some out-of-sample validation statistics for their models, as all of the validation metrics here appear to be in-sample if I am reading them correctly. This could be accomplished by holding out surveys, for instance, in a cross-validation framework. As the authors are also projecting into the future, it would be helpful to see how accurately the model is able to make forward projections, so some validation by withholding recent years of data and seeing how well the model can predict based on past trends would be very useful.

RESPONSES: Thank you for this excellent suggestion. We agree that a more comprehensive validation framework strengthens the reliability of our modelling results. In response, we expanded our model validation to include the following components:

1. **Mean error (ME)** was added alongside average Absolute Difference (AD) to assess potential systematic bias in predictions.

2. **Out-of-sample validation** was conducted using a cross-validation framework by holding out all survey years from 2017–2019. This allowed us to evaluate the model's predictive performance based on historical data.
3. **Sensitivity analysis** was performed to assess the impact of model choice (Besag, BYM, BYM2) on 2030 projections.

We also have now reported the 95% credible interval coverage to evaluate uncertainty calibration at the national level and across wealth and education subgroups.

These results are summarized in **Supplementary Tables S3–S5** (model comparison and validation metrics) and **Supplementary Tables S6–S8** (coverage and subgroup-level uncertainty). Additional visualizations are provided in **Supplementary Figures S1–S14**.

We have extensively revised the modelling framework and validation strategy, finalized BYM2 as the best-performing model, and updated the manuscript accordingly. We sincerely appreciate the reviewer's thoughtful recommendation, which has significantly improved the rigor and transparency of our analysis.

- **Revised Manuscript – Methods – Statistical Analysis:** "Model comparison and selection were conducted using data from 2000 to 2016, with 2017–2019 held out for out-of-sample validation. We assessed model performance using Deviance Information Criterion (DIC), Watanabe-Akaike Information Criterion (WAIC), Absolute Difference, Mean Error, and computational efficiency (runtime in seconds), evaluating each model on in-sample (2000–2016), out-of-sample (2017–2019), and full-period (2000–2019) performance. Among the six model variations, we selected model_bym2_2, a hierarchical BYM2 model that captures within- and between-region spatial dependencies and accounts for national-level structure. It demonstrated the best overall performance across all immunization indicators based on DIC, WAIC, average absolute difference, and mean error. Its nested spatial structure also enhanced interpretability, aligning well with the hierarchical country–region relationships in the data. Details of model comparison and validation are presented in Supplementary Table S3 and Figures S1–S14."

9) **Major comment 9:** Results: The authors write that "The sensitivity analysis using recent trends (2010-2020), presented in Supplementary Figure S27, shows that these trends generally project lower Full immunization coverage for 2030 and slower progress with smaller AARC values. In addition, recent trends appear less effective in capturing regional disparities and variations, likely due to data limitations. In contrast, overall trends (2000-2020) provide more robust projections, with higher coverage levels and better representation of regional diversity". An alternative explanation may be that coverage gains have slowed or plateaued in many locations in more recent years, which has been a phenomenon observed at global, regional, and select national levels by WUENIC among other sources. The maps in S23 do appear to demonstrate, as might be expected, that data sparsity in the model fit using only 2010-2020 shows less regional variation simply due to the exclusion of many previous surveys. I would be cautious, however, with the assertion that overall trends using 2000-2020 provide more robust estimates simply because they are higher in coverage, as one could argue that the inclusion of the earlier years may instead be overestimating the expected gains between 2020-2030, particularly given the relative simplicity of the projection

method used here (a continuation of the logit-linear relationship between coverage and time). The authors explain this well in the subsequent discussion, but I think that the framing in the results section might benefit from some of the same nuance.

RESPONSES: Thank you for this important comment. We agree that greater nuance is needed in interpreting the sensitivity analysis. While the overall trend (2000–2020) provided broader data coverage and higher projected estimates, we acknowledge that including earlier years may overestimate future progress, especially in light of recent stagnation documented by WUENIC and other sources. We have revised both the Results and Discussion sections to clarify that projections based on long-term trends should be interpreted with caution, particularly where more recent data indicate plateauing. We have updated the text accordingly.

- **Revised Manuscript – Results:** "The sensitivity analysis using recent trends (2010–2019), presented in Supplementary Figure S30, projects lower full immunization coverage by 2030 and slower progress, as indicated by smaller AARC values."
- **Revised Manuscript – Discussion:** "We conducted another sensitivity analysis using recent trends (2010–2019), which projected lower full immunization coverage by 2030 and slower progress, as reflected by smaller AARC values. This suggests a deceleration in coverage improvements during the recent period, consistent with global reports, including WUENIC estimates, that document plateauing immunization rates in many countries [5]. Notably, this stagnation was observed even before the COVID-19 pandemic, which further disrupted routine childhood immunization programs across Africa due to lockdowns, healthcare system strain, and interruptions in vaccine delivery [64]. These findings highlight the urgent need for additional efforts and targeted strategies to sustain and accelerate progress, as emphasized by the Immunization Agenda 2030 [7]. At the same time, projections based on overall trends (2000–2019) yield higher coverage projections and better representation of regional variation. However, this approach may overestimate future progress by smoothing over recent stagnation, and thus findings should be interpreted with caution."

10) **Major comment 10:** General: While the manuscript focuses on Africa, the authors' affiliations appear to be all from institutions outside of Africa. I would suggest that the authors seek to include the perspectives of colleagues and experts from the countries included in this study to inform and contextualize these findings if not already done, while providing appropriate acknowledgements and/or authorship to these individuals depending on their contributions.

RESPONSE: Thank you for this thoughtful comment. We fully agree that local perspectives are essential for ensuring contextual relevance and policy impact. One co-author is originally from Africa, and others bring extensive experiences conducting scientific research and/or implementing health projects across African countries through long-standing collaborations with African governments, research institutions and donors. Their insights informed both the analysis and interpretation. We remain committed to strengthening these collaborations and expanding engagement with in-country experts in future work.

Additionally, we presented the preliminary results of this study at the 2024 World Congress of Epidemiology (WCE) in South Africa. During this meeting, we received valuable feedback from African public health experts, which informed both the interpretation of our results and their policy relevance at national and sub-national levels. These contributions have been acknowledged in the revised manuscript. We are committed to deepening partnerships with African researchers and fully support inclusive, locally engaged research approaches.

- **Revised Manuscript – Acknowledgements:** "We would like to express our sincere appreciation to colleagues and public health experts from several African countries, including those who provided valuable feedback during the 2024 World Congress of Epidemiology in South Africa, which helped strengthen the contextual interpretation of this study."

11) **Minor comment 1:** Please add line numbers to subsequent revisions of the manuscript to facilitate reviewer comments if possible

RESPONSES: Thank you for this suggestion. We have now added line numbers to the revised manuscript to facilitate reviewer comments and improve clarity during the review process.

12) **Minor comment 2:** Methods and throughout: I would suggest specifying in the methods that measles here is presumably MCV1, and not the second dose of measles (since Polio3 and DPT3 have the dose number indicated in the indicator definition).

RESPONSES: Thank you for this suggestion. We confirm that the measles indicator used in this study refers to the first dose of the measles-containing vaccine (MCV1). We have clarified this in the Methods section and revised the manuscript to use the term consistently throughout.

13) **Minor comment 3:** Methods: it would be helpful to have some additional information on how the data were geolocated and what shapefile was used for the analysis. From looking at the maps it appears that the unit of analysis was generally the first administrative level; were the data matched to shapefiles from DHS? A few lines explaining explicitly what the subnational unit of analysis was and what shapefile was used would be helpful. Were there any instances in which subnational boundaries changed over the study period; if so, would also explain how those were handled?

RESPONSES: Thank you for this insightful comment. We have clarified the geolocation procedures and spatial units used in our analysis. Specifically, we used first administrative-level units (Admin-1), as defined in the DHS GPS datasets, as our primary unit of analysis. Survey clusters were georeferenced using shapefiles from the DHS Spatial Data Repository corresponding to each survey year. To ensure consistency across time, we harmonized sub-national regions by aligning all surveys to the most recent administrative boundaries. In countries where administrative boundaries changed, smaller units were merged into broader,

stable regions to maintain comparability over time, following established practices in spatiotemporal epidemiology. These details have been added to the revised Methods section for transparency and reproducibility.

- **Revised Manuscript – Methods – Data sources:** “We analyzed data at the first administrative-level units (Admin-1), which served as the primary unit of analysis. These units were matched to national boundaries using shapefiles from the DHS Spatial Data Repository. For countries with multiple survey rounds, we harmonized sub-national boundaries to align with the most recent administrative definitions. In cases where boundaries had changed over time, smaller units were merged into broader regions to ensure consistency in regional comparisons, following established practices in spatiotemporal mapping of IPUMS DHS data [21].”

14) **Minor comment 4:** For all figures, I would consider providing at least one additional year that indicates the estimates at the end of the modeling period, to help distinguish somewhat between the estimates in years for which data was available and those which were purely projections. For instance, it would be useful in the maps in Figure 2 and Figure 4 to see not only the year 2000 and the year 2030, but also the last year in which the authors have available data (appears to be 2019)? Figure 1 shows this nicely by including 2000, 2020, and 2030, so that also could be an alternative approach. The 30 year gap between 2000 and 2030 hides some of the benefit of the analysis in producing a quasi-contemporaneous set of estimates for the present.

RESPONSES: Thank you for this helpful suggestion. We agree that including an intermediate time point improves interpretability, especially for distinguishing modeled estimates based on observed data from future projections. In response, we have revised Figures 2 and 4 to include 2020 estimates alongside those for 2000 and 2030. Figure 3 has also been updated with 2020 panels to provide a clearer picture of recent trends.

While Figure 5 continues to summarize overall changes from 2000 to 2030, we have added Supplementary Figures S23–S24 to separately illustrate changes in inequality during 2000–2020 and 2020–2030. Corresponding updates have also been made to Supplementary Tables S6–S8, which now include estimates for 2000 and 2020, as well as AARCs for both the historical (2000–2020) and projected (2020–2030) periods. Similarly, Tables S12–S13 on socioeconomic inequality now present estimates in 2000 and 2020, along with changes over the same intervals. All figure legends and table captions have been revised accordingly.

15) **Minor comment 5:** For figures S5-S9, is there a reason why these are given for BCG only? It would be helpful to see these for the other indicators as well for completeness. Since the authors are providing results through 2030, I would also suggest extending these plots to include the projections through that year.

RESPONSES: Thank you for this helpful comment. The initial focus on BCG in Figures S5–S9 was intended to illustrate model comparison and specification using a representative vaccine.

BCG was chosen due to its widespread use and easier validation against external data sources. However, we agree that extending this analysis to additional indicators would improve completeness and transparency. To avoid overwhelming the reader with excessive figures (e.g., 6 models × 5 indicators = 30 figures), we have opted to add time trend plots for all five immunization indicators (BCG, DPT3, Polio3, MCV1, and Full Immunization) using only the final selected model (BYM2_2). These new plots are now presented in Supplementary Figures S10–S14. Figures S1–S9 remain focused on BCG for detailed model comparison.

We chose not to extend these plots through 2030, as projections are less informative for model validation and may cause confusion. Instead, the revised plots now distinguish between in-sample (2000–2016) and out-of-sample (2017–2019) periods, with observed data and projected trends indicated accordingly. Additionally, Supplementary Figure S32 has been added to compare 2030 projections across selected models (Besag, BYM, and BYM2) as part of our model sensitivity analysis. All corresponding figure legends and manuscript references have been updated.

16) **Minor comment 6:** Last paragraph of results: "The sensitivity analysis using recent trends (2010-2020), presented in Supplementary Figure S27" - I think that this is S23? I would double-check the numbering of all figures to ensure that there wasn't an alteration that caused these to be mismatched.

RESPONSES: Thank you for pointing this out. We have carefully reviewed and corrected the figure numbering throughout the manuscript and supplementary materials to ensure consistency and accuracy. We appreciate your attention to detail.
