# Supplementary material for: Progress and inequality in child immunization in 38 African countries, 2000–2030: A spatio-temporal Bayesian analysis at national and sub-national levels
Source: PLoS Med. 2025 Jul 29;22(7):e1004664. doi: 10.1371/journal.pmed.1004664 (PMC12306735; doi:10.1371/journal.pmed.1004664)
Supplement: Attachment — Submitted filename: PlosM_Response to Reviewers_20250624.pdf [file pmed.1004664.s007.pdf]

Dear Editor and Reviewers,

Thank you very much for your positive feedback and the opportunity to revise our manuscript titled *"Progress and inequality in child immunization in 38 African countries, 2000–2030: A spatio-temporal Bayesian analysis at national and sub-national levels"* (PMEDICINE-D-25-01067R2).

We sincerely appreciate the constructive comments and thoughtful suggestions from the academic editor and reviewers, which have helped us further strengthen the manuscript. We have carefully addressed all remaining editorial and reviewer points.

A detailed, point-by-point response is provided below, and we have submitted the revised manuscript as requested. We hope the revisions meet your expectations and look forward to your decision.

Yours sincerely,
The Authors

EDITORIAL COMMENTS:

Primary Comments:

1) We agree that is makes sense to keep the SDI ordering in Figure 1.

RESPONSE: Thank you. We have retained the SDI-based ordering in Figure 1 as agreed

2) Could you modify figure 1 to make the numbers in the columns more readable, as commented on by R1? we are happy to keep the 3 columns, but would prefer the numbers to be easier to read.

RESPONSE: We have revised Figure 1 to improve the readability of the numeric columns, following Reviewer 1's feedback.

3) Please address the remaining two concerns from R3 with textual changes

RESPONSE: We have addressed the two remaining comments from Reviewer 3 with textual additions in the Methods (line 259) and Discussion sections (lines 581 & 585).

4) Please address the comment of R1 regarding the clarity in line 355.

RESPONSE: We have revised the sentence at line 355 for improved clarity, as suggested by Reviewer 1.

5) Please consider modifying figure 5 to make the datapoints easier to distinguish.

RESPONSE: We have updated Figure 5 with adjusted axis limits to enhance the visibility of data points.

6) Regarding the R code, Please provide us with a link to the Github repository. Ideally, we would like you to provide us with a DOI (e.g. generated by Zenodo).

RESPONSE: Thank you. We have created a GitHub repository, uploaded the R code, and included the link in the updated Data Availability Statement.

Other Comments:

7) Please confirm that your title complies with to PLOS Medicine's style. Your title must be nondeclarative and not a question. It should begin with main concept if possible. "Effect of" should be used only if causality can be inferred, i.e., for an RCT. Please place the study design ("A randomized controlled trial," "A retrospective study," "A modelling study," etc.) in the subtitle (ie, after a colon).

RESPONSE: Thank you for the guidance. We confirm that the title complies with PLOS Medicine's style.

8) Please confirm that your abstract complies with our requirements, including format (three sections: Background, Methods and Findings, and Conclusions) and providing all the information relevant to this study type https://journals.plos.org/plosmedicine/s/submission-guidelines#loc-abstract

RESPONSE: We confirm that the abstract has been revised to follow the required three-section format (Background, Methods and Findings, and Conclusions) and includes all necessary information.

9) In the last sentence of the Abstract Methods and Findings section, please describe the main limitation(s) of the study's methodology.

RESPONSE: Thank you. We have now added a sentence in the Methods and Findings section of the abstract to describe the main methodological limitation.

- **Revised Manuscript – Abstract – Line 70:** "The analysis was limited to DHS surveys 2000–2019, excluding more recent data during the COVID-19 period and potentially overestimating trends in data-sparse settings."

10) At this stage, we ask that you include a short, non-technical Author Summary of your research to make findings accessible to a wide audience that includes both scientists and non-scientists. The Author Summary should immediately follow the Abstract in your revised manuscript. This text is subject to editorial change and should be distinct from the scientific abstract. Ideally each sub-heading should contain 2-3 single sentence, concise bullet points containing the most salient points from your study. In the final bullet point of 'What Do These Findings Mean?' Please include the main limitations of the study in non-technical language.
Please see our author guidelines for more information: https://journals.plos.org/plosmedicine/s/revising-your-manuscript#loc-author-summary.

RESPONSE: We have added a non-technical Author Summary following the abstract, in line with the PLOS Medicine format and guidance. This includes bullet points and states the study's main limitation.

11) Please check that any use of statistical terms (such as trend or significant) are supported by the data, and if not please remove them.

RESPONSE: Thank you. We have reviewed the manuscript and ensured that all statistical terms are appropriately used and supported by data. Any unsupported terms have been removed or rephrased.

12) We expect all researchers with submissions to PLOS in which author-generated code underpins the findings in the manuscript to make all author-generated code available without restrictions upon publication of the work. In cases where code is central to the manuscript, we may require the code to be made available as a condition of publication. Authors are responsible for ensuring that the code is reusable and well documented. Please make any custom code available, either as part of your data deposition or as a supplementary file. Please add a sentence to your data availability statement regarding any code used in the study, e.g. "The code used in the analysis is available from Github [URL] and archived in Zenodo [DOI link]" Please review our guidelines at https://journals.plos.org/plosmedicine/s/materials-software-and-code-sharing and ensure that your code is shared in a way that follows best practice and facilitates reproducibility and reuse. Because Github depositions can be readily changed or deleted, we encourage you to make a permanent DOI'd copy (e.g. in Zenodo) and provide the URL.

RESPONSE: Thank you. We have uploaded the R code used in this study to the following GitHub repository: https://github.com/nguyenthephuong1/Child-immunization-africa. We have also updated the Data Availability Statement to reflect this.

13) Please remove the 'conclusions' subheading from the discussion. Please also remove any other subheadings from the discussion.

RESPONSE: We have removed the 'Conclusions' subheading and any other subheadings in the Discussion section, as requested.

14) Please consider avoiding the use of red and green in order to make your figure more accessible.

RESPONSE: Thank you for the suggestion. We recognize the importance of accessibility and have revised the color schemes accordingly. Specifically, we adjusted the colors in

Figures 1, 3 and 6 to be more colorblind-friendly, and all other figures already use colorblind-friendly palettes. We remain open to making any further adjustments if needed.

15) Please confirm that the appropriate usage rights apply to the use of the maps. Please see our guidelines for map images: https://journals.plos.org/plosmedicine/s/figures#loc-maps

RESPONSE: We confirm that all maps used in this study comply with the appropriate usage rights. Geographic boundary data were obtained from open-access sources (DHS Spatial Data Repository and GADM) for academic and non-commercial use under CC BY 4.0. This has been clearly stated in the figure captions.

16) PLOS has a 'Inclusivity in Global Research' policy which aims to promote collaboration and inclusivity in global health research. You are required to complete PLOS' questionnaire on inclusivity in global research and submit it with your revised paper. The policy and questionnaire can be found at https://journals.plos.org/plosone/s/best-practices-in-research-reporting.

RESPONSE: We have completed and uploaded the 'Inclusivity in Global Research' questionnaire as Supporting Information, following PLOS guidelines. We have also added the subsection 'Inclusivity in global research' to the Methods section.

17) As you state no ethical approval is needed, please amend your ethics statement.

RESPONSE: Thank you. We have revised the ethics statement to clarify that no ethical approval was required.

Revised Manuscript – Methods – Ethics statement: "This study is a secondary analysis of publicly available, de-identified data with no identifiable personal information. As such, no ethical approval was required."

18) Please ensure that the study is reported according to the RECORD guideline and include the completed RECORD checklist as Supporting Information. When completing the checklist, please use section and paragraph numbers, rather than page numbers. Please add the following statement, or similar, to the Methods: "This study is reported as per RECORD guideline (S1 Checklist).

RESPONSE: We confirm that the study has been reported in accordance with the RECORD guideline. The completed RECORD checklist (S1 Checklist) has been included as Supporting Information, with section and paragraph numbers provided. A statement has also been added to the Methods section.

19) Please modify the following sentence to ensure correct citation formatting, by removing the years: "“Notably, prior multi-country modelling efforts, such as Mosser et al. (2019) and Utazi et al. (2018), have mapped fine-scale immunization coverage across Africa, providing valuable spatial insights, but they did not incorporate socioeconomic stratifications, forward projections, or detailed inequality analyses at sub-national levels [17, 18].” You could consider "modelling efforts, e.g. [8, 9]", or "shown in the modelling work by Mosser and colleagues [17] and Utazi et al. [18]".

RESPONSE: Thank you for your guidance. We have revised the sentence accordingly.

- **Revised Manuscript – Introduction – Line 160:** “Notably, prior multi-country modelling efforts, such as those by Mosser et al. [17] and Utazi et al. [18], have mapped fine-scale immunization coverage across Africa, providing valuable spatial insights, but they did not incorporate socioeconomic stratifications, forward projections, or detailed inequality analyses at sub-national levels.”

REVIEWER #1 COMMENTS:

Comments to the Author: The methods and data appear sound. I can imagine the results being useful for policy in many African countries. The paper comes at an important time given the recent disruption to aid programs. I have only minor comments which are entirely optional for the authors to change or not.

RESPONSE: We sincerely thank the reviewer for the positive feedback and helpful suggestions. Below we respond to each of the comments.

1) **Comment 1:** Figure 1 now includes a third column of numbers, and the numbers are now a little squeezed in. For example, some of the negative signs for AARC are barely visible (e.g., Egypt, full immunisation) and some are potentially cut as there's no room. I prefer the previous version with two rows of numbers as I think this new version is too cluttered.

RESPONSE: Thank you for the helpful comment. We retained the three columns to present the full information but have now adjusted the layout of Figure 1 to improve spacing and readability.

2) **Comment 2:** The authors could consider hexagonal maps in future research as an alternative to standard choropleth maps (e.g., Figure 2), see the sugarbag package in R https://github.com/srkobakian/sugarbag. This approach will better show the geographically smaller countries.

RESPONSE: Thank you for the insightful suggestion. We agree that hexagonal maps could improve the visibility of smaller countries and will consider incorporating this approach in future research.

3) **Comment 3:** Line 335: "This pattern of disparity was consistent across all immunization indicators, although BCG and MCV1 had higher coverage than DPT3 and Polio3". Could be more specific about "higher" by giving an average percentage.

RESPONSE: Thank you for your suggestion. As this statement is based on visual interpretation of spatial patterns across the 38 countries, we do not report precise averages. However, we have revised the sentence to clarify this interpretation using approximate average differences from the data.

Revised Manuscript – Results – Line 369: "This pattern of disparity was consistent across all immunization indicators, although BCG generally showed higher coverage (approximately 10% higher) than MCV1, DPT3 and Polio3, as reflected by the greater presence of blue areas in the maps."

4) **Comment 4:** Some white space in Figure 5 could be reduced by reducing the upper limits of the x and y axis to 1.5 and 4, respectively. This would allow the estimates of more countries to be clearly seen. You could consider drawing a circle centred on the (0,0) coordinate with a suitable radius that indicated no meaningful change.

RESPONSE: Thank you for this helpful suggestion. We have adjusted the x- and y-axis limits in Figure 5 as recommended. We chose not to add a reference circle at (0,0) to avoid overcrowding the figure.

5) **Comment 5:** Figure 6. "m" in the facet headings is potentially vague and could be changed to "months", e.g., "12 to 23 months".

RESPONSE: Thank you for pointing this out. We have now updated the facet labels in Figure 6 to use full terms (e.g., "12 to 23 months") to enhance clarity.

6) **Comment 6:** Line 607. Providing the link to the Github repository would be useful.

RESPONSE: Thank you for this suggestion. We have now added the GitHub link to the Data Availability Statement in the manuscript.

- **Revised Manuscript – Data Availability Statement – Line 647:** "All R code used for data analysis and modeling is available on GitHub: https://github.com/nguyenthephuong1/child-immunization-africa"

REVIEWER #3 COMMENTS:

Comments to the Author: Many thanks to the authors for all of their responses and additions to the manuscript. My questions have all been thoroughly addressed. My only remaining comments (see below) are on major comment #6, regarding the characterization of the WUENIC estimates, and major comment #8, where I think that a sentence reflecting on the mean error out of sample validation results would be a helpful addition. Otherwise, I thank the authors for their thorough work and believe that the modifications made in response to reviewers' comments have strengthened the manuscript.

RESPONSES: Thank you for the encouraging feedback and thoughtful suggestions. We are glad the revisions have addressed the earlier concerns and have updated the manuscript to reflect the two remaining points below.

1) **Major comment #6:** Regarding the use of the logit-linear model for time trends: I appreciate the author's explanation and the additional text added to the analysis and limitations section. Within the constraints of this temporal modeling approach, I think that the text is now clear and appropriately conveys the limitations. My one additional comment is that WUENIC's estimates are not technically "administrative" estimates, but instead use a rule-based approach to integrate both survey and administrative (or official country-reported) data, so the language here may need to be slightly revised.

RESPONSES: Thank you for this suggestion. We have revised the relevant sentence to more accurately characterize the WUENIC estimates.

- **Revised Manuscript – Discussion – Line 613:** "While the logit-linear time trend structure allows for interpretable and consistent projections, it may over-smooth local variation in data-sparse settings or diverge from estimates like WUENIC, which incorporate survey, administrative, and country-reported data [5]."

2) **Major comment #8:** Regarding model validation: I appreciate the addition of these analyses - both the out of sample validation and the addition of mean error. My one remaining comment is that it might be useful to add a brief sentence about mean error to the main text. In lines 255-263 the authors comment on the average AD and ranges; a similar statement & interpretation of the mean error results would be useful to the reader. In particular it is interesting that the in-sample mean error is essentially zero, while the out-of-sample mean error (holding out 2017-2019) is consistently positive, ranging from 1.27% to 7.86% for the BYM2_2 model across indicators. This might suggest a tendency towards overestimation in more recent years in the absence of data, which is worth pointing out as a potential limitation (perhaps reflecting some of the additional discussion about coverage stagnation and the limitations of forward-projection using the logit-linear time trend approach). A sentence in the main text reflecting these findings would be helpful for readers' interpretation of the results.

RESPONSES: Thank you for this insightful comment. We have now added the statement of the mean error in the Methods section, and added an interpretation in the Discussion section.

- **Revised Manuscript – Methods – Line 286:** "While in-sample ME was close to zero, out-of-sample ME (holding out 2017–2019) was consistently positive (1.27% to 7.86%), suggesting a possible tendency to overestimate recent progress."

- **Revised Manuscript – Discussion – Line 617:** "The consistently positive out-of-sample ME suggests a tendency to overestimate recent progress, possibly reflecting the stagnation in coverage trends, and should be interpreted with appropriate caution."
